# Identification of Multiple Novel Protein Biomarkers Shed by Human Serous Ovarian Tumors into the Blood of Immunocompromised Mice and Verified in Patient Sera

**DOI:** 10.1371/journal.pone.0060129

**Published:** 2013-03-27

**Authors:** Lynn A. Beer, Huan Wang, Hsin-Yao Tang, Zhijun Cao, Tony Chang-Wong, Janos L. Tanyi, Rugang Zhang, Qin Liu, David W. Speicher

**Affiliations:** 1 Center for Systems and Computational Biology, and Molecular and Cellular Oncogenesis Program, The Wistar Institute, Philadelphia, Pennsylvania, United States of America; 2 Ovarian Cancer Research Center, The University of Pennsylvania, Philadelphia, Pennsylvania, United States of America; 3 Gene Expression and Regulation Program, The Wistar Institute, Philadelphia, Pennsylvania, United States of America; Baylor College of Medicine, United States of America

## Abstract

The most cancer-specific biomarkers in blood are likely to be proteins shed directly by the tumor rather than less specific inflammatory or other host responses. The use of xenograft mouse models together with in-depth proteome analysis for identification of human proteins in the mouse blood is an under-utilized strategy that can clearly identify proteins shed by the tumor. In the current study, 268 human proteins shed into mouse blood from human OVCAR-3 serous tumors were identified based upon human vs. mouse species differences using a four-dimensional plasma proteome fractionation strategy. A multi-step prioritization and verification strategy was subsequently developed to efficiently select some of the most promising biomarkers from this large number of candidates. A key step was parallel analysis of human proteins detected in the tumor supernatant, because substantially greater sequence coverage for many of the human proteins initially detected in the xenograft mouse plasma confirmed assignments as tumor-derived human proteins. Verification of candidate biomarkers in patient sera was facilitated by in-depth, label-free quantitative comparisons of serum pools from patients with ovarian cancer and benign ovarian tumors. The only proteins that advanced to multiple reaction monitoring (MRM) assay development were those that exhibited increases in ovarian cancer patients compared with benign tumor controls. MRM assays were facilely developed for all 11 novel biomarker candidates selected by this process and analysis of larger pools of patient sera suggested that all 11 proteins are promising candidate biomarkers that should be further evaluated on individual patient blood samples.

## Introduction

Epithelial ovarian cancer (EOC) is the fifth-leading cause of cancer-related deaths in women, with a higher fatality-to-case ratio than any other gynecologic malignancy in the United States. [1], [2], [3] A major problem is that greater than two-thirds of EOC cases are diagnosed at advanced stages (Stages 3 or 4), when five-year survival is about 33%. In contrast, when the disease is diagnosed at Stage 1, five-year survival is approximately 90%. [2] CA125 is the best known EOC biomarker; however, 50–60% of early-stage EOC does not express CA125. In addition, while greater than 80% of advanced EOC has elevated CA125, this is not a sufficient diagnosis, as CA125 levels are also elevated in a number of other conditions. [1], [4], [5], [6] Due to the low incidence of ovarian cancer in the general population, the specificity and sensitivity requirements for early screening are quite high, and achieving suitable performance is likely to require a panel of biomarkers superior to most existing biomarkers. [2], [7], [8], [9] Additional biomarkers, either instead of or in conjunction with CA125, are needed for predicting clinical outcome, stratifying therapeutic options, monitoring response to therapy, and detecting reoccurrence of the disease.

Although proteomic technologies have improved dramatically, discovering novel blood biomarkers for cancers remains formidable due to the vast complexity of the plasma proteome and the likelihood that any tumor-specific proteins will be present at very low abundance. In addition, comparison of patient and control serum or plasma to discover biomarkers is complicated by the fact that cancers and other conditions induce inflammatory responses involving changes in abundance of multiple blood proteins, and these changes are not very specific to a single disease. [10] Identifying cancer-specific changes in the context of this great complexity and inflammation-induced variability is very difficult when patient serum samples are directly analyzed to discover new biomarkers using proteomics. For this reason, many investigators have turned to alternative strategies for initial discovery of candidate biomarkers, including proteome analysis of: EOC cell lines, cell surface proteins in EOC cell lines, proteins shed by these cells into the media (the secretome), and patient ascites. [11], [12], [13], [14], [15], [16] While all of these methods identify many proteins associated with EOC, a critical missing factor is that it is not apparent which of these proteins will migrate into the blood and be potential EOC serum/plasma biomarkers. Furthermore, changes in abundance levels of a protein in the tumor tissue do not necessarily correlate with their abundance levels in the blood.

The primary experimental systems where migration into the blood is assured are mouse models. Hence, one approach is to identity quantitative differences in plasma or serum of genetically engineered mice bearing murine ovarian tumors compared with appropriate controls. [17] While this model provides more consistent genetic and environmental backgrounds compared with patients, the complication of sorting protein changes caused by inflammatory host responses from proteins shed by the tumor persists. An important under-utilized alternative that circumvents the host response problem is xenograft mouse models, where in-depth proteome analysis can identify human proteins shed by the tumor into the murine blood based upon species differences in peptide sequences found in serum or plasma. Another advantage of the xenograft mouse model system, like genetically engineered mice, is a higher tumor-to-blood-volume ratio compared with patients, as well as homogeneous genetic backgrounds, environment, and diet to minimize confounding factors.

We recently identified 106 human proteins shed by a human endometrial ovarian cancer cell line (TOV-112D) into the blood of SCID mice using an in-depth 4D proteome analysis of this xenografted mouse serum. Specifically, serum was depleted of the three most abundant serum proteins followed by microscale solution isoelectrofocusing (MicroSol IEF), SDS-PAGE, and LC-MS/MS. [18] Furthermore, pilot validation of selected candidate biomarkers demonstrated that some of these proteins could be detected in human serum using multiple reaction monitoring (MRM) analysis–and three tested biomarkers were shown to be significantly elevated in cancer patients compared with normal donors. [18] Although that study identified several new biomarkers, the verification and initial validation steps were not very efficient. The success rate of establishing MRM assays for targeted candidate biomarkers using advanced EOC patient serum pools was less than 50%, and only about half of the proteins quantified using these MRM assays exhibited elevated levels in pilot analyses of advanced EOC patients using pools of patient sera ( [18] and data not shown). Hence, overall, only about 20% of the TOV-112D candidate biomarkers selected for verification and initial validation in patients resulted in successful MRM assays that exhibited elevated levels in EOC in a small pilot analysis.

The current study was designed to improve ovarian cancer biomarker discovery using the xenograft mouse model system, improve the efficiency of MRM assay development and preliminary verification of biomarker candidates, and identify candidates that may include biomarkers with some degree of specificity for the serous ovarian cancer subtype. High grade serous ovarian cancers are very aggressive and account for most deaths from EOC, although they only constitute approximately half of EOC cases. Plasma from SCID mice bearing OVCAR-3 serous ovarian tumors was analyzed using an optimized 4-D plasma proteome analysis method, which resulted in identification of 268 human proteins based on two or more peptides. Parallel analysis of supernatants from the serous tumor excised from the SCID mice and incubated briefly in cell culture media increased the sequence coverage for many of the human proteins, thereby both confirming these proteins were human and providing more proteotypic peptides for MRM assay development. Selected high-priority candidate biomarkers were then compared to a dataset from a label-free quantitative comparison of serum pools from cancer patients with advanced-stage EOC and benign controls. Finally, candidate biomarkers observed to be elevated in pooled sera from advanced EOC cancer patients in the label-free analysis were selected for multiplexed assay development using targeted mass spectrometry analysis with the multiple reaction monitoring (MRM) method. MRM analysis is an alternative mass spectrometry approach utilizing the discriminating power of triple quadropole mass spectrometers, or their equivalent, to select and quantify a series of specific analytes and associated fragment ions. MRM enables rapid, quantitative measures of proteins in complex mixtures such as plasma and serum, without a dependency on the generation of antibodies or immunoassays. [19], [20] Overall, the strategy used in this study proved to be highly efficient, as 100% of the proteins selected for MRM assay development resulted in successful assay development and showed elevated levels in serum pools of advanced EOC patients compared with an approximately 20% success rate in earlier studies that attempted to quantify and verify TOV-112D candidate biomarkers.

## Materials and Methods

### Reagents

Molecular-biology-grade ethanol (200 proof); LC-MS-grade formic acid; sodium phosphate monobasic; *N*,*N*-dimethylacrylamide (DMA), ammonium bicarbonate; and iodoacetamide were purchased from Sigma-Aldrich (St. Louis, MO). Sodium dodecyl sulfate (SDS), 2-mercaptoethanol, and Tris were purchased from Bio-Rad (Hercules, CA). ZOOM focusing buffers and thiourea were obtained from Invitrogen (Carlsbad, CA). PlusOne reagents dithiothreitol (DTT), 3-[(3-cholamidopropyl)dimethylammonio]-1-propanesulfonate (CHAPS), and urea were purchased from GE Healthcare (Piscataway, NJ). HPLC-grade acetonitrile was purchased from Thomas Scientific (Swedesboro, NJ). Tris(2-carboxyethyl)phosphine (TCEP) was obtained from Pierce (Rockford, IL), and sequencing-grade modified trypsin was purchased from Promega (Madison, WI).

### Cell Culture

The human EOC serous cell line OVCAR-3 was obtained from the American Type Culture Collection (ATCC, Manassas, VA). The cells were maintained in a 37°C incubator with a 5% CO_2_–95% air atmosphere in RPMI-1640 medium (ATCC) supplemented with 10% fetal calf serum.

### Ovarian Cancer Growth *in vivo*

This study was carried out in accordance with the recommendations in the Guide for the Care and use of Laboratory Animals of the National Institutes of Health under protocol #111959 using severe combined immunodeficiency (SCID) mice. The study protocol was reviewed and approved by The Wistar Institute's Institutional Animal Care and Use Committee (IACUC). All efforts were made to minimize suffering.

Nine SCID mice were injected subcutaneously in the flank with 50 µl of OVCAR-3 cells (2×10^6^) mixed 1∶1 with 50 µL Matrigel (BD Biosciences, San Jose, CA). Tumors were allowed to grow until final tumor size was estimated to be at least 1 cm^3^ using calipers, but less than 10% of body weight. Blood was collected, mice were euthanized, and tumors were removed at 12 weeks post-injection. Necrosis of tumor tissue was assessed by microscopic inspection of hematoxylin and eosin (H&E) stained, paraffin-embedded sections (5 µm).

### SCID Mouse Plasma

Blood was collected from SCID mice containing visible OVCAR-3 tumors (see above section) by cardiac puncture under anesthesia into Microtainer 0.5 mL K_2_ EDTA Blood Collection Tubes (Becton Dickinson, Franklin Lakes, NJ). The tubes were centrifuged for 3 min at room temperature, and aliquots of plasma from individual mice were snap-frozen and stored at −80°C. Plasma from the four mice that contained the largest, minimally neurotic tumors were subsequently thawed and pooled to average variations among individual mice. The pooled plasma was re-aliquoted, snap-frozen, and stored at −80°C until analysis using the 4-D fractionation method (immunoaffinity depletion/MicroSol IEF/SDS gel and trypsin digestion/LC-MS/MS). Total protein concentration of the pooled plasma was measured using a BCA Protein Assay (Pierce).

### Tumor Supernatant Isolation

Immediately after removing tumors from eight of the nine SCID mice bearing the largest OVCAR-3 tumors, a section of fresh ovarian tumor tissue from each mouse was cut into small pieces (2–3 mm^3^), placed in the upper chamber of a 5 µm PVDF microcentrifuge filter (Millipore, Billerica, MA), and washed three times with 400 µL of PBS for 1 min each. The tissue sections were then transferred to the upper chamber of a 0.22 µm PVDF microcentrifuge filter and incubated in 400 µL of serum-free RPMI-1640 medium for 2 h in 5% CO_2_, 95% air at 37°C. After incubation, the supernatant (conditioned media) was recovered by centrifugation, then frozen and stored at −80°C until needed. For tumor supernatant analysis, 500 µL aliquots of conditioned media from tumors from four mice were thawed, pooled, and concentrated to ∼30 µL by ultrafiltration using a 10 K MWCO concentration unit. Membrane rinses using 1% SDS, 50 mM Tris, pH 8.5 were combined with the concentrated sample to maximize protein recovery.

### Human Serum

Human sera from normal subjects and patients with benign ovarian tumors, early-stage ovarian cancer, and late-stage ovarian cancer were collected before clinical treatment at approximately the time of diagnosis and prior to surgery as previously described. [18] Descriptions of the patients, healthy controls, and pooling strategies are summarized in **Table S2**. All research in this study involving human specimens was conducted under The Wistar Institute's Institutional Review Board (IRB) approved protocols, #2109171, #EX2110012, and #2602221 and in accordance with Health Insurance Portability and Accountability Act (HIPAA) requirements. All human samples were derived from subjects with informed written consent. Data were analyzed anonymously.

### Immunoaffinity Removal of Major Blood Proteins

The pooled mouse plasma was depleted using a 4.6×100 mm MARS Mouse-3 HPLC column (Agilent Technologies, Wilmington, DE), essentially as previously described, [18] with the exception that a total of 400 µL of pooled plasma was diluted five-fold with equilibration buffer, filtered through a 0.22 µm microcentrifuge filter, and applied to the antibody column in eight serial injections of 250 µL per depletion.

Human serum samples (typically 30–60 µL) were depleted of the 20-most-abundant serum proteins using a ProteoPrep20 Immunodepletion Column (Sigma), as described previously. [21].

### MicroSol IEF Fractionation

Immunodepleted and concentrated mouse plasma (2.2 mg) was fractionated by MicroSol IEF as previously described, [18], [22], [23] using a ZOOM-IEF fractionator (Invitrogen) where the separation chambers were defined by immobilized gel membranes having pH values of 3.0, 4.6, 5.4, 6.2, and 12.0, respectively.

### SDS-PAGE/in-gel Trypsin Digestion

After evaluation of the MicroSol IEF separation on analytical SDS gels, concentrated samples were loaded onto multiple lanes of pre-cast 12% Bis-Tris NuPAGE gels (Invitrogen) and separated for discrete distances (1 or 4 cm). The concentrated tumor supernatants from both pools were loaded onto multiple lanes of pre-cast 12% Bis-Tris NuPAGE gels (Invitrogen) and separated for 6 cm. Gels were stained with Colloidal Blue (Invitrogen), each gel lane was sliced into uniform 1 mm slices, and corresponding slices from triplicate lanes were combined in a single well of a 96-well pierced digestion plate (Bio-Machines, Inc., Carrboro, NC) and digested overnight with 0.02 µg/µL of modified trypsin, as previously described. [21], [24].

### LC-MS/MS

Tryptic digests were analyzed using an LTQ-Orbitrap XL mass spectrometer (Thermo Scientific, Waltham, MA) interfaced with a Nano-ACQUITY UPLC system (Waters, Milford, MA), as described previously. [21] For each tryptic digest, 8 µL was injected onto a UPLC Symmetry trap column (180 µm i.d. × 2 cm packed with 5 µm C18 resin; Waters), and tryptic peptides were separated by RP-HPLC on a BEH C18 nanocapillary analytical column (75 µm i.d. × 25 cm, 1.7 µm particle size; Waters). The mass spectrometer was set to scan *m/z* from 400 to 2000. The full MS scan was collected at 60,000 resolution in the Orbitrap in profile mode followed by data-dependant MS/MS scans on the six most abundant ions exceeding a minimum threshold of 1000 collected in the linear trap. Monoisotopic precursor selection was enabled and charge-state screening was enabled to reject z = 1 ions. Ions subjected to MS/MS were excluded from repeated analysis for 60 s.

### Data Processing

MS/MS spectra were extracted and searched using the SEQUEST algorithm (v. 28, rev. 13, University of Washington, Seattle, WA) in Bioworks (v. 3.3.1, Thermo Scientific) against a combined human and mouse UniRef100 protein sequence database (v. June 20,2011) to which commonly observed “contaminants” (trypsin, keratins, etc.) were added. A decoy database was produced by reversing the protein sequence of each database entry, and the entire reversed database was appended in front of the forward human and mouse databases, respectively. Spectra were searched with a partial tryptic constraint of up to two missed cleavages, 100 ppm precursor mass tolerance, 1 Da fragment ion mass tolerance, static modification of cys (+99.06840 for samples alkylated with DMA or +57.0215 Da for samples alkylated with IAM), and variable modification of methionine (+15.9949). The use of a partial tryptic constraint and 100 ppm precursor tolerance for the database search had recently been shown to enhance depth of analysis for serum and plasma proteomes. [25] Consensus protein lists were created using DTASelect (v. 2,0, licensed from Scripps Research Institute, La Jolla, CA) and the following filters were applied: full tryptic constraint, mass accuracy ≤10 ppm, and ΔCn ≥0.05. [25] FDR was estimated from the ratio of unique peptides matching reverse sequences to the number of unique peptides matching forward sequences. Non-redundant peptide totals derived from DTASelect and used for FDR calculations include variable modifications and different charge states as separate peptides. Different charge states and variable modifications of methionine oxidation were collapsed into a single unique peptide count, and peptides shared among multiple proteins were assigned to the protein having the highest sequence coverage, as previously described. [18].

Proteins identified in the database search were sorted into “human,” “mouse,” or “indistinguishable” based upon their species-specific sequences, as previously described. [18] To confirm species-specific assignments, putative uniquely human and mouse sequences were searched against the mouse and human UniRef100 databases (v. 6/20/11), respectively, using BLAST. Keratins and other presumed contaminants were removed from the entire dataset.

### Label-free Quantitation of Patient Serum Pools

To determine whether candidate biomarkers could be detected in ovarian cancer patient sera, in-depth discovery mode analyses of patient serum pools were conducted followed by global label-free quantitative comparisons. One pool of serum from benign patients and three pools of advanced ovarian cancer patient serum samples were made as described in **Table S2**. Pools were immunodepleted and separated on a 1 D SDS gel, each serum proteome was separated into 40 fractions, and each slice was digested with trypsin and analyzed by LC-MS/MS using a 4 h gradient at 200 nL/min consisting of 5–28% B over 168 min, 28–50% B over 51.5 min, 50–80% B over 5 min, and 80% B for 4.5 min, before returning to 5% B over 0.5 min. A short blank gradient was run in between samples to minimize carryover. Full-MS and LC-MS/MS data from fractions 17–32, which encompassed the 8–50 kDa region of the gel, were analyzed using Rosetta Elucidator software (version 3.3, Rosetta Biosoftware, Seattle, WA) to compare peptide signal intensities in full MS scans. Based on peptide elution profiles and ion signal density, data for this label-free comparison was trimmed to 16–200 min. Retention time (RT) alignment, feature identification (discrete ion signals), feature extraction, and protein identifications were performed by the Elucidator system as previously described. [21], [26].

### Label-free Multiple Reaction Monitoring

MRM experiments were performed on a 5500 QTRAP hybrid triple quadrupole/linear ion trap mass spectrometer (AB Sciex, Foster City, CA) interfaced with a Nano-ACQUITY UPLC system with the column heater maintained at 45°C. Tryptic digests were injected using the partial loop injection mode onto a UPLC Symmetry trap column (180 µm i.d. × 2 cm packed with 5 µm C18 resin), and then separated by RP-HPLC on a BEH C18 nanocapillary analytical column (75 mm i.d. × 25 cm, 1.7 mm particle size; Waters). Chromatography was performed with solvent A, consisting of Milli-Q water with 0.1% formic acid, and solvent B, as acetonitrile with 0.1% formic acid. Peptides and transitions used for quantitation were selected from discovery results (typically the mouse tumor supernatant) and further verified by MRM-initiated detection and sequencing (MIDAS) using the 5500 QTRAP mass spectrometer. MIDAS experiments were performed at 200 nL/min with a 77-min gradient consisting of 5–28% B over 42 min, 28–50% B over 25.5 min, 50–80% B over 5 min, and 80% B for 4.5 min, before returning to 5% B over 0.5 min. To increase throughput, after optimal peptides and transitions were established, label-free MRM assays were performed with a 41-min gradient, in which peptides were eluted at 400 nL/min for 5–35% B over 38 min and 35% B for 3 min, before returning to 5% B over 0.5 min.

MRM data were acquired with a spray voltage of 3300 V, curtain gas of 20 p.s.i., nebulizer gas of 10 p.s.i., interface heater temperature of 150°C, and a pause time of 3 ms. Multiple MRM transitions were monitored per peptide at unit resolution in both Q1 and Q3 quadrupoles to maximize specificity. Scheduled MRM was used to reduce the number of concurrent transitions and maximize the dwell time for each transition. The detection window was set at 2 min, and the target scan time was set at 1.8 s. Data analysis was performed using Skyline v.1.2. [27] The transition with the strongest signal for each peptide was used for quantification unless interference from the matrix was observed. In these cases, another transition free of interference was chosen for quantification.

## Results and Discussion

### Overview of Discovery, Prioritization and Verification of EOC Biomarkers Using a Xenograft Serous EOC Mouse Model

**Figure 1:**
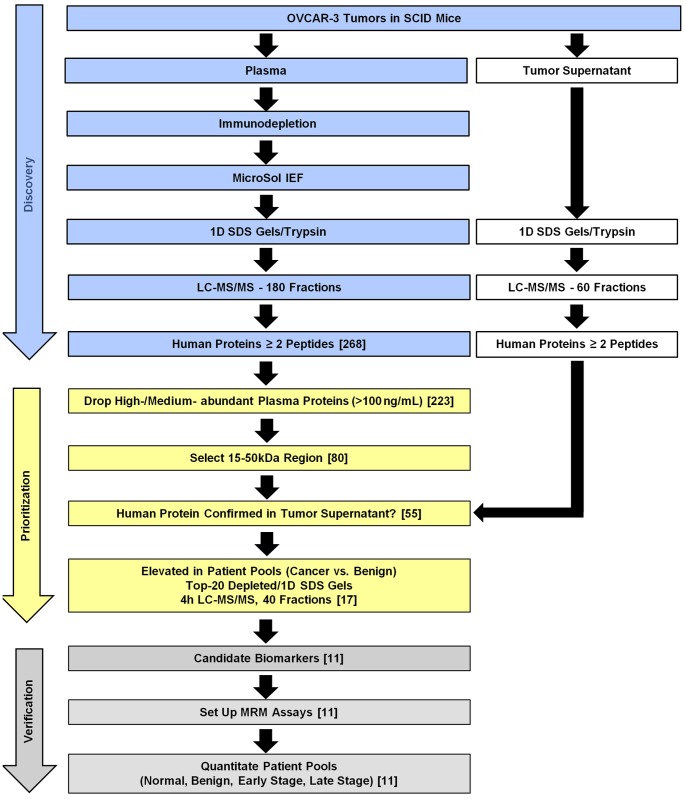
Scheme for ovarian cancer biomarker discovery and efficient verification using a xenograft mouse model. Candidate biomarkers were discovered in the xenograft mouse plasma using a 4D plasma proteome profiling method. Parallel analysis of the tumor supernatant was used to confirm human protein identifications and expand sequence coverage. A multi-step prioritization method ensured that only those proteins detectable in advanced EOC patient sera at elevated levels advanced to MRM assay development and verification in larger pools of patient sera.

The strategies used to improve ovarian cancer biomarker discovery using the xenograft mouse model system, select high priority candidate biomarkers, and improve the efficiency of MRM assay development and biomarker verification are outlined in **Figure 1**. In the discovery phase, OVCAR-3, an established human serous cell line, was grown in SCID mice. Xenograft mouse plasma pooled from four mice with the largest tumors was subjected to extensive fractionation (180 fractions) using a 4 D plasma proteome separation method developed in our laboratory which consists of immunoaffinity depletion of major serum proteins, MicroSol IEF, 1D SDS-PAGE, and LC-MS/MS. [24] Representative analytical and preparative 1D SDS gels showing MicroSol IEF fractions prior to LC-MS/MS analysis can be found in **Figure S1 B and C**, respectively. Human proteins identified in the plasma by at least two peptides, at least one of which was uniquely human, were prioritized and verified as illustrated and described in further detail below. Although this biomarker candidate discovery study used a cell line representative of a late stage tumor, our working hypothesis is that the best cancer biomarkers will be shed by the tumor into the blood and will correlate with tumor size. These biomarkers will ideally be detectable in serum or plasma at higher levels than in control subjects, even when the tumors are small, and the levels of these biomarkers will increase as the tumor grows. By utilizing the xenograft mouse model and identifying human proteins, we are assured that the candidate biomarkers are derived from the tumor and shed into the blood, at least in this model system.

### Analyses of the Xenograft Mouse Plasma Proteome and Corresponding Tumor Supernatants

**Figure 2:**
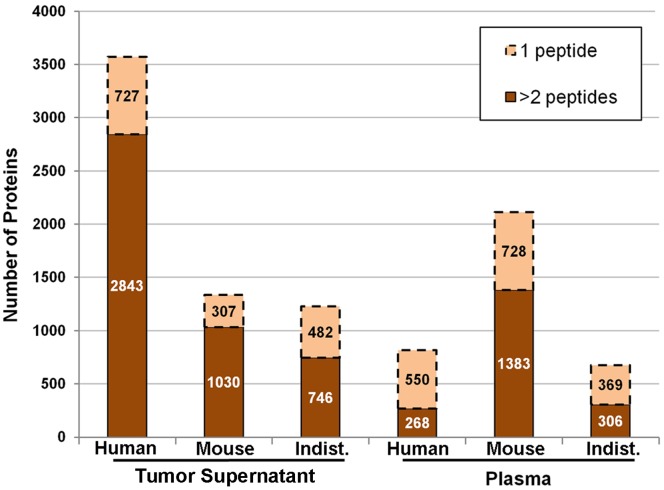
Proteins identified in the xenograft plasma and tumor supernatant. The numbers of unique (nonredundant) proteins identified in the OVCAR-3 xenograft tumor supernatant and mouse plasma are shown after sorting proteins based on species classification where at least one peptide was uniquely human, uniquely mouse, or indistinguishable (Indist.), that is, all detected peptides were common to human and mouse homologs.

Plasma from four mice containing OVCAR-3 tumors that were at least 1 cm^3^ was pooled and analyzed using the 4D method described above. Fractionation using MicroSol IEF and 1D SDS gels yielded 180 fractions and subsequent analysis of these fractions by LC-MS/MS produced more than 1.1 million spectra, which were searched against a combined human and mouse database. A total of 3647 non-redundant human and mouse proteins were initially identified by 22,890 peptides at a peptide FDR of 5.7% for all proteins, and a 0.5% FDR for protein identifications with two or more peptides. After species classification, 268 human proteins were identified by two or more peptides and an additional 550 by a single peptide (**Figure 2**). Because the FDR was considerably lower for proteins with ≥2 peptides in both the plasma and tumor supernatant samples, we only considered proteins having two or more peptide identifications for downstream analyses.

Due to the difficulty of detecting low abundance human proteins in the mouse plasma, tumor supernatants from the same batch of SCID mice with OVCAR-3 tumors were analyzed to attempt to achieve more extensive sequence coverage of human proteins detected in the mouse plasma. Concentrated tumor supernatants were separated on 1D SDS gels, each lane was sliced into 60 uniform fractions (**Figure S1A**), and each sample was digested with trypsin followed by LC-MS/MS analysis resulting in 487,076 MS/MS spectra, which were searched against a combined mouse and human database. A total of 6066 unique proteins were identified from 46,111 peptides at a peptide FDR of 0.9%. Eliminating single peptide proteins resulted in 4619 unique protein entries, with a peptide FDR of 0.03%. This list of high-confidence proteins (≥2 peptides) identified from the combined human and mouse dataset is listed in **Table S1**. This complete dataset was divided into “human” and “mouse” based upon the presence of at least one peptide unique to that species, while “indistinguishable” proteins contained only peptides common to both species. A total of 2843 human proteins were identified by two or more peptides, and an additional 727 human proteins were identified by single peptides (**Figure 2**).

Interestingly, the tumor supernatant dataset provided a much greater depth of analysis both in terms of total proteins identified and sequence coverage of most proteins, despite the less extensive fractionation used. Also, the proportion of total identified proteins that could be assigned as human was far higher in the tumor supernatant. In part, this was expected because the plasma analysis was dominated by detection of high and medium abundance mouse plasma proteins. However, it also indicates that the contribution of mouse cells in the tumor, including fibroblasts and vascular cells, was relatively minor compared to shedding of proteins by the human tumor cells.

### The Tumor Supernatant Increases Sequence Coverage of Human Proteins Detected in the Xenograft Mouse Plasma

Over 2800 human proteins from the tumor were identified in the supernatant and this dataset is a possible source of additional plasma biomarkers for ovarian cancer. However, unless the proteins were also detected in the mouse plasma, there is no assurance that proteins observed in the supernatant would be shed into and be detectable in the blood. Furthermore, the plasma analysis had already identified nearly 300 human proteins, which exceeds the number of proteins that could be feasibly tested in human serum. Hence, in this study, the larger tumor supernatant dataset was only used to confirm the “human” assignment of proteins identified in the mouse plasma, although this large dataset almost certainly contains additional potential plasma biomarkers that could be explored in future studies. Confirmation of apparent human proteins in the mouse plasma is important because many proteins were assigned as human based upon the detection of one human peptide and one or more peptides with sequences common to both species. A few of these apparent human proteins may be false positives while others could represent unreported mouse polymorphisms, mouse sequences not reported in the database, etc. Also, some identified proteins represent a protein family but the peptides identified in the plasma do not unambiguously define a unique isoform. Therefore, for most of the human proteins identified in the plasma, the tumor supernatant dataset improved the confidence of species assignment and in some cases more clearly defined family member(s) present in the xenograft plasma samples by confirming the original peptide and protein identifications and, in most cases, providing more extensive peptide coverage.

**Figure 3:**
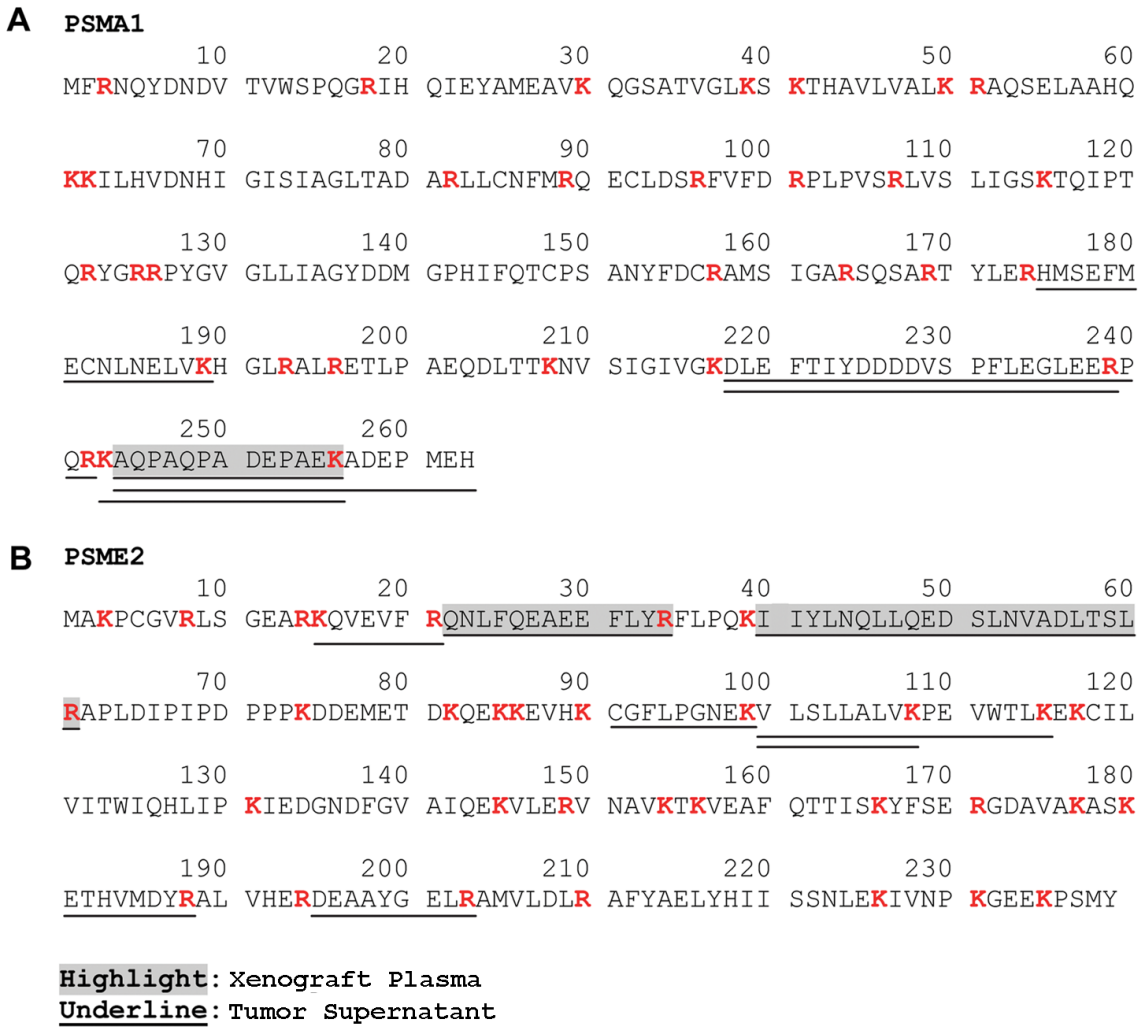
Sequence coverage for selected human proteins from the xenograft plasma and tumor supernatants. Examples of candidate biomarkers, PSMA1 and PSME2, where increased sequence coverage is obtained from analysis of the tumor supernatant (underlined peptides) compared to the xenograft plasma (grey highlight). Tryptic sites (K or R) are indicated in red.

Examples of using the tumor supernatant data to expand the utility of data from the xenograft plasma are shown in **Figure 3**. PSMA1 (**Figure 3A**) was identified by a total of 10 peptides in the mouse plasma, but only a single one of these was a uniquely human peptide. This protein could have been de-prioritized because of its high homology to a mouse counterpart and the possibility that the single uniquely human peptide in the plasma might have been a false positive identification or unknown mouse sequence variant. However, the tumor supernatant identified an additional five peptides that were uniquely human, thus increasing the confidence of the species assignment for the original plasma identification. **Figure 3B** shows PSME2, a protein identified by two uniquely human peptides in the plasma dataset, and therefore the species assignment as human is well supported. But, the tumor supernatant analysis identified six additional human peptides, thereby providing more proteotypic peptides for setting up MRM assays.

The tumor supernatant and plasma datasets were compared to a study by Pitteri *et al.* that identified candidate biomarkers by comparing a genetically engineered mouse model and secretomes of ovarian cancer cells. [17] That study validated eight proteins found to be at higher abundance levels in ovarian cancer patients’ plasma, and they also described identification of an additional nine proteins previously identified as ovarian cancer plasma biomarkers. Of the 17 candidate markers described by Pitteri *et al.*, we identified eight proteins (CTSB, FASN, IGFBP2, LCN2, MIF, THBS1, WFDC2, and NRCAM) in our tumor supernatant analysis, and three proteins (FASN, IGFBP2, and LCN1) in the high-confidence xenograft plasma dataset.

We also compared the results from the current study using OVCAR-3 cells, a serous EOC cell line to an earlier xenograft mouse study using an endometrioid EOC cell line (TOV-112D) where we identified three new biomarkers of ovarian cancer that could distinguish cancer patients from normal individuals. [18] These three biomarkers, CLIC1, CTSD, and PRDX6, were all identified in the current study.

Overall, these comparisons show that different biomarker discovery strategies result in detection of overlapping, but non-identical sets of biomarkers. These data also demonstrate that analysis of the tumor supernatant in parallel with xenograft mouse plasma is useful for confirming candidate biomarkers detected in xenograft mouse plasma.

### Prioritization and Selection of Candidate Biomarkers for Verification Using Patient Sera

Efficient methods for selecting the best candidate biomarkers and economically verifying them in serum or plasma of EOC patients are needed because, some, but not all proteins shed by EOC tumors into blood are expected to be good biomarkers of the disease. Furthermore, some proteins detected in the xenograft mouse model may not be detectable in human blood using current methods either because the concentration in human blood is below detection limits of available assays or because in some cases the shedding may be unique to the mouse model. As noted above, when we evaluated a panel of candidate biomarkers from the TOV-112D xenograft mice tumors, the overall success in setting up MRM assays and demonstrating elevated levels of the targeted biomarker in EOC patient sera was only about 20%. Hence, an important challenge is to develop appropriate methods for more efficient triaging of candidate biomarkers and evaluating them in serum of EOC patients.

The xenograft plasma proteome was prioritized starting with the 268 human proteins identified by two or more peptides (**Figure 2**). This dataset was further refined by removing a few trypsin and keratin contaminants that were missed at the initial contaminant-removal step due to ambiguous protein descriptions or isoform differences. In addition, proteins known to be in normal human plasma at medium- to high-abundant levels (>100 ng/mL [28], [29]) and hemoglobins were removed. Such proteins were not considered to be viable candidate biomarkers because the contribution of shedding from a small tumor is unlikely to be discernible above the normal variation of that protein in the general population. For example, if a protein is normally in the plasma of unaffected individuals in the 1–5 µg/mL range and the protein is also shed by a typical ovarian tumor, which contributes another 50 ng/mL of that protein into the plasma, the contribution from the tumor is not detectable above normal variation.

### Verification of Candidate Biomarkers in the 15–50 kDa Region Using the Tumor Supernatant and Label-free Discovery Proteomics Analysis of Patient Pools

**Figure 4:**
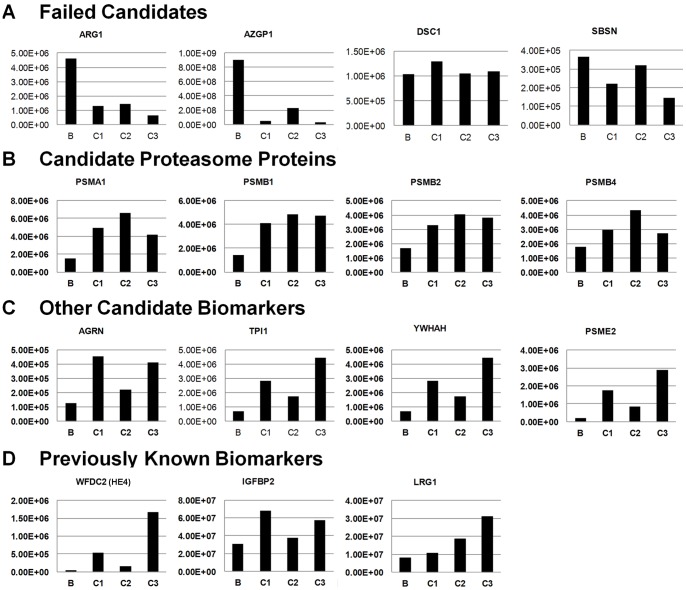
Quantitative comparisons of candidate biomarkers using label-free discovery mode LC-MS/MS analysis of patient serum pools. Summed protein intensities from a Rosetta Elucidator label-free analysis are shown for benign (B, n = 9) and three different late-stage ovarian cancer pools (C1, n = 9; C2, n = 9; C3, n = 5). (A) Representative proteins that failed this screen because intensities were lower or showed no difference in cancer pools compared with benign disease. (B) Four different isoforms of the proteasome complex selected for further validation because they showed elevated levels in all cancer pools compared with benign sera. (C) Additional representative proteins selected for further validation because they showed elevated levels in all cancer pools compared with benign sera. (D) Three biomarkers previously reported by others and re-discovered in the current study (see **Table 1**).

Candidate biomarkers in the 15–50 kDa region of the gel were selected for further prioritization and verification because this was the region of the gel that contained the largest density of human proteins in the xenograft plasma analysis. By focusing on a discrete region of the gel we could increase the subsequent throughput of MRM assays by minimizing the number of fractions that need to be analyzed to quantitate the targeted group of candidate biomarkers. Candidates in this region that were identified with at least the same number of peptides in the tumor supernatant were considered further (**Figure 1** and **Table 1**). These candidate biomarkers were then compared to data from an in-depth label-free quantitative comparison of pools of patient sera using a 4 h gradient for the LC-MS/MS runs. One serum pool from patients with benign tumors (pool B, n = 9), was compared to three serum pools from patients with advanced ovarian cancer (pool C1: stage 3, n = 9; pool C2: stage 3, n = 9; pool C3: stage 4, n = 5). Descriptions of the patients and the sample pooling strategy are provided in **Table S2**. Acquisition of full MS and data-dependent MS/MS scans were identical to those described for the xenograft proteome analyses, with the exception that ions subjected to MS/MS were excluded from repeated analysis for 180 s. Xenograft plasma candidate biomarkers that could be detected in these human serum pools were quantitatively compared across pools using peptide ion signal intensities from the Rosetta Elucidator System's peptide report results. Peptides were grouped into consensus proteins by protein description and peptide intensities were summed for each protein. The criteria for selecting candidates for further validation were proteins that showed increases in all three cancer pools compared with the benign serum, and where the average intensity of the three cancer pools was at least 1.7 times that of the benign serum pool (**Figure 4**). Candidates whose protein intensities did not increase in cancer were not considered to be good biomarkers (**Figure 4A**). For example, ARG1 and AZGP1 failed because they showed decreases in cancer relative to benign disease–a trend that does not correlate with cancer burden, and DSC1 and SBSN were not further considered because the benign and cancer pools exhibited similar levels of these proteins. **Figure 4B** shows a number of proteasome subunits that exhibited increases in ovarian cancer. The proteasome complex is responsible for degradation of proteins crucial to cell cycle regulation and apoptosis and has been recognized as a potential target for cancer therapy. [30] Specific proteasome subunits, including PSMB2 and PSMB4, have been identified as upregulated in gene expression profiles of ovarian carcinomas. [31], [32] Interestingly, circulating intact proteasomes have recently been reported to correlate with EOC, [33] but the assay used in that study did not distinguish specific isoforms or quantify subunits that may not have been in intact proteasomes. **Figure 4C** shows representative additional promising candidates. One candidate, AGRN, is a 215 kDa protein previously identified as being upregulated in ovarian cancer tissue samples compared with normal and non-ovarian tissue samples, [34] but it has not previously been reported to be a serum biomarker for EOC. In this study, it was identified by SDS-PAGE as both the intact 215 kDa protein and as a 43 kDa fragment from the C-terminal region of the protein in the tumor supernatant. In contrast, only the 43 kDa fragment which is presumably a proteolytic fragment produced by proteolysis either in the tumor or in the blood was detected in the xenograft plasma. The peptides quantitated in **Figure 4C** belong to the fragment and correlate with ovarian cancer in this experiment. Additionally, six proteins, including ANXA1, FABP5, PSMB3, PSMB6, PSMB8, and PSMB9 were deprioritized because they were either closely related to other selected biomarkers, or based on biology were considered unlikely to be specific to ovarian cancer. Finally, three biomarkers previously reported by others were detected in either the xenograft mouse plasma or tumor supernatant or both (**Table 1**) and were within the targeted 15–50 kDa region of the gel. These known biomarkers, which included HE4 (WFDC2) one of the two FDA approved ovarian cancer biomarkers, were included in our prioritization and verification analyses as known biomarker references. As expected, these three proteins exhibited increased levels in the cancer pools compared with the benign pool (**Figure 4D**). CA125 was not identified in the xenograft plasma, presumably due to its extensive glycosylation and low concentration as well as the high complexity of plasma; however, it was identified in the tumor supernatant by its alternative protein name ‘Mucin-16′ (**Table S2**).

**Table 1:** Candidate biomarkers for validation in patient serum pools.

Gene Name	Protein Description	# Peptides^a^(Supernatant)	# Peptides^a^(Plasma)
***B. Previously reported biomarkers re-identified in this study***			
IGFBP2	Insulin-like growth factor-binding protein 2 [38]	9/5	5/4
WFDC2 (HE4)	WAP four-disulfide core domain protein 2 [39]	2/0	0
LRG1	Leucine-rich alpha-2-glycoprotein [40]	0	1/1
***A. Novel candidates identified from xenograft mouse plasma and verified in tumor supernatant***			
AGRN	Agrin and agrin fragments	61/9	5/1
PSME2	Proteasome activator complex subunit 2	8/11	2/8
TPI1	Triosephosphate isomerase	7/18	4/10
DDAH2	N(G),N(G)-dimethylarginine dimethylaminohydrolase 2	7/8	1/4
GM2A	GM2 ganglioside activator protein (GM2A), mRNA	3/0	3/0
YWHAB	14-3-3 protein beta/alpha	2/8	1/12
YWHAH	14-3-3 protein eta	2/9	1/7
PSMA1	Proteasome subunit alpha type-1	6/14	1/10
PSMB1	Proteasome subunit beta type-1	3/9	1/6
PSMB2	Proteasome subunit beta type-2	1/10	1/6
PSMB4	Proteasome subunit beta type-4	3/5	2/5

a Number of peptides that are uniquely human/number of peptides common to mouse and human homolog.

### Potential Correlation of Biomarkers with Gene Expression

**Figure 5:**
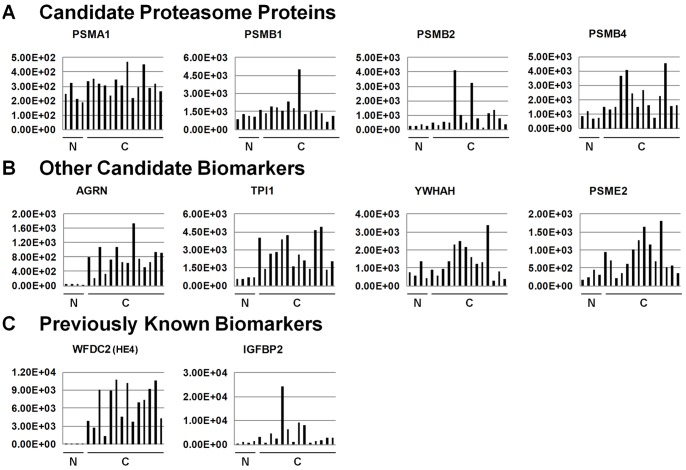
Gene expression of candidate biomarkers in ovarian tissues. Gene expression levels for normal ovary tissue (N; n = 4) and papillary serous ovarian carcinoma primary tumor samples (C, n = 14) are shown. (A) Representative candidate proteasome proteins. (B) Representative novel proteins identified in the xenograft mouse plasma. (C) Two previously reported ovarian cancer biomarkers that were identified in the current dataset. Microarray hybridization data were processed and scaled as previously described. [36], [37] Data were extracted from www.BioGPS.org.

To evaluate whether gene expression in ovarian tumor tissues could be a useful indicator of whether a protein is promising serum biomarker, we queried our candidate biomarkers from **Table 1** against published microarray hybridization data using BioGPS, a centralized gene portal of combined gene annotation resources. [35] Specifically, gene expression levels for normal ovarian tissue (n = 4) and papillary serous ovarian carcinoma primary tumor samples (n = 14) [36], [37] were extracted for each of the candidate markers listed in **Table 1**. **Figure 5** shows examples of gene expression patterns for some of our novel and two known ovarian cancer biomarkers. These gene expression levels can be compared with the observed levels of these same proteins in the benign and EOC patient serum pools (**Figure 4**). Some proteins show similar trends; that is, elevated levels in both the serum and tumor tissue levels for EOC, including AGRN, TPI1, and HE4. However, other proteins do not exhibit much similarity between tissue expression and serum levels. For proteins such as YWHAH and PSME2, gene expression levels overlap extensively between normal and cancer tissue, but the serum levels of these proteins show similar patterns to those for AGRN and TPI1. Also, PSMA1 exhibits similar expression levels between normal and EOC tumor samples but much higher levels in the serum of EOC patients compared with benign tumor controls. Interestingly, at the gene expression level, each of the four illustrated proteasome subunits exhibits differing expression patterns at the cancer tissue level but all four subunits show elevated serum levels in all cancer patient pools compared with the benign sera.

Overall, these comparisons suggest that gene expression levels are not reliable indicators of blood levels of a given protein, and use of gene expression levels to predict blood biomarkers is likely to be of limited value. This is not surprising because: 1) gene expression levels do not always correlate with protein abundance within cells; 2) shedding of proteins into the extracellular space and, more specifically, into the vascular system, does not necessarily depend upon the tissue levels of that protein; and 3) changes in proteolytic processing, PTM levels, or other processing of proteins that might affect their blood concentration may differ between normal and cancer states.

### MRM Assays and Quantitation of Normal, Benign, Early Stage EOC and Late Stage EOC Serum Pools

**Figure 6:**
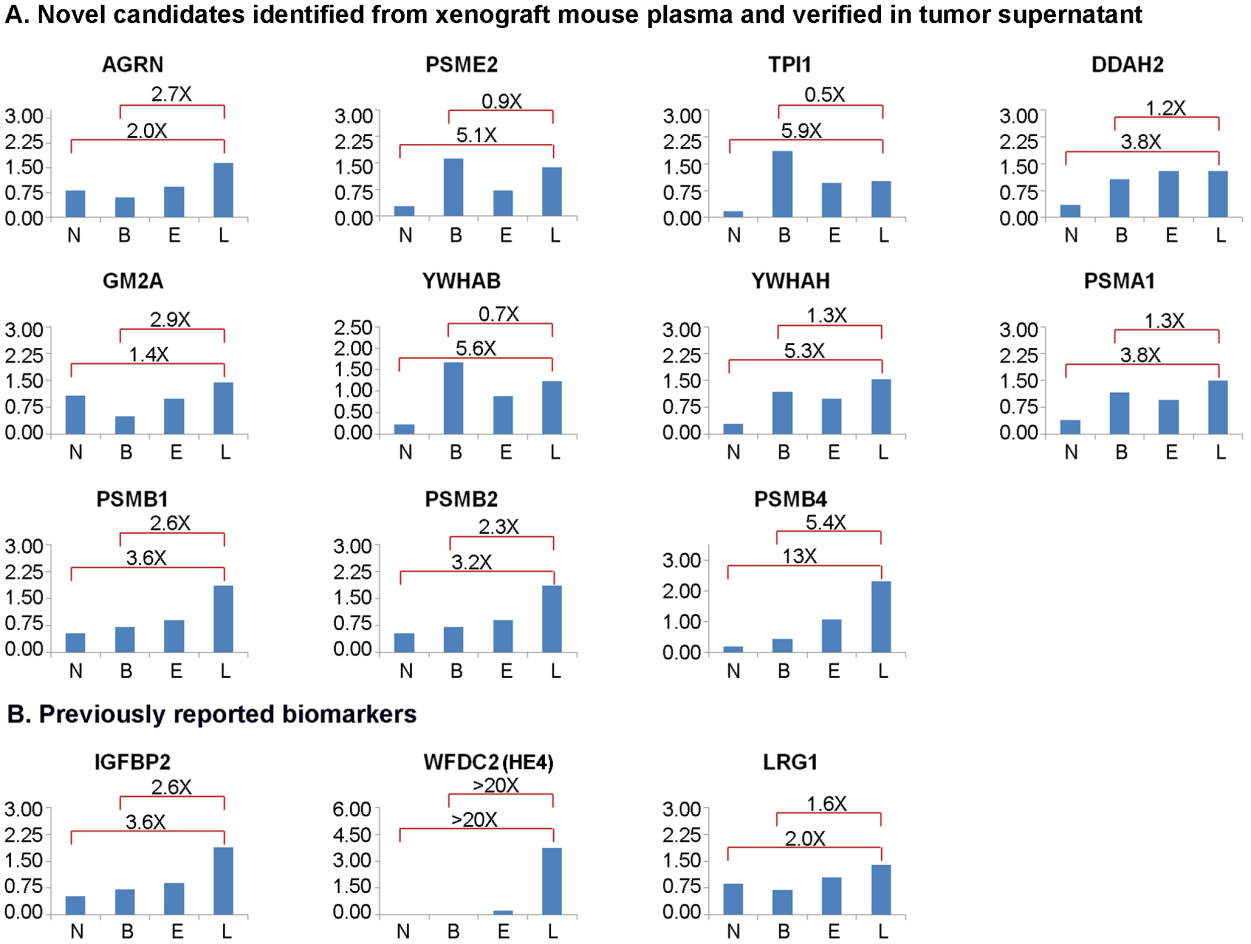
Verification of promising candidate biomarkers using a label-free MRM assay. Normalized relative protein amounts are shown for serum pools of normal (N; n = 9), benign (B; n = 10), early-stage ovarian cancer (E; stages 1 and 2; n = 18), and late-stage ovarian cancer (L; stage 3; n = 29). Ratios of late-stage cancer to benign disease and normal donors are shown above the histograms. (A) The 11 novel high priority biomarkers from the 15–50 kDa region of the gel. (B)Three previously reported biomarkers included as references and analyzed in parallel with the novel biomarkers.

We subsequently attempted to set up MRM assays for the 11 novel and three known biomarkers shown in **Table 1** as described in Methods. MRM assays achieve high selectivity by monitoring the combination of the specific mass/charge of a parent ion and a unique fragment ion produced after collision to quantify the targeted peptide in a complex mixture. MRM assays targeting at least two peptides per protein were successfully established for all targeted proteins. The methods were integrated into a single multiplexed MRM assay that was subsequently used to quantitate the levels of these proteins in four serum pools, including: a normal serum pool (pool N; n = 9), a benign ovarian tumor pool (pool B, n = 10), an early-stage ovarian cancer pool (pool E: stage 1 and 2, n = 18), and a late-stage cancer pool (pool L: stage 3, n = 29). The cancer pools included serum from patients with different EOC histotypes, although the majority of tumors were the serous subtype as is typically the case in groups of EOC patients. Details of the patients and samples used to prepare these pools are summarized in **Table S2**. The peptides and transitions used in the integrated multiplex MRM assay, as well as the resulting relative quantitative data for the four pools, are shown in **Table S3**. Resulting relative protein quantities for the four pools are summarized in **Figure 6A** for the 11 novel candidates. The levels in the same serum pools of the three previously reported biomarkers are shown for reference in **Figure 6B**.

These results confirm that quantitative MRM assays were established for all 11 targeted proteins and that all targeted proteins showed elevated levels in initial analysis of sera from advanced EOC. This 100% success using these two criteria is dramatically better than the 20% success rate achieved for setting up MRM assays for TOV-112D derived candidate biomarkers showing elevated levels in initial screens of sera from advanced EOC. This more efficient selection of candidate biomarkers was achieved because the current strategy utilized additional criteria prior to attempting to set up MRM assays. The key advantages of the current approach include analysis of the tumor supernatant to extend sequence coverage for putative human plasma proteins in the xenograft mouse plasma and comparison of remaining high priority candidate biomarkers to an in-depth discovery mode quantitative comparison of serum pools from benign and advanced EOC patients. This latter analysis identified those proteins detectable in patient serum as well as those proteins exhibiting elevated levels in EOC patient sera. Interestingly, approximately two-thirds of the high priority candidates from the xenograft mouse plasma both expected to be in the 15–50 kDa region and verified in the tumor supernatant, were detected in the patient pools and half of these met the criteria used above for elevated levels in EOC serum. This 20% success rate is very similar to that obtained with the TOV-112D candidate biomarkers. The major difference in the current study is that time and expenses were not invested in attempting to set up MRM assays for the 75–80% of biomarkers that would ultimately fail to be detected in patient plasma or that would not show elevated levels in advanced EOC patient serum. Although substantial mass spectrometer and analysis time was invested in conducting the in-depth discovery mode quantitative comparison of serum pools from benign and advanced EOC patients, these analyses do not need to be repeated as the same dataset can be used to screen future candidate biomarkers. Additionally, this study includes pools of mixed histotypes that approximate the mixtures of cancer subtypes typically seen clinically because the numbers of available samples and the assay throughput were too low to distinguish potential subtype specific biomarkers. One goal of future studies using higher throughput assays such as sandwich ELISA will be to carefully evaluate potential relationships between EOC subtypes and these biomarker candidates.

### Conclusions

In the current study, improved strategies for both discovery and triaging novel blood biomarkers for EOC have been developed. The utility of analyzing xenograft mouse plasma and the corresponding tumor supernatant in parallel was demonstrated. The presence of human proteins in the plasma demonstrated these proteins were produced by the tumor and shed into the blood, but many of these assignments were based upon only a few peptides. Analysis of the tumor supernatant produced far more extensive sequence coverage for human proteins and confirmed many of the proteins identified in the plasma as human. In most cases an increased sequence coverage provided additional peptide candidates for potential MRM assays. A second key step in the prioritization and verification strategy was to compare candidate plasma biomarkers to an in-depth, label-free comparison of benign disease and advanced cancer patient serum pools to prescreen candidate biomarkers prior to setting up MRM assays. By extending reverse-phase gradients for the discovery mode analysis of these samples to four hours, the detection sensitivity is similar to that of MRM assays using shorter gradients. That is, if a protein cannot be detected in this dataset, it will probably not be feasible to set up an MRM assay and, therefore, effort is not wasted in assay development. Furthermore, by comparing candidate biomarker levels in the benign and advanced cancer patient pools, only those proteins showing elevated levels in the cancer sera advance to MRM assay development. This new approach reduces the effort invested in setting up MRM assays by about four-fold relative to the biomarkers detected in advanced EOC patient sera at elevated levels. Based upon initial screening of large pools of normal, benign, early, and advanced ovarian cancer sera, all of the biomarkers selected for MRM assay development in the current study should move forward and be further evaluated using serum or plasma from individual patients and controls. Although the fold changes observed in the pooled samples for most of these candidate biomarkers are not as large as HE4, the ranges of values for these biomarkers in individual EOC and control sera need to be determined in order to compare their diagnostic capacities to HE4 and CA125. While it is unlikely that most individual biomarkers will prove to be superior to HE4 or CA125, it is more likely that combinations with each other or with CA125 and HE4 could outperform the use CA125 or HE4 alone. Finally, the strategies developed in this study demonstrate that in-depth analysis of xenograft mouse plasma with efficient pilot verification using multiplexed assays can efficiently identify multiple promising candidate EOC biomarkers. This approach can be readily applied to further in-depth analysis of the OVCAR-3 cell line, as well as other EOC cell lines to identify additional EOC plasma biomarkers.

## Supporting Information

Figure S1 Analysis of OVCAR-3 xenograft plasma and tumor supernatant. (A) SDS-PAGE of concentrated media from the OVCAR-3 tumor supernatant. The sample was separated for 6 cm, the gel lane was sliced into 60 uniform fractions, digested with trypsin and analyzed by LC-MS/MS. (B) Analytical SDS-PAGE of unfractionated, depleted mouse plasma (DP), and MicroSol IEF fractions (F1–F4) and membrane extractions (M1–M5). (C) Representative preparative SDS-PAGE of the samples shown in panel B. Distances samples were separated are indicated and gel lanes were cut into 1 mm slices for trypsin digestion and subsequent LC-MS/MS analysis.
(PDF)

Table S1 Proteins identified in the OVCAR-3 tumor supernatant by two or more unique peptides.
(PDF)

Table S2 Sample classification for ovarian cancer patient sera.
(PDF)

Table S3 Peptide transitions monitored by MRM.
(PDF)
