# Supplementary figures and images for: Identification of Multiple Novel Protein Biomarkers Shed by Human Serous Ovarian Tumors into the Blood of Immunocompromised Mice and Verified in Patient Sera
Source: PLoS One. 2013 Mar 27;8(3):e60129. doi: 10.1371/journal.pone.0060129 (PMC3609810; doi:10.1371/journal.pone.0060129)

Figure S1

**A.**

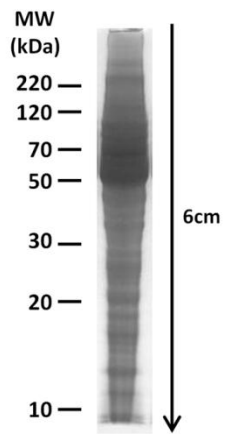

**B.**

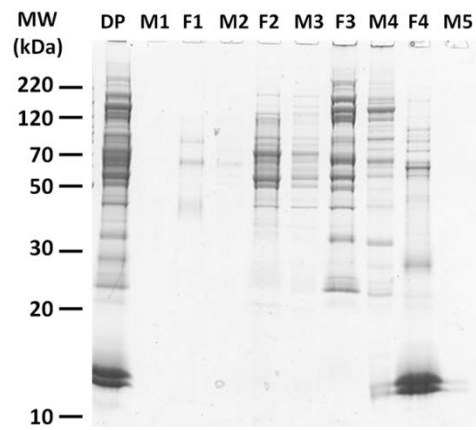

**C.**

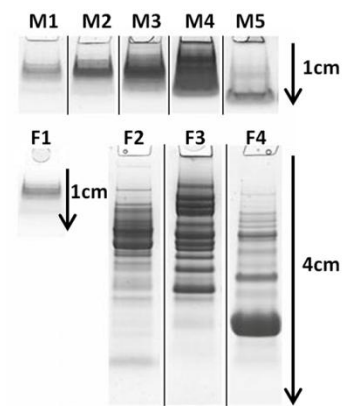

Supplement: Figure S1 — Analysis of OVCAR-3 xenograft plasma and tumor supernatant. (A) SDS-PAGE of concentrated media from the OVCAR-3 tumor supernatant. The sample was separated for 6 cm, the gel lane was sliced into 60 uniform fractions, digested with trypsin and analyzed by LC-MS/MS. (B) Analytical SDS-PAGE of unfractionated, depleted mouse plasma (DP), and MicroSol IEF fractions (F1–F4) and membrane extractions (M1–M5). (C) Representative preparative SDS-PAGE of the samples shown in panel B. Distances samples were separated are indicated and gel lanes were cut into 1 mm slices for trypsin digestion and subsequent LC-MS/MS analysis. (PDF) [file pone.0060129.s001.pdf]
