# Supplementary material for: Identification of Multiple Novel Protein Biomarkers Shed by Human Serous Ovarian Tumors into the Blood of Immunocompromised Mice and Verified in Patient Sera
Source: PLoS One. 2013 Mar 27;8(3):e60129. doi: 10.1371/journal.pone.0060129 (PMC3609810; doi:10.1371/journal.pone.0060129)
Supplement: Table S1 — Proteins identified in the OVCAR-3 tumor supernatant by two or more unique peptides. (PDF) [file pone.0060129.s002.pdf]

Table S1. Proteins identified in the OVCAR-3 tumor supernatant by two or more unique peptides.

Protein or Protein Group	Accession Number	Unique Peptide Count	Spectrum Count	Sequence Coverage (%)	Length	MolWt	pI	Descriptive Name
1	Q14204	249	1239	64.9	4646	532412	6.4	Cytoplasmic dynein 1 heavy chain 1
2	Q15149-4	246	1340	59.5	4547	516204	5.8	Isoform 4 of Plectin
3	E9QM71	240	1141	62.4	4644	532050	6.4	Uncharacterized protein
4	Q09666	238	3747	50.2	5890	629114	6.1	Neuroblast differentiation-associated protein AHNAK
5	Q9UPN3	206	572	51.3	5430	620426	5.4	Microtubule-actin cross-linking factor 1, isoforms 1/2/3/5
6	E9QMZ5	183	865	44.3	4543	517292	5.8	Uncharacterized protein
6	Q9QXS1-3	183	865	44.3	4543	517320	5.8	Isoform PLEC-1A of Plectin
7	P78527	167	897	48.6	4128	469093	7.1	DNA-dependent protein kinase catalytic subunit
8	Q13813	158	700	71.4	2472	284538	5.3	Spectrin alpha chain, brain
9	Q7Z6Z7-2	152	969	50.8	4358	480203	5.2	Isoform 2 of E3 ubiquitin-protein ligase HUWE1
9	Q7Z6Z7	152	969	50.7	4374	481896	5.2	E3 ubiquitin-protein ligase HUWE1
10	E9PZ16	150	852	49.1	4383	469789	6.5	Uncharacterized protein
11	A3KGU7	149	602	68.1	2477	285151	5.3	Spectrin alpha 2
11	B9EKJ1	149	602	68.1	2477	285178	5.3	Spna2 protein
12	B2ZZ83	144	815	78.8	2633	281633	5.7	Filamin B
13	Q8WXI7	140	743	8.8	22152	2354561	6	Mucin-16
14	Q01082	133	597	70.3	2364	274608	5.6	Spectrin beta chain, brain 1
15	B9EKP5	132	1436	67.8	2639	280470	6	Filamin, alpha
15	Q8BTM8	132	1436	67.5	2647	281191	6	Filamin-A
16	A2AFQ0	129	815	41.3	4378	482697	5.2	HECT, UBA and WWE domain containing 1
16	Q7TMY8	129	815	41.3	4377	482668	5.2	E3 ubiquitin-protein ligase HUWE1
16	Q4JG03	129	815	41.3	4378	482751	5.2	Mcl-1 ubiquitin ligase
16	A2AFQ1	129	815	41.3	4377	482640	5.2	HECT, UBA and WWE domain containing 1
17	P21333	129	1591	70.1	2647	280737	6.1	Filamin-A
18	P49327	128	1260	72	2511	273424	6.4	Fatty acid synthase
19	UPI0001BB0BFA	128	291	31.6	5256	596494	6.4	Protein ALO17 isoform 1
20	Q5T4S7-3	127	470	35.2	5159	571859	6	Isoform 3 of E3 ubiquitin-protein ligase UBR4
20	Q5T4S7	127	470	35	5183	573849	6	E3 ubiquitin-protein ligase UBR4
20	Q5T4S7-2	127	470	34.9	5204	575966	6	Isoform 2 of E3 ubiquitin-protein ligase UBR4
21	Q62261	126	532	66.6	2363	274221	5.6	Spectrin beta chain, brain 1
22	Q9Y490	126	1185	69.4	2541	269765	6.1	Talin-1
23	A2AIM8	122	994	69.5	2541	269819	6.2	Talin 1
24	P35579	112	750	59.1	1960	226530	5.6	Myosin-9
25	UPI00006070DC	111	411	64.7	2591	276597	5.7	Filamin-B
25	E9QNV9	111	411	64.4	2602	277822	5.7	Uncharacterized protein
26	UPI0000DBD688	109	653	48.1	3061	333333	5.8	collagen alpha-1(XII) chain
26	E9PX70	109	653	48	3064	333731	5.8	Uncharacterized protein
26	Q60847-2	109	653	48	3065	333672	5.8	Isoform 2 of Collagen alpha-1(XII) chain
26	Q60847-5	109	653	47.9	3068	334069	5.8	Isoform 5 of Collagen alpha-1(XII) chain
26	Q60847	109	653	47.1	3120	340215	5.6	Collagen alpha-1(XII) chain
27	E1NZA1	107	521	52.1	2671	292742	7.4	Peroxisome proliferator activated receptor interacting complex protein
27	UPI0000451CA7	107	521	52.1	2671	292708	7.4	Translational activator GCN1
28	B1ART6	105	278	24.8	5333	608246	5.4	Microtubule-actin crosslinking factor 1 (Fragment)
28	E9PVY6	105	278	24.3	5430	619491	5.4	Uncharacterized protein
29	Q68FD5	103	712	77.3	1675	191555	5.7	Clathrin heavy chain 1
29	Q5SXR6	103	712	77.1	1679	191985	5.7	Clathrin, heavy polypeptide (Hc)
30	Q00610-2	103	723	79	1639	187889	5.7	Isoform 2 of Clathrin heavy chain 1
30	Q00610	103	723	77.3	1675	191613	5.7	Clathrin heavy chain 1
31	Q8VDD5	99	631	56.6	1960	226370	5.7	Myosin-9
32	O60437	97	792	61	1756	204745	5.6	Periplakin
32	UPI000034ECE3	97	792	61	1756	204676	5.6	periplakin
33	A2AN08-3	96	335	23.7	5156	570308	6.1	Isoform 3 of E3 ubiquitin-protein ligase UBR4
33	A2AN08	96	335	23.6	5180	572298	6.1	E3 ubiquitin-protein ligase UBR4
33	A2AN08-5	96	335	23.4	5208	575184	6	Isoform 5 of E3 ubiquitin-protein ligase UBR4
34	UPI0001E6B5EB	94	345	45.4	3057	330028	7.6	PREDICTED: collagen alpha-3(VI) chain isoform 1
34	UPI0001E6B858	94	345	45.4	3062	330512	7.8	PREDICTED: collagen alpha-3(VI) chain isoform 4
35	E9Q8N1	94	130	3.8	33467	3715911	6.4	Uncharacterized protein
35	E9Q8K5	94	130	3.8	33467	3715952	6.4	Uncharacterized protein
35	A2ASS6	94	130	3.6	35213	3906375	6.2	Titin
36	P01024	87	496	67.7	1663	187147	6.4	Complement C3
37	O15230	87	232	33	3695	399740	7	Laminin subunit alpha-5
38	Q80XP1	86	586	65.7	1663	186483	6.7	Complement component 3
39	Q80YX1	85	429	61.2	2110	231805	4.9	Tenascin
40	Q3UGY5	83	567	52.5	2386	262841	5.8	Putative uncharacterized protein
41	P46940	82	513	63.1	1657	189251	6.5	Ras GTPase-activating-like protein IQGAP1
42	P50851	81	275	37.9	2863	319107	5.6	Lipopolysaccharide-responsive and beige-like anchor protein
43	D6RGG3	81	378	33.1	3062	333203	5.5	Uncharacterized protein
43	Q99715	81	378	33.1	3063	333147	5.5	Collagen alpha-1(XII) chain
44	Q9NYQ8	81	154	26.8	4349	479321	5.2	Protocadherin Fat 2
45	O15020	79	138	53.2	2390	271323	6.1	Spectrin beta chain, brain 2
45	UPI0000135DB9	79	138	53.2	2390	271293	6.1	Spectrin beta chain, brain 2
46	O75643	79	215	50.7	2136	244505	6.1	U5 small nuclear ribonucleoprotein 200 kDa helicase
47	Q14980	79	280	48.3	2115	238257	5.8	Nuclear mitotic apparatus protein 1
48	Q4LE33	78	338	52.4	2233	244402	4.9	TNC variant protein (Fragment)
49	Q86UP2	74	420	59.1	1357	156275	5.6	Kinectin
50	P42858	74	168	35.4	3142	347603	6.2	Huntingtin
51	Q8VHX6-2	73	141	42.8	2693	287363	6	Isoform 2 of Filamin-C
51	Q8VHX6	73	141	42.3	2726	291117	5.9	Filamin-C
52	O43707	73	1546	83.8	911	104854	5.4	Alpha-actinin-4
53	Q6P2Q9	71	107	40.9	2335	273599	8.8	Pre-mRNA-processing-splicing factor 8
54	Q9QXQ0	70	1363	79.9	911	104915	5.4	Alpha-actinin-4
55	O00468	70	490	49.5	2045	214844	6.4	Agrin
56	Q93008	70	161	36.2	2570	292278	5.8	Probable ubiquitin carboxyl-terminal hydrolase FAF-X
57	Q6ZQK2	69	351	52.9	1681	191311	6.6	MKIAA0051 protein (Fragment)
57	UPI0001F78C4C	69	351	52.9	1681	191283	6.5	IQ motif containing GTPase activating protein 1
58	Q8WXH0	69	136	12.7	6885	796457	5.4	Nesprin-2
58	Q8WXH0-2	69	136	12.6	6907	798877	5.4	Isoform 2 of Nesprin-2
59	Q91ZX7	68	126	19.1	4545	504745	5.4	Prolow-density lipoprotein receptor-related protein 1
60	Q7KZ85	67	144	50.6	1726	199071	4.9	Transcription elongation factor SPT6
61	P07814	66	181	56.7	1512	170590	7.3	Bifunctional aminoacyl-tRNA synthetase
62	B2RWW6	65	337	30.6	2671	293027	7.5	GCN1 general control of amino-acid synthesis 1-like 1 (Yeast)
62	Q3UHQ5	65	337	30.6	2671	292989	7.3	Putative uncharacterized protein
62	E9PVA8	65	337	30.6	2671	293018	7.4	Uncharacterized protein
63	Q92817	65	158	42.6	2033	231602	7	Envoplakin
64	Q9P273	65	106	35.5	2699	300950	6.4	Teneurin-3
65	P15924	65	315	27.2	2871	331774	6.8	Desmoplakin
66	A2AQ53	63	90	29.3	2873	312298	4.9	Fibrillin 1
67	E9Q414	63	70	19	4505	509437	6.8	Uncharacterized protein
68	P53621	63	215	68.2	1224	138345	7.7	Coatomer subunit alpha
69	Q64727	62	432	69.1	1066	116717	6	Vinculin
70	P18206-2	62	474	68.9	1066	116722	6.1	Isoform 1 of Vinculin
70	P18206	62	474	64.8	1134	123799	5.7	Vinculin
71	UPI000163999A	62	118	46.9	2162	236020	6.5	UPI000163999A UniRef100 entry

Table S1. Proteins identified in the OVCAR-3 tumor supernatant by two or more unique peptides.

Protein or Protein Group	Accession Number	Unique Peptide Count	Spectrum Count	Sequence Coverage (%)	Length	MolWt	pI	Descriptive Name
71	P27708	62	118	45.6	2225	242981	6.5	CAD protein
72	Q07954	62	117	17.5	4544	504610	5.4	Prolow-density lipoprotein receptor-related protein 1
73	E9QPW0	61	297	55.8	1495	165852	6.7	Uncharacterized protein
73	D3YW52	61	297	55.3	1507	167282	6.8	Uncharacterized protein
74	Q14152	61	277	44.6	1382	166569	6.8	Eukaryotic translation initiation factor 3 subunit A
74	Q24JU4	61	277	44.6	1382	166470	6.7	Eukaryotic translation initiation factor 3, subunit A
75	UPI000024DD4E	60	313	51	1607	177187	5.2	Laminin subunit gamma-1 precursor
76	E9Q616	60	1089	12.2	5656	604265	6.3	Uncharacterized protein
77	Q5VYK3	60	163	46.4	1845	204289	7.1	Proteasome-associated protein ECM29 log
77	UPI0000DD7F7A	60	163	42.4	2017	223692	8.7	Proteasome-associated protein ECM29 log
78	P35580	60	112	46	1976	228997	5.5	Myosin-10
78	B2RWP9	60	112	45.7	1985	230028	5.6	MYH10 protein
78	P35580-2	60	112	45.6	1992	230777	5.6	Isoform 2 of Myosin-10
78	P35580-3	60	112	45.5	1997	231367	5.5	Isoform 3 of Myosin-10
78	Q4LE45	60	112	45	2018	233847	5.6	MYH10 variant protein (Fragment)
79	Q9Y6D6	60	177	42.7	1849	208766	5.9	Brefeldin A-inhibited guanine nucleotide-exchange protein 1
80	Q8CIE6	59	205	62.2	1224	138446	7.7	Coatomer subunit alpha
81	P28665	59	259	52.5	1476	165297	6.4	Murinoglobulin-1
82	P07724	58	2414	85	608	68693	6.1	Serum albumin
83	A8K8U1	58	513	60.2	1230	136317	5.8	cDNA FLJ77762, highly similar to sapiens cullin-associated and neddylation-dissociated 1 (CAND1), mRNA
83	Q86VP6	58	513	60.2	1230	136375	5.8	Cullin-associated NEDD8-dissociated protein 1
84	P98160	58	241	16.7	4391	468835	6.5	Basement membrane-specific heparan sulfate proteoglycan core protein
85	B2RXW7	57	161	49.7	1738	192904	7.5	Complement component 4B (Childo blood group)
86	P55060	57	1000	73.6	971	110417	5.8	Exportin-2
87	Q16531	57	234	55.6	1140	126968	5.3	DNA damage-binding protein 1
88	P11047	56	256	47.8	1609	177602	5.1	Laminin subunit gamma-1
89	P55268	56	171	41	1798	195980	6.5	Laminin subunit beta-2
90	B2ZZ91	56	88	20	3269	377122	5	Golgin B1
91	Q9NR09	56	179	18	4857	530260	6	Baculoviral IAP repeat-containing protein 6
91	UPI000159689D	56	179	18	4857	530274	6	baculoviral IAP repeat-containing protein 6
92	E9PGE1	55	122	60.1	1253	138750	5.5	Uncharacterized protein
92	E7EX90	55	122	60	1256	139094	5.7	Uncharacterized protein
93	Q9NYU2	55	81	53.1	1555	177189	5.6	UDP-glucose:glycoprotein glucosyltransferase 1
94	Q460N5	55	111	42.1	1801	202799	7.2	Poly [ADP-ribose] polymerase 14
95	E9QQ50	54	210	41.7	1344	161936	6.8	Uncharacterized protein
95	P23116	54	210	41.7	1344	161936	6.8	Eukaryotic translation initiation factor 3 subunit A
96	E9QN70	54	211	34.5	1834	202440	5	Uncharacterized protein
96	Q3UHL7	54	211	34.5	1834	202444	5	Putative uncharacterized protein
97	UPI0000E67207	54	191	25.5	3083	338149	6.7	laminin subunit alpha-1 precursor
98	B2RUT2	54	77	33.6	1979	227596	5.3	Thyroid hormone receptor interactor 11
98	Q15643	54	77	33.6	1979	227584	5.3	Thyroid receptor-interacting protein 11
99	Q6ZQ38	53	473	54.2	1230	136331	5.8	Cullin-associated NEDD8-dissociated protein 1
100	P07900	53	1084	72	732	84660	5	Heat shock protein HSP 90-alpha
101	P55072	52	820	78.3	806	89322	5.3	Transitional endoplasmic reticulum ATPase Transitional endoplasmic reticulum ATPase
102	P22314	52	553	78.7	1058	117849	5.8	Ubiquitin-like modifier-activating enzyme 1
103	B0V043	52	151	53.7	1264	140466	7.6	Valyl-tRNA synthetase
103	P26640	52	151	53.7	1264	140476	7.6	Valyl-tRNA synthetase
104	E7EX73	52	498	43.8	1436	158644	5.2	Uncharacterized protein
104	D3DNT2	52	498	39.3	1600	175618	5.3	Eukaryotic translation initiation factor 4 gamma, 1, isoform CRA_c
105	P58252	51	557	67.9	858	95314	6.8	Elongation factor 2
105	Q3UDC8	51	557	67.9	858	95268	6.8	Putative uncharacterized protein
106	Q61147	51	227	57.7	1061	121151	5.8	Ceruloplasmin
106	E9PZD8	51	227	56.4	1086	124211	5.9	Uncharacterized protein
107	P13639	51	622	67.9	858	95338	6.8	Elongation factor 2
108	UPI0000167B25	51	236	31.3	2355	259223	5.7	fibronectin isoform 3 preproprotein
109	P12270	51	168	23.5	2363	267290	5	Nucleoprotein TPR
110	P25391	51	96	22.3	3075	337084	6.4	Laminin subunit alpha-1
111	O14980	50	495	65.4	1071	123386	6.1	Exportin-1
112	Q96T23	50	250	40.2	1441	163820	5	Remodeling and spacing factor 1
113	P02545	49	821	72.3	664	74140	7	Prelamin-A/C
114	P34932	49	333	68.7	840	94331	5.2	Heat shock 70 kDa protein 4
115	B4DZJ7	49	661	59.4	1065	118185	5.4	cDNA FLJ55629, highly similar to Transcription elongation factor SPT5
115	O00267	49	661	58.2	1087	121000	5.1	Transcription elongation factor SPT5
116	Q02952-3	49	188	42.5	1677	180989	4.4	Isoform 3 of A-kinase anchor protein 12
116	Q02952-2	49	188	42.3	1684	181688	4.4	Isoform 2 of A-kinase anchor protein 12
116	Q02952	49	188	40	1782	191480	4.4	A-kinase anchor protein 12
117	Q9P2E9	49	290	41	1410	152472	8.6	Ribosome-binding protein 1
117	A7BI36	49	290	37.5	1540	165715	9	p180/ribosome receptor
118	B7ZNH7	48	135	38.1	1794	192753	5.1	Collagen, type XIV, alpha 1
119	O00410	48	238	66.5	1097	123630	4.9	Importin-5
120	C9JSL9	48	76	41.4	1411	162359	5.6	Uncharacterized protein
120	Q15075	48	76	41.4	1411	162465	5.7	Early endosome antigen 1
121	Q7KZF4	47	178	68.4	910	101997	7.2	Staphylococcal nuclease domain-containing protein 1
121	Q59FF0	47	178	64.5	964	107433	7.5	EBNA-2 co-activator variant (Fragment)
122	Q5CAQ5	47	307	55.4	802	92340	4.9	Tumor rejection antigen (Gp96) 1
122	P14625	47	307	55.3	803	92469	4.8	Endoplasmin
123	P35573	47	64	42.2	1532	174763	6.8	Glycogen debranching enzyme
124	Q14839	47	135	33.9	1912	218003	5.9	Chromodomain-helicase-DNA-binding protein 4
124	Q14839-2	47	135	33.4	1940	220846	6	Isoform 2 of Chromodomain-helicase-DNA-binding protein 4
125	P48678	46	591	67.8	665	74238	7	Prelamin-A/C
126	Q08211	46	182	52.5	1270	140958	6.8	ATP-dependent RNA helicase A
127	Q9C0C9	46	105	46.8	1292	141293	5.1	Ubiquitin-conjugating enzyme E2 O
128	Q14683	46	88	43.2	1233	143233	7.6	Structural maintenance of chromosomes protein 1A
129	B0AZQ4	45	57	44	1217	141529	7.2	cDNA, FLJ79494, highly similar to Structural maintenance of chromosome 3
129	Q9UQE7	45	57	44	1217	141541	7.2	Structural maintenance of chromosomes protein 3
129	Q9CW03	45	57	44	1217	141555	7.2	Structural maintenance of chromosomes protein 3
129	Q86VX4	45	57	44	1217	141507	7.2	Structural maintenance of chromosomes 3
129	Q6P5E5	45	57	44	1216	141441	7.2	Structural maintenace of chromosomes 3
130	Q921I1	45	607	67	697	76724	7.2	Serotransferrin
131	P07901	45	928	61.1	733	84788	5	Heat shock protein HSP 90-alpha
132	P06909	45	153	54.5	1234	139082	6.9	Complement factor H
132	E9Q8I0	45	153	53.8	1252	141271	7.1	Uncharacterized protein
133	P14618	45	2032	80.4	531	57937	7.8	Pyruvate kinase isozymes M1/M2
134	Q13200	45	211	65.6	908	100200	5.2	26S proteasome non-ATPase regulatory subunit 2
135	B7ZLW8	45	61	51.1	1265	140599	5.5	5-methyltetrahydrofolate-cysteine methyltransferase
135	Q99707	45	61	51.1	1265	140527	5.6	Methionine synthase
136	P41252	45	61	46.9	1262	144498	6.1	Isoleucyl-tRNA synthetase, cytoplasmic
137	P19096	44	119	25.6	2504	272426	6.6	Fatty acid synthase

Table S1. Proteins identified in the OVCAR-3 tumor supernatant by two or more unique peptides.

Protein or Protein Group	Accession Number	Unique Peptide Count	Spectrum Count	Sequence Coverage (%)	Length	MolWt	pI	Descriptive Name
138	Q9Y678	44	305	69.9	874	97718	5.5	Coatomer subunit gamma
139	P53618	44	357	60.5	953	107142	6	Coatomer subunit beta
140	Q14997	44	65	33.4	1843	211332	6.9	Proteasome activator complex subunit 4
141	Q1HE25	43	525	80.5	914	105568	5.4	Actinin alpha 1 isoform b
142	P35221	43	496	66.3	906	100071	6.3	Catenin alpha-1
143	Q14697-2	43	204	63.9	966	109438	6.2	Isoform 2 of Neutral alpha-glucosidase AB
144	E9PLK3	43	267	62.2	915	102988	5.6	Uncharacterized protein
144	P55786	43	267	61.9	919	103276	5.7	Puromycin-sensitive aminopeptidase
145	P49588	43	138	61.8	968	106810	5.5	Alanyl-tRNA synthetase, cytoplasmic
146	E9PEZ3	43	147	46.5	1269	141061	5.4	Uncharacterized protein
146	UPI0001AE73A7	43	147	46.4	1272	141386	5.4	Protein diaphanous log 1 (Diaphanous-related formin-1) (DRF1).
146	Q6URC4	43	147	46.4	1272	141303	5.5	Diaphanous 1
146	O60610	43	147	46.4	1272	141347	5.4	Protein diaphanous log 1
147	E9PYU6	42	135	12.6	4854	529426	6.1	Uncharacterized protein
147	O88738	42	135	12.6	4845	528429	6.1	Ubiquitin-conjugating enzyme
148	P35606	42	192	69.4	906	102487	5.3	Coatomer subunit beta'
149	P22102	42	217	57.6	1010	107767	6.7	Trifunctional purine biosynthetic protein adenosine-3
149	Q3B7A7	42	217	57.6	1010	107723	6.8	Phosphoribosylglycinamide formyltransferase, phosphoribosylglycinamide synthetase, phosphoribosylaminoimidazole synthetase
149	Q59HH3	42	217	55.6	1046	112138	7.4	Phosphoribosylglycinamide formyltransferase, phosphoribosylglycinamide synthetase, phosphoribosylaminoimidazole synthetase isoform 1 variant (Fragment)
150	P00450	42	215	51.8	1065	122205	5.7	Ceruloplasmin
151	O60841	42	122	41.1	1220	138827	5.5	Eukaryotic translation initiation factor 5B
152	P26358	42	89	32.4	1616	183164	7.8	DNA (cytosine-5)-methyltransferase 1
153	Q15393	41	98	50.3	1217	135577	5.3	Splicing factor 3B subunit 3
153	Q921M3	41	98	50.3	1217	135550	5.3	Splicing factor 3B subunit 3
154	Q3UBU0	41	240	52.4	802	92476	4.8	Putative uncharacterized protein
155	E9QQ49	41	59	38.9	1551	176433	5.6	Uncharacterized protein
156	P26639	41	188	65.6	723	83435	6.7	Threonyl-tRNA synthetase, cytoplasmic
156	Q53GX7	41	188	65.6	723	83445	6.7	Threonyl-tRNA synthetase variant (Fragment)
157	Q92598	41	139	64.9	858	96865	5.4	Heat shock protein 105 kDa
158	Q9UIA9	41	169	51	1087	123907	6.3	Exportin-7
158	E7ESC6	41	169	50.9	1088	124066	6.5	Uncharacterized protein
159	B2R5V9	41	159	46.5	1268	141379	6.9	cDNA, FLJ92652, highly similar to sapiens high density lipoprotein binding protein (vigilin)(HDLBP), mRNA
160	Q7L576	41	93	39.2	1253	145182	6.9	Cytoplasmic FMR1-interacting protein 1
161	O75116	41	50	39.1	1388	160899	6	Rho-associated protein kinase 2
162	Q92673	41	44	24.7	2214	248425	5.6	Sortilin-related receptor
163	A1BN54	40	480	78.4	887	102720	5.5	Alpha actinin 1a
164	P52480	40	1548	76.8	531	57845	7.5	Pyruvate kinase isozymes M1/M2
165	Q3TXV1	40	187	62.7	908	100145	5.2	Putative uncharacterized protein
165	Q8VDM4	40	187	62.7	908	100203	5.2	26S proteasome non-ATPase regulatory subunit 2
166	E9QAI5	40	71	26.4	2162	236247	6.5	Uncharacterized protein
166	B2RQC6	40	71	25.7	2225	243236	6.4	Carbamoyl-phosphate synthetase 2, aspartate transcarbamylase, and dihydroorotase
167	Q10567-3	40	214	58.8	939	103564	5.2	Isoform C of AP-1 complex subunit beta-1
168	Q8TEX9	40	282	57.8	1081	118715	5	Importin-4
168	Q8TEX9-2	40	282	57.7	1083	118901	4.9	Isoform 2 of Importin-4
169	P10586-2	40	53	32.2	1898	211685	6.4	Isoform 2 of Receptor-type tyrosine-protein phosphatase F
169	P10586	40	53	32	1907	212877	6.3	Receptor-type tyrosine-protein phosphatase F
170	P45974-2	39	193	58.6	835	93308	5.1	Isoform Short of Ubiquitin carboxyl-terminal hydrolase 5
171	A0AVT1	39	96	53.2	1052	117970	6.1	Ubiquitin-like modifier-activating enzyme 6
172	P33176	39	140	48.4	963	109685	6.5	Kinesin-1 heavy chain
173	Q9P2J5	39	89	48	1176	134466	7.3	Leucyl-tRNA synthetase, cytoplasmic
174	Q9NTJ3	39	48	39.2	1288	147182	6.8	Structural maintenance of chromosomes protein 4
175	Q3TMY8	38	132	61.1	971	109361	5	Putative uncharacterized protein
176	B9EHR3	38	58	32.2	1560	173089	5.4	Tenascin N
176	Q80Z71	38	58	32.2	1560	173115	5.4	Tenascin-N
177	A2ALW0	38	93	30.1	1840	203702	7.1	Novel protein containing 10 HEAT domains
177	Q6PDI5-2	38	93	30.1	1840	203927	7.2	Isoform 2 of Proteasome-associated protein ECM29 homolog
177	Q6PDI5	38	93	30.1	1840	203716	7.1	Proteasome-associated protein ECM29 homolog
177	E9QKS7	38	93	30.1	1840	203913	7.2	Uncharacterized protein
178	B4DE59	38	909	76.7	563	62616	5.2	cDNA FLJ60424, highly similar to Junction plakoglobin
179	B2R6D0	38	105	56.9	953	105838	5.4	cDNA, FLJ92896, highly similar to sapiens proteasome (prosome, macropain) 26S subunit, non-ATPase, 1 (PSMD1), mRNA
179	Q99460	38	105	56.9	953	105836	5.4	26S proteasome non-ATPase regulatory subunit 1
180	P11216	38	126	56.9	843	96696	6.9	Glycogen phosphorylase, brain form
181	Q14566	38	220	52.7	821	92889	5.4	DNA replication licensing factor MCM6
182	B4E0E1	38	163	47.3	993	111127	8.9	cDNA FLJ53442, highly similar to Poly (ADP-ribose) polymerase 1 (EC 2.4.2.30)
182	P09874	38	163	46.4	1014	113084	8.9	Poly [ADP-ribose] polymerase 1
183	B4DIM0	38	96	43.8	1145	125137	8.2	cDNA FLJ56442, highly similar to ATP-citrate synthase (EC 2.3.3.8)
184	Q9H2G2	38	77	41	1235	142695	5.1	STE20-like serine/threonine-protein kinase
185	A7E2Y5	38	54	23	2243	254451	6.8	DnaJ (Hsp40) log, subfamily C, member 13
185	O75165	38	54	23	2243	254412	6.7	DnaJ log subfamily C member 13
186	Q3TXG4	37	194	55.4	943	103935	5.2	Adaptor protein complex AP-1, beta 1 subunit, isoform CRA_b
186	Q8CC13	37	194	54.8	953	104998	5.1	Putative uncharacterized protein
187	Q02053	37	364	50.4	1058	117809	5.7	Ubiquitin-like modifier-activating enzyme 1
188	B9EIU1	37	76	31	1512	169962	7.6	Glutamyl-prolyl-tRNA synthetase
188	Q8CGC7	37	76	31	1512	170052	7.7	Bifunctional aminoacyl-tRNA synthetase
188	E9QKC4	37	76	31	1512	170078	7.7	Uncharacterized protein
189	Q969I0	37	1440	85.5	365	41108	5	KRT8 protein (Fragment)
190	P13010	37	381	68.3	732	82705	5.8	X-ray repair cross-complementing protein 5
191	P47897	37	137	62.7	775	87799	7.2	Glutaminyl-tRNA synthetase
192	P07384	37	220	61.9	714	81890	5.7	Calpain-1 catalytic subunit
193	B2R7C5	37	151	58.5	808	91009	5.8	cDNA, FLJ93378, highly similar to sapiens MCM3 minichromosome maintenance deficient 3 (S. cerevisiae) (MCM3), mRNA
193	Q53HJ4	37	151	58.5	808	90981	5.7	Minichromosome maintenance protein 3 variant (Fragment)
193	P25205	37	151	58.5	808	90981	5.8	DNA replication licensing factor MCM3
194	B4DLA6	37	160	55.7	823	92722	7	cDNA FLJ54365, highly similar to DNA replication licensing factor MCM4
194	B3KMX0	37	160	53.1	863	96604	6.7	cDNA FLJ12837 fis, clone NT2RP2003228, highly similar to DNA replication licensing factor MCM4
195	B7Z815	37	70	49.3	1086	126269	5.7	Ubiquitin carboxyl-terminal hydrolase
195	Q93009	37	70	48.5	1102	128302	5.6	Ubiquitin carboxyl-terminal hydrolase 7
196	B2R721	37	79	38.8	1019	117811	6.6	cDNA, FLJ93240, highly similar to sapiens insulin-degrading enzyme (IDE), mRNA

Table S1. Proteins identified in the OVCAR-3 tumor supernatant by two or more unique peptides.

Protein or Protein Group	Accession Number	Unique Peptide Count	Spectrum Count	Sequence Coverage (%)	Length	MolWt	pI	Descriptive Name
196	P14735	37	79	38.8	1019	117968	6.6	Insulin-degrading enzyme
197	P24928	37	72	27.5	1970	217174	7.4	DNA-directed RNA polymerase II subunit RPB1
198	P20918	36	198	63.4	812	90782	6.6	Plasminogen
199	Q9QZE5	36	259	60.5	874	97513	5.3	Coatomer subunit gamma
199	E9Q5P4	36	259	60.3	877	97958	5.5	Uncharacterized protein
200	P97927	36	96	26.7	1816	201818	6.2	Laminin subunit alpha-4
201	E5RKB6	36	42	19.8	2571	276470	6.6	FELE-1
201	UPI000019347C	36	42	19.8	2571	276471	6.6	stabilin-1 precursor
202	E9Q555	36	60	9.3	5150	584506	6.8	Uncharacterized protein
203	P49915	36	190	73	693	76715	6.9	GMP synthase [glutamine-hydrolyzing]
204	P50990	36	70	70.1	548	59621	5.6	T-complex protein 1 subunit theta
205	P49321	36	162	65.5	788	85238	4.3	Nuclear autoantigenic sperm protein
206	B2RBR9	36	455	62	876	97184	4.8	cDNA, FLJ95650, highly similar to sapiens karyopherin (importin) beta 1 (KPNB1), mRNA
206	Q14974	36	455	62	876	97170	4.8	Importin subunit beta-1
207	E7EQR4	36	525	57.3	586	69372	6.2	Uncharacterized protein
207	Q6NUR7	36	525	57.3	586	69242	6.3	Ezrin
207	P15311	36	525	57.3	586	69413	6.3	Ezrin
208	Q8WUM4	36	178	50.2	868	96023	6.5	Programmed cell death 6-interacting protein
209	O95373	36	413	47	1038	119516	4.8	Importin-7
210	Q13308	36	84	46.4	1070	118392	7.1	Inactive tyrosine-protein kinase 7
210	B7Z477	36	84	46.1	1078	119225	7	cDNA FLJ55335, highly similar to Tyrosine-protein kinase-like 7
210	E9PFZ5	36	84	46.1	1078	119197	7	Uncharacterized protein
211	Q15020	36	58	44.3	963	109935	5.6	Squamous cell carcinoma antigen recognized by T-cells 3
212	O43847	36	76	40.3	1150	131571	5	Nardilysin
212	B1AKJ5	36	76	38.1	1219	139413	5	Nardilysin (N-arginine dibasic convertase)
212	O43847-2	36	76	38.1	1218	139284	5	Isoform 2 of Nardilysin
213	P35556	36	51	16.8	2912	314775	4.9	Fibrillin-2
214	O75533	35	183	43.1	1304	145830	7.1	Splicing factor 3B subunit 1
214	Q99NB9	35	183	43.1	1304	145816	7.1	Splicing factor 3B subunit 1
215	P10493	35	101	38.9	1245	136623	5.5	Nidogen-1
215	Q3TKX9	35	101	38.9	1245	136538	5.4	Nidogen 1
216	Q3V1M8	35	125	36.8	1268	141742	6.9	Putative uncharacterized protein
216	Q8VDJ3	35	125	36.8	1268	141742	6.9	Vigilin
217	P00558	35	539	76.7	417	44615	8.1	Phosphoglycerate kinase 1
218	O60763	35	218	52.6	962	107895	4.9	General vesicular transport factor p115
219	P27816-6	35	193	44.5	1135	119958	6.2	Isoform 6 of Microtubule-associated protein 4
219	P27816	35	193	43.8	1152	121005	5.4	Microtubule-associated protein 4
220	A8K8N7	35	46	43.3	1338	144724	5.8	Phosphoribosylformylglycinamidine synthase (FGAR amidotransferase), isoform CRA_b
221	O60264	35	81	34.8	1052	121905	8.1	SWI/SNF-related matrix-associated actin-dependent regulator of chromatin subfamily A member 5
222	Q13576	35	41	30.5	1575	180577	5.6	Ras GTPase-activating-like protein IQGAP2
222	UPI0000E56ECC	35	41	30.5	1575	180578	5.6	ras GTPase-activating-like protein IQGAP2
223	Q8IWV7	35	48	25.4	1749	200209	6	E3 ubiquitin-protein ligase UBR1
224	P42345	35	62	21.8	2549	288890	7.2	Serine/threonine-protein kinase mTOR
225	Q3TFE8	34	438	59.5	875	97113	4.8	Putative uncharacterized protein
225	Q6GTI5	34	438	59.5	876	97184	4.8	Karyopherin (Importin) beta 1
226	O55029	34	174	55.9	905	102449	5.3	Coatomer subunit beta'
227	A2AVJ7	34	170	27.6	1464	158395	9.2	Ribosome binding protein 1
227	Q99PL5	34	170	25.2	1605	172878	9.3	Ribosome-binding protein 1
228	P08238	34	478	72	724	83264	5	Heat shock protein HSP 90-beta
229	Q92973-2	34	142	56.6	890	101310	5	Isoform 2 of Transportin-1
230	P55884	34	147	56.1	814	92482	5	Eukaryotic translation initiation factor 3 subunit B
230	E7EME9	34	147	52.4	872	98916	5.1	Uncharacterized protein
230	P55884-2	34	147	52.3	873	99029	5.1	Isoform 2 of Eukaryotic translation initiation factor 3 subunit B
231	P11586	34	81	52.3	935	101559	7.3	C-1-tetrahydrofolate synthase, cytoplasmic
232	P49736	34	154	46.3	904	101896	5.5	DNA replication licensing factor MCM2
233	Q01970	34	59	40.7	1234	138799	5.9	1-phosphatidylinositol-4,5-bisphosphate phosphodiesterase beta-3
234	O94979	34	250	34.3	1220	133015	6.9	Protein transport protein Sec31A
234	D6REX3	34	250	33.4	1251	136226	7	Uncharacterized protein
235	P35222	33	327	60.7	781	85497	5.9	Catenin beta-1
235	Q3UZT7	33	327	60.7	781	85497	5.9	Putative uncharacterized protein
235	Q02248	33	327	60.7	781	85471	5.9	Catenin beta-1
236	P63017	33	600	73.4	646	70871	5.5	Heat shock cognate 71 kDa protein
236	Q3UBA6	33	600	73.4	646	70899	5.6	Putative uncharacterized protein
236	Q3TQ13	33	600	73.4	646	70871	5.5	Putative uncharacterized protein
236	Q3TH56	33	600	73.4	646	70872	5.4	Putative uncharacterized protein
237	P20152	33	343	70.4	466	53688	5.1	Vimentin
238	P26041	33	270	59.3	577	67767	6.6	Moesin
239	Q61316	33	184	49.6	841	94133	5.2	Heat shock 70 kDa protein 4
239	Q571M2	33	184	44.8	930	103260	5.9	MKIAA4025 protein (Fragment)
240	Q7TPT7	33	77	37.2	1263	140229	7.8	Valyl-tRNA synthetase
240	Q9Z1Q9	33	77	37.2	1263	140215	7.8	Valyl-tRNA synthetase
241	E9QNV4	33	63	14.6	3105	342781	6.1	Uncharacterized protein
241	UPI0000E6A55D	33	63	14.5	3118	343815	6.1	laminin subunit alpha-2
242	Q92499	33	197	63.4	740	82432	7.2	ATP-dependent RNA helicase DDX1
243	B2R8R5	33	307	58.9	835	88560	5.8	cDNA, FLJ94025, highly similar to sapiens tripartite motif-containing 28 (TRIM28), mRNA
243	Q13263	33	307	58.9	835	88550	5.8	Transcription intermediary factor 1-beta
244	B2RAH7	33	86	55.5	710	80734	5.9	cDNA, FLJ94921, highly similar to sapiens prolyl endopeptidase (PREP), mRNA
244	Q9UM02	33	86	55.5	710	80734	5.9	Prolyl endopeptidase
244	P48147	33	86	55.5	710	80700	5.9	Prolyl endopeptidase
245	P11021	33	204	53.2	654	72333	5.2	78 kDa glucose-regulated protein
246	B2RBE5	33	107	43.1	866	101300	7.4	cDNA, FLJ95468, highly similar to sapiens transcriptional coactivator tubedown-100 (TBDN100),transcript variant 1, mRNA
246	Q9BXJ9	33	107	43.1	866	101272	7.4	N-alpha-acetyltransferase 15, NatA auxiliary subunit
247	Q9P2R3	33	52	40.7	1169	128399	6.1	Ankyrin repeat and FYVE domain-containing protein 1
248	Q9Y2A7	33	69	40.5	1128	128790	6.6	Nck-associated protein 1
248	Q9Y2A7-2	33	69	40.3	1134	129517	6.7	Isoform 2 of Nck-associated protein 1
249	P29144	33	38	38.1	1249	138349	6.3	Tripeptidyl-peptidase 2
250	Q92626	33	64	34.8	1479	165274	7.2	Peroxidasin log
251	Q7Z406	33	49	25.2	1995	227868	5.6	Myosin-14
251	E9PHG4	33	49	25.1	2003	228663	5.6	Uncharacterized protein
251	UPI0000551C8C	33	49	24.8	2028	231214	5.6	Myosin-14 (Myosin heavy chain 14) (Myosin heavy chain, nonmuscle IIc) (Nonmuscle myosin heavy chain IIc) (NMHC II-C).
251	Q7Z406-2	33	49	24.7	2036	232009	5.6	Isoform 2 of Myosin-14
252	Q13085-2	33	38	19.2	2288	259684	6.6	Isoform 2 of Acetyl-CoA carboxylase 1
252	Q13085	33	38	18.7	2346	265551	6.4	Acetyl-CoA carboxylase 1
253	O54882	32	137	48.9	942	104669	6.5	PK-120

Protein or Protein Group	Accession Number	Unique Peptide Count	Spectrum Count	Sequence Coverage (%)	Length	MolWt	pI	Descriptive Name
253	Q8C7G9	32	137	48.9	942	104660	6.4	Inter alpha-trypsin inhibitor, heavy chain 4
253	Q505P8	32	137	48.9	942	104703	6.5	Inter alpha-trypsin inhibitor, heavy chain 4
254	Q3U7X9	32	94	48.3	938	104051	6.8	Putative uncharacterized protein
254	Q6PEE6	32	94	48.3	938	104002	6.8	Adaptor protein complex AP-2, alpha 2 subunit
254	Q69ZW4	32	94	46.8	967	107342	8.3	MKIAA0899 protein (Fragment)
255	Q3V117	32	76	37.1	1101	120795	7.4	ATP citrate lyase
256	Q2HZ94	32	39	28.5	1456	164981	6.8	Macrophage mannose receptor 1
256	Q61830	32	39	28.5	1456	165066	6.9	Macrophage mannose receptor 1
257	E9PVC5	32	304	24.2	1594	175458	5.4	Uncharacterized protein
258	Q53EM5	32	194	74.6	623	67906	7.8	Transketolase variant (Fragment)
258	P29401	32	194	74.6	623	67878	7.7	Transketolase
258	B4DE31	32	194	73.7	631	68742	7.7	cDNA FLJ54957, highly similar to Transketolase (EC 2.2.1.1)
258	E7EPA7	32	194	73.7	631	68814	7.5	Uncharacterized protein
259	B7Z373	32	289	66.8	742	81941	5.8	Gelsolin (Amyloidosis, Finnish type), isoform CRA_b
259	B7Z9A0	32	289	65.7	755	83103	5.8	cDNA FLJ56212, highly similar to Gelsolin
259	P06396	32	289	63.4	782	85697	6.3	Gelsolin
260	Q96TA1	32	168	63.6	733	82683	6.2	Niban-like protein 1
260	Q5VVW7	32	168	62.5	746	84138	6.2	Chromosome 9 open reading frame 88, isoform CRA_c
261	P42224	32	99	60.5	750	87335	6.1	Signal transducer and activator of transcription 1-alpha/beta
262	P12956	32	237	59.9	609	69843	6.6	X-ray repair cross-complementing protein 6
263	Q96QK1	32	147	54.6	796	91707	5.5	Vacuolar protein sorting-associated protein 35
264	P21399	32	77	49.6	889	98399	6.7	Cytoplasmic aconitate hydratase
265	Q96P70	32	188	43.4	1041	115963	4.8	Importin-9
266	A8K8S9	32	70	41.6	1173	133335	6.1	cDNA FLJ78424, highly similar to sapiens ubiquitination factor E4B (UFD2 log, yeast) (UBE4B), mRNA
266	O95155-2	32	70	41.6	1173	133275	6.1	Isoform 2 of Ubiquitin conjugation factor E4 B
266	O95155	32	70	37.5	1302	146184	6.6	Ubiquitin conjugation factor E4 B
267	Q9H2M9	32	57	36.6	1393	155984	5.6	Rab3 GTPase-activating protein non-catalytic subunit
268	Q9C0C2	32	124	27.4	1729	181795	4.9	182 kDa tankyrase-1-binding protein
269	E1P647	31	138	47	935	104336	5.5	Adaptor-related protein complex 2, beta 1 subunit, isoform CRA_b
269	Q9DBG3	31	138	46.9	937	104583	5.4	AP-2 complex subunit beta
269	P63010	31	138	46.9	937	104553	5.4	AP-2 complex subunit beta
269	E1P646	31	138	46.3	949	105476	5.4	Adaptor-related protein complex 2, beta 1 subunit, isoform CRA_d
269	A8K916	31	138	46.2	951	105702	5.3	cDNA FLJ78481, highly similar to sapiens adaptor-related protein complex 2, beta 1 subunit, mRNA
269	Q5SWR1	31	138	46.2	951	105722	5.3	Adaptor-related protein complex 2, beta 1 subunit
269	Q3ZB97	31	138	46.2	951	105692	5.3	Adaptor-related protein complex 2, beta 1 subunit, isoform CRA_b
270	Q99K51	31	108	61.3	630	70742	5.6	Plastin-3
270	B1AX58	31	108	60.4	639	71746	5.6	Plastin 3 (T-isoform)
271	E9QKZ2	31	137	42.6	1040	115951	4.8	Uncharacterized protein
271	Q91YE6	31	137	42.6	1041	116052	4.8	Importin-9
272	Q14AX9	31	74	29.3	1479	167045	6	Mannose receptor, C type 2
272	Q64449	31	74	29.3	1479	167093	6	C-type mannose receptor 2
273	B8JJN0	31	127	28.4	1276	142325	7.4	Complement component 2 (Within H-2S)
274	P11142	31	535	73.4	646	70898	5.5	Heat shock cognate 71 kDa protein
275	P13797	31	109	61.3	630	70811	5.6	Plastin-3
276	Q08J23	31	68	56.6	767	86471	6.8	tRNA (cytosine-5-)-methyltransferase NSUN2
277	Q9HCB6	31	273	50.2	807	90974	6.1	Spondin-1
277	UPI00001AEF58	31	273	50.2	806	90903	6.1	Spondin-1 precursor (F-spondin) (Vascular smooth muscle cell growth- promoting factor).
278	E7ESQ4	31	100	47.3	932	103307	7.4	Uncharacterized protein
278	O94973	31	100	47	939	103960	7	AP-2 complex subunit alpha-2
278	O94973-2	31	100	46.9	940	104089	6.9	Isoform 2 of AP-2 complex subunit alpha-2
279	A8K2U2	31	69	42.9	917	102359	6.1	cDNA FLJ75392, highly similar to sapiens hexokinase II (HKII) mRNA
279	P52789	31	69	42.9	917	102380	6.1	Hexokinase-2
280	A8K5Y7	31	78	41.3	1204	136310	5.9	cDNA FLJ78655, highly similar to sapiens exportin 5 (XPO5), mRNA
280	Q9HAV4	31	78	41.3	1204	136311	5.8	Exportin-5
280	C9J4N8	31	78	41.3	1204	136295	5.8	Uncharacterized protein
281	P47989	31	48	40.7	1333	146424	7.7	Xanthine dehydrogenase/oxidase
282	P42285	31	75	40.3	1042	117805	6.5	Superkiller viralicidic activity 2-like 2
283	Q9NZB2	31	131	36.6	1118	121888	8.9	Constitutive coactivator of PPAR-gamma-like protein 1
283	Q9NZB2-6	31	131	35.7	1146	125272	9	Isoform F of Constitutive coactivator of PPAR-gamma-like protein 1
284	P30622-2	31	42	28.8	1392	156781	5.4	Isoform 3 of CAP-Gly domain-containing linker protein 1
285	Q6PGP7	31	38	26.1	1564	175485	7.5	Tetratricopeptide repeat protein 37
286	Q9UPU5	31	57	17	2620	294364	6.1	Ubiquitin carboxyl-terminal hydrolase 24
287	Q61598	30	85	75.1	445	50537	6.2	Rab GDP dissociation inhibitor beta
287	Q3UPA3	30	85	65.2	512	57737	8	Putative uncharacterized protein (Fragment)
288	P20029	30	130	53.1	655	72422	5.2	78 kDa glucose-regulated protein
288	Q3U9G2	30	130	53.1	655	72406	5.2	Putative uncharacterized protein
288	Q3TKF8	30	130	53.1	655	72350	5.2	Putative uncharacterized protein
288	Q3TI47	30	130	53.1	655	72346	5.2	Putative uncharacterized protein
289	O88307	30	30	17.2	2215	247120	5.6	Sortilin-related receptor
289	Q3UHM3	30	30	17.2	2215	247085	5.5	Putative uncharacterized protein
290	Q63870	30	33	15.3	2944	295229	6.3	Collagen alpha-1(VII) chain
291	E9PYH0	30	82	10.7	3354	366895	4.6	Uncharacterized protein
291	E9QQ15	30	82	10.7	3353	366530	4.6	Uncharacterized protein
292	P06733	30	1387	80	434	47169	7.4	Alpha-enolase
293	P50395	30	98	73	445	50663	6.5	Rab GDP dissociation inhibitor beta
293	B4DLV7	30	98	72.4	449	51154	8.2	cDNA FLJ60299, highly similar to Rab GDP dissociation inhibitor beta
293	E7EU23	30	98	72.4	449	51181	8.2	Uncharacterized protein
294	A8K202	30	98	71.1	591	64510	6.7	5-aminoimidazole-4-carboxamide ribonucleotide formyltransferase/IMP cyclohydrolase, isoform CRA_g
295	P08107	30	449	70	641	70052	5.6	Heat shock 70 kDa protein 1A/1B
296	B3KU10	30	149	67.2	662	75404	6	cDNA FLJ39036 fis, clone NT2RP7008360, highly similar to Interferon-induced GTP-binding protein Mx1
297	Q9H4A4	30	118	63.1	650	72596	5.8	Aminopeptidase B
298	A8K690	30	158	55.4	543	62655	6.8	cDNA FLJ76863, highly similar to sapiens stress-induced-phosphoprotein 1 (Hsp70/Hsp90-organizing protein) (STIP1), mRNA
298	P31948	30	158	55.4	543	62639	6.8	Stress-induced-phosphoprotein 1
299	P54136	30	88	54.5	660	75379	6.7	Arginyl-tRNA synthetase, cytoplasmic
300	Q6PCE3	30	101	54.2	622	70442	7.2	Glucose 1,6-bisphosphate synthase
301	P13667	30	132	51.9	645	72933	5.1	Protein disulfide-isomerase A4
302	Q5HYE4	30	78	47.2	831	94638	6.8	Cysteinyl-tRNA synthetase, isoform CRA_e
303	Q14CX7	30	48	43.3	972	112292	6.6	N-alpha-acetyltransferase 25, NatB auxiliary subunit
304	Q9Y5B9	30	76	36.8	1047	119914	5.7	FACT complex subunit SPT16

Table S1. Proteins identified in the OVCAR-3 tumor supernatant by two or more unique peptides.

Protein or Protein Group	Accession Number	Unique Peptide Count	Spectrum Count	Sequence Coverage (%)	Length	MolWt	pI	Descriptive Name
305	A8K984	30	34	33.5	1197	135596	8.4	cDNA FLJ78259
305	O95347	30	34	33.5	1197	135656	8.4	Structural maintenance of chromosomes protein 2
306	P46939	30	60	12.1	3433	394467	5.3	Utrophin
307	Q6GU23	29	78	61.1	722	83126	7.1	Signal transducer and activator of transcription 3
307	P40763	29	78	57.3	770	88068	6.3	Signal transducer and activator of transcription 3
307	Q3ULI4	29	78	57.3	770	88082	6.4	Putative uncharacterized protein
307	Q3U5Q4	29	78	57.3	770	88068	6.4	Putative uncharacterized protein
307	P42227	29	78	57.3	770	88054	6.3	Signal transducer and activator of transcription 3
308	P09411	29	400	69.8	417	44550	7.9	Phosphoglycerate kinase 1
309	Q9D0R2	29	120	45.4	722	83356	7.4	Threonyl-tRNA synthetase, cytoplasmic
310	Q3TGL4	29	136	30.9	1174	126498	4.6	Fibulin 2, isoform CRA_b
310	UPI0000F0A5D2	29	136	29.7	1221	131834	4.7	fibulin-2 isoform a
311	UPI0000F59908	29	35	27.4	1388	160615	6	rho-associated protein kinase 2
311	P70336	29	35	27.4	1388	160585	6	Rho-associated protein kinase 2
311	A5XDA7	29	35	26.4	1444	166759	5.9	ROCK2 splice variant (Fragment)
311	E9PYM9	29	35	26.4	1445	166877	5.9	Uncharacterized protein
312	Q13228	29	71	82.2	472	52391	6.4	Selenium-binding protein 1
312	E9PE02	29	71	75.5	514	56866	6.5	Uncharacterized protein
313	O75083	29	78	71.9	606	66194	6.7	WD repeat-containing protein 1
314	P08670	29	236	63.7	466	53652	5.1	Vimentin
315	Q15436	29	94	63.7	765	86161	7.1	Protein transport protein Sec23A
316	O00429-2	29	141	60	725	80536	6.7	Isoform 4 of Dynamin-1-like protein
316	Q59GN9	29	141	57.9	751	83579	7.7	Dynamin-like protein DYNIV-11 variant (Fragment)
317	P11940	29	309	53.1	636	70671	9.5	Polyadenylate-binding protein 1
317	Q5R8F7	29	309	53.1	636	70643	9.5	Polyadenylate-binding protein 1
318	P33992	29	68	48.1	734	82286	8.4	DNA replication licensing factor MCM5
319	Q12906	29	119	44.4	894	95339	8.8	Interleukin enhancer-binding factor 3
319	UPI000059D66A	29	119	44.2	898	95808	8.8	interleukin enhancer-binding factor 3 isoform d
320	E9PHY5	29	63	41.5	935	104360	5.4	Uncharacterized protein
321	P15144	29	40	39.1	967	109539	5.5	Aminopeptidase N
321	Q59E93	29	40	38.7	977	110593	5.5	Membrane alanine aminopeptidase variant (Fragment)
322	Q9HC35	29	93	38	981	108916	6.4	Echinoderm microtubule-associated protein-like 4
323	Q9BTW9	29	45	37.7	1192	132600	6.2	Tubulin-specific chaperone D
323	Q9BTW9-4	29	45	36	1248	138669	6.4	Isoform 4 of Tubulin-specific chaperone D
324	O60271-4	29	34	33	1307	144681	5.2	Isoform 4 of C-Jun-amino-terminal kinase-interacting protein 4
324	D6RC32	29	34	32.9	1312	145263	5.2	Uncharacterized protein
324	O60271-2	29	34	32.9	1311	145135	5.2	Isoform 2 of C-Jun-amino-terminal kinase-interacting protein 4
325	Q92900-2	29	53	32.2	1118	123036	6.7	Isoform 2 of Regulator of nonsense transcripts 1
325	Q92900	29	53	31.9	1129	124345	6.6	Regulator of nonsense transcripts 1
326	A6NEM2	29	68	20	2080	213474	7.3	Uncharacterized protein
327	Q8VHM5	28	108	52.4	632	70888	8.1	Heterogeneous nuclear ribonucleoprotein R
327	Q3UMT8	28	108	52.4	632	70916	8.1	Putative uncharacterized protein
327	Q3U8W9	28	108	52.4	632	70878	8.1	Putative uncharacterized protein
327	O43390	28	108	52.3	633	70943	8.1	Heterogeneous nuclear ribonucleoprotein R
328	P13020	28	192	59.9	780	85942	6.2	Gelsolin
329	Q8JZQ9	28	112	41.5	803	91370	5	Eukaryotic translation initiation factor 3 subunit B
329	E9PX78	28	112	34.4	969	108836	5.5	Uncharacterized protein
329	Q8CIJ3	28	112	34.3	970	108983	5.5	Eif3b protein
330	Q8BKG3	28	65	33.8	1062	117532	6.9	Inactive tyrosine-protein kinase 7
331	P00338	28	803	83.7	332	36689	8.3	L-lactate dehydrogenase A chain
332	P04075	28	382	83.2	364	39420	8.1	Fructose-bisphosphate aldolase A
333	O60701	28	80	69.4	494	55024	7.1	UDP-glucose 6-dehydrogenase
334	P36871	28	74	66.7	562	61449	6.8	Phosphoglucomutase-1
335	B5BUB5	28	163	58.8	408	46867	7.1	Autoantigen La (Fragment)
335	P05455	28	163	58.8	408	46837	7.1	Lupus La protein
336	Q00839	28	299	45.2	825	90585	6	Heterogeneous nuclear ribonucleoprotein U
337	O94874	28	105	42.7	794	89595	6.8	E3 UFM1-protein ligase 1
338	Q5GLZ8-2	28	40	42.7	1049	117697	6.3	Isoform 2 of Probable E3 ubiquitin-protein ligase HERC4
338	Q5GLZ8	28	40	42.4	1057	118563	6.2	Probable E3 ubiquitin-protein ligase HERC4
339	O95786	28	45	41.2	925	106600	6.4	Probable ATP-dependent RNA helicase DDX58
340	P22059	28	67	38.9	807	89421	7.3	Oxysterol-binding protein 1
341	B4DVQ5	28	129	38.2	898	103645	5.5	cDNA FLJ55450, highly similar to Eukaryotic translation initiation factor 3 subunit 8
341	A1KYQ7	28	129	37.6	913	105404	5.7	Cell migration-inducing protein 17
341	Q99613	28	129	37.6	913	105344	5.7	Eukaryotic translation initiation factor 3 subunit C
342	Q14126	28	98	35	1118	122294	5.2	Desmoglein-2
343	C9J6D7	28	45	28.2	1310	146826	6.2	Uncharacterized protein
343	O75153	28	45	28.2	1309	146669	6.1	Protein KIAA0664
344	E9PM46	28	35	27.1	1355	154697	5.1	Uncharacterized protein
345	Q5VW36	28	34	23.7	1801	200071	6.6	Uncharacterized protein KIAA1797
346	Q3TFG3	27	209	79.3	406	46182	5.5	Putative uncharacterized protein
347	Q8K0E8	27	100	67.8	481	54753	7.1	Fibrinogen beta chain
348	E9Q1S3	27	91	59.2	736	82956	7.5	Uncharacterized protein
348	Q01405	27	91	57	765	86162	7.1	Protein transport protein Sec23A
349	D3YY36	27	63	57.9	622	68601	7.9	Uncharacterized protein
349	Q9DBD0	27	63	51.4	700	76766	7.2	Putative uncharacterized protein
350	Q920B9	27	59	31.2	1047	119825	5.7	FACT complex subunit SPT16
350	UPI0000026527	27	59	31.2	1047	119839	5.7	FACT complex subunit SPT16
351	P26641	27	277	77.6	437	50119	6.7	Elongation factor 1-gamma
351	B4DTG2	27	277	69.6	487	56150	7.7	cDNA FLJ56389, highly similar to Elongation factor 1-gamma
352	P28838-2	27	79	73.8	488	52771	6.7	Isoform 2 of Cytosol aminopeptidase
352	P28838	27	79	69.4	519	56166	7.9	Cytosol aminopeptidase
353	B3KQT9	27	100	64	480	54103	7.2	cDNA PSEC0175 fis, clone OVARC1000169, highly similar to Protein disulfide-isomerase A3 (EC 5.3.4.1)
353	P30101	27	100	60.8	505	56782	6.4	Protein disulfide-isomerase A3
354	P17858	27	111	53.8	780	85018	7.5	6-phosphofructokinase, liver type
355	B2RBA6	27	83	46.6	719	81280	6.5	cDNA, FLJ95407, highly similar to sapiens MCM7 minichromosome maintenance deficient 7 (S. cerevisiae) (MCM7), mRNA
355	P33993	27	83	46.6	719	81308	6.5	DNA replication licensing factor MCM7
356	O43719	27	49	46.2	755	85853	4.4	HIV Tat-specific factor 1
357	P23246	27	281	43.8	707	76150	9.4	Splicing factor, proline- and glutamine-rich
358	O43592	27	70	41.5	962	109964	5.4	Exportin-T
359	A8QI98	27	32	39.2	958	109058	7.1	DIS3
360	Q5JSH3	27	31	38.9	913	101366	5.5	WD repeat-containing protein 44
361	A8K2P8	27	84	38.1	901	99934	5.3	cDNA FLJ76245, highly similar to sapiens desmocollin 2 (DSC2), transcript variant Dsc2a, mRNA
361	Q02487	27	84	38.1	901	99962	5.3	Desmocollin-2
361	A9X9L0	27	84	38.1	901	99950	5.3	Desmocollin 2
361	B4DLJ5	27	84	37.6	912	101083	5.3	cDNA FLJ55716, highly similar to Desmocollin-2
361	E9PHQ8	27	84	37.5	914	101400	5.3	Uncharacterized protein
362	Q9Y3P9	27	33	30.6	1069	121737	5.3	Rab GTPase-activating protein 1
363	Q8TAQ2-2	27	128	28.7	1130	124841	5.6	Isoform 2 of SWI/SNF complex subunit SMARCC2

Table S1. Proteins identified in the OVCAR-3 tumor supernatant by two or more unique peptides.

Protein or Protein Group	Accession Number	Unique Peptide Count	Spectrum Count	Sequence Coverage (%)	Length	MolWt	pI	Descriptive Name
363	Q59G16	27	128	28	1156	127377	5.5	SWI/SNF-related matrix-associated actin-dependent regulator of chromatin c2 isoform b variant (Fragment)
363	Q59GV3	27	128	27.8	1164	128088	5.6	SWI/SNF-related matrix-associated actin-dependent regulator of chromatin c2 isoform b variant (Fragment)
363	Q8TAQ2	27	128	26.7	1214	132879	5.7	SWI/SNF complex subunit SMARCC2
364	Q9D8N0	26	221	76.4	437	50061	6.7	Elongation factor 1-gamma
365	P27773	26	83	58	505	56678	6.2	Protein disulfide-isomerase A3
366	Q61702	26	150	41.9	907	101083	7	Inter-alpha-trypsin inhibitor heavy chain H1
366	Q91WU7	26	150	41.8	909	101343	7.3	Inter-alpha trypsin inhibitor, heavy chain 1 (Fragment)
367	Q3UGT5	26	79	38.6	843	96672	6.8	Phosphorylase
367	Q8CI94	26	79	38.6	843	96730	6.7	Glycogen phosphorylase, brain form
367	Q3V3U0	26	79	38.6	843	96716	6.7	Phosphorylase
367	Q3UYH9	26	79	38.6	843	96762	6.7	Phosphorylase
368	B2RUJ7	26	33	27	1335	146560	7.6	Xanthine dehydrogenase
368	Q00519	26	33	27	1335	146518	7.6	Xanthine dehydrogenase/oxidase
368	E9QLM9	26	33	27	1335	146562	7.6	Uncharacterized protein
369	P35441	26	75	26.5	1170	129647	5	Thrombospondin-1
369	Q8CGB2	26	75	26.5	1171	129718	5	Thrombospondin 1
370	E9QJZ4	26	34	13.2	2907	313818	4.8	Uncharacterized protein
371	Q96KP4	26	80	73.9	475	52878	6	Cytosolic non-specific dipeptidase
372	Q9NTK5	26	62	73.7	396	44744	7.8	Obg-like ATPase 1
373	Q9Y230	26	59	65.4	463	51157	5.6	RuvB-like 2
374	E7ES34	26	402	59.6	445	48975	5.4	Uncharacterized protein
375	Q9NY33	26	115	59	737	82589	5.1	Dipeptidyl peptidase 3
375	UPI0000EE4697	26	115	57.5	757	84376	5	UPI0000EE4697 UniRef100 entry
376	P07237	26	120	55.7	508	57116	4.9	Protein disulfide-isomerase
377	O43242	26	53	54.5	534	60978	8.4	26S proteasome non-ATPase regulatory subunit 3
377	Q6IBN0	26	53	54.5	534	60946	8.4	PSMD3 protein
378	B4E0X8	26	231	51.2	629	66232	7.6	cDNA FLJ61021, highly similar to Far upstream element-binding protein 1
379	P56192	26	60	39.9	900	101116	6.2	Methionyl-tRNA synthetase, cytoplasmic
380	P52272-2	26	161	40.2	691	73621	8.8	Isoform 2 of Heterogeneous nuclear ribonucleoprotein M
380	P52272	26	161	38.1	730	77516	8.7	Heterogeneous nuclear ribonucleoprotein M
381	E9PMZ9	26	87	38.3	827	92409	4.6	Uncharacterized protein
381	E9PKF6	26	87	37.8	838	93733	4.6	Uncharacterized protein
381	Q5H9R7-6	26	87	37.6	844	94445	4.6	Isoform 6 of Serine/threonine-protein phosphatase 6 regulatory subunit 3
381	Q5H9R7-2	26	87	36.6	867	96957	4.6	Isoform 2 of Serine/threonine-protein phosphatase 6 regulatory subunit 3
381	Q5H9R7	26	87	36.3	873	97669	4.6	Serine/threonine-protein phosphatase 6 regulatory subunit 3
381	Q5H9R7-5	26	87	36.1	879	98485	4.6	Isoform 5 of Serine/threonine-protein phosphatase 6 regulatory subunit 3
382	O95163	26	29	29.1	1332	150254	5.9	Elongator complex protein 1
383	Q14112-2	26	54	26.6	1274	140866	5.3	Isoform 2 of Nidogen-2
383	Q14112	26	54	24.7	1375	151254	5.3	Nidogen-2
384	Q92878	26	51	21.2	1312	153892	6.9	DNA repair protein RAD50
384	Q92878-2	26	51	21.1	1318	154587	6.9	Isoform 2 of DNA repair protein RAD50
385	P07942	26	100	17.4	1786	198037	4.9	Laminin subunit beta-1
385	UPI0000EE4033	26	100	17.1	1810	200477	5	Laminin subunit beta-1 precursor (Laminin B1 chain).
386	Q15746-4	26	69	17.8	1794	197398	6.5	Isoform 3B of Myosin light chain kinase, smooth muscle
386	D3DN97	26	69	17.3	1845	203044	6.2	Myosin, light polypeptide kinase, isoform CRA_d
386	Q15746-2	26	69	17.3	1845	203054	6.2	Isoform 2 of Myosin light chain kinase, smooth muscle
386	Q15746-3	26	69	17.1	1863	205057	6.5	Isoform 3A of Myosin light chain kinase, smooth muscle
386	Q15746-6	26	69	16.7	1913	210584	6.2	Isoform Del-1790 of Myosin light chain kinase, smooth muscle
386	Q15746	26	69	16.7	1914	210713	6.2	Myosin light chain kinase, smooth muscle
387	P62258	25	888	83.9	255	29174	4.7	14-3-3 protein epsilon 14-3-3 protein epsilon
388	P07437	25	1600	72.7	444	49671	4.9	Tubulin beta chain Tubulin beta chain
389	P05064	25	147	78.8	364	39356	8.1	Fructose-bisphosphate aldolase A
389	A6ZI44	25	147	68.7	418	45120	7.9	Fructose-bisphosphate aldolase
390	P32261	25	158	60.4	465	52004	6.5	Antithrombin-III
391	Q69ZZ7	25	150	48.7	729	82055	6.6	MKIAA0762 protein (Fragment)
391	Q8VCC9	25	150	44	807	90821	6	Spondin-1
392	Q91VR5	25	157	48.2	740	82500	7.2	ATP-dependent RNA helicase DDX1
393	Q9WVS5	25	48	45.5	547	59569	5.6	Chaperonin containing TCP-1 theta subunit
393	P42932	25	48	45.4	548	59556	5.6	T-complex protein 1 subunit theta
393	Q8BVY8	25	48	45.4	548	59555	5.8	Putative uncharacterized protein
393	Q3UKQ2	25	48	45.4	548	59557	5.5	Putative uncharacterized protein
394	P27546	25	76	32.8	1125	117429	5	Microtubule-associated protein 4
394	P27546-2	25	76	32.8	1124	117343	5	Isoform 2 of Microtubule-associated protein 4
395	Q64514-2	25	29	29.6	1249	138463	6.4	Isoform Short of Tripeptidyl-peptidase 2
396	Q05D44	25	88	28.6	1216	137616	5.6	Eukaryotic translation initiation factor 5B
397	Q5XK22	25	31	30.8	1187	137142	6.4	Ace protein (Fragment)
397	P09470	25	31	27.9	1312	150918	6.6	Angiotensin-converting enzyme
398	P60174	25	436	92.4	249	26669	6.9	Triosephosphate isomerase
399	Q8IWP6	25	776	72.6	445	49753	4.9	Class IVb beta tubulin
400	D3DPU2	25	134	70.5	475	51673	8	Adenylyl cyclase-associated protein
401	E9PC18	25	131	68.2	532	60208	8.5	Uncharacterized protein
401	P06744	25	131	65.1	558	63147	8.3	Glucose-6-phosphate isomerase
402	P78371	25	54	67.7	535	57488	6.4	T-complex protein 1 subunit beta
403	P23526	25	87	66	432	47716	6.3	Adenosylcysteinase
404	P68104	25	920	64.9	462	50141	9	Elongation factor 1-alpha 1
404	Q6IPT9	25	920	64.9	462	50185	9.1	Elongation factor 1-alpha
404	Q6IPN6	25	920	64.9	462	50123	9	Elongation factor 1-alpha
404	Q53HR5	25	920	64.9	462	50125	9	Elongation factor 1-alpha (Fragment)
404	Q53HQ7	25	920	64.9	462	50199	8.9	Elongation factor 1-alpha (Fragment)
404	Q53HM9	25	920	64.9	462	50142	8.9	Elongation factor 1-alpha (Fragment)
405	A6NJA2	25	60	64.3	459	52386	5.8	Ubiquitin carboxyl-terminal hydrolase
405	P54578	25	60	59.7	494	56069	5.3	Ubiquitin carboxyl-terminal hydrolase 14
405	UPI00015DFA37	25	60	55.5	532	59930	7.6	Ubiquitin carboxyl-terminal hydrolase 14 (EC 3.1.2.15) (Ubiquitin thioesterase 14) (Ubiquitin-specific-processing protease 14) (Deubiquitinating enzyme 14).
406	B3KX11	25	76	63	522	57946	6.9	cDNA FLJ44436 fis, clone UTERU2019706, highly similar to T-complex protein 1 subunit gamma
406	Q5SZY0	25	76	63	522	57972	6.9	Chaperonin containing TCP1, subunit 3 (Gamma)
406	E9PAQ6	25	76	60.5	544	60463	6.5	Uncharacterized protein
406	P49368	25	76	60.4	545	60534	6.5	T-complex protein 1 subunit gamma
407	Q02790	25	89	61.7	459	51805	5.4	Peptidyl-prolyl cis-trans isomerase FKBP4
408	P00352	25	60	61.5	501	54862	6.7	Retinal dehydrogenase 1
409	P13798	25	66	59	732	81225	5.5	Acylamino-acid-releasing enzyme
409	C9JIF9	25	66	58.6	737	81674	5.5	Uncharacterized protein
410	Q9UBT2	25	109	57.5	640	71224	5.3	SUMO-activating enzyme subunit 2
411	P09960	25	94	57	611	69285	6.2	Leukotriene A-4 hydrolase
412	Q9Y262	25	61	54.1	564	66727	6.3	Eukaryotic translation initiation factor 3 subunit L
413	E7EU87	25	296	50.6	555	58901	6	Uncharacterized protein

Table S1. Proteins identified in the OVCAR-3 tumor supernatant by two or more unique peptides.

Protein or Protein Group	Accession Number	Unique Peptide Count	Spectrum Count	Sequence Coverage (%)	Length	MolWt	pI	Descriptive Name
414	P78347-2	25	52	37.1	957	107970	7.9	Isoform 2 of General transcription factor II-I
414	UPI0000E0AC33	25	52	36.4	976	110035	7.4	general transcription factor II-I isoform 5
414	Q499G6	25	52	36.4	976	110080	7.6	General transcription factor II, i
414	P78347-4	25	52	36.3	977	110106	7.4	Isoform 4 of General transcription factor II-I
414	P78347-3	25	52	36.3	978	110280	6.7	Isoform 3 of General transcription factor II-I
414	P78347	25	52	35.6	998	112416	6.4	General transcription factor II-I
415	Q9Y6D5	25	57	29.1	1785	202037	6.3	Brefeldin A-inhibited guanine nucleotide-exchange protein 2
416	C9J4D0	25	31	23.1	1458	162985	5.4	Uncharacterized protein
416	Q08378	25	31	22.5	1498	167354	5.4	Golgin subfamily A member 3
417	P11388	25	37	21	1531	174384	8.7	DNA topoisomerase 2-alpha
417	P11388-2	25	37	20.6	1557	177500	8.8	Isoform 2 of DNA topoisomerase 2-alpha
417	C9J5P2	25	37	20.5	1568	178882	8.7	DNA topoisomerase 2
417	P11388-3	25	37	20.5	1567	178711	8.8	Isoform 3 of DNA topoisomerase 2-alpha
417	P11388-4	25	37	19.9	1612	182680	8.6	Isoform 4 of DNA topoisomerase 2-alpha
418	A5YKK6	25	58	12.9	2376	266937	7.1	CCR4-NOT transcription complex subunit 1
418	A5YKK6-2	25	58	12.9	2371	266379	7	Isoform 2 of CCR4-NOT transcription complex subunit 1
419	Q01484	25	38	8.8	3924	430259	5.1	Ankyrin-2
420	Q8WVW5	24	2210	86.2	363	40503	6.1	Putative uncharacterized protein (Fragment)
420	P60709	24	2210	83.5	375	41737	5.5	Actin, cytoplasmic 1 Actin, cytoplasmic 1
420	Q53G99	24	2210	83.5	375	41765	5.6	Beta actin variant (Fragment)
420	Q3UAF7	24	2210	83.5	375	41751	5.5	Putative uncharacterized protein
420	Q3U5R4	24	2210	83.5	375	41709	5.5	Putative uncharacterized protein
420	P63261	24	2210	83.5	375	41793	5.5	Actin, cytoplasmic 2 Actin, cytoplasmic 2
421	Q14240	24	264	72	407	46402	5.5	Eukaryotic initiation factor 4A-II Eukaryotic initiation factor 4A-II
421	Q14240-2	24	264	71.8	408	46489	5.5	Isoform 2 of Eukaryotic initiation factor 4A-II Isoform 2 of Eukaryotic initiation factor 4A-II
422	D3YZP9	24	135	53.5	469	52939	7.3	MCG115019
422	Q16204	24	135	53	474	53291	7.3	Coiled-coil domain-containing protein 6
422	UPI000013D3A6	24	135	43.1	583	65774	7.3	Coiled-coil domain-containing protein 6 (H4 protein) (Papillary thyroid carcinoma-encoded protein).
423	Q99LG2	24	75	41.4	887	100456	5	Transportin-2
423	Q6IN77	24	75	41.4	887	100408	5	Transportin 2
423	Q4LE60	24	75	34.9	1051	117677	5.6	TNPO2 variant protein (Fragment)
424	P63101	24	609	86.1	245	27771	4.8	14-3-3 protein zeta/delta
425	P17182	24	905	68.2	434	47141	6.8	Alpha-enolase
426	Q3TJX0	24	63	63.3	627	70135	5.3	Putative uncharacterized protein
426	Q61233	24	63	63.3	627	70149	5.3	Plastin-2
427	Q8QZY1	24	53	52.3	564	66613	6.4	Eukaryotic translation initiation factor 3 subunit L
428	Q9WU78	24	137	33.1	869	96010	6.5	Programmed cell death 6-interacting protein
428	Q3TED2	24	137	33.1	869	96024	6.5	Programmed cell death 6 interacting protein
428	B8JJL8	24	137	33	874	96773	6.5	Programmed cell death 6 interacting protein
428	Q9WU78-3	24	137	33	874	96759	6.5	Isoform 3 of Programmed cell death 6-interacting protein
428	Q80Y09	24	137	33	872	96312	6.5	Pdcd6ip protein
429	Q3UPL0-2	24	115	22.7	1191	129564	7.4	Isoform 2 of Protein transport protein Sec31A
429	Q3UPL0	24	115	22	1230	133569	6.8	Protein transport protein Sec31A
430	P63104	24	615	86.1	245	27745	4.8	14-3-3 protein zeta/delta
431	P15121	24	136	77.5	316	35853	7	Aldose reductase
432	P07355	24	167	68.7	339	38604	7.8	Annexin A2
433	P49419	24	52	61.6	539	58487	8	Alpha-aminoadipic semialdehyde dehydrogenase
434	P26038	24	132	59.3	577	67820	6.4	Moesin
435	P48643	24	57	58.4	541	59671	5.6	T-complex protein 1 subunit epsilon
436	P17987	24	49	58.3	556	60344	6.1	T-complex protein 1 subunit alpha
437	P14868	24	59	58.1	501	57136	6.5	Aspartyl-tRNA synthetase, cytoplasmic
438	P31751	24	65	57	481	55769	6.4	RAC-beta serine/threonine-protein kinase
439	Q14651	24	86	55.6	629	70254	5.4	Plastin-1
440	P50895	24	146	55.4	628	67405	5.8	Basal cell adhesion molecule
441	P38606	24	90	52.7	617	68304	5.5	V-type proton ATPase catalytic subunit A
442	B3KWV3	24	59	47.9	702	80460	6.3	cDNA FLJ43930 fis, clone TESTI4013441, highly similar to 1,4-alpha-glucan branching enzyme (EC 2.4.1.18)
442	Q04446	24	59	47.9	702	80460	6.3	1,4-alpha-glucan-branching enzyme
443	B7Z468	24	118	47.6	754	80999	5.1	cDNA FLJ56102, highly similar to sapiens calpastatin (CAST), transcript variant 8, mRNA
443	P20810-5	24	118	47.5	756	81169	5.2	Isoform 5 of Calpastatin
443	E7EQQ6	24	118	46.7	769	82437	5.2	Uncharacterized protein
444	P17844	24	166	45.9	614	69148	8.9	Probable ATP-dependent RNA helicase DDX5
445	Q92945	24	130	45.9	711	73115	7.3	Far upstream element-binding protein 2
446	P48444	24	137	49.5	511	57210	6.2	Coatomer subunit delta
446	B0YIW6	24	137	45.8	552	61626	5.8	Archain 1, isoform CRA_a
446	Q6MZV5	24	137	45.8	552	61598	5.8	Putative uncharacterized protein DKFZp686M09245
447	Q9Y263	24	69	42.9	795	87157	6.4	Phospholipase A-2-activating protein
448	A8K9D9	24	36	39.4	899	96678	6.2	Nuclear factor of kappa light polypeptide gene enhancer in B-cells 2 (P49/p100), isoform CRA_a
448	Q00653	24	36	39.3	900	96749	6.2	Nuclear factor NF-kappa-B p100 subunit
449	O15397	24	55	38	1037	119938	5.2	Importin-8
450	Q86XP3	24	109	37.1	938	102975	7	ATP-dependent RNA helicase DDX42
451	Q9H2U1	24	63	31.5	1008	114760	7.7	Probable ATP-dependent RNA helicase DHX36
452	D6W633	24	28	27.9	1310	143431	6.2	Protein tyrosine phosphatase, receptor type, S, isoform CRA_a
452	Q13332-6	24	28	19.1	1910	212484	6.7	Isoform 2 of Receptor-type tyrosine-protein phosphatase S
452	Q13332-3	24	28	18.9	1928	214656	6.5	Isoform PTPS-MEB of Receptor-type tyrosine-protein phosphatase S
452	Q13332-4	24	28	18.8	1939	215928	6.6	Isoform PTPS-MEC of Receptor-type tyrosine-protein phosphatase S
452	Q13332-2	24	28	18.8	1939	215978	6.5	Isoform PTPS-MEA of Receptor-type tyrosine-protein phosphatase S
452	Q13332	24	28	18.7	1948	217039	6.5	Receptor-type tyrosine-protein phosphatase S
453	Q13439-4	24	41	13.1	2228	260948	5.4	Isoform 4 of Golgin subfamily A member 4
453	Q13439-3	24	41	13.1	2223	260322	5.4	Isoform 3 of Golgin subfamily A member 4
453	Q13439	24	41	13.1	2230	261137	5.4	Golgin subfamily A member 4
453	E9PH44	24	41	13	2243	262325	5.4	Uncharacterized protein
454	Q8NFC6	24	28	11.3	3051	330467	5.1	Biorientation of chromosomes in cell division protein 1-like
455	Q709C8-4	24	28	9.9	3584	402957	6.4	Isoform 4 of Vacuolar protein sorting-associated protein 13C
455	Q709C8-2	24	28	9.8	3627	408072	6.6	Isoform 2 of Vacuolar protein sorting-associated protein 13C
455	Q709C8-3	24	28	9.5	3710	417279	6.6	Isoform 3 of Vacuolar protein sorting-associated protein 13C
455	Q709C8	24	28	9.4	3753	422393	6.8	Vacuolar protein sorting-associated protein 13C
456	P68363	23	1742	77.2	451	50152	5.1	Tubulin alpha-1B chain Tubulin alpha-1B chain
457	Q13838	23	206	66.6	428	48991	5.7	Spliceosome RNA helicase DDX39B
457	Q9Z1N5	23	206	66.6	428	49035	5.7	Spliceosome RNA helicase Ddx39b
458	P06151	23	493	80.1	332	36499	7.7	L-lactate dehydrogenase A chain
459	Q6ZWX6	23	75	74.9	315	36108	5.1	Eukaryotic translation initiation factor 2 subunit 1
460	Q3TWE3	23	86	53.6	509	57087	4.9	Putative uncharacterized protein
461	P14685	23	46	45.8	530	60699	8.2	26S proteasome non-ATPase regulatory subunit 3
461	Q3TP95	23	46	45.8	530	60718	8.4	Proteasome (Prosome, macropain) 26S subunit, non-ATPase, 3
462	Q8VCQ8	23	213	40.6	530	60453	7.4	Caldesmon 1
462	E9Q0M9	23	213	40.3	534	61652	6.9	Uncharacterized protein

Table S1. Proteins identified in the OVCAR-3 tumor supernatant by two or more unique peptides.

Protein or Protein Group	Accession Number	Unique Peptide Count	Spectrum Count	Sequence Coverage (%)	Length	MolWt	pI	Descriptive Name
462	E9QA16	23	213	40.1	536	61837	6.9	Uncharacterized protein
463	Q3THQ5	23	105	39	543	62501	6.7	Putative uncharacterized protein
463	Q60864	23	105	39	543	62582	6.8	Stress-induced-phosphoprotein 1
464	Q3TVV6	23	199	37.1	800	87946	6.2	Putative uncharacterized protein
464	Q8VEK3	23	199	37.1	800	87918	6.2	Heterogeneous nuclear ribonucleoprotein U
464	Q3ULH5	23	199	37.1	800	87846	6.3	Putative uncharacterized protein
465	E9QME9	23	91	35.5	889	99358	6	Uncharacterized protein
465	Q61704	23	91	35.5	889	99366	6	Inter-alpha-trypsin inhibitor heavy chain H3
466	Q61703	23	136	34.6	946	105928	7.3	Inter-alpha-trypsin inhibitor heavy chain H2
467	Q3TZ32	23	82	30.8	968	106860	5.7	Putative uncharacterized protein
467	Q8BGQ7	23	82	30.8	968	106908	5.7	Alanyl-tRNA synthetase, cytoplasmic
467	Q3UD67	23	82	30.8	968	106878	5.7	Putative uncharacterized protein
468	E9QQ65	23	103	17.2	1280	147031	6	Uncharacterized protein
468	E9QQ64	23	103	16.9	1304	149942	5.9	Uncharacterized protein
468	UPI0001278276	23	103	16.6	1327	152543	5.9	kinectin
469	P13489	23	73	81.3	461	49974	4.8	Ribonuclease inhibitor
470	Q9BQE3	23	787	77.1	449	49895	5.1	Tubulin alpha-1C chain
471	Q9Y2T3	23	62	76.7	454	51003	5.7	Guanine deaminase
472	P43686	23	98	75.8	418	47366	5.2	26S protease regulatory subunit 6B
473	P05198	23	83	74.9	315	36112	5.1	Eukaryotic translation initiation factor 2 subunit 1
474	P62136	23	101	73.9	330	37512	6.3	Serine/threonine-protein phosphatase PP1-alpha catalytic subunit
475	P27797	23	71	72.9	417	48142	4.4	Calreticulin
476	P14866	23	198	71.8	589	64133	8.2	Heterogeneous nuclear ribonucleoprotein L
477	Q9NZU5	23	62	67.9	365	40833	7.9	LIM and cysteine-rich domains protein 1
478	Q13153	23	81	59.6	545	60647	5.8	Serine/threonine-protein kinase PAK 1
479	UPI00003D2EAD	23	72	53.9	666	74798	5.9	xaa-Pro aminopeptidase 1 isoform 1
480	P27694	23	78	53.6	616	68138	7.2	Replication protein A 70 kDa DNA-binding subunit
481	Q15046	23	91	52.4	597	68048	6.4	Lysyl-tRNA synthetase
481	Q15046-2	23	91	50.1	625	71497	6.8	Isoform Mitochondrial of Lysyl-tRNA synthetase
482	B2R8Z8	23	169	47.4	623	69602	8.8	cDNA, FLJ94136, highly similar to sapiens synaptotagmin binding, cytoplasmic RNA interacting protein (SYNCRIP), mRNA
482	O60506	23	169	47.4	623	69603	8.6	Heterogeneous nuclear ribonucleoprotein Q
483	Q05682-5	23	172	43.8	532	61213	6.7	Isoform 5 of Caldesmon
483	Q05682-4	23	172	43.3	538	62663	6.4	Isoform 4 of Caldesmon
483	B7Z6G4	23	172	42.9	543	62742	5.9	cDNA FLJ53193, highly similar to sapiens caldesmon 1 (CALD1), transcript variant 5, mRNA
484	P23921	23	37	39.1	792	90070	7.1	Ribonucleoside-diphosphate reductase large subunit
485	P51003	23	35	39.1	745	82843	7.4	Poly(A) polymerase alpha
485	UPI00015DFDD9	23	35	39.1	744	82651	7.4	Poly(A) polymerase alpha (EC 2.7.7.19) (PAP) (Polynucleotide adenylyltransferase alpha).
486	Q8N163	23	95	39	923	102902	5.2	Protein KIAA1967
487	P41250	23	63	38.6	739	83166	7	Glycyl-tRNA synthetase
488	Q9NR30	23	70	35.5	783	87344	9.3	Nucleolar RNA helicase 2
489	P55285	23	167	34.6	790	88309	4.9	Cadherin-6
490	B3KMC9	23	47	32.3	950	108613	7.5	cDNA FLJ10711 fis, clone NT2RP3000917, highly similar to 5'-3' exoribonuclease 2 (EC 3.1.11.-)
490	Q9H0D6	23	47	32.3	950	108583	7.5	5'-3' exoribonuclease 2
491	P53992	23	119	31.2	1094	118325	7.1	Protein transport protein Sec24C
492	Q24JP5	23	61	31.2	1023	110110	5.6	Transmembrane protein 132A
492	Q24JP5-2	23	61	31.2	1024	110197	5.6	Isoform 2 of Transmembrane protein 132A
493	Q9UHP3	23	33	27	1055	122218	5.3	Ubiquitin carboxyl-terminal hydrolase 25
493	UPI00015DF741	23	33	26.2	1086	125635	5.4	Ubiquitin carboxyl-terminal hydrolase 25 (EC 3.1.2.15) (Ubiquitin thioesterase 25) (Ubiquitin-specific-processing protease 25) (Deubiquitinating enzyme 25) (USP on chromosome 21).
493	Q9UHP3-1	23	33	26.2	1087	125750	5.4	Isoform USP25b of Ubiquitin carboxyl-terminal hydrolase 25
493	Q9UHP3-3	23	33	25.3	1125	129963	5.4	Isoform USP25m of Ubiquitin carboxyl-terminal hydrolase 25
494	O76046	23	31	24.8	1246	137794	6.1	Putative RNA helicase Ski2w
494	Q5JP73	23	31	24.8	1246	137737	6.1	Superkiller viralicidic activity 2-like (S. cerevisiae)
494	Q15477	23	31	24.8	1246	137755	6.1	Helicase SKI2W
495	Q9Y2W1	23	135	24.7	955	108666	10.2	Thyroid hormone receptor-associated protein 3
496	B4DHJ3	23	38	23.8	1167	133027	7.2	DNA-directed RNA polymerase
496	C9J2Y9	23	38	23.8	1167	133057	7.2	DNA-directed RNA polymerase
496	P30876	23	38	23.7	1174	133896	6.9	DNA-directed RNA polymerase II subunit RPB2
497	B3KMS0	23	59	23.4	1401	157195	6.6	Condensin complex subunit 1
497	Q15021	23	59	23.4	1401	157182	6.6	Condensin complex subunit 1
498	O60333-2	23	35	17.1	1770	199261	5.5	Isoform 2 of Kinesin-like protein KIF1B
498	Q4R9M7	23	35	17	1783	200574	5.5	Kinesin family member 1Bbeta isoform IV
498	Q4R9M8	23	35	16.9	1797	201950	5.5	Kinesin family member 1Bbeta isoform III
498	O60333	23	35	16.7	1816	204475	5.6	Kinesin-like protein KIF1B
498	Q4R9M9	23	35	16.7	1809	203641	5.6	Kinesin family member 1Bbeta isoform II
498	Q4VXC4	23	35	16.6	1823	205139	5.5	Kinesin family member 1B
499	E9PKG1	22	70	74.5	325	37709	6.1	Uncharacterized protein
499	Q8C2D7	22	70	70.6	343	39562	5.6	Putative uncharacterized protein
499	Q99873-2	22	70	69.7	347	39929	5.6	Isoform 2 of Protein arginine N-methyltransferase 1
499	Q80WB1	22	70	69.1	350	40292	5.8	Prmt1 protein (Fragment)
499	Q8WUW5	22	70	68.8	352	40417	5.5	PRMT1 protein (Fragment)
499	Q63009	22	70	68.6	353	40522	5.6	Protein arginine N-methyltransferase 1
499	Q80WV6	22	70	68.4	354	40759	5.7	Prmt1 protein
499	Q99873	22	70	67	361	41516	5.4	Protein arginine N-methyltransferase 1
499	Q9JIF0	22	70	65.2	371	42436	5.4	Protein arginine N-methyltransferase 1
500	UPI0000D62797	22	676	64.2	352	37270	9.1	heterogeneous nuclear ribonucleoprotein A2/B1
500	O88569	22	676	64	353	37403	8.9	Heterogeneous nuclear ribonucleoproteins A2/B1
500	P22626	22	676	64	353	37430	8.9	Heterogeneous nuclear ribonucleoproteins A2/B1
501	P62191	22	94	62.3	440	49185	6.2	26S protease regulatory subunit 4 26S protease regulatory subunit 4
501	Q53HB3	22	94	62.3	440	49213	6.2	Proteasome 26S ATPase subunit 1 variant (Fragment)
502	P35998	22	51	59.1	433	48634	5.9	26S protease regulatory subunit 7
502	P46471	22	51	59.1	433	48648	5.9	26S protease regulatory subunit 7
503	Q5T6W5	22	1092	54.7	428	47557	5.6	Heterogeneous nuclear ribonucleoprotein K
503	P61979-3	22	1092	53.3	439	48511	5.9	Isoform 3 of Heterogeneous nuclear ribonucleoprotein K Isoform 3 of Heterogeneous nuclear ribonucleoprotein K
503	B2M1R6	22	1092	53.2	440	48562	5.5	Heterogeneous nuclear ribonucleoprotein K Heterogeneous nuclear ribonucleoprotein K
503	E9Q8D6	22	1092	52	450	49879	5.9	Uncharacterized protein
503	E9PVM4	22	1092	51.1	458	50855	6.4	Uncharacterized protein
504	O35286	22	71	40.5	795	91007	7.5	Putative pre-mRNA-splicing factor ATP-dependent RNA helicase DHX15
504	Q3TFE5	22	71	40.5	795	91023	7.4	Putative uncharacterized protein
504	O43143	22	71	40.5	795	90933	7.5	Putative pre-mRNA-splicing factor ATP-dependent RNA helicase DHX15
505	Q13616	22	38	39	776	89678	8	Cullin-1
505	Q9WTX6	22	38	39	776	89691	8	Cullin-1

Protein or Protein Group	Accession Number	Unique Peptide Count	Spectrum Count	Sequence Coverage (%)	Length	MolWt	pI	Descriptive Name
505	Q3UIA5	22	38	39	776	89677	8	Putative uncharacterized protein
506	Q3UBP6	22	1069	79.7	375	41769	5.5	Putative uncharacterized protein
507	P68373	22	824	72.8	449	49909	5.1	Tubulin alpha-1C chain
508	P10126	22	859	64.3	462	50114	9	Elongation factor 1-alpha 1
508	Q3UA81	22	859	64.3	462	50113	9.1	Elongation factor 1-alpha
509	P07356	22	153	63.7	339	38676	7.7	Annexin A2
510	P40124	22	70	63.3	474	51575	7.5	Adenylyl cyclase-associated protein 1
510	Q8BPT7	22	70	63.3	474	51565	7.5	Adenylyl cyclase-associated protein
511	P50247	22	71	60.4	432	47688	6.5	Adenosylhomocysteinase
512	P07759	22	385	59.8	418	46880	5.2	Serine protease inhibitor A3K
513	Q3U6X6	22	58	54.4	562	61369	6.4	Putative uncharacterized protein
513	Q5RJV4	22	58	54.4	562	61384	6.6	Phosphoglucomutase 2
513	Q66JR7	22	58	51.9	590	64158	6.8	Pgm2 protein (Fragment)
514	P23953	22	151	52.5	554	61172	5.2	Liver carboxylesterase N
515	O89020	22	64	48.4	608	69379	5.8	Afamin
515	O89020-3	22	64	48.1	611	69666	5.6	Isoform 3 of Afamin
515	E9Q826	22	64	46.4	634	72245	5.6	Uncharacterized protein
516	Q62009-5	22	126	45	783	87098	7.7	Isoform 5 of Periostin
516	Q62009-3	22	126	43.5	810	89987	7.9	Isoform 3 of Periostin
516	Q62009-2	22	126	43.4	811	90255	7.3	Isoform 2 of Periostin
516	Q62009	22	126	42	838	93144	7.5	Periostin
517	P08003	22	65	37	638	71982	5.3	Protein disulfide-isomerase A4
518	Q3TCS0	22	58	35.9	710	80682	5.7	Putative uncharacterized protein
518	Q9QUR6	22	58	35.9	710	80752	5.7	Prolyl endopeptidase
519	E9PV24	22	118	34.5	789	87429	6.1	Uncharacterized protein
520	Q3B7Z2	22	54	29.6	751	84689	7.2	Oxysterol-binding protein 1
520	E9QPD4	22	54	27.6	805	88797	7.2	Uncharacterized protein
521	A6PWC3	22	43	25.2	1117	127773	4.9	Nardilysin, N-arginine dibasic convertase, NRD convertase 1
521	Q8R320	22	43	24.2	1161	132877	4.9	Nardilysin, N-arginine dibasic convertase, NRD convertase 1
521	Q8BHG1	22	43	24.2	1161	132891	4.9	Nardilysin
521	A2A9Q2	22	43	22.9	1229	140620	4.9	Nardilysin, N-arginine dibasic convertase, NRD convertase 1
522	O70481	22	28	17.5	1757	200215	6	E3 ubiquitin-protein ligase UBR1
522	Q2M4I1	22	28	17.5	1757	200203	6	Ubiquitin protein ligase E3 component n-recognin 1
523	P08758	22	69	72.8	320	35937	5	Annexin A5
524	B5BUB1	22	50	70.2	456	50216	6.4	RuvB-like 1 (Fragment)
524	Q9Y265	22	50	70.2	456	50228	6.4	RuvB-like 1
525	P07910	22	382	67.3	306	33670	5.1	Heterogeneous nuclear ribonucleoproteins C1/C2
526	Q8N995	22	53	68.8	509	56238	5.4	cDNA FLJ38173 fis, clone FCBBF1000053, highly similar to HYDROXYMETHYLGLUTARYL-COA SYNTHASE, CYTOPLASMIC
526	Q01581	22	53	67.3	520	57294	5.4	Hydroxymethylglutaryl-CoA synthase, cytoplasmic
527	P17980	22	63	66.5	439	49204	5.2	26S protease regulatory subunit 6A
528	Q15008	22	51	63.5	389	45531	5.6	26S proteasome non-ATPase regulatory subunit 6
529	Q8NC51-3	22	293	61.8	393	43135	8.4	Isoform 3 of Plasminogen activator inhibitor 1 RNA-binding protein
530	Q32Q12	22	470	59.2	292	32642	8.5	Nucleoside diphosphate kinase
531	Q9H4M9	22	48	58.4	534	60627	6.8	EH domain-containing protein 1
532	Q9BY44	22	89	53.8	585	64990	8.9	Eukaryotic translation initiation factor 2A
533	P20700	22	92	50.9	586	66408	5.2	Lamin-B1
534	O60502	22	29	41.5	916	102915	4.9	Bifunctional protein NCOAT
535	Q9Y5L0-3	22	71	41	909	102541	5.6	Isoform 3 of Transportin-3
535	Q9Y5L0	22	71	40.4	923	104203	5.6	Transportin-3
536	Q8NE71	22	51	40.1	845	95926	6.8	ATP-binding cassette sub-family F member 1
537	Q13618-2	22	36	36	744	86234	8	Isoform 2 of Cullin-3
537	Q13618	22	36	34.9	768	88930	8.5	Cullin-3
538	Q15459	22	69	34	793	88886	5.2	Splicing factor 3A subunit 1
539	B7ZKS3	22	25	29.3	1035	119018	6	Ubiquitin specific peptidase 48
539	Q86UV5	22	25	29.3	1035	119032	6.1	Ubiquitin carboxyl-terminal hydrolase 48
539	B7ZKS7	22	25	28.9	1047	120526	6.1	USP48 protein
540	Q12768	22	30	26.6	1159	134286	7	WASH complex subunit strumpellin
541	Q5VTR2	22	24	26.5	975	113662	5.9	E3 ubiquitin-protein ligase BRE1A
542	A6N6J7	22	34	23.8	1440	162728	5.7	JARID1C protein
542	P41229	22	34	22	1560	175719	5.6	Lysine-specific demethylase 5C
542	B0QZ44	22	34	22	1559	175591	5.6	Jumonji, AT rich interactive domain 1C
543	D9ZGF8	22	28	23.8	1354	158243	5.9	Rho-associated, coiled-coil containing protein kinase 1
543	Q13464	22	28	23.8	1354	158174	5.9	Rho-associated protein kinase 1
544	Q9H3S7	22	48	18.7	1636	178972	6.9	Tyrosine-protein phosphatase non-receptor type 23
545	Q8IWV8-4	22	28	18.2	1755	200539	6.2	Isoform 4 of E3 ubiquitin-protein ligase UBR2
546	Q7Z3U7-2	22	42	17.7	1676	185982	6.2	Isoform 2 of Protein MON2 log
546	B7ZM73	22	42	17.6	1688	187318	6	MON2 protein
546	B9EGP5	22	42	17.4	1711	189688	6.1	MON2 protein
546	Q7Z3U7-4	22	42	17.3	1717	190357	6.1	Isoform 4 of Protein MON2 log
546	Q7Z3U7	22	42	17.3	1718	190486	6.1	Protein MON2 log
547	P62140	21	191	69.7	327	37187	6.2	Serine/threonine-protein phosphatase PP1-beta catalytic subunit Serine/threonine-protein phosphatase PP1-beta catalytic subunit
548	P61221	21	95	44.9	599	67314	8.3	ATP-binding cassette sub-family E member 1 ATP-binding cassette sub-family E member 1
548	Q3TJM9	21	95	44.9	599	67280	8.3	Putative uncharacterized protein
548	Q3TIU8	21	95	44.9	599	67215	8.3	Putative uncharacterized protein
549	A8K2R3	21	52	41.5	730	81170	6.2	cDNA FLJ75083, highly similar to sapiens amine oxidase (flavin containing) domain 2 (AOF2),mRNA
549	O60341	21	52	35.6	852	92903	6.5	Lysine-specific histone demethylase 1A
549	Q6ZQ88	21	52	35.5	853	92851	6.5	Lysine-specific histone demethylase 1A
549	A3KG93	21	52	34.7	873	94659	6.2	Amine oxidase (Flavin containing) domain 2
549	O60341-2	21	52	34.6	876	95155	6.2	Isoform 2 of Lysine-specific histone demethylase 1A
550	P17751	21	259	76.7	249	26713	7.3	Triosephosphate isomerase
551	P40142	21	88	52.3	623	67631	7.5	Transketolase
552	Q8C2E1	21	95	51.8	589	65323	5.1	Putative uncharacterized protein
553	O88342	21	47	49.3	606	66407	6.6	WD repeat-containing protein 1
553	Q80ZI9	21	47	45.4	658	72113	8.4	WD repeat domain 1 (Fragment)
554	Q62318	21	174	29.9	834	88847	5.8	Transcription intermediary factor 1-beta
555	Q640N1	21	78	25.3	1128	128365	5.1	Adipocyte enhancer-binding protein 1
556	B1ATS3	21	29	21.7	1169	128652	5.9	Ankyrin repeat and FYVE domain containing 1
556	Q810B6	21	29	21.7	1169	128604	5.9	Ankyrin repeat and FYVE domain-containing protein 1
556	B7ZP20	21	29	21.7	1169	128636	5.9	Ankyrin repeat and FYVE domain containing 1
557	O00151	21	239	79.3	329	36072	7	PDZ and LIM domain protein 1
558	P18669	21	282	79.1	254	28804	7.2	Phosphoglycerate mutase 1
558	UPI00001AF8FE	21	282	79.1	254	28850	7.2	UPI00001AF8FE UniRef100 entry
558	Q53G35	21	282	79.1	254	28832	7.2	Phosphoglycerate mutase (Fragment)
559	P28482	21	67	75.6	360	41390	7	Mitogen-activated protein kinase 1
560	P04083	21	123	66.5	346	38714	7	Annexin A1
561	P52209	21	108	65.4	483	53140	7.2	6-phosphogluconate dehydrogenase, decarboxylating
562	Q9UQ80	21	64	65	394	43787	6.6	Proliferation-associated protein 2G4
563	B7Z9L0	21	40	62.9	483	52331	6.6	T-complex protein 1 subunit delta

Table S1. Proteins identified in the OVCAR-3 tumor supernatant by two or more unique peptides.

Protein or Protein Group	Accession Number	Unique Peptide Count	Spectrum Count	Sequence Coverage (%)	Length	MolWt	pI	Descriptive Name
563	P50991	21	40	56.4	539	57924	7.8	T-complex protein 1 subunit delta
564	P50454	21	160	62.2	418	46441	8.7	Serpin H1
565	Q9Y617	21	64	68.9	370	40423	7.7	Phosphoserine aminotransferase
565	B4DHQ3	21	64	61.4	415	45355	9	Phosphoserine aminotransferase
565	UPI0001AE6DE5	21	64	61.4	415	45256	8.9	UPI0001AE6DE5 UniRef100 entry
566	P34896	21	49	57.8	483	53083	7.7	Serine hydroxymethyltransferase, cytosolic
567	P48637	21	36	56.3	474	52385	5.9	Glutathione synthetase
568	P49591	21	49	55.8	514	58777	6.4	Seryl-tRNA synthetase, cytoplasmic
568	Q5T5C7	21	49	53.5	536	61313	7.1	Seryl-tRNA synthetase
569	P35241	21	87	53.5	583	68564	6.4	Radixin
570	O43175	21	117	52	533	56651	6.7	D-3-phosphoglycerate dehydrogenase
571	Q9UHX1-2	21	133	50.4	542	58172	5.3	Isoform 2 of Poly(U)-binding-splicing factor PUF60
571	Q9UHX1	21	133	48.8	559	59876	5.3	Poly(U)-binding-splicing factor PUF60
572	P54577	21	43	46.6	528	59144	7	Tyrosyl-tRNA synthetase, cytoplasmic
573	Q08380	21	249	45	585	65331	5.3	Galectin-3-binding protein
574	O95782-2	21	57	43.9	955	105361	7.7	Isoform B of AP-2 complex subunit alpha-1
574	O95782	21	57	42.9	977	107546	7	AP-2 complex subunit alpha-1
575	Q9Y5K6	21	78	40.5	639	71451	6.4	CD2-associated protein
576	Q8TDB6	21	66	37.3	740	83554	8.1	E3 ubiquitin-protein ligase DTX3L
577	P19367-4	21	54	36.7	905	101104	7	Isoform 4 of Hexokinase-1
577	E9PCK0	21	54	36.7	905	101085	6.9	Uncharacterized protein
577	A8K7J7	21	54	36.2	917	102387	6.7	cDNA FLJ78173, highly similar to sapiens hexokinase 1 (HK1) mRNA
577	P19367-2	21	54	36.2	916	102201	6.6	Isoform 2 of Hexokinase-1
577	P19367	21	54	36.2	917	102486	6.8	Hexokinase-1
577	P19367-3	21	54	36	921	102738	6.8	Isoform 3 of Hexokinase-1
577	E7ENR4	21	54	34.9	952	106313	7.1	Uncharacterized protein
577	B3KXY9	21	54	34.9	952	106251	7.2	cDNA FLJ46359 fis, clone TESTI4049786, highly similar to Hexokinase-1 (EC 2.7.1.1)
578	B7Z5J7	21	52	35.2	739	82058	6.8	cDNA FLJ58682, highly similar to Vesicle-fusing ATPase (EC 3.6.4.6)
578	P46459	21	52	34.9	744	82594	7	Vesicle-fusing ATPase
579	Q1KMD3	21	69	33.6	747	85105	4.9	Heterogeneous nuclear ribonucleoprotein U-like protein 2
580	O75534	21	39	33.1	798	88885	6.2	Cold shock domain-containing protein E1
581	Q9UI26	21	72	31	975	112535	5.3	Importin-11
582	P19338	21	246	29.3	710	76615	4.7	Nucleolin
583	UPI00015DFF58	21	85	29.9	869	98118	7	Eukaryotic translation initiation factor 4 gamma 2 (eIF-4-gamma 2) (eIF-4G 2) (eIF4G 2) (p97) (Death-associated protein 5) (DAP-5).
583	P78344-2	21	85	29.9	869	98150	7	Isoform 2 of Eukaryotic translation initiation factor 4 gamma 2
583	P78344	21	85	28.7	907	102362	7.1	Eukaryotic translation initiation factor 4 gamma 2
583	Q59G42	21	85	27.7	940	105828	7.4	Eukaryotic translation initiation factor 4 gamma, 2 variant (Fragment)
583	B4DZF2	21	85	26.5	980	110260	8	cDNA FLJ59571, highly similar to Eukaryotic translation initiation factor 4gamma 2
584	Q9Y4E8-3	21	30	25.3	956	109796	5.2	Isoform 3 of Ubiquitin carboxyl-terminal hydrolase 15
585	Q8IY21	21	30	17.9	1712	197852	7.6	Probable ATP-dependent RNA helicase DDX60
586	Q12905	20	65	79.5	390	43062	5.3	Interleukin enhancer-binding factor 2 Interleukin enhancer-binding factor 2
586	Q3UXI9	20	65	79.5	390	43010	5.1	Putative uncharacterized protein
587	A8K3Z3	20	53	62.8	398	44784	8.2	Proteasome (Prosome, macropain) 26S subunit, ATPase, 5, isoform CRA_b
587	UPI0000D62378	20	53	62.2	402	45292	8.2	26S protease regulatory subunit 8 (Proteasome 26S subunit ATPase 5) (Proteasome subunit p45) (p45/SUG) (Thyroid hormone receptor- interacting protein 1) (TRIP1).
587	P62195	20	53	61.6	406	45626	7.5	26S protease regulatory subunit 8 26S protease regulatory subunit 8
588	Q8TC62	20	47	59	417	48659	8.6	Septin 7
588	Q8C2A3	20	47	58.9	418	48755	8.6	Putative uncharacterized protein
588	Q3UNN1	20	47	58.9	418	48677	8.6	Putative uncharacterized protein
588	Q3LIE9	20	47	58.9	418	48774	8.6	Predicted protein product of Nbla02942
588	E9Q1G8	20	47	58.9	418	48798	8.7	Uncharacterized protein
588	A8K3D0	20	47	56.4	436	50509	8.7	cDNA FLJ75185
588	O55131	20	47	56.4	436	50550	8.6	Septin-7
588	E7EPK1	20	47	56.4	436	50567	8.6	Uncharacterized protein
588	Q16181	20	47	56.3	437	50680	8.6	Septin-7
588	E9Q9F5	20	47	56.3	437	50649	8.6	Uncharacterized protein
588	UPI0000D61C26	20	47	56.2	438	50809	8.5	Septin-7 (CDC10 protein log).
589	P21614	20	89	68.9	476	53600	5.5	Vitamin D-binding protein
590	P14211	20	65	65.9	416	47995	4.5	Calreticulin
591	P48036	20	55	64.9	319	35752	5	Annexin A5
592	P50516	20	73	43.6	617	68326	5.6	V-type proton ATPase catalytic subunit A
593	P22892	20	96	33.7	822	91350	6.8	AP-1 complex subunit gamma-1
593	Q8CBB7	20	96	33.6	825	91722	6.8	Adaptor protein complex AP-1, gamma 1 subunit
593	Q3UKX8	20	96	33.6	825	91695	6.8	Putative uncharacterized protein
594	D3Z4Y4	20	329	28.6	563	60362	7.8	Uncharacterized protein
595	Q8BHN3	20	81	28.6	944	106911	6.1	Neutral alpha-glucosidase AB
595	A1A4T2	20	81	28	966	109404	6.2	Alpha glucosidase 2 alpha neutral subunit
596	Q32NY0	20	24	17.4	1376	157344	5.1	Ubiquitin carboxyl-terminal hydrolase
597	O00764	20	65	82.7	312	35102	6.1	Pyridoxal kinase
598	P25786	20	84	81.7	263	29556	6.6	Proteasome subunit alpha type-1
598	P25786-2	20	84	79.9	269	30239	7	Isoform Long of Proteasome subunit alpha type-1
599	O95861	20	56	79.9	308	33392	5.7	3'(2'),5'-bisphosphate nucleotidase 1
600	B2RDE8	20	148	77.5	240	26805	4.7	cDNA, FLJ96580, highly similar to sapiens hepatoma-derived growth factor (high-mobility group protein 1-like) (HDGF), mRNA
601	Q15435	20	47	75.8	360	41564	4.9	Protein phosphatase 1 regulatory subunit 7
602	Q15181	20	397	74.7	289	32660	5.9	Inorganic pyrophosphatase
603	P27695	20	119	72.6	318	35554	8.1	DNA-(apurinic or apyrimidinic site) lyase
604	P61158	20	81	72	418	47371	5.9	Actin-related protein 3
605	Q6IBS0	20	127	69.1	349	39548	6.9	Twinfilin-2
606	P22234	20	79	68.7	425	47079	7.2	Multifunctional protein ADE2
607	A8KAP9	20	232	68.4	412	46554	8	Argininosuccinate synthase
607	P00966	20	232	68.4	412	46530	8	Argininosuccinate synthase
608	Q06323	20	169	68.3	249	28723	6	Proteasome activator complex subunit 1
609	Q9BT78	20	51	68.2	406	46269	5.8	COP9 signalosome complex subunit 4
610	P07195	20	325	65.6	334	36639	6.1	L-lactate dehydrogenase B chain
611	O75874	20	76	61.4	414	46659	7	Isocitrate dehydrogenase [NADP] cytoplasmic
612	Q9Y3I0	20	51	56.8	505	55210	7.2	tRNA-splicing ligase RtcB log
613	O00231	20	43	56.6	422	47464	6.5	26S proteasome non-ATPase regulatory subunit 11
614	P11172	20	57	55	480	52222	7.2	Uridine 5'-monophosphate synthase
615	Q16851-2	20	44	56.7	497	55677	7.9	Isoform 2 of UTP--glucose-1-phosphate uridylyltransferase
615	Q16851	20	44	55.5	508	56940	8.1	UTP--glucose-1-phosphate uridylyltransferase
615	C9JKD6	20	44	55.5	508	56967	7.9	Uncharacterized protein

Table S1. Proteins identified in the OVCAR-3 tumor supernatant by two or more unique peptides.

Protein or Protein Group	Accession Number	Unique Peptide Count	Spectrum Count	Sequence Coverage (%)	Length	MolWt	pI	Descriptive Name
615	B4DUP2	20	44	54.5	517	57791	8.1	cDNA FLJ56155, highly similar to UTP--glucose-1-phosphate uridylyltransferase 2 (EC 2.7.7.9)
615	E7EUC7	20	44	54.5	517	57805	8.1	Uncharacterized protein
616	Q16881-5	20	49	59.3	499	54604	6.5	Isoform 5 of Thioredoxin reductase 1, cytoplasmic
616	E2QRB9	20	49	54.7	541	59580	7.3	Uncharacterized protein
616	B7Z2S5	20	49	54.1	547	60022	6.7	cDNA FLJ56075, highly similar to Thioredoxin reductase 1, cytoplasmic (EC 1.8.1.9)
616	Q16881-4	20	49	53.7	551	60269	6.7	Isoform 4 of Thioredoxin reductase 1, cytoplasmic
616	E9PNQ6	20	49	52.1	568	62036	6.7	Uncharacterized protein
616	E7ESI6	20	49	49.7	596	65360	8.1	Uncharacterized protein
616	E7EW10	20	49	48.4	612	67059	6.8	Uncharacterized protein
616	Q16881	20	49	45.6	649	70756	7.4	Thioredoxin reductase 1, cytoplasmic
616	UPI0000D6223A	20	49	45.5	651	71153	7.5	UPI0000D6223A UniRef100 entry
617	D6REN6	20	39	52.8	489	54847	5.5	Uncharacterized protein
617	P12081	20	39	50.7	509	57411	5.9	Histidyl-tRNA synthetase, cytoplasmic
618	B4DTU0	20	89	48.1	570	63665	7.3	cDNA FLJ59249, highly similar to CD166 antigen
618	UPI0000EE206C	20	89	48.1	570	63634	7.7	UPI0000EE206C UniRef100 entry
618	Q13740-2	20	89	48.1	570	63666	7.1	Isoform 2 of CD166 antigen
618	Q13740	20	89	47	583	65102	6.3	CD166 antigen
619	P11413-2	20	30	44	561	63827	6.9	Isoform Long of Glucose-6-phosphate 1-dehydrogenase
620	Q14247	20	179	44	550	61586	5.4	Src substrate cortactin
620	Q53HG7	20	179	44	550	61558	5.4	Cortactin isoform a variant (Fragment)
621	B7Z8X5	20	86	43.7	483	52703	7.9	cDNA FLJ61541, highly similar to sapiens PDZ and LIM domain 5 (PDLIM5), transcript variant 2, mRNA
622	Q01813	20	48	42.1	784	85596	7.6	6-phosphofructokinase type C
623	O76094	20	55	39	671	74606	9.3	Signal recognition particle 72 kDa protein
624	Q16643	20	61	35.3	649	71429	4.5	Drebrin
624	UPI000013E0B5	20	61	35.3	649	71439	4.5	drebrin isoform a
625	Q13617	20	33	35.8	745	86983	6.9	Cullin-2
625	B7Z6K8	20	33	34.9	764	89504	7.1	cDNA FLJ56037, highly similar to Cullin-2
625	UPI0000EE4467	20	33	34.9	764	89492	7.1	cullin-2 isoform a
626	O43747	20	101	33.7	822	91351	6.8	AP-1 complex subunit gamma-1
626	Q8IY97	20	101	33.6	825	91757	6.8	Adaptor-related protein complex 1, gamma 1 subunit
626	O43747-2	20	101	33.6	825	91723	6.8	Isoform 2 of AP-1 complex subunit gamma-1
627	Q12959-5	20	53	35.5	853	95166	6	Isoform 5 of Disks large log 1
627	Q12959-3	20	53	34.8	871	97076	5.9	Isoform 3 of Disks large log 1
627	Q12959	20	53	33.5	904	100455	5.8	Disks large log 1
628	Q14139	20	51	29.8	1066	122560	5.2	Ubiquitin conjugation factor E4 A
628	Q14139-2	20	51	29.6	1073	123522	5.2	Isoform 2 of Ubiquitin conjugation factor E4 A
629	O94985-2	20	42	28.9	971	108643	4.9	Isoform 2 of Calsyntenin-1
629	O94985	20	42	28.6	981	109793	4.9	Calsyntenin-1
630	P28340	20	20	24.9	1107	123631	7	DNA polymerase delta catalytic subunit
630	E7EVW0	20	20	24.4	1133	126388	7.2	DNA polymerase
631	Q9C0E2	20	40	24.2	1151	130139	5	Exportin-4
631	UPI00015DFDED	20	40	24.2	1151	130153	5.1	Exportin-4 (Exp4).
632	B7ZKM9	20	28	20.5	1408	154303	6.4	NEO1 protein
632	Q92859-2	20	28	20.5	1408	154304	6.4	Isoform 2 of Neogenin
632	Q92859	20	28	19.8	1461	160016	6.5	Neogenin
633	Q6YHK3	20	27	17.4	1445	161688	5.8	CD109 antigen
634	Q9P265	20	23	17.3	1576	171491	8.1	Disco-interacting protein 2 log B
635	Q92621	20	36	14	2012	227919	6.2	Nuclear pore complex protein Nup205
636	P58107	20	69	8.3	5090	555629	5.6	Epiplakin
637	P62333	19	104	58.4	389	44173	7.5	26S protease regulatory subunit 10B 26S protease regulatory subunit 10B
637	UPI00001FD556	19	104	56.3	403	45797	7.8	26S protease regulatory subunit 10B
638	Q3U6V5	19	50	63.3	387	43572	5.4	Putative uncharacterized protein
638	Q96CG1	19	50	60.6	404	45463	5.6	ETF1 protein
638	B7Z7P8	19	50	57.9	423	47476	5.6	cDNA FLJ56175, highly similar to Eukaryotic peptide chain release factor subunit1
638	Q3TL10	19	50	56.1	437	48973	5.8	Putative uncharacterized protein
638	Q3TF02	19	50	56.1	437	49030	5.8	Putative uncharacterized protein
638	P62495	19	50	56.1	437	49031	5.7	Eukaryotic peptide chain release factor subunit 1 Eukaryotic peptide chain release factor subunit 1
639	D3Z2H9	19	63	52.8	248	28992	4.8	Uncharacterized protein
639	Q58E70	19	63	52.8	248	29021	4.8	Tpm3 protein
639	E2RB38	19	63	52.8	248	29033	4.8	Uncharacterized protein
640	Q9R1P4	19	65	81.7	263	29547	6.4	Proteasome subunit alpha type-1
641	Q9CVB6	19	73	74	300	34357	7.3	Actin-related protein 2/3 complex subunit 2
642	Q9D1A2	19	32	68	475	52767	5.7	Cytosolic non-specific dipeptidase
643	Q91XF8	19	102	63.5	395	45029	5.5	Apolipoprotein A-IV
643	Q9DBN0	19	102	63.5	395	45044	5.5	Putative uncharacterized protein
644	Q5HZJ8	19	52	53.1	414	46674	7.2	Isocitrate dehydrogenase [NADP]
644	Q3UAV7	19	52	53.1	414	46674	7.2	Isocitrate dehydrogenase [NADP]
644	O88844	19	52	53.1	414	46660	6.9	Isocitrate dehydrogenase [NADP] cytoplasmic
644	E9PWT9	19	52	52.3	421	47474	6.9	Uncharacterized protein
644	Q8C338	19	52	52.1	422	47545	6.9	Isocitrate dehydrogenase [NADP]
645	Q8VCM7	19	178	51.8	436	49391	5.9	Fibrinogen gamma chain
645	Q3UER8	19	178	51	443	50349	5.6	Putative uncharacterized protein
646	E9Q5J9	19	61	46.3	285	33281	4.8	Uncharacterized protein
647	O08528	19	49	26.7	917	102535	6.1	Hexokinase-2
648	Q9Z1Z0-2	19	153	28.1	897	100195	5	Isoform 2 of General vesicular transport factor p115
648	Q9Z1Z0	19	153	26.3	959	106983	4.9	General vesicular transport factor p115
649	P28271	19	48	26.1	889	98179	7.5	Cytoplasmic aconitate hydratase
649	Q3TQ15	19	48	26.1	889	98126	7.5	Aconitase 1
649	Q8VDC3	19	48	25.8	899	99103	7.3	Cytoplasmic aconitase
650	B2RX13	19	33	17.8	1475	165118	7.1	Peroxidasin homolog (Drosophila)
650	Q3UQ28	19	33	17.8	1475	165133	7.1	Peroxidasin homolog
650	E9QNQ9	19	33	17.8	1475	165102	7.1	Uncharacterized protein
651	B1AY13	19	40	10.7	2617	293999	6.2	Ubiquitin carboxyl-terminal hydrolase 24
651	E9PV45	19	40	10.7	2618	294128	6.2	Uncharacterized protein
652	Q99497	19	201	87.3	189	19891	6.8	Protein DJ-1
653	P00918	19	79	83.5	260	29246	7.4	Carbonic anhydrase 2
654	P06132	19	87	81.5	367	40787	6.1	Uroporphyrinogen decarboxylase
655	P04406	19	930	78.5	335	36053	8.5	Glyceraldehyde-3-phosphate dehydrogenase
656	P11766	19	79	75.1	374	39724	7.5	Alcohol dehydrogenase class-3
657	O15144	19	74	74	300	34333	7.3	Actin-related protein 2/3 complex subunit 2
658	Q9UL46	19	117	74.9	239	27402	5.7	Proteasome activator complex subunit 2
658	Q2TNB3	19	117	74.9	239	27349	5.6	Cell migration-inducing protein 22
658	A8MZ76	19	117	71.9	249	28602	6.4	Uncharacterized protein
658	C9JE52	19	117	70.5	254	29127	6.7	Uncharacterized protein
659	P14923	19	170	66.8	745	81745	6.1	Junction plakoglobin
660	P23193	19	77	65.1	301	33970	8.4	Transcription elongation factor A protein 1
661	P23528	19	993	78.3	166	18502	8.1	Cofilin-1
661	E9PK25	19	993	63.7	204	22728	8.3	Uncharacterized protein

Table S1. Proteins identified in the OVCAR-3 tumor supernatant by two or more unique peptides.

Protein or Protein Group	Accession Number	Unique Peptide Count	Spectrum Count	Sequence Coverage (%)	Length	MolWt	pI	Descriptive Name
662	A8K4T6	19	42	58.7	504	56224	5.6	cDNA FLJ76282, highly similar to sapiens proteasome (prosome, macropain) 26S subunit, non-ATPase, 5 (PSMD5), mRNA
662	Q16401	19	42	58.7	504	56196	5.5	26S proteasome non-ATPase regulatory subunit 5
663	P30740	19	52	55.4	379	42742	6.3	Leukocyte elastase inhibitor
664	O00232	19	46	53.1	456	52904	7.6	26S proteasome non-ATPase regulatory subunit 12
665	B7Z4T9	19	51	52.5	499	54804	7.7	Chaperonin containing TCP1, subunit 7 (Eta), isoform CRA_c
665	Q99832	19	51	48.3	543	59367	7.6	T-complex protein 1 subunit eta
665	Q6IBT3	19	51	48.3	543	59329	7.6	CCT7 protein
665	Q53HV2	19	51	48.3	543	59341	7.6	Chaperonin containing TCP1, subunit 7 (Eta) variant (Fragment)
666	P39023	19	82	49.4	403	46109	10.2	60S ribosomal protein L3
667	Q8IXW6	19	33	53.2	570	61979	6.5	Dihydropyrimidinase-like 3
667	Q14195	19	33	53.2	570	61963	6.5	Dihydropyrimidinase-related protein 3
667	B3SXQ8	19	33	44.3	684	73911	6.4	Collapsin response mediator protein 4 long variant
668	Q59EF6	19	56	38.3	729	83109	5.1	Calpain 2, large [catalytic] subunit variant (Fragment)
669	UPI0001AE6739	19	74	38.5	566	63397	8.5	Septin-9 (MLL septin-like fusion protein) (MLL septin-like fusion protein MSF-A) (Ovarian/Breast septin) (Ov/Br septin) (Septin D1).
669	Q9UHD8-7	19	74	38.4	567	63502	8.7	Isoform 7 of Septin-9
669	Q9UHD8-2	19	74	38.4	568	63666	8.4	Isoform 2 of Septin-9
669	Q9UHD8-5	19	74	37.7	579	64682	7.6	Isoform 5 of Septin-9
669	Q9UHD8	19	74	37.2	586	65402	9	Septin-9
670	Q09161	19	44	37	790	91839	6.4	Nuclear cap-binding protein subunit 1
671	P41226	19	31	34.4	1012	111694	6	Ubiquitin-like modifier-activating enzyme 7
672	B1AJV0	19	35	32.8	1005	117265	7.8	SWI/SNF related, matrix associated, actin dependent regulator of chromatin, subfamily a, member 1 (Fragment)
672	E9PCY3	19	35	31.9	1033	120589	8.1	Uncharacterized protein
672	P28370	19	35	31.3	1054	122605	8.1	Probable global transcription activator SNF2L1
672	B7ZLQ5	19	35	30.8	1070	124327	8.4	SMARCA1 protein
673	Q9NZ08	19	31	26.4	941	107235	6.5	Endoplasmic reticulum aminopeptidase 1
673	Q9NZ08-2	19	31	26.2	948	107841	6.3	Isoform 2 of Endoplasmic reticulum aminopeptidase 1
674	Q9NTZ6	19	45	26.1	932	97396	8.6	RNA-binding protein 12
675	Q15042	19	27	25.6	981	110524	5.6	Rab3 GTPase-activating protein catalytic subunit
675	C9J837	19	27	25.4	988	111285	5.5	Uncharacterized protein
676	Q96T76	19	55	25.2	1030	113289	6.4	MMS19 nucleotide excision repair protein log
677	Q13435	19	47	24.2	895	100228	5.7	Splicing factor 3B subunit 2
678	Q9HCE1	19	30	23.6	1003	113671	8.8	Putative helicase MOV-10
679	O15357	19	22	20.2	1258	138599	6.5	Phosphatidylinositol-3,4,5-trisphosphate 5-phosphatase 2
680	P50748	19	24	11.8	2209	250746	6	Kinetochore-associated protein 1
681	O43149	19	29	9.4	2961	331075	5.9	Zinc finger ZZ-type and EF-hand domain-containing protein 1
682	P63244	18	206	84.2	317	35077	7.7	Guanine nucleotide-binding protein subunit beta-2-like 1 Guanine nucleotide-binding protein subunit beta-2-like 1
683	Q921M7	18	38	74.1	324	36776	6.1	Protein FAM49B
683	Q9NUQ9	18	38	74.1	324	36748	6.1	Protein FAM49B
684	Q59HD4	18	534	67.3	373	39525	7.1	Poly(RC)-binding protein 2 isoform b variant (Fragment) Poly(RC)-binding protein 2 isoform b variant (Fragment)
685	Q7L1Q6	18	65	60.6	419	48043	5.9	Basic leucine zipper and W2 domain-containing protein 1 Basic leucine zipper and W2 domain-containing protein 1
685	Q53FN7	18	65	59.9	424	48618	6.1	BZW1 protein variant (Fragment)
685	B4DLZ8	18	65	56.3	451	51282	6.6	cDNA FLJ55789, highly similar to Rattus norvegicus basic leucine zipper and W2 domains 1 (Bzw1), mRNA
686	P61201	18	122	53.7	443	51597	5.5	COP9 signalosome complex subunit 2 COP9 signalosome complex subunit 2
687	D3YVE6	18	99	53.4	266	30038	10.5	MCG18601
687	Q6P1A9	18	99	53.4	266	30025	10.6	Ribosomal protein L7A
687	Q5EBG5	18	99	53.4	266	29905	10.6	Ribosomal protein L7A
687	P62424	18	99	53.4	266	29996	10.6	60S ribosomal protein L7a
687	P12970	18	99	53.4	266	29977	10.6	60S ribosomal protein L7a
687	Q80UT7	18	99	52.6	270	30474	10.6	Rpl7a protein (Fragment)
688	P09651-3	18	578	70.4	267	29386	9.1	Isoform 2 of Heterogeneous nuclear ribonucleoprotein A1
688	Q3U7F3	18	578	58.8	320	34238	9.2	Putative uncharacterized protein
688	P49312	18	578	58.8	320	34196	9.2	Heterogeneous nuclear ribonucleoprotein A1 Heterogeneous nuclear ribonucleoprotein A1
688	P09651	18	578	50.5	372	38747	9.1	Heterogeneous nuclear ribonucleoprotein A1
688	E1BKB5	18	578	50.4	373	38804	9.1	Uncharacterized protein
688	Q5EBP8	18	578	50.4	373	38834	9.1	Heterogeneous nuclear ribonucleoprotein A1
689	P35235	18	35	40.4	597	68460	7.3	Tyrosine-protein phosphatase non-receptor type 11
689	Q06124	18	35	40.4	597	68436	7.3	Tyrosine-protein phosphatase non-receptor type 11
690	Q92841	18	97	32.3	650	72372	8.6	Probable ATP-dependent RNA helicase DDX17
690	Q501J6	18	97	32.3	650	72400	8.6	Probable ATP-dependent RNA helicase DDX17
690	Q3U741	18	97	32.2	652	72585	8.7	DEAD (Asp-Glu-Ala-Asp) box polypeptide 17, isoform CRA_a
690	Q92841-3	18	97	32.2	652	72557	8.7	Isoform 3 of Probable ATP-dependent RNA helicase DDX17
690	UPI00003670EA	18	97	28.8	729	80255	8.3	UPI00003670EA UniRef100 entry
690	Q92841-4	18	97	28.8	729	80273	8.3	Isoform 4 of Probable ATP-dependent RNA helicase DDX17
690	UPI0001AE634C	18	97	28.7	731	80440	8.4	UPI0001AE634C UniRef100 entry
690	UPI0001533DB4	18	97	28.7	731	80458	8.4	probable ATP-dependent RNA helicase DDX17 isoform 3
690	Q59F66	18	97	28.5	737	81068	7.9	DEAD box polypeptide 17 isoform p82 variant (Fragment)
691	E7EVJ5	18	50	17.6	1227	142577	7.2	Uncharacterized protein
691	E7EVF4	18	50	17.2	1253	145673	7.1	Uncharacterized protein
691	Q96F07-2	18	50	17.2	1253	145673	7.1	Isoform 2 of Cytoplasmic FMR1-interacting protein 2
691	Q5SQX6	18	50	17.2	1253	145659	7.1	Cytoplasmic FMR1-interacting protein 2
692	B7STB7	18	51	56.6	346	38750	7.4	Annexin A1
692	Q4FK88	18	51	56.6	346	38734	7.7	Anxa1 protein
692	P10107	18	51	56.6	346	38734	7.4	Annexin A1
693	Q3UKP2	18	129	51.7	460	51318	7.8	Hemopexin, isoform CRA_f
693	Q91X72	18	129	51.7	460	51341	7.8	Hemopexin
694	Q99JI4	18	39	48.6	389	45536	5.5	26S proteasome non-ATPase regulatory subunit 6
695	Q19LI2	18	58	47.9	512	56554	6.8	Alpha-1B-glycoprotein
696	P80315	18	34	47.5	539	58066	8	T-complex protein 1 subunit delta
696	Q3TII0	18	34	47.5	539	58084	8	T-complex protein 1 subunit delta
697	Q61696	18	306	47.1	641	70079	5.7	Heat shock 70 kDa protein 1A
697	Q3TAI8	18	306	47.1	641	70093	5.7	Putative uncharacterized protein
697	P17879	18	306	47	642	70176	5.7	Heat shock 70 kDa protein 1B
698	Q9D8E6	18	103	43.4	419	47154	11	60S ribosomal protein L4
699	P14733	18	59	36.9	588	66786	5.2	Lamin-B1
700	Q3V3W7	18	47	35.5	732	83206	6	Putative uncharacterized protein
700	Q8BH61	18	47	35.5	732	83207	5.9	Coagulation factor XIII A chain
701	P19221	18	31	35.1	618	70269	6.4	Prothrombin
702	P46460	18	43	28.6	744	82613	6.9	Vesicle-fusing ATPase
703	A7ISP9	18	55	25.6	977	108710	6.4	EMAP-like protein 4
703	E9QML0	18	55	25.3	988	109972	6.5	Uncharacterized protein
704	E9QLI1	18	46	21.8	1172	129896	4.8	Uncharacterized protein
704	Q8CG21	18	46	21.8	1172	129882	4.8	Thbs2
704	Q03350	18	46	21.8	1172	129912	4.8	Thrombospondin-2

Protein or Protein Group	Accession Number	Unique Peptide Count	Spectrum Count	Sequence Coverage (%)	Length	MolWt	pI	Descriptive Name
705	P11087-2	18	46	21.6	1225	117821	5.7	Isoform 2 of Collagen alpha-1(I) chain
705	E9QKB9	18	46	21.5	1226	117918	5.7	Uncharacterized protein
705	P11087	18	46	18.2	1453	138033	5.8	Collagen alpha-1(I) chain
706	B9EKK3	18	20	16.5	1575	180550	5.6	IQ motif containing GTPase activating protein 2
706	Q3UQ44	18	20	16.5	1575	180527	5.6	Ras GTPase-activating-like protein IQGAP2
707	Q8BL66	18	25	15.2	1411	160914	5.8	Early endosome antigen 1
708	A2ABW7	18	45	6.2	3718	404056	6.7	Laminin, alpha 5
708	Q61001	18	45	6.2	3718	404013	6.7	Laminin subunit alpha-5
709	P11802	18	38	81.5	303	33730	7	Cyclin-dependent kinase 4
710	P00491	18	112	79.2	289	32118	6.9	Purine nucleoside phosphorylase
710	Q8N7G1	18	112	78.2	293	32551	7.2	cDNA FLJ25678 fis, clone TST04067, highly similar to PURINE NUCLEOSIDE PHOSPHORYLASE (EC 2.4.2.1)
711	Q14914	18	74	75.4	329	35870	8.3	Prostaglandin reductase 1
712	P23919	18	35	74.5	212	23819	8.3	Thymidylate kinase
713	Q9Y696	18	74	70.8	253	28772	5.6	Chloride intracellular channel protein 4
714	Q15366	18	261	69.9	365	38580	6.8	Poly(rC)-binding protein 2
715	Q7L2H7	18	81	69.8	374	42503	5.6	Eukaryotic translation initiation factor 3 subunit M
716	P14550	18	75	69.5	325	36573	6.8	Alcohol dehydrogenase [NADP+]
717	Q9UNS2	18	40	69	423	47873	6.7	COP9 signalosome complex subunit 3
718	Q9Y3F4	18	76	70.6	350	38438	5.1	Serine-threonine kinase receptor-associated protein
718	B4DNJ6	18	76	68	363	39778	5.3	cDNA FLJ51909, highly similar to Serine-threonine kinase receptor-associatedprotein
719	P17174	18	48	65.9	413	46248	7	Aspartate aminotransferase, cytoplasmic
720	P53582	18	46	65.8	386	43215	7.2	Methionine aminopeptidase 1
721	Q53GN6	18	43	65.7	376	42917	5.7	Proteasome 26S non-ATPase subunit 13 isoform 1 variant (Fragment)
721	Q9UNM6	18	43	65.7	376	42946	5.8	26S proteasome non-ATPase regulatory subunit 13
722	Q53FE8	18	60	65.1	370	40572	5.1	cDNA FLJ36526 fis, clone TRACH2003347, highly similar to NSFL1 cofactor p47 (Fragment)
723	B2RBP3	18	52	64.8	463	51822	5.5	cDNA, FLJ95615, highly similar to sapiens ubiquitin-activating enzyme E1C (UBA3 log, yeast)(UBE1C), mRNA
723	Q8TBC4	18	52	64.8	463	51852	5.5	NEDD8-activating enzyme E1 catalytic subunit
724	Q9Y266	18	48	61.6	331	38243	5.4	Nuclear migration protein nudC
725	Q9NZL9	18	36	59.6	334	37552	7.4	Methionine adenosyltransferase 2 subunit beta
726	P35237	18	51	58.8	376	42622	5.3	Serpin B6
726	UPI00004155CD	18	51	54	409	46402	5.8	Serpin B6 (Placental thrombin inhibitor) (Cytoplasmic antiproteinase) (CAP) (Proteinase inhibitor 6) (PI-6).
727	Q5TFE4	18	44	58.5	455	51845	6.3	5'-nucleotidase domain-containing protein 1
728	Q6P1N9	18	46	56.9	297	33602	7	Putative deoxyribonuclease TATDN1
729	P35813	18	26	56.5	382	42448	5.4	Protein phosphatase 1A
729	UPI0000E01530	18	26	47.5	455	51366	7.1	protein phosphatase 1A isoform 3
730	Q3LXA3	18	35	54.1	575	58947	7.5	Bifunctional ATP-dependent dihydroxyacetone kinase/FAD-AMP lyase (cyclizing)
731	P41091	18	43	53.4	472	51110	8.4	Eukaryotic translation initiation factor 2 subunit 3
732	E7ETZ4	18	48	53.9	408	46913	8.4	Uncharacterized protein
732	B3KM68	18	48	52.5	419	48190	6.7	cDNA FLJ10398 fis, clone NT2RM4000349, highly similar to sapiens basic leucine zipper and W2 domains 2 (BZW2), mRNA
732	Q9Y6E2	18	48	52.5	419	48162	6.7	Basic leucine zipper and W2 domain-containing protein 2
733	P21281	18	41	52.1	511	56501	5.8	V-type proton ATPase subunit B, brain isoform
734	D9YZV3	18	52	51.4	284	32737	4.8	Tropomyosin 1 (Alpha) isoform 3
735	A8K7D9	18	41	50.9	529	57834	5.4	cDNA FLJ78270, highly similar to sapiens karyopherin alpha 2 (RAG cohort 1, importin alpha 1)(KPNA2), mRNA
735	Q6NVW7	18	41	50.9	529	57965	5.4	Karyopherin alpha 2 (RAG cohort 1, importin alpha 1)
735	P52292	18	41	50.9	529	57862	5.4	Importin subunit alpha-2
736	Q12765	18	50	52.7	414	46382	4.8	Secernin-1
736	B4DHM0	18	50	50.2	434	48843	4.8	cDNA FLJ59687, highly similar to Secernin-1
737	E7EWF1	18	125	46.6	406	45499	11	Uncharacterized protein
737	P36578	18	125	44.3	427	47697	11.1	60S ribosomal protein L4
737	Q59GY2	18	125	42.9	441	48996	11	Ribosomal protein L4 variant (Fragment)
738	Q8N1G4	18	79	45.3	583	63473	8.3	Leucine-rich repeat-containing protein 47
739	O95394	18	46	46.1	542	59852	6.3	Phosphoacetylglucosamine mutase
739	B4DX94	18	46	43.9	570	62911	6.1	cDNA FLJ55543, highly similar to Phosphoacetylglucosamine mutase (EC 5.4.2.3)
740	O43252	18	74	43.6	624	70833	6.9	Bifunctional 3'-phosphoadenosine 5'-phosphosulfate synthase 1
741	O43776	18	87	42.3	548	62943	6.3	Asparaginyl-tRNA synthetase, cytoplasmic
742	Q9ULV4	18	50	45.1	474	53249	7.1	Coronin-1C
742	Q53G58	18	50	45.1	474	53281	7.1	Coronin, actin binding protein, 1C variant (Fragment)
742	Q59EA2	18	50	42.7	501	56370	8.2	Coronin, actin binding protein, 1C variant (Fragment)
742	A7MAP1	18	50	40.6	527	58948	7.8	Coronin-1C_i3 protein
743	B4DRA5	18	36	39.8	742	83810	7.1	cDNA FLJ61346, highly similar to Protein transport protein Sec23B
743	Q15437	18	36	38.5	767	86479	6.9	Protein transport protein Sec23B
744	Q9UHB9	18	82	39.1	627	70730	8.6	Signal recognition particle 68 kDa protein
745	Q03169	18	55	36.9	654	72661	6.5	Tumor necrosis factor alpha-induced protein 2
746	E9PG40	18	57	31.5	714	80833	4.8	Uncharacterized protein
746	P05067-7	18	57	30.7	733	82916	4.8	Isoform L-APP733 of Amyloid beta A4 protein
746	B4DII8	18	57	30.2	746	84521	4.8	cDNA FLJ50531, highly similar to Amyloid beta A4 protein (APP) (ABPP)(Alzheimer disease amyloid protein log)
746	P05067-8	18	57	30	751	84819	4.8	Isoform APP751 of Amyloid beta A4 protein
746	P05067-9	18	57	29.9	752	85040	4.8	Isoform L-APP752 of Amyloid beta A4 protein
746	P05067	18	57	29.2	770	86943	4.8	Amyloid beta A4 protein
747	Q9Y6K5	18	23	29.3	1087	121170	8.4	2'-5'-oligoadenylate synthase 3
748	E9PL22	18	31	29.6	937	104779	5.7	Uncharacterized protein
748	B7Z2N4	18	31	28.2	981	109705	6.1	cDNA FLJ56074, highly similar to 150 kDa oxygen-regulated protein (Orp150)
748	Q9Y4L1	18	31	27.7	999	111335	5.2	Hypoxia up-regulated protein 1
749	P29692	18	157	63.3	281	31122	5	Elongation factor 1-delta
749	UPI000179584C	18	157	27.6	646	71266	6.4	UPI000179584C UniRef100 entry
749	B2RAR6	18	157	27.5	647	71450	6.4	cDNA, FLJ95068, highly similar to sapiens eukaryotic translation elongation factor 1 delta (guanine nucleotide exchange protein) (EEF1D), transcript variant 1, mRNA
749	P29692-2	18	157	27.5	647	71408	6.4	Isoform 2 of Elongation factor 1-delta
749	D3DWK1	18	157	27.5	647	71422	6.4	Eukaryotic translation elongation factor 1 delta (Guanine nucleotide exchange protein), isoform CRA_b
749	E9PRY8	18	157	25.5	697	76570	7	Uncharacterized protein
750	Q6PKG0	18	97	23.2	1096	123510	8.8	La-related protein 1
751	Q13045	18	18	17.5	1269	144751	6.1	Protein flightless-1 log
752	P55196-2	18	58	16.5	1612	181998	6.5	Isoform 1 of Afadin
752	C9JX92	18	58	15.3	1743	197637	6.5	Uncharacterized protein
752	P55196-3	18	58	15.3	1743	197652	6.5	Isoform 3 of Afadin
752	P55196-1	18	58	14.6	1816	205604	6.8	Isoform 2 of Afadin
753	Q59EZ3	18	21	9.7	2414	266112	5.8	Insulin-like growth factor 2 receptor variant (Fragment)
753	E7ENI8	18	21	9.4	2491	274387	5.9	Uncharacterized protein

Table S1. Proteins identified in the OVCAR-3 tumor supernatant by two or more unique peptides.

Protein or Protein Group	Accession Number	Unique Peptide Count	Spectrum Count	Sequence Coverage (%)	Length	MolWt	pI	Descriptive Name
753	P11717	18	21	9.4	2491	274373	5.9	Cation-independent mannose-6-phosphate receptor
754	P60510	17	80	79.5	307	35080	5.1	Serine/threonine-protein phosphatase 4 catalytic subunit Serine/threonine-protein phosphatase 4 catalytic subunit
755	Q16576	17	53	74.8	425	47820	5	Histone-binding protein RBBP7
755	Q60973	17	53	74.8	425	47790	5	Histone-binding protein RBBP7
755	Q3UJI2	17	53	74.8	425	47776	5	Putative uncharacterized protein
756	Q5R507	17	92	63.6	272	30629	6	F-actin-capping protein subunit beta F-actin-capping protein subunit beta
756	B2R7T8	17	92	63.6	272	30657	6	cDNA, FLJ93598, highly similar to sapiens capping protein (actin filament) muscle Z-line, beta (CAPZB), mRNA
756	A2AMV7	17	92	57.5	301	33767	6.4	CAPZB3
756	B1AK88	17	92	57.5	301	33781	6.4	Capping protein (Actin filament) muscle Z-line, beta
756	E7EQ40	17	92	51.8	334	37455	6.1	Uncharacterized protein
757	P38919	17	38	57.2	411	46871	6.7	Eukaryotic initiation factor 4A-III
757	Q91VC3	17	38	57.2	411	46840	6.7	Eukaryotic initiation factor 4A-III
758	O35737	17	191	51.2	449	49199	6.3	Heterogeneous nuclear ribonucleoprotein H
758	P31943	17	191	51.2	449	49229	6.3	Heterogeneous nuclear ribonucleoprotein H
758	UPI00001AF4DD	17	191	48.7	472	51230	6.8	Heterogeneous nuclear ribonucleoprotein H (hnRNP H).
758	Q8C2Q7	17	191	48.7	472	51218	6.8	Heterogeneous nuclear ribonucleoprotein H1
759	E7EU96	17	51	50.9	385	45311	7.9	Uncharacterized protein
759	Q8R0X4	17	51	50.1	391	45134	7.7	Casein kinase 2, alpha 1 polypeptide
759	Q61177	17	51	50.1	391	45180	7.7	Casein kinase II alpha subunit
759	P68400	17	51	50.1	391	45144	7.7	Casein kinase II subunit alpha
759	E7EN85	17	51	49.6	395	45682	7.9	Uncharacterized protein
759	Q5U5J2	17	51	49.4	397	45909	7.9	CSNK2A1 protein
760	P60228	17	84	45.8	445	52221	6	Eukaryotic translation initiation factor 3 subunit E Eukaryotic translation initiation factor 3 subunit E
760	Q6IAX5	17	84	45.8	445	52206	6	EIF3S6 protein
760	Q3T102	17	84	45.8	445	52191	6	Eukaryotic translation initiation factor 3 subunit E
761	Q7TQI3	17	105	85.2	271	31270	4.9	Ubiquitin thioesterase OTUB1
762	P37804	17	106	77.1	201	22576	8.8	Transgelin
763	Q9CPV4	17	27	76.8	298	33317	5.5	Glyoxalase domain-containing protein 4
764	P18760	17	649	76.5	166	18560	8.1	Cofilin-1
765	E9PZF0	17	412	60.3	267	30200	8.7	Uncharacterized protein
766	D3Z6C3	17	164	56.8	264	29825	9.7	Uncharacterized protein
766	Q3UAC2	17	164	56.8	264	29913	9.7	Putative uncharacterized protein
766	P97351	17	164	56.8	264	29885	9.7	40S ribosomal protein S3a
767	P16125	17	244	55.4	334	36572	6.1	L-lactate dehydrogenase B chain
768	Q3TJK3	17	143	51.8	417	46535	8.5	Putative uncharacterized protein
768	Q5U4D0	17	143	51.8	417	46534	8.8	Serpinh1 protein
768	Q3TWG9	17	143	51.8	417	46530	8.8	Putative uncharacterized protein
769	Q3UJK2	17	218	49.7	392	42914	8.4	Putative uncharacterized protein
769	Q9CY58-2	17	218	49.7	392	42944	8.4	Isoform 2 of Plasminogen activator inhibitor 1 RNA-binding protein
769	Q9CY58	17	218	47.9	407	44714	8.5	Plasminogen activator inhibitor 1 RNA-binding protein
770	Q00896	17	168	48.5	412	45823	5.4	Alpha-1-antitrypsin 1-3
771	P45376	17	42	46.5	316	35732	7.2	Aldose reductase
771	UPI000060684B	17	42	46.5	316	35745	7.5	UPI000060684B UniRef100 entry
771	Q5U415	17	42	46.5	316	35746	7.2	Aldo-keto reductase family 1, member B3 (Aldose reductase)
772	Q6P1B1	17	52	44.6	623	69591	5.5	Xaa-Pro aminopeptidase 1
773	P27659	17	64	43.4	403	46124	10.2	60S ribosomal protein L3
773	Q91VJ6	17	64	43.4	403	46110	10.2	MCG11520, isoform CRA_b
773	Q3UB90	17	64	43.4	403	46080	10.2	Putative uncharacterized protein
773	Q3UB67	17	64	43.4	403	46168	10.2	Putative uncharacterized protein
773	Q3UB15	17	64	43.4	403	46124	10.2	Putative uncharacterized protein
774	Q08879	17	59	34.5	705	78033	5.2	Fibulin-1
775	B2RWX8	17	44	32.2	603	67261	7.5	Complement component factor i
775	Q61129	17	44	32.2	603	67235	7.4	Complement factor I
776	Q8CC78	17	97	29.2	706	79736	6	Putative uncharacterized protein
776	Q3TCV6	17	97	27.5	749	84758	6.1	Putative uncharacterized protein
776	Q8R1F1	17	97	27.5	749	84819	5.9	Niban-like protein 1
776	Q8BQ71	17	97	27.5	749	84849	5.9	Putative uncharacterized protein
776	Q3UDW4	17	97	27.5	749	84765	5.9	Putative uncharacterized protein
776	Q3UC84	17	97	27.5	749	84803	6	Putative uncharacterized protein
776	Q3U8I3	17	97	27.5	749	84761	6	Putative uncharacterized protein
777	A2A432	17	44	21.6	970	110699	8.4	Cullin-4B
778	UPI0000023CC5	17	19	10.3	2327	252305	5.4	chondroitin sulfate proteoglycan 4 precursor
779	E9Q7G0	17	43	8.8	2094	235628	5.9	Uncharacterized protein
779	Q80Y35	17	43	8.8	2094	235656	5.9	Nuclear mitotic apparatus protein 1
780	P27348	17	150	86.1	245	27764	4.8	14-3-3 protein theta
781	Q96FW1	17	111	85.2	271	31284	4.9	Ubiquitin thioesterase OTUB1
781	UPI0001AE6C7D	17	111	75	308	35215	5.6	UPI0001AE6C7D UniRef100 entry
782	P10768	17	58	85.1	282	31463	7	S-formylglutathione hydrolase
783	Q13347	17	43	75.7	325	36502	5.6	Eukaryotic translation initiation factor 3 subunit I
784	O94760	17	44	75.4	285	31122	5.8	N(G),N(G)-dimethylarginine dimethylaminohydrolase 1
785	Q15691	17	137	74.3	268	29999	5.1	Microtubule-associated protein RP/EB family member 1
786	Q13126	17	72	73.9	283	31236	7.2	S-methyl-5'-thioadenosine phosphorylase
787	P52943	17	154	70.7	208	22493	8.7	Cysteine-rich protein 2
788	O95433	17	75	69.8	338	38274	5.5	Activator of 90 kDa heat shock protein ATPase log 1
789	Q5JQ10	17	50	72.2	345	38703	6.7	Adenosine kinase
789	P55263	17	50	68.8	362	40545	6.7	Adenosine kinase
790	Q9UBE0	17	67	66.8	346	38450	5.3	SUMO-activating enzyme subunit 1
791	Q86UA3	17	53	62.8	376	42508	6.8	Chromosome 12 open reading frame 10
792	Q9HB71	17	99	62.7	228	26210	8.2	Calcyclin-binding protein
793	Q07960	17	41	59.9	439	50436	6.3	Rho GTPase-activating protein 1
794	P00390-2	17	41	61.2	479	51701	7.7	Isoform Cytoplasmic of Glutathione reductase, mitochondrial
794	C8KIL8	17	41	59.4	493	53027	8.8	Glutathion reductase delta8 alternative splicing variant
794	P00390	17	41	56.1	522	56257	8.5	Glutathione reductase, mitochondrial
795	P61246	17	176	57.7	260	29586	9.7	40S ribosomal protein S3a (Fragment)
795	P61247	17	176	56.8	264	29945	9.7	40S ribosomal protein S3a
796	O96019	17	31	57.3	429	47461	5.6	Actin-like protein 6A
796	Q53FS0	17	31	57.3	429	47481	5.7	Actin-like 6A isoform 1 variant (Fragment)
797	P43034	17	36	57.3	410	46638	7.4	Platelet-activating factor acetylhydrolase IB subunit alpha
797	UPI0001AE6665	17	36	57.3	410	46448	7.5	Platelet-activating factor acetylhydrolase IB subunit alpha (PAF acetylhydrolase 45 kDa subunit) (PAF-AH 45 kDa subunit) (PAF-AH alpha) (PAFAH alpha) (Lissencephaly-1 protein) (LIS-1).
798	P23381	17	41	55.2	471	53165	6.2	Tryptophanyl-tRNA synthetase, cytoplasmic
799	Q9BUB1	17	45	56.8	382	43067	5.1	PRKAR2A protein
799	A8KAH7	17	45	53.7	404	45504	5.1	cDNA FLJ75444, highly similar to sapiens protein kinase, cAMP-dependent, regulatory, type II, alpha (PRKAR2A), mRNA
799	P13861	17	45	53.7	404	45518	5.1	cAMP-dependent protein kinase type II-alpha regulatory subunit
800	A6NFN4	17	25	53.4	528	59420	5.2	Uncharacterized protein
800	Q13564	17	25	52.8	534	60246	5.4	NEDD8-activating enzyme E1 regulatory subunit
801	Q9ULA0	17	34	55.2	475	52428	7.4	Aspartyl aminopeptidase

Table S1. Proteins identified in the OVCAR-3 tumor supernatant by two or more unique peptides.

Protein or Protein Group	Accession Number	Unique Peptide Count	Spectrum Count	Sequence Coverage (%)	Length	MolWt	pI	Descriptive Name
801	E7ETB3	17	34	53.1	493	54531	7.7	Uncharacterized protein
802	P30566	17	39	53.1	484	54889	7.1	Adenylosuccinate lyase
802	Q71UA4	17	39	53.1	484	54871	7.1	Adenylosuccinate lyase
803	P37837	17	95	52.5	337	37540	6.8	Transaldolase
804	Q9UJU6	17	64	50.2	430	48207	5	Drebrin-like protein
805	Q53GI5	17	37	48.7	423	48109	7.9	Adaptor-related protein complex 1, mu 2 subunit variant (Fragment)
805	Q9Y6Q5	17	37	48.7	423	48109	8.2	AP-1 complex subunit mu-2
806	D6RGI3	17	35	49.4	425	49006	7	Septin 11, isoform CRA_b
806	Q9NVA2	17	35	49	429	49398	6.8	Septin-11
806	D6RER5	17	35	48.6	432	49809	6.7	Uncharacterized protein
806	Q9NVA2-2	17	35	47.8	439	50823	7	Isoform 2 of Septin-11
807	O00148	17	115	46.1	427	49130	5.7	ATP-dependent RNA helicase DDX39A
808	P43490	17	34	45.6	491	55521	7.2	Nicotinamide phosphoribosyltransferase
809	B2RDD7	17	38	44.6	637	72710	6.3	cDNA, FLJ96564, highly similar to sapiens SKB1 log (S. pombe) (SKB1), mRNA
809	O14744	17	38	44.6	637	72684	6.3	Protein arginine N-methyltransferase 5
810	P12955	17	39	44.6	493	54548	6	Xaa-Pro dipeptidase
811	P32455	17	41	42.7	592	67931	6.3	Interferon-induced guanylate-binding protein 1
812	Q14103-3	17	320	48	306	32835	8.2	Isoform 3 of Heterogeneous nuclear ribonucleoprotein D0
812	Q14103	17	320	41.4	355	38434	7.8	Heterogeneous nuclear ribonucleoprotein D0
813	Q9HC38-2	17	34	68.8	298	33233	5.6	Isoform 2 of Glyoxalase domain-containing protein 4
813	E7EMY5	17	34	40.9	501	54690	8.7	Uncharacterized protein
813	Q9Y3E8	17	34	40.7	504	55012	8.7	Uncharacterized protein C17orf25
814	Q8TAT6	17	69	39.8	608	68120	6.4	Nuclear protein localization protein 4 log
814	E7EVD8	17	69	39.3	616	69360	6.7	Uncharacterized protein
814	Q8TAT6-2	17	69	39.2	617	69461	6.7	Isoform 2 of Nuclear protein localization protein 4 log
815	B4E3E8	17	90	33.9	646	71355	6.6	cDNA FLJ60399, highly similar to ATP-dependent RNA helicase DDX3X (EC 3.6.1.-)
815	UPI000013ED8C	17	90	33.1	661	73156	7.2	ATP-dependent RNA helicase DDX3X isoform 2
815	O00571	17	90	33.1	662	73244	7.2	ATP-dependent RNA helicase DDX3X
815	B5BTY4	17	90	33.1	662	73172	7.4	ATP-dependent RNA helicase DDX3X
816	Q14134-2	17	90	33.9	570	63844	7	Isoform Beta of Tripartite motif-containing protein 29
816	Q14134	17	90	32.8	588	65835	7.1	Tripartite motif-containing protein 29
816	Q53G41	17	90	32.8	588	65817	7	Tripartite motif protein TRIM29 isoform alpha variant (Fragment)
817	Q8IV36-2	17	32	29.9	787	88616	6.1	Isoform 2 of UPF0663 transmembrane protein C17orf28
817	Q8IV36	17	32	29.8	788	88745	6.1	UPF0663 transmembrane protein C17orf28
818	P52888	17	39	28.6	689	78840	6	Thimet oligopeptidase
818	Q96CV8	17	39	28.6	689	78824	6	Thimet oligopeptidase 1
819	A8K6F7	17	62	27.2	929	101675	6.1	Unc-45 log A (C. elegans), isoform CRA_a
819	Q9H3U1	17	62	26.8	944	103077	6.1	Protein unc-45 log A
820	Q8TD19	17	26	25.7	979	107168	5.7	Serine/threonine-protein kinase Nek9
821	Q9BXP5-5	17	78	25.5	839	96223	5.8	Isoform 5 of Serrate RNA effector molecule log
821	Q9BXP5-4	17	78	24.6	871	100147	6	Isoform 4 of Serrate RNA effector molecule log
821	Q9BXP5-3	17	78	24.5	875	100537	6	Isoform 3 of Serrate RNA effector molecule log
821	Q9BXP5-2	17	78	24.5	872	100276	6	Isoform 2 of Serrate RNA effector molecule log
821	Q9BXP5	17	78	24.4	876	100667	6	Serrate RNA effector molecule log
822	Q13620-1	17	44	23.7	895	102299	7.9	Isoform 2 of Cullin-4B
822	E0CX08	17	44	23.6	900	102756	8	Cullin 4B, isoform CRA_e
822	Q13620	17	44	23.2	913	103982	7.4	Cullin-4B
823	Q9UKU6	17	30	21.3	1024	116999	7	Thyrotropin-releasing hormone-degrading ectoenzyme
824	B2R8Y6	17	47	21.8	1162	134062	5.3	cDNA, FLJ94121, highly similar to sapiens stromal antigen 2 (STAG2), mRNA
824	Q8N3U4	17	47	20.6	1231	141326	5.4	Cohesin subunit SA-2
824	Q6MZM3	17	47	20.6	1231	141301	5.4	Putative uncharacterized protein DKFZp686C21148
824	B1AMT5	17	47	20	1268	145751	5.5	Stromal antigen 2
824	Q6MZP3	17	47	20	1268	145675	5.5	Putative uncharacterized protein DKFZp686I05169
824	Q68DE9	17	47	20	1268	145781	5.5	Putative uncharacterized protein DKFZp781H1753
825	D3DSM4	17	69	17.7	1336	135510	6.5	Collagen, type XVIII, alpha 1, isoform CRA_d
825	P39060-2	17	69	17.7	1339	135761	6.5	Isoform 3 of Collagen alpha-1(XVIII) chain
825	P39060-1	17	69	15.6	1519	154018	5.7	Isoform 2 of Collagen alpha-1(XVIII) chain
825	D3DSM5	17	69	15.6	1516	153767	5.7	Collagen, type XVIII, alpha 1, isoform CRA_b
825	UPI00018147FD	17	69	13.5	1751	177935	6	collagen alpha-1(XVIII) chain isoform 3 precursor
825	P39060	17	69	13.5	1754	178187	6	Collagen alpha-1(XVIII) chain
826	D3DX70	17	24	13.3	1583	184657	5.1	GRIP and coiled-coil domain containing 2, isoform CRA_a
826	UPI00015E0A4F	17	24	13.3	1583	184653	5.1	Ran-binding protein 2-like 4 (RanBP2L4).
826	Q8IWJ2	17	24	12.5	1684	195908	5.1	GRIP and coiled-coil domain-containing protein 2
827	E1P559	17	30	11.8	1816	201823	6.3	Laminin, alpha 4, isoform CRA_b
827	Q5D044	17	30	11.8	1816	201882	6.3	LAMA4 protein
828	P61106	16	222	85.1	215	23897	6.2	Ras-related protein Rab-14 Ras-related protein Rab-14
829	P51149	16	356	77.3	207	23490	6.7	Ras-related protein Rab-7a Ras-related protein Rab-7a
830	P32233	16	30	59.4	367	40512	8.9	Developmentally-regulated GTP-binding protein 1
830	Q9Y295	16	30	59.4	367	40542	8.9	Developmentally-regulated GTP-binding protein 1
831	P61160	16	82	53.6	394	44761	6.7	Actin-related protein 2 Actin-related protein 2
832	Q06138	16	30	49.9	341	39842	7.2	Calcium-binding protein 39
832	Q9Y376	16	30	49.9	341	39869	6.9	Calcium-binding protein 39
832	Q8VDZ8	16	30	49.9	341	39843	6.9	Cab39 protein
833	Q5T7C4	16	99	65.8	158	18311	9.7	High-mobility group box 1 High-mobility group box 1
833	Q59GW1	16	99	59.1	176	20164	9.7	High-mobility group box 1 variant (Fragment)
833	Q8BNM0	16	99	57.5	181	20648	9.8	Putative uncharacterized protein (Fragment)
833	UPI0000608C59	16	99	48.8	213	24606	6.8	PREDICTED: high mobility group protein B1-like
833	P09429	16	99	48.4	215	24894	5.7	High mobility group protein B1
833	Q497Z6	16	99	48.4	215	24908	5.7	High mobility group box 1
833	P63158	16	99	48.4	215	24894	5.7	High mobility group protein B1
834	Q3TWV4	16	25	45.5	433	49389	9.5	Adaptor protein complex AP-2, mu1, isoform CRA_a Adaptor protein complex AP-2, mu1, isoform CRA_a
834	Q96CW1	16	25	45.3	435	49655	9.5	AP-2 complex subunit mu AP-2 complex subunit mu
834	Q3TH69	16	25	45.3	435	49727	9.5	Putative uncharacterized protein
834	B4DNB9	16	25	42.8	460	52338	9.5	cDNA FLJ53069, highly similar to AP-2 complex subunit mu-1
834	E9PFW3	16	25	42.8	460	52304	9.5	Uncharacterized protein
835	Q6P1J9	16	61	37.1	531	60577	9.6	Parafibromin Parafibromin
836	A1A563	16	29	22.8	860	97304	9.2	Eukaryotic translation initiation factor 2C, 2
836	Q9UKV8	16	29	22.8	859	97208	9.2	Protein argonaute-2
836	Q8CJG0	16	29	22.8	860	97323	9.2	Protein argonaute-2
837	B2R8Y4	16	344	16.2	901	103252	5.6	cDNA, FLJ94117, highly similar to sapiens actinin, alpha 3 (ACTN3), mRNA
837	Q08043	16	344	16.2	901	103241	5.5	Alpha-actinin-3
837	O88990	16	344	16.2	900	103043	5.4	Alpha-actinin-3
838	P68254	16	138	86.1	245	27778	4.8	14-3-3 protein theta
839	Q9WVA4	16	368	81.4	199	22395	8.2	Transgelin-2
840	D3Z0Y2	16	102	74.5	200	22494	8.9	Uncharacterized protein
841	Q91VH6	16	34	73.7	297	33692	7.2	Protein MEMO1
842	UPI0000473750	16	79	72.7	231	26159	5.2	proteasome activator complex subunit 2 isoform 2
842	P97372	16	79	70.3	239	27057	5.7	Proteasome activator complex subunit 2

Table S1. Proteins identified in the OVCAR-3 tumor supernatant by two or more unique peptides.

Protein or Protein Group	Accession Number	Unique Peptide Count	Spectrum Count	Sequence Coverage (%)	Length	MolWt	pI	Descriptive Name
843	P62908	16	86	68.3	243	26674	9.7	40S ribosomal protein S3
844	Q9QYB1	16	66	67.6	253	28729	5.6	Chloride intracellular channel protein 4
845	Q64010	16	56	61.2	304	33815	5.6	Adapter molecule crk
846	Q8BTX5	16	34	60.2	352	39846	6.7	Putative uncharacterized protein
846	Q91WK2	16	34	60.2	352	39832	6.7	Eukaryotic translation initiation factor 3 subunit H
847	P97371	16	115	57.8	249	28673	6	Proteasome activator complex subunit 1
848	Q3T9L0	16	112	45.2	427	49085	5.7	Putative uncharacterized protein
848	Q8VDW0	16	112	45.2	427	49067	5.7	ATP-dependent RNA helicase DDX39A
848	Q3UVN0	16	112	45.2	427	49083	5.7	Putative uncharacterized protein
849	P06745	16	43	41.2	558	62767	8.1	Glucose-6-phosphate isomerase
850	Q3UXJ2	16	40	35.9	651	70913	7	Putative uncharacterized protein
850	A1L354	16	40	34.3	683	74615	7.1	Transforming growth factor, beta induced
850	P82198	16	40	34.3	683	74597	7.1	Transforming growth factor-beta-induced protein ig-h3
851	UPI0001889CC6	16	106	25.5	552	61392	7.4	UPI0001889CC6 UniRef100 entry
852	P09405	16	161	24.3	707	76723	4.8	Nucleolin
852	Q8CD23	16	161	24.3	707	76866	4.8	Putative uncharacterized protein
852	Q3TL52	16	161	24.3	707	76751	4.8	Putative uncharacterized protein
853	Q04857	16	58	23.3	1025	108489	5.4	Collagen alpha-1(VI) chain
854	Q8TDQ7	16	71	85.5	276	31085	7	Glucosamine-6-phosphate isomerase 2
855	P04792	16	174	84.9	205	22783	6.4	Heat shock protein beta-1
856	P37802	16	458	84.4	199	22391	8.2	Transgelin-2
857	Q14232	16	38	78.4	305	33712	7.3	Translation initiation factor eIF-2B subunit alpha
858	A8K237	16	36	77.9	276	31491	8.1	cDNA FLJ75913, highly similar to sapiens cytochrome b5 reductase 2 (CYB5R2), transcript variant 1, mRNA
859	Q13630	16	47	77.9	321	35893	6.6	GDP-L-fucose synthase
860	Q9Y316	16	34	76.8	297	33733	7.2	Protein MEMO1
861	Q9NQR4	16	54	72.1	276	30608	7.2	Omega-amidase NIT2
862	P06493	16	50	71.7	297	34081	8.4	Cyclin-dependent kinase 1
863	Q15257	16	57	69.8	358	40668	5.9	Serine/threonine-protein phosphatase 2A activator
864	Q00796	16	56	68.9	357	38325	8	Sorbitol dehydrogenase
865	P23396	16	89	68.3	243	26688	9.7	40S ribosomal protein S3
866	P48739	16	48	68.6	271	31540	6.9	Phosphatidylinositol transfer protein beta isoform
866	B7Z7Q0	16	48	68.1	273	31700	6.7	Phosphatidylinositol transfer protein, beta, isoform CRA_a
867	P21266	16	36	68	225	26560	5.5	Glutathione S-transferase Mu 3
868	P30041	16	369	67.4	224	25035	6.4	Peroxiredoxin-6
869	Q15417	16	174	63.8	329	36414	6.1	Calponin-3
870	P67809	16	149	63.3	324	35924	9.9	Nuclease-sensitive element-binding protein 1
871	P40925	16	60	62.3	334	36426	7.3	Malate dehydrogenase, cytoplasmic
871	UPI0001E92A3D	16	60	59.1	352	38628	7.7	malate dehydrogenase, cytoplasmic isoform 1
872	P46108	16	57	61.2	304	33831	5.6	Adapter molecule crk
873	Q9H2U2	16	60	60.5	334	37920	7.4	Inorganic pyrophosphatase 2, mitochondrial
873	Q9H2U2-2	16	60	57.9	349	39638	6.9	Isoform 2 of Inorganic pyrophosphatase 2, mitochondrial
874	P12277	16	55	60.4	381	42644	5.6	Creatine kinase B-type
875	O15372	16	50	60.2	352	39930	6.5	Eukaryotic translation initiation factor 3 subunit H
875	Q53HR0	16	50	60.2	352	39914	6.5	Eukaryotic translation initiation factor 3, subunit 3 gamma, 40kDa variant (Fragment)
876	UPI0001B1A4B8	16	76	60	422	45804	5.4	perilipin-3 isoform 3
877	Q02750	16	33	59.8	393	43439	6.6	Dual specificity mitogen-activated protein kinase kinase 1
878	P34949	16	32	57.9	423	46656	6	Mannose-6-phosphate isomerase
879	O75436	16	32	57.2	327	38170	6.5	Vacuolar protein sorting-associated protein 26A
880	Q13561	16	44	57.9	401	44231	5.2	Dynactin subunit 2
880	Q13561-3	16	44	57.6	403	44473	5.2	Isoform 3 of Dynactin subunit 2
880	B2RBK5	16	44	57.1	406	44820	5.2	cDNA, FLJ95559, sapiens dynactin 2 (p50) (DCTN2), mRNA
881	P12429	16	56	56	323	36375	5.9	Annexin A3
882	P39748	16	58	55	380	42593	8.6	Flap endonuclease 1
883	P27707	16	42	54.2	260	30519	5.2	Deoxycytidine kinase
884	O15264	16	19	53.7	365	42090	8.4	Mitogen-activated protein kinase 13
885	P39687	16	105	52.6	249	28585	4.1	Acidic leucine-rich nuclear phosphoprotein 32 family member A
886	P26599	16	227	51.4	531	57221	9.2	Polypyrimidine tract-binding protein 1
886	P26599-2	16	227	49.6	550	59037	9.2	Isoform 2 of Polypyrimidine tract-binding protein 1
886	Q9BUQ0	16	227	49	557	59633	9.2	Polypyrimidine tract binding protein 1
887	Q9BZK7	16	50	47.5	514	55595	5.6	F-box-like/WD repeat-containing protein TBL1XR1
888	P54727	16	50	47.2	409	43171	4.8	UV excision repair protein RAD23 log B
889	P30520	16	42	46.5	456	50097	6.6	Adenylosuccinate synthetase isozyme 2
890	A1JUI8	16	36	45.5	488	53681	6.1	Chaperonin subunit 6A (Fragment)
891	Q9BR76	16	40	44.6	489	54235	5.9	Coronin-1B
892	P55010	16	42	44.5	431	49223	5.6	Eukaryotic translation initiation factor 5
893	P04040	16	21	43.8	527	59756	7.4	Catalase
894	P49189	16	24	43.5	494	53802	5.9	4-trimethylaminobutyraldehyde dehydrogenase
894	B9EKV4	16	24	41.5	518	56292	6.6	Aldehyde dehydrogenase 9 family, member A1
895	A8K525	16	201	41.2	471	54288	9.2	cDNA FLJ76817, highly similar to sapiens non-POU domain containing, octamer-binding (NONO), mRNA
895	Q15233	16	201	41.2	471	54232	8.9	Non-POU domain-containing octamer-binding protein
896	B3KML1	16	56	41	519	58472	5.1	cDNA FLJ11308 fis, clone PLACE1010074, highly similar to Sorting nexin-2
896	O60749	16	56	41	519	58471	5.1	Sorting nexin-2
897	B3KT11	16	18	41	503	57593	7.1	cDNA FLJ37430 fis, clone BRAWH2001679, highly similar to sapiens NMD3 log (NMD3), mRNA
897	Q96D46	16	18	41	503	57603	7.1	60S ribosomal export protein NMD3
898	P35611-2	16	34	40.9	631	69985	6.4	Isoform 2 of Alpha-adducin
898	E7EV99	16	34	40.8	632	70056	6.4	Uncharacterized protein
898	A2A3N8	16	34	39	662	73333	6.5	Adducin 1 (Alpha)
898	E7ENY0	16	34	38.9	663	73404	6.5	Uncharacterized protein
898	P35611	16	34	35	737	80955	5.8	Alpha-adducin
898	P35611-3	16	34	33.6	768	84303	6	Isoform 3 of Alpha-adducin
899	Q15833-2	16	32	39	590	66138	6.5	Isoform 2 of Syntaxin-binding protein 2
899	Q53GF4	16	32	38.8	593	66367	6.6	Syntaxin binding protein 2 variant (Fragment)
899	Q15833	16	32	38.8	593	66453	6.5	Syntaxin-binding protein 2
899	E7EQD5	16	32	38.1	604	67695	6.7	Uncharacterized protein
900	Q8NBF2	16	30	37.5	726	79444	5.6	NHL repeat-containing protein 2
901	P10155	16	30	36.6	538	60671	8	60 kDa SS-A/Ro ribonucleoprotein
902	Q14258	16	69	35.7	630	70974	8.1	E3 ubiquitin/ISG15 ligase TRIM25
903	Q96G03	16	49	35.1	612	68283	6.7	Phosphoglucomutase-2
904	P40222	16	21	35	546	61891	6.5	Alpha-taxilin
905	UPI00015DFEB1	16	39	34.9	648	73424	8	ATP-dependent DNA helicase Q1 (EC 3.6.1.-) (DNA-dependent ATPase Q1).
905	P46063	16	39	34.8	649	73457	7.9	ATP-dependent DNA helicase Q1
906	P46060	16	41	34.4	587	63542	4.7	Ran GTPase-activating protein 1
907	B3GQS7	16	41	32.7	569	60680	6	Mitochondrial heat shock 60kD protein 1 variant 1
907	P10809	16	41	32.5	573	61055	5.9	60 kDa heat shock protein, mitochondrial
908	Q96AC1	16	26	32.9	680	77861	6.7	Fermitin family log 2
908	B5TJY2	16	26	32.6	687	78676	6.7	KINDLIN2 isoform 2
909	Q9BR63	16	34	30.3	585	65701	6.8	FARSB protein (Fragment)
909	Q9NSD9	16	34	30.1	589	66116	6.8	Phenylalanyl-tRNA synthetase beta chain

Table S1. Proteins identified in the OVCAR-3 tumor supernatant by two or more unique peptides.

Protein or Protein Group	Accession Number	Unique Peptide Count	Spectrum Count	Sequence Coverage (%)	Length	MolWt	pI	Descriptive Name
910	Q14764	16	27	29.9	893	99327	5.5	Major vault protein
911	Q32MZ4-3	16	21	28.3	752	82689	4.6	Isoform 3 of Leucine-rich repeat flightless-interacting protein 1
911	Q32MZ4-2	16	21	27.2	784	86404	4.7	Isoform 2 of Leucine-rich repeat flightless-interacting protein 1
911	Q32MZ4	16	21	26.4	808	89253	4.7	Leucine-rich repeat flightless-interacting protein 1
912	P13591-4	16	61	29.3	726	80312	4.9	Isoform 4 of Neural cell adhesion molecule 1
912	P13591-1	16	61	25.1	848	93361	4.9	Isoform 2 of Neural cell adhesion molecule 1
913	A0AV37	16	25	24.9	837	92756	9.3	Plakophilin 2
913	Q99959	16	25	23.6	881	97415	9.3	Plakophilin-2
913	B8QGS6	16	25	23.6	881	97401	9.3	Plakophilin-2
913	B8QFA1	16	25	23.6	881	97388	9.3	Plakophilin-2 transcript variant 2b
914	UPI00004120FF	16	18	24.6	1053	112966	6.3	serine/threonine-protein phosphatase 6 regulatory ankyrin repeat subunit A isoform a
915	UPI0000470C59	16	30	22	1077	113456	5.7	large proline-rich protein BAT3 isoform d
915	B0UX83	16	30	21	1126	118703	5.6	HLA-B associated transcript 3
915	P46379-2	16	30	21	1126	118693	5.6	Isoform 2 of Large proline-rich protein BAG6
915	P46379	16	30	20.9	1132	119409	5.6	Large proline-rich protein BAG6
915	UPI0000EE4D04	16	30	20.4	1162	122352	5.7	UPI0000EE4D04 UniRef100 entry
915	P46379-3	16	30	20.4	1162	122342	5.7	Isoform 3 of Large proline-rich protein BAG6
916	B2RAH5	16	91	19.4	1030	115329	5.4	cDNA, FLJ94919, highly similar to sapiens protein phosphatase 1, regulatory (inhibitor) subunit 12A (PPP1R12A), mRNA
916	O14974	16	91	19.4	1030	115281	5.4	Protein phosphatase 1 regulatory subunit 12A
917	Q9BQS8	16	29	14.4	1478	166982	4.9	FYVE and coiled-coil domain-containing protein 1
917	B7ZKT7	16	29	14.2	1498	168887	4.9	Putative uncharacterized protein
918	B6ZDQ0	16	25	13.4	1880	211307	6.1	Uncharacterized protein
918	Q6YHU6	16	25	12.9	1953	219605	6.1	Thyroid adenoma-associated protein
918	C9JER1	16	25	12.8	1954	219734	6.1	Uncharacterized protein
919	Q8N3C0	16	36	13.2	2202	251458	7.1	Activating signal cointegrator 1 complex subunit 3
920	C9JK41	16	28	12.5	1812	201249	5.8	Uncharacterized protein
920	UPI00015E02B1	16	28	12.4	1826	202901	5.9	Kinesin-like protein KIF13B (Kinesin-like protein GAKIN).
921	B1A8Z4	16	37	12.3	1613	181260	8	SMARCA4 isoform 3
921	B1A8Z7	16	37	12.3	1614	181347	8	SMARCA4 isoform 1
921	B1A8Z6	16	37	12.3	1616	181602	8.1	SMARCA4 isoform 4
921	B1A8Z5	16	37	12.3	1617	181689	8.1	SMARCA4 isoform 2
921	P51532	16	37	12.1	1647	184644	7.9	Transcription activator BRG1
921	Q9HBD4	16	37	11.9	1679	188147	8.2	SMARCA4 isoform 2
921	B9EGQ8	16	37	11.8	1681	189447	8.2	SMARCA4 protein
922	Q99996	16	24	4.7	3911	453670	5	A-kinase anchor protein 9
922	UPI0001AE70FE	16	24	4.7	3861	447766	5	UPI0001AE70FE UniRef100 entry
922	UPI00003674DA	16	24	4.7	3842	446011	5	A-kinase anchor protein 9 (Protein kinase A-anchoring protein 9) (PRKA9) (A-kinase anchor protein 450 kDa) (AKAP 450) (A-kinase anchor protein 350 kDa) (AKAP 350) (hgAKAP 350) (AKAP 120-like protein) (Protein hyperion) (Protein yotiao) (Centrosome- and Go
922	UPI000013D66F	16	24	4.7	3896	451926	5	A-kinase anchor protein 9 (Protein kinase A-anchoring protein 9) (PRKA9) (A-kinase anchor protein 450 kDa) (AKAP 450) (A-kinase anchor protein 350 kDa) (AKAP 350) (hgAKAP 350) (AKAP 120-like protein) (Protein hyperion) (Protein yotiao) (Centrosome- and Go
922	Q99996-5	16	24	4.7	3857	447755	5	Isoform 5 of A-kinase anchor protein 9
922	Q99996-3	16	24	4.7	3899	452113	5	Isoform 3 of A-kinase anchor protein 9
922	Q99996-2	16	24	4.7	3907	452990	5	Isoform 2 of A-kinase anchor protein 9
922	Q99996-6	16	24	4.6	3929	455426	5	Isoform 6 of A-kinase anchor protein 9
923	P61981	15	202	82.2	247	28303	4.9	14-3-3 protein gamma 14-3-3 protein gamma
924	P62714	15	86	80.3	309	35575	5.4	Serine/threonine-protein phosphatase 2A catalytic subunit beta isoform Serine/threonine-protein phosphatase 2A catalytic subunit beta isoform
924	Q8WZ56	15	86	80.3	309	35631	5.4	Serine/threonine-protein phosphatase
924	Q5U0I7	15	86	80.3	309	35605	5.4	Serine/threonine-protein phosphatase
925	B9ZVU1	15	285	47	281	30865	8.8	Uncharacterized protein
925	Q12771	15	285	46.2	286	31481	7.4	P37 AUF1
925	UPI0000435439	15	285	46	287	30608	8.4	heterogeneous nuclear ribonucleoprotein D0 isoform d
925	Q14103-4	15	285	46	287	30672	8.4	Isoform 4 of Heterogeneous nuclear ribonucleoprotein D0
925	B4DTC3	15	285	43.6	303	33612	8.5	cDNA FLJ54150, highly similar to Heterogeneous nuclear ribonucleoprotein D0
925	UPI000043543A	15	285	39.3	336	36208	8.1	heterogeneous nuclear ribonucleoprotein D0 isoform b
925	Q14103-2	15	285	39.3	336	36272	8.1	Isoform 2 of Heterogeneous nuclear ribonucleoprotein D0
926	A8K885	15	40	36.9	406	46648	6.3	cDNA FLJ77179, highly similar to sapiens sorting nexin 6 (SNX6) mRNA
926	Q9UNH7	15	40	36.9	406	46649	6.2	Sorting nexin-6
926	Q9CZ03	15	40	36.9	406	46649	6.2	Putative uncharacterized protein
926	Q6P8X1	15	40	36.9	406	46635	6	Sorting nexin-6
927	P14576	15	22	36.7	504	55721	8.8	Signal recognition particle 54 kDa protein
927	P61011	15	22	36.7	504	55705	8.8	Signal recognition particle 54 kDa protein
928	B1AQE8	15	38	7.2	2820	317227	7.3	Neurofibromatosis 1
928	P21359-2	15	38	7.2	2818	317032	7.3	Isoform 1 of Neurofibromin
928	Q04690	15	38	7.1	2841	319595	7.4	Neurofibromin
928	P21359	15	38	7.1	2839	319372	7.4	Neurofibromin
929	P16015	15	68	83.8	260	29366	7.4	Carbonic anhydrase 3
930	P35700	15	78	66.3	199	22176	8.1	Peroxiredoxin-1
931	Q9JII6	15	48	62.8	325	36587	7.4	Alcohol dehydrogenase [NADP+]
932	E9PX42	15	239	58	333	35757	8	Uncharacterized protein
932	P16858	15	239	58	333	35810	8.2	Glyceraldehyde-3-phosphate dehydrogenase
933	Q9DAW9	15	136	57.6	330	36429	5.7	Calponin-3
934	P08226	15	116	55	311	35867	5.7	Apolipoprotein E
934	Q6GTX3	15	116	55	311	35853	5.7	Apoe protein
934	Q4FK40	15	116	55	311	35848	5.7	Apoe protein
934	Q3UBS0	15	116	55	311	35907	5.7	Putative uncharacterized protein
935	Q3U3L3	15	28	47.4	378	42569	5.7	Putative uncharacterized protein
935	Q60854	15	28	47.4	378	42599	5.7	Serpin B6
936	P35585	15	26	46.3	423	48543	7.3	AP-1 complex subunit mu-1
937	Q3TEU8	15	38	44.7	474	53108	6.8	Putative uncharacterized protein
937	Q9WUM4	15	38	44.7	474	53121	7.1	Coronin-1C
937	Q5PPQ7	15	38	44.7	474	53093	7.1	Coronin, actin binding protein 1C
938	D3YTY9	15	66	42.9	480	53205	5.1	Uncharacterized protein
939	P80313	15	33	36.9	544	59652	7.8	T-complex protein 1 subunit eta
939	Q8C5Q5	15	33	36.9	544	59636	7.8	Putative uncharacterized protein
939	Q3TJN2	15	33	36.9	544	59638	7.8	Putative uncharacterized protein
939	Q3TET0	15	33	36.9	544	59684	7.8	Putative uncharacterized protein
940	Q8CAA7	15	49	25.9	621	70280	6.5	Glucose 1,6-bisphosphate synthase
941	UPI00004296E7	15	53	21.5	745	84940	4.9	heterogeneous nuclear ribonucleoprotein U-like protein 2
942	Q8BWZ3	15	21	19.7	972	111707	6.5	N-alpha-acetyltransferase 25, NatB auxiliary subunit
943	P51432	15	23	18.2	1234	139492	6	1-phosphatidylinositol-4,5-bisphosphate phosphodiesterase beta-3

Table S1. Proteins identified in the OVCAR-3 tumor supernatant by two or more unique peptides.

Protein or Protein Group	Accession Number	Unique Peptide Count	Spectrum Count	Sequence Coverage (%)	Length	MolWt	pI	Descriptive Name
943	Q8CI86	15	23	18.2	1234	139499	5.9	Phospholipase C, beta 3
943	Q3UIS0	15	23	18.2	1234	139487	5.9	Phospholipase C, beta 3, isoform CRA_a
943	Q3TWI4	15	23	18.2	1234	139501	6	Putative uncharacterized protein
944	E9Q6U9	15	55	17.9	1271	119605	9.1	Uncharacterized protein
944	Q01149	15	55	16.5	1372	129557	9.2	Collagen alpha-2(I) chain
945	Q3TW36	15	23	16.2	1244	137555	6.3	Putative uncharacterized protein
945	Q8CDP6	15	23	16.2	1244	137508	6.3	Putative uncharacterized protein
945	Q6NZR5	15	23	16.2	1244	137524	6.3	Superkiller viralicidic activity 2-like (S. cerevisiae)
946	B2RY15	15	43	16	2542	271644	5.6	Tln2 protein
946	E9PUM4	15	43	16	2542	271665	5.6	Uncharacterized protein
947	A8K486	15	681	86.1	165	18013	6.9	Peptidyl-prolyl cis-trans isomerase
947	P62937	15	681	86.1	165	18012	7.8	Peptidyl-prolyl cis-trans isomerase A
948	P12004	15	89	82.4	261	28769	4.7	Proliferating cell nuclear antigen
948	Q6FHF5	15	89	82.4	261	28706	4.7	Proliferating cell nuclear antigen (Fragment)
949	O95865	15	44	80.7	285	29644	6	N(G),N(G)-dimethylarginine dimethylaminohydrolase 2
950	P30711	15	48	77.1	240	27335	7.5	Glutathione S-transferase theta-1
951	O43809	15	49	73.1	227	26227	8.8	Cleavage and polyadenylation specificity factor subunit 5
952	Q9Y5P6	15	28	72.2	360	39834	6.6	Mannose-1-phosphate guanyltransferase beta
952	Q9Y5P6-2	15	28	67.2	387	42622	6.3	Isoform 2 of Mannose-1-phosphate guanyltransferase beta
953	Q5RLJ0	15	62	70.9	244	27969	6.4	CLE
953	Q9Y224	15	62	70.9	244	28068	6.7	UPF0568 protein C14orf166
954	P60900	15	116	69.1	246	27399	6.7	Proteasome subunit alpha type-6
955	P62979	15	364	68.6	156	17965	9.6	Ubiquitin-40S ribosomal protein S27a
955	Q5RKT7	15	364	68.6	156	17905	9.6	Ribosomal protein S27a
956	P16152	15	92	67.1	277	30375	8.3	Carbonyl reductase [NADPH] 1
957	Q06830	15	122	65.8	199	22110	8.1	Peroxiredoxin-1
958	Q99598	15	59	64.8	290	33112	6.6	Translin-associated protein X
959	P54920	15	17	64.7	295	33233	5.4	Alpha-soluble NSF attachment protein
960	P52788	15	27	63.4	366	41268	5	Spermine synthase
961	Q99426	15	43	62.7	244	27326	5.1	Tubulin-folding cofactor B
962	Q12792	15	111	61.4	350	40283	7	Twinfilin-1
962	UPI00015DFEA8	15	111	58.3	369	42365	7.2	Twinfilin-1 (Protein A6) (Protein tyrosine kinase 9).
963	Q15019	15	55	59.3	361	41487	6.6	Septin-2
963	Q15019-2	15	55	54	396	45461	6.9	Isoform 2 of Septin-2
964	P46109	15	48	59.1	303	33777	6.7	Crk-like protein
965	P20042	15	37	58.9	333	38388	5.8	Eukaryotic translation initiation factor 2 subunit 2
966	Q9HAB8	15	31	52.4	311	34005	6.7	Phosphopantothenate--cysteine ligase
967	O00629	15	77	49.1	521	57887	5	Importin subunit alpha-4
968	Q03154	15	32	59.6	408	45885	6.2	Aminoacylase-1
968	B4DNW0	15	32	48.8	498	55995	7	cDNA FLJ60317, highly similar to Aminoacylase-1 (EC 3.5.1.14)
969	O14745	15	73	46.9	358	38868	5.8	Na(+)/H(+) exchange regulatory cofactor NHE-RF1
970	Q6XQN6	15	38	46.7	538	57578	5.7	Nicotinate phosphoribosyltransferase
971	Q13098-5	15	31	45.8	487	55093	6.6	Isoform 4 of COP9 signalosome complex subunit 1
971	Q13098	15	31	45.4	491	55537	6.8	COP9 signalosome complex subunit 1
971	Q13098-7	15	31	42.3	527	59050	6.3	Isoform 2 of COP9 signalosome complex subunit 1
972	P42167	15	134	44.9	454	50670	9.4	Lamina-associated polypeptide 2, isoforms beta/gamma
973	Q9UMS4	15	34	44.8	504	55181	6.6	Pre-mRNA-processing factor 19
974	Q8IWB7	15	21	42.4	410	46324	7.3	WD repeat and FYVE domain-containing protein 1
975	Q5R644	15	50	41.7	504	56770	5.9	Apoptosis inhibitor 5
975	Q9BZZ5-4	15	50	41.7	504	56733	5.9	Isoform 4 of Apoptosis inhibitor 5
976	Q53FH8	15	51	41.6	404	46816	7.1	Sorting nexin 5 variant (Fragment)
976	Q9Y5X3	15	51	41.6	404	46817	6.8	Sorting nexin-5
976	Q6P5V6	15	51	40.8	412	47438	7.5	SNX5 protein (Fragment)
977	P04424	15	31	40.5	464	51658	6.5	Argininosuccinate lyase
978	Q13409-3	15	41	40.8	612	68426	5.3	Isoform 2C of Cytoplasmic dynein 1 intermediate chain 2
978	Q13409-2	15	41	39.6	632	70645	5.2	Isoform 2B of Cytoplasmic dynein 1 intermediate chain 2
979	UPI000198CA76	15	46	42.1	470	51071	8.2	UPI000198CA76 UniRef100 entry
979	P12268	15	46	38.5	514	55805	6.9	Inosine-5'-monophosphate dehydrogenase 2
980	P38159	15	139	38.1	391	42332	10.1	Heterogeneous nuclear ribonucleoprotein G
981	P50579	15	28	37.9	478	52892	5.8	Methionine aminopeptidase 2
982	P52294	15	46	37.5	538	60222	5	Importin subunit alpha-1
982	Q5BKZ2	15	46	37.5	538	60309	5	Karyopherin alpha 1 (Importin alpha 5)
983	B7ZKK7	15	29	36.4	546	61511	8.3	EIF2AK2 protein
983	P19525	15	29	36.1	551	62094	8.4	Interferon-induced, double-stranded RNA-activated protein kinase
984	B3KTA3	15	26	36	472	52277	7.6	cDNA FLJ37935 fis, clone CTONG2005290, highly similar to FASCIN
984	Q16658	15	26	34.5	493	54530	7.2	Fascin
984	Q96IH1	15	26	34	500	55136	7.2	FSCN1 protein (Fragment)
985	Q96KG9-3	15	24	38.3	707	78696	8.4	Isoform 3 of N-terminal kinase-like protein
985	E9PS17	15	24	35	775	85719	5.7	Uncharacterized protein
985	E9PK59	15	24	34.7	780	86183	6.6	Uncharacterized protein
985	Q96KG9-4	15	24	34.7	781	86312	6.6	Isoform 4 of N-terminal kinase-like protein
985	Q96KG9-2	15	24	34.3	791	88089	6.3	Isoform 2 of N-terminal kinase-like protein
985	Q96KG9	15	24	33.5	808	89631	6.3	N-terminal kinase-like protein
986	B4DKV7	15	42	34.3	659	73315	6	cDNA FLJ60287, highly similar to Epidermal growth factor receptor kinase substrate 8-like protein 1
986	Q8TE68	15	42	31.3	723	80251	6	Epidermal growth factor receptor kinase substrate 8-like protein 1
987	P10909	15	334	31.6	449	52495	6.3	Clusterin
987	P10909-2	15	334	28.3	501	57833	6.7	Isoform 2 of Clusterin
988	E9PFM7	15	84	30.4	710	80291	8.1	Uncharacterized protein
988	Q8IXQ6-2	15	84	26.4	819	92272	7.6	Isoform 2 of Poly [ADP-ribose] polymerase 9
989	E7ETU9	15	25	29.4	703	81170	6.6	Uncharacterized protein
989	O00469	15	25	28.1	737	84686	6.7	Procollagen-lysine,2-oxoglutarate 5-dioxygenase 2
990	Q14677	15	85	28.8	625	68259	6.4	Clathrin interactor 1
991	Q9Y446	15	37	26.2	797	87082	9.3	Plakophilin-3
992	P43243	15	88	26	847	94623	6.3	Matrin-3
992	A8MXP9	15	88	24.6	895	99967	6	Uncharacterized protein
993	Q96FS1	15	67	25.1	830	92388	7.2	CTNND1 protein (Fragment)
993	O60716-5	15	67	22.3	933	104156	7	Isoform 1A of Catenin delta-1
993	E9PN49	15	67	22.2	938	104848	6.9	Uncharacterized protein
993	Q68DU0	15	67	22.2	938	104818	6.9	Putative uncharacterized protein DKFZp781O2021
993	O60716-3	15	67	22.2	939	104977	6.9	Isoform 1AC of Catenin delta-1
993	O60716-2	15	67	21.6	962	107349	6.3	Isoform 1AB of Catenin delta-1
993	O60716	15	67	21.5	968	108170	6.2	Catenin delta-1
994	O15294	15	17	21	1046	116924	6.7	UDP-N-acetylglucosamine--peptide N-acetylglucosaminyltransferase 110 kDa subunit
995	Q9BPX3	15	34	19.7	1015	114334	5.6	Condensin complex subunit 3
996	A8K586	15	15	17.5	1094	121290	6	cDNA FLJ76822, highly similar to sapiens adaptor-related protein complex 3, beta 1 subunit (AP3B1), mRNA
996	O00203	15	15	17.5	1094	121320	6	AP-3 complex subunit beta-1
997	Q9BQG0	15	23	15.3	1328	148854	9.3	Myb-binding protein 1A
997	Q9BQG0-2	15	23	15.2	1332	149366	9.3	Isoform 2 of Myb-binding protein 1A

Table S1. Proteins identified in the OVCAR-3 tumor supernatant by two or more unique peptides.

Protein or Protein Group	Accession Number	Unique Peptide Count	Spectrum Count	Sequence Coverage (%)	Length	MolWt	pI	Descriptive Name
998	B2R6U9	15	28	15	1218	133859	6.1	cDNA, FLJ93126, highly similar to sapiens jagged 1 (Alagille syndrome) (JAG1), mRNA
998	P78504	15	28	15	1218	133799	6.1	Protein jagged-1
999	Q8IUD2-2	15	16	16.8	992	113920	6.3	Isoform 2 of ELKS/Rab6-interacting/CAST family member 1
999	Q8IUD2-3	15	16	15.3	1088	124904	6.1	Isoform 3 of ELKS/Rab6-interacting/CAST family member 1
999	Q8IUD2	15	16	15	1116	128086	6	ELKS/Rab6-interacting/CAST family member 1
1000	Q86WJ2	15	38	14.4	1440	162272	5.9	Mutant receptor type protein tyrosine phosphatase K
1000	Q68DT8	15	38	14.4	1440	162130	5.9	Putative uncharacterized protein DKFZp686C2268
1000	Q15262-2	15	38	14.4	1440	162173	5.9	Isoform 2 of Receptor-type tyrosine-protein phosphatase kappa
1000	B2RTQ8	15	38	14.3	1446	163020	5.9	PTPRK protein
1000	Q5TG12	15	38	14.2	1458	164202	5.8	Protein tyrosine phosphatase, receptor type, K
1000	B7ZMG0	15	38	14.2	1462	164612	5.8	PTPRK protein
1000	E9PGC5	15	38	14.1	1472	166028	5.9	Uncharacterized protein
1001	UPI0001AE6D3D	15	26	13.7	1836	200240	6.7	Ankyrin-3 (ANK-3) (Ankyrin-G).
1001	UPI00002288BB	15	26	13.5	1861	202591	6.7	ankyrin-3 isoform 3
1001	Q7Z3G4	15	26	13.5	1861	202591	6.7	Putative uncharacterized protein DKFZp686P17114
1001	B4DIL1	15	26	13.4	1868	204228	6.9	cDNA FLJ58990, highly similar to Ankyrin-3
1001	E9PE32	15	26	13.4	1868	204141	6.9	Uncharacterized protein
1001	Q12955	15	26	5.7	4377	480416	6.5	Ankyrin-3
1002	A8K9U1	15	21	10.8	1698	191158	6	cDNA FLJ76468, highly similar to sapiens cullin 7 (CUL7), mRNA
1002	B4DYZ0	15	21	10.3	1782	199776	6	cDNA FLJ56181, highly similar to Cullin-7
1003	P49792	15	36	6.6	3224	358201	6.2	E3 SUMO-protein ligase RanBP2
1004	Q8IVF2	15	79	3.4	5795	616641	5.4	Protein AHNAK2
1005	P62987	14	674	66.4	128	14728	9.8	Ubiquitin-60S ribosomal protein L40 Ubiquitin-60S ribosomal protein L40
1006	C9J9K3	14	149	63.6	264	29506	5.2	Uncharacterized protein
1006	B2CY77	14	149	56.9	295	32850	4.9	Laminin receptor (Fragment)
1006	P14206	14	149	56.9	295	32838	4.9	40S ribosomal protein SA
1006	P08865	14	149	56.9	295	32854	4.9	40S ribosomal protein SA
1006	D3YTT7	14	149	56.9	295	32838	4.9	MCG2650
1007	Q9CSU0	14	69	55.8	326	36884	6	Regulation of nuclear pre-mRNA domain-containing protein 1B
1007	Q9NQG5	14	69	55.8	326	36900	6	Regulation of nuclear pre-mRNA domain-containing protein 1B
1008	O35864	14	33	55.4	334	37549	6.5	COP9 signalosome complex subunit 5
1008	Q92905	14	33	55.4	334	37579	6.5	COP9 signalosome complex subunit 5
1009	B4DM97	14	44	53	334	38275	7	cDNA FLJ55002, highly similar to Alpha-centractin
1009	E9PGF2	14	44	52.8	335	38372	7	Uncharacterized protein
1009	P61163	14	44	47.1	376	42614	6.6	Alpha-centractin Alpha-centractin
1010	P46781	14	87	52.1	194	22591	10.7	40S ribosomal protein S9 40S ribosomal protein S9
1011	Q96IR1	14	172	53.1	243	27260	9.9	RPS4X protein (Fragment) RPS4X protein (Fragment)
1011	P62701	14	172	49	263	29598	10.2	40S ribosomal protein S4, X isoform 40S ribosomal protein S4, X isoform
1011	Q53HV1	14	172	49	263	29579	10.1	Ribosomal protein S4, X-linked X isoform variant (Fragment)
1011	Q3UXQ6	14	172	49	263	29564	10.2	Putative uncharacterized protein
1012	Q2TAY7	14	38	42.5	513	57544	7.2	WD40 repeat-containing protein SMU1 WD40 repeat-containing protein SMU1
1013	B4DII5	14	39	29.5	533	59641	5	cDNA FLJ53998, highly similar to Importin alpha-7 subunit
1013	O60684	14	39	29.3	536	60030	5	Importin subunit alpha-7
1013	O35345	14	39	29.3	536	59964	4.9	Importin subunit alpha-7
1013	B4DWX3	14	39	29	541	60580	5	cDNA FLJ53229, highly similar to Importin alpha-7 subunit
1014	Q05DR5	14	38	30.6	633	72784	6.7	Ssrp1 protein (Fragment)
1014	Q08945	14	38	27.4	709	81075	6.9	FACT complex subunit SSRP1
1014	Q08943	14	38	27.4	708	80860	6.8	FACT complex subunit SSRP1
1014	E9QK81	14	38	27.2	713	81377	6.8	Uncharacterized protein
1014	Q08943-2	14	38	27.2	713	81475	7	Isoform 2 of FACT complex subunit SSRP1
1015	A8DUK4	14	405	89.8	147	15748	7.7	Beta-globin
1016	D3ZCX0	14	42	74.7	277	31461	8.6	RCG55799, isoform CRA_a
1017	Q3UAJ1	14	487	74.4	164	17943	7.9	Peptidyl-prolyl cis-trans isomerase
1018	P15626	14	34	61.5	218	25717	7.4	Glutathione S-transferase Mu 2
1019	Q3TN31	14	46	60.1	248	27908	8.8	Proteasome subunit alpha type
1019	Q9Z2U0	14	46	60.1	248	27855	8.5	Proteasome subunit alpha type-7
1020	P42208	14	55	59	361	41526	6.5	Septin-2
1021	O70400	14	94	58.7	327	35774	6.8	PDZ and LIM domain protein 1
1022	Q62348	14	47	57.9	228	26201	6.4	Translin
1023	P14152	14	39	56.3	334	36511	6.6	Malate dehydrogenase, cytoplasmic
1024	Q6IRU2	14	40	56	248	28468	4.7	Tropomyosin alpha-4 chain
1025	O35685	14	23	51.8	332	38358	5.3	Nuclear migration protein nudC
1026	Q3TF62	14	25	49.7	429	47520	5.5	Putative uncharacterized protein
1026	Q9Z2N8	14	25	49.7	429	47448	5.6	Actin-like protein 6A
1027	P97298	14	41	48.2	417	46234	7	Pigment epithelium-derived factor
1028	Q3UAG2	14	61	42.7	483	53246	7.2	6-phosphogluconate dehydrogenase, decarboxylating
1028	Q9DCD0	14	61	42.7	483	53247	7.2	6-phosphogluconate dehydrogenase, decarboxylating
1029	Q9Z0P5-2	14	80	42.9	347	39312	6.7	Isoform 2 of Twinfilin-2
1029	Q9Z0P5	14	80	42.7	349	39471	6.8	Twinfilin-2
1030	Q3TG21	14	31	42.1	382	43816	7.8	Putative uncharacterized protein
1030	Q9Z1G3	14	31	42.1	382	43888	7.5	V-type proton ATPase subunit C 1
1031	Q9Z204-4	14	322	41.8	292	32223	5.1	Isoform 4 of Heterogeneous nuclear ribonucleoproteins C1/C2
1031	Q9Z204-3	14	322	41.6	293	32322	5.1	Isoform 3 of Heterogeneous nuclear ribonucleoproteins C1/C2
1031	Q9Z204-2	14	322	40.7	300	33056	5	Isoform C1 of Heterogeneous nuclear ribonucleoproteins C1/C2
1032	P16460	14	108	39.1	412	46585	8.2	Argininosuccinate synthase
1032	Q3UEJ7	14	108	39.1	412	46557	8.2	Argininosuccinate synthase
1033	P26638	14	31	39.1	512	58389	6.3	Seryl-tRNA synthetase, cytoplasmic
1033	Q3U6F6	14	31	39.1	512	58359	6.3	Putative uncharacterized protein
1033	Q8C483	14	31	37.3	536	61167	7.4	Seryl-aminoacyl-tRNA synthetase
1034	Q61553	14	24	31.8	493	54508	6.9	Fascin
1035	P24527	14	52	31.3	611	69021	6.4	Leukotriene A-4 hydrolase
1035	Q3UY71	14	52	31.3	611	69051	6.4	Putative uncharacterized protein
1036	Q9ESB3	14	63	31.4	525	59133	7.7	Histidine-rich glycoprotein
1036	Q6YK32	14	63	30.8	536	60439	7.6	Histidine-rich glycoprotein HRG
1036	Q6YKA2	14	63	30.8	536	60495	7.6	Histidine-rich glycoprotein Hrg
1037	Q02788	14	16	22.1	1034	110334	6.4	Collagen alpha-2(VI) chain
1038	Q9UKK9	14	34	82.2	219	24328	4.9	ADP-sugar pyrophosphatase
1038	A6NFX8	14	34	77.6	232	25895	5.2	Uncharacterized protein
1039	B4DRY4	14	36	76.4	191	22610	5.3	cDNA FLJ60842, highly similar to Glutathione S-transferase Mu 2 (EC 2.5.1.18)
1039	E9PEM9	14	36	76.4	191	22644	5.3	Uncharacterized protein
1039	E9PHN6	14	36	75.6	193	22876	5.2	Uncharacterized protein
1039	P28161	14	36	67	218	25745	6.4	Glutathione S-transferase Mu 2
1039	UPI0000169F93	14	36	66.4	220	26098	5.5	Glutathione S-transferase Mu 2 (EC 2.5.1.18) (GSTM2-2) (GST class-mu 2).
1040	Q15404	14	39	74.7	277	31540	8.6	Ras suppressor protein 1
1041	P60981	14	223	73.9	165	18506	7.9	Destrin
1042	P19623	14	31	72.5	302	33825	5.5	Spermidine synthase
1043	P78417	14	37	71.8	241	27566	6.6	Glutathione S-transferase omega-1

Table S1. Proteins identified in the OVCAR-3 tumor supernatant by two or more unique peptides.

Protein or Protein Group	Accession Number	Unique Peptide Count	Spectrum Count	Sequence Coverage (%)	Length	MolWt	pI	Descriptive Name
1044	P29373	14	1007	71.7	138	15693	5.4	Cellular retinoic acid-binding protein 2
1045	Q9NPF4	14	32	70.1	335	36427	6.4	Probable O-sialoglycoprotein endopeptidase
1046	Q86X76-2	14	42	73.9	291	31859	6.9	Isoform 1 of Nitrilase log 1
1046	B7Z410	14	42	68.9	312	34265	7.6	cDNA FLJ50791, highly similar to Nitrilase log 1 (EC 3.5.-.-)
1046	Q86X76-4	14	42	68.9	312	34323	7.4	Isoform 5 of Nitrilase log 1
1046	Q86X76	14	42	65.7	327	35896	7.7	Nitrilase log 1
1047	B3KR50	14	44	67.7	217	25194	6.3	cDNA FLJ33691 fis, clone BRAWH2002976, highly similar to GROWTH FACTOR RECEPTOR-BOUND PROTEIN 2
1047	P62993	14	44	67.7	217	25206	6.3	Growth factor receptor-bound protein 2
1048	Q08257	14	22	66	329	35207	8.4	Quinone oxidoreductase
1049	Q9H4A5	14	31	66	285	32767	5.8	Golgi phosphoprotein 3-like
1050	P21964	14	54	65.7	271	30037	5.5	Catechol O-methyltransferase
1051	O14579	14	45	64	308	34482	5.1	Coatomer subunit epsilon
1052	P18065	14	92	62.5	325	34814	7.5	Insulin-like growth factor-binding protein 2
1053	E9PGT1	14	53	60.1	223	25572	6.9	Uncharacterized protein
1053	Q15631	14	53	58.8	228	26183	6.4	Translin
1054	O14818	14	53	60.1	248	27887	8.5	Proteasome subunit alpha type-7
1055	D0EKE5	14	26	57.3	337	38489	6.8	Aryl hydrocarbon receptor interacting protein
1056	Q9NX46	14	34	56.2	363	38947	5.1	Poly(ADP-ribose) glycohydrolase ARH3
1057	O14929	14	32	55.1	419	49513	5.7	Histone acetyltransferase type B catalytic subunit
1058	Q8WXX5	14	70	55	260	29910	5.7	DnaJ log subfamily C member 9
1059	Q92747	14	28	53.5	370	41569	8.2	Actin-related protein 2/3 complex subunit 1A
1060	Q9UBQ7	14	40	53.4	328	35668	7.4	Glyoxylate reductase/hydroxypyruvate reductase
1061	Q9P0L0	14	90	51	249	27893	8.6	Vesicle-associated membrane protein-associated protein A
1062	A8MUW5	14	48	50.6	433	45547	8.7	Uncharacterized protein
1063	E9PCI9	14	62	49.6	353	40532	5.2	Uncharacterized protein
1063	P14324	14	62	41.8	419	48276	6.2	Farnesyl pyrophosphate synthase
1064	B4DJQ8	14	56	46.6	446	50152	7	cDNA FLJ55694, highly similar to Dipeptidyl-peptidase 1 (EC 3.4.14.1)
1065	P09972	14	40	57.4	364	39456	6.9	Fructose-bisphosphate aldolase C
1065	B7Z3K9	14	40	46.3	451	48408	8.1	Fructose-bisphosphate aldolase
1066	Q02878	14	107	45.1	288	32728	10.6	60S ribosomal protein L6
1066	Q8TBK5	14	107	45.1	288	32742	10.6	60S ribosomal protein L6
1066	Q8N5Z7	14	107	45.1	288	32726	10.6	60S ribosomal protein L6
1066	Q9HBB3	14	107	45	289	32891	10.6	60S ribosomal protein L6
1067	P07954-2	14	33	48.8	467	50213	7.4	Isoform Cytoplasmic of Fumarate hydratase, mitochondrial
1067	P07954	14	33	44.7	510	54637	8.8	Fumarate hydratase, mitochondrial
1068	Q5JVF3-2	14	16	44.8	397	45773	8.5	Isoform 2 of PCI domain-containing protein 2
1068	Q5JVF3	14	16	44.6	399	46030	8.5	PCI domain-containing protein 2
1068	A6NM51	14	16	44.5	400	46134	8.5	Uncharacterized protein
1068	C9J439	14	16	44.4	401	46294	8.5	Uncharacterized protein
1068	Q5JVF3-4	14	16	39.3	453	52100	8.5	Isoform 4 of PCI domain-containing protein 2
1069	A8MQ60	14	33	44.2	364	41856	7	Uncharacterized protein
1069	P21283	14	33	42.1	382	43942	7.5	V-type proton ATPase subunit C 1
1070	Q9UI12-2	14	20	44.5	465	54151	6.5	Isoform 2 of V-type proton ATPase subunit H
1070	B3KUZ7	14	20	42.9	483	55911	6.5	cDNA FLJ40986 fis, clone UTERU2014898, highly similar to Vacuolar ATP synthase subunit H (EC 3.6.3.14)
1070	Q9UI12	14	20	42.9	483	55883	6.5	V-type proton ATPase subunit H
1071	P13796	14	40	42.7	627	70289	5.4	Plastin-2
1072	Q9GZT8	14	36	42.7	377	41968	6.7	NIF3-like protein 1
1073	A5PLK7	14	39	42	457	49679	8.7	RCC2 protein (Fragment)
1073	Q9P258	14	39	36.8	522	56085	8.8	Protein RCC2
1074	P31350	14	47	47	389	44878	5.4	Ribonucleoside-diphosphate reductase subunit M2
1074	D6W4Z6	14	47	40.8	449	51077	6.5	HCG23833, isoform CRA_b
1074	UPI000066D98A	14	47	40.8	449	51093	6.5	ribonucleoside-diphosphate reductase subunit M2 isoform 1
1075	P41743	14	72	39.6	596	68262	5.8	Protein kinase C iota type
1076	B2R665	14	54	39.4	546	59242	4.4	cDNA, FLJ92810, highly similar to sapiens protein phosphatase 1G (formerly 2C), magnesium-dependent, gamma isoform (PPM1G), mRNA
1076	O15355	14	54	39.4	546	59272	4.4	Protein phosphatase 1G
1077	P55795	14	122	37.4	449	49264	6.3	Heterogeneous nuclear ribonucleoprotein H2
1078	Q96JB5	14	44	31.6	506	56921	4.8	CDK5 regulatory subunit-associated protein 3
1079	Q8N8S7	14	59	31.5	591	66510	6.9	Protein enabled log
1080	Q5PRF9	14	89	31.3	694	75483	6.8	Protein Smaug log 2
1081	A0MZ66	14	25	30.1	631	71640	5.3	Shootin-1
1081	A0MZ66-3	14	25	29.3	649	73609	5.4	Isoform 3 of Shootin-1
1082	Q7Z4V5	14	41	28.2	671	74317	7.5	Hepatoma-derived growth factor-related protein 2
1083	B3KM47	14	25	22.5	1000	111115	5.5	cDNA FLJ10273 fis, clone HEMBB1001137, highly similar to SEC23-interacting protein
1083	Q9Y6Y8	14	25	22.5	1000	111076	5.5	SEC23-interacting protein
1084	UPI000011DDE0	14	29	24.7	810	90855	6.1	UPI000011DDE0 UniRef100 entry
1084	Q53GS1	14	29	22.8	878	98262	5.6	MutS log 2 variant (Fragment)
1084	Q53FK0	14	29	22.8	878	98222	5.7	MutS log 2 variant (Fragment)
1084	E9PHA6	14	29	21.7	921	103187	6.1	Uncharacterized protein
1084	P43246	14	29	21.4	934	104743	5.8	DNA mismatch repair protein Msh2
1085	B2RCJ6	14	15	19	1224	136777	6.9	cDNA, FLJ96114, highly similar to sapiens bromodomain and WD repeat domain containing 2 (BRWD2), mRNA
1085	Q9BZH6	14	15	19	1224	136685	6.9	WD repeat-containing protein 11
1085	C9J8X2	14	15	19	1224	136670	6.9	Bromodomain and WD repeat domain containing 2, isoform CRA_c
1086	Q14118	14	57	18.2	895	97441	8.6	Dystroglycan
1087	B7Z2R8	14	32	16.5	1221	137330	8	cDNA FLJ53324, highly similar to Tight junction protein ZO-2
1088	Q9P2D3-3	14	29	12.6	1982	214992	7.4	Isoform 3 of HEAT repeat-containing protein 5B
1088	B9EK47	14	29	12	2071	224274	7.2	HEAT repeat containing 5B
1088	Q9P2D3	14	29	12	2071	224300	7.2	HEAT repeat-containing protein 5B
1089	O94822	14	27	11.9	1766	200550	6.3	E3 ubiquitin-protein ligase listerin
1090	Q149P0	14	32	11.5	1855	206015	5.8	GBF1 protein
1090	UPI0001EC5F2E	14	32	11.5	1856	206143	5.8	Golgi-specific brefeldin A-resistance guanine nucleotide exchange factor 1 isoform 2
1090	Q92538	14	32	11.5	1859	206444	5.7	Golgi-specific brefeldin A-resistance guanine nucleotide exchange factor 1
1091	Q14C86-4	14	17	11.3	1439	160605	5.2	Isoform 4 of GTPase-activating protein and VPS9 domain-containing protein 1
1091	Q14C86-5	14	17	11.2	1452	162230	5.2	Isoform 5 of GTPase-activating protein and VPS9 domain-containing protein 1
1091	Q14C86-2	14	17	11.1	1460	162897	5.2	Isoform 2 of GTPase-activating protein and VPS9 domain-containing protein 1
1091	Q14C86	14	17	11	1478	164979	5.2	GTPase-activating protein and VPS9 domain-containing protein 1
1091	Q14C86-6	14	17	10.9	1487	166168	5.2	Isoform 6 of GTPase-activating protein and VPS9 domain-containing protein 1
1092	D3DS86	14	24	7.9	2610	289367	5.4	HECT domain containing 1, isoform CRA_a
1092	Q9ULT8	14	24	7.9	2610	289383	5.4	E3 ubiquitin-protein ligase HECTD1

Table S1. Proteins identified in the OVCAR-3 tumor supernatant by two or more unique peptides.

Protein or Protein Group	Accession Number	Unique Peptide Count	Spectrum Count	Sequence Coverage (%)	Length	MolWt	pI	Descriptive Name
1092	UPI00015DFD38	14	24	7.8	2612	289488	5.4	E3 ubiquitin-protein ligase HECTD1 (HECT domain-containing protein 1) (E3 ligase for inhibin receptor) (EULIR).
1093	C9JUT5	14	33	4.2	4862	532362	6	Uncharacterized protein
1093	Q15751	14	33	4.2	4861	532234	6	Probable E3 ubiquitin-protein ligase HERC1
1094	P63208	13	151	85.3	163	18658	4.5	S-phase kinase-associated protein 1
1094	Q9WTX5	13	151	85.3	163	18672	4.5	S-phase kinase-associated protein 1
1095	O00487	13	60	65.2	310	34577	6.5	26S proteasome non-ATPase regulatory subunit 14 26S proteasome non-ATPase regulatory subunit 14
1096	P62906	13	130	55.3	217	24831	9.9	60S ribosomal protein L10a 60S ribosomal protein L10a
1097	P62826	13	192	52.8	216	24423	7.5	GTP-binding nuclear protein Ran GTP-binding nuclear protein Ran
1097	Q3ULW0	13	192	52.8	216	24351	7.9	Putative uncharacterized protein
1097	UPI0000E59AAE	13	192	48.7	234	26816	9.6	GTP-binding nuclear protein Ran (GTPase Ran) (Ras-like protein TC4) (Androgen receptor-associated protein 24).
1098	P70288	13	21	43	488	55302	5.9	Histone deacetylase 2
1098	Q92769	13	21	43	488	55364	5.9	Histone deacetylase 2
1098	UPI0000E5924D	13	21	36.1	582	65538	7	Histone deacetylase 2 (HD2).
1099	Q9D5V5	13	25	22.3	780	90974	7.8	Cullin-5
1099	Q93034	13	25	22.3	780	90955	8	Cullin-5
1099	E9PV12	13	25	21	828	95851	8.8	Uncharacterized protein
1100	P62975	13	339	100	76	8565	7.2	Ubiquitin
1101	Q3UCL5	13	116	81.4	183	20756	6	Ferritin
1101	Q9CPX4	13	116	81.4	183	20772	6	Ferritin
1102	B2KFH8	13	79	75.5	188	19908	6.8	Parkinson disease (Autosomal recessive, early onset) 7
1102	Q99LX0	13	79	75.1	189	20021	6.8	Protein DJ-1
1103	Q9Z1Q5	13	50	73.9	241	27013	5.2	Chloride intracellular channel protein 1
1104	Q8CDN6	13	47	70.9	289	32237	5	Thioredoxin-like protein 1
1105	O88958	13	77	63	289	32550	6.5	Glucosamine-6-phosphate isomerase 1
1106	Q61166	13	101	61.9	268	30016	5.2	Microtubule-associated protein RP/EB family member 1
1107	Q9DCT8	13	52	61.1	208	22727	8.6	Cysteine-rich protein 2
1108	Q99PT1	13	159	58.3	204	23407	5.2	Rho GDP-dissociation inhibitor 1
1109	Q9CXW3	13	68	57.2	229	26510	7.9	Calcyclin-binding protein
1110	P53810	13	30	55.4	271	31893	6.4	Phosphatidylinositol transfer protein alpha isoform
1110	Q3UE53	13	30	55.4	271	31927	6.4	Putative uncharacterized protein
1110	Q3TGI6	13	30	43.2	347	39648	8.7	Putative uncharacterized protein (Fragment)
1110	UPI0001F79390	13	30	43	349	40036	8.7	UPI0001F79390 UniRef100 entry
1110	Q3TIQ3	13	30	42.9	350	40215	8.8	Putative uncharacterized protein (Fragment)
1111	P28352	13	71	54.6	317	35490	7.9	DNA-(apurinic or apyrimidinic site) lyase
1112	Q04447	13	41	54.3	381	42713	5.7	Creatine kinase B-type
1113	P97447	13	35	59.6	280	31889	8.4	Four and a half LIM domains protein 1
1113	A2AEX6	13	35	54	309	35081	8.4	Four and a half LIM domains 1
1114	P23492	13	29	50.9	289	32277	6.2	Purine nucleoside phosphorylase
1114	Q543K9	13	29	50.9	289	32263	6.2	Pnp protein
1115	Q00623	13	163	50.4	264	30587	5.9	Apolipoprotein A-I
1115	Q8BPD5	13	163	50.4	264	30615	5.7	Putative uncharacterized protein
1115	Q3V2G1	13	163	50.4	264	30685	5.9	Putative uncharacterized protein
1116	Q9DCJ9	13	17	50	320	35130	8	N-acetylneuraminate lyase
1117	Q3TFK4	13	24	47.1	323	36326	5.9	Putative uncharacterized protein
1117	Q8C1X9	13	24	47.1	323	36356	5.8	Annexin A3
1117	Q3UBI0	13	24	47.1	323	36384	5.8	Annexin A3
1117	Q3U737	13	24	47.1	323	36368	5.8	Putative uncharacterized protein
1118	Q93092	13	82	46.3	337	37387	7	Transaldolase
1119	Q9WV55	13	61	44.6	249	27855	8.4	Vesicle-associated membrane protein-associated protein A
1120	P14148	13	45	44.1	270	31420	10.9	60S ribosomal protein L7
1120	Q3UBI6	13	45	44.1	270	31351	10.8	Putative uncharacterized protein
1121	Q9QZF2	13	28	38.8	557	61360	7.1	Glypican-1
1122	P70333	13	126	36.1	449	49280	6.3	Heterogeneous nuclear ribonucleoprotein H2
1123	P05201	13	25	35.4	413	46232	7.2	Aspartate aminotransferase, cytoplasmic
1124	P59325	13	31	35.4	429	48968	5.5	Eukaryotic translation initiation factor 5
1125	P49182	13	47	35.1	478	54497	7.3	Heparin cofactor 2
1125	Q5FW62	13	47	35.1	478	54463	7.3	Serine (Or cysteine) peptidase inhibitor, clade D, member 1
1126	Q3TXT4	13	38	47.5	299	34907	8.4	Putative uncharacterized protein
1126	Q3TJN6	13	38	35.1	404	46798	6.4	Putative uncharacterized protein
1126	Q9D8U8	13	38	35.1	404	46797	6.6	Sorting nexin-5
1126	Q8C5E7	13	38	35.1	404	46798	6.4	Putative uncharacterized protein
1127	P97290	13	66	34.7	504	55638	6.4	Plasma protease C1 inhibitor
1128	Q5FW75	13	51	34.6	894	103919	5.5	Actinin alpha 2
1129	E9Q4P1	13	17	33.9	410	46218	7.3	Uncharacterized protein
1129	Q5DTX9	13	17	33.8	411	46348	7.3	MKIAA1435 protein (Fragment)
1130	Q06890	13	47	31.5	448	51656	5.7	Clusterin
1131	A2AAN1	13	34	26.1	625	70574	8.6	Signal recognition particle 68
1131	Q8BMA6	13	34	26.1	625	70622	8.5	Signal recognition particle 68 kDa protein
1132	UPI000154AA0D	13	34	21.4	917	101873	6.7	UPI000154AA0D UniRef100 entry
1132	Q6GQU1	13	34	21.4	918	102302	6.8	Hexokinase 1, isoform CRA_d
1132	Q3TJE3	13	34	21.4	918	102279	6.8	Putative uncharacterized protein
1132	P17710-3	13	34	21.4	918	102301	6.9	Isoform HK1 of Hexokinase-1
1132	D3Z2I0	13	34	20.8	941	105240	6.9	Uncharacterized protein
1132	UPI000154AA0B	13	34	20.7	945	105575	6.9	hexokinase-1 isoform HK1-sb
1132	P17710-2	13	34	20.7	945	105574	7	Isoform HK1-SB of Hexokinase-1
1132	P17710	13	34	20.1	974	108302	6.9	Hexokinase-1
1132	E9PXQ3	13	34	20.1	974	108303	6.8	Uncharacterized protein
1133	Q3UXN3	13	28	20.5	831	94908	6.8	Putative uncharacterized protein
1133	Q9ER72	13	28	20.5	831	94860	6.8	Cysteinyl-tRNA synthetase, cytoplasmic
1134	P07742	13	24	20.2	792	90219	6.9	Ribonucleoside-diphosphate reductase large subunit
1134	Q91YM8	13	24	20.2	792	90210	6.7	Ribonucleoside-diphosphate reductase
1134	Q6NZB3	13	24	20.2	792	90151	6.6	Ribonucleoside-diphosphate reductase
1135	P42703-2	13	33	24.8	719	81287	7	Isoform 2 of Leukemia inhibitory factor receptor
1135	P42703	13	33	16.3	1092	122574	6	Leukemia inhibitory factor receptor
1135	Q3U234	13	33	16.3	1092	122602	6	Putative uncharacterized protein
1136	Q6NXK4	13	17	13.4	1262	144270	6.6	Isoleucine-tRNA synthetase
1136	Q8BU30	13	17	13.4	1262	144256	6.6	Isoleucyl-tRNA synthetase, cytoplasmic
1137	E9QLA4	13	19	12.6	1439	157176	6.4	Uncharacterized protein
1137	Q7TQG5	13	19	12.4	1465	159967	6.7	Neogenin
1137	E9QLA3	13	19	12.3	1481	161730	6.6	Uncharacterized protein
1137	E9QK04	13	19	12.2	1492	162919	6.5	Uncharacterized protein
1138	Q8BX70-2	13	13	5.6	3623	405829	6.6	Isoform 2 of Vacuolar protein sorting-associated protein 13C
1138	E9QLN1	13	13	5.5	3709	415416	6.8	Uncharacterized protein
1138	Q8BX70-3	13	13	5.5	3708	415303	6.8	Isoform 3 of Vacuolar protein sorting-associated protein 13C
1138	Q8BX70	13	13	5.4	3748	420092	6.8	Vacuolar protein sorting-associated protein 13C
1139	Q9BRG1	13	27	76.7	176	20748	6.3	Vacuolar protein-sorting-associated protein 25
1140	P00492	13	56	76.1	218	24579	6.7	Hypoxanthine-guanine phosphoribosyltransferase
1140	Q6LET3	13	56	76.1	218	24588	6.8	HPRT1 protein (Fragment)
1141	P55327-2	13	89	75.5	184	19863	5	Isoform 2 of Tumor protein D52
1141	C9J502	13	89	74.7	186	20090	5	Uncharacterized protein

Table S1. Proteins identified in the OVCAR-3 tumor supernatant by two or more unique peptides.

Protein or Protein Group	Accession Number	Unique Peptide Count	Spectrum Count	Sequence Coverage (%)	Length	MolWt	pI	Descriptive Name
1141	D3YTI0	13	89	67.1	207	22474	5.7	Uncharacterized protein
1141	P55327	13	89	62.1	224	24327	4.8	Tumor protein D52
1141	D0UFD3	13	89	59.7	233	25401	5	Prostate leucine zipper isoform 3
1141	D0UFD2	13	89	58.4	238	25922	5	Prostate leucine zipper variant 2
1141	D0UFD1	13	89	56.3	247	26941	5.1	Prostate leucine zipper variant 1
1141	P55327-3	13	89	56	248	26382	6.4	Isoform 3 of Tumor protein D52
1142	Q96DE0	13	39	74.4	195	21273	6.9	U8 snoRNA-decapping enzyme
1143	Q9H0R4	13	31	74.1	259	28536	6.2	Haloacid dehalogenase-like hydrolase domain-containing protein 2
1144	O00299	13	68	73.9	241	26923	5.2	Chloride intracellular channel protein 1
1144	Q53FB0	13	68	73.9	241	27015	5.2	Chloride intracellular channel 1 variant (Fragment)
1145	P30039	13	20	73.3	288	31785	6.5	Phenazine biosynthesis-like domain-containing protein
1146	Q59G46	13	54	73.2	280	31355	4.8	Thioredoxin-like 1 variant (Fragment)
1146	B2R960	13	54	70.9	289	32237	5	cDNA, FLJ94230, highly similar to sapiens thioredoxin-like 1 (TXNL1), mRNA
1146	O43396	13	54	70.9	289	32251	5	Thioredoxin-like protein 1
1147	Q53G01	13	49	70.1	251	28179	6	Chloride intracellular channel 5 variant (Fragment)
1147	Q9NZA1	13	49	42.9	410	46503	4.8	Chloride intracellular channel protein 5
1148	P09211	13	313	69.5	210	23356	5.6	Glutathione S-transferase P
1149	Q96CN7	13	46	69.5	298	32237	7.4	Isochorismatase domain-containing protein 1
1150	P04183	13	37	68.4	234	25469	8.5	Thymidine kinase, cytosolic
1151	A8MW68	13	41	67.1	286	33066	7	Uncharacterized protein
1151	Q53GC7	13	41	67.1	286	32967	5.8	Capping protein (Actin filament) muscle Z-line, alpha 2 variant (Fragment)
1151	P47755	13	41	67.1	286	32949	5.8	F-actin-capping protein subunit alpha-2
1152	UPI0001DD3809	13	33	71.3	279	30792	4.4	peroxisomal biogenesis factor 19 isoform c
1152	P40855	13	33	66.6	299	32807	4.3	Peroxisomal biogenesis factor 19
1153	O43399	13	135	66.5	206	22238	5.4	Tumor protein D54
1153	UPI0000D610E3	13	135	62.6	219	23739	6.2	Tumor protein D54 (hD54) (Tumor protein D52-like 2).
1153	Q5U0E0	13	135	62.3	220	23787	5.7	Tumor protein D52-like 2
1153	Q5JWU6	13	135	59.8	229	24854	6.4	Tumor protein D52-like 2
1154	P41227	13	31	66.4	235	26458	5.6	N-alpha-acetyltransferase 10, NatA catalytic subunit
1155	P31150	13	32	63.8	447	50583	5.1	Rab GDP dissociation inhibitor alpha
1156	P30085	13	55	61.7	196	22222	5.6	UMP-CMP kinase
1157	Q96GG9	13	34	61.4	259	30124	5.3	DCN1-like protein 1
1158	P09104	13	20	60.8	434	47269	5	Gamma-enolase
1159	Q96GX9	13	43	64.9	242	27125	7.1	Probable methylthioribulose-1-phosphate dehydratase
1159	B4DY17	13	43	60.6	259	29255	8.2	cDNA FLJ58961, highly similar to APAF1-interacting protein
1160	P09661	13	40	60.4	255	28416	8.6	U2 small nuclear ribonucleoprotein A'
1161	Q53H82	13	32	59.7	288	32806	6.8	Beta-lactamase-like protein 2
1162	Q9GZS3	13	31	59.7	305	33581	5.5	WD repeat-containing protein 61
1163	Q9UHD1	13	52	58.7	332	37490	7.9	Cysteine and histidine-rich domain-containing protein 1
1164	O00273	13	59	57.4	331	36522	4.8	DNA fragmentation factor subunit alpha
1164	Q53HN4	13	59	57.4	331	36594	4.8	DNAation factor, 45kDa, alpha polypeptide isoform 1 variant (Fragment)
1165	O15305	13	29	57.3	246	28082	6.8	Phosphomannomutase 2
1166	P53004	13	36	57.1	296	33428	6.4	Biliverdin reductase A
1167	Q9Y315	13	21	56.6	318	35231	8.9	Putative deoxyribose-phosphate aldolase
1168	Q53FI7	13	32	59.6	280	31896	8.2	Four and a half LIM domains 1 variant (Fragment)
1168	B7Z5T4	13	32	56.4	296	33579	8.4	Four and a half LIM domains 1, isoform CRA c
1168	B7Z793	13	32	54	309	34997	8.3	cDNA FLJ55259, highly similar to Four and a half LIM domains protein 1
1169	Q53FA7	13	20	54.5	332	35536	7.2	Quinone oxidoreductase PIG3
1170	P78346	13	23	63.8	268	29321	8.9	Ribonuclease P protein subunit p30
1170	E9PB02	13	23	53.1	322	35564	9.6	Uncharacterized protein
1171	P07858	13	101	52.8	339	37822	6.3	Cathepsin B
1172	Q14192	13	28	52.7	279	32193	7.5	Four and a half LIM domains protein 2
1172	UPI00015E0A35	13	28	37.8	389	44205	8.1	Four and a half LIM domains protein 2 (FHL-2) (Skeletal muscle LIM- protein 3) (SLIM 3) (LIM domain protein DRAL).
1172	Q2XQU9	13	28	37.8	389	44177	8.1	FHL2 isoform 5
1173	P22061	13	43	66.1	227	24636	7.2	Protein-L-isoaspartate(D-aspartate) O-methyltransferase
1173	E9PAN3	13	43	52.4	286	30358	6.7	Uncharacterized protein
1174	O75367-3	13	33	52.3	371	39489	9.8	Isoform 3 of Core histone macro-H2A.1
1174	O75367	13	33	52.2	372	39617	9.8	Core histone macro-H2A.1
1175	P19784	13	23	52	350	41213	8.6	Casein kinase II subunit alpha'
1176	P53367	13	51	51.5	373	41738	6.7	Arfaptin-1
1177	Q5VU58	13	41	51.2	248	28922	4.8	Tropomyosin 3
1178	Q5TDH0	13	47	51.1	399	44523	5	Protein DDI1 log 2
1178	Q5TDH0-3	13	47	48.7	419	46571	5.1	Isoform 3 of Protein DDI1 log 2
1179	Q99536	13	31	50.6	393	41920	6.3	Synaptic vesicle membrane protein VAT-1 log
1180	O14908	13	27	50.2	333	36049	6.3	PDZ domain-containing protein GIPC1
1181	O75347	13	43	54.6	108	12855	5.3	Tubulin-specific chaperone A
1181	E5RJD8	13	43	49.6	119	14306	5.1	Uncharacterized protein
1182	Q15717	13	63	52.5	326	36092	9.2	ELAV-like protein 1
1182	B4DVB8	13	63	48.4	353	38996	9.5	cDNA FLJ60076, highly similar to ELAV-like protein 1
1183	Q12904	13	68	47.8	312	34353	8.4	Aminoacyl tRNA synthase complex-interacting multifunctional protein 1
1184	B4DR80	13	18	47.1	412	45836	5.6	cDNA FLJ61159, highly similar to Serine/threonine-protein kinase 24 (EC 2.7.11.1)
1184	Q5U0E6	13	18	45	431	47913	5.4	Serine/threonine kinase 24 (STE20 log, yeast)
1184	Q9Y6E0	13	18	43.8	443	49308	5.7	Serine/threonine-protein kinase 24
1184	B4DT57	13	18	43.1	450	49998	5.6	cDNA FLJ61383, highly similar to Serine/threonine-protein kinase 24 (EC 2.7.11.1)
1185	P07339	13	63	46.6	412	44552	6.5	Cathepsin D
1186	Q9UNE7	13	19	46.5	303	34856	5.9	E3 ubiquitin-protein ligase CHIP
1187	Q14847	13	115	56.3	261	29717	7	LIM and SH3 domain protein 1
1187	C9JVR8	13	115	46.2	318	35446	8.5	Uncharacterized protein
1187	Q14847-2	13	115	45.5	323	36014	8.6	Isoform 2 of LIM and SH3 domain protein 1
1188	Q96KB5	13	22	46	322	36085	5.1	Lymphokine-activated killer T-cell-originated protein kinase
1188	B4DX68	13	22	44.4	333	37211	5.1	cDNA FLJ58333, highly similar to T-lymphokine-activated killer cell-originated protein kinase (EC 2.7.12.2)
1189	Q9UL15	13	23	43.8	447	51200	6	BAG family molecular chaperone regulator 5
1190	P18031	13	34	43.4	435	49967	6.3	Tyrosine-protein phosphatase non-receptor type 1
1191	P29218	13	30	51.6	277	30189	5.3	Inositol monophosphatase 1
1191	B7Z6Q4	13	30	42.6	336	36723	8.1	cDNA FLJ54837, highly similar to Inositol monophosphatase (EC 3.1.3.25)
1191	UPI000192950E	13	30	42.6	336	36695	7.9	inositol monophosphatase 1 isoform 2
1192	Q16543	13	76	41.3	378	44468	5.2	Hsp90 co-chaperone Cdc37
1193	E7EVX8	13	45	40.4	493	54766	5.8	Uncharacterized protein
1193	Q8WWY3	13	45	39.9	499	55456	5.8	U4/U6 small nuclear ribonucleoprotein Prp31
1194	Q9H3P7	13	40	40.2	528	60593	5.1	Golgi resident protein GCP60
1195	B7Z254	13	41	43.2	437	47838	5.1	cDNA FLJ58502, highly similar to Protein disulfide-isomerase A6 (EC 5.3.4.1)

Table S1. Proteins identified in the OVCAR-3 tumor supernatant by two or more unique peptides.

Protein or Protein Group	Accession Number	Unique Peptide Count	Spectrum Count	Sequence Coverage (%)	Length	MolWt	pI	Descriptive Name
1195	Q15084	13	41	43	440	48121	5.1	Protein disulfide-isomerase A6
1195	B7Z4M8	13	41	42.5	445	48701	5.2	cDNA FLJ58023, highly similar to Protein disulfide-isomerase A6 (EC 5.3.4.1)
1195	B5MBW7	13	41	42.5	445	48646	5.1	Uncharacterized protein
1195	B3KY95	13	41	38.7	488	53289	5.5	cDNA FLJ16143 fis, clone BRAMY2038516, highly similar to Protein disulfide-isomerase A6 (EC 5.3.4.1)
1196	P30419	13	42	38.7	496	56806	7.8	Glycylpeptide N-tetradecanoyltransferase 1
1197	Q15942	13	33	38.1	572	61277	6.7	Zyxin
1198	Q9UBB4	13	25	37.9	475	53489	5.2	Ataxin-10
1199	P49354	13	33	44.3	379	44409	5.1	Protein farnesyltransferase/geranylgeranyltransferase type-1 subunit alpha
1199	D3DSY9	13	33	36.8	457	52610	5.6	Farnesyltransferase, CAAX box, alpha, isoform CRA_a
1200	B2RDR4	13	28	36.6	421	47966	7.7	cDNA, FLJ96732, highly similar to sapiens testis derived transcript (3 LIM domains) (TES), transcript variant 1, mRNA
1200	Q9UGI8	13	28	36.6	421	47996	7.7	Testin
1201	B7Z7F3	13	27	36	494	52895	4.8	cDNA FLJ58549, highly similar to Ran-binding protein 3
1201	Q9H6Z4-2	13	27	31.7	562	59694	4.8	Isoform 2 of Ran-binding protein 3
1202	P16930	13	22	35.8	419	46374	6.9	Fumarylacetoacetase
1203	P17812	13	40	35.4	591	66690	6.5	CTP synthase 1
1204	P12931	13	20	32.5	536	59835	7.4	Proto-oncogene tyrosine-protein kinase Src
1204	P12931-2	13	20	32.1	542	60589	7.8	Isoform 2 of Proto-oncogene tyrosine-protein kinase Src
1205	B4DEP6	13	184	32.4	587	66128	5.7	cDNA FLJ59206, highly similar to Eukaryotic translation initiation factor 4B
1205	E7EPC9	13	184	32.4	587	66113	5.7	Uncharacterized protein
1205	P23588	13	184	31.1	611	69151	5.7	Eukaryotic translation initiation factor 4B
1205	E7EX17	13	184	30.8	616	69698	5.7	Uncharacterized protein
1205	B4DRM3	13	184	30.8	616	69726	5.7	cDNA FLJ54492, highly similar to Eukaryotic translation initiation factor 4B
1206	Q15642-4	13	24	41.7	456	52700	6.8	Isoform 4 of Cdc42-interacting protein 4
1206	Q15642-2	13	24	34.9	545	62592	5.3	Isoform 2 of Cdc42-interacting protein 4
1206	UPI000059D64B	13	24	32	593	67731	6.1	UPI000059D64B UniRef100 entry
1206	Q15642-3	13	24	32	593	67603	6.2	Isoform 3 of Cdc42-interacting protein 4
1206	Q15642	13	24	31.6	601	68352	5.7	Cdc42-interacting protein 4
1207	P07686	13	27	31.1	556	63111	6.8	Beta-hexosaminidase subunit beta
1208	Q96RS6	13	20	30.9	583	66756	5.1	NudC domain-containing protein 1
1209	Q05BV5	13	36	30.2	660	71801	7	OGFR protein
1210	Q9UBF2	13	32	29.6	871	97622	5.8	Coatomer subunit gamma-2
1211	UPI00015DFD4A	13	30	32.4	574	65012	6	Sec1 family domain-containing protein 1 (Syntaxin-binding protein 1- like 2) (Sly1p).
1211	B7Z4U7	13	30	32.3	575	65061	5.8	Sec1 family domain containing 1, isoform CRA_b
1211	B7Z738	13	30	31.9	583	66046	5.8	cDNA FLJ57213, highly similar to Sec1 family domain-containing protein 1
1211	A8MVJ7	13	30	29	642	72378	6.3	Uncharacterized protein
1211	Q8WVM8	13	30	29	642	72380	6.3	Sec1 family domain-containing protein 1
1211	Q53GW1	13	30	29	642	72308	6.4	Vesicle transport-related protein isoform a variant (Fragment)
1212	Q9Y6M1-1	13	22	31.3	556	61843	8.3	Isoform 2 of Insulin-like growth factor 2 mRNA-binding protein 2
1212	Q9Y6M1	13	22	29	599	66121	8.5	Insulin-like growth factor 2 mRNA-binding protein 2
1213	Q96GF2	13	28	29.1	633	68448	5.3	GSPT1 protein
1213	E7EQZ3	13	28	28.9	636	68601	5.3	Uncharacterized protein
1213	UPI0000EE662A	13	28	28.9	637	68672	5.3	eukaryotic peptide chain release factor GTP-binding subunit ERF3A isoform 1
1213	UPI0000EE6629	13	28	28.9	636	68573	5.3	eukaryotic peptide chain release factor GTP-binding subunit ERF3A isoform 2
1213	UPI000049DE01	13	28	28.9	637	68700	5.3	UPI000049DE01 UniRef100 entry
1214	Q9H6S3	13	31	27.3	715	80621	6.8	Epidermal growth factor receptor kinase substrate 8-like protein 2
1214	B7ZKL3	13	31	26.7	731	82323	7	EPS8L2 protein
1215	Q13421-4	13	55	27.2	621	67941	6.2	Isoform 4 of Mesothelin
1215	Q13421-3	13	55	27.2	622	68070	6	Isoform 2 of Mesothelin
1215	C9J1E9	13	55	25.8	656	71524	7.4	Uncharacterized protein
1215	Q13421-2	13	55	25.8	656	71492	7.4	Isoform 3 of Mesothelin
1216	Q14444-2	13	136	26.1	694	76862	5.1	Isoform 2 of Caprin-1
1216	Q14444	13	136	25.5	709	78366	5.2	Caprin-1
1217	UPI0001AE6845	13	45	23.4	849	93864	4.7	UPI0001AE6845 UniRef100 entry
1218	P18858	13	22	20.7	919	101736	5.6	DNA ligase 1
1219	Q96A65	13	22	19.6	974	110498	6.5	Exocyst complex component 4
1220	Q8WX93-4	13	35	21.4	672	73322	7.7	Isoform 4 of Palladin
1220	B3KTG2	13	35	18.5	777	85894	6.9	cDNA FLJ38193 fis, clone FCBBF1000280, highly similar to sapiens palladin, cytoskeletal associated protein (PALLD), mRNA
1220	Q8WX93-3	13	35	14.4	1001	108631	7.3	Isoform 3 of Palladin
1220	Q8WX93-5	13	35	12.4	1159	127826	6.9	Isoform 5 of Palladin
1220	Q8WX93	13	35	10.4	1383	150564	7.1	Palladin
1221	Q9NYF8	13	57	18.3	920	106122	10	Bcl-2-associated transcription factor 1
1221	Q9NYF8-2	13	57	18.3	918	105948	10	Isoform 2 of Bcl-2-associated transcription factor 1
1222	B4E3I7	13	27	17.7	986	110732	5.3	cDNA FLJ56396, highly similar to Huntingtin-interacting protein 1
1222	E7ES17	13	27	17.7	986	110663	5.3	Uncharacterized protein
1222	O00291	13	27	16.9	1037	116221	5.3	Huntingtin-interacting protein 1
1223	B7ZLP5	13	68	17	914	102581	5.4	SAFB protein
1223	A0AV56	13	68	16.9	917	102855	5.5	SAFB protein
1223	Q15424	13	68	16.9	915	102642	5.5	Scaffold attachment factor B1
1223	B7ZLP6	13	68	16.9	916	102768	5.5	SAFB protein
1223	A8K329	13	68	16.9	916	102754	5.4	cDNA FLJ76656, highly similar to sapiens scaffold attachment factor B (SAFB), mRNA
1224	Q8WWM7	13	27	16.2	1075	113374	8.6	Ataxin-2-like protein
1225	O14617-5	13	13	13.7	1215	136651	7.6	Isoform 5 of AP-3 complex subunit delta-1
1226	Q8IX12-2	13	51	13.8	1135	131043	5.8	Isoform 2 of Cell division cycle and apoptosis regulator protein 1
1226	Q8IX12	13	51	13.7	1150	132821	5.8	Cell division cycle and apoptosis regulator protein 1
1227	Q14160	13	34	12.5	1630	174884	5.1	Protein scribble log
1227	E7EMI2	13	34	12.3	1654	177194	5.1	Uncharacterized protein
1227	UPI000035199C	13	34	12.3	1655	177739	5.1	protein scribble log isoform a
1227	Q14160-3	13	34	12.3	1655	177693	5.1	Isoform 3 of Protein scribble log
1228	P52701	13	25	11.8	1360	152785	6.9	DNA mismatch repair protein Msh6
1229	O95602	13	13	11.2	1720	194810	7	DNA-directed RNA polymerase I subunit RPA1
1230	Q7Z460-2	13	36	10.3	1471	162110	8.8	Isoform 2 of CLIP-associating protein 1
1230	E7EUA5	13	36	10.3	1471	162067	8.8	Uncharacterized protein
1230	A2RU21	13	36	10.2	1479	162914	8.8	CLASP1 protein
1230	B7ZLX3	13	36	10.2	1477	163061	8.5	CLASP1 protein
1230	Q7Z460-3	13	36	10.1	1494	164565	8.5	Isoform 3 of CLIP-associating protein 1
1230	Q7Z460	13	36	9.8	1538	169450	9	CLIP-associating protein 1
1231	Q69YN4-3	13	25	9.6	1797	201055	4.8	Isoform 3 of Protein virilizer log
1231	Q69YN4	13	25	9.5	1812	202023	5	Protein virilizer log
1232	Q6ZNJ1	13	27	8.4	2754	302515	6.4	Neurobeachin-like protein 2

Table S1. Proteins identified in the OVCAR-3 tumor supernatant by two or more unique peptides.

Protein or Protein Group	Accession Number	Unique Peptide Count	Spectrum Count	Sequence Coverage (%)	Length	MolWt	pI	Descriptive Name
1233	D3DUP2	13	40	7.2	2107	222701	6.4	WNK lysine deficient protein kinase 1, isoform CRA_d
1233	UPI0001D411C5	13	40	7.1	2134	225501	6.4	serine/threonine-protein kinase WNK1 isoform 2
1233	Q9H4A3-2	13	40	7.1	2135	225558	6.4	Isoform 2 of Serine/threonine-protein kinase WNK1
1233	D3DUP1	13	40	6.3	2382	250753	6.3	WNK lysine deficient protein kinase 1, isoform CRA_b
1233	Q9H4A3	13	40	6.3	2382	250791	6.3	Serine/threonine-protein kinase WNK1
1233	Q9H4A3-6	13	40	5.7	2642	279711	6.3	Isoform 5 of Serine/threonine-protein kinase WNK1
1233	Q9H4A3-5	13	40	5.7	2634	279535	6.5	Isoform 4 of Serine/threonine-protein kinase WNK1
1234	Q15365	12	169	71.9	356	37498	7.1	Poly(rC)-binding protein 1 Poly(rC)-binding protein 1
1235	B5MCE8	12	27	69.1	278	31704	8.2	Uncharacterized protein
1235	Q1JQB2	12	27	58.9	326	36955	6.8	Mitotic checkpoint protein BUB3 Mitotic checkpoint protein BUB3
1235	O43684	12	27	58.5	328	37155	6.8	Mitotic checkpoint protein BUB3
1236	P35293	12	48	67	206	23035	5.4	Ras-related protein Rab-18
1236	Q9NP72	12	48	67	206	22977	5.2	Ras-related protein Rab-18
1237	B4DY28	12	195	66.8	187	20042	8.5	cDNA FLJ61189, highly similar to Cysteine and glycine-rich protein 1
1237	P97315	12	195	64.8	193	20583	8.6	Cysteine and glycine-rich protein 1
1237	P21291	12	195	64.8	193	20567	8.6	Cysteine and glycine-rich protein 1
1238	P53994	12	135	64.2	212	23548	6.5	Ras-related protein Rab-2A
1238	P61019	12	135	64.2	212	23546	6.5	Ras-related protein Rab-2A
1239	Q9D3C4	12	136	69	168	19653	8.4	Putative uncharacterized protein
1239	Q7TPD9	12	136	69	168	19607	8.4	Arpc4 protein
1239	P59998	12	136	69	168	19667	8.4	Actin-related protein 2/3 complex subunit 4 Actin-related protein 2/3 complex subunit 4
1239	UPI000198C8E6	12	136	64.1	181	21059	8.8	UPI000198C8E6 UniRef100 entry
1239	C9JWM7	12	136	62	187	21588	8.6	Uncharacterized protein
1240	P61289	12	74	60.6	254	29506	6	Proteasome activator complex subunit 3 Proteasome activator complex subunit 3
1240	P61289-2	12	74	57.7	267	30887	6.1	Isoform 2 of Proteasome activator complex subunit 3
1241	P83916	12	145	60.5	185	21418	4.9	Chromobox protein log 1 Chromobox protein log 1
1241	E9PCS5	12	145	59.3	189	21915	4.9	Uncharacterized protein
1242	D3YWT1	12	129	57.1	331	35182	6.9	MCG11326, isoform CRA_b
1242	P31942-2	12	129	57.1	331	35239	6.9	Isoform 2 of Heterogeneous nuclear ribonucleoprotein H3
1242	P31942	12	129	54.6	346	36926	6.9	Heterogeneous nuclear ribonucleoprotein H3
1242	D3Z3N4	12	129	54.6	346	36869	6.9	MCG11326, isoform CRA_a
1243	P62917	12	138	52.9	257	28025	11	60S ribosomal protein L8 60S ribosomal protein L8
1243	Q3UJS0	12	138	52.9	257	28067	11	Putative uncharacterized protein
1244	D3YWP3	12	135	49.4	156	17698	10.4	MCG1034428
1244	UPI0000026315	12	135	49.4	156	17725	10.3	PREDICTED: 60S ribosomal protein L23a-like
1244	P62750	12	135	49.4	156	17695	10.4	60S ribosomal protein L23a 60S ribosomal protein L23a
1245	P31153	12	28	48.1	395	43661	6.5	S-adenosylmethionine synthase isoform type-2
1245	Q3THS6	12	28	48.1	395	43689	6.5	S-adenosylmethionine synthase isoform type-2
1246	Q6UXN9	12	45	45.7	313	35079	7.7	WD repeat-containing protein 82 WD repeat-containing protein 82
1247	Q96BS4	12	27	55.8	260	28449	9.4	FBL protein (Fragment)
1247	P22087	12	27	45.2	321	33784	10.2	rRNA 2'-O-methyltransferase fibrillarin
1247	Q3UJS2	12	27	44.3	327	34376	10.3	Putative uncharacterized protein
1247	P35550	12	27	44.3	327	34307	10.2	rRNA 2'-O-methyltransferase fibrillarin
1248	E9PWH9	12	50	43.1	204	24127	11.6	Uncharacterized protein
1248	Q3U7D2	12	50	43.1	204	24146	11.6	Ribosomal protein L15
1248	P61313	12	50	43.1	204	24146	11.6	60S ribosomal protein L15 60S ribosomal protein L15
1248	E9QAZ2	12	50	43.1	204	24077	11.6	Uncharacterized protein
1249	P61759	12	37	50.5	196	22436	6.3	Prefoldin subunit 3
1249	P61758	12	37	50.3	197	22658	7.1	Prefoldin subunit 3
1249	D3DWY7	12	37	42.5	233	26535	9	von Hippel-Lindau binding protein 1, isoform CRA_b
1250	D3YYL9	12	292	41.6	317	34226	8.8	Uncharacterized protein
1250	Q6P6I7	12	292	41.5	318	34377	9	Hnrpa3 protein
1250	E7ERJ4	12	292	41.5	318	34476	9.1	Uncharacterized protein Uncharacterized protein
1250	E7EN79	12	292	38.4	344	36668	9	Uncharacterized protein
1250	UPI0001E6B716	12	292	36.5	362	39298	9	PREDICTED: heterogeneous nuclear ribonucleoprotein A3-like
1250	P51991	12	292	34.9	378	39595	9	Heterogeneous nuclear ribonucleoprotein A3
1250	Q8BG05	12	292	34.8	379	39652	9	Heterogeneous nuclear ribonucleoprotein A3
1250	Q3UZG3	12	292	34.8	379	39751	9.1	Putative uncharacterized protein
1251	B7U582	12	173	26.2	633	69724	5.7	Heat shock protein 70-2
1251	P14659	12	173	26.2	633	69642	5.7	Heat shock-related 70 kDa protein 2
1251	P54652	12	173	26	639	70021	5.7	Heat shock-related 70 kDa protein 2
1252	B2RY56-2	12	53	19	838	99552	6.3	Isoform 2 of RNA-binding protein 25
1252	B2RY56	12	53	18.9	841	99926	6.4	RNA-binding protein 25
1252	P49756	12	53	18.9	843	100186	6.3	RNA-binding protein 25
1253	Q9R1P3	12	50	76.1	201	22906	7	Proteasome subunit beta type-2
1254	Q9R0P5	12	83	69.1	165	18522	8	Destrin
1255	P00920	12	42	68.1	260	29033	7	Carbonic anhydrase 2
1256	P47754	12	34	67.1	286	32967	5.8	F-actin-capping protein subunit alpha-2
1257	O08547	12	120	66.5	215	24741	8.5	Vesicle-trafficking protein SEC22b
1258	Q61171	12	59	65.7	198	21779	5.4	Peroxiredoxin-2
1259	Q99LD8	12	33	65.6	285	29646	6	N(G),N(G)-dimethylarginine dimethylaminohydrolase 2
1260	E9QNF4	12	44	64.3	168	19343	8.6	Uncharacterized protein
1260	Q6PGB6-2	12	44	64.3	168	19327	8.8	Isoform 2 of N-alpha-acetyltransferase 50, NatE catalytic subunit
1260	Q6PGB6	12	44	63.9	169	19414	8.8	N-alpha-acetyltransferase 50, NatE catalytic subunit
1260	Q6PGB6-4	12	44	61.7	175	20044	8.4	Isoform 4 of N-alpha-acetyltransferase 50, NatE catalytic subunit
1261	Q9CQ65	12	38	56.2	283	31062	7.1	S-methyl-5'-thioadenosine phosphorylase
1262	P17918	12	37	53.3	261	28785	4.8	Proliferating cell nuclear antigen
1262	Q91ZH2	12	37	53.3	261	28845	4.8	Proliferating cell nuclear antigen
1263	E9Q450	12	35	52.5	284	32848	4.8	Uncharacterized protein
1264	P08249	12	25	49.4	338	35611	8.7	Malate dehydrogenase, mitochondrial
1265	O70433	12	26	48	279	32073	7.3	Four and a half LIM domains protein 2
1265	Q8VDP9	12	26	48	279	32057	7.3	Four and a half LIM domains 2
1266	O70250	12	34	47.8	253	28827	8.5	Phosphoglycerate mutase 2
1267	O55215	12	90	55.2	250	27231	9.6	Ribosomal protein S2
1267	E9PV46	12	90	47.1	293	31169	10.1	Uncharacterized protein
1267	Q3TXS9	12	90	47.1	293	31188	10.2	Putative uncharacterized protein
1267	P25444	12	90	47.1	293	31231	10.2	40S ribosomal protein S2
1268	O70435	12	32	43.9	255	28405	5.4	Proteasome subunit alpha type-3
1268	Q9DCD8	12	32	43.9	255	28490	5.6	Proteasome subunit alpha type
1269	Q3U3T6	12	18	42.5	431	47955	5.3	Putative uncharacterized protein
1269	Q99KH8	12	18	42.5	431	47954	5.4	Serine/threonine-protein kinase 24
1270	Q91WQ0	12	49	38	397	44735	5.2	Serine (Or cysteine) peptidase inhibitor, clade A, member 6
1271	P47911	12	47	35.1	296	33510	10.7	60S ribosomal protein L6
1271	UPI000024FB1D	12	47	35.1	296	33482	10.6	PREDICTED: 60S ribosomal protein L6-like
1272	Q8CHH9-2	12	19	34	430	49974	6.1	Isoform 2 of Septin-8
1272	Q8CHH9	12	19	34	429	49812	6	Septin-8
1272	B7ZC46	12	19	33.2	440	50896	6.1	Septin 8
1272	B1AQY9	12	19	33	442	51146	6.1	Septin 8
1273	Q9DBC7	12	19	31	381	43185	5.3	cAMP-dependent protein kinase type I-alpha regulatory subunit
1273	Q3TYK4	12	19	23.1	510	57281	6.9	Putative uncharacterized protein

Table S1. Proteins identified in the OVCAR-3 tumor supernatant by two or more unique peptides.

Protein or Protein Group	Accession Number	Unique Peptide Count	Spectrum Count	Sequence Coverage (%)	Length	MolWt	pI	Descriptive Name
1274	Q3UGD0	12	59	22.2	591	63299	8.2	Putative uncharacterized protein
1274	Q8CI51	12	59	22.2	591	63329	8.2	PDZ and LIM domain protein 5
1275	Q62029	12	111	19.4	628	69061	8.8	Poly A binding protein, cytoplasmic 2
1276	Q9JKR6	12	17	19.4	999	111181	5.2	Hypoxia up-regulated protein 1
1277	Q7TNZ4	12	14	16.6	842	93587	7	Glycosylphosphatidylinositol-specific phospholipase D
1277	Q8VCU2	12	14	16.6	842	93624	6.8	Glycosylphosphatidylinositol specific phospholipase D1
1277	Q8R432	12	14	16.6	842	93654	6.7	Glycosylphosphatidylinositol phospholipase D
1278	Q80UE5	12	28	17	918	101814	5.3	Erythrocyte protein band 4.1-like 2, isoform CRA_c
1278	B6ZHD0	12	28	15.8	988	109895	5.4	Erythrocyte protein band 4.1-like 2
1278	UPI000024DA70	12	28	15.8	988	109940	5.4	band 4.1-like protein 2
1278	Q80UE3	12	28	15.8	988	109970	5.4	Protein 4.1G
1278	Q3UNQ6	12	28	15.8	988	109997	5.4	Putative uncharacterized protein
1278	O70318	12	28	15.8	988	109833	5.5	Band 4.1-like protein 2
1279	Q8C2E7	12	14	14.2	1159	134110	7.1	WASH complex subunit strumpellin
1280	Q6WKZ8	12	12	9.5	1755	199154	6.3	E3 ubiquitin-protein ligase UBR2
1280	Q6WKZ8-2	12	12	9.5	1755	199197	6.3	Isoform 2 of E3 ubiquitin-protein ligase UBR2
1281	P39061-2	12	30	12.4	1315	134204	6	Isoform 3 of Collagen alpha-1(XVIII) chain
1281	P39061-1	12	30	10.7	1527	155950	5.4	Isoform 2 of Collagen alpha-1(XVIII) chain
1281	E9QPX1	12	30	9.2	1774	182296	5.7	Uncharacterized protein
1281	P39061	12	30	9.2	1774	182171	5.6	Collagen alpha-1(XVIII) chain
1282	A2ASQ1	12	58	8.2	1950	207537	6.3	Agrin
1283	Q6PDN3	12	31	7.9	1941	212924	6.2	Myosin light chain kinase, smooth muscle
1283	B1B1A8	12	31	7.8	1950	213608	6.2	Myosin, light polypeptide kinase
1284	O76070	12	111	80.3	127	13331	4.9	Gamma-synuclein
1285	P05161	12	167	77	165	17887	7.5	Ubiquitin-like protein ISG15
1286	O95336	12	39	76.7	258	27547	6.1	6-phosphogluconolactonase
1287	P30086	12	65	75.9	187	21057	7.5	Phosphatidylethanolamine-binding protein 1
1288	Q9BUH6	12	38	73.5	204	21640	5.5	Uncharacterized protein C9orf142
1289	Q9BU89	12	17	73.2	302	32904	4.8	Deoxyhypusine hydroxylase
1290	Q9UKY7	12	47	72.1	258	27335	6.4	Protein CDV3 log
1291	Q8N959	12	110	79.6	323	35226	6.3	cDNA FLJ38330 fis, clone FCBBF3025280, highly similar to NDRG1 PROTEIN
1291	E9PDL6	12	110	72	357	38701	6.2	Uncharacterized protein
1291	Q92597	12	110	65.2	394	42835	5.8	Protein NDRG1
1291	Q597H1	12	110	65.2	394	42819	5.8	Transformation-related protein 14
1292	UPI0000E5A10A	12	53	72.3	242	25743	8.1	L-xylulose reductase isoform 2
1292	Q7Z4W1	12	53	71.7	244	25913	8.1	L-xylulose reductase
1293	P20338	12	54	71.4	213	23871	6.1	Ras-related protein Rab-4A
1294	Q8IXM2	12	21	78.5	172	17900	7.3	Chromatin complexes subunit BAP18
1294	C9J4G0	12	21	70.3	192	19961	5.6	HCG32827, isoform CRA_d
1295	P52566	12	43	70.1	201	22988	5.2	Rho GDP-dissociation inhibitor 2
1296	P20618	12	74	69.7	241	26489	8.1	Proteasome subunit beta type-1
1296	Q53FT8	12	74	69.7	241	26491	8.1	Proteasome beta 1 subunit variant (Fragment)
1297	P54819-2	12	27	70.7	232	25615	7.9	Isoform 2 of Adenylate kinase 2, mitochondrial
1297	P54819	12	27	68.6	239	26478	7.8	Adenylate kinase 2, mitochondrial
1298	Q9UBQ5	12	65	68.3	218	25060	4.9	Eukaryotic translation initiation factor 3 subunit K
1299	P00568	12	64	67	194	21635	8.6	Adenylate kinase isoenzyme 1
1299	Q53EY8	12	64	67	194	21619	8.9	Adenylate kinase 1 variant (Fragment)
1299	Q5T9B7	12	64	61.9	210	23411	8.6	Adenylate kinase 1
1300	Q9HD15	12	42	65.7	236	25673	7	Steroid receptor RNA activator 1
1301	E7EQ69	12	48	64.3	168	19311	8.8	Uncharacterized protein
1301	Q9GZZ1	12	48	63.9	169	19398	8.8	N-alpha-acetyltransferase 50, NatE catalytic subunit
1302	Q04323	12	25	63.6	297	33325	5.3	UBX domain-containing protein 1
1303	P51580	12	22	63.3	245	28180	6.2	Thiopurine S-methyltransferase
1303	Q9BS45	12	22	63.3	245	28233	6.4	Thiopurine S-methyltransferase
1304	Q9H3H3	12	25	63.3	251	27355	5.5	UPF0696 protein C11orf68
1304	UPI0000228EAC	12	25	54.3	293	31517	6.3	basophilic leukemia expressed protein BLES03 isoform 1
1305	P42574	12	36	63.2	277	31608	6.5	Caspase-3
1306	Q6IBT1	12	94	62.5	277	29937	7.7	Proteasome subunit beta type
1306	Q99436	12	94	62.5	277	29965	7.7	Proteasome subunit beta type-7
1306	Q86U62	12	94	62.5	277	30018	8	Proteasome subunit beta type
1307	P36543	12	32	62.4	226	26145	8	V-type proton ATPase subunit E 1
1308	Q9UBW8	12	36	61.8	275	30277	8.2	COP9 signalosome complex subunit 7a
1309	UPI000020A341	12	122	60.9	197	22310	6.1	retinol-binding protein 1 isoform a
1310	P28074	12	63	59.7	263	28480	6.9	Proteasome subunit beta type-5
1311	P52565	12	178	58.3	204	23207	5.1	Rho GDP-dissociation inhibitor 1
1312	O76003	12	42	58.2	335	37432	5.4	Glutaredoxin-3
1313	A8K4W5	12	33	57.9	397	41379	6.9	cDNA FLJ76813, highly similar to sapiens acetyl-Coenzyme A acetyltransferase 2 (acetoacetyl Coenzyme A thiolase), mRNA
1313	Q9BWD1	12	33	57.9	397	41351	6.9	Acetyl-CoA acetyltransferase, cytosolic
1313	Q59GW6	12	33	56.9	404	42136	7.4	Acetyl-CoA acetyltransferase, cytosolic variant (Fragment)
1314	B2R514	12	35	57.9	297	33576	6	cDNA, FLJ92300, sapiens COP9 subunit 6 (MOV34 log, 34 kD) (COPS6), mRNA
1314	E7EM64	12	35	52.8	326	36064	5.7	Uncharacterized protein
1314	Q7L5N1	12	35	52.6	327	36163	5.7	COP9 signalosome complex subunit 6
1315	P52597	12	135	57.6	415	45672	5.6	Heterogeneous nuclear ribonucleoprotein F
1316	B4DMT5	12	55	57.3	307	33240	5.6	cDNA FLJ52696, highly similar to Eukaryotic translation initiation factor 3 subunit 5
1316	O00303	12	55	49.3	357	37564	5.4	Eukaryotic translation initiation factor 3 subunit F
1317	Q9H3H0	12	26	56.7	247	28275	9.4	Vacuolar ATP synthase subunit D log
1317	Q9Y5K8	12	26	56.7	247	28263	9.4	V-type proton ATPase subunit D
1318	Q59EU6	12	50	54.7	265	29774	6.2	EF hand domain family, member D1 variant (Fragment)
1319	Q9Y5K5-2	12	33	55.1	316	36079	5.2	Isoform 2 of Ubiquitin carboxyl-terminal hydrolase isozyme L5
1319	E9PGR0	12	33	54.9	317	36419	5.3	Uncharacterized protein
1319	E7EQ55	12	33	54.5	319	36500	5.9	Uncharacterized protein
1319	Q9Y5K5-4	12	33	53.4	326	37353	5.3	Isoform 4 of Ubiquitin carboxyl-terminal hydrolase isozyme L5
1319	Q9Y5K5-3	12	33	53	328	37478	5.4	Isoform 3 of Ubiquitin carboxyl-terminal hydrolase isozyme L5
1319	Q9Y5K5	12	33	52.9	329	37607	5.3	Ubiquitin carboxyl-terminal hydrolase isozyme L5
1319	Q5LJA5	12	33	49	355	40423	5.3	Ubiquitin carboxyl-terminal hydrolase L5
1319	Q5LJA9	12	33	47.3	368	41695	5.5	Ubiquitin carboxyl-terminal hydrolase L5 (Fragment)
1320	O75822	12	29	52.3	258	29062	4.8	Eukaryotic translation initiation factor 3 subunit J
1321	P78406	12	23	51.4	368	40968	7.8	mRNA export factor
1322	B2RDV2	12	18	50.9	411	43518	5.9	cDNA, FLJ96778, highly similar to sapiens aminopeptidase-like 1 (NPEPL1), mRNA
1323	P46777	12	96	50.5	297	34363	9.7	60S ribosomal protein L5
1324	Q9BRF8	12	23	50.3	314	35548	6.2	Calcineurin-like phosphoesterase domain-containing protein 1
1325	Q86WR0	12	37	49.5	208	24479	6.8	Coiled-coil domain-containing protein 25
1326	Q0QF37	12	29	54.8	305	31969	7.9	Malate dehydrogenase (Fragment)
1326	P40926	12	29	49.4	338	35503	8.7	Malate dehydrogenase, mitochondrial
1326	Q6FHZ0	12	29	49.4	338	35559	8.7	Malate dehydrogenase
1327	Q6FI81	12	38	49.4	312	33582	5.6	Anamorsin
1328	B4DDT7	12	27	49.2	299	34084	8.5	cDNA FLJ58311, highly similar to 5'-AMP-activated protein kinase subunit gamma-1
1328	P54619	12	27	44.4	331	37579	6.9	5'-AMP-activated protein kinase subunit gamma-1

Table S1. Proteins identified in the OVCAR-3 tumor supernatant by two or more unique peptides.

Protein or Protein Group	Accession Number	Unique Peptide Count	Spectrum Count	Sequence Coverage (%)	Length	MolWt	pI	Descriptive Name
1329	P55145	12	31	48.6	179	20257	8.4	Mesencephalic astrocyte-derived neurotrophic factor
1329	UPI000013D9C8	12	31	37.2	234	26903	10.1	UPI000013D9C8 UniRef100 entry
1330	O43813	12	19	48.4	399	45283	7.8	LanC-like protein 1
1330	Q6FHH6	12	19	48.4	399	45267	7.8	LANCL1 protein (Fragment)
1331	Q16539	12	24	48.1	360	41293	5.8	Mitogen-activated protein kinase 14
1332	Q05048	12	22	47.8	431	48358	6.6	Cleavage stimulation factor subunit 1
1333	Q86UY0	12	34	51.9	360	40369	5.8	TXNDC5 protein
1333	Q658S9	12	34	47.7	392	43505	5.5	Putative uncharacterized protein DKFZp666I134 (Fragment)
1333	Q8NBS9	12	34	43.3	432	47629	6	Thioredoxin domain-containing protein 5
1334	P15880	12	95	47.1	293	31324	10.2	40S ribosomal protein S2
1335	Q9H1E3	12	82	46.9	243	27296	5.1	Nuclear ubiquitous casein and cyclin-dependent kinases substrate
1336	P17612-2	12	19	47.5	343	39822	8.7	Isoform 2 of cAMP-dependent protein kinase catalytic subunit alpha
1336	P17612	12	19	46.4	351	40590	8.8	cAMP-dependent protein kinase catalytic subunit alpha
1337	Q53F20	12	89	46.2	266	30434	3.9	Acidic (Leucine-rich) nuclear phosphoprotein 32 family, member E variant (Fragment)
1337	Q9BTT0	12	89	45.9	268	30692	3.8	Acidic leucine-rich nuclear phosphoprotein 32 family member E
1338	Q9UJ70	12	25	46.2	344	37376	6.2	N-acetyl-D-glucosamine kinase
1339	P51570	12	20	49	392	42272	6.5	Galactokinase
1339	Q6ZP37	12	20	48.5	396	42952	7.2	CDNA FLJ26554 fis, clone LNF01773, highly similar to Galactokinase
1339	B4E1G6	12	20	45.5	422	45358	6.7	Galactokinase 1
1340	Q08752	12	28	44.9	370	40764	7.2	Peptidyl-prolyl cis-trans isomerase D
1340	Q6FGM6	12	28	44.9	370	40773	7.2	PPID protein
1341	P25788-2	12	36	46	248	27647	5.3	Isoform 2 of Proteasome subunit alpha type-3
1341	P25788	12	36	44.7	255	28433	5.3	Proteasome subunit alpha type-3
1342	A5GFU6	12	29	44.1	379	44179	6.3	GNAS complex locus
1342	P63092-2	12	29	43.9	380	44266	6.3	Isoform Gnas-2 of Guanine nucleotide-binding protein G(s) subunit alpha isoforms short
1342	P63092	12	29	42.4	394	45665	5.8	Guanine nucleotide-binding protein G(s) subunit alpha isoforms short
1342	A6NI00	12	29	42.3	395	45752	5.8	Uncharacterized protein
1342	Q5JWF2-2	12	29	16.3	1023	109626	5.1	Isoform XLas-2 of Guanine nucleotide-binding protein G(s) subunit alpha isoforms XLas
1342	Q5JWF2	12	29	16.1	1037	111024	5	Guanine nucleotide-binding protein G(s) subunit alpha isoforms XLas
1343	Q13148	12	53	44	414	44740	6.2	TAR DNA-binding protein 43
1344	O75821	12	44	43.1	320	35611	6.1	Eukaryotic translation initiation factor 3 subunit G
1345	Q6DKJ4	12	26	42.3	435	48392	5	Nucleoredoxin
1346	Q8N806	12	21	41.4	425	47999	4.8	Putative E3 ubiquitin-protein ligase UBR7
1347	Q99447	12	22	40.6	389	43835	6.9	Ethanolamine-phosphate cytidylyltransferase
1347	UPI0000D4CB10	12	22	38.8	407	45622	6.7	ethanolamine-phosphate cytidylyltransferase isoform 1
1348	Q9BS26	12	22	40.4	406	46971	5.3	Endoplasmic reticulum resident protein 44
1349	Q13867	12	30	39.6	455	52562	6.3	Bleomycin hydrolase
1350	Q9BWF3	12	43	39.6	364	40314	7.1	RNA-binding protein 4
1351	Q13283	12	111	39.3	466	52164	5.5	Ras GTPase-activating protein-binding protein 1
1352	B4DZK0	12	25	39	510	57321	8.5	cDNA FLJ56391, highly similar to Cysteine protease ATG4B (EC 3.4.22.-)
1353	P26196	12	43	38.5	483	54417	8.7	Probable ATP-dependent RNA helicase DDX6
1354	Q9P2T1	12	30	44	348	37874	7.2	GMP reductase 2
1354	Q6PKC0	12	30	37.4	409	45008	8.3	GMP reductase
1355	Q9Y570	12	31	36	386	42315	6	Protein phosphatase methylesterase 1
1356	P35520	12	19	35.4	551	60587	6.6	Cystathionine beta-synthase
1356	P35520-2	12	19	34.5	565	61863	6.4	Isoform 2 of Cystathionine beta-synthase
1357	Q15645	12	27	34.7	432	48551	6.1	Thyroid receptor-interacting protein 13
1358	Q9H993	12	21	34.7	441	51172	5.8	UPF0364 protein C6orf211
1359	Q59F94	12	34	34	600	63740	5.2	Ataxin-1 ubiquitin-like interacting protein variant (Fragment)
1360	Q9NX58	12	42	34	379	43615	9.5	Cell growth-regulating nucleolar protein
1361	Q8WVY7	12	23	32.7	318	36805	6.5	Ubiquitin-like domain-containing CTD phosphatase 1
1362	Q9Y383	12	50	31.1	392	46514	10	Putative RNA-binding protein Luc7-like 2
1363	P10644	12	18	31	381	42982	5.3	cAMP-dependent protein kinase type I-alpha regulatory subunit
1363	Q68DQ4	12	18	31	381	42948	5.3	Putative uncharacterized protein DKFZp779L0468
1364	Q9UK59	12	26	28.5	544	61555	5.5	Lariat debranching enzyme
1365	O95793-2	12	41	33.1	496	54934	9.5	Isoform Short of Double-stranded RNA-binding protein Staufen log 1
1365	Q5JW29	12	41	32.7	502	55635	9.5	Staufen protein T4 variant
1365	A8K622	12	41	28.4	577	63192	9.4	cDNA FLJ75871, highly similar to sapiens staufen, RNA binding protein (STAU), transcript variant T3, mRNA
1365	O95793	12	41	28.4	577	63182	9.4	Double-stranded RNA-binding protein Staufen log 1
1366	Q8WX92	12	22	26.2	580	65697	6.1	Negative elongation factor B
1367	P08621	12	51	26.1	437	51557	9.9	U1 small nuclear ribonucleoprotein 70 kDa
1368	A8K4C2	12	43	24.8	604	66822	8.7	cDNA FLJ75458, highly similar to sapiens sulfhydryl oxidase mRNA
1368	O00391-2	12	43	24.8	604	66861	8.6	Isoform 2 of Sulfhydryl oxidase 1
1368	A8MXT8	12	43	24.8	604	66847	8.6	Uncharacterized protein
1368	O00391	12	43	20.1	747	82578	8.9	Sulfhydryl oxidase 1
1369	Q86YQ0	12	24	24.2	765	84721	8.1	HZGJ
1370	Q8IZ83	12	21	23.6	802	85127	6.8	Aldehyde dehydrogenase family 16 member A1
1371	Q06210-2	12	31	22.9	681	76759	6.9	Isoform 2 of Glucosamine--fructose-6-phosphate aminotransferase [isomerizing] 1
1371	Q06210	12	31	22.3	699	78806	7.1	Glucosamine--fructose-6-phosphate aminotransferase [isomerizing] 1
1372	Q58EY4	12	53	20.2	1105	122911	5.7	SWI/SNF related, matrix associated, actin dependent regulator of chromatin, subfamily c, member 1
1372	Q92922	12	53	20.2	1105	122867	5.8	SWI/SNF complex subunit SMARCC1
1373	Q92854	12	25	20.2	862	96150	8	Semaphorin-4D
1373	Q4LE41	12	25	20	870	97153	8	SEMA4D variant protein (Fragment)
1374	Q86TI2	12	14	19.7	863	98263	6.5	Dipeptidyl peptidase 9
1375	B2RAM6	12	31	17	1056	119132	5.7	cDNA, FLJ95005, highly similar to sapiens kinesin family member 11 (KIF11), mRNA
1375	P52732	12	31	17	1056	119159	5.6	Kinesin-like protein KIF11
1376	Q7Z3J2	12	33	16.8	963	109562	7.2	UPF0505 protein C16orf62
1376	UPI0001E1129C	12	33	15.4	1052	118614	7.8	hypothetical protein LOC57020
1377	Q08379	12	17	16.5	1002	113086	5	Golgin subfamily A member 2
1378	E7EWZ6	12	13	17.2	1002	111131	5.6	Uncharacterized protein
1378	B7Z883	12	13	17.2	1002	111145	5.6	cDNA FLJ51414, highly similar to Integrin alpha-V
1378	A5YM53	12	13	16.4	1048	116105	5.7	ITGAV protein
1378	P06756	12	13	16.4	1048	116038	5.7	Integrin alpha-V
1379	B7ZKT9	12	23	15.6	1174	136516	7.4	KIAA1033 protein
1380	B0AZM4	12	12	15.4	1353	156837	7.1	cDNA, FLJ79464, highly similar to sapiens aquarius (Fragment)
1380	A8K6F0	12	12	14.1	1485	171295	6.3	cDNA FLJ75449, highly similar to sapiens aquarius log (mouse) (AQR), mRNA

Table S1. Proteins identified in the OVCAR-3 tumor supernatant by two or more unique peptides.

Protein or Protein Group	Accession Number	Unique Peptide Count	Spectrum Count	Sequence Coverage (%)	Length	MolWt	pI	Descriptive Name
1380	O60306	12	12	14.1	1485	171294	6.4	Intron-binding protein aquarius
1381	O75339	12	19	12.2	1184	132565	8.4	Cartilage intermediate layer protein 1
1381	UPI0001779559	12	19	12.2	1184	132566	8.3	cartilage intermediate layer protein 1 preproprotein
1382	Q52LW3	12	32	11.6	1261	142063	6.7	Rho GTPase-activating protein 29
1383	P07996	12	28	11	1170	129383	4.9	Thrombospondin-1
1383	Q59E99	12	28	10.5	1225	134849	5	Thrombospondin 1 variant (Fragment)
1384	O14497	12	22	7.3	2285	242042	6.7	AT-rich interactive domain-containing protein 1A
1385	Q14008-2	12	32	7.2	1972	218524	8.1	Isoform 2 of Cytoskeleton-associated protein 5
1385	Q14008	12	32	6.9	2032	225493	7.8	Cytoskeleton-associated protein 5
1385	Q14008-3	12	32	6.9	2039	226256	7.8	Isoform 3 of Cytoskeleton-associated protein 5
1386	Q70CQ2-3	12	22	4.9	3312	377599	5.9	Isoform 3 of Ubiquitin carboxyl-terminal hydrolase 34
1386	C9JV64	12	22	4.9	3311	377471	5.9	Ubiquitin carboxyl-terminal hydrolase
1386	B9ZVR8	12	22	4.8	3394	387027	5.9	Ubiquitin carboxyl-terminal hydrolase
1386	Q70CQ2-2	12	22	4.8	3395	387155	6	Isoform 2 of Ubiquitin carboxyl-terminal hydrolase 34
1386	Q70CQ2	12	22	4.6	3546	404234	5.8	Ubiquitin carboxyl-terminal hydrolase 34
1386	C9JGA8	12	22	4.6	3546	404282	5.9	Ubiquitin carboxyl-terminal hydrolase
1387	P62487	11	46	93.6	172	19294	5.5	DNA-directed RNA polymerase II subunit RPB7 DNA-directed RNA polymerase II subunit RPB7
1388	P84085	11	626	80	180	20530	6.8	ADP-ribosylation factor 5 ADP-ribosylation factor 5
1389	P61086	11	66	74.5	200	22407	5.4	Ubiquitin-conjugating enzyme E2 K Ubiquitin-conjugating enzyme E2 K
1390	Q09028-3	11	76	76.3	410	46158	5.1	Isoform 3 of Histone-binding protein RBBP4
1390	Q09028	11	76	73.6	425	47656	4.9	Histone-binding protein RBBP4 Histone-binding protein RBBP4
1390	Q60972	11	76	73.6	425	47655	4.9	Histone-binding protein RBBP4
1391	P61224	11	150	72.8	184	20825	5.8	Ras-related protein Rap-1b Ras-related protein Rap-1b
1392	Q6PJ77	11	61	70.6	153	16470	5.6	BTF3L4 protein (Fragment)
1392	Q96K17	11	61	68.4	158	17271	6.4	Transcription factor BTF3 log 4 Transcription factor BTF3 log 4
1393	P62491	11	172	68.1	216	24394	6.5	Ras-related protein Rab-11A Ras-related protein Rab-11A
1394	Q53GF5	11	70	67.1	234	25841	8	Proteasome subunit alpha type (Fragment)
1394	P49722	11	70	67.1	234	25926	8.3	Proteasome subunit alpha type-2
1394	P25787	11	70	67.1	234	25899	7.4	Proteasome subunit alpha type-2
1394	P17220	11	70	67.1	234	25927	7.4	Proteasome subunit alpha type-2
1394	E9Q3A4	11	70	63.8	246	27426	8.2	Uncharacterized protein
1394	Q3UWT6	11	70	63.6	247	27510	8.2	Proteasome subunit alpha type
1395	O75934	11	40	63.6	225	26131	5.6	Pre-mRNA-splicing factor SPF27 Pre-mRNA-splicing factor SPF27
1396	P62249	11	159	61	146	16445	10.2	40S ribosomal protein S16 40S ribosomal protein S16
1396	Q5CZY9	11	159	51.7	172	19274	10.2	Rps16 protein
1397	Q4JGY1	11	176	62.6	171	18871	7.6	Cellular nucleic acid binding protein beta variant 1 Cellular nucleic acid binding protein beta variant 1
1397	P53996	11	176	60.1	178	19592	7.6	Cellular nucleic acid-binding protein Cellular nucleic acid-binding protein
1398	P46782	11	96	57.4	204	22876	9.7	40S ribosomal protein S5
1398	Q91V55	11	96	57.4	204	22876	9.7	Ribosomal protein S5
1399	Q8VC94	11	100	61.1	167	19024	9.8	Rpl11 protein Rpl11 protein
1399	Q08ES8	11	100	57.6	177	20094	9.6	Cell growth-inhibiting protein 34
1399	P62913	11	100	57.3	178	20252	9.6	60S ribosomal protein L11 60S ribosomal protein L11
1400	B4DL66	11	24	53	287	32892	6.8	cDNA FLJ56297, highly similar to Rattus norvegicus ubiquitin-conjugating enzyme E2Z (putative) (Ube2z), mRNA
1400	Q9H832	11	24	42.9	354	38210	5.6	Ubiquitin-conjugating enzyme E2 Z
1400	Q3UE37	11	24	42.7	356	38368	5.6	Ubiquitin-conjugating enzyme E2 Z
1401	B2RCX0	11	117	51.3	277	32134	4.2	cDNA, FLJ96345, sapiens SET translocation (myeloid leukemia-associated) (SET),mRNA
1401	Q9EQU5-2	11	117	51.3	277	32105	4.2	Isoform 2 of Protein SET
1401	Q01105-2	11	117	51.3	277	32103	4.2	Isoform 2 of Protein SET
1402	P60891	11	48	48.1	318	34834	7	Ribose-phosphate pyrophosphokinase 1 Ribose-phosphate pyrophosphokinase 1
1402	UPI00001C4EFA	11	48	48.1	318	34824	7	hypothetical protein LOC328099
1402	Q53FW2	11	48	48.1	318	34864	6.8	Ribose-phosphate pyrophosphokinase (Fragment)
1402	Q3TI27	11	48	48.1	318	34865	7	Ribose-phosphate pyrophosphokinase
1403	Q5TCU3	11	31	43.7	284	32815	4.7	Tropomyosin 2 (Beta)
1403	Q61344	11	31	43.7	284	32801	4.7	Beta-tropomyosin
1404	Q07955	11	42	44.4	248	27745	10.4	Serine/arginine-rich splicing factor 1 Serine/arginine-rich splicing factor 1
1404	E9QKI1	11	42	43.5	253	28329	10.1	Uncharacterized protein
1405	P04899	11	20	39.4	355	40451	5.5	Guanine nucleotide-binding protein G(i) subunit alpha-2
1405	Q3TXK7	11	20	39.4	355	40489	5.5	Putative uncharacterized protein
1406	Q80XR5	11	127	38.6	471	53121	9.1	U2 small nuclear ribonucleoprotein auxiliary factor (U2AF) 2, isoform CRA_a U2 small nuclear ribonucleoprotein auxiliary factor (U2AF) 2, isoform CRA_a
1406	P26368	11	127	38.3	475	53501	9.1	Splicing factor U2AF 65 kDa subunit
1406	P26369	11	127	38.3	475	53517	9.1	Splicing factor U2AF 65 kDa subunit
1407	O60884	11	21	38.1	412	45746	6.5	DnaJ log subfamily A member 2
1407	Q9QYJ0	11	21	38.1	412	45746	6.5	DnaJ log subfamily A member 2
1407	Q3TFF0	11	21	38.1	412	45747	6.4	Putative uncharacterized protein
1408	B7Z382	11	17	34.6	532	61441	5.7	cDNA FLJ53335, highly similar to Cytosolic purine 5'-nucleotidase (EC 3.1.3.5)
1408	Q3V1L4	11	17	32.9	560	64809	6.2	Cytosolic purine 5'-nucleotidase
1408	A8K6K2	11	17	32.8	561	64960	6.1	cDNA FLJ75877, highly similar to sapiens 5'-nucleotidase, cytosolic II (NT5C2), mRNA
1408	P49902	11	17	32.8	561	64970	6.1	Cytosolic purine 5'-nucleotidase
1408	E9Q9M1	11	17	31.4	586	67704	6.8	Uncharacterized protein
1409	A8K566	11	13	28.3	501	58791	5.4	cDNA FLJ78246, highly similar to sapiens splicing factor 3a, subunit 3, 60kDa (SF3A3), mRNA
1409	Q9D554	11	13	28.3	501	58842	5.3	Splicing factor 3A subunit 3
1409	Q12874	11	13	28.3	501	58849	5.4	Splicing factor 3A subunit 3
1409	B3KY12	11	13	28.3	501	58777	5.4	cDNA FLJ46581 fis, clone THYMU3043200, highly similar to Splicing factor 3A subunit 3
1410	Q5R914	11	25	25.8	511	57659	6.3	Serine/threonine-protein phosphatase
1410	P63328-2	11	25	25.8	511	57615	6.3	Isoform 2 of Serine/threonine-protein phosphatase 2B catalytic subunit alpha isoform
1410	P63328	11	25	25.3	521	58644	5.9	Serine/threonine-protein phosphatase 2B catalytic subunit alpha isoform
1410	Q08209	11	25	25.3	521	58688	5.9	Serine/threonine-protein phosphatase 2B catalytic subunit alpha isoform
1411	E9PUF2	11	24	42.1	285	31596	5.9	Uncharacterized protein
1411	UPI0001F78A36	11	24	26.5	452	49230	6	UPI0001F78A36 UniRef100 entry
1411	Q9Y2H0-3	11	24	26.5	453	49478	6	Isoform 3 of Disks large-associated protein 4
1411	B1AZP2-3	11	24	26.5	453	49460	6	Isoform 3 of Disks large-associated protein 4
1411	Q3UCF7	11	24	26.3	457	49849	6.2	Putative uncharacterized protein (Fragment)
1411	B7ZNS2	11	24	12.3	975	106170	7.2	Dlgap4 protein
1411	B1AZP2	11	24	12.1	992	108037	7.1	Disks large-associated protein 4
1411	Q9Y2H0-1	11	24	12.1	989	107567	7.3	Isoform 2 of Disks large-associated protein 4

Table S1. Proteins identified in the OVCAR-3 tumor supernatant by two or more unique peptides.

Protein or Protein Group	Accession Number	Unique Peptide Count	Spectrum Count	Sequence Coverage (%)	Length	MolWt	pI	Descriptive Name
1411	Q9Y2H0	11	24	12.1	992	108012	7.1	Disks large-associated protein 4
1411	B1AZP2-2	11	24	12.1	989	107551	7.3	Isoform 2 of Disks large-associated protein 4
1412	P35278	11	56	78.2	216	23412	8.4	Ras-related protein Rab-5C
1413	P68510	11	63	77.6	246	28212	4.9	14-3-3 protein eta
1414	Q00915	11	94	73.3	135	15846	5.2	Retinol-binding protein 1
1415	P97314	11	79	62.7	193	20926	8.6	Cysteine and glycine-rich protein 2
1415	Q62908	11	79	62.7	193	20940	8.6	Cysteine and glycine-rich protein 2
1416	P26516	11	31	61.4	321	36540	6.8	26S proteasome non-ATPase regulatory subunit 7
1417	Q9DBP5	11	45	58.7	196	22165	5.8	UMP-CMP kinase
1418	Q64674	11	20	57.9	302	33995	5.5	Spermidine synthase
1418	Q8BSZ8	11	20	57.9	302	34019	5.6	Putative uncharacterized protein
1419	P00493	11	45	57.8	218	24570	6.7	Hypoxanthine-guanine phosphoribosyltransferase
1419	Q99KF5	11	45	57.8	218	24544	6.7	Hypoxanthine guanine phosphoribosyl transferase 1
1420	Q9DBZ5	11	43	57.8	218	25087	4.9	Eukaryotic translation initiation factor 3 subunit K
1421	D3Z0F5	11	33	56.2	297	33592	6	Uncharacterized protein
1421	O88545	11	33	51.5	324	35880	5.7	COP9 signalosome complex subunit 6
1422	O55234	11	34	49.6	264	28532	7	Proteasome subunit beta type-5
1423	P70195	11	55	48.4	277	29891	8	Proteasome subunit beta type-7
1424	Q80ZP8	11	27	52.1	165	19012	8.1	Armet protein
1424	P0C5H9	11	27	48	179	20388	8.3	Mesencephalic astrocyte-derived neurotrophic factor
1425	P70122	11	24	46.8	250	28781	8.8	Ribosome maturation protein SBDS
1426	E9PXX7	11	21	46.2	344	38532	5.4	Uncharacterized protein
1426	Q91W90	11	21	38.1	417	46416	5.8	Thioredoxin domain-containing protein 5
1427	Q8C845	11	48	45.8	240	26800	5.1	EF hand domain containing 2
1428	Q8BTQ1	11	53	47.2	305	33757	9.6	Putative uncharacterized protein (Fragment)
1428	Q3UFF9	11	53	44.2	326	36150	8.9	Putative uncharacterized protein
1428	Q8BW03	11	53	44.2	326	36153	9	Putative uncharacterized protein
1428	Q8BM84	11	53	44.2	326	36197	9.2	Putative uncharacterized protein
1428	Q78QY3	11	53	44.2	326	36169	9	ELAV (Embryonic lethal, abnormal vision, Drosophila)-like 1 (Hu antigen R), isoform CRA_a
1429	Q61792	11	99	42.6	263	29994	7	LIM and SH3 domain protein 1
1430	UPI0001E6B7EC	11	86	38.9	296	33955	9.5	PREDICTED: 60S ribosomal protein L5-like isoform 2
1430	P47962	11	86	38.7	297	34401	9.8	60S ribosomal protein L5
1430	Q3U850	11	86	38.7	297	34357	9.8	Putative uncharacterized protein
1431	O89053	11	16	37.3	461	50989	6.5	Coronin-1A
1431	Q3U232	11	16	37.3	461	50959	6.5	Putative uncharacterized protein
1432	Q9WUM3	11	19	30.6	484	53912	5.8	Coronin-1B
1433	P80317	11	24	29.9	531	58004	7.1	T-complex protein 1 subunit zeta
1433	Q52KG9	11	24	29.9	531	58076	6.9	Chaperonin containing Tcp1, subunit 6a (Zeta)
1433	Q3TIX8	11	24	29.9	531	58103	6.9	Putative uncharacterized protein
1434	O88487	11	28	30.4	612	68394	5.3	Cytoplasmic dynein 1 intermediate chain 2
1434	D6Q0F7	11	28	30.1	618	69206	5.3	Cytoplasmic dynein intermediate chain 2 isoform 2.5
1434	Q3TPJ8	11	28	29.4	632	70613	5.2	Dynein cytoplasmic 1 intermediate chain 2
1434	A2BFF9	11	28	29.2	638	71424	5.2	Dynein cytoplasmic 1 intermediate chain 2
1434	D6Q0F6	11	28	28.6	650	72801	5.2	Cytoplasmic dynein intermediate chain 2 isoform 2.2
1434	D6Q0F5	11	28	28.4	655	73319	5.2	Cytoplasmic dynein intermediate chain 2 isoform 2.1
1435	Q3TI63	11	35	25.1	522	58999	5.2	Putative uncharacterized protein
1435	Q3U4S1	11	35	25.1	522	59012	5.2	Putative uncharacterized protein
1436	Q3TX27	11	39	24.8	650	72416	5.3	Arginyl aminopeptidase (Aminopeptidase B), isoform CRA_a
1436	Q8VCT3	11	39	24.8	650	72343	5.3	Aminopeptidase B
1437	A2A6U3	11	28	23	565	63773	8.3	Septin 9
1437	Q80UG5-3	11	28	22.6	576	64775	7.5	Isoform 3 of Septin-9
1437	Q80UG5	11	28	22.3	583	65575	8.9	Septin-9
1438	Q99JF8	11	35	22	528	59697	9.1	PC4 and SFRS1-interacting protein
1439	Q5SUR0	11	16	14.4	1337	144629	5.7	Phosphoribosylformylglycinamidine synthase
1440	Q9DBR7	11	47	13.4	1029	114996	5.5	Protein phosphatase 1 regulatory subunit 12A
1441	Q59IW6	11	41	11.5	1179	129538	9.6	Target of Nesh-SH3 variant 5
1442	A2AJY2	11	43	11.2	1345	138292	4.9	Procollagen type XV
1442	O35206	11	43	11	1367	140472	4.9	Collagen alpha-1(XV) chain
1443	B5THE2	11	13	8	1827	208548	5.9	Maltase-glucoamylase
1444	P10599	11	356	91.4	105	11737	4.9	Thioredoxin
1445	P06703	11	294	91.1	90	10180	5.5	Protein S100-A6
1446	P30046	11	128	90.7	118	12712	7.3	D-dopachrome decarboxylase
1447	Q9BVJ7	11	39	85.3	150	16588	8.2	Dual specificity protein phosphatase 23
1448	P15374	11	26	81.3	230	26183	4.9	Ubiquitin carboxyl-terminal hydrolase isozyme L3
1449	Q96EK6	11	63	80.4	184	20749	8	Glucosamine 6-phosphate N-acetyltransferase
1450	Q9ULC4	11	72	80.1	181	20555	8.8	Malignant T cell-amplified sequence 1
1451	P51148	11	57	78.2	216	23483	8.4	Ras-related protein Rab-5C
1452	Q04917	11	49	77.6	246	28219	4.8	14-3-3 protein eta
1453	P09936	11	83	75.3	223	24824	5.5	Ubiquitin carboxyl-terminal hydrolase isozyme L1
1454	Q15102	11	24	75.3	231	25734	6.8	Platelet-activating factor acetylhydrolase IB subunit gamma
1455	P49721	11	53	74.1	201	22836	7	Proteasome subunit beta type-2
1456	Q9H2H8	11	31	72.7	161	18155	6.8	Peptidyl-prolyl cis-trans isomerase-like 3
1456	UPI000188195B	11	31	65	180	20272	7.4	UPI000188195B UniRef100 entry
1457	P31947	11	30	70.2	248	27774	4.7	14-3-3 protein sigma
1458	Q9UL26	11	21	68.6	194	21855	8.2	Ras-related protein Rab-22A
1459	Q8WZA0	11	34	68.4	190	21495	4.9	Protein LZIC
1460	Q8TCD5	11	38	66.2	201	23383	6.6	5'(3')-deoxyribonucleotidase, cytosolic type
1461	P32969	11	57	66.1	192	21863	10	60S ribosomal protein L9
1462	A5YM50	11	83	65.1	218	24517	6.2	RAB11B protein
1462	Q15907	11	83	65.1	218	24488	5.9	Ras-related protein Rab-11B
1463	Q9Y2B0	11	50	64.8	182	20652	4.9	Protein canopy log 2
1464	Q9BY32	11	40	63.4	194	21446	5.6	Inosine triphosphate pyrophosphatase
1465	Q9UBP6	11	18	63.4	276	31471	7.6	tRNA (guanine-N(7)-)-methyltransferase
1466	Q9UK41	11	25	61.1	221	25425	5.5	Vacuolar protein sorting-associated protein 28 log
1467	Q13257	11	42	61	205	23510	5.1	Mitotic spindle assembly checkpoint protein MAD2A
1468	Q96EI5	11	26	59.5	215	24647	5.2	Transcription elongation factor A protein-like 4
1468	Q96EI5-2	11	26	35.8	358	40578	7.4	Isoform 2 of Transcription elongation factor A protein-like 4
1469	P32119	11	51	58.6	198	21892	6	Peroxiredoxin-2
1470	P24941	11	19	56	298	33930	8.7	Cyclin-dependent kinase 2
1471	Q13868	11	33	56	293	32789	7.5	Exosome complex exonuclease RRP4
1472	Q00169	11	28	55.6	270	31806	6.5	Phosphatidylinositol transfer protein alpha isoform
1473	Q8NCW5	11	41	59	288	31675	7.7	Apolipoprotein A-I-binding protein
1473	B4DP80	11	41	55.4	307	33628	8.7	cDNA FLJ56357, highly similar to sapiens apolipoprotein A-I binding protein (APOA1BP), mRNA
1474	Q9BVM2	11	18	54.2	203	23240	9	Protein DPCD
1475	Q86V81	11	78	54.1	257	26888	11.2	THO complex subunit 4
1476	P30040	11	24	54	261	28993	7.3	Endoplasmic reticulum resident protein 29
1477	A6NDG6	11	36	53.9	321	34006	6.1	Phosphoglycolate phosphatase
1478	P45973	11	69	53.4	191	22225	5.9	Chromobox protein log 5
1479	Q53FP0	11	44	52.5	261	29916	7.4	Pyridoxine 5'-phosphate oxidase variant (Fragment)
1479	Q9NVS9	11	44	52.5	261	29988	7.1	Pyridoxine-5'-phosphate oxidase
1480	P13716	11	15	52.4	330	36295	6.8	Delta-aminolevulinic acid dehydratase
1481	Q9P287	11	28	51.6	314	35979	4.6	BRCA2 and CDKN1A-interacting protein

Table S1. Proteins identified in the OVCAR-3 tumor supernatant by two or more unique peptides.

Protein or Protein Group	Accession Number	Unique Peptide Count	Spectrum Count	Sequence Coverage (%)	Length	MolWt	pI	Descriptive Name
1482	Q8N8N7	11	28	51	351	38499	5.4	Prostaglandin reductase 2
1483	B3KNG6	11	18	50.5	315	37107	4.6	cDNA FLJ14554 fis, clone NT2RM2001839, highly similar to Calumenin
1483	D6QS48	11	18	49.2	323	38079	4.6	Calumenin isoform 4
1484	Q9NZL4	11	29	50.3	362	39474	5.2	Hsp70-binding protein 1
1485	A6NMH6	11	18	50	430	49916	6.2	Uncharacterized protein
1485	UPI000020C588	11	18	48.6	442	51178	6.2	septin-8 isoform c
1485	Q92599	11	18	44.5	483	55756	6.3	Septin-8
1486	P67936	11	38	49.6	248	28522	4.7	Tropomyosin alpha-4 chain
1487	Q6FHU0	11	30	49.6	272	29696	5.8	PSMB8 protein (Fragment)
1487	P28062-2	11	30	49.6	272	29770	5.8	Isoform 2 of Proteasome subunit beta type-8
1487	P28062	11	30	48.9	276	30354	7.8	Proteasome subunit beta type-8
1487	Q5QNR8	11	30	48.9	276	30354	7.4	Proteasome subunit beta type
1488	Q9BUF5	11	17	48.7	446	49857	4.9	Tubulin beta-6 chain
1489	A8MUD9	11	63	48.1	208	24433	10.7	Uncharacterized protein
1489	P18124	11	63	40.3	248	29226	10.7	60S ribosomal protein L7
1489	UPI00015E0367	11	63	36.8	272	32275	10.8	60S ribosomal protein L7.
1490	B7Z8S7	11	24	47.4	378	41263	6.2	Secernin 2, isoform CRA_b
1490	Q9BU04	11	24	44.6	401	43530	6.2	Ses2 protein (Fragment)
1491	Q9NR45	11	30	47.4	359	40308	6.7	Sialic acid synthase
1492	Q15293	11	30	47.1	331	38890	5	Reticulocalbin-1
1493	A8K718	11	26	47	315	35116	6.8	cDNA FLJ77502
1493	Q9H0W9	11	26	47	315	35117	6.7	Ester hydrolase C11orf54
1494	P49903	11	24	46.4	392	42911	6	Selenide, water dikinase 1
1495	Q9Y3A5	11	25	46.4	250	28764	8.7	Ribosome maturation protein SBDS
1496	Q13177	11	18	45.4	524	58043	6	Serine/threonine-protein kinase PAK 2
1497	O00233	11	29	44.8	223	24682	7	26S proteasome non-ATPase regulatory subunit 9
1498	Q96IJ6	11	22	44.8	420	46291	7.2	Mannose-1-phosphate guanyltransferase alpha
1499	B2R7R5	11	19	44.7	369	40884	7.4	cDNA, FLJ93570, highly similar to sapiens phosphoribosyl pyrophosphate synthetase-associated protein 2 (PRPSAP2), mRNA
1499	O60256	11	19	44.7	369	40926	7.4	Phosphoribosyl pyrophosphate synthase-associated protein 2
1500	A8K5B0	11	14	44.6	379	42573	8.2	cDNA FLJ75682, highly similar to sapiens armadillo repeat containing, X-linked 3 (ARMCX3), transcript variant 1, mRNA
1500	Q9UH62	11	14	44.6	379	42501	8.4	Armadillo repeat-containing X-linked protein 3
1501	P42025	11	25	43.4	376	42293	6.4	Beta-centractin
1502	O60547	11	18	43.3	372	41950	7.3	GDP-mannose 4,6 dehydratase
1503	P09012	11	67	42.6	282	31280	9.8	U1 small nuclear ribonucleoprotein A
1504	Q99729-3	11	124	42.5	285	30588	7.9	Isoform 3 of Heterogeneous nuclear ribonucleoprotein A/B
1505	B2RD19	11	21	42.4	309	34352	7.3	cDNA, FLJ96419, highly similar to sapiens fructosamine-3-kinase-related protein (FN3KRP), mRNA
1505	Q9HA64	11	21	42.4	309	34412	7.3	Ketosamine-3-kinase
1506	O00154-6	11	27	48.9	329	36568	7.8	Isoform 6 of Cytosolic acyl coenzyme A thioester hydrolase
1506	O00154-4	11	27	47.6	338	37420	7.3	Isoform 4 of Cytosolic acyl coenzyme A thioester hydrolase
1506	O00154	11	27	42.4	380	41796	8.5	Cytosolic acyl coenzyme A thioester hydrolase
1507	B4E363	11	20	42.3	477	54158	7.8	cDNA FLJ50378, highly similar to Phenylalanyl-tRNA synthetase alpha chain (EC 6.1.1.20)
1507	Q9Y285	11	20	39.8	508	57564	7.8	Phenylalanyl-tRNA synthetase alpha chain
1508	O75937	11	32	42.3	253	29842	9.1	DnaJ log subfamily C member 8
1509	Q9NQ48	11	18	42.1	299	34592	5.4	Leucine zipper transcription factor-like protein 1
1510	B5BUC0	11	21	41.9	420	46698	8.9	Glycogen synthase kinase-3 beta (Fragment)
1510	P49841	11	21	41.9	420	46744	8.8	Glycogen synthase kinase-3 beta
1510	P49841-2	11	21	40.6	433	48034	8.8	Isoform 2 of Glycogen synthase kinase-3 beta
1511	Q86TY5	11	138	85.1	121	13897	9.1	Full-length cDNA clone CS0DI041YE05 of Placenta of sapiens (human)
1511	P17931	11	138	41.2	250	26152	8.5	Galectin-3
1511	Q59FR8	11	138	39.9	258	27118	8.4	LGALS3 protein variant (Fragment)
1512	Q9NZ32	11	18	41.2	417	46307	7.4	Actin-related protein 10
1513	Q86TX2	11	15	41.1	421	46277	7.3	Acyl-coenzyme A thioesterase 1
1514	B2RAW8	11	16	40.7	403	46103	7.8	cDNA, FLJ95164, highly similar to sapiens SEC14-like 2 (S. cerevisiae) (SEC14L2), mRNA
1514	O76054	11	16	40.7	403	46145	7.8	SEC14-like protein 2
1515	Q8TCE6-2	11	13	41.3	349	39799	6.8	Isoform 2 of Protein FAM45A
1515	Q8TCE6	11	13	40.3	357	40513	6.6	Protein FAM45A
1516	UPI0000E20EFA	11	131	40.2	291	33141	9.3	RPS10-NUDT3 protein
1517	Q92890	11	44	44.6	307	34500	6.7	Ubiquitin fusion degradation protein 1 log
1517	C9JS35	11	44	39.9	343	38688	6.7	Uncharacterized protein
1517	Q92890-1	11	44	39.9	343	38725	6.3	Isoform Long of Ubiquitin fusion degradation protein 1 log
1518	P55212	11	34	39.9	293	33310	6.9	Caspase-6
1519	Q99933-3	11	26	49.3	274	31117	5.1	Isoform 3 of BAG family molecular chaperone regulator 1
1519	Q99933	11	26	39.1	345	38779	7.8	BAG family molecular chaperone regulator 1
1520	A8K9A5	11	17	39	313	35815	7.2	Thymidylate synthase
1520	Q53FB7	11	17	39	313	35717	6.8	Thymidylate synthase (Fragment)
1520	P04818	11	17	39	313	35716	7	Thymidylate synthase
1521	Q13011	11	30	39	328	35816	8	Delta(3,5)-Delta(2,4)-dienoyl-CoA isomerase, mitochondrial
1522	Q8N335	11	22	39	351	38419	7	Glycerol-3-phosphate dehydrogenase 1-like protein
1523	B3KSG0	11	30	47.3	408	44772	5.8	cDNA FLJ36142 fis, clone TESTI2025006, highly similar to sapiens myo-inositol 1-phosphate synthase A1 (ISYNA1), mRNA
1523	Q9NPH2-2	11	30	44.9	430	47146	5.4	Isoform 2 of Inositol-3-phosphate synthase 1
1523	B3KRT1	11	30	38.3	504	55155	5	cDNA FLJ34860 fis, clone NT2NE2012790, highly similar to sapiens myo-inositol 1-phosphate synthase A1 (ISYNA1), mRNA
1523	UPI000013C7F4	11	30	38.3	504	55136	5	inositol-3-phosphate synthase 1 isoform 2
1523	Q9NPH2	11	30	34.6	558	61068	5.7	Inositol-3-phosphate synthase 1
1524	A8K702	11	25	38.2	484	52820	5.1	cDNA FLJ78285, highly similar to sapiens gasdermin domain containing 1, mRNA
1524	P57764	11	25	38.2	484	52801	5.1	Gasdermin-D
1524	UPI0000E5AF91	11	25	34.8	532	57735	5.4	UPI0000E5AF91 UniRef100 entry
1525	Q7Z2Q5	11	23	39.2	429	46405	6.6	C-terminal binding protein 1
1525	Q13363	11	23	38.2	440	47535	6.8	C-terminal-binding protein 1
1526	Q9NXA8-2	11	15	38.1	299	32674	8.9	Isoform 2 of NAD-dependent deacetylase sirtuin-5
1526	Q9NXA8	11	15	36.8	310	33881	8.5	NAD-dependent deacetylase sirtuin-5
1527	Q9BY67-2	11	35	41.7	333	36915	5.1	Isoform 2 of Cell adhesion molecule 1
1527	UPI0001AE6C86	11	35	37.3	373	41667	5	UPI0001AE6C86 UniRef100 entry
1527	A0A4Z1	11	35	35.9	387	42737	5.7	CADM1 protein
1527	UPI00001FA3EC	11	35	33.6	414	45624	5.1	cell adhesion molecule 1 isoform 2
1527	A4FVB5	11	35	33.5	415	45780	5.1	CADM1 protein (Fragment)
1527	Q9BY67	11	35	31.4	442	48509	5.1	Cell adhesion molecule 1
1528	A8K6V7	11	44	35.4	449	50816	5.4	cDNA FLJ76053, highly similar to sapiens Ras-GTPase activating protein SH3 domain-binding protein 2 (G3BP2), transcript variant 3, mRNA
1528	Q9UN86-2	11	44	35.4	449	50817	5.4	Isoform B of Ras GTPase-activating protein-binding protein 2
1528	Q9UN86	11	44	33	482	54121	5.6	Ras GTPase-activating protein-binding protein 2
1529	Q4VC03	11	23	35.3	660	72391	9.3	PABPC4 protein

Table S1. Proteins identified in the OVCAR-3 tumor supernatant by two or more unique peptides.

Protein or Protein Group	Accession Number	Unique Peptide Count	Spectrum Count	Sequence Coverage (%)	Length	MolWt	pI	Descriptive Name
1529	Q6IQ30	11	23	35.3	660	72361	9.3	PABPC4 protein
1530	Q96HE7	11	15	35.3	468	54393	5.7	ERO1-like protein alpha
1530	UPI0000407C2B	11	15	35.3	468	54404	5.9	ERO1-like protein alpha precursor (EC 1.8.4.-) (ERO1-Lalpha) (Oxidoreductin-1-Lalpha) (Endoplasmic oxidoreductin-1-like protein) (ERO1-L).
1531	Q99829	11	32	33.9	537	59059	5.8	Copine-1
1532	A8K168	11	25	33.6	572	64136	6.1	Malic enzyme
1532	P48163	11	25	33.6	572	64150	6.1	NADP-dependent malic enzyme
1533	B4DJQ5	11	48	33.1	535	60134	4.4	cDNA FLJ59211, highly similar to Glucosidase 2 subunit beta
1533	E7EQZ9	11	48	33.1	535	60192	4.4	Uncharacterized protein
1534	P25685	11	25	32.6	340	38044	8.6	DnaJ log subfamily B member 1
1534	Q6FI51	11	25	32.6	340	38010	8.6	DNAJB1 protein
1535	Q9BPW0	11	13	33.5	484	55096	6.2	Serine/threonine-protein phosphatase (Fragment)
1535	P53041	11	13	32.5	499	56879	6.3	Serine/threonine-protein phosphatase 5
1536	B2RCS5	11	40	31.5	894	103920	5.5	Actinin, alpha 2, isoform CRA_b
1536	P35609	11	40	31.5	894	103854	5.5	Alpha-actinin-2
1537	P52306	11	20	31.5	607	66317	5.3	Rap1 GTPase-GDP dissociation stimulator 1
1537	UPI000049DFE5	11	20	31.5	607	66317	5.3	rap1 GTPase-GDP dissociation stimulator 1 isoform 2
1537	UPI000004A01F	11	20	31.4	608	66388	5.3	rap1 GTPase-GDP dissociation stimulator 1 isoform 1
1537	Q4QQI8	11	20	31.4	608	66316	5.4	RAP1, GTP-GDP dissociation stimulator 1
1538	Q06203	11	16	30.8	517	57399	6.8	Amidophosphoribosyltransferase
1538	Q53H22	11	16	30.8	517	57385	6.8	Amidophosphoribosyltransferase (Fragment)
1539	Q86X02	11	16	30.3	465	53010	6	Cerebellar degeneration-related protein 2-like
1540	P30154	11	41	29.5	601	66214	4.9	Serine/threonine-protein phosphatase 2A 65 kDa regulatory subunit A beta isoform
1540	P30154-2	11	41	26.5	667	73585	5.1	Isoform 2 of Serine/threonine-protein phosphatase 2A 65 kDa regulatory subunit A beta isoform
1541	Q6DD88	11	28	27.7	541	60542	5.7	Atlastin-3
1542	B3KUB9	11	26	27.5	527	62034	6	SWAP-70 protein, isoform CRA_b
1543	Q03252	11	42	27.5	600	67689	5.3	Lamin-B2
1544	Q69YN2	11	19	25.1	538	60619	7.2	CWF19-like protein 1
1545	O75475	11	37	24.3	530	60103	9.1	PC4 and SFRS1-interacting protein
1546	A8K9G0	11	29	23.5	689	75633	8.3	cDNA FLJ78634, highly similar to sapiens solute carrier family 34 (sodium phosphate), member 2 (SLC34A2), mRNA
1546	O95436-2	11	29	23.5	689	75657	8.2	Isoform 2 of Sodium-dependent phosphate transport protein 2B
1547	B2RN19	11	14	22	509	58256	8.6	TRM5 tRNA methyltransferase 5 log (S. cerevisiae)
1547	Q32P41	11	14	22	509	58246	8.6	tRNA (guanine-N(1)-)-methyltransferase
1548	Q6IN85-2	11	43	21.8	820	93896	4.9	Isoform 2 of Serine/threonine-protein phosphatase 4 regulatory subunit 3A
1548	Q6IN85	11	43	21.5	833	95368	4.9	Serine/threonine-protein phosphatase 4 regulatory subunit 3A
1549	Q96BP3	11	13	20.9	646	73575	7.1	Peptidylprolyl isomerase domain and WD repeat-containing protein 1
1550	UPI0001AE6E02	11	38	17.6	841	93626	5.3	Actin filament-associated protein 1-like 2.
1551	P10253	11	25	17.5	952	105324	6	Lysosomal alpha-glucosidase
1552	A8K923	11	20	15.9	933	105222	4.8	cDNA FLJ78690, highly similar to sapiens protein phosphatase 4, regulatory subunit 1 (PPP4R1), mRNA
1552	Q8TF05-2	11	20	15.9	933	105195	4.8	Isoform 2 of Serine/threonine-protein phosphatase 4 regulatory subunit 1
1552	Q8TF05	11	20	15.6	950	107004	4.8	Serine/threonine-protein phosphatase 4 regulatory subunit 1
1553	UPI0000EE3A32	11	15	15.6	1235	140288	5.5	UPI0000EE3A32 UniRef100 entry
1553	C9JIP0	11	15	14.7	1316	149001	5.6	Uncharacterized protein
1553	Q12769	11	15	13.4	1436	162121	5.5	Nuclear pore complex protein Nup160
1554	Q6WCQ1	11	24	14.6	1025	116533	6.2	Myosin phosphatase Rho-interacting protein
1554	B9EGI2	11	24	14.5	1037	118047	6.4	Myosin phosphatase Rho interacting protein
1554	Q6WCQ1-2	11	24	14.5	1038	118103	6.4	Isoform 2 of Myosin phosphatase Rho-interacting protein
1555	Q9P2B2	11	11	14.3	879	98556	6.6	Prostaglandin F2 receptor negative regulator
1556	B4DKA8	11	14	15.5	1012	115080	8.5	Ubiquitin carboxyl-terminal hydrolase
1556	A8K8N5	11	14	14	1118	127493	8.6	Ubiquitin carboxyl-terminal hydrolase
1556	P40818	11	14	14	1118	127523	8.5	Ubiquitin carboxyl-terminal hydrolase 8
1557	A8K6X3	11	36	13.6	1032	117471	9.3	cDNA FLJ78679, highly similar to sapiens DEAD (Asp-Glu-Ala-Asp) box polypeptide 46 (DDX46), mRNA
1558	P55011-3	11	26	13.4	1196	129679	6.5	Isoform 2 of Solute carrier family 12 member 2
1558	P55011	11	26	13.2	1212	131447	6.4	Solute carrier family 12 member 2
1559	A0JP11	11	12	12.2	1358	153158	7.2	Phosphoinositide-3-kinase, regulatory subunit 4
1559	Q99570	11	12	12.2	1358	153103	7.2	Phosphoinositide 3-kinase regulatory subunit 4
1559	B2RAJ6	11	12	12.2	1358	153191	7.2	cDNA, FLJ94957, highly similar to sapiens phosphoinositide-3-kinase, regulatory subunit 4, p150 (PIK3R4), mRNA
1560	Q59EC0	11	22	11.7	1244	137833	8.5	Adenosine deaminase, RNA-specific isoform ADAR-a variant (Fragment)
1561	A2A284	11	12	11.5	1291	148660	6	Phospholipase C, gamma 1
1561	P19174	11	12	11.5	1290	148532	6	1-phosphatidylinositol-4,5-bisphosphate phosphodiesterase gamma-1
1561	Q4LE43	11	12	10.5	1412	161302	7.2	PLCG1 variant protein (Fragment)
1562	Q10570	11	15	10.6	1443	160883	6.4	Cleavage and polyadenylation specificity factor subunit 1
1563	Q07157-2	11	47	10.7	1668	186965	6.8	Isoform Short of Tight junction protein ZO-1
1563	UPI0000E59B99	11	47	10.6	1692	189674	6.7	Tight junction protein ZO-1 (Zonula occludens 1 protein) (Zona occludens 1 protein) (Tight junction protein 1).
1563	UPI0001AE689D	11	47	10.4	1727	193249	6.8	Tight junction protein ZO-1 (Zonula occludens 1 protein) (Zona occludens 1 protein) (Tight junction protein 1).
1563	A9CQZ8	11	47	10.2	1761	196814	6.6	Tight junction protein ZO-1 (Fragment)
1563	Q07157	11	47	10.2	1748	195457	6.7	Tight junction protein ZO-1
1564	Q5K651	11	14	10.2	1589	184280	7.8	Sterile alpha motif domain-containing protein 9
1565	E1P614	11	14	8.2	1918	220246	7.3	Zinc finger, NFX1-type containing 1, isoform CRA_b
1565	Q9P2E3	11	14	8.2	1918	220225	7.3	NFX1-type zinc finger-containing protein 1
1566	A6NHR9	11	14	6.8	2005	226372	7.3	Structural maintenance of chromosomes flexible hinge domain-containing protein 1
1567	Q6UVK1	11	11	6.5	2322	250534	5.5	Chondroitin sulfate proteoglycan 4
1568	P49454	11	23	3.9	3210	367765	5.1	Centromere protein F
1569	O95714	11	12	3.1	4834	527234	6.3	E3 ubiquitin-protein ligase HERC2
1570	O14950	10	133	79.7	172	19779	4.8	Myosin regulatory light chain 12B Myosin regulatory light chain 12B
1571	P29762	10	69	78.1	137	15566	5.4	Cellular retinoic acid-binding protein 1 Cellular retinoic acid-binding protein 1
1572	P63279	10	86	70.3	158	18007	8.7	SUMO-conjugating enzyme UBC9 SUMO-conjugating enzyme UBC9
1573	P63241	10	427	68.8	154	16832	5.2	Eukaryotic translation initiation factor 5A-1 Eukaryotic translation initiation factor 5A-1
1573	P63241-2	10	427	57.6	184	20170	7	Isoform 2 of Eukaryotic translation initiation factor 5A-1
1574	P61326	10	188	68.5	146	17164	6.1	Protein mago nashi log Protein mago nashi log
1574	Q9CQL1	10	188	68.5	146	17092	6.4	Protein mago nashi log 1-related
1574	Q96A72	10	188	67.6	148	17276	6.4	Protein mago nashi log 2
1575	P62166	10	24	67.4	190	21879	4.8	Neuronal calcium sensor 1 Neuronal calcium sensor 1

Table S1. Proteins identified in the OVCAR-3 tumor supernatant by two or more unique peptides.

Protein or Protein Group	Accession Number	Unique Peptide Count	Spectrum Count	Sequence Coverage (%)	Length	MolWt	pI	Descriptive Name
1576	P28066	10	172	66	241	26411	4.8	Proteasome subunit alpha type-5 Proteasome subunit alpha type-5
1576	Q3TUX3	10	172	66	241	26397	4.8	Proteasome subunit alpha type
1577	P25789	10	52	62.1	261	29484	7.7	Proteasome subunit alpha type-4
1577	Q9R1P0	10	52	62.1	261	29471	7.7	Proteasome subunit alpha type-4
1578	P61601	10	35	59.1	193	22245	5.4	Neurocalcin-delta Neurocalcin-delta
1579	P61586	10	267	56	193	21768	6.1	Transforming protein RhoA
1579	Q9QUI0	10	267	56	193	21782	6.1	Transforming protein RhoA
1580	P61026	10	58	54	200	22541	8.4	Ras-related protein Rab-10 Ras-related protein Rab-10
1581	Q861U5	10	204	61.8	131	15398	10.4	Similar to 40S ribosomal protein S18 (Fragment)
1581	P62269	10	204	53.3	152	17719	11	40S ribosomal protein S18 40S ribosomal protein S18
1581	Q5SVZ5	10	204	53.3	152	17749	10.9	MCG116671
1582	O43447	10	86	52.5	177	19208	8.1	Peptidyl-prolyl cis-trans isomerase H Peptidyl-prolyl cis-trans isomerase H
1583	D2HUN9	10	81	51.9	208	24173	10.3	40S ribosomal protein S8 (Fragment)
1583	UPI00001C4753	10	81	51.9	208	24152	10.2	UPI00001C4753 UniRef100 entry
1583	P62241	10	81	51.9	208	24205	10.3	40S ribosomal protein S8 40S ribosomal protein S8
1584	O00743	10	29	48.2	305	35144	5.7	Serine/threonine-protein phosphatase 6 catalytic subunit
1584	Q9CQR6	10	29	48.2	305	35159	5.7	Serine/threonine-protein phosphatase 6 catalytic subunit
1585	P61254	10	95	46.2	145	17258	10.6	60S ribosomal protein L26 60S ribosomal protein L26
1585	Q6IBH6	10	95	46.2	145	17244	10.6	RPL26 protein
1586	B2R6K4	10	28	44.4	340	37301	6.2	cDNA, FLJ92996, highly similar to sapiens guanine nucleotide binding protein (G protein), beta polypeptide 1 (GNB1), mRNA
1586	P62873	10	28	44.4	340	37377	6	Guanine nucleotide-binding protein G(I)/G(S)/G(T) subunit beta-1 Guanine nucleotide-binding protein G(I)/G(S)/G(T) subunit beta-1
1587	P61962	10	46	38.3	342	38926	5.5	DDB1- and CUL4-associated factor 7 DDB1- and CUL4-associated factor 7
1588	O14979-3	10	204	37.3	244	27191	8.6	Isoform 3 of Heterogeneous nuclear ribonucleoprotein D-like
1588	B4DTA2	10	204	33.6	271	30214	8.7	cDNA FLJ60148, highly similar to sapiens heterogeneous nuclear ribonucleoprotein D-like (HNRPDL), transcript variant 2, mRNA
1588	Q9Z130	10	204	30.2	301	33559	7.3	Heterogeneous nuclear ribonucleoprotein D-like
1588	O14979-2	10	204	30.2	301	33589	7.3	Isoform 2 of Heterogeneous nuclear ribonucleoprotein D-like
1588	UPI00015AA288	10	204	28.2	323	35648	7.8	UPI00015AA288 UniRef100 entry
1588	O14979	10	204	21.7	420	46437	9.6	Heterogeneous nuclear ribonucleoprotein D-like
1588	D3YTQ3	10	204	21.7	420	46270	9.6	Uncharacterized protein
1589	A8K4L9	10	28	33.2	392	43449	8	cDNA FLJ75459
1589	P70318	10	28	33.2	392	43389	8	Nucleolysin TIAR
1590	B7ZBW3	10	470	24.4	463	50390	9	Elongation factor 1-alpha
1590	Q05639	10	470	24.4	463	50470	9	Elongation factor 1-alpha 2
1590	P62631	10	470	24.4	463	50454	9	Elongation factor 1-alpha 2
1591	Q9JJU8	10	26	87.7	114	12811	4.9	SH3 domain-binding glutamic acid-rich-like protein
1592	Q9CQV8	10	107	82.9	246	28086	4.8	14-3-3 protein beta/alpha
1593	P62965	10	67	78.1	137	15592	5.4	Cellular retinoic acid-binding protein 1
1594	P70296	10	32	74.3	187	20830	5.4	Phosphatidylethanolamine-binding protein 1
1594	Q3TGC5	10	32	74.3	187	20889	5.6	Putative uncharacterized protein
1595	Q9CQ60	10	29	71.2	257	27254	5.8	6-phosphogluconolactonase
1596	Q9JKB1	10	22	70.9	230	26152	5	Ubiquitin carboxyl-terminal hydrolase isozyme L3
1597	Q61599	10	40	69.5	200	22851	5.1	Rho GDP-dissociation inhibitor 2
1598	O70456	10	20	66.9	248	27713	4.8	14-3-3 protein sigma
1598	Q3TEZ1	10	20	66.9	248	27706	4.8	Stratifin
1599	P45591	10	247	66.9	166	18710	7.9	Cofilin-2
1600	P51410	10	42	65.1	192	21881	10	60S ribosomal protein L9
1600	UPI0000026075	10	42	65.1	192	21853	9.9	UPI0000026075 UniRef100 entry
1601	Q9R0Y5	10	48	63.4	194	21540	5.8	Adenylate kinase isoenzyme 1
1601	Q9R0Y5-2	10	48	58.6	210	23116	5.8	Isoform 2 of Adenylate kinase isoenzyme 1
1602	P30412	10	27	63.2	212	22794	7.5	Peptidyl-prolyl cis-trans isomerase C
1603	P09528	10	79	61	182	21067	5.9	Ferritin heavy chain
1604	Q9R0P3	10	23	61	282	31320	7.1	S-formylglutathione hydrolase
1605	P99029-2	10	79	73.5	162	17015	7.9	Isoform Cytoplasmic+peroxisomal of Peroxiredoxin-5, mitochondrial
1605	P99029	10	79	56.7	210	21897	8.9	Peroxiredoxin-5, mitochondrial
1606	P48428	10	21	54.6	108	12758	5.3	Tubulin-specific chaperone A
1607	O09061	10	63	54.2	240	26372	7.8	Proteasome subunit beta type-1
1608	P24369	10	46	52.8	216	23713	9.6	Peptidyl-prolyl cis-trans isomerase B
1609	Q5FWB6	10	54	51.4	317	34186	6.2	MCG17387, isoform CRA_a
1610	P48758	10	29	50.9	277	30641	8.3	Carbonyl reductase [NADPH] 1
1611	Q91V64	10	35	49.8	297	32033	7.4	Isochorismatase domain-containing protein 1
1612	Q9R1P1	10	44	49.8	205	22965	6.5	Proteasome subunit beta type-3
1613	D3YYE1	10	35	49	198	22954	4.4	Uncharacterized protein
1613	D3Z7M9	10	35	41.6	233	26861	4	Uncharacterized protein
1613	O35381	10	35	39.3	247	28538	4.1	Acidic leucine-rich nuclear phosphoprotein 32 family member A
1614	Q9CYR7	10	20	47.9	303	33728	6.4	Putative uncharacterized protein
1615	O35887	10	18	47.3	315	37064	4.7	Calumenin
1615	Q3UG11	10	18	47.3	315	37065	4.6	Putative uncharacterized protein
1616	Q5NCU4	10	28	46.8	301	34322	4.9	Secreted acidic cysteine rich glycoprotein
1616	P07214	10	28	46.7	302	34450	4.9	SPARC
1617	Q9CRA5	10	35	46.3	298	33752	6.4	Golgi phosphoprotein 3
1618	P23506	10	37	57.3	227	24634	7.6	Protein-L-isoaspartate(D-aspartate) O-methyltransferase
1618	E0CYV0	10	37	45.5	286	30441	7.2	Protein-L-isoaspartate O-methyltransferase
1619	Q9CWS0	10	19	44.9	285	31381	6	N(G),N(G)-dimethylarginine dimethylaminohydrolase 1
1620	Q9QXC1	10	20	43.6	388	42713	6.6	Fetuin-B
1620	Q8CB17	10	20	43.6	388	42743	6.6	Putative uncharacterized protein
1620	Q6YJU2	10	20	41.9	403	44372	6.8	GUGU alpha
1621	P68181-2	10	14	43.2	338	39449	8.6	Isoform 2 of cAMP-dependent protein kinase catalytic subunit beta
1621	P68181-3	10	14	43.1	339	39520	8.6	Isoform 3 of cAMP-dependent protein kinase catalytic subunit beta
1621	P68181	10	14	41.6	351	40708	8.6	cAMP-dependent protein kinase catalytic subunit beta
1621	P68181-4	10	14	36.7	398	46014	8.6	Isoform 4 of cAMP-dependent protein kinase catalytic subunit beta
1622	Q91YR1	10	67	41.4	350	40079	6.7	Twinfilin-1
1623	Q9Z2M7	10	20	41.3	242	27657	6.4	Phosphomannomutase 2
1624	Q3TL79	10	41	41.1	338	38145	5.5	Putative uncharacterized protein
1624	Q8BK64	10	41	41.1	338	38117	5.5	Activator of 90 kDa heat shock protein ATPase homolog 1
1625	Q9DB79	10	52	40.4	166	19415	10.1	Putative uncharacterized protein
1626	P01898	10	20	38.8	325	37251	5.2	H-2 class I histocompatibility antigen, Q10 alpha chain
1627	Q8C0E2	10	16	37.8	336	39125	7.4	Vacuolar protein sorting-associated protein 26B
1628	P28653	10	34	37.1	369	41639	7.3	Biglycan
1629	P51885	10	95	36.1	338	38265	6.4	Lumican
1630	Q8R5C5	10	27	35.9	376	42281	6.4	Beta-centractin
1631	Q3U7I9	10	63	35.6	404	44314	7.6	Putative uncharacterized protein
1631	P18242	10	63	35.1	410	44954	7.1	Cathepsin D
1631	Q3U7P0	10	63	35.1	410	44964	7.1	Putative uncharacterized protein

Table S1. Proteins identified in the OVCAR-3 tumor supernatant by two or more unique peptides.

Protein or Protein Group	Accession Number	Unique Peptide Count	Spectrum Count	Sequence Coverage (%)	Length	MolWt	pI	Descriptive Name
1631	Q3U651	10	63	35.1	410	45000	7.2	Putative uncharacterized protein
1631	Q3TXL5	10	63	35.1	410	44927	7.1	Putative uncharacterized protein
1631	Q3TWR6	10	63	35.1	410	44972	7.1	Putative uncharacterized protein
1631	Q3TWD0	10	63	35.1	410	44928	7.2	Putative uncharacterized protein
1631	Q8C243	10	63	32.4	445	48374	7.3	Putative uncharacterized protein
1632	P97807-2	10	21	38.1	467	50067	7.9	Isoform Cytoplasmic of Fumarate hydratase, mitochondrial
1632	P97807	10	21	35.1	507	54371	9	Fumarate hydratase, mitochondrial
1632	Q3UIA9	10	21	35.1	507	54357	9	Fumarate hydratase 1
1633	P07310	10	19	34.6	381	43045	7.1	Creatine kinase M-type
1633	Q9D6U7	10	19	34.6	381	43031	7.1	Putative uncharacterized protein
1634	Q8CB58	10	140	35.8	489	52631	9	MCG13402, isoform CRA_d
1634	Q8BGJ5	10	140	33.1	529	56892	9.2	MCG13402, isoform CRA_a
1634	Q3T984	10	140	33	530	56963	9.2	Putative uncharacterized protein
1634	Q80T07	10	140	31.6	554	59179	9.2	Polypirimidine tract binding protein
1634	Q922I7	10	140	31.5	555	59322	9.2	MCG13402, isoform CRA_c
1634	Q5RJV5	10	140	31.5	555	59264	9.2	Polypyrimidine tract binding protein 1
1634	Q3U5I2	10	140	31.5	555	59276	9.2	Putative uncharacterized protein
1635	Q99KJ8	10	23	32.8	402	44117	5.3	Dynactin subunit 2
1635	UPI0001DAD7E3	10	23	32.7	404	44359	5.2	dynactin subunit 2 isoform 2
1635	UPI0001DAD7E5	10	23	32.4	407	44706	5.2	dynactin subunit 2 isoform 1
1636	Q91WP6	10	17	32.5	418	46718	5.8	Serine protease inhibitor A3N
1637	O08663	10	14	30.1	478	52922	5.8	Methionine aminopeptidase 2
1637	Q3UI33	10	14	29.5	488	54117	5.8	Methionine aminopeptidase
1638	O08553	10	20	25.9	572	62278	6.4	Dihydropyrimidinase-related protein 2
1639	P70389	10	23	25	603	66960	6.6	Insulin-like growth factor-binding protein complex acid labile subunit
1639	Q570Z9	10	23	24.4	619	68499	6.6	MKIAA4111 protein (Fragment)
1639	UPI0001F78FD4	10	23	22	687	76370	6.3	UPI0001F78FD4 UniRef100 entry
1639	Q9JIL0	10	23	22	687	76340	6.3	Als splice variant 2 (Fragment)
1640	P17563	10	20	24.8	472	52514	6.3	Selenium-binding protein 1
1641	Q3THP1	10	48	23.6	738	82883	5.3	Putative uncharacterized protein
1641	Q8C0I6	10	48	23.6	738	82898	5.4	Putative uncharacterized protein
1641	Q8BU29	10	48	23.6	738	82883	5.3	Dipeptidylpeptidase 3, isoform CRA_c
1641	Q6NXZ0	10	48	23.6	738	82955	5.4	Dipeptidylpeptidase 3
1641	Q3UDF3	10	48	23.6	738	82884	5.3	Putative uncharacterized protein
1641	Q3UDD5	10	48	23.6	738	82958	5.4	Putative uncharacterized protein
1642	Q3TDD8	10	48	21.8	609	68710	6.4	Putative uncharacterized protein
1642	Q8BGD9	10	48	21.8	611	68840	5.7	Eukaryotic translation initiation factor 4B
1642	Q3UGC0	10	48	21.8	611	68810	5.7	Putative uncharacterized protein
1642	Q3TSY9	10	48	21.8	611	68840	5.7	Putative uncharacterized protein
1642	Q3TLQ9	10	48	21.8	611	68818	5.7	Putative uncharacterized protein
1643	O08529	10	22	18.1	700	79872	5	Calpain-2 catalytic subunit
1643	Q3U3A8	10	22	18.1	700	79800	5	Putative uncharacterized protein
1644	Q8BVG4	10	12	16	862	98001	6.6	Dipeptidyl peptidase 9
1645	O88207	10	14	9.6	1838	183676	5	Collagen alpha-1(V) chain
1646	E9Q043	10	17	9.2	1732	189090	9.4	Uncharacterized protein
1647	D3Z0Q0	10	16	8.4	1184	132304	8.2	Cartilage intermediate layer protein, nucleotide pyrophosphohydrolase, isoform CRA_a
1647	Q66K08	10	16	8.4	1184	132334	8.2	Cartilage intermediate layer protein 1
1648	Q9ET54-3	10	21	15.7	663	72368	7.7	Isoform 3 of Palladin
1648	Q9ET54-5	10	21	15.5	670	72985	7.5	Isoform 5 of Palladin
1648	Q9ET54-4	10	21	10.4	1002	108287	6.8	Isoform 4 of Palladin
1648	Q9ET54-6	10	21	7.5	1391	150553	6.2	Isoform 6 of Palladin
1648	Q9ET54	10	21	7.4	1408	152131	6.2	Palladin
1649	UPI0001E6B8CE	10	90	4.3	3558	368792	6.4	PREDICTED: protein AHNAK2-like
1650	P31946-2	10	113	83.6	244	27850	4.8	Isoform Short of 14-3-3 protein beta/alpha
1650	P31946	10	113	82.9	246	28082	4.8	14-3-3 protein beta/alpha
1651	Q9Y3C4	10	33	80.6	175	19661	6.8	TP53RK-binding protein
1652	O75368	10	40	78.9	114	12774	5.2	SH3 domain-binding glutamic acid-rich-like protein
1653	Q15185	10	103	78.8	160	18697	4.5	Prostaglandin E synthase 3
1654	P18085	10	263	76.1	180	20511	7.2	ADP-ribosylation factor 4
1655	O75223	10	49	74.5	188	21008	5.1	Gamma-glutamylcyclotransferase
1656	Q9P000	10	25	74.2	198	21819	5.9	COMM domain-containing protein 9
1657	Q14019	10	88	70.4	142	15945	5.7	Coactosin-like protein
1658	P07737	10	437	70	140	15054	8.3	Profilin-1
1659	P68402	10	46	69.4	229	25569	5.9	Platelet-activating factor acetylhydrolase IB subunit beta
1660	P19387	10	18	67.3	275	31441	4.9	DNA-directed RNA polymerase II subunit RPB3
1661	Q86X83	10	28	67.3	199	22745	6.7	COMM domain-containing protein 2
1662	Q9Y530	10	22	67.1	152	17025	8.3	Uncharacterized protein C6orf130
1663	P32321	10	54	66.9	178	20016	7.6	Deoxycytidylate deaminase
1663	P32321-2	10	54	63	189	21014	7.5	Isoform 2 of Deoxycytidylate deaminase
1664	Q9Y3C8	10	24	66.5	167	19458	7.4	Ubiquitin-fold modifier-conjugating enzyme 1
1665	Q01469	10	123	65.2	135	15164	7	Fatty acid-binding protein, epidermal
1666	A6NIH7	10	18	64.9	251	28137	5.7	Protein unc-119 log B
1667	P78330	10	20	64.9	225	25008	5.7	Phosphoserine phosphatase
1668	P36405	10	30	64.8	182	20456	7.2	ADP-ribosylation factor-like protein 3
1669	UPI000198CDA3	10	39	67	191	21714	6.5	UPI000198CDA3 UniRef100 entry
1669	Q9UES0	10	39	67	191	21607	6.5	SNARE protein Ykt6 (Fragment)
1669	O15498	10	39	64.6	198	22418	6.9	Synaptobrevin log YKT6
1670	P02794	10	94	63.4	183	21226	5.6	Ferritin heavy chain
1670	Q6NS36	10	94	50	232	26219	6.5	Ferritin (Fragment)
1671	Q16527	10	77	62.7	193	20954	8.6	Cysteine and glycine-rich protein 2
1672	P15927	10	36	61.1	270	29247	6.1	Replication protein A 32 kDa subunit
1672	P15927-2	10	36	59.4	278	30156	7	Isoform 2 of Replication protein A 32 kDa subunit
1672	P15927-3	10	36	46.1	358	38810	8.9	Isoform 3 of Replication protein A 32 kDa subunit
1673	Q9H2J4	10	17	61.1	239	27614	4.8	Phosducin-like protein 3
1674	Q9GZP4	10	25	59.7	211	24178	5.8	PITH domain-containing protein 1
1675	Q15274	10	28	59.6	297	30846	6.2	Nicotinate-nucleotide pyrophosphorylase [carboxylating]
1676	P57735	10	28	59.2	213	23496	6	Ras-related protein Rab-25
1677	P07203	10	31	59.1	203	21938	6.5	Glutathione peroxidase 1
1678	B3KPC7	10	42	58.8	153	16971	6	Actin-related protein 2/3 complex subunit 5
1678	Q9BPX5	10	42	58.8	153	16941	6.6	Actin-related protein 2/3 complex subunit 5-like protein
1679	Q9Y508	10	41	58.8	228	25694	7.3	RING finger protein 114
1680	Q9UHY7	10	34	58.2	261	28933	4.8	Enolase-phosphatase E1
1681	Q13907	10	24	57.7	227	26319	6.4	Isopentenyl-diphosphate Delta-isomerase 1
1682	Q13162	10	32	57.2	271	30540	6.3	Peroxiredoxin-4
1683	O75663	10	30	56.6	272	31444	5.9	TIP41-like protein
1684	P30044-2	10	193	74.1	162	17031	7.2	Isoform Cytoplasmic+peroxisomal of Peroxiredoxin-5, mitochondrial
1684	P30044	10	193	56.1	214	22086	8.7	Peroxiredoxin-5, mitochondrial
1685	B4E1H6	10	24	55.5	218	23782	7.5	Guanylate kinase 1, isoform CRA_e
1686	Q13938	10	25	52.9	189	20967	4.9	Calcyphosin
1686	Q8NF12	10	25	31.1	322	35036	6.7	FLJ00390 protein (Fragment)

Table S1. Proteins identified in the OVCAR-3 tumor supernatant by two or more unique peptides.

Protein or Protein Group	Accession Number	Unique Peptide Count	Spectrum Count	Sequence Coverage (%)	Length	MolWt	pI	Descriptive Name
1687	B2R7M3	10	38	52.8	320	35448	8.4	cDNA, FLJ93510, highly similar to sapiens JTV1 gene (JTV1), mRNA
1687	Q13155	10	38	52.8	320	35349	8.2	Aminoacyl tRNA synthase complex-interacting multifunctional protein 2
1688	Q53H96	10	17	52.2	274	28649	7.5	Pyrroline-5-carboxylate reductase 3
1689	B4DT01	10	38	51.5	305	33435	8.1	cDNA FLJ60347, highly similar to sapiens hydroxyacylglutathione hydrolase (HAGH), transcript variant 1, mRNA
1689	Q16775	10	38	51	308	33806	8.1	Hydroxyacylglutathione hydrolase, mitochondrial
1690	A8K4Z4	10	72	51.4	317	34239	6	cDNA FLJ75549, highly similar to sapiens ribosomal protein, large, P0 (RPLP0), transcript variant 1, mRNA
1690	Q53HW2	10	72	51.4	317	34302	6	Ribosomal protein P0 variant (Fragment)
1690	P05388	10	72	51.4	317	34274	6	60S acidic ribosomal protein P0
1691	B4DTR0	10	31	51.2	260	29653	6.5	cDNA FLJ54885, weakly similar to Mus musculus potassium channel tetramerisation domain containing 11 (Kctd11), mRNA
1691	Q4G0X4	10	31	51.2	260	29643	6.5	BTB/POZ domain-containing protein KCTD21
1692	Q9UN19	10	17	51.1	280	32194	7.8	Dual adapter for phosphotyrosine and 3-phosphotyrosine and 3-phosphoinositide
1693	P39019	10	79	51	145	16060	10.3	40S ribosomal protein S19
1693	Q8WVX7	10	79	47.1	157	17282	10.5	Ribosomal protein S19 (Fragment)
1694	O15347	10	31	50	200	22980	8.4	High mobility group protein B3
1695	P49720	10	66	49.8	205	22949	6.5	Proteasome subunit beta type-3
1696	UPI000045684C	10	71	50	250	26552	9.6	Deoxyuridine 5'-triphosphate nucleotidohydrolase, mitochondrial precursor (EC 3.6.1.23) (dUTPase) (dUTP pyrophosphatase).
1696	P33316	10	71	49.6	252	26706	9.6	Deoxyuridine 5'-triphosphate nucleotidohydrolase, mitochondrial
1696	P33316-3	10	71	49.6	252	26563	9.4	Isoform 3 of Deoxyuridine 5'-triphosphate nucleotidohydrolase, mitochondrial
1697	Q9UKL6	10	30	49.5	214	24843	5.8	Phosphatidylcholine transfer protein
1698	P26583	10	41	49.3	209	24034	7.8	High mobility group protein B2
1699	Q9BVG4	10	28	48.9	233	26057	4.8	UPF0368 protein Cxorf26
1700	Q9H0Q0	10	14	48	323	37313	6	Protein FAM49A
1701	B4DQU7	10	52	47.5	278	31614	7.6	cDNA FLJ55032, highly similar to Caspase-7 (EC 3.4.22.-)
1701	P55210	10	52	43.6	303	34277	6.1	Caspase-7
1701	P55210-3	10	52	39.3	336	37815	6.4	Isoform Alpha' of Caspase-7
1702	Q96GA7	10	21	47.1	329	34674	6.9	Serine dehydratase-like
1703	Q8IW45	10	11	47	347	36576	8.1	Carbohydrate kinase domain-containing protein
1704	P23284	10	57	46.8	216	23743	9.4	Peptidyl-prolyl cis-trans isomerase B
1705	Q96HY6	10	45	46.5	314	35611	5.1	DDRGK domain-containing protein 1
1706	Q96C86	10	20	46.3	337	38609	6.4	Scavenger mRNA-decapping enzyme DcpS
1707	P46736-2	10	19	50.2	291	33150	6	Isoform 1 of Lys-63-specific deubiquitinase BRCC36
1707	P46736-3	10	19	50	292	33237	6	Isoform 3 of Lys-63-specific deubiquitinase BRCC36
1707	P46736	10	19	46.2	316	36072	5.9	Lys-63-specific deubiquitinase BRCC36
1708	E7EQL0	10	15	45.8	286	31417	8.6	Uncharacterized protein
1708	Q9NZ30	10	15	45.8	286	31303	8.9	Uncharacterized hypothalamus protein HCDASE
1708	Q9NTX5	10	15	42.7	307	33698	8.2	Enoyl-CoA hydratase domain-containing protein 1
1709	P40121	10	36	45.7	348	38499	6.2	Macrophage-capping protein
1710	Q969E4	10	30	45.5	200	22502	4.9	Transcription elongation factor A protein-like 3
1711	P36969-2	10	50	51.8	170	19375	7.9	Isoform Cytoplasmic of Phospholipid hydroperoxide glutathione peroxidase, mitochondrial
1711	P36969	10	50	44.7	197	22025	8.4	Phospholipid hydroperoxide glutathione peroxidase, mitochondrial
1711	UPI000096F213	10	50	37.6	234	26948	10.1	phospholipid hydroperoxide glutathione peroxidase, mitochondrial isoform C precursor
1712	A8MV37	10	21	44.2	308	34012	5.7	Uncharacterized protein
1712	P55735	10	21	42.2	322	35541	5.5	Protein SEC13 log
1713	P31689	10	29	44.1	397	44868	7.1	DnaJ log subfamily A member 1
1714	UPI00006C0D04	10	27	41.9	370	40821	8.9	PREDICTED: actin-related protein 2/3 complex subunit 1B-like
1714	O15143	10	27	41.7	372	40950	8.4	Actin-related protein 2/3 complex subunit 1B
1715	Q9BSV6	10	13	41.6	310	33652	8.4	tRNA-splicing endonuclease subunit Sen34
1716	Q9UNP9-2	10	26	43.6	296	33085	5.6	Isoform B of Peptidyl-prolyl cis-trans isomerase E
1716	Q9UNP9	10	26	42.9	301	33431	5.6	Peptidyl-prolyl cis-trans isomerase E
1716	Q3S611	10	26	41.1	314	35020	6.2	Cyclophilin-33B
1716	Q5TGA3	10	26	41.1	314	34991	6.4	Peptidylprolyl isomerase E (Cyclophilin E)
1717	P48556	10	26	40.6	350	39612	9.7	26S proteasome non-ATPase regulatory subunit 8
1718	Q5VW32	10	22	39.4	411	46476	7.6	BRO1 domain-containing protein BROX
1719	Q53GE7	10	18	39	292	33595	4.9	Tetratricopeptide repeat domain 1 variant (Fragment)
1719	Q99614	10	18	39	292	33526	4.8	Tetratricopeptide repeat protein 1
1719	Q53HD9	10	18	39	292	33454	4.9	Tetratricopeptide repeat domain 1 variant (Fragment)
1720	Q9NWY4	10	16	38.4	346	39436	6.8	UPF0609 protein C4orf27
1721	Q96P16-2	10	33	40.8	289	32917	6.7	Isoform 2 of Regulation of nuclear pre-mRNA domain-containing protein 1A
1721	Q96P16	10	33	37.8	312	35720	7.5	Regulation of nuclear pre-mRNA domain-containing protein 1A
1722	Q53HC9	10	14	39.5	387	43603	5.1	Protein TSSC1
1722	A8MUM1	10	14	37	414	46320	5.1	Uncharacterized protein
1723	B4DMI6	10	32	36.7	316	35175	7.8	WDR45-like, isoform CRA_f
1723	Q5MNZ6	10	32	33.7	344	38122	7.6	WD repeat domain phosphoinositide-interacting protein 3
1724	Q13501	10	28	35.9	440	47687	5.2	Sequestosome-1
1725	P53365	10	38	35.8	341	37856	6	Arfaptin-2
1726	B4E0U6	10	38	35.4	359	40161	5.3	cDNA FLJ53687, highly similar to Hsc70-interacting protein
1726	P50502	10	38	34.4	369	41332	5.3	Hsc70-interacting protein
1727	O43237	10	24	34.8	492	54099	6.4	Cytoplasmic dynein 1 light intermediate chain 2
1728	P09543	10	15	34.4	421	47579	9.1	2',3'-cyclic-nucleotide 3'-phosphodiesterase
1729	P50148	10	13	34.3	359	42142	5.7	Guanine nucleotide-binding protein G(q) subunit alpha
1730	Q9H0P0-1	10	15	38.4	297	33915	5.9	Isoform 1 of Cytosolic 5'-nucleotidase 3
1730	Q9H0P0	10	15	33.9	336	37948	7.1	Cytosolic 5'-nucleotidase 3
1731	B2R9H3	10	12	32.4	374	42755	5.5	cDNA, FLJ94391, highly similar to sapiens serine (or cysteine) proteinase inhibitor, clade B (ovalbumin), member 8 (SERPINB8), mRNA
1732	UPI0001EC9622	10	14	33	455	50243	5.5	programmed cell death protein 4 isoform 3
1732	B4DKX4	10	14	33	455	50229	5.5	cDNA FLJ58014, highly similar to sapiens programmed cell death 4, transcript variant 1, mRNA
1732	UPI00015E017C	10	14	32.8	457	50394	5.5	UPI00015E017C UniRef100 entry
1732	B5ME91	10	14	32.8	458	50576	5.5	Uncharacterized protein
1732	B2R6E2	10	14	32	469	51631	5.2	cDNA, FLJ92910, highly similar to sapiens programmed cell death 4 (neoplastic transformation inhibitor) (PDCD4), transcript variant 1, mRNA
1732	Q53EL6	10	14	32	469	51735	5.2	Programmed cell death protein 4
1733	P31146	10	16	31.5	461	51026	6.7	Coronin-1A
1734	Q9UHL4	10	24	31.3	492	54341	6.3	Dipeptidyl peptidase 2
1735	Q59GU6	10	22	32.6	432	50099	7	Sorting nexin 1 isoform a variant (Fragment)
1735	A6NKH4	10	22	29.7	474	53304	5	Sorting nexin 1, isoform CRA_d
1735	Q53GY8	10	22	27	522	59012	5.2	Sorting nexin 1 isoform a variant (Fragment)
1735	Q13596	10	22	27	522	59070	5.1	Sorting nexin-1

Table S1. Proteins identified in the OVCAR-3 tumor supernatant by two or more unique peptides.

Protein or Protein Group	Accession Number	Unique Peptide Count	Spectrum Count	Sequence Coverage (%)	Length	MolWt	pI	Descriptive Name
1736	Q96FC7	10	23	29.3	376	42486	6.4	Phytanoyl-CoA hydroxylase-interacting protein-like
1737	Q6YP21	10	15	29.1	454	51400	8.2	Kynurenine--oxoglutarate transaminase 3
1738	Q99733	10	33	29.9	375	42823	4.7	Nucleosome assembly protein 1-like 4
1738	B2R6J4	10	33	29.9	375	42795	4.7	cDNA, FLJ92975, highly similar to sapiens nucleosome assembly protein 1-like 4 (NAP1L4), mRNA
1738	B7ZAK9	10	33	29.2	384	43836	4.7	cDNA, FLJ79223, highly similar to Nucleosome assembly protein 1-like 4
1738	A8MZ22	10	33	29	386	44079	4.7	Nucleosome assembly protein 1-like 4b
1738	B7ZB83	10	33	29	386	44080	4.7	cDNA, FLJ79447, highly similar to Nucleosome assembly protein 1-like 4
1738	B4DS05	10	33	29	386	44095	4.7	cDNA FLJ59403, highly similar to Nucleosome assembly protein 1-like 4
1739	A8K0B6	10	32	28.4	395	44674	7.6	cDNA FLJ77858, highly similar to sapiens N-acylsphingosine amidohydrolase (acid ceramidase) 1 (ASAH1), transcript variant 1, mRNA
1739	Q53H01	10	32	28.4	395	44692	7.6	N-acylsphingosine amidohydrolase (Acid ceramidase) 1 preproprotein isoform a variant (Fragment)
1739	D3DSQ1	10	32	20.5	546	60683	8.8	N-acylsphingosine amidohydrolase (Acid ceramidase) 1, isoform CRA_c
1740	C9JG97	10	22	28.7	415	44693	4.6	Uncharacterized protein
1740	C9JEH3	10	22	27.4	435	46822	4.4	Angio-associated, migratory cell protein, isoform CRA_a
1740	Q13685	10	22	27.4	434	46751	4.4	Angio-associated migratory cell protein
1741	P35659	10	31	27.2	375	42674	8.6	Protein DEK
1742	B4DWU2	10	29	30.3	393	43568	6.6	cDNA FLJ54191, highly similar to Annexin A7
1742	B4DHY4	10	29	27.4	434	47739	5.7	cDNA FLJ55060, highly similar to Annexin A7
1742	Q53HM8	10	29	25.5	466	50228	7.2	Annexin VII isoform 1 variant (Fragment)
1742	P20073-2	10	29	25.5	466	50316	6.6	Isoform 2 of Annexin A7
1742	B2R657	10	29	24.4	488	52640	5.5	cDNA, FLJ92798, highly similar to sapiens annexin A7 (ANXA7), transcript variant 2, mRNA
1742	P20073	10	29	24.4	488	52739	5.7	Annexin A7
1743	B4E365	10	20	24.2	624	70031	7	cDNA FLJ56210, highly similar to Zinc phosphodiesterase ELAC protein 2 (EC 3.1.26.11)
1743	E7ES68	10	20	24.2	624	70005	7	Uncharacterized protein
1743	UPI000013F7BC	10	20	18.7	807	90037	8	Zinc phosphodiesterase ELAC protein 2 (EC 3.1.26.11) (Ribonuclease Z 2) (RNase Z 2) (tRNase Z 2) (tRNA 3 endonuclease 2) (ElaC log protein 2) (Heredity prostate cancer protein 2).
1743	E9PGJ0	10	20	18.7	809	90228	8	Uncharacterized protein
1743	UPI0000EE6311	10	20	18.3	825	92091	7.8	zinc phosphodiesterase ELAC protein 2 isoform 2
1743	Q9BQ52	10	20	18.3	826	92219	7.9	Zinc phosphodiesterase ELAC protein 2
1744	Q8NBH6	10	20	25.4	638	70578	5.9	cDNA PSEC0266 fis, clone NT2RP3003649, highly similar to sapiens fibulin-1D mRNA
1744	P23142	10	20	23	703	77214	5.2	Fibulin-1
1744	UPI00001C1A97	10	20	23	703	77242	5.3	fibulin-1 isoform D precursor
1745	B4DIT7	10	15	22.6	606	68649	5.3	cDNA FLJ58187, highly similar to Protein-glutamine gamma-glutamyltransferase 2(EC 2.3.2.13)
1745	P21980	10	15	19.9	687	77329	5.2	Protein-glutamine gamma-glutamyltransferase 2
1746	O75131	10	18	21.8	537	60131	5.8	Copine-3
1747	E2RRT1	10	192	24.8	548	59712	9.5	Uncharacterized protein
1747	Q15637-6	10	192	23.8	571	61889	9.3	Isoform 6 of Splicing factor 1
1747	B7Z1Q1	10	192	22.2	613	65387	8.4	cDNA FLJ54737, highly similar to Splicing factor 1
1747	Q15637-3	10	192	21.8	623	67277	9.2	Isoform 3 of Splicing factor 1
1747	E7ESU3	10	192	21.8	624	67374	9.2	Uncharacterized protein
1747	B4DJU4	10	192	21.6	630	67350	9.1	cDNA FLJ53344, highly similar to Splicing factor 1
1747	B0KWK3	10	192	21.3	638	68633	9.2	Splicing factor 1 isoform 2 (Predicted)
1747	Q15637	10	192	21.3	639	68330	9	Splicing factor 1
1747	Q15637-5	10	192	17.8	764	80621	9.5	Isoform 5 of Splicing factor 1
1748	Q9Y3Z3	10	18	18.8	626	72201	7.1	SAM domain and HD domain-containing protein 1
1749	Q8N573-2	10	18	17.5	839	93734	5.2	Isoform 2 of Oxidation resistance protein 1
1749	Q8N573-5	10	18	17.4	846	94692	5.3	Isoform 5 of Oxidation resistance protein 1
1749	D3HIS6	10	18	16.8	873	97916	5.4	Oxidation resistance 1
1749	Q8N573	10	18	16.8	874	97970	5.5	Oxidation resistance protein 1
1750	Q68E01	10	24	16.2	1043	118070	5.8	Integrator complex subunit 3
1750	UPI0001AE78D3	10	24	16.2	1043	118084	5.8	UPI0001AE78D3 UniRef100 entry
1750	Q68E01-2	10	24	16.2	1042	118013	5.8	Isoform 2 of Integrator complex subunit 3
1751	A8K6Q8	10	17	15.7	760	84873	6.6	cDNA FLJ75881, highly similar to sapiens transferrin receptor (p90, CD71) (TFRC), mRNA
1751	P02786	10	17	15.7	760	84871	6.6	Transferrin receptor protein 1
1752	Q15334	10	12	15.3	1064	115418	6.3	Lethal(2) giant larvae protein log 1
1753	Q6P1M3-2	10	13	15.2	1015	112927	7.3	Isoform A of Lethal(2) giant larvae protein log 2
1753	Q6P1M3	10	13	15.1	1020	113448	7.5	Lethal(2) giant larvae protein log 2
1754	Q14157-4	10	84	16.6	976	103168	6.9	Isoform 4 of Ubiquitin-associated protein 2-like
1754	Q14157-1	10	84	16.5	983	103930	7	Isoform 2 of Ubiquitin-associated protein 2-like
1754	Q14157-3	10	84	15.2	1068	112580	6.6	Isoform 3 of Ubiquitin-associated protein 2-like
1754	B4DYY5	10	84	15	1078	113560	6.7	cDNA FLJ55671, highly similar to Ubiquitin-associated protein 2-like
1754	Q14157	10	84	14.9	1087	114534	7.1	Ubiquitin-associated protein 2-like
1755	P52735-3	10	31	14.9	839	97032	6.9	Isoform 3 of Guanine nucleotide exchange factor VAV2
1755	UPI000179A876	10	31	14.9	839	97120	7.1	Protein vav-2.
1756	Q68EM8	10	12	14.7	1017	113407	6.6	MAN2C1 protein
1756	Q9NTJ4	10	12	14.3	1040	115835	6.6	Alpha-mannosidase 2C1
1757	P35442	10	22	12.7	1172	129991	4.8	Thrombospondin-2
1758	A1L3A9	10	15	11.6	1232	138540	5.3	TBC1 domain family, member 9B (With GRAM domain)
1758	Q66K14-2	10	15	11.6	1233	138670	5.3	Isoform 2 of TBC1 domain family member 9B
1758	Q66K14	10	15	11.4	1250	140525	5.3	TBC1 domain family member 9B
1758	B9A6K8	10	15	11.4	1250	140497	5.2	TBC1 domain family, member 9B
1759	O95490-3	10	15	11.8	1123	126162	6.9	Isoform 3 of Latrophilin-2
1759	A8MW65	10	15	11.4	1170	131524	7	Uncharacterized protein
1759	UPI000059CFFE	10	15	9.8	1351	151250	6.6	UPI000059CFFE UniRef100 entry
1759	E9PEC7	10	15	9.6	1391	155852	6.5	Uncharacterized protein
1759	B1ALU1	10	15	9.6	1384	155548	6.1	Latrophilin 2
1759	O95490-2	10	15	9.5	1403	157177	6.2	Isoform 2 of Latrophilin-2
1759	B1ALU2	10	15	9.4	1416	158796	6.4	Latrophilin 2
1759	B1ALU3	10	15	9.3	1431	160514	6.3	Latrophilin 2
1759	O95490	10	15	9.1	1459	163348	6.4	Latrophilin-2
1759	B1ALU5	10	15	9.1	1461	163447	6.1	Latrophilin 2
1759	B1ALU4	10	15	9	1474	165066	6.3	Latrophilin 2
1760	Q9P2M7	10	19	8.9	1197	136386	5.5	Cingulin
1760	B9EK46	10	19	8.8	1203	137103	5.5	Cingulin
1761	A4QN19	10	15	7.9	1727	186492	5.4	SEC16A protein (Fragment)
1761	A4QN18	10	15	7	1961	211073	5.5	SEC16A protein (Fragment)
1761	O15027-4	10	15	6.4	2154	231133	5.7	Isoform 4 of Protein transport protein Sec16A
1761	O15027-2	10	15	6.4	2134	228868	5.6	Isoform 2 of Protein transport protein Sec16A

Table S1. Proteins identified in the OVCAR-3 tumor supernatant by two or more unique peptides.

Protein or Protein Group	Accession Number	Unique Peptide Count	Spectrum Count	Sequence Coverage (%)	Length	MolWt	pI	Descriptive Name
1761	O15027-3	10	15	6.3	2159	231249	5.6	Isoform 3 of Protein transport protein Sec16A
1761	O15027	10	15	6.3	2179	233515	5.6	Protein transport protein Sec16A
1761	O15027-5	10	15	6.2	2201	235750	5.7	Isoform 5 of Protein transport protein Sec16A
1762	Q99102-13	10	17	14.6	1176	130415	6.3	Isoform 13 of Mucin-4
1762	UPI0001AE7669	10	17	9	1912	203125	6	Mucin-4 precursor (Pancreatic adenocarcinoma mucin) (Testis mucin) (Ascites sialoglycoprotein) (ASGP) (Tracheobronchial mucin) [Contains: Mucin-4 alpha chain (Ascites sialoglycoprotein 1) (ASGP-1); Mucin-4 beta chain (Ascites sialoglycoprotein 2) (ASGP-2)
1762	Q99102-17	10	17	8.9	1943	206629	6.3	Isoform 17 of Mucin-4
1762	Q99102	10	17	7.9	2169	231542	6.3	Mucin-4
1762	E7ESK3	10	17	3.3	5284	530070	5.4	Uncharacterized protein
1762	E9PDY6	10	17	3.2	5412	542314	5.3	Uncharacterized protein
1763	A5D987	9	89	76.3	152	17085	6	Ubiquitin carrier protein
1763	P61088	9	89	76.3	152	17138	6.6	Ubiquitin-conjugating enzyme E2 N Ubiquitin-conjugating enzyme E2 N
1764	P60660	9	141	73.5	151	16930	4.7	Myosin light polypeptide 6 Myosin light polypeptide 6
1765	P67870	9	58	67.4	215	24942	5.6	Casein kinase II subunit beta Casein kinase II subunit beta
1766	Q3SYF1	9	30	64.2	162	18885	8.4	Sorting nexin 12 Sorting nexin 12
1766	O70493	9	30	63	165	19116	7.3	Sorting nexin-12
1766	D3ZDQ6	9	30	62.7	166	19246	7.2	Uncharacterized protein
1766	D4A719	9	30	61.2	170	19808	8.4	Uncharacterized protein
1766	UPI0000D61D89	9	30	60.5	172	19819	7.8	Sorting nexin-12.
1766	Q9UMY4	9	30	60.5	172	19730	7.9	Sorting nexin-12
1767	P61020	9	76	59.5	215	23707	8.1	Ras-related protein Rab-5B Ras-related protein Rab-5B
1767	UPI00006042CF	9	76	45.7	280	30625	8	PREDICTED: ras-related protein Rab-5B
1768	Q13185	9	144	59	183	20811	5.3	Chromobox protein log 3 Chromobox protein log 3
1768	Q32P00	9	144	59	183	20812	5.1	Chromobox log 3 (Drosophila HP1 gamma)
1769	P08708	9	157	57	135	15550	9.8	40S ribosomal protein S17
1769	P63276	9	157	57	135	15524	9.8	40S ribosomal protein S17
1769	Q8BT90	9	157	54.6	141	16141	9.9	Putative uncharacterized protein (Fragment)
1770	Q53FX5	9	32	56.9	197	21820	8	Lin-7 log C variant (Fragment)
1770	Q9NUP9	9	32	56.9	197	21834	8.4	Protein lin-7 log C Protein lin-7 log C
1771	UPI0000565CDE	9	144	56.8	192	21883	10	UPI0000565CDE UniRef100 entry
1771	P62081	9	144	56.2	194	22127	10.1	40S ribosomal protein S7 40S ribosomal protein S7
1772	P61966	9	84	54.4	158	18733	5.7	AP-1 complex subunit sigma-1A AP-1 complex subunit sigma-1A
1772	UPI000198CE93	9	84	45	191	22178	9.2	UPI000198CE93 UniRef100 entry
1773	P41223	9	54	53.5	144	17000	8.8	Protein BUD31 log Protein BUD31 log
1774	P62805	9	420	53.4	103	11367	11.4	Histone H4 Histone H4
1775	P62158	9	32	69.1	149	16838	4.2	Calmodulin Calmodulin
1775	E7ETZ0	9	32	68.7	150	16966	4.2	Uncharacterized protein
1775	UPI00018815D7	9	32	55.1	187	20762	4.4	UPI00018815D7 UniRef100 entry
1775	E7EMB3	9	32	52.6	196	21689	4.6	Uncharacterized protein
1775	Q3UKW2	9	32	52.3	197	21560	4.3	Putative uncharacterized protein
1776	O95372	9	46	51.5	231	24737	7.2	Acyl-protein thioesterase 2
1776	Q9WTL7	9	46	51.5	231	24794	7.2	Acyl-protein thioesterase 2
1777	Q9NRW1	9	128	51	208	23462	5.5	Ras-related protein Rab-6B Ras-related protein Rab-6B
1778	P60953	9	82	62.8	191	21259	6.5	Cell division control protein 42 log Cell division control protein 42 log
1778	B4E1U9	9	82	50.8	236	26528	7.6	cDNA FLJ54776, highly similar to Cell division control protein 42 log
1779	Q922F4	9	86	45	447	50090	4.9	Tubulin beta-6 chain Tubulin beta-6 chain
1780	B2RE59	9	10	40.1	299	33617	8	cDNA, FLJ93148, highly similar to sapiens RCD1 required for cell differentiation1 log (S. pombe) (RQCD1), mRNA
1780	Q9JKY0	9	10	40.1	299	33601	8	Cell differentiation protein RCD1 log
1780	Q92600	9	10	40.1	299	33631	8	Cell differentiation protein RCD1 log
1781	Q8VE80	9	12	39.9	351	38738	6.1	THO complex subunit 3
1781	Q96J01	9	12	39.9	351	38772	6.1	THO complex subunit 3
1782	P62753	9	120	33.3	249	28681	10.8	40S ribosomal protein S6 40S ribosomal protein S6
1782	Q96DV6	9	120	33.3	249	28709	10.9	40S ribosomal protein S6
1783	Q9CWZ7	9	15	30.8	312	34732	5.4	Gamma-soluble NSF attachment protein
1783	Q99747	9	15	30.8	312	34746	5.4	Gamma-soluble NSF attachment protein
1783	D3Z4B2	9	15	27.2	353	39367	6	Uncharacterized protein
1784	B2R9L6	9	15	23.1	372	42747	8.8	cDNA, FLJ94450, highly similar to sapiens cyclin-dependent kinase 9 (CDC2-related kinase) (CDK9), mRNA
1784	Q99J95	9	15	23.1	372	42762	8.8	Cyclin-dependent kinase 9
1784	P50750	9	15	23.1	372	42778	8.8	Cyclin-dependent kinase 9
1784	C9E1C9	9	15	23.1	372	42762	8.9	CDK9
1784	B5BU53	9	15	23.1	372	42779	8.7	Cyclin-dependent kinase 9
1784	P50750-2	9	15	17.6	489	53365	9.5	Isoform 2 of Cyclin-dependent kinase 9
1785	C9J2R9	9	28	24.8	460	51567	5.5	Uncharacterized protein
1785	A3KGC1	9	28	21	544	60781	5.4	p21 (CDKN1A)-activated kinase 3
1785	O75914-2	9	28	21	544	60693	5.5	Isoform 2 of Serine/threonine-protein kinase PAK 3
1785	Q61036	9	28	20.4	559	62398	5.4	Serine/threonine-protein kinase PAK 3
1785	O75914	9	28	20.4	559	62310	5.5	Serine/threonine-protein kinase PAK 3
1785	B1GX80	9	28	20.2	565	63001	5.5	PAK3c protein
1785	B1GX78	9	28	20.2	565	62913	5.6	PAK3c protein
1785	B1GX81	9	28	19.7	580	64618	5.5	PAK3cb protein
1785	B1GX79	9	28	19.7	580	64530	5.5	PAK3cb protein
1786	Q9DAW6	9	14	20.2	521	58370	7.3	U4/U6 small nuclear ribonucleoprotein Prp4
1786	Q8BTJ0	9	14	20.2	521	58354	7.4	Putative uncharacterized protein
1786	Q6IAP9	9	14	20.2	521	58307	7.4	PRPF4 protein
1786	B2R7V4	9	14	20.1	522	58449	7.4	cDNA, FLJ93619, highly similar to sapiens PRP4 pre-mRNA processing factor 4 log (yeast) (PRPF4), mRNA
1786	O43172	9	14	20.1	522	58449	7.4	U4/U6 small nuclear ribonucleoprotein Prp4
1786	Q59EL4	9	14	19.6	537	60022	7.6	PRPF4 protein variant (Fragment)
1787	B4E0H3	9	20	18.5	610	66573	9	cDNA FLJ53386, highly similar to Signal recognition particle receptor subunit alpha
1787	E9PJS4	9	20	18.5	610	66559	9	Uncharacterized protein
1787	Q9DBG7	9	20	17.8	636	69623	9	Signal recognition particle receptor subunit alpha
1787	Q6IAX9	9	20	17.7	638	69894	9	SRPR protein
1787	P08240	9	20	17.7	638	69811	9	Signal recognition particle receptor subunit alpha
1788	A8DUV1	9	306	83.8	142	15141	8.2	Alpha-globin
1789	P61750	9	260	76.1	180	20397	7.2	ADP-ribosylation factor 4
1790	Q05816	9	100	72.6	135	15137	6.5	Fatty acid-binding protein, epidermal
1791	P62962	9	257	72.1	140	14957	8.3	Profilin-1
1792	Q9CQI6	9	75	70.4	142	15944	5.4	Coactosin-like protein
1793	P04247	9	25	66.9	154	17070	7.6	Myoglobin
1794	Q60692	9	23	65.1	238	25379	5.1	Proteasome subunit beta type-6
1795	P35285	9	17	60.8	194	21802	8.2	Ras-related protein Rab-22A
1796	Q9CRC9	9	38	58.3	276	31084	6.9	Glucosamine-6-phosphate isomerase 2
1797	Q9WTP6-2	9	20	59.5	232	25606	7.4	Isoform 2 of Adenylate kinase 2, mitochondrial
1797	Q9WTP6	9	20	57.7	239	26469	7.4	Adenylate kinase 2, mitochondrial

Table S1. Proteins identified in the OVCAR-3 tumor supernatant by two or more unique peptides.

Protein or Protein Group	Accession Number	Unique Peptide Count	Spectrum Count	Sequence Coverage (%)	Length	MolWt	pI	Descriptive Name
1798	Q3TKM9	9	45	57.6	151	16228	5.7	Actin-related protein 2/3 complex subunit 5
1798	Q9CPW4	9	45	57.6	151	16288	5.7	Actin-related protein 2/3 complex subunit 5
1799	Q9CQW1	9	25	56.1	198	22314	6.4	Synaptobrevin homolog YKT6
1800	Q3TF84	9	68	54.3	186	20409	6.7	Putative uncharacterized protein
1800	Q922Q8	9	68	32.9	307	34877	9.5	Leucine-rich repeat-containing protein 59
1801	P10649	9	13	53.2	218	25970	8	Glutathione S-transferase Mu 1
1802	Q9QXT0	9	33	52.7	182	20767	5.1	Protein canopy homolog 2
1803	P63325	9	159	51.5	165	18916	10.2	40S ribosomal protein S10
1803	Q3U9P0	9	159	51.5	165	18931	10.3	Putative uncharacterized protein
1804	Q9WVQ5	9	33	49.8	241	26949	6.9	Probable methylthioribulose-1-phosphate dehydratase
1805	P15327	9	13	49.4	259	29978	7.1	Bisphosphoglycerate mutase
1806	O08807	9	23	48.9	274	31053	7.2	Peroxiredoxin-4
1807	D3Z7T1	9	13	48.6	280	32524	4.9	Uncharacterized protein
1807	Q8BH97	9	13	41.5	328	38002	4.9	Reticulocalbin-3
1808	O08583	9	57	48.2	255	26940	11.2	THO complex subunit 4
1809	P19157	9	51	48.1	210	23609	7.8	Glutathione S-transferase P 1
1810	Q3UJ44	9	22	46.1	349	38670	6.7	Putative uncharacterized protein
1810	Q99LB4	9	22	46.1	349	38769	6.9	Capping protein (Actin filament), gelsolin-like
1811	P55258	9	65	44.9	207	23668	9.1	Ras-related protein Rab-8A
1811	Q3UHW5	9	65	44.7	208	23635	8.5	Putative uncharacterized protein
1812	Q6PJ18	9	24	44.7	284	32958	4.7	Tpm2 protein
1813	Q9D1M0	9	17	41.3	322	35566	5.4	Protein SEC13 homolog
1814	Q9R1T2	9	34	37.4	350	38620	5.4	SUMO-activating enzyme subunit 1
1815	Q3TCE7	9	20	36.8	372	41092	8.3	Putative uncharacterized protein
1815	Q9WV32	9	20	36.8	372	41149	8.4	Actin-related protein 2/3 complex subunit 1B
1815	Q9CRC4	9	20	36.8	372	41064	8.4	Actin related protein 2/3 complex, subunit 1B
1815	Q3U094	9	20	36.8	372	41065	8.1	Putative uncharacterized protein
1815	Q91Z25	9	20	36.4	376	41498	8.4	Arpc1b protein
1816	Q99J77	9	21	36.8	359	40024	7.1	N-acetylneuraminic acid synthase (Sialic acid synthase)
1817	Q14AA6	9	46	35.2	216	24357	8	MCG49183
1818	Q9D1E6	9	19	34.4	244	27386	5.2	Tubulin-folding cofactor B
1819	Q3UGC7	9	18	34.1	261	29344	4.8	Eukaryotic translation initiation factor 3, subunit J
1819	Q66JS6	9	18	33.8	263	29486	4.8	Eukaryotic translation initiation factor 3 subunit J
1820	Q8K183	9	10	34	312	35015	6.3	Pyridoxal kinase
1821	Q07456	9	29	33	349	39029	6.3	Protein AMBP
1822	Q9CXX7	9	43	31.4	287	32266	9.9	Putative uncharacterized protein
1822	Q62189	9	43	31.4	287	31835	9.8	U1 small nuclear ribonucleoprotein A
1822	E9Q4T6	9	43	31.2	288	32353	9.9	Uncharacterized protein
1823	Q61247	9	50	30.5	491	54972	6.3	Alpha-2-antiplasmin
1824	UPI0001E6B548	9	31	31	432	47256	6.7	PREDICTED: protein disulfide-isomerase A6-like
1824	Q922R8	9	31	30.5	440	48100	5.1	Protein disulfide-isomerase A6
1825	Q01339	9	19	29	345	38619	8.2	Beta-2-glycoprotein 1
1825	Q9CY42	9	19	29	345	38688	8.3	Putative uncharacterized protein
1826	Q99020	9	112	33.7	285	30831	7.9	Heterogeneous nuclear ribonucleoprotein A/B
1826	Q3TMZ8	9	112	29.5	325	35595	6.9	Putative uncharacterized protein
1826	Q20BD0	9	112	28.9	332	36211	6.9	Heterogeneous nuclear ribonucleoprotein A/B
1827	E9QN57	9	31	26.2	362	40539	5.3	Uncharacterized protein
1827	Q99L47	9	31	25.6	371	41656	5.3	Hsc70-interacting protein
1827	Q3U9V1	9	31	25.6	371	41655	5.4	Putative uncharacterized protein
1827	Q3U8D2	9	31	25.6	371	41557	5.2	Putative uncharacterized protein
1828	P32067	9	30	23.4	415	47756	9.8	Lupus La protein homolog
1828	Q8BTU4	9	30	23.4	415	47657	9.7	Putative uncharacterized protein
1829	Q8R2Y2-2	9	24	20.5	606	66901	5.8	Isoform 2 of Cell surface glycoprotein MUC18
1829	Q8R2Y2	9	24	19.1	648	71546	5.8	Cell surface glycoprotein MUC18
1830	P48722-2	9	14	18.4	817	92252	5.9	Isoform 2 of Heat shock 70 kDa protein 4L
1830	P48722	9	14	17.9	838	94382	5.7	Heat shock 70 kDa protein 4L
1831	Q99KC8	9	17	16.8	793	87143	6.6	von Willebrand factor A domain-containing protein 5A
1832	P20060	9	15	16.4	536	61116	8.1	Beta-hexosaminidase subunit beta
1833	A0AUN0	9	19	15.1	842	95117	6	Sec23ip protein (Fragment)
1833	Q6NZC7	9	19	12.7	998	110780	5.9	SEC23-interacting protein
1833	Q4G0C0	9	19	12.7	998	110751	5.7	Sec23 interacting protein
1834	Q99KD5	9	10	11.8	944	103447	6.3	Protein unc-45 homolog A
1835	UPI000024F693	9	20	11.6	1085	124462	5.2	UPI000024F693 UniRef100 entry
1836	Q3UMB9	9	18	11.1	1173	136370	7.4	WASH complex subunit 7
1837	Q6ZQ58	9	47	10.7	1072	121152	8.8	La-related protein 1
1837	UPI00001E46C4	9	47	10.7	1072	121125	8.8	la-related protein 1
1838	UPI0001F795E5	9	16	8.4	1222	115176	8.4	UPI0001F795E5 UniRef100 entry
1838	Q8K173	9	16	8.4	1222	115140	8.4	Col3a1 protein (Fragment)
1838	P08121	9	16	7	1464	138944	6.5	Collagen alpha-1(III) chain
1838	Q8BLW4	9	16	7	1464	138942	6.6	Putative uncharacterized protein
1838	Q8BKY2	9	16	7	1464	138947	6.5	Putative uncharacterized protein
1838	Q7TT32	9	16	7	1464	138972	6.5	Collagen, type III, alpha 1
1838	Q3UH72	9	16	7	1464	138886	6.6	Putative uncharacterized protein
1839	Q64511	9	13	6.8	1612	181908	8.3	DNA topoisomerase 2-beta
1840	E9QP46	9	17	1.5	6870	782157	5.3	Uncharacterized protein
1840	Q6ZWQ0	9	17	1.5	6874	782738	5.3	Nesprin-2
1841	P49773	9	84	81	126	13802	7	Histidine triad nucleotide-binding protein 1
1842	Q9UHA4	9	29	80.6	124	13623	7.4	Ragulator complex protein LAMTOR3
1843	P00441	9	144	79.9	154	15936	6.1	Superoxide dismutase [Cu-Zn]
1844	O75884	9	22	79.6	186	21000	6.2	Putative hydrolase RBBP9
1845	P31949	9	158	76.2	105	11740	7.2	Protein S100-A11
1846	UPI0001F78044	9	15	78.5	158	17234	5.4	UPI0001F78044 UniRef100 entry
1846	Q8TDP1	9	15	75.6	164	17840	5	Ribonuclease H2 subunit C
1847	P46926	9	72	75.4	289	32668	6.9	Glucosamine-6-phosphate isomerase 1
1848	Q8WZ82	9	19	75.3	227	24418	6.9	Ovarian cancer-associated gene 2 protein
1849	P24666	9	67	72.8	158	18042	6.8	Low molecular weight phosphotyrosine protein phosphatase
1849	Q59EH3	9	67	69.7	165	18698	7.9	Acid phosphatase 1 isoform c variant (Fragment)
1850	O14933	9	32	71.9	153	17769	7.9	Ubiquitin/ISG15-conjugating enzyme E2 L6
1851	P55957	9	43	71.8	195	21995	5.4	BH3-interacting domain death agonist
1851	P55957-2	9	43	58.1	241	26836	7.1	Isoform 2 of BH3-interacting domain death agonist
1852	P04632	9	149	71.6	268	28316	5.2	Calpain small subunit 1
1853	P28072	9	26	66.9	239	25358	4.9	Proteasome subunit beta type-6
1854	O43324	9	18	66.7	174	19811	8.6	Eukaryotic translation elongation factor 1 epsilon-1
1855	Q13232	9	36	66.3	169	19015	7.8	Nucleoside diphosphate kinase 3
1856	B2RAZ4	9	47	64.6	195	21628	5.6	cDNA, FLJ95208, highly similar to sapiens PYD and CARD domain containing (PYCARD), transcript variant 1, mRNA
1856	Q9ULZ3	9	47	64.6	195	21627	6.4	Apoptosis-associated speck-like protein containing a CARD
1857	P20290	9	92	64.6	206	22168	9.4	Transcription factor BTF3
1858	O75608	9	41	64.3	230	24670	6.8	Acyl-protein thioesterase 1
1859	E5RFF9	9	13	63	192	22653	4.9	Uncharacterized protein
1859	Q9BRT9	9	13	54.3	223	26047	5	DNA replication complex GINS protein SLD5
1860	E9PJF0	9	18	62.3	316	35737	8.7	Uncharacterized protein
1860	E9PQW4	9	18	58.1	339	38547	9	Uncharacterized protein
1860	UPI0000D579B4	9	18	55.2	357	40088	7.5	mitogen-activated protein kinase 3 isoform 2

Table S1. Proteins identified in the OVCAR-3 tumor supernatant by two or more unique peptides.

Protein or Protein Group	Accession Number	Unique Peptide Count	Spectrum Count	Sequence Coverage (%)	Length	MolWt	pI	Descriptive Name
1860	P27361	9	18	52	379	43136	6.8	Mitogen-activated protein kinase 3
1861	P52907	9	25	61.9	286	32923	5.7	F-actin-capping protein subunit alpha-1
1862	C9J0K6	9	23	72.9	155	17605	5.6	Uncharacterized protein
1862	A8MTH6	9	23	61.7	183	20345	5.3	Uncharacterized protein
1862	P30626	9	23	57.1	198	21676	5.6	Sorcin
1863	P54105	9	57	61.2	237	26215	4.1	Methylosome subunit pICln
1864	Q9BUL8	9	25	59.4	212	24702	8.2	Programmed cell death protein 10
1865	O60362	9	15	59.1	242	27310	8.9	Putative uncharacterized protein 44M2.1
1865	Q9NXG2	9	15	40.5	353	39315	7.9	THUMP domain-containing protein 1
1865	UPI000059D337	9	15	32.6	439	48549	8.8	UPI000059D337 UniRef100 entry
1865	Q6MZT3	9	15	32.6	439	48491	8.9	Putative uncharacterized protein DKFZp686C1054
1866	O43665-3	9	29	58.6	181	21210	7.5	Isoform 3 of Regulator of G-protein signaling 10
1867	Q9BRJ7	9	15	58.3	211	23338	8.9	Protein syndesmos
1868	P80188	9	30	57.6	198	22588	8.9	Neutrophil gelatinase-associated lipocalin
1868	B2ZDQ1	9	30	57.3	199	22687	8.9	Neutrophil gelatinase-associated lipocalin
1868	UPI0000D61913	9	30	57	200	22902	8.9	Neutrophil gelatinase-associated lipocalin precursor (NGAL) (p25) (25 kDa alpha-2-microglobulin-related subunit of MMP-9) (Lipocalin-2) (Oncogene 24p3).
1869	P13693	9	82	57	172	19595	4.9	Translationally-controlled tumor protein
1869	Q5W0H4	9	82	52.1	188	21526	5.5	Tumor protein, translationally-controlled 1
1870	P56537	9	43	56.7	245	26599	4.7	Eukaryotic translation initiation factor 6
1871	P09417	9	23	56.6	244	25789	7.4	Dihydropteridine reductase
1872	B4DDV0	9	83	55.6	169	19103	6	cDNA FLJ51791, highly similar to Lactoylglutathione lyase (EC 4.4.1.5)
1872	Q04760	9	83	51.1	184	20778	5.3	Lactoylglutathione lyase
1873	Q9NZD2	9	14	54.5	209	23850	7.4	Glycolipid transfer protein
1874	P07451	9	31	53.8	260	29557	7.3	Carbonic anhydrase 3
1875	D6RD78	9	88	52.9	191	21246	10	Uncharacterized protein
1875	Q96AG4	9	88	32.9	307	34930	9.6	Leucine-rich repeat-containing protein 59
1876	P60983	9	36	57	142	16713	5.3	Glia maturation factor beta
1876	E7EWZ9	9	36	52.9	153	18053	5.3	Uncharacterized protein
1876	Q9BS35	9	36	52.6	154	18110	5.3	GMFB protein
1877	Q9UL25	9	35	52	225	24348	7.9	Ras-related protein Rab-21
1878	Q96FX7	9	23	51.6	289	31382	7.4	tRNA (adenine-N(1)-)-methyltransferase catalytic subunit TRMT61A
1879	Q9Y3C1	9	20	51.1	178	21188	9.9	Nucleolar protein 16
1880	UPI0001F76FA6	9	19	50.6	261	29737	6.2	HSPE1-MOBKL3 protein
1881	B7ZAD1	9	24	50	240	27295	8.7	NAD(P)H dehydrogenase, quinone 1, isoform CRA_b
1881	P15559	9	24	43.8	274	30868	8.9	NAD(P)H dehydrogenase [quinone] 1
1882	Q5HYI5	9	84	49.8	209	23420	7.5	Putative uncharacterized protein DKFZp313C1541
1882	Q8WUD1	9	84	48.1	216	24214	7.8	Ras-related protein Rab-2B
1883	P10746	9	20	48.7	265	28628	5.5	Uroporphyrinogen-III synthase
1884	Q9H9Q2	9	31	48.5	264	29622	6.2	COP9 signalosome complex subunit 7b
1885	P37235	9	15	47.7	193	22313	5.4	Hippocalcin-like protein 1
1886	Q92688-2	9	53	60	195	22277	4.3	Isoform 2 of Acidic leucine-rich nuclear phosphoprotein 32 family member B
1886	Q53F35	9	53	46.8	250	28673	4.1	Acidic (Leucine-rich) nuclear phosphoprotein 32 family, member B variant (Fragment)
1886	Q92688	9	53	46.6	251	28788	4.1	Acidic leucine-rich nuclear phosphoprotein 32 family member B
1887	Q9NZD8	9	11	45.8	308	34960	6.3	Maspardin
1888	P40306	9	13	45.1	273	28936	7.8	Proteasome subunit beta type-10
1889	B4DDF4	9	49	45	298	32617	7	cDNA FLJ52469, highly similar to Calponin-2
1889	Q99439	9	49	43.4	309	33697	7.3	Calponin-2
1889	Q6FHE4	9	49	43.4	309	33709	7.3	CNN2 protein
1889	B4DUT8	9	49	40.6	330	35944	7.3	cDNA FLJ52765, highly similar to Calponin-2
1890	P25325	9	14	44.4	297	33178	6.6	3-mercaptopyruvate sulfurtransferase
1890	UPI000057921F	9	14	41.6	317	35250	6.6	3-mercaptopyruvate sulfurtransferase isoform 1
1891	Q7L266	9	18	44.2	308	32055	6.2	L-asparaginase
1892	Q96C19	9	31	44.2	240	26697	5.2	EF-hand domain-containing protein D2
1893	Q9NRD1	9	15	44	293	33933	6.1	F-box only protein 6
1894	UPI0001E8F1C3	9	34	54.9	244	26976	6.5	UPI0001E8F1C3 UniRef100 entry
1894	O94903	9	34	48.7	275	30344	7.5	Proline synthase co-transcribed bacterial log protein
1894	D3DSW3	9	34	43.2	310	34042	8.4	Proline synthetase co-transcribed log (Bacterial), isoform CRA_b
1895	B4DXQ8	9	30	44.6	260	29132	9.6	cDNA FLJ52940, highly similar to Mortality factor 4-like protein 2
1895	B3KWX6	9	30	43	270	30451	9.5	cDNA FLJ44127 fis, clone THYMU2006420, highly similar to Mortality factor 4-like protein 2
1895	Q53EY5	9	30	40.3	288	32312	9.7	MORF-related gene X variant (Fragment)
1895	Q15014	9	30	40.3	288	32308	9.7	Mortality factor 4-like protein 2
1896	Q00688	9	42	42.9	224	25177	9.3	Peptidyl-prolyl cis-trans isomerase FKBP3
1896	Q53GD8	9	42	42.9	224	25204	9.4	FK506-binding protein 3 variant (Fragment)
1897	Q5HYL6	9	26	42.9	352	39555	5.2	Putative uncharacterized protein DKFZp686E1899
1897	Q9NYL9	9	26	42.9	352	39595	5.2	Tropomodulin-3
1898	P26373	9	61	42.2	211	24261	11.7	60S ribosomal protein L13
1898	Q6NZ55	9	61	42.2	211	24265	11.7	60S ribosomal protein L13
1899	Q96C23	9	20	41.5	342	37766	6.7	Aldose 1-epimerase
1900	B9A044	9	19	41.6	233	26816	7.9	Uncharacterized protein
1900	B8ZZ50	9	19	41.1	236	27219	7.2	Uncharacterized protein
1900	Q59FE1	9	19	39.9	243	27829	7.5	Eukaryotic translation initiation factor 4E member 2 variant (Fragment)
1900	O60573	9	19	39.6	245	28362	8.9	Eukaryotic translation initiation factor 4E type 2
1901	O95825	9	14	40.7	349	38697	5.8	Quinone oxidoreductase-like protein 1
1902	O76071	9	17	40.4	339	37840	5	Probable cytosolic iron-sulfur protein assembly protein CIAO1
1903	Q9BUP3	9	12	40.1	242	27049	8.4	Oxidoreductase HTATIP2
1904	Q49AR2	9	12	39.8	442	49967	4.8	UPF0489 protein C5orf22
1905	Q9UKD2	9	18	39.7	239	27560	8.3	mRNA turnover protein 4 log
1906	O76027	9	16	39.1	345	38364	5.8	Annexin A9
1907	A8K335	9	41	38.4	318	36047	7.4	cDNA FLJ76254, highly similar to sapiens gamma-glutamyl hydrolase (GGH), mRNA
1907	Q92820	9	41	38.4	318	35964	7.1	Gamma-glutamyl hydrolase
1908	Q4G0F5	9	12	37.8	336	39155	7.3	Vacuolar protein sorting-associated protein 26B
1909	Q9NP79	9	17	36.8	307	33879	6.3	Vacuolar protein sorting-associated protein VTA1 log
1910	P36952	9	10	35.7	375	42100	6	Serpin B5
1911	B4DVB4	9	14	35.6	351	38003	6.4	cDNA FLJ54796, highly similar to sapiens selenocysteine lyase (SCLY), mRNA
1911	E7ESG3	9	14	35.6	351	38033	6.4	Uncharacterized protein
1911	Q96I15	9	14	28.1	445	48149	7.1	Selenocysteine lyase
1912	B2RAT6	9	15	35.4	379	43347	7	Galactose-1-phosphate uridylyltransferase
1912	P07902	9	15	35.4	379	43363	7	Galactose-1-phosphate uridylyltransferase
1913	O43765	9	22	35.1	313	34063	4.9	Small glutamine-rich tetratricopeptide repeat-containing protein alpha
1914	Q15814	9	15	35	346	39248	5.7	Tubulin-specific chaperone C
1915	Q2TU34	9	17	34.9	338	36814	7	Fructose-1,6-bisphosphatase 1

Table S1. Proteins identified in the OVCAR-3 tumor supernatant by two or more unique peptides.

Protein or Protein Group	Accession Number	Unique Peptide Count	Spectrum Count	Sequence Coverage (%)	Length	MolWt	pI	Descriptive Name
1916	Q53EY9	9	12	34.7	403	44478	7	F-box only protein 22 isoform a variant (Fragment)
1916	Q8NEZ5	9	12	34.7	403	44508	7	F-box only protein 22
1917	Q96S82	9	16	37.4	380	40510	5.1	Ubiquitin-like protein 7
1917	D3DW56	9	16	33.8	420	45137	5.4	Ubiquitin-like 7 (Bone marrow stromal cell-derived), isoform CRA_a
1918	UPI0000D4E5C4	9	16	38.5	314	34684	7.2	Aflatoxin B1 aldehyde reductase member 2 (EC 1.-.-.-) (AFB1-AR 1) (Aldoketoreductase 7).
1918	O43488	9	16	33.7	359	39589	7.2	Aflatoxin B1 aldehyde reductase member 2
1919	O95630	9	20	33.5	424	48077	6.3	STAM-binding protein
1920	P28289	9	16	33.4	359	40569	5.1	Tropomodulin-1
1921	Q96ER3	9	24	31.9	474	53558	4.5	Protein SAAL1
1922	P08397	9	15	30.5	361	39330	7.2	Porphobilinogen deaminase
1923	P55036	9	22	30.5	377	40737	4.8	26S proteasome non-ATPase regulatory subunit 4
1924	Q99816	9	20	30	390	43944	6.4	Tumor susceptibility gene 101 protein
1925	Q5QPL9	9	31	36.7	237	24665	10.5	RNA binding protein, autoantigenic (HnRNP-associated with lethal yellow log (Mouse)) (Fragment)
1925	A8K4T9	9	31	29.9	291	30398	9.5	cDNA FLJ77421, highly similar to sapiens autoantigen p542 mRNA
1925	Q9UKM9	9	31	28.4	306	32463	9.2	RNA-binding protein Raly
1925	Q53GL6	9	31	28.3	307	32550	9.2	RNA binding protein (Autoantigenic, hnRNP-associated with lethal yellow) long isoform variant (Fragment)
1926	O94888	9	15	29.4	489	54862	5.2	UBX domain-containing protein 7
1927	P18754	9	13	29.9	421	44969	7.5	Regulator of chromosome condensation
1927	E9PAT9	9	13	28.8	438	46753	8.1	Uncharacterized protein
1927	Q6NT97	9	13	27.9	452	48146	8.2	Regulator of chromosome condensation 1
1928	P50613	9	13	28	346	39038	8.5	Cyclin-dependent kinase 7
1929	Q13619	9	20	27.9	759	87680	8.1	Cullin-4A
1930	Q9NR46	9	11	28.1	395	43974	6	Endophilin-B2
1930	B7ZC38	9	11	27.8	400	44361	5.8	SH3-domain GRB2-like endophilin B2
1930	Q9NR46-2	9	11	27.5	404	44766	5.6	Isoform 2 of Endophilin-B2
1931	Q14790-2	9	13	28.2	464	53769	5.2	Isoform 2 of Caspase-8
1931	Q14790	9	13	27.3	479	55391	5.1	Caspase-8
1931	Q14790-4	9	13	26.4	496	57701	5.4	Isoform 4 of Caspase-8
1931	UPI0000456ECD	9	13	24.3	538	61836	5.3	caspase-8 isoform G precursor
1931	Q14790-9	9	13	24.3	538	61864	5.3	Isoform 9 of Caspase-8
1932	Q86U75	9	13	27.3	619	67017	6	Dihydropyrimidinase-like 2
1933	B3KM43	9	16	25.1	621	68798	6.1	cDNA FLJ10228 fis, clone HEMBB1000119, highly similar to N-acetylserotonin O-methyltransferase-like protein
1933	O95671	9	16	25.1	621	68857	6.1	N-acetylserotonin O-methyltransferase-like protein
1934	A8K8C2	9	13	23.7	527	59332	6.8	cDNA FLJ76932, highly similar to sapiens tubulin-specific chaperone e (TBCE), mRNA
1934	Q15813	9	13	23.7	527	59346	6.8	Tubulin-specific chaperone E
1934	B7Z3P1	9	13	21.6	578	64852	7.3	cDNA FLJ59210, highly similar to Tubulin-specific chaperone E
1935	Q59GY3	9	23	29	279	31865	11	Arginine/serine-rich splicing factor 6 variant (Fragment)
1935	Q13247-3	9	23	24.2	335	38419	11	Isoform SRP55-3 of Serine/arginine-rich splicing factor 6
1935	A8K588	9	23	23.5	344	39488	11.4	cDNA FLJ76823, highly similar to sapiens splicing factor, arginine/serine-rich 6 (SFRS6), mRNA
1935	Q13247	9	23	23.5	344	39587	11.4	Serine/arginine-rich splicing factor 6
1936	B4DVY1	9	34	25.9	499	58141	5.7	cDNA FLJ51552, highly similar to Eukaryotic translation initiation factor 3 subunit 7
1936	B4DTF8	9	34	24.2	533	62040	5.6	cDNA FLJ53917, highly similar to Eukaryotic translation initiation factor 3 subunit 7
1936	A8MWD3	9	34	23.5	548	63903	6.2	Uncharacterized protein
1936	O15371	9	34	23.5	548	63973	6	Eukaryotic translation initiation factor 3 subunit D
1937	P19474	9	12	23.2	475	54170	6.4	E3 ubiquitin-protein ligase TRIM21
1938	Q9H074-2	9	22	27.8	400	45631	4.5	Isoform 2 of Polyadenylate-binding protein-interacting protein 1
1938	Q9H074	9	22	23.2	479	53525	4.8	Polyadenylate-binding protein-interacting protein 1
1939	Q96NY8	9	17	21	510	55454	5.4	Poliovirus receptor-related protein 4
1940	Q9UQB8-5	9	23	22.1	520	57445	8.8	Isoform 5 of Brain-specific angiogenesis inhibitor 1-associated protein 2
1940	Q9UQB8-4	9	23	22.1	521	57359	8.9	Isoform 4 of Brain-specific angiogenesis inhibitor 1-associated protein 2
1940	Q9UQB8-6	9	23	22	522	57430	8.9	Isoform 6 of Brain-specific angiogenesis inhibitor 1-associated protein 2
1940	Q9UQB8-2	9	23	21.5	534	59014	9	Isoform 2 of Brain-specific angiogenesis inhibitor 1-associated protein 2
1940	Q9UQB8	9	23	20.8	552	60868	8.9	Brain-specific angiogenesis inhibitor 1-associated protein 2
1941	Q9Y2X3	9	29	19.8	529	59578	8.9	Nucleolar protein 58
1942	B7Z5E4	9	14	23.1	445	50431	8.9	cDNA FLJ54614, highly similar to Vacuolar protein sorting-associated protein 45
1942	B7Z360	9	14	19.3	534	61107	8.3	cDNA FLJ54353, highly similar to Vacuolar protein sorting-associated protein 45
1942	Q9NRW7	9	14	18.1	570	65077	8.2	Vacuolar protein sorting-associated protein 45
1943	Q9BTT9	9	15	21.1	673	76483	6.5	GYS1 protein
1943	P13807	9	15	19.3	737	83786	6.2	Glycogen [starch] synthase, muscle
1943	Q53ER0	9	15	19.3	737	83858	6.1	Glycogen synthase 1 (Muscle) variant (Fragment)
1944	Q0EFC6	9	16	20.7	545	62135	9.1	HSR1 protein
1944	E9PBB2	9	16	20.1	561	64258	9.5	Uncharacterized protein
1944	B4DYK6	9	16	18.7	605	68256	5.5	cDNA FLJ56887, highly similar to sapiens guanine nucleotide binding protein-like 1 (GNL1), mRNA
1944	P36915	9	16	18.6	607	68661	5.8	Guanine nucleotide-binding protein-like 1
1945	B4E1E2	9	21	18.5	661	74854	8	cDNA FLJ61530, highly similar to Hepatocyte growth factor-regulated tyrosine kinase substrate
1945	O14964	9	21	15.7	777	86192	6.2	Hepatocyte growth factor-regulated tyrosine kinase substrate
1946	O43278-2	9	16	18.5	513	56885	6.2	Isoform 2 of Kunitz-type protease inhibitor 1
1946	O43278	9	16	18	529	58398	6.3	Kunitz-type protease inhibitor 1
1947	Q9BTE3-2	9	10	17.2	640	72749	5.8	Isoform 2 of Mini-chromosome maintenance complex-binding protein
1947	Q9BTE3	9	10	17.1	642	72980	5.9	Mini-chromosome maintenance complex-binding protein
1948	Q5RFA0	9	19	16.4	590	66219	5.1	Negative elongation factor D
1948	Q8IXH7	9	19	16.4	590	66247	5.1	Negative elongation factor C/D
1949	P05556	9	14	16.3	798	88415	5.4	Integrin beta-1
1950	Q8TE77	9	13	16.2	659	72996	5.3	Protein phosphatase Slingshot log 3
1951	B2RAU8	9	120	16	699	73604	9.4	cDNA, FLJ95131, highly similar to sapiens nucleolar and coiled-body phosphoprotein 1 (NOLC1), mRNA
1951	Q14978	9	120	16	699	73603	9.5	Nucleolar and coiled-body phosphoprotein 1
1951	Q14978-2	9	120	15.8	709	74747	9.5	Isoform Beta of Nucleolar and coiled-body phosphoprotein 1
1952	Q96ST2-2	9	20	22.9	494	56109	5.4	Isoform 2 of Protein IWS1 log
1952	Q96ST2-3	9	20	18.5	612	69108	4.9	Isoform 3 of Protein IWS1 log
1952	C9J221	9	20	18.5	612	69078	4.9	Uncharacterized protein
1952	B4DGM5	9	20	15.5	729	82122	4.8	cDNA FLJ53983, highly similar to IWS1 log
1952	E7EX51	9	20	14.7	771	86674	4.7	Uncharacterized protein
1952	Q96ST2	9	20	13.8	819	91955	4.7	Protein IWS1 log

Table S1. Proteins identified in the OVCAR-3 tumor supernatant by two or more unique peptides.

Protein or Protein Group	Accession Number	Unique Peptide Count	Spectrum Count	Sequence Coverage (%)	Length	MolWt	pI	Descriptive Name
1953	O95834	9	10	19	649	70679	6.3	Echinoderm microtubule-associated protein-like 2
1953	B7Z3I2	9	10	15.5	796	86471	6.9	cDNA FLJ59545, highly similar to Echinoderm microtubule-associated protein-like 2
1953	B7Z3Q9	9	10	15.1	815	88589	6.9	cDNA FLJ56452, highly similar to Echinoderm microtubule-associated protein-like 2
1954	Q9BWU0	9	19	14.9	796	88814	5.2	Kanadaptin
1955	UPI0001DAFE3F	9	13	14.2	869	97977	7.5	dynamin-2 isoform 5
1955	P50570-2	9	13	14.2	866	97652	7.6	Isoform 2 of Dynamin-2
1955	P50570	9	13	14.1	870	98064	7.5	Dynamin-2
1956	O75146	9	11	14	1068	119388	6.7	Huntingtin-interacting protein 1-related protein
1957	O75150-4	9	9	14.9	901	102046	6.5	Isoform 4 of E3 ubiquitin-protein ligase BRE1B
1957	A8K6K1	9	9	13.4	1000	113549	6.3	cDNA FLJ78667, highly similar to sapiens ring finger protein 40 (RNF40), transcript variant 1, mRNA
1957	O75150	9	9	13.4	1001	113650	6.2	E3 ubiquitin-protein ligase BRE1B
1958	Q9Y6K1	9	18	12.5	912	101858	6.6	DNA (cytosine-5)-methyltransferase 3A
1959	UPI000023B84F	9	10	11.4	980	110311	5.4	liprin-beta-1 isoform 4
1959	UPI000023B84D	9	10	11.1	1005	113195	5.7	liprin-beta-1 isoform 1
1960	Q96F88	9	10	10.6	1024	114681	9.2	Processing of 1, ribonuclease P/MRP subunit (S. cerevisiae)
1960	Q99575	9	10	10.6	1024	114709	9.2	Ribonucleases P/MRP protein subunit POP1
1961	B7Z6F7	9	11	10.5	1274	141132	6.1	cDNA FLJ61705, highly similar to Symplekin
1961	Q92797	9	11	10.5	1274	141148	6.1	Symplekin
1962	E7EN22	9	12	10.9	1372	150659	6.4	Ubiquitin carboxyl-terminal hydrolase
1962	E9PEG8	9	12	10.5	1419	156035	6.4	Uncharacterized protein
1962	B4DGT3	9	12	10.5	1419	156051	6.4	Ubiquitin carboxyl-terminal hydrolase
1962	A5PKX8	9	12	10.3	1447	158726	7	Ubiquitin carboxyl-terminal hydrolase (Fragment)
1962	A5PL36	9	12	10.3	1449	158974	7	Ubiquitin carboxyl-terminal hydrolase (Fragment)
1963	B3KMR5	9	13	10.3	1297	143672	8.8	cDNA FLJ12434 fis, clone NT2RM1000037, highly similar to sapiens KIAA0690 protein
1963	Q5JTH9	9	13	10.3	1297	143702	8.8	RRP12-like protein
1964	E7EQT4	9	16	9.6	1094	122261	5.6	Uncharacterized protein
1964	B4DQZ7	9	16	9.6	1096	122558	5.7	cDNA FLJ50838, highly similar to Apoptotic chromatin condensation inducer in thenucleus (Fragment)
1964	UPI0001B8380A	9	16	8.1	1301	147388	6.7	apoptotic chromatin condensation inducer in the nucleus isoform 3
1964	B2RTT4	9	16	7.9	1328	150583	6.4	ACIN1 protein
1964	Q9UKV3	9	16	7.8	1341	151861	6.4	Apoptotic chromatin condensation inducer in the nucleus
1965	Q5VZ89	9	12	7.1	1673	186856	6.9	DENN domain-containing protein 4C
1966	Q13535-2	9	21	5.4	2580	294217	7.4	Isoform 2 of Serine/threonine-protein kinase ATR
1966	Q13535	9	21	5.3	2644	301365	7.4	Serine/threonine-protein kinase ATR
1966	Q13535-3	9	21	5.3	2610	297478	7.5	Isoform 3 of Serine/threonine-protein kinase ATR
1967	B7WNZ6	9	18	4.3	2752	301570	9.1	Uncharacterized protein
1967	Q9Y520-3	9	18	4.3	2701	295829	9	Isoform 3 of Protein PRRC2C
1967	E9PEL0	9	18	4.3	2702	296059	9	Uncharacterized protein
1967	UPI0000E265EC	9	18	4.2	2817	308606	9.1	protein BAT2-like 2
1967	Q9Y520-6	9	18	4.2	2753	301668	9.1	Isoform 6 of Protein PRRC2C
1967	Q9Y520-4	9	18	4.2	2818	308703	9.1	Isoform 4 of Protein PRRC2C
1967	E7EPN9	9	18	4.2	2819	308776	9.1	Uncharacterized protein
1967	Q9Y520-5	9	18	4.1	2850	312732	9.1	Isoform 5 of Protein PRRC2C
1967	E9PF21	9	18	4.1	2850	312706	9.1	Uncharacterized protein
1967	UPI0001AE797B	9	18	4	2896	316911	9.1	HBxAg transactivated protein 2
1967	Q9Y520-7	9	18	4	2899	317178	9.1	Isoform 7 of Protein PRRC2C
1967	Q9Y520	9	18	4	2897	317008	9.1	Protein PRRC2C
1967	E7EX58	9	18	4	2898	317081	9.1	Uncharacterized protein
1968	Q9BYK8	9	13	4	2649	294649	7.5	Peroxisomal proliferator-activated receptor A-interacting complex 285 kDa protein
1969	Q9NU22	9	13	2.5	5596	632827	5.7	Midasin
1970	Q9UPN1	8	22	75.2	294	33774	5.3	Serine/threonine-protein phosphatase (Fragment)
1970	P36873	8	22	68.4	323	36984	6.5	Serine/threonine-protein phosphatase PP1-gamma catalytic subunit
1970	Q3U7K1	8	22	68.4	323	37033	6.5	Serine/threonine-protein phosphatase
1970	P63087	8	22	68.4	323	36984	6.5	Serine/threonine-protein phosphatase PP1-gamma catalytic subunit
1970	P63087-2	8	22	65.6	337	38504	6.1	Isoform Gamma-2 of Serine/threonine-protein phosphatase PP1-gamma catalytic subunit
1970	P36873-2	8	22	65.6	337	38518	6.1	Isoform Gamma-2 of Serine/threonine-protein phosphatase PP1-gamma catalytic subunit
1971	P68036	8	222	66.9	154	17862	8.5	Ubiquitin-conjugating enzyme E2 L3 Ubiquitin-conjugating enzyme E2 L3
1972	P62330	8	43	65.7	175	20082	9	ADP-ribosylation factor 6 ADP-ribosylation factor 6
1972	Q5U025	8	43	65.7	175	20211	9	ADP-ribosylation factor 6
1973	P62841	8	108	64.8	145	17040	10.4	40S ribosomal protein S15 40S ribosomal protein S15
1974	Q99JZ4	8	20	64.1	198	22399	6.7	SAR1 gene log A (S. cerevisiae)
1974	Q9NR31	8	20	64.1	198	22367	6.7	GTP-binding protein SAR1a
1975	P45878	8	56	63.6	140	15344	8.9	Peptidyl-prolyl cis-trans isomerase FKBP2
1975	P26885	8	56	62.7	142	15649	9.1	Peptidyl-prolyl cis-trans isomerase FKBP2
1976	Q3UGM5	8	15	62.7	225	26002	5.8	Putative uncharacterized protein
1976	Q9Y3A3	8	15	62.7	225	26032	5.8	Mps one binder kinase activator-like 3 Mps one binder kinase activator-like 3
1977	P83876	8	93	62	142	16786	5.8	Thioredoxin-like protein 4A Thioredoxin-like protein 4A
1978	P47813	8	37	61.1	144	16460	5.2	Eukaryotic translation initiation factor 1A, X-chromosomal Eukaryotic translation initiation factor 1A, X-chromosomal
1979	E9QKZ3	8	142	61.4	202	22299	6.2	Uncharacterized protein
1979	E7ETK2	8	142	60.5	205	22595	7.2	Uncharacterized protein
1979	P62820	8	142	60.5	205	22678	6.2	Ras-related protein Rab-1A Ras-related protein Rab-1A
1980	UPI0000110661	8	22	67.5	169	19169	4.8	SERINE/THREONINE PHOSPHATASE 2B
1980	Q4T3K0	8	22	67.5	169	19129	4.8	Chromosome undetermined SCAF10022, whole genome shotgun sequence. (Fragment)
1980	P63098	8	22	67.1	170	19300	4.8	Calcineurin subunit B type 1 Calcineurin subunit B type 1
1980	D3YTA9	8	22	60.3	189	21445	5	Uncharacterized protein
1981	Q3T0M0	8	37	55.4	186	20917	7	Vacuolar protein sorting-associated protein 29
1981	Q9UBQ0-2	8	37	55.4	186	20927	7	Isoform 2 of Vacuolar protein sorting-associated protein 29
1982	Q9H8S9	8	74	51.9	216	25080	7	Mps one binder kinase activator-like 1B Mps one binder kinase activator-like 1B
1983	Q15286	8	60	48.3	201	23025	8.3	Ras-related protein Rab-35 Ras-related protein Rab-35
1984	P62851	8	131	47.2	125	13742	10.1	40S ribosomal protein S25 40S ribosomal protein S25
1985	D3Z3R2	8	89	46.7	184	21279	10.1	Uncharacterized protein
1985	Q9CPR4	8	89	46.7	184	21423	10.2	60S ribosomal protein L17
1985	Q3T025	8	89	46.7	184	21397	10.2	60S ribosomal protein L17
1985	P18621	8	89	46.7	184	21397	10.2	60S ribosomal protein L17 60S ribosomal protein L17
1985	Q505B1	8	89	46.5	185	21481	10.2	Rpl17 protein (Fragment)
1985	UPI000024FCB4	8	89	44.6	193	22275	10.2	UPI000024FCB4 UniRef100 entry
1985	Q80V08	8	89	44.3	194	22434	10.3	Rpl17 protein (Fragment)
1985	UPI0001E6B60E	8	89	39.6	217	25035	9.8	PREDICTED: 60S ribosomal protein L17-like isoform 2

Table S1. Proteins identified in the OVCAR-3 tumor supernatant by two or more unique peptides.

Protein or Protein Group	Accession Number	Unique Peptide Count	Spectrum Count	Sequence Coverage (%)	Length	MolWt	pI	Descriptive Name
1985	UPI0000D4CB8E	8	89	37.7	228	26373	10.1	RPL17-C18orf32 protein isoform 1
1985	UPI0001E6B61E	8	89	32.2	267	30544	10	PREDICTED: 60S ribosomal protein L17-like
1986	Q03958	8	19	45.7	127	14455	8.9	Prefoldin subunit 6
1986	O15212	8	19	45	129	14583	8.9	Prefoldin subunit 6
1987	Q9D883	8	18	40.2	239	27815	8.8	Splicing factor U2AF 35 kDa subunit
1987	Q01081	8	18	40	240	27872	8.8	Splicing factor U2AF 35 kDa subunit
1988	P61964	8	16	39.5	334	36589	8.3	WD repeat-containing protein 5 WD repeat-containing protein 5
1988	Q3UNQ3	8	16	39.5	334	36574	8.3	Putative uncharacterized protein
1989	P84098	8	61	31.6	196	23466	11.5	60S ribosomal protein L19 60S ribosomal protein L19
1990	Q99KV1	8	12	28.2	358	40555	6.3	DnaJ log subfamily B member 11
1990	Q9UBS4	8	12	28.2	358	40514	6.2	DnaJ log subfamily B member 11
1991	Q16630-3	8	67	22.6	478	52326	6.4	Isoform 3 of Cleavage and polyadenylation specificity factor subunit 6
1991	C9JGC2	8	67	22.5	479	52357	6.4	Uncharacterized protein
1991	Q6NVF9	8	67	19.6	551	59153	7.2	Cleavage and polyadenylation specificity factor subunit 6
1991	Q16630	8	67	19.6	551	59210	7.2	Cleavage and polyadenylation specificity factor subunit 6
1991	Q16630-2	8	67	18.4	588	63471	7.7	Isoform 2 of Cleavage and polyadenylation specificity factor subunit 6
1992	Q66K91	8	100	26	231	23656	10.9	Small nuclear ribonucleoprotein polypeptides B and B1
1992	Q5XPV6	8	100	26	231	23657	10.7	Small nuclear ribonucleoprotein polypeptide B
1992	P27048	8	100	26	231	23656	10.9	Small nuclear ribonucleoprotein-associated protein B
1992	B4DVS0	8	100	25.6	234	24088	10.8	Small nuclear ribonucleoprotein polypeptides B and B1, isoform CRA_c
1992	P63162	8	100	25	240	24614	11.2	Small nuclear ribonucleoprotein-associated protein N Small nuclear ribonucleoprotein-associated protein N
1992	P14678	8	100	25	240	24610	11.2	Small nuclear ribonucleoprotein-associated proteins B and B'
1992	E7ERB2	8	100	24.8	242	27210	9.4	Uncharacterized protein
1992	Q9UIS4	8	100	24.7	243	27267	9.4	Small nuclear ribonucleoprotein B'
1992	B3KVR1	8	100	24.6	244	25076	11	cDNA FLJ41124 fis, clone BRACE2014850, highly similar to Small nuclear ribonucleoprotein-associated protein N
1992	Q15182	8	100	21.1	285	29671	10.1	SNRPB protein
1992	A8MT02	8	100	20.9	287	29871	10.5	Uncharacterized protein
1992	E7ENP4	8	100	20.9	287	29862	10.2	Uncharacterized protein
1992	P14678-3	8	100	20.8	289	30032	10.2	Isoform SM-B1 of Small nuclear ribonucleoprotein-associated proteins B and B'
1993	Q59H57	8	193	36	300	31994	9.5	Fusion (Involved in t(12;16) in malignant liposarcoma) isoform a variant (Fragment)
1993	B4DR70	8	193	25.2	429	44812	9	cDNA FLJ58049, highly similar to RNA-binding protein FUS
1993	Q8CFQ9	8	193	20.9	517	52602	9.4	Fusion, derived from t(12;16) malignant liposarcoma (Human)
1993	Q3USY4	8	193	20.8	518	52630	9.3	Putative uncharacterized protein
1993	P56959	8	193	20.8	518	52673	9.4	RNA-binding protein FUS
1993	UPI000040A0A0	8	193	20.7	522	53198	9.4	RNA-binding protein FUS isoform 3
1993	A8K4H1	8	193	20.6	525	53411	9.4	cDNA FLJ78268, highly similar to sapiens fusion (involved in t(12;16) in malignant liposarcoma), transcript variant 1, mRNA
1993	P35637-2	8	193	20.6	525	53355	9.4	Isoform Short of RNA-binding protein FUS
1993	E7EUX0	8	193	20.6	524	53312	9.4	Uncharacterized protein
1993	Q8TBR3	8	193	20.5	526	53400	9.4	Fusion (Involved in t(12;16) in malignant liposarcoma)
1993	Q13344	8	193	20.5	528	53377	9.4	Fus-like protein (Fragment)
1993	P35637	8	193	20.5	526	53426	9.4	RNA-binding protein FUS
1994	Q12996	8	24	20.4	717	82922	8.1	Cleavage stimulation factor subunit 3
1994	Q99LI7	8	24	20.4	717	82877	8.1	Cleavage stimulation factor subunit 3
1994	Q3TXN6	8	24	20.4	715	82582	8.2	Putative uncharacterized protein (Fragment)
1995	Q7M6Y3-2	8	43	20.6	597	64648	9	Isoform 2 of Phosphatidylinositol-binding clathrin assembly protein
1995	Q7M6Y3-3	8	43	20.3	605	65623	9	Isoform 3 of Phosphatidylinositol-binding clathrin assembly protein
1995	Q7M6Y3-4	8	43	20.2	610	66184	8.8	Isoform 4 of Phosphatidylinositol-binding clathrin assembly protein
1995	Q13492-3	8	43	20.2	610	66393	8.8	Isoform 3 of Phosphatidylinositol-binding clathrin assembly protein
1995	B5BU72	8	43	20.2	610	66394	8.7	Phosphatidylinositol-binding clathrin assembly protein isoform 2
1995	Q13492-2	8	43	19.5	632	68764	7.9	Isoform 2 of Phosphatidylinositol-binding clathrin assembly protein
1995	UPI00016395DD	8	43	19.1	645	70037	8.2	UPI00016395DD UniRef100 entry
1995	A8K5U9	8	43	18.9	652	70756	7.6	cDNA FLJ75056, highly similar to sapiens phosphatidylinositol binding clathrin assembly protein (PICALM), mRNA
1995	Q7M6Y3-6	8	43	18.9	652	70568	7.9	Isoform 6 of Phosphatidylinositol-binding clathrin assembly protein
1995	Q13492	8	43	18.9	652	70755	7.9	Phosphatidylinositol-binding clathrin assembly protein
1995	Q7M6Y3	8	43	18.6	660	71543	7.9	Phosphatidylinositol-binding clathrin assembly protein
1995	A8MX97	8	43	18.6	660	71739	8.2	Uncharacterized protein
1995	Q570Z8	8	43	18.2	674	72927	9	MKIAA4114 protein (Fragment)
1995	Q4LE54	8	43	17.1	721	77397	8.4	PICALM variant protein (Fragment)
1996	Q9Z1K5	8	12	17.7	555	64017	5.1	Protein ariadne-1 log
1996	Q9Y4X5	8	12	17.6	557	64118	5.1	Protein ariadne-1 log
1997	Q9NQX3	8	10	15.1	736	79748	5.4	Gephyrin
1997	E9QKJ1	8	10	14.4	769	83282	5.6	Uncharacterized protein
1998	A2AT37	8	10	7.6	1269	147552	5.6	UPF2 regulator of nonsense transcripts log (Yeast)
1998	Q9HAU5	8	10	7.6	1272	147810	5.7	Regulator of nonsense transcripts 2
1999	Q8CGF7-2	8	13	7.4	1079	121577	8.5	Isoform 2 of Transcription elongation regulator 1
1999	O14776-2	8	13	7.4	1077	121690	8.5	Isoform 2 of Transcription elongation regulator 1
1999	O14776	8	13	7.3	1098	123901	8.6	Transcription elongation regulator 1
1999	Q8CGF7	8	13	7.3	1100	123788	8.6	Transcription elongation regulator 1
2000	B7Z6H4	8	8	6.6	1369	153509	8.5	DNA-directed RNA polymerase
2000	B2RXC6	8	8	6.5	1390	155737	8.5	DNA-directed RNA polymerase
2000	O14802	8	8	6.5	1390	155641	8.5	DNA-directed RNA polymerase III subunit RPC1
2001	Q5T321	8	23	2.8	2943	327548	6.2	Neurobeachin
2001	UPI00004566E0	8	23	2.8	2946	327863	6.2	Protein neurobeachin (Lysosomal-trafficking regulator 2) (Protein BCL8B).
2001	Q9EPN1-4	8	23	2.8	2931	326153	6.2	Isoform 4 of Neurobeachin
2001	Q9EPN1-3	8	23	2.8	2904	323201	6.2	Isoform 3 of Neurobeachin
2001	Q9EPN1	8	23	2.8	2936	326742	6.2	Neurobeachin
2001	Q8NFP9	8	23	2.8	2946	327821	6.2	Neurobeachin
2002	P14069	8	116	91	89	10051	5.5	Protein S100-A6
2003	P99027	8	28	85.2	115	11651	4.5	60S acidic ribosomal protein P2
2004	P62774	8	32	72.9	118	12861	5.5	Myotrophin
2005	Q3TIH9	8	82	66.9	154	17863	8.2	Ubiquitin carrier protein
2006	O70251	8	48	63.1	225	24694	4.7	Elongation factor 1-beta
2007	P13832	8	51	62.2	172	19895	4.8	Myosin regulatory light chain RLC-A
2008	Q9D2M8	8	190	61.4	145	16367	8	Ubiquitin-conjugating enzyme E2 variant 2
2009	A7VJ98	8	19	56.3	142	16722	5.4	Glia maturation factor beta
2009	Q9CQI3	8	19	56.3	142	16723	5.2	Glia maturation factor beta
2009	Q80X87	8	19	55.9	143	16824	5.2	Glia maturation factor, beta

Table S1. Proteins identified in the OVCAR-3 tumor supernatant by two or more unique peptides.

Protein or Protein Group	Accession Number	Unique Peptide Count	Spectrum Count	Sequence Coverage (%)	Length	MolWt	pI	Descriptive Name
2010	P22935	8	626	55.8	138	15746	5.7	Cellular retinoic acid-binding protein 2
2011	P08228	8	18	55.2	154	15943	6.5	Superoxide dismutase [Cu-Zn]
2012	P11352	8	23	51.7	201	22179	7.2	Glutathione peroxidase 1
2012	Q5RJH8	8	23	51.7	201	22292	7.2	Glutathione peroxidase
2013	Q3TF08	8	21	71.8	174	20201	6	Putative uncharacterized protein (Fragment)
2013	Q00724	8	21	62.2	201	23206	6	Retinol-binding protein 4
2013	E9QQ51	8	21	51	245	28405	8	Uncharacterized protein
2014	Q9D0B6	8	21	49.5	198	22223	4.5	UPF0368 protein Cxorf26 homolog
2015	Q7TMS6	8	12	48.8	209	23662	7.4	Glycolipid transfer protein
2015	Q9JL62	8	12	48.8	209	23690	7.4	Glycolipid transfer protein
2016	Q8VHN8	8	18	48.8	211	23414	9.3	Protein syndesmos
2016	Q8VHN8-2	8	18	43.5	237	26459	9.6	Isoform 2 of Protein syndesmos
2017	P99026	8	27	48.5	264	29116	5.7	Proteasome subunit beta type-4
2018	Q9CPU0	8	72	47.3	184	20810	5.5	Lactoylglutathione lyase
2019	P22599	8	71	47.2	413	45975	5.5	Alpha-1-antitrypsin 1-2
2020	P62748	8	13	47.2	193	22338	5.5	Hippocalcin-like protein 1
2021	Q63829	8	10	47.2	195	22037	5.6	COMM domain-containing protein 3
2022	Q80UW8	8	20	46.7	210	24570	6	DNA-directed RNA polymerases I, II, and III subunit RPABC1
2023	Q6P069-2	8	16	50.3	183	20296	5.3	Isoform 2 of Sorcin
2023	Q6P069	8	16	46.5	198	21627	5.6	Sorcin
2024	P63073	8	19	45.2	217	25053	6.1	Eukaryotic translation initiation factor 4E
2025	Q8K3C3	8	22	44.7	190	21537	5	Protein LZIC
2026	Q9CR09	8	13	44.3	167	19481	7.4	Ubiquitin-fold modifier-conjugating enzyme 1
2027	Q9D859	8	46	43.8	192	21464	8.5	Putative uncharacterized protein
2028	D3YX50	8	29	43.1	160	18609	10.4	MCG4465
2028	UPI0000D668F8	8	29	43.1	160	18570	10.5	PREDICTED: 60S ribosomal protein L21-like
2028	UPI000002570F	8	29	43.1	160	18606	10.5	PREDICTED: 60S ribosomal protein L21-like
2028	Q9CQM8	8	29	43.1	160	18579	10.5	MCG120646
2029	P35980	8	20	41.5	188	21645	11.8	60S ribosomal protein L18
2029	Q642K1	8	20	41.5	188	21644	11.8	Ribosomal protein L18
2030	P29699	8	153	41.4	345	37326	6.5	Alpha-2-HS-glycoprotein
2030	Q3UEK5	8	153	41.4	345	37280	6.5	Putative uncharacterized protein
2030	Q3TIU3	8	153	41.4	345	37298	6.4	Putative uncharacterized protein
2031	Q3THC1	8	18	40.5	222	24692	6.4	Proteasome (Prosome, macropain) 26S subunit, non-ATPase, 9
2031	Q9CR00	8	18	40.5	222	24720	6.4	26S proteasome non-ATPase regulatory subunit 9
2032	P45377	8	19	40.2	316	36121	6.4	Aldose reductase-related protein 2
2033	P58389	8	31	39.9	323	36710	6.4	Serine/threonine-protein phosphatase 2A activator
2034	P48774	8	13	39.7	224	26635	7.2	Glutathione S-transferase Mu 5
2035	Q64012-2	8	18	40.5	296	31169	9.4	Isoform 1 of RNA-binding protein Raly
2035	Q64012	8	18	38.5	312	33158	8.8	RNA-binding protein Raly
2036	Q9CY64	8	11	38	295	33525	7	Biliverdin reductase A
2037	Q08093	8	43	37.4	305	33156	7.6	Calponin-2
2038	P47963	8	31	37	211	24305	11.5	60S ribosomal protein L13
2038	UPI000024F513	8	31	37	211	24252	11.5	PREDICTED: 60S ribosomal protein L13-like
2039	P21550	8	125	36.9	434	47025	7.2	Beta-enolase
2039	Q4FK59	8	125	36.9	434	46998	6.7	Enolase
2040	P10605	8	43	36	339	37280	5.9	Cathepsin B
2040	Q3TVS6	8	43	36	339	37320	6	Putative uncharacterized protein
2041	P28063	8	16	35.9	276	30260	6.7	Proteasome subunit beta type-8
2042	UPI0001F7935A	8	19	36	278	30770	5.3	UPI0001F7935A UniRef100 entry
2042	Q8BVQ0	8	19	35.8	279	30929	5.3	Putative uncharacterized protein
2042	Q9ERF3	8	19	32.8	305	33773	5.4	WD repeat-containing protein 61
2043	Q3V471	8	69	50.5	186	19938	9.5	Putative uncharacterized protein (Fragment)
2043	P16110	8	69	35.6	264	27515	8.4	Galectin-3
2043	Q8C253	8	69	35.6	264	27415	8.5	Lectin, galactose binding, soluble 3
2044	Q62000	8	21	34.9	298	34012	5.7	Mimecan
2045	Q64726	8	12	34.9	307	35256	6.2	Zinc-alpha-2-glycoprotein
2045	Q9DBB7	8	12	34.9	307	35332	6.2	Zinc-alpha-2-glycoprotein 1
2046	Q3TXD3	8	17	31.5	406	43125	6.4	Putative uncharacterized protein
2046	Q62465	8	17	31.5	406	43097	6.4	Synaptic vesicle membrane protein VAT-1 homolog
2047	Q9CR26	8	14	28.8	309	33913	6.1	Vacuolar protein sorting-associated protein VTA1 homolog
2048	Q3UAZ7	8	33	33.1	181	20534	9.8	Putative uncharacterized protein (Fragment)
2048	Q9CT19	8	33	31.4	191	21736	9.9	Putative uncharacterized protein (Fragment)
2048	P30681	8	33	28.6	210	24162	7.3	High mobility group protein B2
2048	Q3U566	8	33	28.6	210	24176	7.3	MCG4647
2049	O35744	8	11	27.9	398	44458	5.7	Chitinase-3-like protein 3
2049	Q3UV87	8	11	27.9	398	44468	5.7	Putative uncharacterized protein
2050	Q9D020-1	8	11	30	297	33790	5.7	Isoform 1 of Cytosolic 5'-nucleotidase 3
2050	Q9D020	8	11	26.9	331	37252	6.7	Cytosolic 5'-nucleotidase 3
2051	Q62418-3	8	34	27.1	432	48341	4.9	Isoform 3 of Drebrin-like protein
2051	Q62418-2	8	34	27	433	48428	4.9	Isoform 2 of Drebrin-like protein
2051	Q62418	8	34	26.8	436	48700	4.9	Drebrin-like protein
2052	Q3UM23	8	8	25.9	456	49844	4.8	Putative uncharacterized protein
2052	Q91VI7	8	8	25.9	456	49817	4.8	Ribonuclease inhibitor
2053	UPI0001F7968F	8	14	26.6	482	52394	8.8	UPI0001F7968F UniRef100 entry
2053	Q3UAS7	8	14	26.6	482	51844	7.5	Putative uncharacterized protein (Fragment)
2053	Q3TXK9	8	14	26.5	483	52566	8.6	Putative uncharacterized protein (Fragment)
2053	P47791	8	14	25.6	500	53663	8	Glutathione reductase, mitochondrial
2054	Q61239	8	14	23.6	377	44013	4.9	Protein farnesyltransferase/geranylgeranyltransferase type-1 subunit alpha
2055	Q7TNV0	8	11	22.6	380	43159	6.9	Protein DEK
2056	Q3U0C4	8	17	20.3	454	50941	5.6	Putative uncharacterized protein
2056	Q9R111	8	17	20.3	454	51013	5.5	Guanine deaminase
2057	Q6A028	8	22	17.8	585	68996	6	Switch-associated protein 70
2058	Q9JMH6-2	8	14	21.8	499	54394	6.3	Isoform 2 of Thioredoxin reductase 1, cytoplasmic
2058	Q9JMH6	8	14	17.8	613	66934	7.4	Thioredoxin reductase 1, cytoplasmic
2059	Q08481-2	8	11	18.1	697	77917	6.7	Isoform 2 of Platelet endothelial cell adhesion molecule
2059	Q08481-3	8	11	17.6	716	80004	6.9	Isoform 3 of Platelet endothelial cell adhesion molecule
2059	Q08481	8	11	17.3	727	81263	6.7	Platelet endothelial cell adhesion molecule
2059	B1ARB3	8	11	17.2	732	81917	6.9	Platelet/endothelial cell adhesion molecule 1
2059	Q8CAW4	8	11	17.2	732	81987	7	Putative uncharacterized protein
2060	Q7TSV4	8	20	16.6	620	68748	6.1	Phosphoglucomutase-2
2061	Q3TCJ7	8	13	16.3	620	71764	7.2	Putative uncharacterized protein
2061	Q60710	8	13	16.1	627	72650	8	SAM domain and HD domain-containing protein 1
2061	UPI0001DD34BC	8	13	16	631	72860	7.3	UPI0001DD34BC UniRef100 entry
2061	E9Q0K6	8	13	15.5	651	74935	7.3	Uncharacterized protein
2062	Q3UAS4	8	38	14.3	545	59977	5.2	Putative uncharacterized protein
2062	Q3UE29	8	38	14.2	551	60584	5.3	Putative uncharacterized protein
2062	E9PZ00	8	38	14.2	551	60672	5.2	Uncharacterized protein
2062	Q8BFQ1	8	38	14.1	554	61051	5.2	Prosaposin, isoform CRA_a
2062	Q3U8C4	8	38	14.1	554	61023	5.2	Putative uncharacterized protein
2062	Q3U897	8	38	14.1	554	61027	5.3	Putative uncharacterized protein
2062	Q3U825	8	38	14.1	554	61085	5.2	Putative uncharacterized protein
2062	Q3U5W2	8	38	14.1	554	61066	5.2	Putative uncharacterized protein

Table S1. Proteins identified in the OVCAR-3 tumor supernatant by two or more unique peptides.

Protein or Protein Group	Accession Number	Unique Peptide Count	Spectrum Count	Sequence Coverage (%)	Length	MolWt	pI	Descriptive Name
2062	Q3TXP9	8	38	14.1	554	61067	5.2	Putative uncharacterized protein
2062	Q3TXJ0	8	38	14.1	553	60923	5.2	Putative uncharacterized protein
2062	Q3TWL8	8	38	14.1	554	61009	5.2	Putative uncharacterized protein
2062	Q3TWE9	8	38	14.1	554	61035	5.3	Putative uncharacterized protein
2062	Q3TKK3	8	38	14.1	554	61053	5.3	Putative uncharacterized protein
2062	Q3TKB2	8	38	14.1	554	61065	5.3	Putative uncharacterized protein
2062	Q3TIT5	8	38	14.1	554	61024	5.2	Putative uncharacterized protein
2062	B2RUD7	8	38	14	556	61294	5.2	Psap protein
2062	Q61207	8	38	14	557	61422	5.2	Sulfated glycoprotein 1
2063	E9Q661	8	10	12	1067	119339	7.3	Uncharacterized protein
2063	E9PVR1	8	10	10	1285	142337	6	Uncharacterized protein
2063	Q80X82	8	10	10	1284	142283	6.1	Symplekin
2063	Q3U2V0	8	10	10	1282	142258	6.2	Putative uncharacterized protein
2063	Q3U2B5	8	10	10	1282	142057	6.1	Putative uncharacterized protein
2063	E9QMP4	8	10	9.9	1288	142620	6.1	Uncharacterized protein
2064	Q9ERV6	8	19	16	643	71477	7	Epidermal growth factor receptor isoform 2
2064	Q9WVF5	8	19	15.7	655	72907	7	Epidermal growth factor receptor
2064	Q01279	8	19	8.5	1210	134853	6.9	Epidermal growth factor receptor
2064	Q9EP98	8	19	8.5	1210	134841	6.9	Epidermal growth factor receptor
2065	E9PYJ6	8	9	5.6	1712	189556	6.9	Uncharacterized protein
2065	A2AKG8	8	9	5.3	1798	198948	6.7	Uncharacterized protein KIAA1797
2066	P14174	8	408	93.9	115	12476	7.9	Macrophage migration inhibitory factor
2067	P05387	8	33	85.2	115	11665	4.5	60S acidic ribosomal protein P2
2068	P09382	8	231	77.8	135	14716	5.5	Galectin-1
2069	Q15370	8	25	76.3	118	13133	4.9	Transcription elongation factor B polypeptide 2
2070	Q9BRA2	8	97	75.6	123	13941	5.5	Thioredoxin domain-containing protein 17
2071	P58546	8	45	72.9	118	12895	5.5	Myotrophin
2072	UPI0001FD28E7	8	27	73.8	225	24513	5.6	D-ribulose-5-phosphate-3-epimerase
2072	Q96AT9	8	27	72.8	228	24928	5.6	Ribulose-phosphate 3-epimerase
2073	Q9NWV4	8	27	71.2	160	18048	5	UPF0587 protein C1orf123
2074	P07741	8	41	70	180	19608	6	Adenine phosphoribosyltransferase
2075	O00762	8	17	69.3	179	19652	7.4	Ubiquitin-conjugating enzyme E2 C
2076	Q8NBT2	8	16	69	197	22443	4.7	Kinetochore protein Spc24
2077	Q9NVP2	8	20	68.8	202	22434	4.6	Histone chaperone ASF1B
2078	Q9H773	8	14	68.2	170	18681	5	dCTP pyrophosphatase 1
2079	Q9Y281	8	41	66.9	166	18737	7.9	Cofilin-2
2080	Q8TCA0	8	12	66.3	184	20509	6.5	Leucine-rich repeat-containing protein 20
2081	P02511	8	93	64.6	175	20159	7.3	Alpha-crystallin B chain
2082	Q9NRV9	8	19	64	189	21097	5.8	Heme-binding protein 1
2083	P43487	8	31	63.2	201	23310	5.3	Ran-specific GTPase-activating protein
2084	P24534	8	76	63.1	225	24764	4.7	Elongation factor 1-beta
2085	O60232	8	24	62.8	199	21474	5.2	Sjoegren syndrome/scleroderma autoantigen 1
2086	Q9H0A8	8	14	62.3	199	21764	7.3	COMM domain-containing protein 4
2087	P41567	8	36	61.9	113	12732	7.5	Eukaryotic translation initiation factor 1
2088	O75340	8	19	61.3	191	21868	5.4	Programmed cell death protein 6
2089	Q13404	8	188	60.5	147	16495	8	Ubiquitin-conjugating enzyme E2 variant 1
2089	Q13404-7	8	188	52.4	170	19307	8.5	Isoform 5 of Ubiquitin-conjugating enzyme E2 variant 1
2089	Q13404-2	8	188	52.4	170	19228	8.2	Isoform 2 of Ubiquitin-conjugating enzyme E2 variant 1
2089	Q13404-1	8	188	40.3	221	25797	8.3	Isoform 1 of Ubiquitin-conjugating enzyme E2 variant 1
2089	UPI0000367052	8	188	24.1	370	42209	6.7	Ubiquitin-conjugating enzyme E2 variant 1 (UEV-1) (CROC-1) (Ubiquitin- conjugating enzyme variant Kua) (TRAF6-regulated IKK activator 1 beta Uev1A).
2089	UPI0000233611	8	188	24.1	370	42210	6.6	TMEM189-UBE2V1 fusion protein
2090	Q9BSD7	8	12	60	190	20713	9.5	Cancer-related nucleoside-triphosphatase
2091	Q9GZQ3	8	21	59.8	224	24670	7	COMM domain-containing protein 5
2092	P40616	8	22	59.7	181	20418	5.7	ADP-ribosylation factor-like protein 1
2093	Q7Z4H3	8	25	59.3	204	23390	5.5	HD domain-containing protein 2
2094	O95881	8	23	58.7	172	19206	5.4	Thioredoxin domain-containing protein 12
2095	B5BUI8	8	32	57.8	185	20577	8.2	Dual specificity phosphatase 3 (Fragment)
2095	P51452	8	32	57.8	185	20478	7.8	Dual specificity protein phosphatase 3
2096	O15511	8	42	57.6	151	16320	5.7	Actin-related protein 2/3 complex subunit 5
2097	Q9Y6G5	8	12	56.9	202	22966	6.5	COMM domain-containing protein 10
2098	UPI00015DFEA6	8	21	56.2	203	22593	9.5	Protein wibg log (Partner of Y14 and mago log).
2098	Q9BRP8-2	8	21	56.2	203	22705	9.4	Isoform 2 of Partner of Y14 and mago
2098	Q9BRP8	8	21	55.9	204	22656	9.5	Partner of Y14 and mago
2099	Q8IZQ5	8	33	55.7	122	13303	9.7	Selenoprotein H
2100	Q07812-8	8	18	58.7	179	19718	5.4	Isoform Sigma of Apoptosis regulator BAX
2100	Q07812	8	18	54.7	192	21184	5.2	Apoptosis regulator BAX
2100	Q07812-2	8	18	48.2	218	24220	7.9	Isoform Beta of Apoptosis regulator BAX
2101	Q96AZ6	8	10	54.7	181	20363	8.9	Interferon-stimulated gene 20 kDa protein
2102	Q9H444	8	11	54.5	224	24950	4.8	Charged multivesicular body protein 4b
2103	P11441	8	18	52.9	157	17776	8.7	Ubiquitin-like protein 4A
2104	Q99828	8	9	52.9	191	21703	4.8	Calcium and integrin-binding protein 1
2105	B2REB8	8	94	51.7	265	30993	4.2	SET nuclear oncogene
2106	B2R4B9	8	24	50.9	175	20078	5.6	Ferritin
2106	Q8WU07	8	24	50.9	175	20062	5.8	Ferritin
2106	Q6IBT7	8	24	50.9	175	19992	5.8	Ferritin
2106	P02792	8	24	50.9	175	20020	5.8	Ferritin light chain
2107	Q86SZ2	8	15	50.6	158	17983	8.7	Trafficking protein particle complex subunit 6B
2108	Q9NRF9	8	23	50.3	147	16860	4.7	DNA polymerase epsilon subunit 3
2109	O75828	8	11	50.2	277	30850	6.2	Carbonyl reductase [NADPH] 3
2110	Q9NP77	8	23	50	194	22574	5.3	RNA polymerase II subunit A C-terminal domain phosphatase SSU72
2111	Q9NTM9	8	39	49.8	273	29341	8.2	Copper homeostasis protein cutC log
2112	P16949	8	28	49.7	149	17302	6	Stathmin
2113	O75832	8	32	49.1	226	24428	6.1	26S proteasome non-ATPase regulatory subunit 10
2114	P28070	8	56	48.5	264	29204	6	Proteasome subunit beta type-4
2115	Q6P587	8	23	48.2	224	24843	7.4	Fumarylacetoacetate hydrolase domain-containing protein 1
2116	Q9UBI1	8	12	48.2	195	22151	6	COMM domain-containing protein 3
2117	Q9NQ88	8	12	47.8	270	30063	7.7	Probable fructose-2,6-bisphosphatase TIGAR
2118	Q9Y5Z4	8	18	46.8	205	22875	4.6	Heme-binding protein 2
2119	Q15126	8	19	46.4	192	21995	5.7	Phosphomevalonate kinase
2120	Q9H4A6	8	29	46.3	298	33811	6.4	Golgi phosphoprotein 3
2121	B3KP20	8	19	45.9	270	29221	6.4	cDNA FLJ30970 fis, clone HEART2000444, highly similar to sapiens phospholysine phosphohistidine inorganic pyrophosphate phosphatase (LHPP), mRNA
2121	Q9H008	8	19	45.9	270	29165	6.2	Phospholysine phosphohistidine inorganic pyrophosphate phosphatase
2122	P35080-2	8	155	61.4	140	15088	6.1	Isoform IIb of Profilin-2
2122	B4DNH1	8	155	45.7	188	20840	7.8	Profilin
2122	UPI0000EE20CE	8	155	45.7	188	20787	7.5	UPI0000EE20CE UniRef100 entry
2122	D3DNI2	8	155	25.7	334	34944	10.3	Profilin (Fragment)
2123	Q9BV57	8	37	45.3	179	21498	5.7	1,2-dihydroxy-3-keto-5-methylthiopentene dioxygenase
2124	P61006	8	52	44.9	207	23668	9.1	Ras-related protein Rab-8A

Table S1. Proteins identified in the OVCAR-3 tumor supernatant by two or more unique peptides.

Protein or Protein Group	Accession Number	Unique Peptide Count	Spectrum Count	Sequence Coverage (%)	Length	MolWt	pI	Descriptive Name
2125	Q9Y3B8-2	8	17	53.3	199	23208	6.1	Isoform 2 of Oligoribonuclease, mitochondrial
2125	Q9Y3B8	8	17	44.7	237	26833	6.9	Oligoribonuclease, mitochondrial
2126	Q9H081	8	8	44.4	205	24140	5.7	Protein MIS12 log
2127	Q96B26	8	11	44.2	276	30040	5.3	Exosome complex exonuclease RRP43
2128	Q15056	8	81	44	248	27385	7.2	Eukaryotic translation initiation factor 4H
2129	P06748-2	8	344	48.7	265	29465	4.6	Isoform 2 of Nucleophosmin
2129	P06748	8	344	43.9	294	32575	4.8	Nucleophosmin
2130	B3KUR3	8	17	43.4	242	27992	5.8	cDNA FLJ40459 fis, clone TESTI2041800, highly similar to BISPHOSPHOGLYCERATE MUTASE (EC 5.4.2.4)
2130	P07738	8	17	40.5	259	30005	6.5	Bisphosphoglycerate mutase
2131	P46778	8	42	43.1	160	18565	10.5	60S ribosomal protein L21
2132	B3KXN9	8	18	42.6	270	29854	6	cDNA FLJ45793 fis, clone NT2RI2009233
2132	Q96SZ5	8	18	42.6	270	29751	6	2-aminoethanethiol dioxygenase
2133	Q07020	8	28	42.6	188	21634	11.7	60S ribosomal protein L18
2134	O14972	8	19	42.4	297	33010	7.7	Down syndrome critical region protein 3
2135	O95292	8	40	42.4	243	27228	7.3	Vesicle-associated membrane protein-associated protein B/C
2136	P35219	8	12	42.1	290	32973	4.9	Carbonic anhydrase-related protein
2137	Q32Q75	8	21	45.2	217	25067	6.1	Eukaryotic translation initiation factor 4E
2137	P06730	8	21	45.2	217	25097	6.1	Eukaryotic translation initiation factor 4E
2137	B7Z6V1	8	21	41.4	237	27260	6.8	cDNA FLJ55157, highly similar to Eukaryotic translation initiation factor 4E
2137	D6RBW1	8	21	40	245	28512	8.1	Uncharacterized protein
2137	D6RCQ6	8	21	39.5	248	28778	6.4	Uncharacterized protein
2138	P13984	8	20	41	249	28380	9.2	General transcription factor IIF subunit 2
2139	P49366	8	12	39.8	369	40971	5.4	Deoxyhypusine synthase
2140	Q9H8W4	8	11	39.4	249	27798	8.2	Pleckstrin logy domain-containing family F member 2
2141	Q02543	8	24	39.2	176	20762	10.7	60S ribosomal protein L18a
2142	P48507	8	14	39.1	274	30727	6	Glutamate--cysteine ligase regulatory subunit
2143	O00625	8	14	39	290	32113	6.9	Pirin
2144	B2RDZ9	8	16	38.1	291	31874	5.3	cDNA, FLJ96850
2144	Q15024	8	16	38.1	291	31821	5.2	Exosome complex exonuclease RRP42
2145	Q13151	8	34	38	305	30841	9.3	Heterogeneous nuclear ribonucleoprotein A0
2146	Q9NWV8	8	10	37.4	329	36560	4.6	BRISC and BRCA1-A complex member 1
2147	P49770	8	11	37.3	351	38990	6.2	Translation initiation factor eIF-2B subunit beta
2148	Q96BJ3	8	17	37.3	306	35023	6.6	Axin interactor, dorsalization-associated protein
2148	UPI000013EF67	8	17	37.3	306	34925	6.7	PREDICTED: axin interactor, dorsalization-associated protein-like
2149	P30084	8	15	37.2	290	31387	8.1	Enoyl-CoA hydratase, mitochondrial
2150	A8K9V9	8	23	36.1	310	34532	5.4	cDNA FLJ76064
2150	Q96KN1	8	23	36.1	310	34474	5.5	Protein FAM84B
2151	P80217	8	20	36	286	31546	6.1	Interferon-induced 35 kDa protein
2151	P80217-2	8	20	35.8	288	31777	6.1	Isoform 2 of Interferon-induced 35 kDa protein
2152	Q13242	8	22	34.4	221	25542	8.6	Serine/arginine-rich splicing factor 9
2153	O95749	8	10	34	300	34871	6.1	Geranylgeranyl pyrophosphate synthase
2154	E7EQV7	8	20	33.6	542	58329	8	Uncharacterized protein
2154	E7EUE8	8	20	33.6	542	58349	8	Uncharacterized protein
2155	Q05BK6	8	85	33.3	366	40691	6.4	TFG protein
2155	Q7Z426	8	85	30.8	396	43006	5.2	Putative MAPK activating protein
2155	Q92734	8	85	30.5	400	43448	5.1	Protein TFG
2155	Q8TDJ5	8	85	15.2	803	88671	6.3	Tyrosine-protein kinase receptor
2156	Q96S99	8	18	43.4	279	31195	8.2	Pleckstrin logy domain-containing family F member 1
2156	B4DWN9	8	18	33.2	364	40679	8.6	cDNA FLJ55258, highly similar to sapiens pleckstrin logy domain containing, family F member 1, mRNA
2157	P32456	8	12	33.2	591	67209	5.7	Interferon-induced guanylate-binding protein 2
2158	Q9UMX0	8	29	33.1	589	62519	5.1	Ubiquilin-1
2159	O43681	8	16	33	348	38793	4.9	ATPase ASNA1
2160	C7DUW4	8	17	32.9	346	39219	7.4	Mitogen activated protein kinase kinase 3
2160	P46734	8	17	32.9	347	39318	7.4	Dual specificity mitogen-activated protein kinase kinase 3
2160	C9JI18	8	17	32.5	351	39853	7.5	Uncharacterized protein
2160	P46734-3	8	17	32.4	352	39940	7.5	Isoform 2 of Dual specificity mitogen-activated protein kinase kinase 3
2161	P08754	8	12	32.8	354	40532	5.7	Guanine nucleotide-binding protein G(k) subunit alpha
2162	B5BU16	8	11	32.6	334	37464	7.1	Mitogen-activated protein kinase kinase 6
2162	UPI0001AE66AC	8	11	32.6	334	37532	7.1	UPI0001AE66AC UniRef100 entry
2162	P52564	8	11	32.6	334	37492	7.4	Dual specificity mitogen-activated protein kinase kinase 6
2163	Q969U7	8	22	31.8	264	29396	7	Proteasome assembly chaperone 2
2164	Q9BYG5	8	18	31.5	372	41182	5.6	Partitioning defective 6 log beta
2165	Q9Y2Z0-2	8	16	33.9	333	37805	5.2	Isoform 2 of Suppressor of G2 allele of SKP1 log
2165	A8K5T7	8	16	31	365	41052	5.2	cDNA FLJ75365, highly similar to sapiens SUGT1B (SUGT1) mRNA
2165	Q9Y2Z0	8	16	31	365	41024	5.2	Suppressor of G2 allele of SKP1 log
2166	B2RAR3	8	18	30.8	403	43990	7.4	Queuine tRNA-ribosyltransferase
2166	Q9BXR0	8	18	30.8	403	44048	7.2	Queuine tRNA-ribosyltransferase
2167	O95456-2	8	25	33	267	30288	7.8	Isoform 2 of Proteasome assembly chaperone 1
2167	O95456	8	25	30.6	288	32854	7.2	Proteasome assembly chaperone 1
2168	P49247	8	14	29.9	311	33269	8.5	Ribose-5-phosphate isomerase
2169	B4DWN1	8	12	37.2	285	32578	6.5	cDNA FLJ52285, highly similar to Vesicular integral-membrane protein VIP36
2169	D6RBV2	8	12	32.6	325	36543	6.7	Uncharacterized protein
2169	A8K7T4	8	12	29.8	356	40243	7	cDNA FLJ75774, highly similar to sapiens lectin, mannose-binding 2 (LMAN2), mRNA
2169	Q12907	8	12	29.8	356	40229	7	Vesicular integral-membrane protein VIP36
2170	Q9H8Y8	8	31	30.5	452	47145	4.8	Golgi reassembly-stacking protein 2
2170	B4DKT0	8	31	29.7	464	48674	4.8	cDNA FLJ59712, highly similar to Golgi reassembly-stacking protein 2
2171	Q9GZL7	8	12	29.6	423	47708	5.9	Ribosome biogenesis protein WDR12
2172	Q8IUI8	8	13	28.3	442	49766	5.1	Cytokine receptor-like factor 3
2173	B4DHT4	8	16	27.9	462	53510	8.6	cDNA FLJ59519, highly similar to U4/U6.U5 tri-snRNP-associated protein 2
2173	Q53GS9	8	16	22.8	565	65381	8.9	U4/U6.U5 tri-snRNP-associated protein 2
2174	A8K4G7	8	15	26.6	444	49273	6.8	cDNA FLJ78528, highly similar to sapiens vacuolar protein sorting 4B (yeast) (VPS4B), mRNA
2174	O75351	8	15	26.6	444	49302	7.2	Vacuolar protein sorting-associated protein 4B
2174	A8K5D8	8	15	26.6	444	49231	7	cDNA FLJ75934, highly similar to sapiens vacuolar protein sorting 4B (yeast) (VPS4B), mRNA
2175	P06746	8	11	26.6	335	38178	8.9	DNA polymerase beta
2175	Q53EV2	8	11	26.6	335	38162	8.9	Polymerase (DNA directed), beta variant (Fragment)
2176	Q9NR50-2	8	9	28.4	412	46145	7.1	Isoform 2 of Translation initiation factor eIF-2B subunit gamma
2176	Q9NR50	8	9	25.9	452	50240	6.5	Translation initiation factor eIF-2B subunit gamma
2177	P50552	8	21	25.5	380	39830	8.9	Vasodilator-stimulated phosphoprotein
2178	B0VJZ1	8	13	25.3	478	50767	7.5	Leukocyte receptor cluster (LRC) member 9
2179	Q5W0U4	8	11	24.4	402	44381	6.4	B box and SPRY domain-containing protein
2180	Q96Q11-2	8	13	25.4	414	47829	8.5	Isoform 2 of CCA tRNA nucleotidyltransferase 1, mitochondrial

Table S1. Proteins identified in the OVCAR-3 tumor supernatant by two or more unique peptides.

Protein or Protein Group	Accession Number	Unique Peptide Count	Spectrum Count	Sequence Coverage (%)	Length	MolWt	pI	Descriptive Name
2180	Q96Q11	8	13	24.2	434	50128	8.1	CCA tRNA nucleotidyltransferase 1, mitochondrial
2181	Q92879-2	8	17	24.3	482	51546	8.5	Isoform 2 of CUGBP Elav-like family member 1
2181	Q92879-3	8	17	24.2	483	51617	8.5	Isoform 3 of CUGBP Elav-like family member 1
2181	Q92879	8	17	24.1	486	52063	8.5	CUGBP Elav-like family member 1
2181	UPI0000EE39EF	8	17	24	487	52134	8.5	UPI0000EE39EF UniRef100 entry
2181	Q92879-4	8	17	22.9	512	55001	8.4	Isoform 4 of CUGBP Elav-like family member 1
2182	O95396	8	11	23.7	460	49669	6.2	Adenylyltransferase and sulfurtransferase MOCS3
2183	A8K8G3	8	35	23.6	424	46825	4.8	cDNA FLJ77237, highly similar to sapiens sparc/osteonectin, cwcv and kazal-like domains proteoglycan (testican) 2 (SPOCK2), mRNA
2183	Q92563	8	35	23.6	424	46779	4.8	Testican-2
2184	Q9UJA5	8	9	23.5	497	55799	7.5	tRNA (adenine-N(1)-)-methyltransferase non-catalytic subunit TRM6
2185	B4DDV1	8	11	26	434	50520	7.8	cDNA FLJ53857, highly similar to Interferon-induced protein with tetratricopeptide repeats 5
2185	B2R5X9	8	11	23.4	482	55946	7.6	cDNA, FLJ92678, highly similar to sapiens interferon-induced protein with tetratricopeptide repeats 5 (IFIT5), mRNA
2185	Q13325	8	11	23.4	482	55847	7.4	Interferon-induced protein with tetratricopeptide repeats 5
2186	Q8N6H7	8	15	22.3	521	56720	8	ADP-ribosylation factor GTPase-activating protein 2
2187	A8K9B9	8	8	21.8	541	61156	6.7	cDNA FLJ77391, highly similar to sapiens EH-domain containing 4 (EHD4), mRNA
2187	Q9H223	8	8	21.8	541	61175	6.8	EH domain-containing protein 4
2188	O43148	8	14	22.3	476	54844	6.6	mRNA cap guanine-N7 methyltransferase
2188	A8K946	8	14	21	504	57751	7.7	mRNA cap guanine-N7 methyltransferase
2188	O43148-2	8	14	21	504	57725	7.7	Isoform 2 of mRNA cap guanine-N7 methyltransferase
2189	Q9BVJ8	8	22	27.6	409	47094	5	HEXA protein (Fragment)
2189	B4DVA7	8	22	20.9	540	62013	5.3	cDNA FLJ53927, highly similar to Beta-hexosaminidase alpha chain (EC 3.2.1.52)
2190	Q53HT6	8	13	26.4	246	27962	8.4	B-cell receptor-associated protein 31 variant (Fragment)
2190	Q53G72	8	13	26.4	246	27931	8.4	B-cell receptor-associated protein 31 variant (Fragment)
2190	P51572	8	13	26.4	246	27992	8.4	B-cell receptor-associated protein 31
2190	B3KQ79	8	13	20.8	313	34752	7.8	B-cell receptor-associated protein 31, isoform CRA_b
2191	Q3ZCQ8	8	42	26.3	353	39646	8.4	Mitochondrial import inner membrane translocase subunit TIM50
2191	Q0VAB1	8	42	20.4	456	50479	9.4	Translocase of inner mitochondrial membrane 50 log (S. cerevisiae)
2191	Q3ZCQ8-2	8	42	20.4	456	50465	9.4	Isoform 2 of Mitochondrial import inner membrane translocase subunit TIM50
2192	B4DXZ1	8	12	20.2	540	62154	7.1	cDNA FLJ51768, highly similar to Asparagine synthetase (glutamine-hydrolyzing) (EC 6.3.5.4)
2192	P08243	8	12	19.4	561	64370	6.9	Asparagine synthetase [glutamine-hydrolyzing]
2193	Q8NA80	8	41	21.3	534	58309	9.2	cDNA FLJ35762 fis, clone TESTI2004793, moderately similar to sapiens NY-REN-2 antigen mRNA
2193	Q7Z739	8	41	19.5	585	63861	9	YTH domain family protein 3
2193	B4DPX9	8	41	19.4	588	64522	8.2	cDNA FLJ56221, highly similar to YTH domain protein 3
2194	Q92804	8	104	18.8	592	61830	8	TATA-binding protein-associated factor 2N
2194	Q92804-2	8	104	18.8	589	61558	8	Isoform Short of TATA-binding protein-associated factor 2N
2195	O95352	8	12	18.1	703	77960	6.2	Ubiquitin-like modifier-activating enzyme ATG7
2196	P43405-2	8	9	17.8	612	69510	7.6	Isoform Short of Tyrosine-protein kinase SYK
2197	B4DQX8	8	13	17.4	633	71339	5.8	cDNA FLJ51723, highly similar to DCC-interacting protein 13 alpha (Fragment)
2197	Q9UKG1	8	13	15.5	709	79664	5.4	DCC-interacting protein 13-alpha
2198	E9PDE8	8	20	18.3	813	91954	6.1	Uncharacterized protein
2198	Q53ZP9	8	20	17.8	839	94505	5.9	Heat shock protein apg-1
2198	O95757	8	20	17.8	839	94512	5.9	Heat shock 70 kDa protein 4L
2198	B4DZR0	8	20	17.2	868	97562	5.8	cDNA FLJ55529, highly similar to Heat shock 70 kDa protein 4L
2199	B4DUD5	8	28	18.3	553	58618	6.8	cDNA FLJ58787, highly similar to Cleavage stimulation factor 64 kDa subunit
2199	P33240-2	8	28	18	560	59251	6.8	Isoform 2 of Cleavage stimulation factor subunit 2
2199	P33240	8	28	17.5	577	60959	6.8	Cleavage stimulation factor subunit 2
2199	B3V096	8	28	16.9	597	62929	6.9	BetaCstF-64 variant 2
2199	E7EWR4	8	28	16.9	597	62943	6.9	Uncharacterized protein
2200	Q8WUI4-4	8	9	17.2	922	99755	7.5	Isoform 4 of Histone deacetylase 7
2200	Q8WUI4	8	9	16.7	952	102927	7.6	Histone deacetylase 7
2200	Q8WUI4-7	8	9	16.7	954	102906	7.3	Isoform 7 of Histone deacetylase 7
2200	Q8WUI4-5	8	9	16	991	106740	7.5	Isoform 5 of Histone deacetylase 7
2201	Q15582	8	15	16.1	683	74681	7.7	Transforming growth factor-beta-induced protein ig-h3
2201	Q53GU8	8	15	16.1	683	74651	7.7	Transforming growth factor, beta-induced, 68kDa variant (Fragment)
2202	E1B6W5	8	17	57.1	140	15079	8.9	Uncharacterized protein
2202	P22307-2	8	17	55.9	143	15401	8.9	Isoform SCP2 of Non-specific lipid-transfer protein
2202	B4DHP6	8	17	17.2	466	50342	7	cDNA FLJ53991, highly similar to Nonspecific lipid-transfer protein (EC 2.3.1.176)
2202	A6NM69	8	17	15.9	503	54415	6.7	Uncharacterized protein
2202	C9JC79	8	17	15.3	523	56497	6.9	Uncharacterized protein
2202	B4DGJ9	8	17	15.3	523	56497	6.7	cDNA FLJ53982, highly similar to Nonspecific lipid-transfer protein (EC 2.3.1.176)
2202	P22307	8	17	14.6	547	58994	6.9	Non-specific lipid-transfer protein
2202	B2R761	8	17	14.6	547	59022	7	cDNA, FLJ93299, highly similar to sapiens sterol carrier protein 2 (SCP2), mRNA
2203	B4E2E1	8	30	15.2	867	96176	7.1	cDNA FLJ54322, highly similar to Protein transport protein Sec24B
2203	O95487-2	8	30	10.7	1233	133629	7.4	Isoform 2 of Protein transport protein Sec24B
2203	O95487	8	30	10.4	1268	137417	6.7	Protein transport protein Sec24B
2203	B7ZKM8	8	30	10.2	1298	140421	6.6	SEC24B protein
2204	Q14694	8	14	15	798	87134	5.3	Ubiquitin carboxyl-terminal hydrolase 10
2204	UPI0001AE68C9	8	14	15	798	87228	5.3	Ubiquitin carboxyl-terminal hydrolase 10 (EC 3.1.2.15) (Ubiquitin thioesterase 10) (Ubiquitin-specific-processing protease 10) (Deubiquitinating enzyme 10).
2204	Q14694-3	8	14	15	802	87533	5.3	Isoform 3 of Ubiquitin carboxyl-terminal hydrolase 10
2204	Q14694-2	8	14	14.2	846	92597	5.7	Isoform 2 of Ubiquitin carboxyl-terminal hydrolase 10
2205	Q9BXB4	8	20	14.2	747	83643	7.1	Oxysterol-binding protein-related protein 11
2206	Q9H1B7	8	43	14.2	796	82659	8.2	Interferon regulatory factor 2-binding protein-like
2207	B2RCG8	8	10	14.1	686	76933	6.9	cDNA, FLJ96063, highly similar to sapiens rhophilin, Rho GTPase binding protein 2 (RHPN2), mRNA
2207	Q8IUC4	8	10	14.1	686	76993	6.8	Rhophilin-2
2207	B3KUY8	8	10	14.1	686	77025	6.8	cDNA FLJ40927 fis, clone UTERU2006593, highly similar to Rhophilin-2
2208	P20936-2	8	11	13.9	870	100398	7.6	Isoform 2 of Ras GTPase-activating protein 1
2208	B4DTL2	8	11	13.8	880	101546	7.8	cDNA FLJ53191, highly similar to Ras GTPase-activating protein 1
2208	E9PGC0	8	11	13.7	881	101790	7.8	Uncharacterized protein
2208	B4DTX4	8	11	13.7	881	101756	7.8	cDNA FLJ59756, highly similar to Ras GTPase-activating protein 1

Table S1. Proteins identified in the OVCAR-3 tumor supernatant by two or more unique peptides.

Protein or Protein Group	Accession Number	Unique Peptide Count	Spectrum Count	Sequence Coverage (%)	Length	MolWt	pI	Descriptive Name
2208	P20936	8	11	11.6	1047	116403	6.5	Ras GTPase-activating protein 1
2209	B0V0F8	8	11	13.6	669	76933	6.1	DEAH (Asp-Glu-Ala-His) box polypeptide 16 (Fragment)
2209	O60231	8	11	8.7	1041	119263	6.8	Putative pre-mRNA-splicing factor ATP-dependent RNA helicase DHX16
2210	Q2NL82	8	15	12.1	804	91810	7.4	Pre-rRNA-processing protein TSR1 log
2211	B7Z3E5	8	13	11	1039	119056	9.4	cDNA FLJ53573, highly similar to Myosin Ic
2211	O00159-3	8	13	10.9	1044	119628	9.5	Isoform 3 of Myosin-Ic
2211	O00159	8	13	10.7	1063	121682	9.4	Myosin-Ic
2212	Q5W7F7	8	9	11.3	852	97958	5.3	Ubiquitin ligase E3A isoform 1
2212	Q05086	8	9	11	875	100688	5.2	Ubiquitin-protein ligase E3A
2212	Q9BUI6	8	9	11	872	100044	5.2	Ubiquitin protein ligase E3A
2212	Q05086-3	8	9	11	872	100102	5.2	Isoform III of Ubiquitin-protein ligase E3A
2213	A2A2F0	8	10	11.3	1323	147389	6.8	OTTHUMP00000178925 (Fragment)
2213	A2A2E9	8	10	10.1	1491	166527	6.7	OTTHUMP00000030968
2213	Q86X10-3	8	10	10.1	1490	166398	6.8	Isoform 3 of Ral GTPase-activating protein subunit beta
2213	Q86X10	8	10	10	1494	166798	6.8	Ral GTPase-activating protein subunit beta
2214	Q5T1M5	8	33	10.1	1219	133630	5.2	FK506-binding protein 15
2215	Q7Z2Z2-2	8	12	9.8	1069	119887	5.8	Isoform 2 of Elongation factor Tu GTP-binding domain-containing protein 1
2215	Q7Z2Z2	8	12	9.4	1120	125430	5.9	Elongation factor Tu GTP-binding domain-containing protein 1
2216	B3KY56	8	37	9.2	1058	117393	5.9	cDNA FLJ46898 fis, clone UTERU3022168, highly similar to Protein FAM62A
2216	B3KMV5	8	37	8.8	1104	122905	5.8	cDNA FLJ12728 fis, clone NT2RP2000040, highly similar to Protein FAM62A
2216	Q9BSJ8	8	37	8.8	1104	122856	5.8	Extended synaptotagmin-1
2216	Q9BSJ8-2	8	37	8.7	1114	124003	5.8	Isoform 2 of Extended synaptotagmin-1
2217	UPI000022D624	8	23	15.2	604	67223	6.6	epithelial splicing regulatory protein 1 isoform 5
2217	Q6NXG1-2	8	23	15.1	608	67665	6.8	Isoform 2 of Epithelial splicing regulatory protein 1
2217	E9PB47	8	23	14	659	73133	6.8	Uncharacterized protein
2217	Q6NXG1-4	8	23	13.9	663	73574	6.9	Isoform 4 of Epithelial splicing regulatory protein 1
2217	Q6NXG1-3	8	23	13.6	677	75144	6.6	Isoform 3 of Epithelial splicing regulatory protein 1
2217	Q6NXG1	8	23	13.5	681	75585	6.7	Epithelial splicing regulatory protein 1
2217	D7PBN3	8	23	8.7	1061	118616	8.2	ESRP1/RAF1 fusion protein
2218	E5KLM2	8	11	8.6	960	111657	7.9	Mitochondrial dynamin-like 120 kDa protein
2218	E5KLM1	8	11	8.3	997	115911	7.9	Mitochondrial dynamin-like 120 kDa protein
2219	B7ZM80	8	8	8.1	1051	116646	6.8	Integrin, alpha 3 (Antigen CD49C, alpha 3 subunit of VLA-3 receptor)
2219	P26006-2	8	8	8.1	1051	116612	6.8	Isoform Alpha-3A of Integrin alpha-3
2219	P26006	8	8	8	1066	118756	7	Integrin alpha-3
2220	O14981	8	11	8	1849	206886	6.5	TATA-binding protein-associated factor 172
2221	B7ZLD0	8	8	7.3	1568	175449	5.4	LRP6 protein
2221	O75581	8	8	7.1	1613	180428	5.3	Low-density lipoprotein receptor-related protein 6
2222	Q9H1A4	8	10	6.7	1944	216498	6.3	Anaphase-promoting complex subunit 1
2223	E0AD28	8	11	5.4	1829	208447	5.7	T-Dicer
2223	Q9UPY3	8	11	5.1	1922	218680	5.7	Endoribonuclease Dicer
2224	E7EVK3	8	8	5.3	1917	216029	6.2	Uncharacterized protein
2224	Q6ZT12-4	8	8	5.3	1917	215969	6.2	Isoform 4 of E3 ubiquitin-protein ligase UBR3
2224	Q6ZT12	8	8	5.3	1888	212432	6.1	E3 ubiquitin-protein ligase UBR3
2225	Q02388	8	8	3.7	2944	295216	6.3	Collagen alpha-1(VII) chain
2225	Q02388-2	8	8	3.7	2912	292264	6.2	Isoform 2 of Collagen alpha-1(VII) chain
2226	E7ERU0	8	12	3.3	5375	615661	5.7	Uncharacterized protein
2226	E9PBJ8	8	12	3.3	5497	629794	5.7	Uncharacterized protein
2226	E9PEB9	8	12	3.2	5675	649550	6	Uncharacterized protein
2226	Q03001	8	12	2.4	7570	860679	5.2	Dystonin
2226	E9PBT7	8	12	2.4	7461	847987	5.2	Uncharacterized protein
2226	Q03001-11	8	12	2.3	7748	880435	5.4	Isoform 6 of Dystonin
2227	P62306	7	100	88.4	86	9725	4.7	Small nuclear ribonucleoprotein F Small nuclear ribonucleoprotein F
2228	B2R4N3	7	21	79.5	73	8548	7.3	cDNA, FLJ92155, highly similar to sapiens ubiquitin-like 5 (UBL5), mRNA
2228	Q9BZL1	7	21	79.5	73	8547	8.4	Ubiquitin-like protein 5 Ubiquitin-like protein 5
2229	C1J0L1	7	131	67.2	131	13977	10.4	Ribosomal protein L23 (Fragment)
2229	P62829	7	131	62.9	140	14865	10.5	60S ribosomal protein L23 60S ribosomal protein L23
2230	P62736	7	274	66.3	377	42009	5.4	Actin, aortic smooth muscle Actin, aortic smooth muscle
2230	Q9CXK3	7	274	66.3	377	41947	5.5	Putative uncharacterized protein
2230	P68032	7	274	66.3	377	42019	5.4	Actin, alpha cardiac muscle 1 Actin, alpha cardiac muscle 1
2231	O60493	7	48	63.6	162	18762	8.7	Sorting nexin-3 Sorting nexin-3
2231	O70492	7	48	63.6	162	18757	8.7	Sorting nexin-3
2232	O60739	7	58	61.1	113	12824	7.4	Eukaryotic translation initiation factor 1b Eukaryotic translation initiation factor 1b
2233	P62244	7	222	60	130	14839	10.1	40S ribosomal protein S15a 40S ribosomal protein S15a
2234	Q12974	7	58	56.9	167	19127	8.4	Protein tyrosine phosphatase type IVA 2 Protein tyrosine phosphatase type IVA 2
2235	Q969T4	7	49	56	207	22913	7.2	Ubiquitin-conjugating enzyme E2 E3 Ubiquitin-conjugating enzyme E2 E3
2236	P62316	7	138	55.9	118	13527	9.9	Small nuclear ribonucleoprotein Sm D2 Small nuclear ribonucleoprotein Sm D2
2237	P25398	7	136	54.5	132	14515	7.2	40S ribosomal protein S12 40S ribosomal protein S12
2237	UPI0000023CC7	7	136	54.5	132	14514	7.9	PREDICTED: 40S ribosomal protein S12-like
2238	A8MZB2	7	13	51.8	166	18857	5	N-acetyltransferase 5
2238	P61599	7	13	48.3	178	20368	5	N-alpha-acetyltransferase 20, NatB catalytic subunit N-alpha-acetyltransferase 20, NatB catalytic subunit
2238	Q9DB82	7	13	45.7	188	21470	5.1	N-acetyltransferase 5 (ARD1 log, S. cerevisiae)
2239	P62380	7	20	51.6	186	20887	9.5	TATA box-binding protein-like protein 1 TATA box-binding protein-like protein 1
2240	P61018	7	13	49.8	213	23587	6	Ras-related protein Rab-4B
2240	Q91ZR1	7	13	49.8	213	23629	6	Ras-related protein Rab-4B
2240	P61018-2	7	13	42.7	248	27506	6.4	Isoform 2 of Ras-related protein Rab-4B
2241	C9J7T6	7	104	49.7	163	18592	10	Uncharacterized protein Uncharacterized protein
2242	A0JLV3	7	118	48.8	123	13579	10.4	Histone H2B (Fragment)
2242	Q99880	7	118	47.6	126	13952	10.3	Histone H2B type 1-L
2242	Q99879	7	118	47.6	126	13989	10.3	Histone H2B type 1-M
2242	Q99877	7	118	47.6	126	13922	10.3	Histone H2B type 1-N
2242	Q93079	7	118	47.6	126	13892	10.3	Histone H2B type 1-H
2242	Q8CGP2	7	118	47.6	126	13992	10.3	Histone H2B type 1-P
2242	Q8CGP1	7	118	47.6	126	13920	10.3	Histone H2B type 1-K
2242	Q64478	7	118	47.6	126	13920	10.3	Histone H2B type 1-H
2242	Q64475	7	118	47.6	126	13952	10.3	Histone H2B type 1-B
2242	Q5QNW6	7	118	47.6	126	13920	10.3	Histone H2B type 2-F Histone H2B type 2-F
2242	P62807	7	118	47.6	126	13906	10.3	Histone H2B type 1-C/E/F/G/I Histone H2B type 1-C/E/F/G/I
2242	P58876	7	118	47.6	126	13936	10.3	Histone H2B type 1-D Histone H2B type 1-D
2242	P57053	7	118	47.6	126	13944	10.4	Histone H2B type F-S
2242	P10853	7	118	47.6	126	13936	10.3	Histone H2B type 1-F/J/L

Table S1. Proteins identified in the OVCAR-3 tumor supernatant by two or more unique peptides.

Protein or Protein Group	Accession Number	Unique Peptide Count	Spectrum Count	Sequence Coverage (%)	Length	MolWt	pI	Descriptive Name
2242	O60814	7	118	47.6	126	13890	10.3	Histone H2B type 1-K
2242	A8K9J7	7	118	47.6	126	13996	10.3	Histone H2B
2242	Q8CBB6	7	118	44.8	134	14888	10.4	Histone H2B
2242	Q921L4	7	118	44.4	135	14939	10.1	Histone H2B
2242	B4DR52	7	118	36.1	166	18041	10.3	Histone H2B
2243	P11233	7	23	46.1	206	23567	7.1	Ras-related protein Ral-A
2243	P63321	7	23	46.1	206	23553	7.1	Ras-related protein Ral-A
2244	P53680	7	77	44.4	142	17018	6.2	AP-2 complex subunit sigma AP-2 complex subunit sigma
2245	P61081	7	46	42.1	183	20900	7.7	NEDD8-conjugating enzyme Ubc12 NEDD8-conjugating enzyme Ubc12
2246	Q16629-3	7	56	51.5	132	15257	9.6	Isoform 3 of Serine/arginine-rich splicing factor 7
2246	Q16629-2	7	56	50.4	135	15573	9.8	Isoform 2 of Serine/arginine-rich splicing factor 7
2246	UPI000013D48B	7	56	49.6	137	15763	9.8	UPI000013D48B UniRef100 entry
2246	Q8BL97-3	7	56	43.3	157	17890	9.5	Isoform 3 of Serine/arginine-rich splicing factor 7
2246	A6NNE8	7	56	41.2	165	18841	10.6	Uncharacterized protein
2246	B4DEK2	7	56	41.2	165	18815	10.7	cDNA FLJ59182, highly similar to Splicing factor, arginine/serine-rich 7
2246	UPI0001E24E1A	7	56	30.5	223	25584	11.8	serine/arginine-rich splicing factor 7 isoform 4
2246	UPI0000EE2586	7	56	30.1	226	26012	11.8	serine/arginine-rich splicing factor 7 isoform 2
2246	D3Z964	7	56	30.1	226	26023	11.8	RCG61762, isoform CRA_e
2246	UPI0000506E2B	7	56	30	227	26181	11.8	serine/arginine-rich splicing factor 7 isoform 3
2246	C9JAB2	7	56	28.9	235	26928	11.9	Uncharacterized protein
2246	Q3THA6	7	56	28.6	238	27378	11.8	MCG17902, isoform CRA_a
2246	Q16629	7	56	28.6	238	27367	11.8	Serine/arginine-rich splicing factor 7
2246	E9QK40	7	56	25.6	266	30704	11.9	Uncharacterized protein
2246	Q8BL97	7	56	25.5	267	30818	11.9	Serine/arginine-rich splicing factor 7
2247	Q52KF0	7	11	40.3	149	17020	6.3	Thioredoxin-like 4B
2247	Q9NX01	7	11	40.3	149	17015	6	Thioredoxin-like protein 4B
2247	Q53GP2	7	11	40.3	149	16989	6.3	Thioredoxin-like 4B variant (Fragment)
2248	Q8BPG5	7	56	43.8	192	21424	8.5	Putative uncharacterized protein
2248	P63000	7	56	43.8	192	21450	8.5	Ras-related C3 botulinum toxin substrate 1 Ras-related C3 botulinum toxin substrate 1
2248	A4D2P0	7	56	39.8	211	23467	8.6	Ras-related C3 botulinum toxin substrate 1 (Rho family, small GTP binding protein Rac1)
2248	Q3TLP8	7	56	39.8	211	23432	8.7	RAS-related C3 botulinum substrate 1, isoform CRA_a
2249	P62070	7	15	39.7	204	23400	6	Ras-related protein R-Ras2 Ras-related protein R-Ras2
2249	B7Z5Z2	7	15	38.6	210	24196	6	cDNA FLJ61162, highly similar to Ras-related protein R-Ras2
2250	Q712K3	7	14	37.4	238	27166	4.4	Ubiquitin-conjugating enzyme E2 R2 Ubiquitin-conjugating enzyme E2 R2
2251	P62256	7	36	36.1	183	20655	4.7	Ubiquitin-conjugating enzyme E2 H Ubiquitin-conjugating enzyme E2 H
2252	Q59GE6	7	10	37.5	280	31777	7.6	Chromosome 11 open reading frame 8 variant (Fragment)
2252	Q15777	7	10	35.7	294	33360	6.3	Metallophosphoesterase MPPED2
2252	Q9CZJ0	7	10	35.7	294	33374	6.3	Metallophosphoesterase MPPED2
2253	C9JB30	7	24	31.2	218	24582	9	Uncharacterized protein
2253	Q9UPY8-2	7	24	25.6	266	30380	5.3	Isoform 2 of Microtubule-associated protein RP/EB family member 3
2253	D3Z6G3	7	24	25.6	266	30364	5.3	Uncharacterized protein
2253	Q9UPY8	7	24	24.2	281	31982	5.5	Microtubule-associated protein RP/EB family member 3
2253	Q6PER3	7	24	24.2	281	31966	5.5	Microtubule-associated protein RP/EB family member 3
2254	Q14558	7	9	29.2	356	39394	7.2	Phosphoribosyl pyrophosphate synthase-associated protein 1
2254	Q9D0M1	7	9	29.2	356	39432	7.2	Phosphoribosyl pyrophosphate synthase-associated protein 1
2254	Q14558-2	7	9	27	385	42468	8.5	Isoform 2 of Phosphoribosyl pyrophosphate synthase-associated protein 1
2255	Q3UF75	7	13	28.9	336	38361	5.1	Putative uncharacterized protein
2255	Q9NVD7	7	13	26.1	372	42244	5.9	Alpha-parvin
2255	Q9EPC1	7	13	26.1	372	42330	5.9	Alpha-parvin
2255	Q8C5R4	7	13	26.1	372	42330	6.1	Putative uncharacterized protein
2255	UPI0000EE3866	7	13	23.5	412	46590	8.4	alpha-parvin
2256	Q14320	7	22	25.7	339	40242	6.8	Protein FAM50A
2256	Q9WV03	7	22	25.7	339	40252	6.8	Protein FAM50A
2257	Q99JW4	7	13	27.4	325	37240	8	LIM and senescent cell antigen-like-containing domain protein 1
2257	P48059	7	13	27.4	325	37251	8	LIM and senescent cell antigen-like-containing domain protein 1
2257	B7Z483	7	13	26.4	337	38422	8	cDNA FLJ55516, highly similar to Particularly interesting new Cys-His protein
2257	A8CVP1	7	13	24.6	362	41608	8	LIM and senescent cell antigen-like domains 1 isoform D
2257	B7Z907	7	13	24.6	362	41571	8.1	cDNA FLJ55509, highly similar to Particularly interesting newCys-His protein
2257	Q4ZJB3	7	13	23	387	44341	7.8	Lims E protein
2257	E9QP62	7	13	23	387	44340	7.9	Uncharacterized protein
2257	B7Z7R3	7	13	23	387	44390	8	cDNA FLJ55165, highly similar to Particularly interesting new Cys-His protein
2258	Q8WXF1-2	7	10	24.9	393	45571	6.6	Isoform 2 of Paraspeckle component 1
2258	B4DWI8	7	10	21.2	463	52580	7.3	cDNA FLJ57805, highly similar to sapiens paraspeckle component 1 (PSPC1), transcript variant alpha, mRNA
2258	Q8R326-2	7	10	21	466	53022	6.1	Isoform 2 of Paraspeckle component 1
2258	Q8WXF1	7	10	18.7	523	58744	6.7	Paraspeckle component 1
2258	Q8R326	7	10	18.7	523	58758	6.7	Paraspeckle component 1
2259	Q8VH51-3	7	34	28.3	367	39905	6.8	Isoform 3 of RNA-binding protein 39
2259	Q0VGU9	7	34	28.3	367	39818	6.6	Rbm39 protein Rbm39 protein
2259	Q7Z3L0	7	34	27.9	373	40469	6.6	Putative uncharacterized protein DKFZp781I1140
2259	E1P5S2	7	34	27.9	373	40541	6.3	RNA-binding region (RNP1, RRM) containing 2, isoform CRA_b
2259	E9PCZ6	7	34	20.8	499	55960	10.3	Uncharacterized protein
2259	B4DRA0	7	34	20.7	502	56411	10	cDNA FLJ58459, highly similar to RNA-binding region-containing protein 2
2259	B4DLM0	7	34	20.7	503	56333	10.4	cDNA FLJ60296, highly similar to RNA-binding region-containing protein 2
2259	B3KWX7	7	34	20.6	506	56744	10.4	cDNA FLJ44170 fis, clone THYMU2035319, highly similar to RNA-binding region-containing protein 2
2259	A2RRD3	7	34	20.5	508	57090	10	RBM39 protein
2259	Q68DD9	7	34	20.3	513	57444	10.3	Putative uncharacterized protein DKFZp781C0423 (Fragment)
2259	Q8VH51-2	7	34	19.8	524	58771	10.2	Isoform 2 of RNA-binding protein 39
2259	Q5RC80	7	34	19.8	524	58657	10.1	RNA-binding protein 39
2259	Q5BJP4	7	34	19.8	524	58684	10.1	RNA binding motif protein 39
2259	Q8VH51	7	34	19.6	530	59494	10.1	RNA-binding protein 39
2259	Q5RJI0	7	34	19.6	530	59407	10.1	Putative uncharacterized protein
2259	Q14498	7	34	19.6	530	59380	10.1	RNA-binding protein 39
2260	B4DGF8	7	55	19.5	462	51106	7.8	cDNA FLJ57877, highly similar to Cleavage and polyadenylation specificity factor 7
2260	Q8N684-2	7	55	19.5	462	51096	7.8	Isoform 2 of Cleavage and polyadenylation specificity factor subunit 7
2260	Q8BTV2-2	7	55	19.5	462	51057	7.8	Isoform 2 of Cleavage and polyadenylation specificity factor subunit 7

Table S1. Proteins identified in the OVCAR-3 tumor supernatant by two or more unique peptides.

Protein or Protein Group	Accession Number	Unique Peptide Count	Spectrum Count	Sequence Coverage (%)	Length	MolWt	pI	Descriptive Name
2260	Q8N684	7	55	19.1	471	52050	8	Cleavage and polyadenylation specificity factor subunit 7
2260	Q8BTV2	7	55	19.1	471	52011	8	Cleavage and polyadenylation specificity factor subunit 7
2260	Q8N684-3	7	55	17.5	514	56375	8.9	Isoform 3 of Cleavage and polyadenylation specificity factor subunit 7
2261	Q96I25	7	16	18.7	401	44962	6	Splicing factor 45
2261	Q8JZX4	7	16	18.5	405	45304	5.8	Splicing factor 45
2262	P56545	7	12	18.2	445	48945	6.9	C-terminal-binding protein 2
2262	Q3UIX8	7	12	18.2	445	48958	6.8	Putative uncharacterized protein
2262	P56546	7	12	18.2	445	48957	6.9	C-terminal-binding protein 2
2262	Q8IY44	7	12	15.8	513	56201	7.1	CTBP2 protein
2262	Q5SQP8	7	12	15.8	513	56102	7	C-terminal binding protein 2
2262	P56546-2	7	12	8.2	988	107126	7.6	Isoform 2 of C-terminal-binding protein 2
2262	P56545-2	7	12	8.2	985	106187	8	Isoform 2 of C-terminal-binding protein 2
2263	O55222	7	11	17.5	452	51373	8.1	Integrin-linked protein kinase
2263	Q13418	7	11	17.5	452	51419	8.1	Integrin-linked protein kinase
2264	E9QMP5	7	15	17.1	451	52456	6.3	Uncharacterized protein
2264	Q8BS26	7	15	17	452	52619	6.3	Putative uncharacterized protein
2264	B3KVH4	7	15	16	480	55629	6.1	cDNA FLJ16549 fis, clone PLACE7003657, highly similar to RAC-alpha serine/threonine-protein kinase (EC 2.7.11.1)
2264	P31749	7	15	16	480	55686	6.1	RAC-alpha serine/threonine-protein kinase
2264	UPI0001553571	7	15	15.8	488	56393	6.3	RAC-alpha serine/threonine-protein kinase isoform 2
2264	P31748	7	15	15.4	501	57870	5.7	AKT kinase-transforming protein
2265	P52594-2	7	17	12.8	522	54182	8.9	Isoform 2 of Arf-GAP domain and FG repeats-containing protein 1
2265	Q8K2K6-3	7	17	12.6	530	55068	8.5	Isoform 3 of Arf-GAP domain and FG repeats-containing protein 1
2265	Q8K2K6-2	7	17	12.4	540	56196	8.7	Isoform 2 of Arf-GAP domain and FG repeats-containing protein 1
2265	B8ZZY2	7	17	12.4	541	56413	8.6	Uncharacterized protein
2265	Q8K2K6-1	7	17	12	559	57915	8.6	Isoform 1 of Arf-GAP domain and FG repeats-containing protein 1
2265	P52594-3	7	17	12	560	58132	8.6	Isoform 3 of Arf-GAP domain and FG repeats-containing protein 1
2265	Q8K2K6	7	17	11.9	561	58043	8.6	Arf-GAP domain and FG repeats-containing protein 1
2265	P52594	7	17	11.9	562	58260	8.6	Arf-GAP domain and FG repeats-containing protein 1
2265	B3KUL1	7	17	11.8	569	58970	8	cDNA FLJ40132 fis, clone TESTI2012155, highly similar to NUCLEOPORIN-LIKE PROTEIN RIP
2265	E9PHX7	7	17	11.5	584	60767	8.6	Uncharacterized protein
2266	B7ZKY2	7	7	10.3	897	102084	6.4	Calcium/calmodulin-dependent serine protein kinase (MAGUK family)
2266	O70589-3	7	7	10.3	897	102099	6.4	Isoform 3 of Peripheral plasma membrane protein CASK
2266	O14936-3	7	7	10.3	897	102114	6.4	Isoform 3 of Peripheral plasma membrane protein CASK
2266	O70589-4	7	7	10.1	908	103145	6.4	Isoform 4 of Peripheral plasma membrane protein CASK
2266	O70589-5	7	7	10	920	104455	6.4	Isoform 5 of Peripheral plasma membrane protein CASK
2266	B7ZP42	7	7	10	920	104445	6.4	Cask protein
2266	O70589	7	7	9.9	926	105109	6.4	Peripheral plasma membrane protein CASK
2266	O14936	7	7	9.9	926	105123	6.4	Peripheral plasma membrane protein CASK
2266	E9QK70	7	7	9.9	926	105186	6.3	Uncharacterized protein
2267	Q96Q05-3	7	8	6.1	1139	127607	6.7	Isoform 3 of Trafficking protein particle complex subunit 9
2267	Q3U0M1-2	7	8	6.1	1139	127308	6.6	Isoform 2 of Trafficking protein particle complex subunit 9
2267	Q96Q05	7	8	6	1148	128530	6.6	Trafficking protein particle complex subunit 9
2267	Q3U0M1	7	8	6	1148	128231	6.5	Trafficking protein particle complex subunit 9
2267	A6NIF0	7	8	5.5	1258	140671	7.3	Uncharacterized protein
2268	Q9JHS3	7	47	90.4	125	13480	5.4	Ragulator complex protein LAMTOR2
2269	P70349	7	21	77.8	126	13777	6.9	Histidine triad nucleotide-binding protein 1
2270	D3YYD5	7	30	73.4	124	13723	7.9	Uncharacterized protein
2271	O35215	7	14	72.9	118	13077	6.5	D-dopachrome decarboxylase
2272	O88653	7	19	71.8	124	13553	7.3	Ragulator complex protein LAMTOR3
2272	UPI0000608A6F	7	19	71.8	124	13569	6.5	PREDICTED: mitogen-activated protein kinase scaffold protein 1-like
2273	P16045	7	320	70.4	135	14866	5.5	Galectin-1
2274	P43025	7	18	61.4	202	22257	5.6	Tetranectin
2274	Q8CFZ6	7	18	61.4	202	22255	5.6	C-type lectin domain family 3, member b
2275	Q921E2	7	11	61.3	194	21331	7.4	Ras-related protein Rab-31
2276	P48024	7	29	61.1	113	12747	7.5	Eukaryotic translation initiation factor 1
2277	Q3UC76	7	15	60.4	144	16475	5.2	Putative uncharacterized protein
2277	Q60872	7	15	60.4	144	16502	5.2	Eukaryotic translation initiation factor 1A
2278	Q9CQU0	7	19	59.4	170	19049	5.3	Thioredoxin domain-containing protein 12
2279	D3YWR7	7	16	59.3	189	20008	6.9	Uncharacterized protein
2279	Q8BVI4	7	16	46.5	241	25570	7.8	Dihydropteridine reductase
2280	P34022	7	25	57.1	203	23596	5.2	Ran-specific GTPase-activating protein
2280	Q3U6M5	7	25	57.1	203	23497	5.2	Putative uncharacterized protein
2281	P04117	7	32	55.3	132	14650	8.4	Fatty acid-binding protein, adipocyte
2282	D3YZL9	7	64	53	132	14489	7.9	40S ribosomal protein S12
2282	UPI000024EED0	7	64	53	132	14490	7.2	PREDICTED: 40S ribosomal protein S12-like
2282	UPI0000022171	7	64	53	132	14504	7.2	PREDICTED: 40S ribosomal protein S12-like isoform 1
2283	P31532	7	10	52.3	130	15088	9.3	Serum amyloid A-4 protein
2284	A2A9X5	7	20	50.5	194	21939	5.3	5',3'-nucleotidase, cytosolic
2284	Q9JM14	7	20	49	200	23076	5.5	5'(3')-deoxyribonucleotidase, cytosolic type
2285	D3Z125	7	48	50.3	179	19417	5.1	Uncharacterized protein
2285	E9PUA7	7	48	46.4	194	21134	5	Uncharacterized protein
2285	Q62393-3	7	48	45.2	199	21654	5	Isoform 3 of Tumor protein D52
2285	D3Z7X7	7	48	43.7	206	22562	5.5	Uncharacterized protein
2285	Q62393	7	48	40.2	224	24313	4.7	Tumor protein D52
2285	E9QMB6	7	48	36.4	247	26927	5	Uncharacterized protein
2286	P63028	7	60	50	172	19462	4.9	Translationally-controlled tumor protein
2287	P12246	7	12	48.7	224	26247	6.4	Serum amyloid P-component
2288	Q07813	7	10	48.4	192	21395	5	Apoptosis regulator BAX
2289	Q9DAC2	7	15	57.1	168	18946	8.3	Complement component 8, gamma subunit
2289	Q8VCG4	7	15	47.5	202	22508	9.2	Complement component C8 gamma chain
2290	O55135	7	31	46.9	245	26511	4.7	Eukaryotic translation initiation factor 6
2290	Q9R071	7	31	46.9	245	26541	4.7	B4 integrin interactor homolog
2290	Q3UA13	7	31	46.9	245	26543	4.7	Putative uncharacterized protein
2290	Q3U818	7	31	46.9	245	26451	4.7	Putative uncharacterized protein
2291	D3Z315	7	12	45.8	227	25370	4.7	Uncharacterized protein
2291	Q9D1J2	7	12	33.8	308	34557	5.1	Putative uncharacterized protein
2291	O89079	7	12	33.8	308	34567	5.1	Coatomer subunit epsilon
2292	P46412	7	48	41.6	226	25274	8.2	Glutathione peroxidase 3
2293	P14901	7	15	38.8	289	32929	6.5	Heme oxygenase 1
2294	O09131	7	17	37.1	240	27498	7.4	Glutathione S-transferase omega-1
2295	Q3TG45	7	20	35.6	253	28526	6.8	Putative uncharacterized protein
2295	Q99JB5	7	20	35	257	29939	6.4	Proteasome (Prosome, macropain) 26S subunit, non-ATPase, 8
2295	Q9CPS5	7	20	31.1	289	32769	6.4	Putative uncharacterized protein
2295	Q8BKP5	7	20	31.1	289	32709	6.4	Putative uncharacterized protein

Table S1. Proteins identified in the OVCAR-3 tumor supernatant by two or more unique peptides.

Protein or Protein Group	Accession Number	Unique Peptide Count	Spectrum Count	Sequence Coverage (%)	Length	MolWt	pI	Descriptive Name
2295	Q3TVY0	7	20	29.3	307	34827	8.9	Putative uncharacterized protein
2295	Q9CX56	7	20	25.5	353	39930	9.6	26S proteasome non-ATPase regulatory subunit 8
2296	Q9QZE7	7	15	33.8	290	32926	6.6	Translin-associated protein X
2296	UPI000155323C	7	15	28.7	341	38489	8.1	PREDICTED: translin-associated protein X isoform 1
2297	P51859	7	70	32.9	237	26269	4.8	Hepatoma-derived growth factor
2298	P70677	7	19	32.5	277	31475	6.9	Caspase-3
2299	Q9CQM9	7	23	31.2	337	37778	5.6	Glutaredoxin-3
2300	Q8CAY6	7	11	31	397	41298	7.5	Acetyl-CoA acetyltransferase, cytosolic
2301	P52430	7	11	30.7	355	39565	5.2	Serum paraoxonase/arylesterase 1
2302	Q8CHP8	7	23	29.3	321	34541	5.4	Phosphoglycolate phosphatase
2303	UPI0001F78DEE	7	50	29.3	273	29022	10	cold shock domain protein A
2303	Q64369	7	50	29.2	274	29314	10.1	Y-box binding protein (Fragment)
2303	Q9JKB3-2	7	50	27.4	292	30765	9.6	Isoform 2 of DNA-binding protein A
2303	Q68G78	7	50	26.8	299	31822	10.3	Csda protein
2304	O35955	7	8	28.6	273	29063	6.9	Proteasome subunit beta type-10
2304	Q4FJX8	7	8	28.6	273	29118	7.2	Proteasome subunit beta type
2304	Q3UNP9	7	8	28.6	273	29081	6.9	Proteasome subunit beta type
2305	Q3TAI4	7	60	31.6	206	22466	5.7	Putative uncharacterized protein
2305	Q9CZ22	7	60	29.8	218	23859	5.9	Putative uncharacterized protein
2305	Q9CYZ2	7	60	29.5	220	24043	6.1	Tumor protein D54
2305	A2AUD5	7	60	28.4	229	25067	6.5	Tumor protein D52-like 2
2306	Q8BJL4	7	10	28.2	358	40430	7	Lectin, mannose-binding 2
2306	Q9DBH5	7	10	28.2	358	40416	7	Vesicular integral-membrane protein VIP36
2307	Q6WVG3	7	7	27.2	327	35892	5.8	BTB/POZ domain-containing protein KCTD12
2308	P47968	7	13	27.1	303	32451	8	Ribose-5-phosphate isomerase
2309	Q8VCN0	7	9	26.2	477	51985	5.3	Agt protein
2310	P57759	7	13	25.6	262	28823	6.1	Endoplasmic reticulum resident protein 29
2311	Q9DC51	7	14	24.9	354	40538	5.7	Guanine nucleotide-binding protein G(k) subunit alpha
2312	Q91V12-4	7	20	24.3	379	41616	7.7	Isoform D of Cytosolic acyl coenzyme A thioester hydrolase
2312	Q91V12	7	20	24.1	381	42537	8.7	Cytosolic acyl coenzyme A thioester hydrolase
2313	Q9WUB3	7	8	24	842	97286	7.1	Glycogen phosphorylase, muscle form
2314	Q9R0N0	7	9	22.8	391	42176	5.3	Galactokinase
2314	Q80UL3	7	9	22.7	392	42309	5.3	Galactokinase 1
2314	Q9JIA6	7	9	22.7	392	42295	5.3	Galactokinase
2314	Q9CXZ9	7	9	22.7	392	42261	5.3	Putative uncharacterized protein
2315	O35344	7	13	21.7	521	57773	4.9	Importin subunit alpha-3
2316	P16675	7	23	19.8	474	53844	5.9	Lysosomal protective protein
2316	Q9D2D1	7	23	19.8	474	53830	5.9	Putative uncharacterized protein
2317	P13439	7	22	18.5	481	52292	6.6	Uridine 5'-monophosphate synthase
2317	Q3TVV7	7	22	18.5	481	52193	6.5	Putative uncharacterized protein
2318	E9PW67	7	15	18	523	60022	6.5	Uncharacterized protein
2318	Q3TXV7	7	15	18	521	59741	6.7	Putative uncharacterized protein
2318	Q91XG3	7	15	17.8	528	60613	6.5	Hexosaminidase A
2318	Q8BNS6	7	15	17.8	528	60553	6.5	Putative uncharacterized protein
2318	Q3UCP6	7	15	17.8	528	60682	6.7	Putative uncharacterized protein
2318	Q3U936	7	15	17.8	528	60579	6.5	Putative uncharacterized protein
2318	Q3TW10	7	15	17.8	528	60579	6.5	Putative uncharacterized protein
2318	Q3TVI2	7	15	17.8	528	60583	6.5	Putative uncharacterized protein
2319	Q9CT10	7	12	17.9	491	52573	5.1	Ran-binding protein 3
2320	Q9DBB9	7	16	17.6	547	60479	5.9	Carboxypeptidase N subunit 2
2321	Q1KYL8	7	10	15.3	537	60568	8	Gag polyprotein pr65
2321	Q1KYL9	7	10	4.7	1734	193994	8.9	Gag-pro-pol polyprotein
2322	P33434	7	11	15.1	662	74102	5.5	72 kDa type IV collagenase
2323	Q99KI0	7	7	14.4	780	85464	7.9	Aconitate hydratase, mitochondrial
2324	P70356	7	12	14.1	669	73666	8.2	Envelope protein
2324	Q61919	7	12	14.1	669	73626	8.1	Envelope protein
2324	Q60589	7	12	14.1	669	73825	8.1	Envelope glycoprotein
2324	Q2HJ08	7	12	14.1	669	73804	8	Melanoma antigen
2324	Q1KYM0	7	12	14.1	669	73698	8	Env polyprotein
2324	Q04586	7	12	14.1	669	73728	8.2	Env polyprotein
2324	UPI0001E6B600	7	12	13.7	687	75820	8.1	PREDICTED: envelope glycoprotein-like
2325	P06683	7	20	13.5	548	62002	5.8	Complement component C9
2326	P26262	7	10	12.5	638	71369	8	Plasma kallikrein
2326	Q8R0P5	7	10	12.5	638	71383	8	Kallikrein B, plasma 1
2327	P32020-2	7	13	47.6	143	15237	9.3	Isoform SCP2 of Non-specific lipid-transfer protein
2327	P32020	7	13	12.4	547	59126	7.4	Non-specific lipid-transfer protein
2328	UPI0001F78EBD	7	32	12.4	716	79203	4.9	neural cell adhesion molecule 1
2328	E9Q589	7	32	12.3	725	80358	4.9	Uncharacterized protein
2328	P13595-3	7	32	12.3	725	80406	4.9	Isoform 3 of Neural cell adhesion molecule 1
2328	UPI0001F78EBC	7	32	10.6	839	92337	4.9	neural cell adhesion molecule 1
2328	P13595-2	7	32	10.5	848	93540	4.9	Isoform 2 of Neural cell adhesion molecule 1
2328	E9QB01	7	32	10.5	848	93492	4.9	Uncharacterized protein
2328	UPI0001F78EBB	7	32	8	1106	118143	4.8	neural cell adhesion molecule 1
2328	UPI000162ECA8	7	32	8	1115	119298	4.8	neural cell adhesion molecule 1 isoform 3
2328	P13595	7	32	8	1115	119427	4.8	Neural cell adhesion molecule 1
2329	Q6NXL1	7	9	8.2	1032	112676	7.2	Sec24 related gene family, member D (S. cerevisiae)
2330	A2AP28	7	14	7.9	926	105975	6.6	DEAD (Asp-Glu-Ala-Asp) box polypeptide 58
2330	A7YY79	7	14	7.9	925	105786	6.7	Ddx58 protein (Fragment)
2331	P97449	7	10	6.9	966	109651	5.9	Aminopeptidase N
2332	Q3TPN0	7	17	6.4	1403	153943	5.4	Putative uncharacterized protein
2332	Q8CFA3	7	17	6.4	1403	153912	5.4	Nidogen-2
2332	Q7TQF0	7	17	6.4	1403	153913	5.4	Nidogen 2
2333	Q9Y2Q5	7	49	90.4	125	13507	5.4	Ragulator complex protein LAMTOR2
2334	Q99627	7	23	75.6	209	23226	5.4	COP9 signalosome complex subunit 8
2335	Q9Y3D0	7	15	74.2	163	17663	5.2	Mitotic spindle-associated MMXD complex subunit MIP18
2336	P50583	7	32	72.1	147	16829	5.3	Bis(5'-nucleosyl)-tetraphosphatase [asymmetrical]
2337	Q9Y547	7	42	70.8	144	16297	5	Heat shock protein beta-11
2338	A2VDF0	7	27	70.1	154	16765	5.6	Fucose mutarotase
2339	Q969G6	7	24	66.5	155	17623	8.1	Riboflavin kinase
2340	P04080	7	83	66.3	98	11140	7.6	Cystatin-B
2341	Q8NFU3-4	7	25	65.6	122	13189	7.2	Isoform 4 of Thiosulfate sulfurtransferase/rhodanese-like domain-containing protein 1
2342	Q6GMV3	7	18	65	140	15805	9.1	Uncharacterized protein C2orf79
2343	Q6IB68	7	18	65	103	11953	5.9	MYCBP protein
2343	Q99417	7	18	65	103	11967	5.9	C-Myc-binding protein
2344	Q9Y237	7	66	63.4	131	13810	9.8	Peptidyl-prolyl cis-trans isomerase NIMA-interacting 4
2345	O14737	7	38	61.6	125	14285	6	Programmed cell death protein 5
2346	Q53HV6	7	31	61.6	151	16584	7.7	Niemann-Pick disease, type C2 variant (Fragment)
2347	O43598	7	70	60.9	174	19108	5	Deoxyribonucleoside 5'-monophosphate N-glycosidase
2347	UPI0001D3BABD	7	70	43.6	243	25868	5.5	UPI0001D3BABD UniRef100 entry
2348	Q8N668	7	12	59.5	190	21178	6.2	COMM domain-containing protein 1
2349	Q5JS54	7	23	78	123	13775	6.5	Proteasome assembly chaperone 4
2349	C9J2F8	7	23	59.3	162	17656	6.2	HCG22882, isoform CRA_a

Table S1. Proteins identified in the OVCAR-3 tumor supernatant by two or more unique peptides.

Protein or Protein Group	Accession Number	Unique Peptide Count	Spectrum Count	Sequence Coverage (%)	Length	MolWt	pI	Descriptive Name
2350	B4DNR3	7	22	58.9	185	19797	6.4	cDNA FLJ52710, highly similar to Abhydrolase domain-containing protein 14B
2350	Q96IU4	7	22	51.9	210	22346	6.4	Abhydrolase domain-containing protein 14B
2351	Q9H0U4	7	88	58.7	201	22171	5.7	Ras-related protein Rab-1B
2352	Q96C90	7	33	58.5	147	15911	4.9	Protein phosphatase 1 regulatory subunit 14B
2353	Q0VDC6	7	64	57.9	145	15689	9.2	FKBP1A protein
2354	O95295	7	19	56.6	136	14874	9.3	SNARE-associated protein Snapin
2355	Q9Y5S9	7	41	56.3	174	19889	5.7	RNA-binding protein 8A
2356	P45877	7	21	55.7	212	22763	8.4	Peptidyl-prolyl cis-trans isomerase C
2357	Q9BQI0	7	94	55.3	150	17068	7.2	Allograft inflammatory factor 1-like
2357	UPI0001AF80B6	7	94	55.3	150	17068	7.2	IONIZED CALCIUM-BINDING ADAPTER MOLECULE 2
2357	Q9BQI0-2	7	94	47.2	176	20015	7.8	Isoform 2 of Allograft inflammatory factor 1-like
2358	Q96LR5	7	28	55.2	201	22255	7.7	Ubiquitin-conjugating enzyme E2 E2
2359	UPI0000EE787D	7	15	57.6	210	22154	6.5	PREDICTED: Parkinson disease 7 domain-containing protein 1-like
2359	Q8NB37	7	15	55	220	23298	6.6	Parkinson disease 7 domain-containing protein 1
2360	P19388	7	19	54.3	210	24551	6	DNA-directed RNA polymerases I, II, and III subunit RPABC1
2361	Q9Y6A4	7	18	53.9	193	22774	9.8	UPF0468 protein C16orf80
2362	P30043	7	19	52.9	206	22119	7.6	Flavin reductase
2363	P53999	7	68	52.8	127	14395	9.6	Activated RNA polymerase II transcriptional coactivator p15
2364	Q6QNY1	7	9	52.1	142	15961	4.9	Biogenesis of lysosome-related organelles complex 1 subunit 2
2365	Q96GD7	7	20	51.8	303	31931	9.7	CSDA protein
2366	P82979	7	46	51.4	210	23671	6.4	SAP domain-containing ribonucleoprotein
2367	Q9Y5Y2	7	13	50.6	271	28825	5.8	Cytosolic Fe-S cluster assembly factor NUBP2
2368	A8K646	7	20	50	214	23715	5.7	cDNA FLJ75699, highly similar to sapiens osteoclast stimulating factor 1 (OSTF1), mRNA
2368	Q92882	7	20	50	214	23787	5.7	Osteoclast-stimulating factor 1
2369	Q96CD2	7	8	50	204	22395	6.1	Phosphopantothenoylcysteine decarboxylase
2370	Q9NWU2	7	9	48.2	228	26749	5	Protein C20orf11
2371	P06899	7	115	47.6	126	13904	10.3	Histone H2B type 1-J
2371	Q16778	7	115	47.6	126	13920	10.3	Histone H2B type 2-E
2371	P33778	7	115	47.6	126	13950	10.3	Histone H2B type 1-B
2371	P23527	7	115	47.6	126	13906	10.3	Histone H2B type 1-O
2372	Q8N1Q1	7	9	46.9	262	29443	7	Carbonic anhydrase 13
2373	P00374	7	11	46	187	21453	7.4	Dihydrofolate reductase
2374	Q9BV19	7	10	45.7	199	21877	5.9	Uncharacterized protein C1orf50
2375	P26447	7	203	45.5	101	11728	6.1	Protein S100-A4
2376	UPI0001F77AB9	7	15	47.4	228	25199	5.9	UPI0001F77AB9 UniRef100 entry
2376	Q6NVV8	7	15	46.8	231	25635	6.6	IAH1 protein (Fragment)
2376	Q05D21	7	15	45	240	26443	6.5	IAH1 protein (Fragment)
2376	Q2TAA2	7	15	43.5	248	27599	5.3	Isoamyl acetate-hydrolyzing esterase 1 log
2377	Q96MW1	7	16	44.6	224	25248	4.9	Coiled-coil domain-containing protein 43
2378	E9PL57	7	33	44.1	170	19536	7.4	Uncharacterized protein
2379	O43583	7	34	42.9	198	22092	5.3	Density-regulated protein
2380	E9PPJ5	7	30	48.9	131	14374	9.5	Uncharacterized protein
2380	P21741	7	30	44.8	143	15585	9.8	Midkine
2380	E9PLM6	7	30	41	156	16926	9.9	Uncharacterized protein
2381	B2R6P4	7	13	40.5	220	24995	8.6	cDNA, FLJ93048, highly similar to sapiens synaptobrevin-like 1 (SYBL1), mRNA
2381	P51809	7	13	40.5	220	24935	8.6	Vesicle-associated membrane protein 7
2382	Q5RKV6	7	8	39.7	272	28235	6.3	Exosome complex exonuclease MTR3
2383	P46779	7	25	39.4	137	15747	12	60S ribosomal protein L28
2384	P28065	7	14	38.8	219	23264	5	Proteasome subunit beta type-9
2385	Q9BZX2	7	13	38.3	261	29299	6.7	Uridine-cytidine kinase 2
2386	P16455	7	36	44	207	21646	8.1	Methylated-DNA--protein-cysteine methyltransferase
2386	B4DEE8	7	36	38.2	238	25050	8.7	cDNA FLJ56323, highly similar to Methylated-DNA--protein-cysteinemethyltransferase (EC 2.1.1.63)
2387	B3KM36	7	7	37.4	211	23712	6.7	cDNA FLJ10153 fis, clone HEMBA1003417, highly similar to BAG family molecular chaperone regulator 2
2387	O95816	7	7	37.4	211	23772	6.7	BAG family molecular chaperone regulator 2
2387	B3KN59	7	7	37.4	211	23748	6.6	cDNA FLJ13673 fis, clone PLACE1011858, highly similar to BAG family molecular chaperone regulator 2
2388	A6NDU8	7	11	37.1	294	33620	5.3	UPF0600 protein C5orf51
2389	Q6UWP2	7	8	36.9	260	28308	6.6	Dehydrogenase/reductase SDR family member 11
2390	Q9NT62	7	18	36.9	314	35864	4.7	Ubiquitin-like-conjugating enzyme ATG3
2391	Q6ZVX7	7	17	35.3	275	30847	6.6	Non-specific cytotoxic cell receptor protein 1 log
2392	Q7Z531	7	9	37.2	215	24312	8.7	GTP:ATP phosphotransferase
2392	Q7Z4Y4	7	9	35.2	227	25620	9.3	GTP:AMP phosphotransferase
2392	Q9UIJ7	7	9	35.2	227	25565	9.2	GTP:AMP phosphotransferase, mitochondrial
2393	P50402	7	22	35	254	28994	5.5	Emerin
2394	D3DVF0	7	26	35.1	296	32228	7.1	F11 receptor, isoform CRA_a
2394	Q9Y624	7	26	34.8	299	32583	7.9	Junctional adhesion molecule A
2394	B7Z5W1	7	26	34.3	303	32858	8.9	cDNA FLJ54854, highly similar to Junctional adhesion molecule A
2395	B2RCM6	7	15	34	253	28194	9.5	cDNA, FLJ96161
2395	Q96S44	7	15	34	253	28160	9.5	TP53-regulating kinase
2396	P41236	7	14	33.2	205	23015	4.7	Protein phosphatase inhibitor 2
2397	B7Z6B3	7	42	39.1	156	17758	8.6	cDNA FLJ53094, highly similar to Receptor expression-enhancing protein 5
2397	B2R6C4	7	42	33	185	21117	8.1	cDNA, FLJ92888, highly similar to sapiens likely ortholog of mouse deleted in polyposis 1 (DP1), mRNA
2397	Q00765	7	42	32.3	189	21493	8.1	Receptor expression-enhancing protein 5
2398	Q14696	7	15	32.9	234	26077	7.8	LDLR chaperone MESD
2399	Q92876	7	12	32.8	244	26856	7.4	Kallikrein-6
2400	Q9Y657	7	14	32.4	262	29601	7	Spindlin-1
2401	Q5QPM7	7	30	32.3	263	28957	6.4	Proteasome (Prosome, macropain) inhibitor subunit 1 (PI31)
2401	Q92530	7	30	31.4	271	29817	5.7	Proteasome inhibitor PI31 subunit
2402	A9UHW6	7	13	32	222	25423	5.3	MIF4G domain-containing protein
2402	UPI0000456B02	7	13	23.7	299	33661	8.5	MIF4G domain containing
2403	Q9NPD3	7	14	31.8	245	26383	6.5	Exosome complex exonuclease RRP41
2404	B3KNT8	7	38	31.5	368	42762	4.6	cDNA FLJ30458 fis, clone BRACE2009421, highly similar to NUCLEOSOME ASSEMBLY PROTEIN 1-LIKE 1
2404	B7Z2V4	7	38	30.3	383	44600	4.6	cDNA FLJ60539, highly similar to Nucleosome assembly protein 1-like 1
2404	P55209	7	38	29.7	391	45374	4.5	Nucleosome assembly protein 1-like 1
2405	P46976-2	7	23	33	333	37479	5.4	Isoform GN-1 of Glycogenin-1
2405	B2R5R5	7	23	31.4	350	39354	5.5	cDNA, FLJ92583, highly similar to sapiens glycogenin (GYG), mRNA
2405	P46976	7	23	31.4	350	39384	5.5	Glycogenin-1
2406	Q6IPC0	7	12	30.7	453	49702	5.1	Protein phosphatase 1F (PP2C domain containing)
2406	P49593	7	12	30.6	454	49831	5.1	Protein phosphatase 1F
2407	Q9Y296	7	11	35.6	219	24340	6.2	Trafficking protein particle complex subunit 4
2407	E9PN70	7	11	30.4	257	28818	8.5	Uncharacterized protein

Table S1. Proteins identified in the OVCAR-3 tumor supernatant by two or more unique peptides.

Protein or Protein Group	Accession Number	Unique Peptide Count	Spectrum Count	Sequence Coverage (%)	Length	MolWt	pI	Descriptive Name
2408	Q9BQ67	7	9	30.3	446	49419	4.9	Glutamate-rich WD repeat-containing protein 1
2409	A8MZF9	7	7	30	343	38129	8.9	Uncharacterized protein
2409	Q53Y50	7	7	28.3	364	40777	8.9	Developmentally regulated GTP binding protein 2
2409	P55039	7	7	28.3	364	40746	8.9	Developmentally-regulated GTP-binding protein 2
2410	O75818-2	7	9	32.1	340	39327	7	Isoform 2 of Ribonuclease P protein subunit p40
2410	O75818	7	9	30	363	41834	6.7	Ribonuclease P protein subunit p40
2411	P35249	7	7	30	363	39682	8	Replication factor C subunit 4
2412	O00442	7	15	29.8	366	39337	7.9	RNA 3'-terminal phosphate cyclase
2412	O00442-2	7	15	28.8	379	40709	8.3	Isoform 2 of RNA 3'-terminal phosphate cyclase
2413	Q96EP5-2	7	17	31.5	378	40530	8.3	Isoform 2 of DAZ-associated protein 1
2413	Q96EP5	7	17	29.2	407	43383	8.6	DAZ-associated protein 1
2414	Q9H479	7	10	29.1	309	35171	7.6	Fructosamine-3-kinase
2415	Q13287	7	8	28.7	307	35057	5.3	N-myc-interactor
2415	Q8WTW2	7	8	28.7	307	35085	5.3	N-myc (And STAT) interactor
2416	Q8WYQ7	7	23	29.3	311	34691	8.1	Galectin-9
2416	B3KWW5	7	23	28.2	323	35918	8.1	cDNA FLJ44029 fis, clone TESTI4027557, highly similar to Galectin-9
2416	Q53FQ0	7	23	28.2	323	35858	8.1	Galectin 9 short isoform variant (Fragment)
2416	Q3B8N1	7	23	28.2	323	35888	8.1	Lectin, galactoside-binding, soluble, 9
2416	O00182	7	23	25.6	355	39518	9.2	Galectin-9
2417	P42126-2	7	8	29.8	285	30896	8.9	Isoform 2 of 3,2-trans-enoyl-CoA isomerase, mitochondrial
2417	P42126	7	8	28.1	302	32816	8.6	3,2-trans-enoyl-CoA isomerase, mitochondrial
2418	B2RA70	7	15	26.9	543	60831	6.7	cDNA, FLJ94729, highly similar to sapiens v-yes-1 Yamaguchi sarcoma viral oncogene log 1(YES1), mRNA
2418	P07947	7	15	26.9	543	60801	6.7	Proto-oncogene tyrosine-protein kinase Yes
2419	B3KY04	7	7	26.8	325	35731	5.6	cDNA FLJ46506 fis, clone THYMU3030752, highly similar to BTB/POZ domain-containing protein KCTD12
2419	Q96CX2	7	7	26.8	325	35701	5.6	BTB/POZ domain-containing protein KCTD12
2420	Q9H0E2	7	13	34.3	274	30282	6	Toll-interacting protein
2420	Q59FB9	7	13	26.7	352	38895	8.5	Toll interacting protein variant (Fragment)
2421	P16422	7	17	28.7	314	34932	7.5	Epithelial cell adhesion molecule
2421	B5MCA4	7	17	26.3	342	37893	8.5	Uncharacterized protein
2422	Q96EE3	7	9	24.7	360	39649	8.1	Nucleoporin SEH1
2422	Q96EE3-1	7	9	21.1	421	46578	7.9	Isoform B of Nucleoporin SEH1
2423	Q13057	7	10	24.1	564	62329	7	Bifunctional coenzyme A synthase
2424	P02649	7	17	23	317	36154	5.7	Apolipoprotein E
2425	Q9BSL1	7	10	22.7	405	45338	4.9	Ubiquitin-associated domain-containing protein 1
2426	B4DTA9	7	11	24	429	48089	6.4	cDNA FLJ59753
2426	A8K5C2	7	11	22.6	456	51075	6.4	cDNA FLJ75055
2427	Q9H788	7	14	22	454	52727	8.1	SH2 domain-containing protein 4A
2428	E7EV16	7	19	27.9	398	44135	6.5	Uncharacterized protein
2428	B4DRB4	7	19	27.9	398	44163	6.5	cDNA FLJ57173, highly similar to Phospholipid transfer protein
2428	P55058-2	7	19	25.2	441	49272	6.6	Isoform 2 of Phospholipid transfer protein
2428	Q53H91	7	19	22.5	493	54707	7	Phospholipid transfer protein isoform a variant (Fragment)
2428	P55058	7	19	22.5	493	54739	7	Phospholipid transfer protein
2428	B3KUE5	7	19	21.6	513	56635	6.6	Phospholipid transfer protein, isoform CRA_c
2429	B4DKK3	7	17	21.6	366	42018	7.8	cDNA FLJ54791, highly similar to DnaJ log subfamily C member 7
2429	B7Z9W6	7	17	18	438	50067	8.1	cDNA, FLJ78980, highly similar to DnaJ log subfamily C member 7
2429	Q59EH7	7	17	16.4	483	55418	7.5	DnaJ (Hsp40) log, subfamily C, member 7 variant (Fragment)
2429	Q99615	7	17	16	494	56441	7	DnaJ log subfamily C member 7
2430	Q9NR12	7	24	20.8	457	49845	8.4	PDZ and LIM domain protein 7
2431	B4DL54	7	13	19.3	471	52785	5.9	cDNA FLJ59570, highly similar to Protein farnesyltransferase subunit beta (EC 2.5.1.58)
2432	B3KP31	7	11	19.2	478	51489	6.8	cDNA FLJ31047 fis, clone HSYRA2000424, highly similar to Carbohydrate kinase-like protein (EC 2.7.1.-)
2432	Q9UHJ6	7	11	19.2	478	51491	6.8	Sedoheptulokinase
2433	E9PDK5	7	15	21.8	412	45728	8.5	Uncharacterized protein
2433	B4DVE7	7	15	19.1	472	51242	7.7	cDNA FLJ58379, highly similar to Annexin A11
2433	P50995	7	15	17.8	505	54390	7.7	Annexin A11
2434	Q9BZG8	7	11	19.4	443	48805	8.2	Diphthamide biosynthesis protein 1
2434	B4DNK0	7	11	19	453	50246	8.3	cDNA FLJ55582, highly similar to sapiens DPH1 log (DPH1), mRNA
2434	E7ENH3	7	11	19	453	50274	8.2	Uncharacterized protein
2435	B3KU60	7	12	18.6	505	58310	5.2	cDNA FLJ39235 fis, clone OCBBF2007829, highly similar to Mus musculus fatso protein
2435	Q9C0B1	7	12	18.6	505	58282	5.2	Alpha-ketoglutarate-dependent dioxygenase FTO
2436	B2R6S9	7	8	18.5	357	41480	8.9	cDNA, FLJ93097, highly similar to sapiens low density lipoprotein receptor-related protein associated protein 1 (LRPAP1), mRNA
2436	P30533	7	8	18.5	357	41466	8.8	Alpha-2-macroglobulin receptor-associated protein
2437	E5KMT6	7	8	18.5	399	44377	7.7	Pseudouridine synthase
2437	Q9Y606	7	8	17.3	427	47470	8.4	tRNA pseudouridine synthase A, mitochondrial
2438	O00515	7	26	17.8	517	57131	9.7	Ladinin-1
2438	E9PDI4	7	26	17.3	531	58678	9.9	Uncharacterized protein
2439	Q96EK5	7	10	17.1	621	71814	5.5	KIF1-binding protein
2440	B2RDV7	7	8	16.8	650	72620	8.1	cDNA, FLJ96789, highly similar to sapiens dihydrouridine synthase 3-like (S. cerevisiae) (DUS3L), mRNA
2440	Q96G46	7	8	16.8	650	72594	8.1	tRNA-dihydrouridine synthase 3-like
2441	Q9NXV6	7	11	16.7	580	61125	9	CDKN2A-interacting protein
2442	Q9P2G3	7	13	15.8	628	70714	6.7	Kelch-like protein 14
2443	Q6FHF7	7	12	15.2	567	65042	5.7	RABGGTA protein (Fragment)
2443	Q92696	7	12	15.2	567	65072	5.7	Geranylgeranyl transferase type-2 subunit alpha
2444	B5MCF9	7	13	14.4	571	66078	7.5	Uncharacterized protein
2444	O00541-2	7	13	14.1	583	67456	7.5	Isoform 2 of Pescadillo log
2444	O00541	7	13	13.9	588	68003	7.3	Pescadillo log
2445	B4DLY4	7	7	17.1	674	73177	7	cDNA FLJ52327, highly similar to Aconitate hydratase, mitochondrial (EC 4.2.1.3)
2445	B4DJW1	7	7	16.5	697	75949	7.5	cDNA FLJ54329, highly similar to Aconitate hydratase, mitochondrial(EC 4.2.1.3)
2445	B4DZ08	7	7	15.1	761	83413	7.7	cDNA FLJ51705, highly similar to Aconitate hydratase, mitochondrial (EC 4.2.1.3)
2445	Q99798	7	7	14.7	780	85425	7.6	Aconitate hydratase, mitochondrial
2445	B2RBW5	7	7	14.7	780	85453	7.6	cDNA, FLJ95737, highly similar to sapiens aconitase 2, mitochondrial (ACO2), nuclear geneencoding mitochondrial protein, mRNA
2445	A2A274	7	7	14.3	805	87820	7.4	Aconitase 2, mitochondrial
2446	Q9Y5P4-2	7	10	17.9	598	68007	5.5	Isoform 2 of Collagen type IV alpha-3-binding protein
2446	Q9Y5P4	7	10	17.1	624	70835	5.5	Collagen type IV alpha-3-binding protein
2446	B3KUB7	7	10	14.2	752	83708	6.3	Collagen, type IV, alpha 3 (Goodpasture antigen) binding protein, isoform CRA_c

Table S1. Proteins identified in the OVCAR-3 tumor supernatant by two or more unique peptides.

Protein or Protein Group	Accession Number	Unique Peptide Count	Spectrum Count	Sequence Coverage (%)	Length	MolWt	pI	Descriptive Name
2447	Q15057	7	11	14	778	88029	6.8	Arf-GAP with coiled-coil, ANK repeat and PH domain-containing protein 2
2448	Q9ULE6	7	8	14	856	96754	6.5	Paladin
2449	Q96RQ9	7	16	14.5	567	62881	8.7	L-amino-acid oxidase
2449	A2RRH1	7	16	13.9	589	65358	8.5	Interleukin 4 induced 1
2449	Q1WMJ3	7	16	13.9	589	65328	8.5	HCG2043070
2450	Q08AM6	7	13	13.7	782	87973	6.1	Protein VAC14 log
2451	Q9UKN8	7	20	12.5	822	91982	6.7	General transcription factor 3C polypeptide 4
2452	A8K0P8	7	7	11.6	756	87057	5.7	cDNA FLJ78223
2452	Q68CQ4	7	7	11.6	756	87055	5.9	Digestive organ expansion factor log
2453	O75843	7	15	11.3	785	87117	6.6	AP-1 complex subunit gamma-like 2
2454	O94776	7	11	10.6	668	75023	9.7	Metastasis-associated protein MTA2
2455	UPI000150DAB3	7	10	12.3	767	85452	7	epithelial discoidin domain-containing receptor 1 isoform 5 precursor
2455	Q1XI08	7	10	10.7	876	97174	6.5	Discoidin receptor tyrosine kinase isoform b
2455	Q08345	7	10	10.3	913	101128	6.8	Epithelial discoidin domain-containing receptor 1
2455	Q08345-5	7	10	10.2	919	101796	6.8	Isoform 4 of Epithelial discoidin domain-containing receptor 1
2456	Q15386	7	7	10.3	1083	123923	6.7	Ubiquitin-protein ligase E3C
2457	B2RTR1	7	18	9.8	1515	165854	8.3	CLASP2 protein
2457	UPI00004C2784	7	18	9.8	1506	164886	8.2	CLIP-associating protein 2
2457	E7EW49	7	18	9.8	1514	165698	8.2	Uncharacterized protein
2457	E7ERI8	7	18	9.8	1513	165627	8.2	Uncharacterized protein
2458	Q6NT75	7	11	9.5	1215	131420	5.3	HDAC6 protein
2458	Q9UBN7	7	11	9.5	1215	131419	5.3	Histone deacetylase 6
2459	Q6S8J3	7	416	8.9	1075	121363	6.2	POTE ankyrin domain family member E
2460	B2RBM8	7	15	8.3	1102	123616	7.4	cDNA, FLJ95596, highly similar to sapiens activity-dependent neuroprotector (ADNP), mRNA
2460	Q9H2P0	7	15	8.3	1102	123563	7.3	Activity-dependent neuroprotector homeobox protein
2460	Q6DHZ8	7	15	8.3	1102	123447	7.4	Activity-dependent neuroprotector homeobox
2461	E9PJI8	7	9	7.9	1195	127430	6.5	Uncharacterized protein
2461	A7E261	7	9	7.4	1288	137429	6.6	5-oxoprolinase (ATP-hydrolysing)
2461	O14841	7	9	7.4	1288	137457	6.6	5-oxoprolinase
2461	C9JLE8	7	9	7.4	1287	137370	6.6	Uncharacterized protein
2462	Q7Z478	7	12	6.7	1369	155235	8.1	ATP-dependent RNA helicase DHX29
2463	B7ZM70	7	11	7.4	1122	124400	5.8	Putative uncharacterized protein
2463	B3KXB6	7	11	6.4	1299	143904	5.6	cDNA FLJ45101 fis, clone BRAWH3032298, highly similar to Tenascin-N
2463	Q9UQP3	7	11	6.4	1299	144034	5.6	Tenascin-N
2464	Q1RMC7	7	8	6.2	1551	169654	6.2	ROBO1 protein
2464	E9PD49	7	8	6.2	1551	169660	6.2	Uncharacterized protein
2464	B2RXI1	7	8	6	1606	175835	6.1	ROBO1 protein
2464	Q9Y6N7-4	7	8	6	1612	176570	6.1	Isoform 4 of Roundabout log 1
2464	Q2M1J3	7	8	6	1607	175892	6.1	ROBO1 protein
2464	Q1RMC8	7	8	6	1606	175829	6.1	ROBO1 protein
2464	UPI00006C0726	7	8	5.9	1615	176725	6.2	PREDICTED: roundabout log 1-like isoform 2
2464	Q9Y6N7-3	7	8	5.9	1615	176813	6.1	Isoform 3 of Roundabout log 1
2464	Q9Y6N7-2	7	8	5.8	1655	181327	6.1	Isoform 2 of Roundabout log 1
2464	Q9Y6N7	7	8	5.8	1651	180928	6	Roundabout log 1
2465	Q7LBC6	7	20	5.5	1761	191580	7.2	Lysine-specific demethylase 3B
2466	O43157-2	7	9	5.9	1952	214409	5.8	Isoform 2 of Plexin-B1
2466	O43157	7	9	5.4	2135	232296	5.5	Plexin-B1
2467	Q14CF1	7	8	5.6	1722	192074	6.5	TRIP12 protein
2467	Q14669	7	8	4.9	1992	220432	8.5	Probable E3 ubiquitin-protein ligase TRIP12
2467	D4HL82	7	8	4.8	2025	223928	8.4	ULF
2467	Q14CA3	7	8	4.8	2040	225518	8.7	TRIP12 protein
2468	Q14676-4	7	26	7.1	1066	116634	4.9	Isoform 4 of Mediator of DNA damage checkpoint protein 1
2468	E7EVA7	7	26	4.2	1802	195957	5.2	Uncharacterized protein
2468	Q14676-3	7	26	4.2	1802	195985	5.2	Isoform 3 of Mediator of DNA damage checkpoint protein 1
2468	Q14676-2	7	26	4.2	1825	197597	5.7	Isoform 2 of Mediator of DNA damage checkpoint protein 1
2468	E9PGY5	7	26	4.2	1825	197569	5.7	Uncharacterized protein
2468	Q14676	7	26	3.6	2089	226664	5.5	Mediator of DNA damage checkpoint protein 1
2469	E5RHG8	6	48	73	89	9960	5.4	Uncharacterized protein
2469	Q15369	6	48	58	112	12473	4.8	Transcription elongation factor B polypeptide 1 Transcription elongation factor B polypeptide 1
2470	P04350	6	190	69.1	444	49586	4.9	Tubulin beta-4 chain Tubulin beta-4 chain
2471	O70569	6	58	62.9	151	16301	10.1	Ribosomal protein S14
2471	P62263	6	58	62.9	151	16273	10.1	40S ribosomal protein S14 40S ribosomal protein S14
2472	O15514	6	28	62	142	16311	4.8	DNA-directed RNA polymerase II subunit RPB4 DNA-directed RNA polymerase II subunit RPB4
2473	P52434	6	36	59.3	150	17143	4.7	DNA-directed RNA polymerases I, II, and III subunit RPABC3 DNA-directed RNA polymerases I, II, and III subunit RPABC3
2474	P62888	6	110	59.1	115	12784	9.6	60S ribosomal protein L30 60S ribosomal protein L30
2474	UPI00001C64B1	6	110	59.1	115	12830	9.6	PREDICTED: 60S ribosomal protein L30-like
2474	UPI0000024F43	6	110	59.1	115	12832	9.6	PREDICTED: 60S ribosomal protein L30-like
2475	Q6IAA8	6	42	57.8	161	17745	5.1	Ragulator complex protein LAMTOR1
2475	Q9CQ22	6	42	57.8	161	17749	5.1	Ragulator complex protein LAMTOR1
2476	P30050	6	108	54.5	165	17819	9.4	60S ribosomal protein L12
2476	UPI000002163C	6	108	54.5	165	17792	9.4	PREDICTED: 60S ribosomal protein L12-like
2476	Q3TIQ2	6	108	54.5	165	17775	9.4	Putative uncharacterized protein
2476	P35979	6	108	54.5	165	17805	9.4	60S ribosomal protein L12
2477	P84095	6	24	51.8	191	21308	8.1	Rho-related GTP-binding protein RhoG Rho-related GTP-binding protein RhoG
2478	P01112	6	44	50.8	189	21298	5.3	GTPase HRas GTPase HRas
2478	Q6P716	6	44	39.5	243	26290	7.6	Hras1 protein (Fragment)
2479	UPI0000024CCD	6	124	52.2	115	12895	9.9	PREDICTED: 40S ribosomal protein S20-like, partial
2479	P60866	6	124	50.4	119	13373	9.9	40S ribosomal protein S20 40S ribosomal protein S20
2480	P63173	6	80	50	70	8218	10.1	60S ribosomal protein L38
2480	Q9JJI8	6	80	50	70	8204	10.1	60S ribosomal protein L38
2481	B4E241	6	74	49.2	124	14203	10.1	Splicing factor, arginine/serine-rich 3, isoform CRA_a Splicing factor, arginine/serine-rich 3, isoform CRA_a
2481	Q9D6W4	6	74	37.2	164	19358	11.7	Putative uncharacterized protein
2481	P84103	6	74	37.2	164	19330	11.6	Serine/arginine-rich splicing factor 3 Serine/arginine-rich splicing factor 3
2482	P62266	6	138	49	143	15808	10.5	40S ribosomal protein S23 40S ribosomal protein S23
2482	Q9CWI9	6	138	49	143	15836	10.6	Putative uncharacterized protein
2482	Q497E1	6	138	49	143	15808	10.4	Ribosomal protein S23
2482	Q9CZI5	6	138	48.3	145	16017	10.4	Putative uncharacterized protein
2483	B2R7U5	6	19	47.8	186	21356	7.7	cDNA, FLJ93607
2483	Q96BM9	6	19	47.8	186	21416	7.8	ADP-ribosylation factor-like protein 8A
2483	Q8VEH3	6	19	47.8	186	21390	7.8	ADP-ribosylation factor-like protein 8A
2484	Q9CR41	6	21	45	129	14679	4.9	Huntingtin-interacting protein K
2484	Q9NX55	6	21	45	129	14665	4.9	Huntingtin-interacting protein K
2485	P62854	6	196	44.3	115	13015	11	40S ribosomal protein S26 40S ribosomal protein S26
2485	UPI00006090BB	6	196	44.3	115	13051	10.9	predicted gene 6654

Table S1. Proteins identified in the OVCAR-3 tumor supernatant by two or more unique peptides.

Protein or Protein Group	Accession Number	Unique Peptide Count	Spectrum Count	Sequence Coverage (%)	Length	MolWt	pI	Descriptive Name
2485	Q5JVH5	6	196	44.3	115	13001	11	Ribosomal protein 26 (RPS26) pseudogene
2486	B2R4I8	6	13	43.5	193	21705	5.4	cDNA, FLJ92106, highly similar to sapiens adaptor-related protein complex 3, sigma 1 subunit(AP3S1), mRNA
2486	Q92572	6	13	43.5	193	21732	5.4	AP-3 complex subunit sigma-1 AP-3 complex subunit sigma-1
2487	P62253	6	14	42.9	170	19509	5.3	Ubiquitin-conjugating enzyme E2 G1 Ubiquitin-conjugating enzyme E2 G1
2488	Q08024-4	6	22	53.4	148	17633	6	Isoform 4 of Core-binding factor subunit beta
2488	A8K719	6	22	42.2	187	21919	5.8	cDNA FLJ76413, highly similar to sapiens core-binding factor, beta subunit (CBFB), transcript variant 1, mRNA
2488	Q13951-2	6	22	42.2	187	21991	5.6	Isoform 2 of Core-binding factor subunit beta
2488	Q08024	6	22	42.2	187	22030	5.8	Core-binding factor subunit beta
2489	UPI0000605000	6	24	41.9	124	14335	10.5	PREDICTED: 60S ribosomal protein L31-like isoform 1
2489	B2R4C1	6	24	41.6	125	14495	10.5	cDNA, FLJ92036, highly similar to sapiens ribosomal protein L31 (RPL31), mRNA
2489	Q9CY93	6	24	41.6	125	14411	10.5	Putative uncharacterized protein
2489	P62899	6	24	41.6	125	14463	10.5	60S ribosomal protein L31 60S ribosomal protein L31
2490	E4W6B6	6	55	38.9	126	14257	10.5	RPL27/NME2 fusion protein (Fragment)
2490	B2R4D8	6	55	36	136	15770	10.6	60S ribosomal protein L27
2490	P61353	6	55	36	136	15798	10.6	60S ribosomal protein L27 60S ribosomal protein L27
2491	B3KM57	6	8	31.9	285	32773	4.9	cDNA FLJ10347 fis, clone NT2RM2001035, highly similar to CCR4-NOT transcription complex subunit 7
2491	Q9UIV1	6	8	31.9	285	32745	4.8	CCR4-NOT transcription complex subunit 7
2491	Q60809	6	8	31.9	285	32718	4.8	CCR4-NOT transcription complex subunit 7
2491	Q3V231	6	8	31.9	285	32801	4.8	Putative uncharacterized protein
2492	P63165	6	8	45.5	101	11557	5.5	Small ubiquitin-related modifier 1 Small ubiquitin-related modifier 1
2492	B8ZZN6	6	8	31.5	146	16645	6.2	Uncharacterized protein
2493	P62995-3	6	41	42.6	188	21935	10.2	Isoform 3 of Transformer-2 protein log beta
2493	Q5PR75	6	41	34.9	229	26685	11.3	Sfrs10 protein
2493	Q8N1H4	6	41	31.7	252	29201	10.7	cDNA FLJ40872 fis, clone TUTER2000283, highly similar to sapiens transformer-2-beta (SFRS10) gene
2493	E7EQD1	6	41	31.7	252	29187	10.6	Uncharacterized protein
2493	Q68DZ9	6	41	29	276	32321	11.4	Putative uncharacterized protein DKFZp686F18120 (Fragment)
2493	Q59GA1	6	41	28.8	278	32274	11.1	Splicing factor, arginine/serine-rich 10 (Transformer 2 log, Drosophila) variant (Fragment)
2493	B2RDQ3	6	41	27.8	288	33682	11.2	cDNA, FLJ96718, highly similar to sapiens splicing factor, arginine/serine-rich 10 (transformer 2 log, Drosophila) (SFRS10), mRNA
2493	P62995	6	41	27.8	288	33666	11.2	Transformer-2 protein log beta Transformer-2 protein log beta
2493	E9PD75	6	41	27.7	289	33572	10.9	Uncharacterized protein
2494	A2ADR8	6	8	26.3	350	38400	7.2	Protein phosphatase 1, regulatory (Inhibitor) subunit 8
2494	Q8R3G1	6	8	26.2	351	38528	7.4	Nuclear inhibitor of protein phosphatase 1
2494	Q561W4	6	8	26.2	351	38478	7.4	Protein phosphatase 1, regulatory (Inhibitor) subunit 8
2494	Q12972	6	8	26.2	351	38479	7.4	Nuclear inhibitor of protein phosphatase 1
2495	A7MAV4	6	6	26.2	344	38273	4.9	UBE2Q1 protein (Fragment)
2495	Q7Z7E8	6	6	21.3	422	46127	5.1	Ubiquitin-conjugating enzyme E2 Q1
2495	Q7TSS2	6	6	21.3	422	46173	5.1	Ubiquitin-conjugating enzyme E2 Q1
2496	Q96DH6-2	6	17	31.9	251	28421	8.5	Isoform 2 of RNA-binding protein Musashi log 2
2496	Q920Q6-3	6	17	28.4	282	30893	9.1	Isoform 3 of RNA-binding protein Musashi log 2
2496	Q2M2Q8	6	17	24.4	328	35166	8.5	Msi2h protein
2496	Q96DH6	6	17	24.4	328	35197	8.5	RNA-binding protein Musashi log 2
2496	Q920Q6	6	17	23.1	346	36939	8.5	RNA-binding protein Musashi log 2 RNA-binding protein Musashi log 2
2497	Q3UIS6	6	14	23.2	314	36238	9	Putative uncharacterized protein (Fragment)
2497	A5D8W6	6	14	22.6	323	37261	9.1	Mortality factor 4 like 1
2497	UPI0000194F49	6	14	22.6	323	37261	9.2	PREDICTED: predicted gene 6747 isoform 2
2497	Q569V4	6	14	22.6	323	37231	9.2	MCG125079 MCG125079
2497	Q3UBK1	6	14	22.6	323	37201	9.2	Putative uncharacterized protein
2497	B3KTM8	6	14	21	348	40029	9.1	cDNA FLJ38504 fis, clone HCHON2000156, highly similar to Mortality factor 4-like protein 1
2498	P49615	6	6	20.9	292	33288	7.7	Cyclin-dependent kinase 5
2498	Q00535	6	6	20.9	292	33304	7.7	Cyclin-dependent kinase 5
2499	Q3TV90	6	21	26.1	245	28130	6	Putative uncharacterized protein (Fragment)
2499	Q1W6G4	6	21	19.7	325	38405	10	LUC7-like (S. cerevisiae)
2499	Q9NQ29	6	21	17.3	371	43728	9.9	Putative RNA-binding protein Luc7-like 1
2499	Q9CYI4	6	21	17.3	371	43934	9.9	Putative RNA-binding protein Luc7-like 1
2499	Q53G47	6	21	17.3	371	43712	10	LUC7-like isoform b variant (Fragment)
2500	Q969G3-2	6	22	20.9	363	41780	6	Isoform 2 of SWI/SNF-related matrix-associated actin-dependent regulator of chromatin subfamily E member 1
2500	B4DGM3	6	22	19.3	393	44770	4.8	cDNA FLJ55202, highly similar to SWI/SNF-related matrix-associatedactin-dependent regulator of chromatin subfamily E member 1
2500	Q969G3	6	22	18.5	411	46649	4.9	SWI/SNF-related matrix-associated actin-dependent regulator of chromatin subfamily E member 1
2500	O54941	6	22	18.5	411	46638	4.9	SWI/SNF-related matrix-associated actin-dependent regulator chromatin subfamily E member 1
2501	Q6PKC2	6	13	34.3	236	27016	8.2	CROP protein (Fragment)
2501	Q86Y74	6	13	32.3	251	28926	7	CROP protein (Fragment)
2501	A8K3C5	6	13	18.8	432	51442	9.8	cDNA FLJ77715, highly similar to sapiens cisplatin resistance-associated overexpressed protein(LUC7A), mRNA
2501	Q5SUF2	6	13	18.8	432	51450	9.8	Luc7-like protein 3
2501	O95232	6	13	18.8	432	51466	9.8	Luc7-like protein 3
2501	Q5SUF2-3	6	13	17	477	56779	9.9	Isoform 3 of Luc7-like protein 3
2501	Q5SUF2-2	6	13	16.5	491	58436	9.9	Isoform 2 of Luc7-like protein 3
2502	Q9CWU3	6	10	21.8	367	42606	5.4	MCG141011, isoform CRA_b
2502	Q56VW8	6	10	17.9	447	51648	6.2	Lung cancer-related protein 9
2502	P63151	6	10	17.9	447	51692	6.2	Serine/threonine-protein phosphatase 2A 55 kDa regulatory subunit B alpha isoform Serine/threonine-protein phosphatase 2A 55 kDa regulatory subunit B alpha isoform
2502	B4E1T7	6	10	17.5	457	53000	6.5	cDNA FLJ58665, highly similar to Serine/threonine-protein phosphatase 2A 55 kDa regulatory subunit B alpha isoform
2502	Q571J7	6	10	15.7	509	58394	7.4	MCG141011, isoform CRA_e (Fragment)
2503	Q9D4J7-2	6	34	26.8	284	32283	8.6	Isoform 2 of PHD finger protein 6
2503	B4E0G4	6	34	23	331	37628	8.7	cDNA FLJ60207, highly similar to PHD finger protein 6
2503	Q9D4J7	6	34	20.9	364	41139	8.7	PHD finger protein 6
2503	Q8IWS0	6	34	20.8	365	41290	8.7	PHD finger protein 6
2503	E9PC97	6	34	20.8	366	41362	8.7	Uncharacterized protein
2503	B1B0W7	6	34	16.9	451	51048	8.3	PHD finger protein 6
2504	Q96MN4	6	51	15.5	600	62478	9.4	cDNA FLJ32119 fis, clone PEBLM1000034, highly similar to RNA-BINDING PROTEIN EWS
2504	B0QYK1	6	51	15.5	600	62508	9.4	Ewing sarcoma breakpoint region 1
2504	B0QYK0	6	51	15	618	64929	9.4	Ewing sarcoma breakpoint region 1
2504	Q5SUT0	6	51	15	618	64988	9.4	Ewing sarcoma breakpoint region 1

Table S1. Proteins identified in the OVCAR-3 tumor supernatant by two or more unique peptides.

Protein or Protein Group	Accession Number	Unique Peptide Count	Spectrum Count	Sequence Coverage (%)	Length	MolWt	pI	Descriptive Name
2504	Q9D2P0	6	51	14.2	655	68462	9.3	Ewing sarcoma breakpoint region 1
2504	Q96FE8	6	51	14.2	655	68391	9.3	EWSR1 protein
2504	Q6NVA3	6	51	14.2	656	68549	9.3	Ewing sarcoma breakpoint region 1
2504	Q61545	6	51	14.2	655	68418	9.3	RNA-binding protein EWS
2504	Q3UI30	6	51	14.2	655	68575	9.4	Putative uncharacterized protein
2504	Q01844	6	51	14.2	656	68478	9.3	RNA-binding protein EWS
2504	Q96MX4	6	51	14.1	661	68966	9.3	Ewing sarcoma breakpoint region 1, isoform CRA_h
2504	Q5SUS9	6	51	14.1	661	69037	9.3	Ewing sarcoma breakpoint region 1
2505	B2RAD8	6	6	13.4	857	97244	9.2	cDNA, FLJ94852, highly similar to sapiens eukaryotic translation initiation factor 2C, 1 (EIF2C1), mRNA
2505	Q9UL18	6	6	13.4	857	97214	9.2	Protein argonaute-1 Protein argonaute-1
2505	Q8CJG1	6	6	13.4	857	97101	9.1	Protein argonaute-1
2505	E9Q5W9	6	6	13	884	100343	9.2	Uncharacterized protein
2506	A5Z217	6	81	11.1	470	53596	5.3	Mutant desmin
2506	Q549R9	6	81	11.1	470	53562	5.3	Mutant desmin
2506	Q549R8	6	81	11.1	470	53562	5.3	Mutant desmin
2506	Q45VM8	6	81	11.1	470	53579	5.3	Mutant desmin
2506	Q45VM7	6	81	11.1	470	53584	5.3	Mutant desmin
2506	Q45VM6	6	81	11.1	470	53535	5.4	Mutant desmin
2506	Q3V2C6	6	81	11.1	469	53514	5.3	Putative uncharacterized protein
2506	P31001	6	81	11.1	469	53498	5.3	Desmin
2506	P17661	6	81	11.1	470	53536	5.3	Desmin
2507	Q01433-3	6	8	11.1	760	88198	6.2	Isoform Ex1A-3 of AMP deaminase 2
2507	B4DK50	6	8	11	761	88289	6.2	cDNA FLJ55345, highly similar to AMP deaminase 2 (EC 3.5.4.6)
2507	Q9DBT5	6	8	10.5	798	92024	6.2	AMP deaminase 2
2507	Q01433-2	6	8	10.5	798	92071	6.3	Isoform Ex1A-2-3 of AMP deaminase 2
2507	Q01433-4	6	8	10.4	804	92918	6.4	Isoform Ex1B-3 of AMP deaminase 2
2507	A2AE27	6	8	10.2	824	94696	6.5	Adenosine monophosphate deaminase 2 (Isoform L)
2507	Q01433	6	8	9.6	879	100688	6.9	AMP deaminase 2
2507	B2RB47	6	8	9.6	879	100718	6.9	cDNA, FLJ95309, highly similar to sapiens adenosine monophosphate deaminase 2 (isoform L)(AMPD2), mRNA
2508	E7EVD1	6	16	10.6	634	72076	9.7	Uncharacterized protein
2508	B4DM28	6	16	10.6	634	72104	9.7	cDNA FLJ50575, highly similar to U4/U6 small nuclear ribonucleoprotein Prp3
2508	B2R791	6	16	9.8	683	77543	9.5	cDNA, FLJ93335, highly similar to sapiens PRP3 pre-mRNA processing factor 3 log (yeast) (PRPF3), mRNA
2508	Q922U1	6	16	9.8	683	77455	9.5	U4/U6 small nuclear ribonucleoprotein Prp3
2508	Q53GV6	6	16	9.8	683	77441	9.5	PRP3 pre-mRNA processing factor 3 log (Fragment)
2508	Q3UT19	6	16	9.8	683	77265	9.6	Putative uncharacterized protein
2508	O43395-2	6	16	9.8	682	77472	9.5	Isoform 2 of U4/U6 small nuclear ribonucleoprotein Prp3
2508	O43395	6	16	9.8	683	77529	9.5	U4/U6 small nuclear ribonucleoprotein Prp3
2509	O35218	6	15	9.6	782	88383	5.1	Cleavage and polyadenylation specificity factor subunit 2
2509	Q9P2I0	6	15	9.6	782	88487	5.1	Cleavage and polyadenylation specificity factor subunit 2
2510	B3KY60	6	15	9.1	802	92277	8.2	cDNA FLJ16777 fis, clone BRHIP2029567, highly similar to Cell division cycle 5-like protein
2510	Q99459	6	15	9.1	802	92251	8.2	Cell division cycle 5-like protein
2510	Q6A068	6	15	9.1	802	92190	8	Cell division cycle 5-related protein
2511	Q561M1	6	35	62	158	17922	6.5	Acp1 protein
2511	Q9D358-2	6	35	62	158	17941	7	Isoform 2 of Low molecular weight phosphotyrosine protein phosphatase
2512	P07309	6	165	58.5	147	15776	6.2	Transthyretin
2513	P56380	6	18	54.4	147	16989	6.3	Bis(5'-nucleosyl)-tetraphosphatase [asymmetrical]
2513	Q3V1C8	6	18	54.4	147	17037	6.5	Putative uncharacterized protein
2514	Q923D2	6	17	53.9	206	22197	7	Flavin reductase
2515	Q62048	6	115	53.1	130	15054	5	Astrocytic phosphoprotein PEA-15
2516	Q9R257	6	12	51.6	190	21053	5.3	Heme-binding protein 1
2517	Q9EPB4	6	7	50.8	193	21459	5.4	Apoptosis-associated speck-like protein containing a CARD
2518	Q9JJH1	6	28	50.7	148	17025	8.9	Ribonuclease 4
2519	Q9CQE5	6	16	49.7	181	21151	6.8	Regulator of G-protein signaling 10
2520	P62897	6	23	49.5	105	11605	9.6	Cytochrome c, somatic
2520	UPI0000194F8E	6	23	49.5	105	11695	9.6	UPI0000194F8E UniRef100 entry
2521	Q9D7X3	6	18	47	185	20472	6.5	Dual specificity protein phosphatase 3
2522	UPI000019598D	6	16	46.9	286	32910	5.6	PREDICTED: f-actin-capping protein subunit alpha-1-like
2522	Q5RKN9	6	16	46.9	286	32954	5.6	Capping protein (Actin filament) muscle Z-line, alpha 1
2522	Q3UAS2	6	16	46.9	286	32940	5.6	Putative uncharacterized protein
2522	P47753	6	16	46.9	286	32940	5.6	F-actin-capping protein subunit alpha-1
2522	E9PWZ5	6	16	46.2	290	33328	6.5	Uncharacterized protein
2523	P62869	6	22	45.8	118	13170	5	Transcription elongation factor B polypeptide 2
2523	UPI000019B883	6	22	45.8	118	13167	5	transcription elongation factor B (SIII), polypeptide 2
2524	Q8K4Z3	6	14	45.7	282	30973	7.7	Apolipoprotein A-I-binding protein
2525	UPI000154AC1C	6	37	44.3	115	13029	11	PREDICTED: 40S ribosomal protein S26-like
2526	Q62422	6	18	42.3	215	23783	5.7	Osteoclast-stimulating factor 1
2527	P97823-2	6	13	43	221	23367	6.8	Isoform 2 of Acyl-protein thioesterase 1
2527	P97823	6	13	41.3	230	24688	6.6	Acyl-protein thioesterase 1
2528	Q9CQA1	6	8	39.4	188	20795	9.7	Trafficking protein particle complex subunit 5
2529	Q9CZ82	6	15	39.4	208	23645	6.5	Mediator of RNA polymerase II transcription subunit 18
2530	O55013	6	28	38.3	180	20302	5	Trafficking protein particle complex subunit 3
2531	D3YW48	6	56	38.2	241	25310	5.6	Uncharacterized protein
2531	O88456	6	56	34.2	269	28463	5.6	Calpain small subunit 1
2532	Q9D892	6	25	37.9	198	21897	5.9	Inosine triphosphate pyrophosphatase
2533	Q3U5I9	6	7	37.4	270	30338	6.2	Putative uncharacterized protein
2533	Q9R078	6	7	37.4	270	30308	6.2	5'-AMP-activated protein kinase subunit beta-1
2534	P04938	6	8	44.4	151	17560	4.9	Major urinary proteins 11 and 8 (Fragment)
2534	A2AKN9	6	8	37.2	180	20648	5	Major urinary protein 4
2534	Q58EV3	6	8	37.2	180	20600	5.1	Major urinary protein 1
2534	P11588	6	8	37.2	180	20648	5.1	Major urinary protein 1
2534	P02762	6	8	37.2	180	20649	4.9	Major urinary protein 6
2534	A9R9W0	6	8	37.2	180	20647	5.2	Novel member of the major urinary protein (Mup) gene family
2534	A2CEL1	6	8	37.2	180	20634	5.1	Major urinary protein 1
2534	A2BIM8	6	8	37	181	20763	4.9	Major urinary protein 16
2535	Q8BGB7	6	16	37	257	28600	4.9	Enolase-phosphatase E1
2536	E9QN99	6	8	36.7	210	22451	6.5	Uncharacterized protein
2536	Q8VCR7	6	8	36.7	210	22451	6.3	Abhydrolase domain-containing protein 14B
2537	P08074	6	11	36.1	244	25958	9	Carbonyl reductase [NADPH] 2
2538	B7ZNS9	6	22	34.9	258	27986	6.7	Cfd protein
2538	P03953	6	22	34.7	259	28057	6.7	Complement factor D
2539	O09164	6	12	34.3	251	27392	6.8	Extracellular superoxide dismutase [Cu-Zn]
2539	O88592	6	12	34.3	251	27393	6.7	Superoxide dismutase [Cu-Zn]
2540	Q3TAR7	6	9	34.2	219	23398	5.1	Proteasome subunit beta type
2541	P98086	6	13	33.1	245	25986	9.1	Complement C1q subcomponent subunit A
2541	Q9DCM6	6	13	33.1	245	25974	9.1	Complement component 1, q subcomponent, alpha polypeptide
2541	Q3UBX7	6	13	33.1	245	26046	8.9	Putative uncharacterized protein
2541	Q3TXB1	6	13	33.1	245	25975	8.9	Putative uncharacterized protein

Table S1. Proteins identified in the OVCAR-3 tumor supernatant by two or more unique peptides.

Protein or Protein Group	Accession Number	Unique Peptide Count	Spectrum Count	Sequence Coverage (%)	Length	MolWt	pI	Descriptive Name
2542	Q811C8	6	8	33.5	263	27158	8.5	Igfbp7 protein (Fragment)
2542	A0A5E1	6	8	31.3	281	28898	8.2	Insulin-like growth factor binding protein 7
2542	Q61581	6	8	31.3	281	28969	8.3	Insulin-like growth factor-binding protein 7
2542	Q3UFA6	6	8	31.2	282	29056	8.4	Putative uncharacterized protein
2543	Q99LC8	6	16	30.8	305	33816	8.3	Translation initiation factor eIF-2B subunit alpha
2544	Q3TS19	6	6	30.7	261	28331	7	Car1 protein
2545	O70370	6	10	30.6	340	38438	7	Cathepsin S
2545	Q8BSZ5	6	10	30.4	342	38707	7	Cathepsin S
2545	Q3UD32	6	10	30.3	343	38863	7.2	Putative uncharacterized protein
2546	A6H6N4	6	13	26	289	31795	6.2	Four and a half LIM domains 3
2547	Q4VAA2	6	19	25.3	281	29729	6.1	Protein CDV3
2548	Q9EST5	6	10	28.3	272	31079	4	Acidic leucine-rich nuclear phosphoprotein 32 family member B
2548	E9QKP8	6	10	24.4	315	35796	4.2	Uncharacterized protein
2548	Q9EST5-2	6	10	23.4	329	37447	4.2	Isoform 2 of Acidic leucine-rich nuclear phosphoprotein 32 family member B
2549	P26350	6	17	23.4	111	12254	3.8	Prothymosin alpha
2549	Q3U6E4	6	17	23.4	111	12325	3.8	Putative uncharacterized protein
2549	Q3UA54	6	17	15.1	172	18688	4.1	Putative uncharacterized protein (Fragment)
2550	P47877	6	34	23	305	32847	7.6	Insulin-like growth factor-binding protein 2
2551	Q80VJ2	6	16	22.3	220	24325	6.6	Steroid receptor RNA activator 1
2552	Q3U3I6	6	7	21.6	473	52208	7	Putative uncharacterized protein
2552	Q8CAJ7	6	7	21.6	473	52207	7.4	Putative uncharacterized protein
2552	Q56A00	6	7	21.6	473	52207	7.3	Aspartyl aminopeptidase, isoform CRA_a
2553	Q3U646	6	13	20.8	394	44598	8.6	Putative uncharacterized protein
2553	Q9WV54	6	13	20.8	394	44670	8.5	Acid ceramidase
2553	Q3U8A7	6	13	20.8	394	44671	8.3	Putative uncharacterized protein
2554	Q3UUU4	6	11	20.8	427	48787	6.6	Putative uncharacterized protein
2554	Q9R1T4-3	6	11	20.7	429	49058	6.6	Isoform V of Septin-6
2554	Q9R1T4	6	11	20.5	434	49620	6.4	Septin-6
2555	UPI00001E53C2	6	57	24.8	343	38502	9.2	lamina-associated polypeptide 2 isoform beta
2555	Q61029-4	6	57	24.8	343	38423	9.1	Isoform Gamma of Lamina-associated polypeptide 2, isoforms beta/delta/epsilon/gamma
2555	UPI00004347B2	6	57	22.4	380	42650	9.4	lamina-associated polypeptide 2 isoform gamma
2555	Q61029-2	6	57	22.4	380	42572	9.4	Isoform Delta of Lamina-associated polypeptide 2, isoforms beta/delta/epsilon/gamma
2555	Q3TNH0	6	57	20.6	412	46050	9.2	Putative uncharacterized protein
2555	Q61029-3	6	57	20.6	412	45972	9.1	Isoform Epsilon of Lamina-associated polypeptide 2, isoforms beta/delta/epsilon/gamma
2555	Q61029	6	57	18.8	452	50295	9.4	Lamina-associated polypeptide 2, isoforms beta/delta/epsilon/gamma
2555	Q3UR33	6	57	18.8	452	50359	9.4	Putative uncharacterized protein
2555	Q3UCI5	6	57	18.8	452	50373	9.4	Putative uncharacterized protein
2555	Q3U955	6	57	18.8	452	50471	9.5	Putative uncharacterized protein
2555	Q3U7B3	6	57	18.8	452	50502	9.4	Putative uncharacterized protein
2556	Q3UKE5	6	10	20.2	322	36544	8.7	Lectin, galactose binding, soluble 9
2557	Q3TXN1	6	11	19	315	34268	5.5	Putative uncharacterized protein
2557	Q8VDQ1-2	6	11	19	316	34449	5.5	Isoform 2 of Prostaglandin reductase 2
2557	Q8VDQ1	6	11	17.1	351	38015	5.4	Prostaglandin reductase 2
2558	P11680	6	10	18.8	464	50327	7.8	Properdin
2559	Q9DBF1	6	13	18.2	539	58862	7.5	Alpha-aminoadipic semialdehyde dehydrogenase
2560	Q8R121-2	6	13	20.3	394	45298	5.2	Isoform 2 of Protein Z-dependent protease inhibitor
2560	Q8R121	6	13	17.9	448	51797	5.7	Protein Z-dependent protease inhibitor
2561	Q921S3	6	9	17.3	572	63998	7.3	Malic enzyme
2561	Q9DBF9	6	9	17.3	572	63954	7.4	Malic enzyme
2562	Q78ZA7	6	18	17.6	375	42679	4.7	Nucleosome assembly protein 1-like 4
2562	B7ZNL2	6	18	17.1	386	43935	4.7	Nap1l4 protein
2562	E9Q6R0	6	18	15.7	421	47275	4.7	Uncharacterized protein
2562	Q8C1W9	6	18	15.6	422	47362	4.7	Putative uncharacterized protein
2563	UPI0001E6B84E	6	40	16.7	443	50001	5	PREDICTED: tubulin beta-3 chain-like
2564	P23591	6	15	16.5	321	35878	6.7	GDP-L-fucose synthase
2565	P29788	6	15	15.7	478	54849	5.9	Vitronectin
2566	Q8K0J4	6	13	18.9	387	43335	5.3	Epidermal growth factor-containing fibulin-like extracellular matrix protein 1
2566	Q8BPB5	6	13	14.8	493	54953	5.1	EGF-containing fibulin-like extracellular matrix protein 1
2567	Q3UJS6	6	12	14.1	533	59615	5.9	Putative uncharacterized protein
2567	Q8BT60	6	12	14.1	533	59585	5.8	Copine-3
2568	P48025	6	7	13.8	629	71376	7.9	Tyrosine-protein kinase SYK
2568	Q6P1E0	6	7	13.8	629	71342	7.9	Syk protein
2569	Q80YC5	6	25	13.7	597	65701	6.8	Coagulation factor XII
2570	Q9JKV1	6	17	13.3	407	42060	5.1	Proteasomal ubiquitin receptor ADRM1
2571	E9QLP8	6	63	13.2	461	49404	5	Uncharacterized protein
2572	Q8BND5-2	6	19	12.3	661	73297	6.9	Isoform 2 of Sulfhydryl oxidase 1
2572	Q8BND5	6	19	10.8	748	82785	7.2	Sulfhydryl oxidase 1
2573	Q9Z1T2	6	7	9.4	963	106366	4.7	Thrombospondin-4
2574	P46935	6	10	9	887	102706	5.3	E3 ubiquitin-protein ligase NEDD4
2575	Q8VI75	6	20	7.9	1082	119275	5	Importin-4
2576	A6H8H2	6	14	4.1	1906	211456	6.4	Dennd4c protein
2576	A2AJX5	6	14	4	1955	216453	6.2	DENN/MADD domain containing 4C
2577	Q91ZU6-2	6	8	3.1	5380	614934	5.7	Isoform 1 of Dystonin
2577	E9QL23	6	8	3.1	5379	615221	5.7	Uncharacterized protein
2577	E9PXE4	6	8	2.2	7359	829857	5.3	Uncharacterized protein
2577	Q91ZU6-4	6	8	2.2	7402	835096	5.3	Isoform 4 of Dystonin
2577	Q91ZU6	6	8	2.2	7389	833654	5.3	Dystonin
2577	E9PXE5	6	8	2.2	7393	834234	5.3	Uncharacterized protein
2578	P0CG13	6	13	81	121	13314	8.2	Chromosome transmission fidelity protein 8 log
2578	UPI0001E8F538	6	13	68.1	144	15356	9.1	UPI0001E8F538 UniRef100 entry
2579	P35244	6	29	80.2	121	13569	5.1	Replication protein A 14 kDa subunit
2580	P52758	6	34	73	137	14494	8.7	Ribonuclease UK114
2581	Q9UI30	6	51	72	125	14199	5.3	tRNA methyltransferase 112 log
2582	Q9UK76	6	40	70.8	154	16015	5.6	Hematological and neurological expressed 1 protein
2583	Q9UK45	6	22	65	103	11602	5.3	U6 snRNA-associated Sm-like protein LSm7
2584	Q96FQ6	6	107	64.1	103	11801	6.8	Protein S100-A16
2585	P47929	6	32	60.3	136	15075	7.6	Galectin-7
2586	P50238	6	34	59.7	77	8533	8.8	Cysteine-rich protein 1
2587	Q9BYN0	6	12	56.9	137	14259	8.2	Sulfiredoxin-1
2588	P99999	6	34	55.2	105	11749	9.6	Cytochrome c
2589	O95994	6	17	54.9	175	19979	9	Anterior gradient protein 2 log
2589	Q53G64	6	17	54.9	175	19963	9	Anterior gradient 2 log (Fragment)
2590	P09132	6	28	54.9	144	16156	9.8	Signal recognition particle 19 kDa protein
2591	Q9NX24	6	13	54.2	153	17201	8.2	H/ACA ribonucleoprotein complex subunit 2
2592	A8MQB6	6	17	52.5	183	21124	5.3	Uncharacterized protein
2592	Q9NX08	6	17	52.5	183	21090	5.4	COMM domain-containing protein 8
2593	Q13526	6	46	51.5	163	18243	8.8	Peptidyl-prolyl cis-trans isomerase NIMA-interacting 1
2594	O75348	6	24	50.8	118	13758	8.8	V-type proton ATPase subunit G 1

Table S1. Proteins identified in the OVCAR-3 tumor supernatant by two or more unique peptides.

Protein or Protein Group	Accession Number	Unique Peptide Count	Spectrum Count	Sequence Coverage (%)	Length	MolWt	pI	Descriptive Name
2595	P02144	6	8	50.6	154	17184	7.7	Myoglobin
2596	Q04837	6	14	50	148	17260	9.6	Single-stranded DNA-binding protein, mitochondrial
2596	Q567R6	6	14	50	148	17359	9.7	Single-stranded DNA binding protein 1
2597	P63302	6	9	49.4	87	9298	9.2	Selenoprotein W
2597	UPI000179AE55	6	9	48.3	89	9628	9.2	Selenoprotein W.
2598	Q9UHV9	6	56	48.7	154	16648	6.6	Prefoldin subunit 2
2599	A0PJ62	6	59	48.4	124	14611	10.6	RPL14 protein (Fragment)
2599	E7EPB3	6	59	48.4	124	14558	10.2	Uncharacterized protein
2599	B7Z6S8	6	59	46.9	128	15083	10.7	cDNA FLJ51325, highly similar to 60S ribosomal protein L14
2599	P50914	6	59	27.9	215	23432	10.9	60S ribosomal protein L14
2599	A8K7N0	6	59	27.5	218	23646	10.8	cDNA FLJ75556, highly similar to sapiens ribosomal protein L14, mRNA
2599	Q6IPH7	6	59	27.3	220	23787	10.9	RPL14 protein
2600	Q9NP97	6	30	74	96	10922	7.2	Dynein light chain roadblock-type 1
2600	B1AKR6	6	30	48	148	16253	7	Dynein, light chain, roadblock-type 1
2601	E9PRR8	6	8	47.9	146	16748	6.1	Uncharacterized protein
2601	Q8N9N8	6	8	42.4	165	19053	5.2	Probable RNA-binding protein EIF1AD
2602	Q4LDG9	6	8	47.4	190	21533	6.1	Dynein light chain 1, axonemal
2603	O96007	6	14	47.3	188	20944	5.4	Molybdopterin synthase catalytic subunit
2604	Q6FHL9	6	125	53.1	130	15068	5	PEA15 protein
2604	Q15121	6	125	53.1	130	15040	5	Astrocytic phosphoprotein PEA-15
2604	B1AKZ3	6	125	45.7	151	17307	4.9	Phosphoprotein enriched in astrocytes 15
2605	P07108	6	28	50.6	87	10044	6.5	Acyl-CoA-binding protein
2605	P07108-3	6	28	50	88	10145	6.5	Isoform 3 of Acyl-CoA-binding protein
2605	B8ZWD1	6	28	45.4	97	11150	8.3	Diazepam binding inhibitor, splice form 1A(2)
2605	B8ZWD2	6	28	42.3	104	11793	5.5	Diazepam binding inhibitor (GABA receptor modulator, acyl-Coenzyme A binding protein), isoform CRA_a
2605	B8ZWD7	6	28	34.1	129	14367	9.4	Diazepam binding inhibitor, splice form 1A(1)-G
2605	B8ZWD9	6	28	30.6	144	15959	5	Diazepam binding inhibitor, splice form 1D(2)
2605	B8ZWD6	6	28	29.7	148	16495	9.4	Diazepam binding inhibitor, splice form 1G
2606	Q9H7C9	6	32	52.5	122	13332	8.4	UPF0366 protein C11orf67
2606	E9PNP3	6	32	45.4	141	15588	9.1	Uncharacterized protein
2607	Q9Y4Z0	6	44	45.3	139	15350	10	U6 snRNA-associated Sm-like protein LSm4
2608	Q53FT3	6	13	45.2	197	21628	5.5	Uncharacterized protein C11orf73
2609	A8K651	6	7	45	282	31380	4.8	cDNA FLJ75700, highly similar to sapiens complement component 1, q subcomponent binding protein (C1QBP), nuclear gene encoding mitochondrial protein, mRNA
2609	Q07021	6	7	45	282	31362	4.8	Complement component 1 Q subcomponent-binding protein, mitochondrial
2610	P36404	6	11	44.6	184	20878	6.4	ADP-ribosylation factor-like protein 2
2611	B2R4D5	6	45	44.4	178	20521	8.6	cDNA, FLJ92050, highly similar to sapiens actin related protein 2/3 complex, subunit 3, 21kDa (ARPC3), mRNA
2611	UPI00015DFF36	6	45	44.4	178	20529	8.6	Actin-related protein 2/3 complex subunit 3 (ARP2/3 complex 21 kDa subunit) (p21-ARC).
2611	O15145	6	45	44.4	178	20547	8.6	Actin-related protein 2/3 complex subunit 3
2612	Q9Y3B2	6	8	43.1	195	21452	8.2	3'-5' exoribonuclease CSL4 log
2613	A8K005	6	14	42.4	184	20465	5.9	cDNA FLJ77896, highly similar to sapiens Ras log enriched in brain (RHEB), mRNA
2613	Q6NZ61	6	14	42.4	184	20558	5.9	Ras log enriched in brain
2613	Q15382	6	14	42.4	184	20497	5.9	GTP-binding protein Rheb
2614	Q9NV35	6	12	42.1	164	18609	6.1	Probable 7,8-dihydro-8-oxoguanine triphosphatase NUDT15
2615	P05109	6	17	41.9	93	10834	7	Protein S100-A8
2616	P41208	6	15	41.9	172	19738	5	Centrin-2
2617	Q9P0P0	6	11	41.8	153	17909	5	E3 ubiquitin-protein ligase RNF181
2618	Q9H944	6	12	41.5	212	23222	6.9	Mediator of RNA polymerase II transcription subunit 20
2619	Q8N2Z9	6	6	41.3	138	15893	6	Centromere protein S
2620	Q8WTX7	6	6	41	329	36275	5.2	GATS-like protein 3
2621	Q9NQT4	6	8	40.9	235	25249	7.6	Exosome complex exonuclease RRP46
2622	P10301	6	13	40.4	218	23480	6.9	Ras-related protein R-Ras
2623	Q4R379	6	17	44.2	206	23379	6.6	Ras-related protein Ral-B
2623	P11234	6	17	44.2	206	23409	6.6	Ras-related protein Ral-B
2623	B4E040	6	17	39.9	228	25966	7.8	cDNA FLJ55177, highly similar to Ras-related protein Ral-B
2624	P49914	6	10	39.9	203	23256	7.9	5-formyltetrahydrofolate cyclo-ligase
2625	Q9BRX5	6	8	39.8	216	24535	5.3	DNA replication complex GINS protein PSF3
2626	B2RA97	6	8	39.7	214	22957	5.1	HemK methyltransferase family member 2, isoform CRA_a
2627	Q9H910	6	13	45.3	190	20063	9.3	Hematological and neurological expressed 1-like protein
2627	B4DLH4	6	13	39.4	218	23026	9.6	cDNA FLJ53622, highly similar to Hematological and neurological expressed1-like protein
2628	Q9NQT5	6	7	39.3	275	29572	8.1	Exosome complex exonuclease RRP40
2629	B3KPX6	6	7	39	231	25949	6	cDNA FLJ32419 fis, clone SKMUS2000894, highly similar to Ribosyldihydronicotinamide dehydrogenase
2629	P16083	6	7	39	231	25919	6.3	Ribosyldihydronicotinamide dehydrogenase [quinone]
2630	Q9NQP4	6	30	38.8	134	15314	4.5	Prefoldin subunit 4
2631	O75935-3	6	12	45.6	158	18001	6	Isoform 3 of Dynactin subunit 3
2631	O75935	6	12	38.7	186	21119	5.5	Dynactin subunit 3
2632	B2RDN1	6	18	38.4	216	24126	7.7	cDNA, FLJ96690, highly similar to sapiens glutathione transferase zeta 1 (maleylacetoacetate isomerase) (GSTZ1), transcript variant 1, mRNA
2633	O43617	6	30	38.3	180	20274	5	Trafficking protein particle complex subunit 3
2634	Q9BV86	6	7	38.1	223	25387	5.5	Alpha N-terminal protein methyltransferase 1A
2635	P51153	6	13	37.9	203	22774	9.2	Ras-related protein Rab-13
2635	Q504R6	6	13	31.6	244	27199	8.9	RAB13 protein (Fragment)
2636	Q5TZN6	6	10	48.6	208	22629	4.2	Nucleolar protein 3 (Apoptosis repressor with CARD domain)
2636	B4DFL0	6	10	37.4	270	29355	4.3	Nucleolar protein 3 (Apoptosis repressor with CARD domain), isoform CRA_a
2637	Q969H8	6	25	37	173	18795	6.7	UPF0556 protein C19orf10
2638	E9PH29	6	15	36.1	238	25839	7.5	Uncharacterized protein
2638	P30048	6	15	33.6	256	27693	7.8	Thioredoxin-dependent peroxide reductase, mitochondrial
2639	O95716	6	10	36.1	219	24267	4.9	Ras-related protein Rab-3D
2640	Q14257	6	7	35.6	317	36876	4.4	Reticulocalbin-2
2640	UPI000041D59E	6	7	33.7	335	39139	4.5	Reticulocalbin-2 precursor (Calcium-binding protein ERC-55) (E6-binding protein) (E6BP).
2641	B4E0H1	6	18	35	366	42112	8.6	cDNA FLJ50431, highly similar to Discs large log 3
2641	Q5JUW6	6	18	25	512	57937	9.2	Discs, large log 3 (Neuroendocrine-dlg, Drosophila)
2642	Q7Z7N6	6	23	44.2	156	17951	5.1	NUDT1 protein
2642	P36639	6	23	35	197	22520	5.3	7,8-dihydro-8-oxoguanine triphosphatase
2643	A8K4Z2	6	7	34.9	255	28786	6.5	cDNA FLJ77120, highly similar to sapiens replication factor C (activator 1) 5, 36.5kDa (RFC5), transcript variant 2, mRNA
2643	E9PBA2	6	7	34.9	255	28846	6.5	Uncharacterized protein
2643	A8MZ62	6	7	27.9	319	36105	6.9	Uncharacterized protein
2643	Q6LES9	6	7	26.2	340	38397	6.9	RFC5 protein (Fragment)
2643	P40937	6	7	26.2	340	38497	7.2	Replication factor C subunit 5
2643	Q59GW7	6	7	25.4	351	39643	7.8	Replication factor C 5 isoform 1 variant (Fragment)

Table S1. Proteins identified in the OVCAR-3 tumor supernatant by two or more unique peptides.

Protein or Protein Group	Accession Number	Unique Peptide Count	Spectrum Count	Sequence Coverage (%)	Length	MolWt	pI	Descriptive Name
2644	Q9NVT9	6	10	34.8	282	31281	5.8	Armadillo repeat-containing protein 1
2645	Q969J3	6	6	34.7	196	22222	6.4	Loss of heterozygosity 12 chromosomal region 1 protein
2646	Q9NWS0	6	6	34.5	290	32363	5.1	PIH1 domain-containing protein 1
2647	O60344-4	6	6	34.1	255	28306	6.1	Isoform D of Endothelin-converting enzyme 2
2648	Q96S19	6	9	33.8	204	22578	7.8	UPF0585 protein C16orf13
2649	Q15628	6	7	33.7	312	34247	6.3	Tumor necrosis factor receptor type 1-associated DEATH domain protein
2650	O75391	6	6	33.5	227	26034	7.9	Sperm-associated antigen 7
2651	E9PNY1	6	19	33.3	207	22686	6.5	Uncharacterized protein
2651	O95159	6	19	22.3	310	34114	8.1	Zinc finger protein-like 1
2652	P35270	6	16	33.3	261	28048	8	Sepiapterin reductase
2653	A8K5R6	6	23	32.8	250	28609	9.4	cDNA FLJ77138, highly similar to sapiens golgi SNAP receptor complex member 1 (GOSR1), transcript variant 1, mRNA
2653	O95249	6	23	32.8	250	28613	9.4	Golgi SNAP receptor complex member 1
2654	Q13442	6	36	32.6	181	20630	8.9	28 kDa heat- and acid-stable phosphoprotein
2655	Q9NPD8	6	7	32.5	197	22521	8	Ubiquitin-conjugating enzyme E2 T
2656	C9JXB9	6	6	32	244	26884	5.1	Uncharacterized protein
2656	P25686	6	6	24.1	324	35580	6	DnaJ log subfamily B member 2
2657	B7Z472	6	9	31.7	309	35377	5.9	cDNA FLJ51987, highly similar to sapiens queuine tRNA-ribosyltransferase domain containing 1 (QTRTD1), mRNA
2657	Q9H974	6	9	23.6	415	46713	6.8	Queuine tRNA-ribosyltransferase subunit QTRTD1
2658	O14732-2	6	19	34.5	261	28512	7	Isoform 2 of Inositol monophosphatase 2
2658	O14732	6	19	31.2	288	31321	6.6	Inositol monophosphatase 2
2659	Q9BV20-2	6	7	35.7	322	34529	5.7	Isoform 2 of Methylthioribose-1-phosphate isomerase
2659	Q9BV20	6	7	31.2	369	39150	6.3	Methylthioribose-1-phosphate isomerase
2660	Q9BY43	6	6	31.1	222	25098	4.7	Charged multivesicular body protein 4a
2661	Q5JTE4	6	8	34.1	314	35838	5.7	Polymerase (RNA) I polypeptide C, 30kDa
2661	O15160-2	6	8	31.3	342	38647	6	Isoform 2 of DNA-directed RNA polymerases I and III subunit RPAC1
2661	O15160	6	8	30.9	346	39250	5.5	DNA-directed RNA polymerases I and III subunit RPAC1
2662	Q8N4P3	6	7	30.2	179	20329	6.7	Guanosine-3',5'-bis(diphosphate) 3'-pyrophosphohydrolase MESH1
2663	Q96CT7	6	14	30	223	25835	9.5	Coiled-coil domain-containing protein 124
2664	P29966	6	35	29.5	332	31555	4.4	Myristoylated alanine-rich C-kinase substrate
2665	P22352	6	37	29.2	226	25402	8.1	Glutathione peroxidase 3
2666	O75569-2	6	9	30.1	302	33123	8.4	Isoform 2 of Interferon-inducible double stranded RNA-dependent protein kinase activator A
2666	O75569	6	9	29.1	313	34404	8.4	Interferon-inducible double stranded RNA-dependent protein kinase activator A
2667	Q5QPP2	6	8	50.8	195	21490	6.8	UDP-galactose-4-epimerase (Fragment)
2667	Q14376	6	8	28.4	348	38282	6.7	UDP-glucose 4-epimerase
2668	P14649	6	21	30.3	208	22764	5.7	Myosin light chain 6B
2668	B4E368	6	21	28.3	223	24541	9.7	cDNA FLJ60058, highly similar to Myosin light chain 1, slow-twitch muscle A isoform
2669	B2RBQ4	6	7	28.2	333	37151	6.1	cDNA, FLJ95632, sapiens ankyrin repeat domain 2 (stretch responsive muscle)(ANKRD2), mRNA
2669	Q9GZV1	6	7	26.1	360	39859	6.1	Ankyrin repeat domain-containing protein 2
2670	Q92522	6	37	28.2	213	22487	10.8	Histone H1x
2671	Q9NRN9	6	10	28.2	209	23719	6.7	Methyltransferase-like protein 5
2672	B2RD36	6	13	27.8	245	28058	6.8	cDNA, FLJ96437
2672	Q96DG6	6	13	27.8	245	28048	7.2	Carboxymethylenebutenolidase log
2673	Q9BUT1	6	8	27.8	245	26724	7.6	3-hydroxybutyrate dehydrogenase type 2
2674	Q7LG56	6	8	27.1	351	40737	5	Ribonucleoside-diphosphate reductase subunit M2 B
2674	UPI0001E8F504	6	8	23.3	408	47080	7.5	UPI0001E8F504 UniRef100 entry
2674	UPI0001C4CFFD	6	8	22.5	423	48787	8	ribonucleoside-diphosphate reductase subunit M2 B isoform 2
2675	Q8NG26	6	9	26.7	300	33746	6.8	Putative nudix hydrolyase
2675	Q9BW91-2	6	9	26.7	300	33776	6.8	Isoform 2 of ADP-ribose pyrophosphatase, mitochondrial
2675	Q9BW91	6	9	22.9	350	39125	8.2	ADP-ribose pyrophosphatase, mitochondrial
2675	Q96KB3	6	9	22.9	350	39042	8.1	cDNA FLJ14389 fis, clone HEMBA1002876
2676	Q96QR8	6	15	26.6	312	33241	5.4	Transcriptional activator protein Pur-beta
2677	A8K9K8	6	7	26.5	313	35237	9.9	cDNA FLJ75823, highly similar to sapiens dimethyladenosine transferase (HSA9761), mRNA
2677	Q9UNQ2	6	7	26.5	313	35236	10	Probable dimethyladenosine transferase
2678	Q96GD0	6	8	26.4	296	31698	6.5	Pyridoxal phosphate phosphatase
2679	Q9BQA1	6	12	26.3	342	36724	5.2	Methylosome protein 50
2680	O14530	6	9	26.1	226	26534	5.9	Thioredoxin domain-containing protein 9
2681	Q96PJ7	6	13	30.8	211	23758	8.7	Ras-related GTP-binding protein RAB39
2681	C9JBG0	6	13	30.8	211	23897	7.7	Uncharacterized protein
2681	UPI0000E59ED8	6	13	28.1	231	25770	9	UPI0000E59ED8 UniRef100 entry
2681	E7ES60	6	13	27.8	234	26144	9.2	Uncharacterized protein
2681	Q9BZG1	6	13	25.1	259	29044	7.9	Ras-related protein Rab-34
2681	Q6NXT3	6	13	25.1	259	29071	7.9	RAB34 protein
2681	A8MYQ9	6	13	25	260	29201	8.1	Uncharacterized protein
2681	C9J3R5	6	13	24.3	268	29868	9.3	Uncharacterized protein
2681	UPI0001929526	6	13	20.6	316	35185	8.6	ras-related protein Rab-34 isoform 5
2682	Q96JY6	6	19	25	352	37459	8.7	PDZ and LIM domain protein 2
2682	UPI0000E5AE4E	6	19	14.6	602	62746	10.9	PDZ and LIM domain protein 2 isoform 2
2683	C9J8Z4	6	13	24.6	301	32285	6.8	Uncharacterized protein
2683	Q969P0-3	6	13	14.1	526	55808	8.8	Isoform 3 of Immunoglobulin superfamily member 8
2683	Q969P0	6	13	12.1	613	65034	8	Immunoglobulin superfamily member 8
2684	O14562	6	13	24.6	309	33382	5.8	Ubiquitin domain-containing protein UBFD1
2685	Q5SR21	6	10	32.1	274	31862	8.6	DNAation factor, 40kDa, beta polypeptide (Caspase-activated DNase)
2685	O76075	6	10	26	338	39110	9	DNA fragmentation factor subunit beta
2685	B4DZS0	6	10	24.3	362	41956	8.8	cDNA FLJ50316, highly similar to DNAation factor subunit beta (EC 3.-.-.-)
2686	Q9Y312	6	7	25	384	43472	6	Uncharacterized protein C20orf4
2686	A2A2Q9	6	7	24.1	398	45034	7.5	Chromosome 20 open reading frame 4
2687	O43633	6	8	23.9	222	25104	6	Charged multivesicular body protein 2a
2688	Q9H7Z7	6	8	23.9	377	41943	9.2	Prostaglandin E synthase 2
2689	Q9BWH2	6	6	23.8	189	20676	9.7	FUN14 domain-containing protein 2
2690	A8K6X2	6	11	23.5	357	39477	6.5	cDNA FLJ77992, highly similar to sapiens ADP-ribosylarginine hydrolase, mRNA
2690	P54922	6	11	23.5	357	39507	6.5	[Protein ADP-ribosylarginine] hydrolase
2691	Q7L5D6-2	6	13	28.1	274	30523	5	Isoform 2 of Golgi to ER traffic protein 4 log
2691	Q7L5D6	6	13	23.5	327	36504	5.4	Golgi to ER traffic protein 4 log
2692	Q9Y5B8	6	7	23.4	376	42492	6.5	Nucleoside diphosphate kinase 7
2693	B0FTY2	6	17	23	361	40779	5.2	NudC-like protein
2693	Q8IVD9	6	17	23	361	40822	5.3	NudC domain-containing protein 3
2694	Q8IUF8	6	6	22.4	465	52800	6.7	MYC-induced nuclear antigen
2694	Q8IUF8-4	6	6	22.4	464	52672	6.7	Isoform 4 of MYC-induced nuclear antigen
2695	B3KSH8	6	7	21.1	399	43982	5.3	cDNA FLJ36241 fis, clone THYMU2001622, highly similar to Inositol polyphosphate 1-phosphatase (EC 3.1.3.57)

Table S1. Proteins identified in the OVCAR-3 tumor supernatant by two or more unique peptides.

Protein or Protein Group	Accession Number	Unique Peptide Count	Spectrum Count	Sequence Coverage (%)	Length	MolWt	pI	Descriptive Name
2695	P49441	6	7	21.1	399	43998	5.3	Inositol polyphosphate 1-phosphatase
2695	B5BU62	6	7	21.1	399	43966	5.3	Inositol polyphosphate-1-phosphatase
2696	Q9BWS9	6	11	24.2	393	44941	8.6	Chitinase domain-containing protein 1
2696	B7Z705	6	11	22.5	423	48228	7.7	cDNA FLJ56160
2696	B4DN31	6	11	20.9	454	51051	8.9	cDNA FLJ55809
2696	UPI0001AE6B5B	6	11	20.9	454	51039	8.9	Chitinase domain-containing protein 1 precursor (Stabilin-1-interacting chitinase-like protein) (SI-CLP).
2697	B3KUU4	6	7	20.6	446	49718	6.5	NAD synthetase 1, isoform CRA_a
2697	Q6IA69	6	7	13	706	79285	6.4	Glutamine-dependent NAD(+) synthetase
2698	Q53F51	6	7	21	357	41238	6.9	FGF intracellular binding protein isoform b variant (Fragment)
2698	O46431	6	7	21	357	41210	6.8	Acidic fibroblast growth factor intracellular-binding protein
2698	O43427	6	7	20.6	364	41878	6.5	Acidic fibroblast growth factor intracellular-binding protein
2699	E1Y6P6	6	12	27.8	270	31111	5.3	MHC class I antigen (Fragment)
2699	E3Q1I0	6	12	21.6	347	39054	5.9	MHC class I antigen (Fragment)
2699	A4Q9R6	6	12	20.5	366	40676	6	MHC class I antigen
2699	A6H578	6	12	20.5	365	40561	6	MHC class I antigen (Fragment)
2700	UPI0001E6F9FC	6	32	40.7	182	21608	7.6	PREDICTED: 3-hydroxyacyl-CoA dehydratase 3-like isoform 1
2700	Q9P035	6	32	20.4	362	43160	8.9	3-hydroxyacyl-CoA dehydratase 3
2701	P82970	6	24	20.2	282	31525	4.6	High mobility group nucleosome-binding domain-containing protein 5
2702	B8ZZQ6	6	46	24.3	107	11759	3.8	Uncharacterized protein
2702	Q15202	6	46	23.9	109	11959	3.7	Prothymosin alpha
2702	Q8TBK9	6	46	23.6	110	12016	3.8	Prothymosin, alpha
2702	Q86YS2	6	46	23.6	110	12044	3.8	Prothymosin alpha protein
2702	Q5VYG3	6	46	23.6	110	12055	3.8	Novel protein similar to prothymosin, alpha (PTMA)
2702	Q53S24	6	46	23.6	110	12074	3.8	Prothymosin, alpha
2702	Q15204	6	46	23.6	110	12047	3.7	Prothymosin alpha
2702	Q15200	6	46	23.6	110	12085	3.8	Prothymosin alpha
2702	P06454	6	46	23.4	111	12203	3.8	Prothymosin alpha
2702	B8ZZA1	6	46	19.8	131	14252	3.8	Uncharacterized protein
2702	B8ZZW7	6	46	19.1	136	14719	4.1	Uncharacterized protein
2702	UPI00018819F6	6	46	17.6	148	15860	4.2	UPI00018819F6 UniRef100 entry
2703	B2R6S4	6	9	19.1	377	42907	6.5	cDNA, FLJ93089, highly similar to sapiens NCK adaptor protein 1 (NCK1), mRNA
2703	P16333	6	9	19.1	377	42864	6.5	Cytoplasmic protein NCK1
2704	Q9UIC8	6	13	19.2	334	38379	6	Leucine carboxyl methyltransferase 1
2704	A8MQ43	6	13	18.9	338	38834	6.5	Uncharacterized protein
2704	Q9UIC8-2	6	13	17.9	357	41106	7	Isoform 2 of Leucine carboxyl methyltransferase 1
2705	Q86UE4	6	8	18.9	582	63837	9.3	Protein LYRIC
2706	Q9Y6G9	6	13	18.9	523	56579	6.4	Cytoplasmic dynein 1 light intermediate chain 1
2707	C9JH30	6	41	18.5	433	47910	4.8	Uncharacterized protein
2707	UPI00001AECF3	6	41	18.3	437	48267	5	UPI00001AECF3 UniRef100 entry
2708	Q8WZA9	6	7	18.3	623	62717	4.9	Immunity-related GTPase family Q protein
2709	P53602	6	7	18.2	400	43405	7.2	Diphosphomevalonate decarboxylase
2710	Q6EMK4	6	10	18	673	71713	7.4	Vasorin
2711	B4E324	6	11	17.7	480	54270	7	cDNA FLJ60397, highly similar to Lysosomal protective protein (EC 3.4.16.5)
2711	UPI0001BC0385	6	11	17.7	481	54256	7	lysosomal protective protein isoform c precursor
2711	P10619	6	11	17.7	480	54466	6.6	Lysosomal protective protein
2711	Q59EV6	6	11	17.1	497	56120	6.6	Carrier family 6, member 8 variant (Fragment)
2712	D6RJA0	6	8	17.7	362	40127	4.8	Uncharacterized protein
2712	Q8WTS6	6	8	17.5	366	40721	4.6	Histone-lysine N-methyltransferase SETD7
2713	Q9BZE9	6	14	17.7	553	60183	6.6	Tether containing UBX domain for GLUT4
2713	Q9BZE9-2	6	14	15.1	647	69990	8.1	Isoform 2 of Tether containing UBX domain for GLUT4
2714	Q9NY27-3	6	18	20.5	361	40249	4.4	Isoform 3 of Serine/threonine-protein phosphatase 4 regulatory subunit 2
2714	Q9NY27	6	18	17.7	417	46898	4.5	Serine/threonine-protein phosphatase 4 regulatory subunit 2
2715	B2RDH6	6	7	17.6	596	67630	7.1	cDNA, FLJ96613, highly similar to sapiens vacuolar protein sorting 33A (yeast) (VPS33A), mRNA
2715	Q96AX1	6	7	17.6	596	67611	7	Vacuolar protein sorting-associated protein 33A
2716	UPI000187F6B4	6	9	17.7	509	57033	9.5	H/ACA ribonucleoprotein complex subunit 4 isoform 2
2716	UPI00015E0408	6	9	17.6	512	57487	9.5	H/ACA ribonucleoprotein complex subunit 4 (EC 5.4.99.-) (Dyskerin) (Nucleolar protein family A member 4) (snoRNP protein DKC1) (Nopp140- associated protein of 57 kDa) (Nucleolar protein NAP57) (CBF5 log).
2716	A8MUT5	6	9	17.5	514	57645	9.5	Uncharacterized protein
2716	O60832	6	9	17.5	514	57674	9.4	H/ACA ribonucleoprotein complex subunit 4
2717	Q14166	6	6	17.5	644	74404	5.5	Tubulin--tyrosine ligase-like protein 12
2718	E5RHC5	6	26	33.5	209	23692	6.4	Uncharacterized protein
2718	Q8N6T3-3	6	26	17.7	396	43776	8.5	Isoform 3 of ADP-ribosylation factor GTPase-activating protein 1
2718	B7ZBI2	6	26	17.4	403	44467	8.6	ADP-ribosylation factor GTPase activating protein 1
2718	Q8N6T3	6	26	17.2	406	44668	5.7	ADP-ribosylation factor GTPase-activating protein 1
2718	Q53F62	6	26	17.2	406	44669	5.4	ADP-ribosylation factor GTPase activating protein 1 isoform a variant (Fragment)
2718	Q8N6T3-2	6	26	16.9	414	45676	5.8	Isoform 2 of ADP-ribosylation factor GTPase-activating protein 1
2719	Q9BRP4	6	9	17.3	392	42190	6.3	Proteasomal ATPase-associated factor 1
2720	B2R8X4	6	7	17.2	454	51267	5	cDNA, FLJ94105, highly similar to sapiens GA binding protein transcription factor, alpha subunit 60kDa (GABPA), mRNA
2720	Q53FW7	6	7	17.2	454	51281	5	GA binding protein transcription factor, alpha subunit (60kD) variant (Fragment)
2720	Q06546	6	7	17.2	454	51295	5	GA-binding protein alpha chain
2721	B3KP96	6	9	22.4	434	50030	6.7	cDNA FLJ31464 fis, clone NT2NE2001337, highly similar to Tripartite motif-containing protein 16
2721	B3KMJ2	6	9	17.2	564	63971	5.4	cDNA FLJ11173 fis, clone PLACE1007346, highly similar to Tripartite motif-containing protein 16
2721	O95361	6	9	17.2	564	63955	5.5	Tripartite motif-containing protein 16
2722	Q9BY77	6	11	17.8	421	46089	10	Polymerase delta-interacting protein 3
2722	B4E0L0	6	11	17.1	438	48102	9.9	cDNA FLJ54030, highly similar to Polymerase delta-interacting protein 3
2722	UPI0001AE632D	6	11	17.1	438	48104	9.9	UPI0001AE632D UniRef100 entry
2723	A8K243	6	9	16.8	387	44746	6.7	cDNA FLJ78172, highly similar to sapiens butyrobetaine (gamma), 2-oxoglutarate dioxygenase (gamma-butyrobetaine hydroxylase) 1 (BBOX1), mRNA
2723	O75936	6	9	16.8	387	44715	6.7	Gamma-butyrobetaine dioxygenase
2724	Q9H1P3-2	6	8	16.9	468	53971	6.6	Isoform 2 of Oxysterol-binding protein-related protein 2
2724	B2RDK3	6	8	16.5	480	55171	6.4	Oxysterol-binding protein
2724	Q9H1P3	6	8	16.5	480	55201	6.4	Oxysterol-binding protein-related protein 2
2725	Q9H3P2	6	29	16.1	528	57277	9	Negative elongation factor A
2725	Q53GS8	6	29	16.1	528	57349	8.9	Wolf-Hirschhorn syndrome candidate 2 protein variant (Fragment)
2725	B3KM78	6	29	15.8	539	58528	9.2	cDNA FLJ10442 fis, clone NT2RP1000738, highly similar to Negative elongation factor A
2726	P38432	6	13	15.5	576	62608	9.1	Coilin

Table S1. Proteins identified in the OVCAR-3 tumor supernatant by two or more unique peptides.

Protein or Protein Group	Accession Number	Unique Peptide Count	Spectrum Count	Sequence Coverage (%)	Length	MolWt	pI	Descriptive Name
2727	B4DHL7	6	14	15.4	773	85155	5.5	cDNA FLJ59515
2727	Q6P996	6	14	15.1	788	86707	5.4	Pyridoxal-dependent decarboxylase domain-containing protein 1
2728	Q5MIZ7-3	6	13	15.1	764	87377	4.8	Isoform 3 of Serine/threonine-protein phosphatase 4 regulatory subunit 3B
2729	Q8IV08	6	29	14.7	490	54705	6.5	Phospholipase D3
2730	A8MWK3	6	7	14.5	875	97040	4.8	Uncharacterized protein
2730	P19022	6	7	14	906	99809	4.8	Cadherin-2
2731	A8K7Q1	6	6	14.3	461	53883	5.2	cDNA FLJ77770, highly similar to sapiens nucleobindin 1 (NUCB1), mRNA
2731	Q53GX6	6	6	14.3	461	53907	5.2	Nucleobindin 1 variant (Fragment)
2731	Q02818	6	6	14.3	461	53879	5.2	Nucleobindin-1
2732	Q5T8D3-2	6	11	15.1	490	54786	5.2	Isoform 2 of Acyl-CoA-binding domain-containing protein 5
2732	B7Z2R7	6	11	14.1	523	58792	5.3	cDNA FLJ55488, highly similar to sapiens acyl-Coenzyme A binding domain containing 5 (ACBD5), mRNA
2732	Q5T8D3-3	6	11	14.1	525	58978	5.3	Isoform 3 of Acyl-CoA-binding domain-containing protein 5
2733	Q9NPQ8-2	6	11	14.3	525	58943	6.6	Isoform 2 of Synembryn-A
2733	Q9NPQ8-4	6	11	14.2	530	59612	5.3	Isoform 4 of Synembryn-A
2733	Q9NPQ8	6	11	14.1	531	59710	5.3	Synembryn-A
2733	Q9NPQ8-3	6	11	14	537	60372	5.3	Isoform 3 of Synembryn-A
2734	E9PEC0	6	20	14	386	40091	5.1	Uncharacterized protein
2734	Q16186	6	20	13.3	407	42153	5.1	Proteasomal ubiquitin receptor ADRM1
2735	P27824	6	15	13.9	592	67568	4.6	Calnexin
2736	Q4G0J3	6	7	13.9	582	66899	9.6	La-related protein 7
2737	Q9BZQ8	6	6	13.9	928	103135	4.8	Protein Niban
2738	D6RFG1	6	11	36	200	23310	5.8	Uncharacterized protein
2738	D2DU65	6	11	36	200	23324	6	OCLN
2738	UPI0001E6F9BC	6	11	26.6	271	31588	5.5	PREDICTED: occludin-like
2738	D2DU64	6	11	26.6	271	31602	5.7	OCLN
2738	UPI0000D60EA9	6	11	15.4	468	52692	5.9	PREDICTED: occludin-like
2738	Q5U1V4	6	11	15.4	468	52706	6.1	Tight junction protein occludin TM4 minus
2738	A8K3T2	6	11	13.8	522	59136	6.1	cDNA FLJ77961, highly similar to sapiens occludin (OCLN), mRNA
2738	UPI000013CA1F	6	11	13.8	522	59130	5.9	PREDICTED: occludin-like
2738	Q16625	6	11	13.8	522	59144	6.1	Occludin
2739	P53609	6	7	13.8	377	42368	6.8	Geranylgeranyl transferase type-1 subunit beta
2740	P08237	6	7	13.7	780	85182	8	6-phosphofructokinase, muscle type
2740	Q6ZTT1	6	7	12.6	851	93220	8	cDNA FLJ44241 fis, clone THYMU3008436, highly similar to 6-phosphofructokinase, muscle type (EC 2.7.1.11)
2741	Q6FIA3	6	9	13.7	445	51353	5.4	PACSIN2 protein
2741	Q9UNF0	6	9	12.6	486	55739	5.2	Protein kinase C and casein kinase substrate in neurons protein 2
2742	Q7Z4Q2	6	17	13.5	680	74583	5.1	HEAT repeat-containing protein 3
2743	A8K5L5	6	7	13.2	479	51375	5.1	cDNA FLJ75689, highly similar to sapiens poliovirus receptor-related 2 (herpesvirus entry mediator B) (PVRL2), mRNA
2743	Q92692-2	6	7	13.2	479	51359	5.1	Isoform Alpha of Poliovirus receptor-related protein 2
2743	Q92692	6	7	11.7	538	57742	4.8	Poliovirus receptor-related protein 2
2744	UPI0000EE22F1	6	8	13.4	647	73477	5.6	UPI0000EE22F1 UniRef100 entry
2744	Q53F02	6	8	12.7	684	77470	5.7	Cleavage and polyadenylation specific factor 3, 73kDa variant (Fragment)
2744	Q9UKF6	6	8	12.7	684	77486	5.6	Cleavage and polyadenylation specificity factor subunit 3
2745	O95747	6	16	12.5	527	58022	6.4	Serine/threonine-protein kinase OSR1
2746	O43422	6	11	12.4	761	87704	5.9	52 kDa repressor of the inhibitor of the protein kinase
2746	Q2YDB3	6	11	12.4	761	87717	6	Protein-kinase, interferon-inducible double stranded RNA dependent inhibitor, repressor of (P58 repressor)
2747	Q96PK6	6	9	12	669	69492	9.7	RNA-binding protein 14
2748	Q8IVT2	6	22	11.2	679	75357	6.8	Uncharacterized protein C19orf21
2749	Q8TC07-2	6	7	11.1	674	77395	5.4	Isoform 2 of TBC1 domain family member 15
2749	B4DMT9	6	7	11	682	78529	5.4	cDNA FLJ58591, highly similar to TBC1 domain family member 15
2749	E9PH93	6	7	11	682	78573	5.3	Uncharacterized protein
2749	Q8TC07	6	7	10.9	691	79491	5.7	TBC1 domain family member 15
2750	A5PLP0	6	10	10.6	594	67256	6.2	SET domain containing 3
2750	Q86TU7	6	10	10.6	594	67257	6	SET domain-containing protein 3
2751	A8MQC0	6	7	10.2	630	73041	8	Uncharacterized protein
2751	Q567U6	6	7	10.1	631	73198	8.2	Coiled-coil domain-containing protein 93
2752	Q53GG0	6	15	9.6	759	85254	6.8	Epithelial protein lost in neoplasm beta variant (Fragment)
2752	Q9UHB6	6	15	9.6	759	85226	6.8	LIM domain and actin-binding protein 1
2752	Q59FE8	6	15	9.5	769	86107	6.8	Epithelial protein lost in neoplasm beta variant (Fragment)
2753	Q9NVE7	6	6	9.6	773	85991	6.3	Pantothenate kinase 4
2753	B4DHW9	6	6	9.5	781	86954	6.4	cDNA FLJ56439, highly similar to Pantothenate kinase 4 (EC 2.7.1.33)
2754	B4DNJ5	6	10	14	378	43369	6.5	cDNA FLJ51908, highly similar to Dolichyl-diphosphooligosaccharide--proteinglycosyltransferase 67 kDa subunit (EC 2.4.1.119)
2754	B7Z4L4	6	10	12.2	435	49921	6.6	cDNA FLJ50809, highly similar to Dolichyl-diphosphooligosaccharide--protein glycosyltransferase 67 kDa subunit (EC 2.4.1.119)
2754	B4DL99	6	10	9.1	581	65838	6.2	cDNA FLJ51740, highly similar to Dolichyl-diphosphooligosaccharide--proteinglycosyltransferase 67 kDa subunit (EC 2.4.1.119)
2754	Q6IBR0	6	10	8.7	607	68607	6.4	RPN1 protein
2754	Q53EP4	6	10	8.7	607	68580	6.4	Ribophorin I variant (Fragment)
2754	P04843	6	10	8.7	607	68569	6.4	Dolichyl-diphosphooligosaccharide--protein glycosyltransferase subunit 1
2755	Q3B768	6	10	12.2	803	90317	6.4	KIAA0406 protein (Fragment)
2755	O43156	6	10	9	1089	122069	6	TEL2-interacting protein 1 log
2756	Q96D71-2	6	17	9.4	705	76838	5.8	Isoform 2 of RalBP1-associated Eps domain-containing protein 1
2756	B2R7D3	6	17	8.9	744	80848	5.8	cDNA, FLJ93390, highly similar to sapiens RALBP1 associated Eps domain containing 1 (REPS1),mRNA
2756	UPI0001F785F6	6	17	8.8	754	81949	6	UPI0001F785F6 UniRef100 entry
2756	UPI0001765CA1	6	17	8.6	769	83650	5.9	ralBP1-associated Eps domain-containing protein 1 isoform b
2756	Q96D71-3	6	17	8.3	795	86591	5.7	Isoform 3 of RalBP1-associated Eps domain-containing protein 1
2756	Q96D71	6	17	8.3	796	86662	5.7	RalBP1-associated Eps domain-containing protein 1
2757	B9EG90	6	15	8.9	765	90700	9.3	Topoisomerase (DNA) I
2757	P11387	6	15	8.9	765	90726	9.3	DNA topoisomerase 1
2758	Q7Z2W4	6	10	10.1	902	101431	8.4	Zinc finger CCCH-type antiviral protein 1
2758	C9J6P4	6	10	8.9	1024	114085	8.6	Uncharacterized protein
2759	D6RHJ4	6	19	8.3	815	82617	7.4	Uncharacterized protein
2759	Q13428-8	6	19	4.8	1412	144413	9	Isoform 8 of Treacle protein
2759	Q13428-2	6	19	4.8	1411	144314	9	Isoform 2 of Treacle protein
2759	Q13428-7	6	19	4.7	1451	148352	8.9	Isoform 7 of Treacle protein

Table S1. Proteins identified in the OVCAR-3 tumor supernatant by two or more unique peptides.

Protein or Protein Group	Accession Number	Unique Peptide Count	Spectrum Count	Sequence Coverage (%)	Length	MolWt	pI	Descriptive Name
2759	Q13428-6	6	19	4.7	1450	148253	8.9	Isoform 6 of Treacle protein
2759	Q13428-3	6	19	4.6	1489	152205	9	Isoform 3 of Treacle protein
2759	Q13428	6	19	4.6	1488	152105	9	Treacle protein
2759	E7ETY2	6	19	4.6	1488	152303	8.9	Uncharacterized protein
2759	Q13428-4	6	19	4.5	1524	155928	8.9	Isoform 4 of Treacle protein
2759	E9PHK9	6	19	4.5	1525	156057	9	Uncharacterized protein
2760	B7Z670	6	8	8.2	1153	127589	6.2	cDNA FLJ61703, highly similar to Neuronal cell adhesion molecule (Fragment)
2760	Q92823-3	6	8	8.1	1180	130668	6	Isoform 3 of Neuronal cell adhesion molecule
2760	Q14CA1	6	8	8.1	1180	130642	6	NRCAM protein
2760	Q92823-4	6	8	8	1183	131046	5.8	Isoform 4 of Neuronal cell adhesion molecule
2760	Q14BM2	6	8	8	1192	131718	6	NRCAM protein
2760	E9PDA4	6	8	8	1192	131744	6	Uncharacterized protein
2760	UPI0001DD3803	6	8	7.8	1211	133813	5.8	neuronal cell adhesion molecule isoform D precursor
2760	Q92823-2	6	8	7.7	1236	136614	6	Isoform 2 of Neuronal cell adhesion molecule
2760	Q92823-5	6	8	7.3	1308	144375	5.7	Isoform 5 of Neuronal cell adhesion molecule
2760	Q92823	6	8	7.3	1304	143889	5.7	Neuronal cell adhesion molecule
2761	Q27J81-2	6	32	8.3	1240	134617	5.5	Isoform 2 of Inverted formin-2
2761	E7EVT0	6	32	8.2	1250	135695	5.4	Uncharacterized protein
2761	Q27J81	6	32	8.2	1249	135624	5.4	Inverted formin-2
2762	Q8IYB7	6	11	7.6	885	99279	6.1	DIS3-like exonuclease 2
2763	O95479	6	9	7.5	791	88893	7.3	GDH/6PGL endoplasmic bifunctional protein
2764	UPI00003665C9	6	7	7.5	1163	124919	7.3	protein turtle log A isoform b
2764	Q9P2J2	6	7	7.4	1179	126580	7.1	Protein turtle log A
2765	Q5T8P6-3	6	7	7.2	980	110666	9.1	Isoform 3 of RNA-binding protein 26
2765	Q5T8P6-2	6	7	7.2	983	111024	9.1	Isoform 2 of RNA-binding protein 26
2765	Q5T8P6	6	7	7.1	1007	113597	9.2	RNA-binding protein 26
2766	B4DET0	6	21	6.9	1220	139681	6.6	cDNA FLJ55900, highly similar to Pre-mRNA-splicing factor ATP-dependent RNA helicase PRP16 (EC 3.6.1.-)
2766	A8K6G9	6	21	6.8	1227	140474	6.5	cDNA FLJ76871, highly similar to sapiens DEAH (Asp-Glu-Ala-His) box polypeptide 38 (DHX38), mRNA
2766	Q92620	6	21	6.8	1227	140502	6.5	Pre-mRNA-splicing factor ATP-dependent RNA helicase PRP16
2767	Q9P260	6	11	6.2	1216	134630	5.4	LisH domain and HEAT repeat-containing protein KIAA1468
2767	Q9P260-2	6	11	6.1	1250	138744	5.5	Isoform 2 of LisH domain and HEAT repeat-containing protein KIAA1468
2768	Q86SE4	6	7	12.2	534	59803	6.4	L1 cell adhesion molecule (Fragment)
2768	A8K139	6	7	5.2	1257	140033	6.2	cDNA FLJ76744, highly similar to sapiens L1 cell adhesion molecule (L1CAM), transcript variant 1, mRNA
2768	UPI00001AEDC0	6	7	5.2	1248	138907	6.2	neural cell adhesion molecule L1 isoform 3 precursor
2768	P32004-2	6	7	5.2	1253	139517	6.2	Isoform 2 of Neural cell adhesion molecule L1
2768	P32004	6	7	5.2	1257	140003	6.2	Neural cell adhesion molecule L1
2769	B7ZLC9	6	7	5.2	1507	168431	6.6	GEMIN5 protein
2769	Q8TEQ6	6	7	5.2	1508	168589	6.6	Gem-associated protein 5
2770	Q8N3D4	6	9	5.2	1523	161854	4.8	EH domain-binding protein 1-like protein 1
2771	Q6ZS30	6	7	3.7	2694	307236	6.4	Neurobeachin-like protein 1
2772	P49750-4	6	10	3.4	2146	241643	6.6	Isoform 4 of YLP motif-containing protein 1
2773	P46013-2	6	14	3.1	2896	319444	9.5	Isoform Short of Antigen KI-67
2773	P46013	6	14	2.7	3256	358695	9.4	Antigen KI-67
2774	B2RWP4	6	15	2.5	2826	296740	4.8	TACC2 protein
2774	O95359-3	6	15	2.5	2871	301056	4.8	Isoform 3 of Transforming acidic coiled-coil-containing protein 2
2774	E9PBC6	6	15	2.5	2875	302507	4.8	Uncharacterized protein
2774	E7EMZ9	6	15	2.5	2826	296740	4.8	Uncharacterized protein
2774	B7ZMJ9	6	15	2.5	2875	302586	4.8	TACC2 protein
2774	O95359	6	15	2.4	2948	309426	4.8	Transforming acidic coiled-coil-containing protein 2
2775	O94915	6	10	2.5	3013	339598	5.6	Protein furry log-like
2776	O95777	5	76	81.2	96	10403	4.5	N-alpha-acetyltransferase 38, NatC auxiliary subunit N-alpha-acetyltransferase 38, NatC auxiliary subunit
2777	P61970	5	296	80.3	127	14478	5.4	Nuclear transport factor 2 Nuclear transport factor 2
2778	P62312	5	27	76.2	80	9128	9.6	U6 snRNA-associated Sm-like protein LSm6 U6 snRNA-associated Sm-like protein LSm6
2779	P62304	5	203	69.6	92	10804	9.4	Small nuclear ribonucleoprotein E Small nuclear ribonucleoprotein E
2780	Q9Y333	5	32	68.4	95	10835	6.5	U6 snRNA-associated Sm-like protein LSm2 U6 snRNA-associated Sm-like protein LSm2
2781	P68366	5	71	64.5	448	49924	5.1	Tubulin alpha-4A chain Tubulin alpha-4A chain
2782	P84090	5	61	64.4	104	12259	5.9	Enhancer of rudimentary log Enhancer of rudimentary log
2783	P36954	5	22	56	125	14523	5.1	DNA-directed RNA polymerase II subunit RPB9 DNA-directed RNA polymerase II subunit RPB9
2784	P62314	5	116	54.6	119	13282	11.6	Small nuclear ribonucleoprotein Sm D1 Small nuclear ribonucleoprotein Sm D1
2785	P0C0S5	5	62	53.9	128	13553	10.6	Histone H2A.Z Histone H2A.Z
2785	Q71UI9	5	62	53.9	128	13509	10.6	Histone H2A.V Histone H2A.V
2786	D4A492	5	72	53.1	81	8988	5.5	Uncharacterized protein
2786	Q15843	5	72	53.1	81	9072	8.4	NEDD8
2786	P29595	5	72	53.1	81	8972	7.2	NEDD8
2787	P62699	5	5	52.9	121	13842	7.3	Protein yippee-like 5 Protein yippee-like 5
2788	Q3U0J1	5	26	51.8	166	18223	8	Peptidyl-prolyl cis-trans isomerase
2788	Q9Y3C6	5	26	51.8	166	18237	8	Peptidyl-prolyl cis-trans isomerase-like 1
2788	Q9D0W5	5	26	51.8	166	18237	8	Peptidyl-prolyl cis-trans isomerase-like 1
2789	P61923	5	170	50.3	177	20198	4.8	Coatomer subunit zeta-1 Coatomer subunit zeta-1
2789	Q53FU3	5	170	50.3	177	20267	4.9	Coatomer protein complex, subunit zeta 1 variant (Fragment)
2790	Q9Y3B4	5	25	48	125	14585	9.4	Pre-mRNA branch site protein p14 Pre-mRNA branch site protein p14
2791	C9JQ41	5	11	46.2	130	15297	8.9	Uncharacterized protein
2791	UPI0000160289	5	11	44.8	134	15610	8.9	PREDICTED: hypothetical protein isoform 1
2791	E9QN04	5	11	44.8	134	15601	8.6	Uncharacterized protein
2791	Q8R3Q6	5	11	41.7	144	16665	8.2	Coiled-coil domain-containing protein 58
2791	Q4VC31	5	11	41.7	144	16620	7.8	Coiled-coil domain-containing protein 58
2792	P60520	5	11	44.4	117	13667	8.1	Gamma-aminobutyric acid receptor-associated protein-like 2 Gamma-aminobutyric acid receptor-associated protein-like 2
2792	UPI0000024215	5	11	44.4	117	13681	8.1	PREDICTED: gamma-aminobutyric acid receptor-associated protein-like 2-like
2793	Q9CQE6	5	10	42.6	204	22943	4.4	Histone chaperone ASF1A
2793	Q9Y294	5	10	42.6	204	22969	4.4	Histone chaperone ASF1A
2794	P60840	5	17	47.1	121	13335	7.2	Alpha-endosulfine
2794	O43768	5	17	47.1	121	13389	7.2	Alpha-endosulfine
2794	E9PB69	5	17	42.9	133	14868	6.6	Uncharacterized protein
2794	O43768-4	5	17	40.7	140	15534	8.7	Isoform 4 of Alpha-endosulfine
2794	C9JUF4	5	17	39.6	144	16047	7.2	Uncharacterized protein
2794	Q5T5H1	5	17	30.5	187	20994	7.8	Endosulfine alpha
2795	B4DJP9	5	38	37	165	20117	10.1	cDNA FLJ58902, highly similar to FUS-interacting serine-arginine-rich protein1
2795	Q5JRI1	5	38	35.5	172	20913	10.5	FUS interacting protein (Serine/arginine-rich) 1

Table S1. Proteins identified in the OVCAR-3 tumor supernatant by two or more unique peptides.

Protein or Protein Group	Accession Number	Unique Peptide Count	Spectrum Count	Sequence Coverage (%)	Length	MolWt	pI	Descriptive Name
2795	O75494-4	5	38	35.3	173	21000	10.5	Isoform 4 of Serine/arginine-rich splicing factor 10
2795	Q5JRI3	5	38	33.5	182	22135	10.3	FUS interacting protein (Serine/arginine-rich) 1 FUS interacting protein (Serine/arginine-rich) 1
2795	Q3UDH7	5	38	33.3	183	22137	10.4	Putative uncharacterized protein
2795	Q3UA07	5	38	33.3	183	22222	10.3	FUS interacting protein (Serine-arginine rich) 1 FUS interacting protein (Serine-arginine rich) 1
2795	O75494-2	5	38	23.4	261	31213	11.3	Isoform 2 of Serine/arginine-rich splicing factor 10 Isoform 2 of Serine/arginine-rich splicing factor 10
2795	Q53GD7	5	38	23.3	262	31282	11.3	FUS interacting protein (Serine-arginine rich) 1 isoform 2 variant (Fragment)
2795	O75494	5	38	23.3	262	31301	11.3	Serine/arginine-rich splicing factor 10 Serine/arginine-rich splicing factor 10
2795	Q3UYX6	5	38	21.9	278	33158	11.1	Putative uncharacterized protein
2796	Q3TR90	5	16	36	178	19822	8.8	Putative uncharacterized protein
2796	Q9JMG7	5	16	31.7	202	22431	8.4	Hepatoma-derived growth factor-related protein 3
2796	Q9Y3E1	5	16	31.5	203	22620	8	Hepatoma-derived growth factor-related protein 3
2796	Q9JMG7-2	5	16	31.2	205	22819	8.6	Isoform 2 of Hepatoma-derived growth factor-related protein 3
2797	P49207	5	29	35.9	117	13293	11.5	60S ribosomal protein L34
2797	Q9D1R9	5	29	35.9	117	13293	11.5	60S ribosomal protein L34
2798	P50461	5	8	33.5	194	20969	8.5	Cysteine and glycine-rich protein 3
2798	P50462	5	8	33.5	194	20895	8.5	Cysteine and glycine-rich protein 3
2799	B4DP84	5	7	32.1	168	20055	4.9	cDNA FLJ57134, moderately similar to DCN1-like protein 4
2799	Q9CXV9	5	7	22.8	237	27579	5.4	DCN1-like protein 5
2799	Q9BTE7	5	7	22.8	237	27508	5.6	DCN1-like protein 5
2800	C9JXB8	5	85	39.7	121	14369	11.3	Uncharacterized protein
2800	E9Q132	5	85	36.1	133	15347	10.9	Uncharacterized protein
2800	C9JNW5	5	85	32	150	17543	10.8	Ribosomal protein L24, isoform CRA_e
2800	P83731	5	85	30.6	157	17779	11.3	60S ribosomal protein L24 60S ribosomal protein L24
2800	E9QNJ0	5	85	30.2	159	18195	11	Uncharacterized protein
2800	Q3UW40	5	85	30	160	18253	11	Putative uncharacterized protein
2801	B4E1D9	5	9	31.6	228	26745	9.7	cDNA FLJ55318, highly similar to Casein kinase I isoform alpha (EC 2.7.11.1)
2801	Q3U772	5	9	21.4	337	38975	9.6	Putative uncharacterized protein
2801	P48729	5	9	21.4	337	38915	9.6	Casein kinase I isoform alpha Casein kinase I isoform alpha
2801	E9PWB2	5	9	19.3	374	43402	9.4	Uncharacterized protein
2802	O75940	5	10	31.1	238	26711	7.3	Survival of motor neuron-related-splicing factor 30
2802	Q8BGT7	5	10	31.1	238	26753	7.3	Survival of motor neuron-related-splicing factor 30
2803	P62279	5	74	43.9	107	12034	10.6	40S ribosomal protein S13 (Fragment)
2803	P62277	5	74	31.1	151	17222	10.5	40S ribosomal protein S13 40S ribosomal protein S13
2803	UPI0000605E36	5	74	31.1	151	17248	10.6	PREDICTED: 40S ribosomal protein S13-like
2803	UPI00001E4734	5	74	31.1	151	17070	10.2	PREDICTED: 40S ribosomal protein S13-like
2804	D3Z3R1	5	27	30.8	104	12130	11.4	60S ribosomal protein L36
2804	D3YW41	5	27	30.5	105	12256	11.4	60S ribosomal protein L36
2804	UPI0000023ED2	5	27	30.5	105	12313	11.3	PREDICTED: 60S ribosomal protein L36-like
2804	Q9Y3U8	5	27	30.5	105	12254	11.6	60S ribosomal protein L36 60S ribosomal protein L36
2804	Q5M9L1	5	27	30.5	105	12297	11.6	60S ribosomal protein L36
2805	O60869-2	5	25	32.4	139	15481	9.8	Isoform 2 of Endothelial differentiation-related factor 1
2805	O60869	5	25	30.4	148	16369	10	Endothelial differentiation-related factor 1
2805	Q9JMG1	5	25	30.4	148	16369	10	Endothelial differentiation-related factor 1
2806	B2RE68	5	8	30.1	196	21681	6.9	cDNA, FLJ94243
2806	Q5W111	5	8	30.1	196	21666	6.7	Chronic lymphocytic leukemia deletion region gene 6 protein
2806	Q3TFQ1	5	8	30.1	196	21681	6.7	Chronic lymphocytic leukemia deletion region gene 6 protein log
2807	O15400-2	5	18	27.6	239	27400	5.1	Isoform 2 of Syntaxin-7
2807	O15400	5	18	25.3	261	29816	5.6	Syntaxin-7
2807	Q9DCE1	5	18	25.3	261	29747	5.6	Putative uncharacterized protein
2807	Q8BH40	5	18	25.3	261	29737	5.6	Syntaxin 7
2807	O70439	5	18	25.3	261	29821	5.8	Syntaxin-7
2808	B4E322	5	7	23.5	328	37368	7.8	cDNA FLJ53135, highly similar to Choline-phosphate cytidylyltransferase A (EC 2.7.7.15)
2808	P49586	5	7	21	367	41667	7	Choline-phosphate cytidylyltransferase A
2808	P49585	5	7	21	367	41731	7.3	Choline-phosphate cytidylyltransferase A
2808	C9JEJ2	5	7	20.3	380	43264	8.5	Uncharacterized protein
2809	E9QKW3	5	15	22.8	268	30833	11.5	Uncharacterized protein
2809	Q09167	5	15	22.7	269	30891	11.6	Serine/arginine-rich splicing factor 5
2809	Q9D8S5	5	15	22.6	270	30978	11.6	MCG7614, isoform CRA_c
2809	O35326	5	15	22.6	270	30945	11.4	Serine/arginine-rich splicing factor 5
2809	Q13243	5	15	22.4	272	31264	11.6	Serine/arginine-rich splicing factor 5
2809	Q59EK7	5	15	18.7	326	37254	11	CS0DF038YO05 variant (Fragment)
2810	Q8NFX7	5	7	21.9	210	23554	9	Syntaxin-binding protein 6
2810	Q8R3T5	5	7	21.9	210	23671	9.2	Syntaxin-binding protein 6
2810	Q8NFX7-2	5	7	21.9	210	23587	9.3	Isoform 2 of Syntaxin-binding protein 6
2811	P09497-2	5	7	21.3	211	23181	4.7	Isoform Non-brain of Clathrin light chain B
2811	Q6IRU5-2	5	7	21.3	211	23174	4.7	Isoform 2 of Clathrin light chain B
2811	Q3TJ95	5	7	21.3	211	23116	4.7	Putative uncharacterized protein
2812	UPI000059DE07	5	7	24.8	270	32003	9.3	UPI000059DE07 UniRef100 entry
2812	UPI00016114EC	5	7	23.3	287	33610	9.5	UPI00016114EC UniRef100 entry
2812	Q9ERI5-2	5	7	22.5	298	35177	8.6	Isoform 2 of Bifunctional arginine demethylase and lysyl-hydroxylase JMJD6
2812	UPI00016114ED	5	7	21.7	309	35863	8.9	UPI00016114ED UniRef100 entry
2812	A4FUU3	5	7	21.1	317	36633	9	Jmjd6 protein (Fragment)
2812	B2WTI3	5	7	20	335	39241	9.5	Phosphatidylserine receptor transcript variant 1
2812	B2WTI1	5	7	20	335	39361	9.6	Phosphatidylserine receptor transcript variant 1
2812	E9PVN0	5	7	19	352	41000	9.3	Uncharacterized protein
2812	B2WTI4	5	7	18.6	361	42003	9.5	Phosphatidylserine receptor transcript variant 2
2812	Q6NYC1-2	5	7	18	372	43109	8.9	Isoform 2 of Bifunctional arginine demethylase and lysyl-hydroxylase JMJD6
2812	Q9ERI5	5	7	16.6	403	46567	8.9	Bifunctional arginine demethylase and lysyl-hydroxylase JMJD6
2812	Q6NYC1	5	7	16.6	403	46462	8.7	Bifunctional arginine demethylase and lysyl-hydroxylase JMJD6
2812	B2WTI2	5	7	16	420	48221	8.2	Phosphatidylserine receptor transcript variant 2
2813	D3Z5F7	5	6	19.8	333	37245	8.4	Uncharacterized protein
2813	UPI00005A1655	5	6	19.8	333	37199	8.4	BCL2L2-PABPN1 protein
2814	Q99496	5	6	19	336	37655	6.8	E3 ubiquitin-protein ligase RING2
2814	Q9CQJ4	5	6	19	336	37623	6.8	E3 ubiquitin-protein ligase RING2
2815	P23258	5	5	18.6	451	51170	6.1	Tubulin gamma-1 chain
2815	P83887	5	5	18.6	451	51101	6	Tubulin gamma-1 chain
2816	A7UJ17	5	5	17	241	26916	7.6	DnaJ
2816	O75190-2	5	5	17	241	26900	7.6	Isoform B of DnaJ log subfamily B member 6
2816	O54946-2	5	5	16.9	242	26978	7.6	Isoform B of DnaJ log subfamily B member 6
2816	O75190-3	5	5	12.6	325	35714	6.9	Isoform C of DnaJ log subfamily B member 6
2816	O75190	5	5	12.6	326	36087	9.1	DnaJ log subfamily B member 6
2816	A8KAG0	5	5	12.6	326	36015	9.2	cDNA FLJ76979, highly similar to sapiens DnaJ (Hsp40) log, subfamily B, member 6 (DNAJB6), transcript variant 1, mRNA
2816	O54946	5	5	11.2	365	39807	9.4	DnaJ log subfamily B member 6
2817	Q9R020-2	5	13	19.5	293	33321	10	Isoform 2 of Zinc finger Ran-binding domain-containing protein 2

Table S1. Proteins identified in the OVCAR-3 tumor supernatant by two or more unique peptides.

Protein or Protein Group	Accession Number	Unique Peptide Count	Spectrum Count	Sequence Coverage (%)	Length	MolWt	pI	Descriptive Name
2817	E9PUD0	5	13	19.5	293	33264	9.9	Uncharacterized protein
2817	Q5R580	5	13	17.8	320	36318	9.8	Zinc finger Ran-binding domain-containing protein 2
2817	Q9R020	5	13	17.3	330	37350	9.9	Zinc finger Ran-binding domain-containing protein 2
2817	O95218	5	13	17.3	330	37404	10	Zinc finger Ran-binding domain-containing protein 2
2817	D3Z4U0	5	13	17.3	330	37295	9.6	Uncharacterized protein
2817	D3Z4T9	5	13	16.8	340	38364	9.9	Uncharacterized protein
2818	E7EQW6	5	7	18	399	44771	7.2	Uncharacterized protein
2818	B4DP99	5	7	18	399	44737	7.2	cDNA FLJ51496, highly similar to Tyrosine-protein kinase CSK (EC 2.7.10.2)
2818	A8K3B6	5	7	16	450	50702	7.1	cDNA FLJ78579, highly similar to sapiens c-src tyrosine kinase (CSK), mRNA
2818	Q8VCW1	5	7	16	450	50716	7.1	C-src tyrosine kinase
2818	Q53EL3	5	7	16	449	50617	7.1	C-src tyrosine kinase variant (Fragment)
2818	Q3UVH2	5	7	16	450	50730	7.1	Putative uncharacterized protein
2818	P41240	5	7	16	450	50704	7.1	Tyrosine-protein kinase CSK
2818	B5BU52	5	7	16	450	50674	7.1	C-src tyrosine kinase
2819	P16403	5	61	16	213	21365	10.9	Histone H1.2
2819	P15864	5	61	16	212	21267	11	Histone H1.2
2819	A3R0T7	5	61	15.5	219	21879	11	Liver histone H1e
2819	Q4VB24	5	61	15.5	219	21893	11	Histone cluster 1, H1e
2819	P10412	5	61	15.5	219	21865	11	Histone H1.4
2819	B2R984	5	61	15.5	219	21893	11	cDNA, FLJ94268, highly similar to sapiens histone 1, H1e (HIST1H1E), mRNA
2819	Q3U292	5	61	15.4	221	22082	11	Putative uncharacterized protein
2819	P43277	5	61	15.4	221	22099	11	Histone H1.3
2819	P16402	5	61	15.4	221	22350	11	Histone H1.3
2820	O95758-1	5	35	15	521	56502	9.2	Isoform 1 of Regulator of differentiation 1
2820	B1ALY5	5	35	14.9	524	56821	9.2	ROD1 regulator of differentiation 1 (S. pombe)
2820	Q8BHD7	5	35	14.9	523	56701	9.1	Regulator of differentiation 1
2820	E9QKT3	5	35	14.2	551	59465	8.9	Uncharacterized protein
2820	O95758	5	35	14.1	552	59690	9	Regulator of differentiation 1
2820	O95758-4	5	35	14	558	60417	9.2	Isoform 4 of Regulator of differentiation 1
2821	Q5JB52	5	11	12.8	508	56467	7.6	Stromal RNA regulating factor
2821	B7WPG3	5	11	12.8	508	56451	7.8	Uncharacterized protein
2821	A8K894	5	11	12.1	537	59667	7.9	cDNA FLJ77927
2821	Q8WVV9-4	5	11	12.1	537	59648	7.7	Isoform 4 of Heterogeneous nuclear ribonucleoprotein L-like
2821	C9J9G0	5	11	12.1	537	59664	7.9	Heterogeneous nuclear ribonucleoprotein L-like, isoform CRA_a
2821	Q8WVV9	5	11	12	542	60083	7.7	Heterogeneous nuclear ribonucleoprotein L-like
2821	D6W592	5	11	12	542	60099	7.9	Heterogeneous nuclear ribonucleoprotein L-like, isoform CRA_d
2821	UPI0001F78AAD	5	11	11.1	587	63225	6.8	UPI0001F78AAD UniRef100 entry
2821	Q9CSH0	5	11	11.1	588	63391	6.9	Putative uncharacterized protein (Fragment)
2821	Q921F4	5	11	11	591	64125	5.8	Heterogeneous nuclear ribonucleoprotein L-like
2822	B5BU99	5	14	9.1	570	61320	9.1	High glucose-regulated protein 8
2822	Q9Y5A9	5	14	9	579	62334	8.8	YTH domain family protein 2
2822	Q91YT7	5	14	9	579	62280	8.8	Putative uncharacterized protein
2822	Q8K325	5	14	9	579	62306	8.8	YTH domain family 2
2822	Q8BM70	5	14	9	579	62308	8.9	Putative uncharacterized protein
2822	Q3TWU3	5	14	9	579	62294	8.8	Putative uncharacterized protein
2822	E9Q2W5	5	14	8.9	584	62788	8.2	Uncharacterized protein
2823	B3KY11	5	21	8.4	800	93234	9.5	cDNA FLJ46571 fis, clone THYMU3041428, highly similar to Probable ATP-dependent RNA helicase DDX23 (EC 3.6.1.-)
2823	Q9BUQ8	5	21	8.2	820	95583	9.6	Probable ATP-dependent RNA helicase DDX23
2823	D3Z0M9	5	21	8.2	819	95495	9.6	MCG18410, isoform CRA_a
2824	Q719H9	5	10	23.7	257	29405	7.1	BTB/POZ domain-containing protein KCTD1 BTB/POZ domain-containing protein KCTD1
2824	Q5M956-2	5	10	23	265	30381	7.1	Isoform 2 of BTB/POZ domain-containing protein KCTD1
2824	E9Q6T9	5	10	7.1	861	96115	5.8	Uncharacterized protein
2824	UPI00018848F1	5	10	7.1	865	96414	5.7	BTB/POZ domain-containing protein KCTD1 isoform b
2825	A2CF96	5	9	5.7	937	104358	5.5	Kinesin family member 1C (Fragment)
2825	Q5SX63	5	9	5.7	937	104342	5.5	Kinesin family member 1C (Fragment)
2825	Q8VI89	5	9	4.8	1100	122469	7.1	Kinesin superfamily protein 1C
2825	O43896	5	9	4.8	1103	122947	6.9	Kinesin-like protein KIF1C
2825	O35071	5	9	4.8	1100	122434	7	Kinesin-like protein KIF1C
2825	E9Q9B0	5	9	4.8	1112	123759	6.9	Uncharacterized protein
2825	A2CF97	5	9	4.8	1100	122450	7	Kinesin family member 1C
2825	Q6A011	5	9	4.7	1120	124573	6.8	MKIAA0706 protein (Fragment)
2826	Q7L2E3-3	5	7	5.3	1155	129438	8.5	Isoform 3 of Putative ATP-dependent RNA helicase DHX30
2826	D3Z2J3	5	7	5.1	1186	133028	8.6	Uncharacterized protein
2826	Q7L2E3	5	7	5.1	1194	133938	8.8	Putative ATP-dependent RNA helicase DHX30
2826	Q99PU8-3	5	7	5	1223	136486	8.4	Isoform 3 of Putative ATP-dependent RNA helicase DHX30
2826	Q99PU8	5	7	5	1217	136668	8.7	Putative ATP-dependent RNA helicase DHX30
2826	Q7L2E3-2	5	7	5	1222	136115	8.6	Isoform 2 of Putative ATP-dependent RNA helicase DHX30
2827	Q60817	5	56	32.6	215	23384	4.6	Nascent polypeptide-associated complex subunit alpha
2827	Q13765	5	56	32.6	215	23384	4.6	Nascent polypeptide-associated complex subunit alpha
2827	E9PAV3	5	56	3.4	2078	205419	9.6	Uncharacterized protein
2827	P70670	5	56	3.2	2187	220599	9.4	Nascent polypeptide-associated complex subunit alpha, muscle-specific form
2827	E9QMB1	5	56	3.2	2187	220497	9.3	Uncharacterized protein
2828	Q923D4	5	12	83.7	86	10119	6.4	Splicing factor 3B subunit 5
2829	Q91VW3	5	48	75.3	93	10477	5.1	SH3 domain-binding glutamic acid-rich-like protein 3
2830	P01887	5	12	67.2	119	13823	8	Beta-2-microglobulin
2831	P52760	5	6	61.5	135	14255	8.7	Ribonuclease UK114
2832	P63254	5	15	59.7	77	8550	8.6	Cysteine-rich protein 1
2832	Q8C2N7	5	15	59.7	77	8562	8.6	Putative uncharacterized protein
2833	Q9CRB1	5	15	56.6	136	15202	6.9	Lectin, galactose binding, soluble 7
2834	O89086	5	33	52.9	153	16605	7.5	Putative RNA-binding protein 3
2835	P62075	5	8	52.6	95	10458	8.2	Mitochondrial import inner membrane translocase subunit Tim13
2836	P32848	5	7	51.8	110	11931	5.2	Parvalbumin alpha
2837	Q61025	5	10	51.5	132	15236	5.1	Intraflagellar transport protein 20 homolog
2838	Q8VBV7	5	14	50.2	209	23256	5.2	COP9 signalosome complex subunit 8
2839	Q9WU28	5	43	50	154	17356	6.3	Prefoldin subunit 5
2840	Q9D967	5	6	48.8	164	18582	6.8	Magnesium-dependent phosphatase 1
2841	P62627	5	27	52.1	96	10990	7.2	Dynein light chain roadblock-type 1
2841	A2AVR9	5	27	48.1	104	11928	6	Dynein light chain roadblock-type 1
2842	P47957	5	11	46.7	180	19724	6.8	Adenine phosphoribosyltransferase
2843	Q9WV85	5	11	44.4	169	19099	6.7	Nucleoside diphosphate kinase 3
2844	Q8VED9	5	15	43.6	172	18956	5.4	Galectin-related protein A
2845	O70591	5	50	42.2	154	16534	6.6	Prefoldin subunit 2
2846	Q64433	5	14	42.2	102	10963	8.3	10 kDa heat shock protein, mitochondrial
2847	Q9QXA5	5	26	41.6	137	15076	10.1	U6 snRNA-associated Sm-like protein LSm4
2847	Q9CY46	5	26	41.3	138	15204	10.1	LSM4 homolog, U6 small nuclear RNA associated (S. cerevisiae), isoform CRA_b
2848	P07091	5	91	40.6	101	11721	5.3	Protein S100-A4
2849	P10639	5	158	40	105	11675	4.9	Thioredoxin

Table S1. Proteins identified in the OVCAR-3 tumor supernatant by two or more unique peptides.

Protein or Protein Group	Accession Number	Unique Peptide Count	Spectrum Count	Sequence Coverage (%)	Length	MolWt	pI	Descriptive Name
2850	Q9CQQ8	5	14	39.8	103	11636	5.3	U6 snRNA-associated Sm-like protein LSm7
2851	Q9Z0F7	5	21	39.8	123	13160	4.7	Gamma-synuclein
2852	Q3U3C2	5	9	38.9	149	16472	7.7	Putative uncharacterized protein
2852	Q9Z0J0	5	9	38.9	149	16442	7.7	Epididymal secretory protein E1
2852	Q3U8F7	5	9	38.9	149	16442	7.7	Putative uncharacterized protein
2853	Q8BN80	5	10	38.9	190	21432	6.8	Putative uncharacterized protein
2853	Q9CX80	5	10	38.9	190	21466	6.8	Cytoglobin
2854	Q9CPT4	5	18	38.6	166	17982	6.8	UPF0556 protein C19orf10 homolog
2855	Q9JM76	5	36	38.2	178	20525	8.6	Actin-related protein 2/3 complex subunit 3
2856	Q8K2Q0	5	11	37.4	198	21850	5.8	COMM domain-containing protein 9
2857	Q9D7S9	5	13	37.4	219	24576	4.8	Charged multivesicular body protein 5
2858	P16254	5	11	37.3	110	12510	10.2	Signal recognition particle 14 kDa protein
2859	Q78KL9	5	9	41	205	23613	6.2	Rexo2 protein
2859	Q3TAV0	5	9	37.5	224	25372	6.6	Putative uncharacterized protein (Fragment)
2859	Q3T9B4	5	9	35.4	237	26667	7.5	Putative uncharacterized protein
2859	Q9D8S4	5	9	35.4	237	26739	7.2	Oligoribonuclease, mitochondrial
2860	Q9CQI7	5	15	35.1	225	25323	9.7	U2 small nuclear ribonucleoprotein B''
2861	Q8JZY2	5	5	33.2	202	22812	6.7	COMM domain-containing protein 10
2862	UPI0000024400	5	24	32.7	214	24547	10.1	PREDICTED: 60S ribosomal protein L10-like isoform 2
2863	Q91X23	5	25	32.4	207	23867	5.6	Orm1 protein
2864	UPI0001547DD8	5	23	33.8	145	16265	9.2	mitochondrial fission 1 protein isoform 2
2864	Q9CQ92	5	23	32.2	152	17009	8.5	Mitochondrial fission 1 protein
2865	B2M0R9	5	7	31.2	157	17774	8.4	TU11 isoform A
2865	P21126	5	7	31.2	157	17801	8.4	Ubiquitin-like protein 4A
2865	B2M0S0	5	7	31.2	157	17791	8.4	TU11 isoform B
2866	Q3TDI5	5	11	29.8	299	32723	4.3	Peroxisome biogenesis factor 19, isoform CRA_e
2866	Q8VCI5	5	11	29.8	299	32733	4.3	Peroxisomal biogenesis factor 19
2867	D3Z6J9	5	29	29.1	148	16682	11	Uncharacterized protein
2867	UPI0000428882	5	29	29.1	148	16560	10.9	PREDICTED: 60S ribosomal protein L27a
2867	P14115	5	29	29.1	148	16605	11.1	60S ribosomal protein L27a
2868	Q8R0G2	5	14	29	231	25084	6.1	Proteasome (Prosome, macropain) 26S subunit, non-ATPase, 10
2868	Q9Z2X2	5	14	29	231	25054	6.1	26S proteasome non-ATPase regulatory subunit 10
2869	O88587	5	23	26.8	265	29496	5.8	Catechol O-methyltransferase
2869	Q91XH4	5	23	26.8	265	29486	5.8	Catechol-O-methyltransferase 1
2870	Q8CI43	5	13	26.6	207	22749	5.5	Myosin light chain 6B
2871	Q8C476	5	7	26.4	261	29438	6.5	Uridine kinase
2871	Q99PM9	5	7	26.4	261	29404	6.5	Uridine-cytidine kinase 2
2872	D3Z7S0	5	11	32.5	197	21972	6.2	Uncharacterized protein
2872	D3Z4J5	5	11	26	246	27580	7	Uncharacterized protein
2872	Q9D1H7-2	5	11	23.4	274	30560	5	Isoform 2 of Golgi to ER traffic protein 4 homolog
2872	Q9D1H7	5	11	19.6	327	36525	5.4	Golgi to ER traffic protein 4 homolog
2873	Q64520	5	7	28.8	198	21918	6.5	Guanylate kinase
2873	Q564G0	5	7	26	219	24032	7.5	Putative uncharacterized protein
2874	Q4FJY5	5	7	25.8	329	35594	8	Ltb4dh protein
2875	P41317	5	13	25.4	244	25957	5.1	Mannose-binding protein C
2876	Q02105	5	6	25.2	246	25966	8.4	Complement C1q subcomponent subunit C
2877	Q60994	5	11	24.7	247	26841	5.6	Adiponectin
2877	Q6GTX4	5	11	24.7	247	26809	5.6	30 kDa adipocyte complement-related protein
2878	Q62446	5	23	22.3	224	25148	9.3	Peptidyl-prolyl cis-trans isomerase FKBP3
2879	Q9CX86	5	18	22	305	30530	9.3	Putative uncharacterized protein
2880	P55264	5	10	21.6	361	40149	6.2	Adenosine kinase
2881	O70209	5	19	21.5	316	34300	7.9	PDZ and LIM domain protein 3
2882	Q3UPH1	5	20	20.8	443	46298	6	Protein PRRC1
2883	P31230	5	26	20.6	310	33997	8.4	Aminoacyl tRNA synthase complex-interacting multifunctional protein 1
2883	Q8C2U7	5	26	20.1	319	35197	8.6	Putative uncharacterized protein
2884	Q3UDK3	5	6	20.4	339	38069	7.8	Putative uncharacterized protein
2884	Q9D0F6	5	6	20.4	339	38096	7.8	Replication factor C subunit 5
2885	O35295	5	10	19.8	324	33901	5.4	Transcriptional activator protein Pur-beta
2886	P58044	5	7	19.4	227	26289	6.2	Isopentenyl-diphosphate Delta-isomerase 1
2887	Q8R010	5	22	18.8	320	35397	8.1	Aminoacyl tRNA synthase complex-interacting multifunctional protein 2
2888	O54724	5	11	18.6	392	43954	5.5	Polymerase I and transcript release factor
2889	Q3TN35	5	7	18.5	314	34194	5.1	Small glutamine-rich tetratricopeptide repeat (TPR)-containing, alpha, isoform CRA_b
2889	Q8BJU0	5	7	18.4	315	34322	5.1	Small glutamine-rich tetratricopeptide repeat-containing protein alpha
2890	P10518	5	6	18.2	330	36024	6.8	Delta-aminolevulinic acid dehydratase
2890	Q9DD05	5	6	18.2	330	36040	6.8	Delta-aminolevulinic acid dehydratase
2891	Q8BWU5	5	9	18.2	335	36301	6.2	Probable O-sialoglycoprotein endopeptidase
2892	Q9D154	5	6	17.4	379	42575	6.2	Leukocyte elastase inhibitor A
2893	Q3TMB3	5	18	16.7	353	40596	5.7	Putative uncharacterized protein
2893	Q920E5	5	18	16.7	353	40582	5.7	Farnesyl pyrophosphate synthase
2893	Q5M8R9	5	18	16.7	353	40565	5.7	Farnesyl diphosphate synthetase
2893	Q3US29	5	18	16.7	353	40509	5.8	Putative uncharacterized protein
2894	Q8R016	5	7	16	455	52511	6.5	Bleomycin hydrolase
2895	E9Q1Z0	5	245	15.8	538	58224	8.3	Uncharacterized protein
2895	Q8BIS2	5	245	15.8	538	58266	8.3	Putative uncharacterized protein
2895	Q148Q7	5	245	15.8	538	58240	8.3	Putative uncharacterized protein
2896	P05202	5	6	15.8	430	47411	9	Aspartate aminotransferase, mitochondrial
2897	P35505	5	6	15	419	46104	7.4	Fumarylacetoacetase
2898	Q3UKJ0	5	7	14.9	850	97463	7.1	Phosphorylase
2898	Q9ET01	5	7	14.9	850	97431	7.1	Glycogen phosphorylase, liver form
2898	Q91WP9	5	7	14.9	850	97431	7.1	Phosphorylase
2899	P51655	5	7	14.2	557	62586	6.3	Glypican-4
2900	O08749	5	5	13.2	509	54272	7.9	Dihydrolipoyl dehydrogenase, mitochondrial
2901	P47738	5	6	12.5	519	56538	7.6	Aldehyde dehydrogenase, mitochondrial
2901	Q3UJW1	5	6	12.5	519	56526	7.6	Putative uncharacterized protein
2901	Q3U9J7	5	6	12.5	519	56511	7.6	Putative uncharacterized protein
2901	Q3U6I3	5	6	12.5	519	56554	7.6	Putative uncharacterized protein
2901	Q3TVM2	5	6	12.5	519	56596	7.4	Putative uncharacterized protein
2902	P24270	5	6	12	527	59765	7.9	Catalase
2902	Q91XI2	5	6	12	527	59738	7.9	Catalase
2902	Q3TVZ1	5	6	12	527	59731	7.9	Catalase
2903	E9QPE7	5	6	11.1	1938	223355	5.5	Uncharacterized protein
2903	Q8R384	5	6	11	1972	227147	5.5	Myh11 protein
2903	Q69ZX3	5	6	10.9	1984	228266	5.5	MKIAA0866 protein (Fragment)
2904	A2A998	5	9	11.4	543	61008	6.1	Complement component 8, alpha polypeptide
2904	A2A997	5	9	10.7	580	65393	6.2	Complement component 8, alpha polypeptide
2904	Q8K182	5	9	10.6	587	66080	6.5	Complement component C8 alpha chain
2905	Q8BTT6	5	5	9.3	772	88818	5.8	Digestive organ expansion factor homolog
2906	Q3U506	5	10	9.1	503	57007	4.8	Putative uncharacterized protein
2907	D3YUZ8	5	10	8.7	755	83951	5.9	Uncharacterized protein

Table S1. Proteins identified in the OVCAR-3 tumor supernatant by two or more unique peptides.

Protein or Protein Group	Accession Number	Unique Peptide Count	Spectrum Count	Sequence Coverage (%)	Length	MolWt	pI	Descriptive Name
2907	E9Q2L4	5	10	8.1	810	90071	6.5	Uncharacterized protein
2907	UPI0001F78ACE	5	10	7.7	852	94973	6.5	UPI0001F78ACE UniRef100 entry
2907	UPI000058BDEF	5	10	7.7	852	94880	6.2	disks large homolog 2
2907	E9PW19	5	10	7.7	859	95531	6.3	Uncharacterized protein
2907	E9Q2L2	5	10	7.6	870	97432	6.5	Uncharacterized protein
2907	UPI0001F78ACC	5	10	6.8	976	109137	6.2	UPI0001F78ACC UniRef100 entry
2908	Q3U7R1	5	9	7.1	1092	121553	6	Extended synaptotagmin-1
2909	A2ATQ4	5	7	4.9	1944	215992	6.4	Anaphase-promoting complex subunit 1
2909	P53995	5	7	4.9	1944	216085	6.4	Anaphase-promoting complex subunit 1
2909	B7ZNX2	5	7	4.9	1944	215991	6.4	Anaphase promoting complex subunit 1
2910	UPI00004293D6	5	5	4.1	1563	173941	7.1	tetratricopeptide repeat protein 37
2911	Q562L9	5	112	84.5	103	11556	6.5	Actin-like protein (Fragment)
2912	Q9BWJ5	5	17	83.7	86	10135	6.4	Splicing factor 3B subunit 5
2913	O96033	5	9	78.4	88	9755	4.7	Molybdopterin synthase sulfur carrier subunit
2914	P06702	5	33	67.5	114	13242	6.1	Protein S100-A9
2915	Q9Y244	5	10	64.5	141	15789	5.1	Proteasome maturation protein
2916	Q6LC01	5	141	63.6	437	48880	4.8	MRNA encoding beta-tubulin. (from clone D-beta-1) (Fragment)
2917	Q9P0R6	5	11	63.3	139	15648	4.5	GSK3-beta interaction protein
2918	Q5EBL8	5	17	62.9	140	16131	7.2	PDZ domain-containing protein 11
2918	Q5EBL8-2	5	17	51.5	171	19492	7.6	Isoform 2 of PDZ domain-containing protein 11
2919	P51161	5	8	62.5	128	14371	6.8	Gastrotropin
2920	P78537	5	12	62.4	125	14311	8.2	Biogenesis of lysosome-related organelles complex 1 subunit 1
2921	Q96HQ2	5	7	57.8	116	13196	5	CDKN2AIP N-terminal-like protein
2922	P05114	5	12	56	100	10659	9.6	Non-histone chromosomal protein HMG-14
2923	Q16864	5	18	68.1	119	13370	5.5	V-type proton ATPase subunit F
2923	C9J2K4	5	18	55.1	147	16402	5.1	V-type proton ATPase subunit F
2924	O95059	5	8	54	124	13693	7.8	Ribonuclease P protein subunit p14
2925	P31151	5	16	53.5	101	11471	6.8	Protein S100-A7
2926	P23297	5	154	53.2	94	10546	4.5	Protein S100-A1
2927	Q9Y5L4	5	9	52.6	95	10500	8.2	Mitochondrial import inner membrane translocase subunit Tim13
2928	P07311	5	15	51.5	99	11261	9.3	Acylphosphatase-1
2929	Q8IY31	5	10	51.5	132	15281	5.1	Intraflagellar transport protein 20 log
2929	Q8IY31-2	5	10	43	158	18120	5.1	Isoform 2 of Intraflagellar transport protein 20 log
2930	O75607	5	29	51.1	178	19344	4.6	Nucleoplasmin-3
2931	B4DJA5	5	15	50.7	201	22178	8.8	cDNA FLJ51867, highly similar to Ras-related protein Rab-5A
2931	P20339	5	15	47.4	215	23659	8.1	Ras-related protein Rab-5A
2932	Q9UJC5	5	7	50.5	107	12326	6.7	SH3 domain-binding glutamic acid-rich-like protein 2
2933	Q99471	5	57	50	154	17328	6.3	Prefoldin subunit 5
2934	Q9BT73	5	15	50	122	13104	7.9	Proteasome assembly chaperone 3
2935	Q86WQ0	5	9	58.3	139	15876	6.2	Nuclear receptor 2C2-associated protein
2935	B4DW92	5	9	48.5	167	19191	7.5	cDNA FLJ60181, highly similar to sapiens TR4 orphan receptor associated protein TRA16, mRNA
2936	Q6FIE5	5	18	47.2	125	13792	6.1	PHP14 protein
2936	Q9NRX4	5	18	47.2	125	13833	6.1	14 kDa phosphohistidine phosphatase
2937	D6RDH4	5	6	46.8	141	16919	4.4	Uncharacterized protein
2937	Q9Y3D8	5	6	38.4	172	20061	4.6	Adenylate kinase isoenzyme 6
2938	P42771	5	28	48.1	156	16533	5.8	Cyclin-dependent kinase inhibitor 2A, isoforms 1/2/3
2938	A5X2G7	5	28	44.9	167	17883	6.9	Cyclin-dependent kinase inhibitor
2939	Q9NUG6	5	12	44.4	133	15511	6	p53 and DNA damage-regulated protein 1
2940	Q3ZCW2	5	17	43.6	172	18986	5.4	Galectin-related protein
2941	Q9Y2Y0-2	5	7	45.4	152	17711	4.5	Isoform 2 of ADP-ribosylation factor-like protein 2-binding protein
2941	Q9Y2Y0	5	7	42.3	163	18822	4.3	ADP-ribosylation factor-like protein 2-binding protein
2942	Q99584	5	25	41.8	98	11471	6.2	Protein S100-A13
2943	Q9BRJ6	5	13	41.8	194	22083	9.6	Uncharacterized protein C7orf50
2944	UPI0001D3B90F	5	11	41.5	147	16236	8.8	UPI0001D3B90F UniRef100 entry
2945	Q502X2	5	5	41.4	186	21233	4.8	Diablo log (Drosophila)
2945	Q6W3F3	5	5	39.5	195	22284	5.9	SMAC3
2945	Q9NR28	5	5	32.2	239	27131	5.9	Diablo log, mitochondrial
2945	Q53HB7	5	5	32.2	239	27068	6.4	Diablo isoform 1 variant (Fragment)
2946	O60888	5	91	40.8	179	19116	5.5	Protein CutA
2946	O60888-2	5	91	36.9	198	20925	5.5	Isoform A of Protein CutA
2947	O00422	5	10	40.5	153	17561	9.4	Histone deacetylase complex subunit SAP18
2947	UPI000198CCAB	5	10	38.8	160	17807	9.6	UPI000198CCAB UniRef100 entry
2947	UPI000187B6C9	5	10	36	172	19526	9.8	histone deacetylase complex subunit SAP18
2948	Q96AT1	5	11	40.3	154	17465	6.1	Uncharacterized protein KIAA1143
2949	Q9Y221	5	12	40	180	20463	8.5	60S ribosome subunit biogenesis protein NIP7 log
2950	E9PLL6	5	42	39.8	108	12201	11.5	Uncharacterized protein
2950	Q6NZ52	5	42	29.1	148	16478	11	Ribosomal protein L27a
2950	P46776	5	42	29.1	148	16561	11	60S ribosomal protein L27a
2951	B2RA50	5	10	39.3	145	16858	9.1	cDNA, FLJ94698, highly similar to sapiens trafficking protein particle complex 1 (TRAPPC1),mRNA
2951	Q9Y5R8	5	10	39.3	145	16832	9.2	Trafficking protein particle complex subunit 1
2952	D3DSE6	5	13	40.8	245	27610	6.6	Chromosome 21 open reading frame 59, isoform CRA_b
2952	C9J818	5	13	38.8	258	29400	6.9	Uncharacterized protein
2952	P57076	5	13	34.5	290	33224	7.5	Uncharacterized protein C21orf59
2953	Q96A00	5	37	38.8	147	16693	9.4	Protein phosphatase 1 regulatory subunit 14A
2954	B7Z9P5	5	15	61.2	139	15002	6.3	cDNA, FLJ78909, highly similar to sapiens intersex-lik (IXL), mRNA
2954	Q9NX70	5	15	42.5	200	21073	6.3	Mediator of RNA polymerase II transcription subunit 29
2954	B4DUA7	5	15	38.5	221	23473	8.2	Intersex-like (Drosophila)
2955	Q9BW83	5	7	38.2	186	20480	5.4	Intraflagellar transport protein 27 log
2956	Q8N5L8	5	8	38	163	17631	10.3	Alba-like protein C9orf23
2957	Q9NZZ3	5	17	37.4	219	24571	4.8	Charged multivesicular body protein 5
2958	Q8WW12	5	38	37.1	178	18925	7.5	PEST proteolytic signal-containing nuclear protein
2959	Q9NVQ4	5	10	36.9	179	20215	5.8	Fas apoptotic inhibitory molecule 1
2959	Q9NVQ4-2	5	10	31	213	24041	6	Isoform 2 of Fas apoptotic inhibitory molecule 1
2960	B4E0Z3	5	17	36.7	150	16892	10.7	cDNA FLJ52178, highly similar to Lamin-B receptor
2960	E7EVK9	5	17	36.7	150	16878	10.7	Uncharacterized protein
2960	C9JXK0	5	17	25.8	213	24284	9.9	Uncharacterized protein
2960	Q14739	5	17	8.9	615	70703	9.4	Lamin-B receptor
2961	Q5JXB2	5	27	36.6	153	17377	5.9	Putative ubiquitin-conjugating enzyme E2 N-like
2962	O95848	5	6	36.5	222	24118	5.1	Uridine diphosphate glucose pyrophosphatase
2963	UPI0001A51B8C	5	7	42.1	216	23566	9.9	tRNA 2""-phosphotransferase 1 isoform 4
2963	Q86TN4	5	7	36	253	27742	10	tRNA 2'-phosphotransferase 1
2963	UPI0001A51B8D	5	7	35.8	254	27813	10	tRNA 2""-phosphotransferase 1 isoform 5
2963	UPI0001A51B8B	5	7	35.7	255	27898	10	tRNA 2""-phosphotransferase 1 isoform 3
2964	B5BU44	5	12	35.7	199	22731	6.8	CASP2 and RIPK1 domain containing adaptor with death domain
2964	Q8IY43	5	12	35.7	199	22732	6.5	CRADD protein
2964	P78560	5	12	35.7	199	22745	6.8	Death domain-containing protein CRADD
2965	P36507	5	11	35.5	400	44424	6.5	Dual specificity mitogen-activated protein kinase kinase 2
2966	D2KTB9	5	18	35.4	274	29071	5.6	Copper chaperone for superoxide dismutase
2966	Q8NEV0	5	18	35.4	274	29027	5.6	Superoxide dismutase copper chaperone
2966	O14618	5	18	35.4	274	29041	5.6	Copper chaperone for superoxide dismutase

Table S1. Proteins identified in the OVCAR-3 tumor supernatant by two or more unique peptides.

Protein or Protein Group	Accession Number	Unique Peptide Count	Spectrum Count	Sequence Coverage (%)	Length	MolWt	pI	Descriptive Name
2967	Q9BQ61	5	12	35.2	176	18419	9.4	Uncharacterized protein C19orf43
2968	Q9NQE9	5	16	35.2	182	20361	6.6	Histidine triad nucleotide-binding protein 3
2969	P08579	5	22	35.1	225	25486	9.7	U2 small nuclear ribonucleoprotein B''
2970	Q16763	5	9	35.1	222	23845	8.4	Ubiquitin-conjugating enzyme E2 S
2971	Q496C9	5	17	34.9	209	23484	8.3	D-tyrosyl-tRNA(Tyr) deacylase
2971	Q8TEA8	5	17	34.9	209	23424	8.2	D-tyrosyl-tRNA(Tyr) deacylase 1
2972	O14893	5	7	34.6	280	31585	5.6	Survival of motor neuron protein-interacting protein 1
2973	E9PNK6	5	13	33.7	166	18661	5.8	Uncharacterized protein
2973	UPI0000418D0B	5	13	32	175	19456	7.6	tumor protein D53 isoform 2
2973	E9PPQ1	5	13	29.3	191	20987	5.3	Uncharacterized protein
2973	UPI00015E0659	5	13	27.5	204	22476	5.6	Tumor protein D53 (hD53) (Tumor protein D52-like 1).
2973	Q16890	5	13	27.5	204	22449	5.6	Tumor protein D53
2973	UPI0001F7878D	5	13	26.8	209	23000	6	Tumor protein D53 (hD53) (Tumor protein D52-like 1).
2974	B4DEX5	5	10	42.5	160	18185	7.4	Prion protein interacting protein, isoform CRA_d
2974	B1AK96	5	10	33.3	204	22890	7.3	Prion protein interacting protein (Fragment)
2974	O43414-2	5	10	30.6	222	25009	6.5	Isoform 2 of ERI1 exoribonuclease 3
2974	C9JA75	5	10	29.4	231	26139	7	Uncharacterized protein
2974	B4DN03	5	10	26.3	259	29152	8.3	Prion protein interacting protein, isoform CRA_c
2974	E9PDK8	5	10	20.3	335	37026	8.1	Uncharacterized protein
2974	O43414	5	10	20.2	337	37238	8.1	ERI1 exoribonuclease 3
2975	P42766	5	27	33.3	123	14551	11	60S ribosomal protein L35
2976	Q86VX2-2	5	8	32.2	199	22412	5.9	Isoform 2 of COMM domain-containing protein 7
2976	Q86VX2	5	8	32	200	22540	5.9	COMM domain-containing protein 7
2977	Q6WWJ7	5	11	32.3	189	21896	4.8	DELTA3PRO-IL-18
2977	B0YJ28	5	11	31.6	193	22307	4.8	Interleukin-18
2977	Q14116	5	11	31.6	193	22326	4.7	Interleukin-18
2978	Q14919	5	19	32.7	205	22350	5.2	Dr1-associated corepressor
2978	Q14919-2	5	19	31.8	211	23148	5.3	Isoform 2 of Dr1-associated corepressor
2978	C9JCC6	5	19	31.6	212	23205	5.3	Uncharacterized protein
2979	UPI000198C5F7	5	8	41.8	146	16825	6.7	UPI000198C5F7 UniRef100 entry
2979	B7Z322	5	8	30.8	198	22093	5.2	cDNA FLJ52146, highly similar to Grancalcin
2979	E7EP31	5	8	30.8	198	22109	5.2	Uncharacterized protein
2979	P28676	5	8	28.1	217	24010	5.2	Grancalcin
2980	Q08623	5	20	33.3	228	25249	5.3	Pseudouridine-5'-monophosphatase
2980	E9PAV8	5	20	30.3	251	27573	5.5	Uncharacterized protein
2981	P37108	5	43	30.1	136	14570	10	Signal recognition particle 14 kDa protein
2982	Q96PC3-4	5	8	29.9	154	18477	6.3	Isoform 4 of AP-1 complex subunit sigma-3
2983	Q14691	5	6	28.6	196	22988	7.4	DNA replication complex GINS protein PSF1
2984	O95983-2	5	7	32	259	29014	5.2	Isoform 2 of Methyl-CpG-binding domain protein 3
2984	O95983	5	7	28.5	291	32844	5.3	Methyl-CpG-binding domain protein 3
2985	P53384-2	5	7	28.8	309	33412	5.2	Isoform 2 of Cytosolic Fe-S cluster assembly factor NUBP1
2985	P53384	5	7	27.8	320	34535	5.3	Cytosolic Fe-S cluster assembly factor NUBP1
2986	Q53FE5	5	10	27.8	263	28343	8.4	Putative uncharacterized protein (Fragment)
2986	Q9NVZ3	5	10	27.8	263	28339	8.4	Adaptin ear-binding coat-associated protein 2
2986	Q9NVZ3-2	5	10	26.7	273	29464	8.4	Isoform 2 of Adaptin ear-binding coat-associated protein 2
2987	Q9H299	5	49	75.3	93	10438	4.9	SH3 domain-binding glutamic acid-rich-like protein 3
2987	UPI0000D61E9C	5	49	31.1	225	23699	9.1	SH3 domain-binding glutamic acid-rich-like protein 3 (SH3 domain-binding protein 1) (SH3BP-1).
2987	Q86Z22	5	49	31	226	23838	9.1	Putative uncharacterized protein
2987	D3DPK5	5	49	27.2	257	26819	8.4	SH3 domain binding glutamic acid-rich protein like 3, isoform CRA_a (Fragment)
2988	Q9NRX1	5	7	27	252	27924	9.7	RNA-binding protein PNO1
2989	B4DQN2	5	8	26.9	208	23904	8.4	cDNA FLJ52998
2989	UPI0001C0B3BE	5	8	26.9	208	23937	8.4	hypothetical protein LOC63932 isoform 3
2989	Q9H5V9	5	8	25.2	222	25625	8.7	UPF0428 protein CXorf56
2990	Q6ICL3-3	5	6	27.1	273	30345	5.1	Isoform 3 of Uncharacterized protein C22orf25
2990	A8K1E7	5	6	26.8	276	30903	5.2	cDNA FLJ76702
2990	Q6ICL3	5	6	26.8	276	30937	5.2	Uncharacterized protein C22orf25
2990	C9JC99	5	6	26.3	281	31170	5	Uncharacterized protein
2990	B7WNV6	5	6	23.3	317	34952	5.3	Chromosome 22 open reading frame 25, isoform CRA_c
2991	Q9UBV8	5	7	26.8	284	30381	6.5	Peflin
2992	B5MC20	5	7	26.7	217	24796	5	Uncharacterized protein
2992	Q9NWJ5	5	7	24.7	235	26953	5	cDNA FLJ20807 fis, clone ADSE01784
2992	E7EX06	5	7	24.6	236	26881	5.2	Uncharacterized protein
2992	P0C870	5	7	18.4	316	35932	5.4	JmjC domain-containing protein 7
2993	B2R4V2	5	20	26.4	106	12527	10.7	cDNA, FLJ92227, highly similar to sapiens ribosomal protein L36a-like (RPL36AL), mRNA
2993	Q969Q0	5	20	26.4	106	12469	10.7	60S ribosomal protein L36a-like
2994	O60921	5	6	25.7	280	31691	6.8	Checkpoint protein HUS1
2995	Q9ULC3	5	6	25.7	237	26659	6.6	Ras-related protein Rab-23
2996	P09496-2	5	19	25.2	218	23662	4.5	Isoform Non-brain of Clathrin light chain A
2997	Q9H977	5	6	24.6	334	35891	6.2	WD repeat-containing protein 54
2998	Q9NVM6	5	11	24.3	304	34687	8.5	DnaJ log subfamily C member 17
2999	Q8TDN6	5	6	23.8	353	41401	9.9	Ribosome biogenesis protein BRX1 log
3000	UPI00001AE7EF	5	7	23.9	243	26598	9	Probable ribosome biogenesis protein NEP1 (Nucleolar protein EMG1 log) (Protein C2f).
3000	Q92979	5	7	23.8	244	26720	9.2	Ribosomal RNA small subunit methyltransferase NEP1
3001	P40429	5	10	23.6	203	23577	10.9	60S ribosomal protein L13a
3001	Q5QTS3	5	10	23.6	203	23662	11	FWP004
3001	Q53H34	5	10	23.6	203	23558	10.9	Ribosomal protein L13a variant (Fragment)
3002	Q96AU5	5	6	25.8	353	40990	6.4	Protein tyrosine phosphatase, non-receptor type 2
3002	A8K3N4	5	6	23.5	387	45168	8.1	Protein tyrosine phosphatase, non-receptor type 2, isoform CRA_b
3002	P17706	5	6	21.9	415	48473	8.3	Tyrosine-protein phosphatase non-receptor type 2
3003	Q8ND76-3	5	20	27.5	287	33245	7.7	Isoform 3 of Cyclin-Y
3003	Q8ND76-2	5	20	25	316	36504	7.6	Isoform 2 of Cyclin-Y
3003	B2RBH6	5	20	23.2	341	39254	7.2	cDNA, FLJ95513, highly similar to sapiens cyclin fold protein 1 (CFP1), mRNA
3003	Q8ND76	5	20	23.2	341	39337	7.2	Cyclin-Y
3004	B2R5I8	5	5	23.1	225	24956	6.7	cDNA, FLJ92490, highly similar to sapiens RAB32, member RAS oncogene family (RAB32), mRNA
3004	Q13637	5	5	23.1	225	24997	6.5	Ras-related protein Rab-32
3005	D6REQ6	5	23	31.7	218	25309	6.1	Ribonuclease T2, isoform CRA_c
3005	O00584	5	23	27	256	29481	7.1	Ribonuclease T2
3005	A6XND5	5	23	22.5	307	35598	8.1	Ribonuclease T2
3006	B3KWK7	5	5	28.4	194	21844	9.4	Insulin-like growth factor binding protein 3, isoform CRA_b
3006	B3KTQ0	5	5	22.6	243	26735	9.5	cDNA FLJ38581 fis, clone HCHON2008672, highly similar to Insulin-like growth factor-binding protein 3
3006	B4DP07	5	5	22.4	246	27281	8.8	cDNA FLJ53634, highly similar to Insulin-like growth factor-binding protein 3
3006	A6XNC9	5	5	22.2	248	27273	9.3	Insulin-like growth factor binding protein 3 isoform b
3006	A6XND1	5	5	20.9	263	28980	8.5	Insulin-like growth factor binding protein 3 isoform b
3006	B3KVF9	5	5	20.5	268	29321	9	cDNA FLJ16507 fis, clone HCHON2000364, highly similar to Insulin-like growth factor-binding protein 3

Table S1. Proteins identified in the OVCAR-3 tumor supernatant by two or more unique peptides.

Protein or Protein Group	Accession Number	Unique Peptide Count	Spectrum Count	Sequence Coverage (%)	Length	MolWt	pI	Descriptive Name
3006	A6XND0	5	5	20.4	270	29747	8.5	Insulin-like growth factor binding protein 3
3006	B4DN53	5	5	19.9	276	30138	9	cDNA FLJ52568, highly similar to Insulin-like growth factor-binding protein 3
3006	P17936	5	5	18.9	291	31674	8.7	Insulin-like growth factor-binding protein 3
3007	O95721	5	7	22.1	258	28970	5.8	Synaptosomal-associated protein 29
3008	P18440	5	10	22.1	290	33899	6.5	Arylamine N-acetyltransferase 1
3008	Q9HAQ5	5	10	22.1	290	33883	6.5	Arylamine N-acetyltransferase 1
3008	UPI0000210A3F	5	10	18.2	352	40846	6.9	arylamine N-acetyltransferase 1 isoform b
3009	B4DIA9	5	5	21.7	341	37090	6.2	cDNA FLJ61687, highly similar to sapiens UEV and lactate/malate dehyrogenase domains (UEVLD), transcript variant 1, mRNA
3009	E7EPQ7	5	5	16.5	449	49750	7.1	Uncharacterized protein
3009	B4DL43	5	5	16.5	449	49664	7.3	cDNA FLJ54696, highly similar to sapiens UEV and lactate/malate dehyrogenase domains (UEVLD), transcript variant 1, mRNA
3009	Q8IX04	5	5	15.7	471	52264	7.1	Ubiquitin-conjugating enzyme E2 variant 3
3010	Q9NRN7	5	8	21.7	309	35776	6.8	L-aminoadipate-semialdehyde dehydrogenase-phosphopantetheinyl transferase
3011	Q0VDK3	5	6	21.3	367	41009	5.8	AS3MT protein (Fragment)
3011	Q0VDK4	5	6	21.3	366	40908	6.1	AS3MT protein (Fragment)
3011	Q9HBK9	5	6	20.8	375	41748	6.2	Arsenite methyltransferase
3012	B3KTN5	5	6	20.7	450	50853	7.6	cDNA FLJ38538 fis, clone HCHON2001407, highly similar to LanC-like protein 2
3012	Q9NS86	5	6	20.7	450	50854	7.4	LanC-like protein 2
3013	B4DDZ4	5	5	20.4	299	33614	5.9	cDNA FLJ51794, highly similar to Annexin A4
3013	Q6P452	5	5	20.4	299	33552	5.9	ANXA4 protein
3013	P09525	5	5	19.1	319	35883	6.1	Annexin A4
3014	Q15527	5	8	20.3	256	29648	9.2	Surfeit locus protein 2
3015	E7EQ61	5	11	19.9	377	41851	5	Uncharacterized protein
3015	Q9GZZ9	5	11	18.6	404	44863	4.8	Ubiquitin-like modifier-activating enzyme 5
3016	Q59GT6	5	10	20.6	320	35915	5.4	Rab geranylgeranyltransferase, beta subunit variant (Fragment)
3016	P53611	5	10	19.9	331	36924	5	Geranylgeranyl transferase type-2 subunit beta
3017	Q5T6V5	5	5	19.9	341	39029	5.9	UPF0553 protein C9orf64
3018	Q8N9M1	5	17	19.9	422	44746	10.1	Uncharacterized protein C19orf47
3019	Q9NWT6	5	5	21.8	349	40285	5.6	Hypoxia-inducible factor 1-alpha inhibitor
3019	B4DI08	5	5	19.8	383	44275	6.2	cDNA FLJ60091, highly similar to Hypoxia-inducible factor 1 alpha inhibitor(EC 1.14.11.16)
3020	Q96M27	5	21	20.7	445	46701	5.8	Protein PRRC1
3020	Q96M27-3	5	21	19.9	462	48907	5.3	Isoform 3 of Protein PRRC1
3020	C9J436	5	21	19.8	464	48467	5.5	Uncharacterized protein
3020	Q96M27-2	5	21	19.8	464	48525	5.4	Isoform 2 of Protein PRRC1
3021	Q96BN8	5	6	19.3	352	40263	5.5	Protein FAM105B
3022	P78318	5	11	19.2	339	39222	5.4	Immunoglobulin-binding protein 1
3023	Q86W42	5	5	19.1	341	37535	7.4	THO complex subunit 6 log
3024	Q8NFH4	5	6	19	326	36708	5.9	Nucleoporin Nup37
3025	Q6GMV2	5	13	18.9	418	47341	5	SET and MYND domain-containing protein 5
3026	B4DV96	5	10	18.5	286	30361	6.5	cDNA FLJ60169, highly similar to Ribokinase (EC 2.7.1.15)
3026	Q9H477	5	10	16.5	322	34143	5	Ribokinase
3027	B3KTN4	5	5	18.3	421	47077	7.2	Citrate synthase
3027	B4DJV2	5	5	17	453	50432	7.9	Citrate synthase
3027	O75390	5	5	16.5	466	51712	8.3	Citrate synthase, mitochondrial
3028	Q68D16	5	5	18.8	388	43487	8.2	Putative uncharacterized protein DKFZp686D0638 (Fragment)
3028	A8MT37	5	5	18.2	401	44920	8.2	Uncharacterized protein
3028	P49840	5	5	15.1	483	50981	8.7	Glycogen synthase kinase-3 alpha
3029	Q96GK7	5	6	18.2	314	34596	8.2	Fumarylacetoacetate hydrolase domain-containing protein 2A
3030	B3KUZ8	5	6	18.1	371	41326	8.8	Aspartate aminotransferase
3030	E7ERW2	5	6	17.3	387	43030	9.3	Aspartate aminotransferase
3030	B4DJA6	5	6	17.3	387	42987	9.3	Aspartate aminotransferase
3030	P00505	5	6	15.6	430	47518	9	Aspartate aminotransferase, mitochondrial
3031	Q96BW5-2	5	10	20.9	302	33436	7	Isoform 2 of Phosphotriesterase-related protein
3031	Q96BW5	5	10	18.1	349	39018	6.5	Phosphotriesterase-related protein
3032	Q147W7	5	5	18	345	37522	5.2	KRT1B protein
3033	Q7Z6M1	5	5	17.7	372	40565	6.3	Rab9 effector protein with kelch motifs
3034	A8K245	5	12	17.4	396	45446	8.9	cDNA FLJ75441, highly similar to sapiens vaccinia related kinase 1 (VRK1), mRNA
3034	Q99986	5	12	17.4	396	45476	8.9	Serine/threonine-protein kinase VRK1
3035	P15104	5	6	17.4	373	42064	6.9	Glutamine synthetase
3036	Q9GZT9-2	5	8	18.3	404	43666	8.5	Isoform 2 of Egl nine log 1
3036	Q9GZT9	5	8	17.4	426	46021	8.5	Egl nine log 1
3037	Q7Z6K5	5	5	30.1	226	24943	5.8	UPF0552 protein C15orf38
3037	E2QRD5	5	5	17.3	394	43882	5.3	Uncharacterized protein
3038	Q8IYS1	5	5	17.2	436	47776	5.8	Peptidase M20 domain-containing protein 2
3039	B4DW31	5	14	17.1	515	57958	5.1	cDNA FLJ54186, highly similar to Nuclear receptor-binding protein
3039	Q9UHY1	5	14	16.4	535	59845	5.1	Nuclear receptor-binding protein
3040	B4DEB2	5	10	17	400	43221	9.5	cDNA FLJ56491, highly similar to DNA-repair protein XRCC1
3040	E9PEU6	5	10	17	401	43322	9.5	Uncharacterized protein
3040	UPI000013D351	5	10	10.7	633	69449	6.3	DNA-repair protein XRCC1 (X-ray repair cross-complementing protein 1).
3040	P18887	5	10	10.7	633	69477	6.4	DNA repair protein XRCC1
3040	Q59HH7	5	10	10.5	647	71014	6	X-ray repair cross complementing protein 1 variant (Fragment)
3041	B4DI11	5	9	17	417	47670	6	cDNA FLJ55569, highly similar to sapiens secernin 3 (SCRN3), mRNA
3041	Q0VDG5	5	9	16.7	426	48840	5.6	Secernin-3
3041	Q0VDG4	5	9	16.7	424	48544	5.6	Secernin-3
3042	A8K7T8	5	6	16.9	360	40924	6.5	cDNA FLJ77776, highly similar to sapiens hyaluronan and proteoglycan link protein 3 (HAPLN3), mRNA
3042	Q96S86	5	6	16.9	360	40894	6.5	Hyaluronan and proteoglycan link protein 3
3043	B2RCP7	5	11	16.9	349	38011	8	cDNA, FLJ96197, highly similar to sapiens connective tissue growth factor (CTGF), mRNA
3043	Q5M8T4	5	11	16.9	349	38069	7.9	Connective tissue growth factor
3044	Q86VN1-2	5	7	19.2	328	36958	7.5	Isoform 2 of Vacuolar protein-sorting-associated protein 36
3044	Q86VN1	5	7	16.3	386	43817	7.2	Vacuolar protein-sorting-associated protein 36
3045	Q9Y259	5	7	16.2	395	45271	5.5	Choline/ethanolamine kinase
3046	Q96FK6	5	6	16	387	43215	6.1	WD repeat-containing protein 89
3047	Q9UHR4	5	10	15.9	511	56883	8.7	Brain-specific angiogenesis inhibitor 1-associated protein 2-like protein 1
3048	P26022	5	7	15.7	381	41976	5	Pentraxin-related protein PTX3
3049	E7EPY4	5	7	15.4	447	50520	6.4	Uncharacterized protein
3049	Q9NUU7	5	7	14.4	478	53975	6.6	ATP-dependent RNA helicase DDX19A
3050	Q07666-2	5	32	16	418	45861	7.6	Isoform 2 of KH domain-containing, RNA-binding, signal transduction-associated protein 1
3050	E7ET98	5	32	15.8	423	46429	8.8	Uncharacterized protein

Table S1. Proteins identified in the OVCAR-3 tumor supernatant by two or more unique peptides.

Protein or Protein Group	Accession Number	Unique Peptide Count	Spectrum Count	Sequence Coverage (%)	Length	MolWt	pI	Descriptive Name
3050	B4DDX2	5	32	15.8	423	46459	8.8	cDNA FLJ54590, highly similar to KH domain-containing, RNA-binding, signaltransduction-associated protein 1
3050	B2R7B5	5	32	15.1	443	48225	8.7	cDNA, FLJ93365, highly similar to sapiens KH domain containing, RNA binding, signal transduction associated 1 (KHDRBS1), mRNA
3050	Q07666	5	32	15.1	443	48227	8.7	KH domain-containing, RNA-binding, signal transduction-associated protein 1
3051	Q7L5Y1-2	5	7	19.7	361	41024	6.5	Isoform 2 of Mitochondrial enolase superfamily member 1
3051	Q7L5Y1	5	7	16	443	49787	6.5	Mitochondrial enolase superfamily member 1
3051	Q6ZS08	5	7	15	474	52468	5.2	Enolase superfamily member 1 (Protein rTS)
3052	B2R8N0	5	9	14.9	455	52935	7.3	cDNA, FLJ93975, highly similar to sapiens epoxide hydrolase 1, microsomal (xenobiotic) (EPHX1), mRNA
3052	Q6FGZ3	5	9	14.9	455	52961	7.3	EPHX1 protein (Fragment)
3052	P07099	5	9	14.9	455	52949	7.3	Epoxide hydrolase 1
3053	P57772	5	7	14.9	596	65305	8.4	Selenocysteine-specific elongation factor
3054	B4DJ85	5	10	14.5	580	66709	5.3	cDNA FLJ60587, highly similar to Glomulin
3054	Q92990	5	10	14.1	594	68208	5.3	Glomulin
3055	Q92466	5	7	14.5	427	47864	9.5	DNA damage-binding protein 2
3056	B4E1W3	5	7	14.4	444	50080	6.8	cDNA FLJ51732, highly similar to Peroxisomal NADH pyrophosphatase NUDT12 (EC 3.6.1.22)
3056	E7EM93	5	7	14.4	444	50052	6.8	Uncharacterized protein
3056	Q9BQG2	5	7	13.9	462	52076	6.8	Peroxisomal NADH pyrophosphatase NUDT12
3057	B3KN55	5	6	14.3	474	53821	4.8	cDNA FLJ13636 fis, clone PLACE1011160, highly similar to RING finger protein 14
3057	Q9UBS8	5	6	14.3	474	53837	4.8	E3 ubiquitin-protein ligase RNF14
3058	B3KPC1	5	14	14.3	385	43378	6.1	cDNA FLJ31582 fis, clone NT2RI2002117, highly similar to Protein pelota log
3058	Q9BRX2	5	14	14.3	385	43359	6.3	Protein pelota log
3059	Q9NYB0	5	5	14.3	399	44260	4.7	Telomeric repeat-binding factor 2-interacting protein 1
3060	Q5T2T1	5	7	14.1	576	65524	7.1	MAGUK p55 subfamily member 7
3061	Q6PJY9	5	20	27.5	247	24976	6.8	SFRS11 protein (Fragment)
3061	Q6PKC9	5	20	21.2	321	34277	11.5	SFRS11 protein (Fragment)
3061	Q6PJB9	5	20	17.3	394	42957	11.7	SFRS11 protein (Fragment)
3061	Q8IWE6	5	20	14.1	483	53414	10.5	Splicing factor, arginine/serine-rich 11
3061	Q05519	5	20	14	484	53542	10.5	Serine/arginine-rich splicing factor 11
3062	B4DUE0	5	6	13.9	411	45800	6.3	cDNA FLJ58627
3062	E7ES35	5	6	13.9	411	45881	6.2	Uncharacterized protein
3062	Q5R3I4	5	6	12.2	469	52787	6	Tetratricopeptide repeat protein 38
3063	Q5JUA8	5	5	13.9	525	59347	7.5	Vacuolar protein sorting 16 log (S. cerevisiae)
3063	Q9H269-2	5	5	10.5	695	78300	7.9	Isoform 2 of Vacuolar protein sorting-associated protein 16 log
3063	Q9H269	5	5	8.7	839	94694	6.8	Vacuolar protein sorting-associated protein 16 log
3064	B3KRB2	5	6	13.8	661	75008	6.4	cDNA FLJ33964 fis, clone CTONG2019029, highly similar to Pseudouridylate synthase 7 log (EC 5.4.99.-)
3064	Q96PZ0	5	6	13.8	661	75035	6.4	Pseudouridylate synthase 7 log
3064	B3KY42	5	6	13.8	661	75036	6.2	cDNA FLJ46788 fis, clone TRACH3028855, highly similar to Pseudouridylate synthase 7 log (EC 5.4.99.-)
3065	Q9BTD8-4	5	12	14.8	446	47025	9.5	Isoform 4 of RNA-binding protein 42
3065	Q9BTD8-2	5	12	14.7	450	47322	9.5	Isoform 2 of RNA-binding protein 42
3065	Q9BTD8-3	5	12	14.6	451	47393	9.5	Isoform 3 of RNA-binding protein 42
3065	Q9BTD8	5	12	13.8	480	50414	9.6	RNA-binding protein 42
3066	Q01415	5	6	13.5	458	50378	6.6	N-acetylgalactosamine kinase
3067	Q13451	5	5	13.3	457	51212	5.9	Peptidyl-prolyl cis-trans isomerase FKBP5
3067	Q53GX4	5	5	13.3	457	51198	5.9	FK506 binding protein 5 variant (Fragment)
3067	Q2TA84	5	5	13.3	457	51178	5.9	FK506 binding protein 5
3068	P01009-2	5	7	15.3	359	40263	5.5	Isoform 2 of Alpha-1-antitrypsin
3068	P01009	5	7	13.2	418	46737	5.6	Alpha-1-antitrypsin
3069	O14879	5	6	12.9	490	55985	5.2	Interferon-induced protein with tetratricopeptide repeats 3
3070	Q86V87	5	6	12.8	743	82340	5.5	Protein FAM160B2
3071	B7ZLW0	5	10	12.7	612	65774	7.4	LPP protein
3071	Q93052	5	10	12.7	612	65746	7.4	Lipoma-preferred partner
3072	Q8TBX8	5	11	12.6	421	47300	6.8	Phosphatidylinositol-5-phosphate 4-kinase type-2 gamma
3073	A8K9K6	5	9	11.3	594	65978	9.2	cDNA FLJ76962, highly similar to sapiens nucleolar protein 5A (56kDa with KKE/D repeat) (NOL5A), mRNA
3073	O00567	5	9	11.3	594	66050	9.2	Nucleolar protein 56
3074	B7Z7Y3	5	5	11.2	669	73744	6.9	cDNA FLJ61618, highly similar to Discoidin, CUB and LCCL domain-containing protein 2
3074	Q96PD2	5	5	9.7	775	85035	7.2	Discoidin, CUB and LCCL domain-containing protein 2
3074	Q96PD2-2	5	5	9.5	789	86657	7.2	Isoform 2 of Discoidin, CUB and LCCL domain-containing protein 2
3075	O43493-2	5	6	12.4	437	45880	5.6	Isoform TGN46 of Trans-Golgi network integral membrane protein 2
3075	O43493-5	5	6	12.1	447	47058	5.6	Isoform 5 of Trans-Golgi network integral membrane protein 2
3075	UPI000020880A	5	6	11.9	453	47774	6	UPI000020880A UniRef100 entry
3075	UPI0000208809	5	6	11.9	454	47880	5.8	UPI0000208809 UniRef100 entry
3075	O43493-3	5	6	11.9	453	47698	6	Isoform TGN48 of Trans-Golgi network integral membrane protein 2
3075	O43493	5	6	11.2	480	51113	5.7	Trans-Golgi network integral membrane protein 2
3076	P29350-2	5	8	11.5	556	63125	7.2	Isoform 3 of Tyrosine-protein phosphatase non-receptor type 6
3076	B4DPS0	5	8	11	583	66228	7.2	cDNA FLJ60320, highly similar to Tyrosine-protein phosphatase non-receptor type6 (EC 3.1.3.48)
3076	Q53EL0	5	8	10.8	595	67609	7.8	Protein tyrosine phosphatase, non-receptor type 6 isoform 1 variant (Fragment)
3076	P29350	5	8	10.8	595	67561	7.8	Tyrosine-protein phosphatase non-receptor type 6
3076	UPI0001AE6A5B	5	8	10.7	597	67742	7.6	UPI0001AE6A5B UniRef100 entry
3076	Q53XS4	5	8	10.7	597	67719	7.8	Protein tyrosine phosphatase, non-receptor type 6
3076	UPI000014049A	5	8	10.3	624	70131	7.9	tyrosine-protein phosphatase non-receptor type 6 isoform 3
3076	Q9UK67	5	8	10.3	624	70117	7.9	70 kDa SHP-1L protein
3076	C9JG49	5	8	10.2	626	70289	7.9	Protein tyrosine phosphatase, non-receptor type 6, isoform CRA_b
3077	B3KU23	5	9	12.7	607	69737	6.5	cDNA FLJ39097 fis, clone NTONG2000977, highly similar to Neurolysin, mitochondrial (EC 3.4.24.16)
3077	A8K9T8	5	9	10.9	704	80678	6.6	cDNA FLJ76106, highly similar to sapiens neurolysin (metallopeptidase M3 family) (NLN), mRNA
3077	Q9BYT8	5	9	10.9	704	80652	6.6	Neurolysin, mitochondrial
3078	Q8NFF5-2	5	8	13.1	490	54188	6.1	Isoform 2 of FAD synthase
3078	Q8NFF5	5	8	10.9	587	65266	6.9	FAD synthase
3079	O95831	5	9	10.3	613	66901	9	Apoptosis-inducing factor 1, mitochondrial
3079	O95831-3	5	9	10.3	609	66295	8.9	Isoform 3 of Apoptosis-inducing factor 1, mitochondrial
3080	Q5T2E6	5	8	10.3	689	78710	6.6	UPF0668 protein C10orf76
3081	Q9UNF1	5	6	10.2	606	64954	9.3	Melanoma-associated antigen D2
3082	Q5R363	5	7	12.1	547	62033	5.7	SFRS protein kinase 1
3082	B4DS61	5	7	10.3	639	72384	5.6	SFRS protein kinase 1, isoform CRA_f

Table S1. Proteins identified in the OVCAR-3 tumor supernatant by two or more unique peptides.

Protein or Protein Group	Accession Number	Unique Peptide Count	Spectrum Count	Sequence Coverage (%)	Length	MolWt	pI	Descriptive Name
3082	A8K8B2	5	7	10.1	655	74282	6.1	cDNA FLJ78127, highly similar to sapiens SFRS protein kinase 1 (SRPK1), mRNA
3082	Q96SB4	5	7	10.1	655	74325	6.2	Serine/threonine-protein kinase SRPK1
3082	UPI0001D3B9F9	5	7	9.8	671	76040	6.3	Serine/threonine-protein kinase SRPK1 (EC 2.7.11.1) (Serine/arginine- rich protein-specific kinase 1) (SR-protein-specific kinase 1) (SFRS protein kinase 1).
3083	B4DEK5	5	45	12.2	452	50651	5	cDNA FLJ54596, highly similar to Proactivator polypeptide
3083	Q53FJ5	5	45	10.5	524	58141	5.2	Prosaposin (Variant Gaucher disease and variant metachromatic leukodystrophy) variant (Fragment)
3083	P07602-2	5	45	10.5	526	58356	5.1	Isoform Sap-mu-6 of Proactivator polypeptide
3083	P07602	5	45	10.5	524	58113	5.2	Proactivator polypeptide
3083	C9JIZ6	5	45	10.5	526	58354	5.2	Uncharacterized protein
3083	Q59EN5	5	45	10.4	530	58727	5.1	Prosaposin variant (Fragment)
3083	C9J7I6	5	45	10.4	527	58400	5.1	Uncharacterized protein
3083	B1AVU8	5	45	9.8	559	61693	5.2	Prosaposin
3084	E9PCV0	5	8	12.3	505	58345	7	Uncharacterized protein
3084	B4E1F6	5	8	12.3	505	58329	7	cDNA FLJ53268, highly similar to Beta-glucuronidase (EC 3.2.1.31)
3084	P08236-2	5	8	10.3	600	69142	7	Isoform Short of Beta-glucuronidase
3084	B2R6X2	5	8	9.5	651	74706	7	cDNA, FLJ93161, highly similar to sapiens glucuronidase, beta (GUSB), mRNA
3084	P08236	5	8	9.5	651	74732	7	Beta-glucuronidase
3085	Q7Z417	5	9	9.4	695	76121	8.7	Nuclear fragile X mental retardation-interacting protein 2
3086	O75175-2	5	10	10.7	609	64053	5.9	Isoform 2 of CCR4-NOT transcription complex subunit 3
3086	B7Z6J7	5	10	9.1	718	76569	6.4	cDNA FLJ61662, highly similar to CCR4-NOT transcription complex subunit 3
3086	O75175	5	10	8.6	753	81872	6.2	CCR4-NOT transcription complex subunit 3
3087	B8K2L9	5	11	9	630	67281	7	Zinc finger protein 185 variant 630
3087	B8K2M0	5	11	8.6	660	70380	7	Zinc finger protein 185 variant 660
3087	O15231-3	5	11	8.3	690	73613	7	Isoform 3 of Zinc finger protein 185
3087	O15231	5	11	8.3	689	73525	7	Zinc finger protein 185
3087	UPI0001D27F7F	5	11	8.2	692	73711	7	zinc finger protein 185 isoform 2
3087	UPI0001D27F7E	5	11	7.9	721	76857	7	zinc finger protein 185 isoform 1
3088	C9JEA8	5	76	8.8	419	46659	5.7	Uncharacterized protein
3089	Q9Y5A7-2	5	7	9	601	69119	6	Isoform 2 of NEDD8 ultimate buster 1
3089	Q9Y5A7	5	7	8.8	615	70538	6	NEDD8 ultimate buster 1
3090	Q13546-2	5	7	9.3	625	70733	6.2	Isoform 2 of Receptor-interacting serine/threonine-protein kinase 1
3090	Q13546	5	7	8.6	671	75931	6.3	Receptor-interacting serine/threonine-protein kinase 1
3091	P12109	5	9	7.9	1028	108529	5.4	Collagen alpha-1(VI) chain
3092	P02768	5	79	7.7	609	69367	6.3	Serum albumin
3092	B2RBS8	5	79	7.7	609	69393	6.3	cDNA, FLJ95666, highly similar to sapiens albumin (ALB), mRNA
3092	A8K9P0	5	79	7.7	608	69268	6.3	cDNA FLJ78413, highly similar to sapiens albumin, mRNA
3092	B4DPP6	5	79	7.6	618	70363	6.1	cDNA FLJ54371, highly similar to Serum albumin
3092	A6NBZ8	5	79	7.5	627	71705	6.7	Uncharacterized protein
3093	B2RTX8	5	6	7.3	1184	132226	5.5	WAPAL protein
3093	Q7Z5K2	5	6	7.2	1190	132945	5.4	Wings apart-like protein log
3093	E9PH12	5	6	7	1228	137411	5.9	Uncharacterized protein
3094	O60645-2	5	8	8.6	641	73797	7.1	Isoform 2 of Exocyst complex component 3
3094	O60645	5	8	7.3	756	86845	6.1	Exocyst complex component 3
3095	B4DWF8	5	7	10.1	666	70943	6.4	cDNA FLJ53414, highly similar to Nuclear pore complex protein Nup98-Nup96precursor
3095	P52948-4	5	7	7.3	920	96074	7.6	Isoform 4 of Nuclear pore complex protein Nup98-Nup96
3095	A8KA17	5	7	7.2	937	97952	7.4	cDNA FLJ77211, highly similar to sapiens nucleoporin 98kDa (NUP98), transcript variant 3, mRNA
3095	P52948-3	5	7	7.2	937	97836	7.9	Isoform 3 of Nuclear pore complex protein Nup98-Nup96
3095	UPI0001AE6BA5	5	7	3.9	1729	187666	6.4	Nuclear pore complex protein Nup98-Nup96 precursor [Contains: Nuclear pore complex protein Nup98 (Nucleoporin Nup98) (98 kDa nucleoporin); Nuclear pore complex protein Nup96 (Nucleoporin Nup96) (96 kDa nucleoporin)].
3095	P52948-6	5	7	3.9	1714	186267	6.3	Isoform 6 of Nuclear pore complex protein Nup98-Nup96
3095	P52948-2	5	7	3.9	1726	187198	6.5	Isoform 2 of Nuclear pore complex protein Nup98-Nup96
3095	P52948-5	5	7	3.7	1800	195816	6.4	Isoform 5 of Nuclear pore complex protein Nup98-Nup96
3095	P52948	5	7	3.7	1817	197578	6.4	Nuclear pore complex protein Nup98-Nup96
3096	E9PBB0	5	12	7.3	1293	149486	5.5	Uncharacterized protein
3096	B9EG55	5	12	7.2	1298	149942	5.5	GRB10 interacting GYF protein 2
3096	Q6Y7W6	5	12	7.2	1299	150070	5.5	PERQ amino acid-rich with GYF domain-containing protein 2
3096	Q6Y7W6-3	5	12	7.1	1321	152528	5.6	Isoform 2 of PERQ amino acid-rich with GYF domain-containing protein 2
3097	Q5R372-4	5	5	7.2	800	90838	5.6	Isoform 4 of Rab GTPase-activating protein 1-like
3097	Q5R372	5	5	7.1	815	92513	5.3	Rab GTPase-activating protein 1-like
3097	UPI000179A973	5	5	7.1	815	92545	5.3	RAB GTPase activating protein 1-like isoform A
3097	Q5R372-3	5	5	7	827	94002	5.3	Isoform 3 of Rab GTPase-activating protein 1-like
3098	B4E205	5	22	7	857	96273	7.9	cDNA FLJ61651, highly similar to Protein transport protein Sec24A
3098	O95486	5	22	5.5	1093	119749	7.7	Protein transport protein Sec24A
3099	B3KW08	5	13	6.8	1111	127423	6.4	cDNA FLJ41852 fis, clone NT2RI3004133, highly similar to Exportin-6
3099	Q96QU8	5	13	6.8	1125	128883	6.4	Exportin-6
3099	B7ZM10	5	13	6.8	1124	128789	6.3	XPO6 protein
3100	Q71UH4	5	7	6.9	1598	180613	7.9	DNA topoisomerase 2 (Fragment)
3100	Q02880	5	7	6.8	1626	183266	8	DNA topoisomerase 2-beta
3100	Q02880-2	5	7	6.8	1621	182661	8.1	Isoform Beta-1 of DNA topoisomerase 2-beta
3101	A8K4A1	5	11	6.6	963	108155	6.4	cDNA FLJ76790
3101	Q96HW7	5	11	6.6	963	108171	6.4	Integrator complex subunit 4
3102	B3KM89	5	6	7.4	906	100249	6.9	cDNA FLJ10528 fis, clone NT2RP2000943, highly similar to Protein transport protein Sec24D
3102	A8K6V0	5	6	6.5	1032	112962	7.2	cDNA FLJ78676, highly similar to sapiens SEC24 related gene family, member D (SEC24D), mRNA
3102	O94855-2	5	6	6.5	1033	113081	7.2	Isoform 2 of Protein transport protein Sec24D
3102	O94855	5	6	6.5	1032	113010	7.2	Protein transport protein Sec24D
3103	Q5M775-4	5	24	6.3	987	110242	5.9	Isoform 4 of Cytospin-B
3103	B4E2A4	5	24	6.3	987	110293	6	cDNA FLJ53275, highly similar to sapiens spectrin domain with coiled-coils 1 (SPECC1), transcript variant, mRNA
3103	A8MV89	5	24	6	1029	114178	6.9	Uncharacterized protein
3103	Q5M775	5	24	5.8	1068	118585	6.7	Cytospin-B
3104	Q86YS7	5	6	5.9	1000	110447	5.7	Uncharacterized protein KIAA0528
3104	Q86YS7-2	5	6	5.7	1042	114994	6.1	Isoform 2 of Uncharacterized protein KIAA0528
3104	B7ZLK2	5	6	5.7	1042	115022	6.1	KIAA0528 protein
3104	B4DRN7	5	6	5.6	1051	116027	5.9	cDNA FLJ54447
3104	B7ZLL0	5	6	5.6	1053	116339	6.4	KIAA0528 protein
3104	B7ZLK1	5	6	5.6	1051	116055	5.9	KIAA0528 protein

Table S1. Proteins identified in the OVCAR-3 tumor supernatant by two or more unique peptides.

Protein or Protein Group	Accession Number	Unique Peptide Count	Spectrum Count	Sequence Coverage (%)	Length	MolWt	pI	Descriptive Name
3105	Q96P48	5	6	5.5	1450	162191	6.2	Arf-GAP with Rho-GAP domain, ANK repeat and PH domain-containing protein 1
3106	Q96DB6	5	6	19	378	41809	9.1	PDS5A protein (Fragment)
3106	UPI000179A8F6	5	6	5.6	1296	146528	7.9	PDS5, regulator of cohesion maintenance, log A isoform 1
3106	Q29RF7	5	6	5.4	1337	150830	7.9	Sister chromatid cohesion protein PDS5 log A
3107	Q9NRY4	5	5	5.4	1513	172228	6.8	Glucocorticoid receptor DNA-binding factor 1
3107	Q9NRY4-2	5	5	5.4	1499	170513	6.6	Isoform 2 of Glucocorticoid receptor DNA-binding factor 1
3108	E9PG28	5	5	7.8	1025	113273	5.7	Uncharacterized protein
3108	UPI00015E0ADB	5	5	5.7	1401	152954	5.4	Obscurin-like protein 1 precursor.
3108	O75147-1	5	5	5.7	1401	152924	5.4	Isoform 3 of Obscurin-like protein 1
3108	A4KVA4	5	5	5.2	1544	168577	5.4	Obscurin-like 1 isoform C
3108	UPI0001CB27C5	5	5	5.2	1543	168520	5.4	obscurin-like protein 1 isoform 2 precursor
3108	A6NN50	5	5	4.4	1804	197099	5.6	Uncharacterized protein
3108	O75147	5	5	4.2	1896	206945	5.6	Obscurin-like protein 1
3109	B3KU09	5	8	4.9	1314	148460	6.7	cDNA FLJ39034 fis, clone NT2RP7008085, highly similar to sapiens ring finger protein 123 (RNF123), mRNA
3109	Q5XPI4-2	5	8	4.9	1316	148958	6.8	Isoform 2 of E3 ubiquitin-protein ligase RNF123
3109	Q5XPI4	5	8	4.9	1314	148514	6.7	E3 ubiquitin-protein ligase RNF123
3110	Q6P158	5	7	4.8	1386	155604	7.7	Putative ATP-dependent RNA helicase DHX57
3111	B7Z897	5	5	4.6	1113	124015	6.2	cDNA FLJ50429, highly similar to Hamartin
3111	UPI0001A7BA83	5	5	4.4	1163	129695	6.5	hamartin isoform 3
3111	Q92574	5	5	4.4	1164	129766	6.5	Hamartin
3112	Q9NS87	5	7	4.6	1388	160159	6	Kinesin-like protein KIF15
3113	Q9Y4B6	5	5	4.5	1507	169007	5.1	Protein VPRBP
3113	UPI0001AE75C2	5	5	4.5	1507	169000	5	Protein VPRBP (HIV-1 Vpr-binding protein) (VprBP) (Vpr-interacting protein) (DDB1- and CUL4-associated factor 1).
3113	Q9Y4B6-2	5	5	4.5	1506	168936	5.1	Isoform 2 of Protein VPRBP
3114	A9JR72	5	10	4.1	1711	194286	6.4	CDC42 binding protein kinase beta (DMPK-like)
3114	Q9Y5S2	5	10	4.1	1711	194314	6.4	Serine/threonine-protein kinase MRCK beta
3115	A2RUU9	5	6	3.7	1859	209905	5	PCM1 protein
3115	E7EV56	5	6	3.7	1860	210131	5	Uncharacterized protein
3115	Q15154-2	5	6	3.5	1969	222220	5	Isoform 2 of Pericentriolar material 1 protein
3115	A6NNN6	5	6	3.5	1969	222247	5	Uncharacterized protein
3115	UPI0000210A25	5	6	3.4	2024	228558	5	Pericentriolar material 1 protein (PCM-1) (hPCM-1).
3115	Q15154	5	6	3.4	2024	228531	5	Pericentriolar material 1 protein
3115	E7ETA6	5	6	3.4	2016	227729	5	Uncharacterized protein
3115	D3DSQ0	5	6	3.4	2024	228526	5	Pericentriolar material 1, isoform CRA_a
3115	B9EIS5	5	6	3.4	2016	227670	5	PCM1 protein
3116	Q5D862	5	5	3.1	2391	248072	8.3	Filaggrin-2
3117	Q96Q15	5	9	1.8	3661	410501	6.5	Serine/threonine-protein kinase SMG1
3117	UPI00015DFBDF	5	9	1.8	3662	410630	6.5	Serine/threonine-protein kinase SMG1 (EC 2.7.11.1) (SMG-1) (hSMG-1) (Lambda/iota protein kinase C-interacting protein) (Lambda-interacting protein) (61E3.4).
3118	C9J4Z3	4	118	69.1	68	7624	10	Ribosomal protein L37a, isoform CRA_c
3118	Q5M9N6	4	118	57.3	82	8995	10.4	Rpl37a protein
3118	P61513	4	118	51.1	92	10275	10.4	60S ribosomal protein L37a 60S ribosomal protein L37a
3119	P63167	4	88	67.4	89	10366	7.4	Dynein light chain 1, cytoplasmic Dynein light chain 1, cytoplasmic
3120	P49459	4	22	56.6	152	17315	5.1	Ubiquitin-conjugating enzyme E2 A Ubiquitin-conjugating enzyme E2 A
3120	Q53G67	4	22	56.6	152	17287	5.1	Ubiquitin carrier protein (Fragment)
3121	A6NMQ7	4	30	51.1	137	15614	4.6	Ubiquitin carrier protein Ubiquitin carrier protein
3121	P60604	4	30	42.4	165	18566	4.7	Ubiquitin-conjugating enzyme E2 G2 Ubiquitin-conjugating enzyme E2 G2
3121	E9QMQ4	4	30	23.3	301	32793	4.9	Uncharacterized protein
3121	Q3UWG7	4	30	23.1	303	32990	4.9	Ubiquitin carrier protein
3122	P35268	4	105	50.8	128	14787	9.2	60S ribosomal protein L22
3122	Q7Z4W8	4	105	50.8	128	14818	9.3	Heparin-binding protein HBp15
3122	P67984	4	105	50.8	128	14759	9.2	60S ribosomal protein L22
3123	Q9DAM7	4	6	49.6	115	11549	9.3	UPF0444 transmembrane protein C12orf23 log
3123	Q8WUH6	4	6	49.1	116	11748	9.3	UPF0444 transmembrane protein C12orf23
3124	O15116	4	11	42.1	133	15179	5.2	U6 snRNA-associated Sm-like protein LSm1
3124	Q8VC85	4	11	42.1	133	15236	5.4	U6 snRNA-associated Sm-like protein LSm1
3125	Q7RTV0	4	8	40	110	12405	8.4	PHD finger-like domain-containing protein 5A PHD finger-like domain-containing protein 5A
3126	P42677	4	86	39.3	84	9461	9.5	40S ribosomal protein S27 40S ribosomal protein S27
3127	P63272	4	52	38.5	117	13193	8.1	Transcription elongation factor SPT4 Transcription elongation factor SPT4
3127	Q9Z199	4	52	38.5	117	13194	8.1	Transcription elongation factor SPT4 2
3128	E9PI02	4	5	38.1	139	16147	9.6	Uncharacterized protein
3128	E9PMQ6	4	5	12.3	430	46537	6.8	Uncharacterized protein
3128	Q52L52	4	5	11.1	477	51704	6.6	Hsf1 protein
3128	Q00613-2	4	5	10.8	489	52881	6.1	Isoform Short of Heat shock factor protein 1
3128	P38532-2	4	5	10.5	503	54931	5.5	Isoform 1 of Heat shock factor protein 1
3128	P38532	4	5	10.1	525	57224	5.3	Heat shock factor protein 1
3128	Q53HT3	4	5	10	529	57228	5.2	Heat shock transcription factor 1 variant (Fragment)
3128	Q00613	4	5	10	529	57261	5.2	Heat shock factor protein 1
3129	P62879	4	16	37.4	340	37331	6	Guanine nucleotide-binding protein G(I)/G(S)/G(T) subunit beta-2 Guanine nucleotide-binding protein G(I)/G(S)/G(T) subunit beta-2
3130	Q3MHD2	4	18	36.4	195	21701	7.8	Protein LSM12 log Protein LSM12 log
3130	Q3MHD2-2	4	18	35.9	198	21999	6.9	Isoform 2 of Protein LSM12 log
3131	Q8WVJ2	4	19	35.7	157	17676	5.1	NudC domain-containing protein 2
3131	Q9CQ48	4	19	35.7	157	17660	5.1	NudC domain-containing protein 2
3132	Q3UZI5	4	7	36.5	156	17414	9.6	Pleiotrophin, isoform CRA_b
3132	C9JR52	4	7	34.3	166	18877	9.7	Uncharacterized protein
3132	P63089	4	7	33.9	168	18869	9.6	Pleiotrophin
3132	P21246	4	7	33.9	168	18942	9.6	Pleiotrophin
3133	Q5JQ44	4	10	30.9	181	20294	4.8	Putative uncharacterized protein DKFZp547A0616 (Fragment)
3133	P61225	4	10	30.6	183	20504	4.8	Ras-related protein Rap-2b Ras-related protein Rap-2b
3134	Q5PR09	4	27	33.3	135	15874	11.4	Ribosomal protein L32
3134	P62910	4	27	33.3	135	15860	11.3	60S ribosomal protein L32 60S ribosomal protein L32
3134	C9JBL2	4	27	29.4	153	17962	10.6	Uncharacterized protein
3135	B4DP27	4	8	29	169	19259	5.9	cDNA FLJ52153, highly similar to Transmembrane emp24 domain-containing protein 2
3135	E7ESD4	4	8	29	169	19239	6.4	Uncharacterized protein Uncharacterized protein
3135	UPI00015AA329	4	8	27.8	176	20027	9.1	UPI00015AA329 UniRef100 entry
3135	P49020	4	8	25	196	22189	5.2	Transmembrane emp24 domain-containing protein 2 (Fragment)
3135	Q9R0Q3	4	8	24.4	201	22705	5.2	Transmembrane emp24 domain-containing protein 2
3135	Q15363	4	8	24.4	201	22761	5.2	Transmembrane emp24 domain-containing protein 2
3136	A8MSV4	4	4	28.7	174	19671	5.4	Uncharacterized protein
3136	Q969Q5	4	4	24.6	203	23124	6.2	Ras-related protein Rab-24
3136	Q8K133	4	4	24.6	203	23118	6.4	Rab24 protein
3136	P35290	4	4	24.6	203	23144	6.2	Ras-related protein Rab-24

Table S1. Proteins identified in the OVCAR-3 tumor supernatant by two or more unique peptides.

Protein or Protein Group	Accession Number	Unique Peptide Count	Spectrum Count	Sequence Coverage (%)	Length	MolWt	pI	Descriptive Name
3137	Q96H20	4	9	28.7	258	28864	6.7	Vacuolar-sorting protein SNF8
3137	Q9CZ28	4	9	28.7	258	28886	6.7	Vacuolar-sorting protein SNF8
3138	UPI0000605FCF	4	9	28.1	128	14140	8.5	PREDICTED: NHP2-like protein 1-like
3138	P55769	4	9	28.1	128	14174	8.5	NHP2-like protein 1 NHP2-like protein 1
3138	E9PWL3	4	9	28.1	128	14186	8.5	Uncharacterized protein
3138	B1AHD1	4	9	27.3	132	14627	8.5	NHP2 non-histone chromosome protein 2-like 1 (S. cerevisiae)
3139	D3Z6H3	4	10	27	185	20041	6.4	Dynactin 6, isoform CRA_b
3139	Q9WUB4	4	10	26.3	190	20670	6.7	Dynactin subunit 6
3139	O00399	4	10	26.3	190	20747	6.3	Dynactin subunit 6
3140	Q9H1R6	4	19	33	188	21029	4.7	BCL2-like 1 (Fragment)
3140	Q5TE64	4	19	30.2	205	22896	4.7	BCL2-like 1 (Fragment)
3140	Q64373-3	4	19	29.7	209	23357	4.8	Isoform Bcl-X(beta) of Bcl-2-like protein 1
3140	Q64373-4	4	19	29	214	23900	4.7	Isoform Bcl-X(delta-TM) of Bcl-2-like protein 1
3140	Q07817-3	4	19	27.3	227	25290	4.8	Isoform Bcl-X(beta) of Bcl-2-like protein 1
3140	Q64373	4	19	26.6	233	26132	4.9	Bcl-2-like protein 1
3140	Q07817	4	19	26.6	233	26049	4.9	Bcl-2-like protein 1
3140	O35843	4	19	26.4	235	26122	4.7	Bcl-x-gamma
3141	P33947	4	11	25.9	212	24422	8.7	ER lumen protein retaining receptor 2
3141	Q9CQM2	4	11	25.9	212	24454	8.7	ER lumen protein retaining receptor 2
3142	Q9BSB4	4	6	25.7	218	25003	6.1	Autophagy-related protein 101
3142	Q9D8Z6	4	6	25.7	218	25002	6.1	Autophagy-related protein 101
3143	P83881	4	11	24.5	106	12441	10.6	60S ribosomal protein L36a 60S ribosomal protein L36a
3143	B2REA7	4	11	23.2	112	13228	10.5	Ribosomal protein L36a
3143	C9JBJ2	4	11	22	118	13792	10.5	Uncharacterized protein
3143	UPI0001F169B7	4	11	18.3	142	16378	10.4	60S ribosomal protein L36a isoform a
3144	Q17S11	4	12	21.8	376	43158	5.7	SWI/SNF related, matrix associated, actin dependent regulator of chromatin, subfamily b, member 1 SWI/SNF related, matrix associated, actin dependent regulator of chromatin, subfamily b, member 1
3144	Q12824	4	12	21.3	385	44141	6.2	SWI/SNF-related matrix-associated actin-dependent regulator of chromatin subfamily B member 1 SWI/SNF-related matrix-associated actin-dependent regulator of chromatin subfamily B member 1
3144	UPI000013D3AE	4	12	20.8	394	45050	5.8	SWI/SNF-related matrix-associated actin-dependent regulator of chromatin subfamily B member 1 (Integrase interactor 1 protein) (hSNF5) (BAF47).
3144	Q9H836	4	12	20.8	394	45051	5.6	cDNA FLJ13963 fis, clone Y79AA1001299, highly similar to sapiens integrase interactor 1b protein (INI1B)
3145	Q2NLD4	4	7	23.5	285	32015	7	PURA protein (Fragment)
3145	Q2NLC9	4	7	22.3	300	34166	9.2	PURA protein (Fragment)
3145	P42669	4	7	20.9	321	34884	6.4	Transcriptional activator protein Pur-alpha
3145	Q8C6E9	4	7	20.9	321	34982	7.1	Putative uncharacterized protein
3145	Q56A79	4	7	20.8	322	34939	6.7	Purine-rich element binding protein A
3145	Q00577	4	7	20.8	322	34911	6.4	Transcriptional activator protein Pur-alpha
3146	B3KUY1	4	49	20.6	209	24190	11.9	Splicing factor, arginine/serine-rich 2, isoform CRA_d
3146	B4DN89	4	49	20.6	209	24153	11.9	Splicing factor, arginine/serine-rich 2, isoform CRA_g
3146	Q53FN0	4	49	19.5	221	25502	11.9	Splicing factor, arginine/serine-rich 2 variant (Fragment)
3146	Q01130	4	49	19.5	221	25476	11.9	Serine/arginine-rich splicing factor 2 Serine/arginine-rich splicing factor 2
3146	Q8C671	4	49	16.9	254	29001	11.2	Putative uncharacterized protein
3146	E9Q975	4	49	16.9	254	28918	11.4	Uncharacterized protein
3147	Q3UXV4	4	5	21.9	452	51446	5.8	Serine/threonine-protein phosphatase
3147	Q5F2F8	4	5	20	496	56015	5.7	Serine/threonine-protein phosphatase
3147	E9Q6P2	4	5	19.9	497	56098	5.7	Uncharacterized protein
3147	P16298-3	4	5	19.3	514	57982	6.1	Isoform 3 of Serine/threonine-protein phosphatase 2B catalytic subunit beta isoform
3147	P16298-2	4	5	19.3	514	58013	5.6	Isoform 2 of Serine/threonine-protein phosphatase 2B catalytic subunit beta isoform
3147	UPI000154C8F1	4	5	19.2	515	58131	6.1	protein phosphatase 3, catalytic subunit, beta isoform
3147	E0CZ78	4	5	18.9	524	59074	5.9	Serine/threonine-protein phosphatase
3147	Q8N1F0	4	5	18.9	525	59123	5.9	Serine/threonine-protein phosphatase
3147	P48453-2	4	5	18.9	524	58929	5.9	Isoform 1 of Serine/threonine-protein phosphatase 2B catalytic subunit beta isoform
3147	P48453	4	5	18.9	525	59174	5.9	Serine/threonine-protein phosphatase 2B catalytic subunit beta isoform
3147	P16298	4	5	18.9	524	59024	5.9	Serine/threonine-protein phosphatase 2B catalytic subunit beta isoform
3148	Q969T9	4	14	24.9	261	28087	5.9	WW domain-binding protein 2
3148	Q3TRG5	4	14	24.9	261	28079	6.5	Putative uncharacterized protein
3148	P97765	4	14	24.9	261	28032	6.3	WW domain-binding protein 2
3148	E9Q1S7	4	14	18.8	345	36943	9.3	Uncharacterized protein
3148	Q9DBP6	4	14	18.7	348	37194	9.3	Putative uncharacterized protein
3149	UPI000049E0CA	4	10	17.5	229	24770	7.9	UPI000049E0CA UniRef100 entry
3149	Q96CG8	4	10	16.5	243	26224	8	Collagen triple helix repeat-containing protein 1
3149	A2RRY6	4	10	16.3	245	26434	7.5	Collagen triple helix repeat containing 1
3149	Q9D1D6	4	10	16.3	245	26460	7.1	Collagen triple helix repeat-containing protein 1
3149	Q3UAP6	4	10	16.3	245	26460	7.5	MCG15508
3150	Q9JKC8	4	4	16	418	46936	6.9	AP-3 complex subunit mu-1
3150	Q9Y2T2	4	4	16	418	46939	6.9	AP-3 complex subunit mu-1
3151	C9JB13	4	13	15.6	422	46160	4.7	Uncharacterized protein
3151	Q80Y55	4	13	15.5	427	46953	4.4	BSD domain-containing protein 1
3151	Q9NW68	4	13	15.3	430	47163	4.5	BSD domain-containing protein 1
3152	B7Z6Y2	4	8	15.1	385	43165	5.3	cDNA FLJ54942, highly similar to sapiens bridging integrator 1 (BIN1), transcript variant 10, mRNA
3152	O00499-9	4	8	14.2	409	45563	5.5	Isoform BIN1-10-13 of Myc box-dependent-interacting protein 1
3152	O00499-10	4	8	13.7	424	47491	6.6	Isoform BIN1-13 of Myc box-dependent-interacting protein 1
3152	O08539-2	4	8	13.4	434	48014	5.2	Isoform 2 of Myc box-dependent-interacting protein 1
3152	O00499-7	4	8	13.2	439	48258	5.2	Isoform II3 of Myc box-dependent-interacting protein 1
3152	O00499-8	4	8	12.8	454	50185	5.9	Isoform BIN1 of Myc box-dependent-interacting protein 1
3152	Q6P1B9	4	8	12.2	477	52764	5.2	Bin1 protein
3152	O00499-4	4	8	12.2	475	51737	5	Isoform IIC2 of Myc box-dependent-interacting protein 1
3152	O00499-6	4	8	12	482	53020	5.3	Isoform II2 of Myc box-dependent-interacting protein 1
3152	Q8WWH9	4	8	11.8	490	53167	5.1	Amphiphysin IIb-1
3152	O00499-11	4	8	11.7	497	54948	6	Isoform BIN1+12A of Myc box-dependent-interacting protein 1
3152	O00499-3	4	8	11.5	506	55175	5.2	Isoform IIC1 of Myc box-dependent-interacting protein 1
3152	O00499-2	4	8	11.2	518	56499	5.1	Isoform IIB of Myc box-dependent-interacting protein 1
3152	O00499-5	4	8	10.5	550	59937	5	Isoform IID of Myc box-dependent-interacting protein 1
3152	O08539	4	8	9.9	588	64470	5	Myc box-dependent-interacting protein 1
3152	O00499	4	8	9.8	593	64699	5.1	Myc box-dependent-interacting protein 1
3153	Q9UHR5-2	4	7	15.8	292	32069	5.1	Isoform 2 of SAP30-binding protein
3153	Q02614	4	7	14.9	308	33832	4.9	SAP30-binding protein
3153	Q9UHR5	4	7	14.9	308	33870	4.8	SAP30-binding protein
3154	P25490	4	10	14.7	414	44713	6.3	Transcriptional repressor protein YY1

Table S1. Proteins identified in the OVCAR-3 tumor supernatant by two or more unique peptides.

Protein or Protein Group	Accession Number	Unique Peptide Count	Spectrum Count	Sequence Coverage (%)	Length	MolWt	pI	Descriptive Name
3154	Q00899	4	10	14.7	414	44717	6.3	Transcriptional repressor protein YY1
3155	B4DSZ6	4	7	14.3	322	36171	8.4	cDNA FLJ50654, highly similar to Isocitrate dehydrogenase
3155	Q53GL5	4	7	10.2	452	50910	8.5	Isocitrate dehydrogenase [NADP] (Fragment)
3155	P54071	4	7	10.2	452	50906	8.7	Isocitrate dehydrogenase [NADP], mitochondrial
3155	P48735	4	7	10.2	452	50909	8.7	Isocitrate dehydrogenase [NADP], mitochondrial
3156	E9PI99	4	14	32.6	141	16203	7.3	Uncharacterized protein
3156	Q5T6K6	4	14	18.1	254	28450	5.7	Nuclear transcription factor Y, gamma (Fragment)
3156	Q7TPS7	4	14	16.7	275	30647	5.7	Nfyc protein
3156	Q5T6K9	4	14	15.3	301	33702	5.1	Nuclear transcription factor Y, gamma
3156	Q13952-2	4	14	13.7	335	37194	5	Isoform 1 of Nuclear transcription factor Y subunit gamma
3156	P70353	4	14	13.7	335	37254	5	Nuclear transcription factor Y subunit gamma
3156	B4DUS6	4	14	13	354	39346	5.5	cDNA FLJ58328, highly similar to Nuclear transcription factor Y subunit gamma
3156	Q59GY4	4	14	12.2	378	41595	6.1	Nuclear transcription factor Y, gamma variant (Fragment)
3156	E9PF96	4	14	12.1	380	41379	8.1	Uncharacterized protein
3156	Q13952-3	4	14	10.5	439	48150	5.4	Isoform 2 of Nuclear transcription factor Y subunit gamma
3156	Q13952	4	14	10	458	50302	6.1	Nuclear transcription factor Y subunit gamma
3157	Q0QEN7	4	5	15.3	445	48113	5.1	ATP synthase subunit beta (Fragment)
3157	P06576	4	5	12.9	529	56560	5.4	ATP synthase subunit beta, mitochondrial
3157	P56480	4	5	12.9	529	56301	5.3	ATP synthase subunit beta, mitochondrial
3158	Q3TJZ6	4	8	12.6	515	55055	9	Protein FAM98A
3158	Q8NCA5-2	4	8	12.5	518	55272	9	Isoform 2 of Protein FAM98A
3158	Q8NCA5	4	8	12.5	519	55401	9	Protein FAM98A
3159	B4DDI6	4	7	11.5	461	50734	5.1	cDNA FLJ52585
3159	B4DVT3	4	7	11	482	53588	5.4	cDNA FLJ50705
3159	Q8R3P6	4	7	10.3	515	57237	5.2	UPF0464 protein C15orf44 log
3159	Q96SY0	4	7	10.2	518	57471	5.1	UPF0464 protein C15orf44
3160	P98083-2	4	6	12.8	469	51409	7.1	Isoform p52Shc of SHC-transforming protein 1
3160	P29353-7	4	6	12.7	474	51682	7.2	Isoform 7 of SHC-transforming protein 1
3160	P29353-2	4	6	12.7	473	51611	7.2	Isoform p52Shc of SHC-transforming protein 1
3160	P98083	4	6	10.4	579	62608	6.5	SHC-transforming protein 1
3160	P29353	4	6	10.3	583	62822	6.4	SHC-transforming protein 1
3160	P29353-6	4	6	10.3	584	62893	6.4	Isoform 6 of SHC-transforming protein 1
3161	E7ETD9	4	4	34.3	181	20857	9.8	Uncharacterized protein
3161	A1L366	4	4	9.8	635	72057	4.6	RAD21 log (S. pombe)
3161	Q810A8	4	4	9.8	635	72083	4.6	Putative uncharacterized protein
3161	Q61550	4	4	9.8	635	72023	4.6	Double-strand-break repair protein rad21 log
3161	O60216	4	4	9.8	631	71690	4.7	Double-strand-break repair protein rad21 log
3162	Q15124-2	4	9	13.7	387	41790	6.6	Isoform 2 of Phosphoglucomutase-like protein 5
3162	Q15124	4	9	9.3	567	62225	7.2	Phosphoglucomutase-like protein 5
3162	Q8BZF8	4	9	9.3	567	62220	7.2	Phosphoglucomutase-like protein 5
3163	B7Z9H9	4	8	9.8	551	62702	5.6	cDNA, FLJ78843, highly similar to Guanylate cyclase soluble subunit beta-1 (EC4.6.1.2)
3163	B7Z685	4	8	9.8	551	62762	5.6	Guanylate cyclase 1, soluble, beta 3, isoform CRA_c
3163	Q02153-2	4	8	9.2	586	66738	5.2	Isoform HSGC-2 of Guanylate cyclase soluble subunit beta-1
3163	D6RC99	4	8	9.1	594	67519	5.9	Uncharacterized protein
3163	B7Z426	4	8	9	599	68165	5.4	cDNA FLJ59711, highly similar to Guanylate cyclase soluble subunit beta-1 (EC 4.6.1.2)
3163	Q80YP4	4	8	8.9	609	68995	5.5	Gucy1b3 protein
3163	B2RCP1	4	8	8.7	619	70487	5.4	cDNA, FLJ96185, highly similar to sapiens guanylate cyclase 1, soluble, beta 3 (GUCY1B3), mRNA
3163	Q02153	4	8	8.7	619	70515	5.4	Guanylate cyclase soluble subunit beta-1
3163	O54865	4	8	8.7	620	70598	5.3	Guanylate cyclase soluble subunit beta-1
3164	A8K2A8	4	21	7.5	963	108181	5.2	cDNA FLJ77274, highly similar to sapiens importin 13 (IPO13), mRNA
3164	Q8K0C1	4	21	7.5	963	108229	5.3	Importin-13
3164	Q3UDQ3	4	21	7.5	963	108171	5.3	Putative uncharacterized protein
3164	O94829	4	21	7.5	963	108195	5.3	Importin-13
3165	Q80UY7	4	6	23	274	31070	7	Scyl2 protein (Fragment)
3165	Q6P3W7	4	6	6.8	929	103709	8.2	SCY1-like protein 2
3165	Q8CFE4	4	6	6.8	930	103317	8	SCY1-like protein 2
3166	Q3UYZ8	4	6	7.9	598	68597	8.3	Putative uncharacterized protein (Fragment)
3166	Q3USR8	4	6	7.9	596	68350	8.1	Putative uncharacterized protein (Fragment)
3166	P49711	4	6	6.5	727	82785	7	Transcriptional repressor CTCF
3166	Q61164	4	6	6.4	736	83746	6.9	Transcriptional repressor CTCF
3167	B2RBE6	4	4	5.4	924	104081	7	cDNA, FLJ95470, highly similar to sapiens log of yeast Sec5 (SEC5), mRNA
3167	Q9D4H1	4	4	5.4	924	103959	7.2	Exocyst complex component 2
3167	Q96KP1	4	4	5.4	924	104066	6.9	Exocyst complex component 2
3167	B3KPN7	4	4	5.4	924	104124	6.8	cDNA FLJ32002 fis, clone NT2RP7009394, highly similar to Exocyst complex component 2
3168	Q149N2	4	8	6.6	1106	127529	7.9	DOCK4 protein
3168	Q8BMP2	4	8	6.4	1134	131167	7.1	Putative uncharacterized protein
3168	Q8N1I0-2	4	8	3.8	1937	222216	7.6	Isoform 2 of Dedicator of cytokinesis protein 4
3168	E9PXU8	4	8	3.8	1940	222587	7.6	Uncharacterized protein
3168	B2RUG6	4	8	3.8	1936	222195	7.6	Dock4 protein
3168	A4D0S8	4	8	3.7	1966	225219	7.6	Dedicator of cytokinesis 4
3168	UPI0001F796D7	4	8	3.7	1978	226510	7.8	UPI0001F796D7 UniRef100 entry
3168	UPI000198CF9E	4	8	3.7	1999	228634	7.8	UPI000198CF9E UniRef100 entry
3168	Q8N1I0-3	4	8	3.7	1975	226176	7.6	Isoform 3 of Dedicator of cytokinesis protein 4
3168	Q8N1I0	4	8	3.7	1966	225204	7.6	Dedicator of cytokinesis protein 4
3168	Q149N6	4	8	3.7	1966	225280	7.6	Dedicator of cytokinesis 4
3168	P59764	4	8	3.7	1978	226547	7.6	Dedicator of cytokinesis protein 4
3168	Q149N5	4	8	3.6	2011	230090	7.8	DOCK4 protein
3169	Q6DIC7	4	5	3.2	2044	231369	6.9	Phosphatidylinositol 4-kinase, catalytic, alpha polypeptide
3169	P42356	4	5	3.2	2044	231317	6.9	Phosphatidylinositol 4-kinase alpha
3169	E9Q3L2	4	5	3.2	2044	231353	6.9	Uncharacterized protein
3169	B4DYG5	4	5	3.1	2102	236754	7.1	cDNA FLJ60960, highly similar to Phosphatidylinositol 4-kinase alpha (EC 2.7.1.67)
3170	Q69YP8	4	6	3.7	1698	196514	6.2	Putative uncharacterized protein DKFZp762A083 (Fragment)
3170	UPI0000F221D0	4	6	3.1	2062	237321	6.3	myosin X
3170	Q9HD67	4	6	3.1	2058	237345	6.2	Myosin-X
3170	E7EPL7	4	6	3	2069	238515	6.1	Uncharacterized protein
3171	Q6KCD5-2	4	12	2.7	2691	303762	7.8	Isoform 2 of Nipped-B-like protein
3171	Q6KC79-2	4	12	2.7	2697	304343	7.8	Isoform 2 of Nipped-B-like protein
3171	Q6IEH8	4	12	2.6	2804	316066	7.9	Transcriptional regulator
3171	Q6KCD5	4	12	2.6	2798	315450	7.9	Nipped-B-like protein
3171	Q6KC79	4	12	2.6	2804	316051	7.9	Nipped-B-like protein
3172	Q9D1L9	4	18	81.3	91	9642	4.9	Hepatitis B virus X-interacting protein homolog
3173	D3Z016	4	12	63.4	82	9205	8.6	Uncharacterized protein
3173	P0C7N9	4	12	42.3	123	13974	6.1	Proteasome assembly chaperone 4
3174	Q3TFU6	4	16	62.2	90	9417	9.6	Histone H2A
3175	Q9QUH0	4	7	57.9	107	11871	8.4	Glutaredoxin-1
3176	Q8BSL7	4	73	54.7	181	20746	6.6	ADP-ribosylation factor 2

Table S1. Proteins identified in the OVCAR-3 tumor supernatant by two or more unique peptides.

Protein or Protein Group	Accession Number	Unique Peptide Count	Spectrum Count	Sequence Coverage (%)	Length	MolWt	pI	Descriptive Name
3177	Q91VR8	4	4	54.7	75	8761	5.5	Protein BRICK1
3178	Q64522	4	32	52.3	130	14013	10.9	Histone H2A type 2-B
3179	Q9EQI5	4	8	50.4	113	12252	8.7	Chemokine (C-X-C motif) ligand 7, isoform CRA_b
3180	P08905	4	10	50	148	16689	8.8	Lysozyme C-2
3181	Q9D104	4	11	45.8	144	16095	9.9	Putative uncharacterized protein
3182	D3YXP7	4	47	43.8	121	13926	6.2	Uncharacterized protein
3183	Q9CR51	4	16	43.2	118	13724	8	V-type proton ATPase subunit G 1
3184	Q3UCX7	4	15	41.9	215	23600	7.8	Putative uncharacterized protein
3185	P97352	4	9	41.8	98	11158	6.2	Protein S100-A13
3186	Q9CQM5	4	6	41.5	123	14015	4.8	Thioredoxin domain-containing protein 17
3187	UPI0001548D14	4	19	56.1	98	11142	5.3	cytochrome b-5
3187	P56395	4	19	41	134	15241	5.1	Cytochrome b5
3188	Q9CQR2	4	20	41	83	9141	8.5	40S ribosomal protein S21
3189	P49962	4	7	39.5	86	10194	8	Signal recognition particle 9 kDa protein
3190	P31725	4	18	38.9	113	13049	7.2	Protein S100-A9
3191	D3YU66	4	125	38.5	96	10843	5.1	Uncharacterized protein
3191	UPI000002643A	4	125	37.8	98	11141	5.2	UPI000002643A UniRef100 entry
3191	P50543	4	125	37.8	98	11083	5.5	Protein S100-A11
3192	Q8R2K3	4	9	38.5	148	17157	9.8	Single-stranded DNA binding protein 1
3193	Q8R048	4	7	54.4	103	11970	5.9	C-myc binding protein
3193	Q8C5M8	4	7	54.4	103	11998	6.3	Putative uncharacterized protein
3193	A2A7K5	4	7	38.4	146	16614	9.1	C-myc binding protein
3194	Q9DAK9	4	7	37.9	124	13997	5.5	14 kDa phosphohistidine phosphatase
3195	Q64339	4	8	36	161	17898	7.9	Ubiquitin-like protein ISG15
3196	Q99LJ6	4	8	36	186	21061	8.3	Glutathione peroxidase 7
3197	A2A8E2	4	10	35.5	138	15783	5.7	Novel protein (0610037L13Rik)
3197	Q8BHG2	4	10	30.6	160	18020	5.1	UPF0587 protein C1orf123 homolog
3197	Q8BHG2-3	4	10	25	196	22048	6.5	Isoform 3 of UPF0587 protein C1orf123 homolog
3198	P97457	4	5	35.5	169	18955	4.9	Myosin regulatory light chain 2, skeletal muscle isoform
3199	E9QKE3	4	5	34.6	182	20375	5.4	Uncharacterized protein
3199	Q99PR8	4	5	34.6	182	20405	5.4	Heat shock protein beta-2
3199	Q6P3C8	4	5	34.6	182	20322	5.3	Heat shock protein 2
3200	P11031	4	24	34.6	127	14427	9.6	Activated RNA polymerase II transcriptional coactivator p15
3201	E9Q926	4	11	34.4	418	47064	6.1	Uncharacterized protein
3202	P08207	4	10	34	97	11186	6.8	Protein S100-A10
3202	Q3UF30	4	10	34	97	11213	6.8	Putative uncharacterized protein
3203	P25785	4	10	31.8	220	24328	7.5	Metalloproteinase inhibitor 2
3203	Q8BSJ3	4	10	31.8	220	24342	7.5	Putative uncharacterized protein
3203	P30121	4	10	31.8	220	24356	7.5	Metalloproteinase inhibitor 2
3204	Q8BG94	4	6	31	200	22652	5.9	COMM domain-containing protein 7
3205	P21570	4	8	29.7	145	16228	9.4	Angiogenin
3206	Q3TIF8	4	116	29.3	133	15395	10.7	40S ribosomal protein S24
3207	Q6P8I4	4	18	28.7	178	18963	7.5	PEST proteolytic signal-containing nuclear protein
3208	P05977	4	9	28.2	188	20594	5	Myosin light chain 1/3, skeletal muscle isoform
3209	E9PZ20	4	4	28	225	25360	6.2	Uncharacterized protein
3209	P14847	4	4	28	225	25361	6.2	C-reactive protein
3210	Q9EQW9	4	11	27.4	146	16879	8.7	Ionized calcium binding adapter molecule 1
3210	O70200	4	11	27.2	147	16911	8.8	Allograft inflammatory factor 1
3211	UPI00001E501A	4	6	30.6	235	24677	8.6	PREDICTED: ES1 protein homolog, mitochondrial-like isoform 2
3211	Q9D172	4	6	27.1	266	28090	8.8	ES1 protein homolog, mitochondrial
3212	P70202	4	7	26.6	222	25492	5.7	Latexin
3213	Q9CQ89	4	68	26	177	18865	6.8	Protein CutA
3214	E9QJS2	4	6	25.8	225	25054	6.1	Uncharacterized protein
3214	Q99LS3	4	6	25.8	225	25096	6.1	Phosphoserine phosphatase
3215	A2A9W3	4	6	25.1	203	23312	5.2	Likely ortholog of H. sapiens AD023 protein
3215	Q3UBZ5	4	6	23	222	25493	5.1	MIF4G domain-containing protein
3216	Q8R395	4	6	25	224	24493	8.1	COMM domain-containing protein 5
3217	Q05BC5	4	11	24.2	248	27499	6.5	Arfgap1 protein
3217	Q9EPJ9-2	4	11	15.3	392	42957	5.5	Isoform 2 of ADP-ribosylation factor GTPase-activating protein 1
3217	Q6PB94	4	11	15.3	392	42932	5.4	Arfgap1 protein
3217	Q3TGS9	4	11	15.2	394	43184	5.5	ADP-ribosylation factor GTPase activating protein 1
3217	Q9EPJ9	4	11	14.5	414	45288	5.6	ADP-ribosylation factor GTPase-activating protein 1
3218	Q9D7I5	4	8	23.7	270	29144	5.1	Phospholysine phosphohistidine inorganic pyrophosphate phosphatase
3219	Q9WUU7	4	6	23.2	306	33996	6.6	Cathepsin Z
3219	E9Q5W3	4	6	23.1	307	34200	6.8	Uncharacterized protein
3219	Q9ES94	4	6	23.1	307	34175	6.6	Cathepsin Z
3220	O70325	4	11	21.8	197	22079	8.4	Phospholipid hydroperoxide glutathione peroxidase, mitochondrial
3220	Q91XR9	4	11	17	253	29102	10.2	Phospholipid hydroperoxide glutathione peroxidase, nuclear
3220	Q76LV0	4	11	17	253	29044	10.2	Glutathione peroxidase
3221	Q8K354	4	4	21.7	277	30953	6.6	Carbonyl reductase 3
3222	Q3TCH7	4	6	20.4	759	87753	8.3	Cullin-4A
3223	O54998	4	5	20.2	218	24913	6	Peptidyl-prolyl cis-trans isomerase FKBP7
3224	Q3U5R8	4	7	19.5	277	30413	5.2	Putative uncharacterized protein
3224	Q9D066	4	7	19.5	277	30395	5.2	Putative uncharacterized protein
3224	Q924B0	4	7	19.5	277	30429	5.2	Inositol (Myo)-1(Or 4)-monophosphatase 1
3225	Q569Z0	4	34	19	216	23493	11	Ribosomal protein L14
3225	Q9CR57	4	34	18.9	217	23564	11	60S ribosomal protein L14
3225	Q9CWK0	4	34	18.1	227	26316	11.9	Putative uncharacterized protein
3226	UPI0000475CC6	4	13	18.9	233	26184	5.1	nuclear ubiquitous casein and cyclin-dependent kinases substrate isoform 2
3226	Q80XU3	4	13	18.8	234	26313	5.1	Nuclear ubiquitous casein and cyclin-dependent kinases substrate
3227	Q3TDG9	4	7	18.2	274	31179	5.6	Putative uncharacterized protein
3227	Q9ER00	4	7	18.2	274	31195	5.4	Syntaxin-12
3227	Q3TIL5	4	7	18.2	274	31254	5.6	Putative uncharacterized protein
3228	O88968	4	6	18.1	430	47586	6.3	Transcobalamin-2
3229	P14602-2	4	10	18.3	197	21961	7	Isoform B of Heat shock protein beta-1
3229	P14602	4	10	17.2	209	23014	6.5	Heat shock protein beta-1
3230	Q9Z2Y8	4	14	19.7	274	30049	8.3	Proline synthase co-transcribed bacterial homolog protein
3230	Q3UYN1	4	14	16.6	326	35873	8.5	Putative uncharacterized protein
3231	Q99KB8	4	16	16.5	309	34084	7.7	Hydroxyacylglutathione hydrolase, mitochondrial
3232	P43274	4	41	15.1	219	21977	11.1	Histone H1.4
3233	Q8BRQ6	4	17	14.8	216	25153	6.2	Putative uncharacterized protein
3233	Q91WN1	4	17	12.4	259	30059	5.9	DnaJ homolog subfamily C member 9
3234	Q9JHH6	4	5	14.7	422	48871	8	Carboxypeptidase B2
3235	Q9ES30	4	19	18.3	246	26828	6.7	Complement C1q tumor necrosis factor-related protein 3
3235	D3YZ61	4	19	14.1	319	34984	7.6	Uncharacterized protein
3236	Q8BH95	4	5	14.1	290	31474	8.5	Enoyl-CoA hydratase, mitochondrial
3237	Q91XL1	4	9	13.5	342	37431	6.6	Leucine-rich HEV glycoprotein
3238	Q8K2C9	4	10	12.2	362	43131	9.1	3-hydroxyacyl-CoA dehydratase 3
3239	P28650	4	6	11.4	457	50254	8.4	Adenylosuccinate synthetase isozyme 1
3239	P28650-2	4	6	10.8	480	52780	7.8	Isoform 2 of Adenylosuccinate synthetase isozyme 1

Table S1. Proteins identified in the OVCAR-3 tumor supernatant by two or more unique peptides.

Protein or Protein Group	Accession Number	Unique Peptide Count	Spectrum Count	Sequence Coverage (%)	Length	MolWt	pI	Descriptive Name
3240	Q8BK62	4	6	11.3	406	45746	6.2	Olfactomedin-like protein 3
3241	Q8BJY1	4	8	11.1	504	55972	5.2	26S proteasome non-ATPase regulatory subunit 5
3242	Q60749	4	9	9.7	443	48371	8.7	KH domain-containing, RNA-binding, signal transduction-associated protein 1
3243	Q9WVH9	4	8	9.6	448	50193	4.7	Fibulin-5
3244	P97384	4	7	9.3	503	54111	7.7	Annexin A11
3244	Q921F1	4	7	9.3	503	54079	7.7	Annexin A11
3245	P21619	4	6	8.6	596	67318	5.5	Lamin-B2
3246	O08795	4	7	8.3	521	58793	4.5	Glucosidase 2 subunit beta
3246	Q3TWG2	4	7	8.3	521	58694	4.5	Putative uncharacterized protein
3246	O08795-2	4	7	8.1	528	59560	4.5	Isoform 2 of Glucosidase 2 subunit beta
3247	Q3TH60	4	33	26.5	170	19842	9.2	Putative uncharacterized protein (Fragment)
3247	P59326	4	33	8.1	559	60879	9	YTH domain family protein 1
3247	A2AWN8	4	33	7.7	586	63900	8.9	YTH domain family 1
3248	B2RTP7	4	27	6.8	707	70923	8.1	Krt2 protein
3249	P70699	4	7	6.6	953	106248	5.8	Lysosomal alpha-glucosidase
3250	P58281	4	4	5.8	960	111339	7.5	Dynamin-like 120 kDa protein, mitochondrial
3250	E9QLC8	4	4	5.7	978	113230	7.3	Uncharacterized protein
3250	P58281-2	4	4	5.6	997	115593	7.6	Isoform 2 of Dynamin-like 120 kDa protein, mitochondrial
3251	UPI0001F79537	4	4	5.2	667	75497	5.6	complement component 1, r subcomponent
3251	Q566I6	4	4	5	707	80096	5.7	Complement component 1, r subcomponent
3251	Q8CG16	4	4	5	707	80073	5.7	Complement C1r-A subcomponent
3252	Q6GTP5	4	5	4.6	930	106599	6.2	Endoplasmic reticulum aminopeptidase 1
3252	Q9EQH2	4	5	4.6	930	106599	6.1	Endoplasmic reticulum aminopeptidase 1
3253	Q6DID3	4	8	4.2	1268	139572	8.2	Protein SCAF8
3254	Q8BYA0	4	5	4	1196	133321	6.5	Tubulin-specific chaperone D
3255	D3Z598	4	5	3.5	1558	166772	5.1	Uncharacterized protein
3255	Q8K4G1-2	4	5	3.4	1600	171376	5.2	Isoform 2 of Latent-transforming growth factor beta-binding protein 4
3255	E9PUG9	4	5	3.4	1600	171303	5.2	Uncharacterized protein
3255	B2RU79	4	5	3.3	1666	178655	5.5	Latent transforming growth factor beta binding protein 4
3255	Q8K4G1-3	4	5	3.3	1666	178107	5.2	Isoform 3 of Latent-transforming growth factor beta-binding protein 4
3255	Q8K4G1	4	5	3.3	1666	178713	5.4	Latent-transforming growth factor beta-binding protein 4
3255	E9QPS5	4	5	3.3	1665	177885	5.3	Uncharacterized protein
3255	E9QPD9	4	5	3.3	1666	178641	5.5	Uncharacterized protein
3256	E9Q854	4	7	2.5	1812	195843	5.3	Uncharacterized protein
3256	Q0VD84	4	7	2.5	1786	192824	5.4	Ltbp2 protein
3256	O08999	4	7	2.5	1813	195829	5.3	Latent-transforming growth factor beta-binding protein 2
3256	E9QNQ3	4	7	2.5	1809	195543	5.4	Uncharacterized protein
3257	UPI000047059A	4	4	2.5	1903	213497	6.6	protein tyrosine phosphatase, receptor type, D
3257	UPI00015DF284	4	4	2.5	1869	209228	6.4	protein tyrosine phosphatase, receptor type, D
3258	E9PZQ1	4	5	2.2	1711	197655	7.6	Uncharacterized protein
3258	UPI000154B165	4	5	2.2	1710	197554	7.6	cDNA sequence BC013672
3259	E9Q2T3	4	7	1.5	3126	339905	5.4	Uncharacterized protein
3259	Q3V5L4	4	7	1.5	3126	339953	5.4	Tenascin-X
3259	O54796	4	7	1.1	4114	447279	5.2	Tenascin-X
3259	O35452	4	7	1.1	4006	435481	5.2	Tenascin X
3260	Q6FG99	4	105	87.7	114	11568	4.4	RPLP1 protein
3261	E9PLX3	4	21	82.2	90	9528	6.9	Uncharacterized protein
3261	O43504	4	21	81.3	91	9614	4.9	Hepatitis B virus X-interacting protein
3262	P14618-2	4	11	80.8	531	58062	7.7	Isoform M1 of Pyruvate kinase isozymes M1/M2
3263	Q9UE89	4	227	78.9	71	7799	7.2	Beta-actin (Fragment)
3264	C9JM50	4	60	61.7	196	20843	5.4	Uncharacterized protein
3265	Q15819	4	19	61.4	145	16363	8	Ubiquitin-conjugating enzyme E2 variant 2
3266	P47224	4	6	60.2	123	13839	5.5	Guanine nucleotide exchange factor MSS4
3267	P63172	4	14	60.2	113	12452	5.1	Dynein light chain Tctex-type 1
3268	P35754	4	10	58.5	106	11776	8.1	Glutaredoxin-1
3269	P14854	4	32	57	86	10192	7	Cytochrome c oxidase subunit 6B1
3270	Q7Z4G1	4	11	55.3	85	9638	6	COMM domain-containing protein 6
3271	Q8WUW1	4	7	54.7	75	8745	5.5	Protein BRICK1
3271	Q8WUW1-2	4	7	38.7	106	12046	6.8	Isoform 2 of Protein BRICK1
3272	Q96B45	4	7	54.3	105	11564	6.8	UPF0693 protein C10orf32
3273	P61457	4	75	52.9	104	12000	6.8	Pterin-4-alpha-carbinolamine dehydratase
3274	Q9Y2V2	4	26	50.3	147	15892	8.2	Calcium-regulated heat stable protein 1
3275	Q9NRG0	4	24	49.6	131	14711	5.1	Chromatin accessibility complex protein 1
3276	A8MT69	4	6	49.4	81	8959	5.9	Centromere protein X
3277	Q99878	4	67	50	128	13936	10.9	Histone H2A type 1-J
3277	Q96KK5	4	67	50	128	13906	10.9	Histone H2A type 1-H
3277	Q9BTM1	4	67	49.6	129	14019	10.9	Histone H2A.J
3277	B2R5B3	4	67	49.2	130	14111	11.1	Histone H2A
3277	P20671	4	67	49.2	130	14107	10.9	Histone H2A type 1-D
3277	P0C0S8	4	67	49.2	130	14091	10.9	Histone H2A type 1
3278	Q9Y2S0	4	9	48.9	133	15237	5.8	DNA-directed RNA polymerases I and III subunit RPAC2
3279	O75531	4	45	48.3	89	10059	6.1	Barrier-to-autointegration factor
3280	Q9BVM4	4	6	47.1	153	17329	6.9	Gamma-glutamylaminecyclotransferase
3281	Q9HCY8	4	11	46.2	104	11662	5.2	Protein S100-A14
3282	C9J9P1	4	6	44.8	125	14203	5.6	Uncharacterized protein
3282	C9K0E3	4	6	25.1	223	25476	8.5	Uncharacterized protein
3282	Q05655	4	6	8.3	676	77505	7.8	Protein kinase C delta type
3283	P98179	4	32	44.6	157	17170	8.9	Putative RNA-binding protein 3
3284	E5RGS4	4	15	44.4	117	13466	5.5	Uncharacterized protein
3284	O60925	4	15	42.6	122	14210	6.8	Prefoldin subunit 1
3285	Q9UNM1	4	12	46.4	97	10295	9	Chaperonin 10-related protein (Fragment)
3285	P61604	4	12	44.1	102	10932	8.9	10 kDa heat shock protein, mitochondrial
3286	Q9BW61	4	5	44.1	102	11835	8.7	DET1- and DDB1-associated protein 1
3287	UPI00015DFAB0	4	18	58.2	98	11267	5.5	Cytochrome b5.
3287	P00167-2	4	18	58.2	98	11268	5.1	Isoform 2 of Cytochrome b5
3287	Q59F44	4	18	43.2	132	14572	5.7	Cytochrome b-5 isoform 1 variant (Fragment)
3287	P00167	4	18	42.5	134	15330	5	Cytochrome b5
3288	Q9NRY2	4	11	42.3	104	11425	9.3	SOSS complex subunit C
3289	Q9H0N5	4	9	41.5	130	14365	9.1	Pterin-4-alpha-carbinolamine dehydratase 2
3290	P63220	4	85	41	83	9111	8.5	40S ribosomal protein S21
3291	P49789	4	4	40.1	147	16858	7.1	Bis(5'-adenosyl)-triphosphatase
3292	P49458	4	10	39.5	86	10112	8	Signal recognition particle 9 kDa protein
3293	P14621	4	5	39.4	99	11140	9.5	Acylphosphatase-2
3294	Q3KRA6	4	7	38.9	126	14609	6.9	UPF0538 protein C2orf76
3295	P29034	4	10	38.8	98	11117	4.8	Protein S100-A2
3296	Q9BU02	4	6	38.7	230	25566	4.8	Thiamine-triphosphatase
3297	Q96HC4	4	29	38.4	596	63945	8.2	PDZ and LIM domain protein 5
3298	P41134-2	4	7	39.6	149	15586	7.8	Isoform ID-B of DNA-binding protein inhibitor ID-1
3298	P41134	4	7	38.1	155	16132	7	DNA-binding protein inhibitor ID-1
3299	O00264	4	7	37.9	195	21671	4.7	Membrane-associated progesterone receptor component 1
3300	Q9NYJ1	4	8	37.9	87	10134	6	Coiled-coil-helix-coiled-coil-helix domain-containing protein 8

Table S1. Proteins identified in the OVCAR-3 tumor supernatant by two or more unique peptides.

Protein or Protein Group	Accession Number	Unique Peptide Count	Spectrum Count	Sequence Coverage (%)	Length	MolWt	pI	Descriptive Name
3301	O75817	4	7	37.1	140	15651	8.9	Ribonuclease P protein subunit p20
3302	Q6IBA3	4	14	39.7	68	7388	7.2	ATOX1 protein
3302	O00244	4	14	39.7	68	7402	7.2	Copper transport protein ATOX1
3302	E5RIM7	4	14	37	73	7874	7.2	Uncharacterized protein
3303	Q8WWX9	4	5	35.9	145	16082	5.5	Selenoprotein M
3303	UPI0000D62134	4	5	35.9	145	16134	5.6	Thioredoxin-like selenoprotein M precursor (Protein SelM).
3304	Q9NWW6	4	4	34.7	199	23193	4.9	Nicotinamide riboside kinase 1
3305	Q548C9	4	5	34.3	429	49167	6.8	Septin 6
3305	B1AMS2	4	5	34.1	431	49303	6.8	Septin 6
3305	Q14141	4	5	33.9	434	49717	6.7	Septin-6
3305	Q6B514	4	5	33.6	438	50204	6.9	Septin 6 isoform E
3305	E7ETH8	4	5	32.2	457	52236	7.2	Uncharacterized protein
3305	B4DZW0	4	5	32.2	457	52184	7.3	cDNA FLJ58821, highly similar to Septin-6
3305	Q8NFH9	4	5	26.3	559	63143	8	MLL/SEPTIN6 fusion protein (Fragment)
3306	Q96FN9	4	5	33.9	168	18660	7.8	Probable D-tyrosyl-tRNA(Tyr) deacylase 2
3307	Q9Y343	4	11	40.2	169	19818	7.2	Sorting nexin-24
3307	A8MXB7	4	11	33.7	202	23728	7.3	Uncharacterized protein
3308	O43924	4	4	33.3	150	17420	5.7	Retinal rod rhodopsin-sensitive cGMP 3',5'-cyclic phosphodiesterase subunit delta
3309	P54725	4	9	33.1	363	39609	4.6	UV excision repair protein RAD23 log A
3309	Q59EU8	4	9	31.7	379	41256	4.7	UV excision repair protein RAD23 log A variant (Fragment)
3310	Q14011	4	81	33.1	172	18648	9.5	Cold-inducible RNA-binding protein
3311	Q969E8	4	8	33	191	20894	4.4	Pre-rRNA-processing protein TSR2 log
3312	Q8WUD4	4	8	31.9	166	19181	7.4	Coiled-coil domain-containing protein 12
3313	Q53G08	4	7	31.4	185	21370	5.6	DNA replication complex GINS protein PSF2 variant (Fragment)
3313	Q9Y248	4	7	31.4	185	21428	5.4	DNA replication complex GINS protein PSF2
3314	Q3MHL6	4	7	31.2	144	15680	5.2	TSC22 domain family protein 1
3315	Q9UBK9	4	8	31.2	157	18246	7.6	Protein UXT
3316	Q9UMX5	4	5	30.8	172	18856	5.7	Neudesin
3317	P51571	4	6	30.6	173	18999	6.2	Translocon-associated protein subunit delta
3317	UPI0000211CBA	4	6	28.8	184	20213	5.8	translocon-associated protein subunit delta isoform 1 precursor
3318	Q99470	4	8	30.3	211	23026	7.3	Stromal cell-derived factor 2
3319	E9PNW4	4	25	29.6	108	11985	5.8	Uncharacterized protein
3319	P13987	4	25	25	128	14177	6.5	CD59 glycoprotein
3319	E9PR17	4	25	24.6	130	14529	7.8	Uncharacterized protein
3320	O43805	4	15	29.4	119	13596	5.4	Sjoegren syndrome nuclear autoantigen 1
3321	Q8NBY1	4	7	31.6	392	43868	5.4	Uncharacterized protein
3321	Q9P289	4	7	29.8	416	46529	5.3	Serine/threonine-protein kinase MST4
3321	Q499L9	4	7	29.8	416	46530	5.2	Serine/threonine protein kinase MST4
3321	E7ERH7	4	7	29.1	426	48010	5.6	Uncharacterized protein
3322	Q9H446	4	6	28.4	243	27939	4.2	RWD domain-containing protein 1
3323	D6RCL8	4	6	27.7	177	19171	9.3	Uncharacterized protein
3323	Q9NY12-2	4	6	24.6	199	20834	10.4	Isoform 2 of H/ACA ribonucleoprotein complex subunit 1
3323	Q9NY12	4	6	22.6	217	22348	10.9	H/ACA ribonucleoprotein complex subunit 1
3324	UPI0000159292	4	10	27.6	123	14234	11.5	UPI0000159292 UniRef100 entry
3325	A8JZY6	4	7	27.1	284	31580	5.3	cDNA FLJ76983, highly similar to sapiens DPH5 log (S. cerevisiae), mRNA
3325	E7EN60	4	7	27.1	284	31515	5.4	Diphthine synthase
3325	Q9H2P9-4	4	7	27	285	31440	5.3	Isoform 4 of Diphthine synthase
3325	Q9H2P9	4	7	27	285	31651	5.3	Diphthine synthase
3325	Q9H2P9-5	4	7	25.9	297	33005	5.5	Isoform 5 of Diphthine synthase
3325	E7EQB6	4	7	25.9	297	32906	5.4	Uncharacterized protein
3326	P30042-2	4	8	30.4	237	24758	8.1	Isoform Short of ES1 protein log, mitochondrial
3326	UPI0000E5A400	4	8	28	257	27449	7.1	UPI0000E5A400 UniRef100 entry
3326	P30042	4	8	26.9	268	28170	8.3	ES1 protein log, mitochondrial
3327	P80723	4	4	26.9	227	22693	4.6	Brain acid soluble protein 1
3328	Q8N5M4	4	11	26.9	171	20013	8.9	Tetratricopeptide repeat protein 9C
3328	UPI0001796E42	4	11	26.9	171	19984	9.2	PREDICTED: similar to tetratricopeptide repeat domain 9C
3329	UPI0001AE6C7C	4	5	29	217	25075	7.4	UPI0001AE6C7C UniRef100 entry
3329	Q86UY6	4	5	26.6	237	27194	7.4	N-alpha-acetyltransferase 40, NatD catalytic subunit
3330	P03973	4	23	26.5	132	14326	8.7	Antileukoproteinase
3331	A8K8D3	4	7	26.4	212	24062	6	cDNA FLJ77372, highly similar to sapiens SUMO/sentrin specific peptidase family member 8 (SENP8), mRNA
3331	Q96LD8	4	7	26.4	212	24107	6	Sentrin-specific protease 8
3332	Q8WUX2	4	5	26.1	184	20875	5.4	Cation transport regulator-like protein 2
3333	O15217	4	5	25.7	222	25704	8.3	Glutathione S-transferase A4
3334	Q9NSI2-2	4	4	27.4	215	23945	10.9	Isoform B of Uncharacterized protein C21orf70
3334	Q9NSI2	4	4	25.7	230	25456	11.1	Uncharacterized protein C21orf70
3335	Q9UFW8	4	11	25.7	167	18820	9	CGG triplet repeat-binding protein 1
3336	P52298	4	5	25.6	156	18001	8.2	Nuclear cap-binding protein subunit 2
3337	Q5T8T7	4	7	38.1	134	15855	4.9	Mediator complex subunit 22
3337	Q15528-2	4	7	36.4	140	16480	5	Isoform Surf5A of Mediator of RNA polymerase II transcription subunit 22
3337	Q15528	4	7	25.5	200	22221	4.7	Mediator of RNA polymerase II transcription subunit 22
3338	B5MBZ5	4	6	25.1	362	40957	5	Uncharacterized protein
3338	Q9HCN4	4	6	24.3	374	41740	4.9	GPN-loop GTPase 1
3339	Q8TDH9	4	6	25.1	187	21609	7.6	Protein Muted log
3340	Q96H79	4	6	25	300	32962	8.1	Zinc finger CCCH-type antiviral protein 1-like
3341	A2TDT2	4	6	24.8	157	18221	6.5	Mutant B-cell CLL/lymphoma 10
3342	P31431	4	16	24.7	198	21642	4.5	Syndecan-4
3343	E9PJK1	4	10	40.6	165	17963	5	Uncharacterized protein
3343	E9PRJ8	4	10	32.1	209	22516	6.2	Uncharacterized protein
3343	UPI0001F784A9	4	10	30.3	221	23797	6.7	UPI0001F784A9 UniRef100 entry
3343	UPI00001F9AFE	4	10	29.8	225	24440	5.5	UPI00001F9AFE UniRef100 entry
3343	P60033	4	10	28.4	236	25809	5.3	CD81 antigen
3343	E9PIF1	4	10	27.7	242	25903	5.3	Uncharacterized protein
3343	A6NMH8	4	10	24.5	274	29806	6.9	Uncharacterized protein
3344	Q9Y3D6	4	23	24.3	152	16938	8.8	Mitochondrial fission 1 protein
3345	B7ZLI5	4	6	23.8	349	37328	7.7	Family with sequence similarity 98, member C
3345	Q17RN3	4	6	23.8	349	37329	7.2	Protein FAM98C
3346	P23610	4	7	23.7	371	39103	6.8	Factor VIII intron 22 protein
3347	P20962	4	17	23.5	102	11530	4.2	Parathymosin
3348	P00813	4	5	23.4	363	40764	5.9	Adenosine deaminase
3349	E9PD43	4	12	26.2	260	30064	9.9	Uncharacterized protein
3349	A2ABK2	4	12	23.1	294	33708	9.7	RD RNA binding protein (Fragment)
3349	B7Z6T4	4	12	20.2	336	38398	9.3	RD RNA binding protein, isoform CRA_a
3349	E9PCL7	4	12	18.1	375	42732	9.3	Uncharacterized protein
3349	P18615	4	12	17.9	380	43240	9.3	Negative elongation factor E
3350	O95685	4	4	23.1	299	32559	8.1	Protein phosphatase 1 regulatory subunit 3D
3350	Q86X09	4	4	23.1	299	32559	8.1	Protein phosphatase 1, regulatory (Inhibitor) subunit 3D
3351	Q8NC96	4	5	22.9	275	29737	6.8	Adaptin ear-binding coat-associated protein 1
3352	Q8WXD5	4	10	22.8	167	18824	5.1	Gem-associated protein 6
3353	Q9H9H4	4	6	22.8	285	31307	7.3	Vacuolar protein sorting-associated protein 37B

Table S1. Proteins identified in the OVCAR-3 tumor supernatant by two or more unique peptides.

Protein or Protein Group	Accession Number	Unique Peptide Count	Spectrum Count	Sequence Coverage (%)	Length	MolWt	pI	Descriptive Name
3354	Q6P2C8-2	4	4	25.1	275	31351	9.2	Isoform 2 of Mediator of RNA polymerase II transcription subunit 27
3354	Q6P2C8	4	4	22.2	311	35432	9.3	Mediator of RNA polymerase II transcription subunit 27
3355	Q9BS40	4	7	22.1	222	25750	5.8	Latexin
3356	A6NNP0	4	4	22	264	29540	4.4	Uncharacterized protein
3356	Q9UK22	4	4	19.6	296	33328	4.4	F-box only protein 2
3356	C9JQC5	4	4	19.4	299	33493	4.4	Uncharacterized protein
3357	B3KY43	4	4	22	405	44867	5.2	cDNA FLJ46798 fis, clone TRACH3031660, highly similar to cAMP-dependent protein kinase type II-beta regulatory subunit
3357	Q6DHZ2	4	4	21.3	418	46348	4.9	Protein kinase, cAMP-dependent, regulatory, type II, beta
3357	P31323	4	4	21.3	418	46302	4.9	cAMP-dependent protein kinase type II-beta regulatory subunit
3358	Q9H3Q1	4	7	21.9	356	37980	5.2	Cdc42 effector protein 4
3359	Q8NB15-2	4	6	22.6	252	28265	7.7	Isoform 2 of Zinc finger protein 511
3359	Q8NB15	4	6	21.8	262	29428	6.8	Zinc finger protein 511
3360	P15328	4	7	21.4	257	29819	8	Folate receptor alpha
3361	Q8IYL3	4	18	21.4	243	25977	6.9	UPF0688 protein C1orf174
3362	P24593	4	5	21.3	272	30570	8.2	Insulin-like growth factor-binding protein 5
3363	Q70IA6-2	4	5	24.6	142	16500	6.3	Isoform 2 of Mps one binder kinase activator-like 2
3363	B4DKJ9	4	5	21.1	166	19497	7.8	cDNA FLJ60639, highly similar to Mps one binder kinase activator-like 2
3363	Q70IA6	4	5	14.8	237	26927	6.8	Mps one binder kinase activator-like 2
3363	E9PDA5	4	5	13.1	268	30174	7.1	Uncharacterized protein
3363	B4DKP3	4	5	13.1	268	30158	7.1	cDNA FLJ56351, highly similar to Mps one binder kinase activator-like 2
3364	B3KTJ1	4	5	21	291	33048	7.4	cDNA FLJ38349 fis, clone FEBRA1000057, highly similar to PCTP-like protein
3364	Q9Y365	4	5	21	291	33049	7.1	PCTP-like protein
3365	P49755	4	8	21	219	24976	7.5	Transmembrane emp24 domain-containing protein 10
3365	Q53GF9	4	8	20.4	225	25637	6.7	Full-length cDNA 5-PRIME end of clone CS0DF013YM24 of Fetal brain of sapiens (Human) variant (Fragment)
3366	Q9Y3Y2-4	4	19	25.7	202	21918	12	Isoform 3 of Friend of PRMT1 protein
3366	Q9Y3Y2	4	19	21	248	26397	12.2	Friend of PRMT1 protein
3366	Q9Y3Y2-3	4	19	20.9	249	26525	12.2	Isoform 2 of Friend of PRMT1 protein
3367	O60671	4	5	20.9	282	31827	4.8	Cell cycle checkpoint protein RAD1
3368	Q5JPI3	4	6	20.7	329	37541	6.5	Uncharacterized protein C3orf38
3369	Q8N9N7	4	4	20.5	239	26754	8.4	Leucine-rich repeat-containing protein 57
3370	Q8TCF1	4	7	20.5	268	30787	7.4	AN1-type zinc finger protein 1
3371	Q5TA50	4	10	20.1	214	24365	7.2	Glycolipid transfer protein domain-containing protein 1
3372	Q9BT09	4	6	19.8	278	30748	5.5	Protein canopy log 3
3373	Q9NWX6	4	4	19.8	298	34831	8	Probable tRNA(His) guanylyltransferase
3374	P20933	4	11	19.7	346	37208	6.3	N(4)-(beta-N-acetylglucosaminyl)-L-asparaginase
3375	Q9HBL8	4	4	19.7	299	33344	7.5	NmrA-like family domain-containing protein 1
3376	Q9P0M6	4	5	19.4	372	40058	9.7	Core histone macro-H2A.2
3377	O43752	4	9	19.2	255	29176	4.9	Syntaxin-6
3378	B4DW81	4	5	18.9	227	26181	9	cDNA FLJ58863, highly similar to Protein NipSnap3A
3378	Q9UFN0	4	5	17.4	247	28467	9.2	Protein NipSnap log 3A
3379	E9PJE5	4	4	22.1	240	26458	7.5	Uncharacterized protein
3379	Q17R31	4	4	19.3	274	30333	7.2	Putative deoxyribonuclease TATDN3
3379	B7Z1C1	4	4	18.9	281	31180	7.5	cDNA FLJ52692
3379	UPI0000205E43	4	4	18.9	281	31122	7.5	putative deoxyribonuclease TATDN3 isoform 5
3380	B3KP90	4	4	18.8	479	52710	5.1	cDNA FLJ31447 fis, clone NT2NE2000913, highly similar to Protein phosphatase 2C isoform beta (EC 3.1.3.16)
3380	O75688	4	4	18.8	479	52643	5	Protein phosphatase 1B
3381	A2A2L5	4	16	18.4	277	31152	8.5	Chromosome 20 open reading frame 43 (Fragment)
3381	Q9BY42	4	16	16.7	306	33887	8.6	UPF0549 protein C20orf43
3381	B2RB99	4	16	16.7	306	33798	8.6	cDNA, FLJ95387
3381	B4DXL5	4	16	15.2	336	37500	8.4	cDNA FLJ52798
3381	A8MSH5	4	16	15.2	336	37532	8.4	Uncharacterized protein
3382	Q16637-4	4	4	21.2	250	27135	5.2	Isoform SMN-delta57 of Survival motor neuron protein
3382	Q16637-2	4	4	20.2	262	28534	6	Isoform SMN-delta5 of Survival motor neuron protein
3382	Q16637-3	4	4	18.8	282	30450	5.5	Isoform SMN-delta7 of Survival motor neuron protein
3382	E7EQZ4	4	4	18	294	31690	5.7	Uncharacterized protein
3382	Q16637	4	4	18	294	31849	6.5	Survival motor neuron protein
3383	O43291	4	9	17.9	252	28228	8.3	Kunitz-type protease inhibitor 2
3383	Q6LEU8	4	9	17.9	252	28237	8.3	SPINT2 protein (Fragment)
3384	B4DTN4	4	5	17.8	258	27602	8	cDNA FLJ58857, highly similar to N6-adenosine-methyltransferase 70 kDa subunit (EC 2.1.1.62)
3384	B4E2F6	4	5	15.1	305	33149	7	cDNA FLJ61069, highly similar to N6-adenosine-methyltransferase 70 kDa subunit(EC 2.1.1.62)
3384	Q86U44	4	5	7.9	580	64474	6.4	N6-adenosine-methyltransferase 70 kDa subunit
3385	A5YRU8	4	30	20.6	243	26485	6.2	MUC1 isoform T8
3385	Q0VAP6	4	30	19.6	255	27566	6.4	HCG1996357, isoform CRA_f
3385	B6ECA1	4	30	19.3	259	28297	6.7	MUC1 isoform J13
3385	Q0VAP5	4	30	18.9	264	28405	6.7	Mucin 1, cell surface associated
3385	B6ECA3	4	30	18.7	268	29549	7.1	MUC1 isoform J14
3385	A6ZID5	4	30	18.5	270	29249	6.5	MUC1 isoform M2
3385	B1AVR0	4	30	18.3	273	29592	6.5	HCG1996357, isoform CRA_i
3385	A5YRU5	4	30	17.7	282	30431	6.8	MUC1 isoform Z-LSP
3385	B1AVQ5	4	30	10.5	475	49224	6.9	Mucin 1, cell surface associated
3385	UPI000059C524	4	30	10.3	484	50063	7	mucin-1 isoform 10 precursor
3385	E7EUW3	4	30	8.9	561	57285	7.1	Uncharacterized protein
3385	B4DWK6	4	30	8.9	561	57226	7	cDNA FLJ60927, highly similar to Mucin-1
3385	P15941-4	4	30	4	1243	121021	7.5	Isoform 4 of Mucin-1
3385	P15941-3	4	30	4	1252	121803	7.5	Isoform 3 of Mucin-1
3385	P15941-2	4	30	4	1264	122941	7.6	Isoform 2 of Mucin-1
3385	P15941	4	30	4	1255	122102	7.5	Mucin-1
3386	C9IYN7	4	7	24.4	193	21674	6.4	Uncharacterized protein
3386	E9PFB3	4	7	19.4	242	27155	6.4	Uncharacterized protein
3386	B4DP60	4	7	19.4	242	27125	6.4	cDNA FLJ54324
3386	A8MQ59	4	7	17.7	266	29885	6.8	Uncharacterized protein
3386	Q0PNE2	4	7	17.7	266	29793	6.8	UPF0405 protein C3orf75
3387	O95229	4	5	17.7	277	31293	5.2	ZW10 interactor
3388	B4DYD9	4	11	19.3	322	35264	5.2	cDNA FLJ61465, highly similar to sapiens vacuolar protein sorting 37C (VPS37C), mRNA
3388	A5D8V6	4	11	17.5	355	38659	5.2	Vacuolar protein sorting-associated protein 37C
3389	B4DXU3	4	9	17.5	268	30193	9.2	cDNA FLJ54989, highly similar to Mitochondrial import receptor subunit TOM34
3389	Q15785	4	9	15.2	309	34559	9	Mitochondrial import receptor subunit TOM34
3390	P29084	4	7	17.2	291	33044	9.7	Transcription initiation factor IIE subunit beta
3391	Q9Y303	4	6	16.9	409	43748	6.7	Putative N-acetylglucosamine-6-phosphate deacetylase
3391	Q9Y303-2	4	6	15.7	439	46913	6.7	Isoform 2 of Putative N-acetylglucosamine-6-phosphate deacetylase

Table S1. Proteins identified in the OVCAR-3 tumor supernatant by two or more unique peptides.

Protein or Protein Group	Accession Number	Unique Peptide Count	Spectrum Count	Sequence Coverage (%)	Length	MolWt	pI	Descriptive Name
3391	Q9Y303-3	4	6	11.6	594	63595	7.5	Isoform 3 of Putative N-acetylglucosamine-6-phosphate deacetylase
3392	B4DG34	4	9	16.7	251	28386	4.8	cDNA FLJ58988, highly similar to Charged multivesicular body protein 3
3393	Q8NBJ7-2	4	6	25.4	213	24117	6.9	Isoform 2 of Sulfatase-modifying factor 2
3393	Q8NBJ7	4	6	17.9	301	33843	8	Sulfatase-modifying factor 2
3393	UPI0001AE7023	4	6	16.9	320	35923	9.2	UPI0001AE7023 UniRef100 entry
3393	UPI0000EE7471	4	6	16.9	320	35937	9.2	sulfatase-modifying factor 2 isoform b precursor
3393	E7EMF9	4	6	16.7	323	36355	9.3	Uncharacterized protein
3394	P00973-2	4	4	17.3	364	41740	8.9	Isoform p41 of 2'-5'-oligoadenylate synthase 1
3394	E7EMI9	4	4	16.5	381	43747	9.1	Uncharacterized protein
3394	P00973-4	4	4	16.5	382	43944	9.1	Isoform p44 of 2'-5'-oligoadenylate synthase 1
3394	P00973	4	4	15.8	400	46029	8.2	2'-5'-oligoadenylate synthase 1
3394	P00973-3	4	4	15.2	414	47408	8.9	Isoform p48 of 2'-5'-oligoadenylate synthase 1
3394	Q3ZM02	4	4	13.7	459	52166	9.1	2'-5' oligoadenylate synthetase 1 p52 isoform
3394	E7ET41	4	4	13.7	459	52065	9	Uncharacterized protein
3395	P52799	4	18	16.2	333	36923	8.9	Ephrin-B2
3396	O43760	4	16	16.1	224	24810	4.9	Synaptogyrin-2
3396	Q3KQZ2	4	16	13.1	275	30375	9.2	SYNGR2 protein
3397	B2RDG4	4	6	15.9	508	57433	8.8	cDNA, FLJ96597
3397	Q15397	4	6	12.5	648	73584	9.6	Pumilio domain-containing protein KIAA0020
3398	Q9BXK5-2	4	11	37.8	201	22612	4.9	Isoform 1 of Bcl-2-like protein 13
3398	Q9BXK5	4	11	15.7	485	52723	4.4	Bcl-2-like protein 13
3399	Q9UJW0	4	6	15.7	460	52337	7.3	Dynactin subunit 4
3399	UPI0000EE3766	4	6	15.4	467	53152	7.4	dynactin subunit 4 isoform a
3400	Q13426	4	7	15.5	336	38287	5	DNA repair protein XRCC4
3401	B4DLA4	4	5	15.2	355	39319	8.4	cDNA FLJ60123, highly similar to Coiled-coil domain-containing protein 22
3401	B7ZAR0	4	5	9.3	580	65186	7.2	cDNA, FLJ79274, highly similar to Coiled-coil domain-containing protein 22
3401	B4DPB6	4	5	9.3	580	65156	7.2	cDNA FLJ56537, highly similar to Coiled-coil domain-containing protein 22
3401	O60826	4	5	8.6	627	70756	6.8	Coiled-coil domain-containing protein 22
3402	Q96B23-2	4	6	17.8	342	36977	5.1	Isoform 2 of Uncharacterized protein C18orf25
3402	Q96B23	4	6	15.1	403	43324	4.9	Uncharacterized protein C18orf25
3403	C9JP00	4	15	14.7	348	37899	8.7	Uncharacterized protein
3403	Q9NR56	4	15	13.1	388	41817	8.9	Muscleblind-like protein 1
3404	Q8N749	4	6	19.4	346	38713	5.7	TOM1L1 protein
3404	B4E1N0	4	6	14.3	469	52214	5.4	cDNA FLJ58664, highly similar to TOM1-like 1 protein
3404	O75674	4	6	14.1	476	52989	5.2	TOM1-like protein 1
3405	Q8N142	4	5	14.2	457	50208	8.6	Adenylosuccinate synthetase isozyme 1
3406	Q9NQS3	4	7	14.2	549	61003	6.2	Poliovirus receptor-related protein 3
3407	B3KTT0	4	6	13.9	380	42152	5.5	cDNA FLJ38675 fis, clone IMR322000243, highly similar to Nucleoporin Nup43
3407	Q8NFH3	4	6	13.9	380	42151	5.6	Nucleoporin Nup43
3407	UPI0000458924	4	6	12	441	48485	6.2	UPI0000458924 UniRef100 entry
3408	UPI0000D4DA79	4	7	16.3	257	29004	8.8	ribonuclease H2 subunit B isoform 2
3408	Q05DR2	4	7	13.6	308	34755	9.1	RNASEH2B protein (Fragment)
3408	Q5TBB1	4	7	13.5	312	35139	9.1	Ribonuclease H2 subunit B
3408	Q8N451	4	7	12.7	331	37359	9.1	RNASEH2B protein
3409	Q13643	4	6	13.6	280	31192	6.2	Four and a half LIM domains protein 3
3409	Q9P100	4	6	13.6	280	31282	6.2	LIM-only protein FHL3
3410	Q9NZ63	4	8	13.5	289	33688	6.7	Uncharacterized protein C9orf78
3411	A6NKZ2	4	6	13.3	413	47066	6.4	Uncharacterized protein
3411	P51606	4	6	12.9	427	48831	6.4	N-acylglucosamine 2-epimerase
3412	UPI000066D90A	4	4	15.8	386	42656	4.7	UPI000066D90A UniRef100 entry
3412	UPI000066D909	4	4	14.2	430	47642	4.7	UPI000066D909 UniRef100 entry
3412	O75312	4	4	13.3	459	50925	4.7	Zinc finger protein ZPR1
3413	A8MW61	4	6	13.2	514	57182	9.2	Uncharacterized protein
3413	O43660	4	6	13.2	514	57194	9.2	Pleiotropic regulator 1
3414	Q9UBL3-2	4	6	16.4	501	56477	6.9	Isoform 2 of Set1/Ash2 histone methyltransferase complex subunit ASH2
3414	Q9UBL3-3	4	6	15.4	534	60208	7	Isoform 3 of Set1/Ash2 histone methyltransferase complex subunit ASH2
3414	A8K6T5	4	6	13.1	628	68746	5.7	cDNA FLJ75144, highly similar to sapiens ash2 (absent, small, or homeotic)-like (ASH2L), mRNA
3414	Q9UBL3	4	6	13.1	628	68723	5.7	Set1/Ash2 histone methyltransferase complex subunit ASH2
3415	Q9BQE5	4	6	16.9	337	37092	6.7	Apolipoprotein L2
3415	B4E1T5	4	6	12.7	449	48885	6	cDNA FLJ55764, highly similar to Apolipoprotein-L2
3415	UPI0001AE62FE	4	6	12.7	449	48929	6	UPI0001AE62FE UniRef100 entry
3416	P24864-3	4	6	13.2	395	45150	5.9	Isoform 3 of G1/S-specific cyclin-E1
3416	P24864	4	6	12.7	410	47077	6	G1/S-specific cyclin-E1
3417	O60870	4	7	12.5	393	45374	9	DNA/RNA-binding protein KIN17
3418	B4DUI2	4	4	12.4	421	48273	6.4	cDNA FLJ61415, highly similar to Protein kinase C and casein kinase substratein neurons protein 3
3418	Q9UKS6	4	4	12.3	424	48487	6.2	Protein kinase C and casein kinase substrate in neurons protein 3
3418	D3DQR0	4	4	12.2	425	48532	6.3	Protein kinase C and casein kinase substrate in neurons 3, isoform CRA_b
3419	C9JA77	4	49	12.4	355	38553	5	Uncharacterized protein
3420	Q9UPT5-2	4	10	13.3	653	74713	6.4	Isoform 2 of Exocyst complex component 7
3420	B8XXP2	4	10	12.9	676	77303	6.5	EXOC7 splice variant 5
3420	Q9UPT5-1	4	10	12.7	684	78060	6.7	Isoform 1 of Exocyst complex component 7
3420	B5MC69	4	10	12.3	707	80650	6.8	EXOC7 splice variant 6
3420	Q9UPT5	4	10	11.8	735	83382	6.8	Exocyst complex component 7
3421	Q9H0C8	4	15	12.2	392	42907	7.1	Integrin-linked kinase-associated serine/threonine phosphatase 2C
3422	Q9H814	4	6	12.2	394	44403	5.4	Phosphorylated adapter RNA export protein
3423	B4E1M2	4	7	13.7	502	56629	6.3	cDNA FLJ54951, highly similar to Epoxide hydrolase 2 (EC 3.3.2.3)
3423	E5RFU2	4	7	13.2	523	58856	6	Uncharacterized protein
3423	P34913	4	7	12.4	555	62616	6.3	Epoxide hydrolase 2
3423	B4DFE9	4	7	12.1	572	64731	6.5	cDNA FLJ59619, highly similar to Epoxide hydrolase 2 (EC 3.3.2.3)
3423	E7ETW9	4	7	12.1	572	64701	6.7	Uncharacterized protein
3424	E7ENA3	4	8	17.9	385	41675	5.7	Uncharacterized protein
3424	B4DSE2	4	8	17.9	385	41676	5.5	cDNA FLJ57277, highly similar to Tripeptidyl-peptidase 1 (EC 3.4.14.9)
3424	E7EV34	4	8	13.9	496	53828	6.4	Uncharacterized protein
3424	B4E0C7	4	8	13.9	496	53844	6.4	cDNA FLJ58558, highly similar to Tripeptidyl-peptidase 1 (EC 3.4.14.9)
3424	B5MDC1	4	8	12.4	555	60369	6.4	Uncharacterized protein
3424	Q53HP2	4	8	12.3	563	61213	6.5	Tripeptidyl-peptidase I variant (Fragment)

Table S1. Proteins identified in the OVCAR-3 tumor supernatant by two or more unique peptides.

Protein or Protein Group	Accession Number	Unique Peptide Count	Spectrum Count	Sequence Coverage (%)	Length	MolWt	pI	Descriptive Name
3424	O14773	4	8	12.3	563	61248	6.5	Tripeptidyl-peptidase 1
3424	B4DIV8	4	8	12.1	572	62212	6.7	cDNA FLJ56402, highly similar to Tripeptidyl-peptidase 1 (EC 3.4.14.9)
3425	Q5TZP9	4	6	13.1	396	44102	7.4	Cyclin B1
3425	A8K066	4	6	12	433	48237	7.8	cDNA FLJ75563, highly similar to sapiens cyclin B1 (CCNB1), mRNA
3425	P14635	4	6	12	433	48337	7.5	G2/mitotic-specific cyclin-B1
3426	Q8TCB0	4	4	11.9	444	50491	6.9	Interferon-induced protein 44
3427	Q96CS3	4	8	11.9	445	52624	5.6	FAS-associated factor 2
3428	B4DTG6	4	9	11.8	422	46411	9.5	cDNA FLJ54754, highly similar to Protein FAM61A
3428	Q8ND56-2	4	9	10.8	463	50554	9.5	Isoform 2 of Protein LSM14 log A
3428	Q8ND56	4	9	10.8	463	50530	9.5	Protein LSM14 log A
3429	P15529-7	4	6	13.2	355	39804	5.8	Isoform L of Membrane cofactor protein
3429	P15529-6	4	6	12.7	370	41183	6	Isoform J of Membrane cofactor protein
3429	P15529-4	4	6	12.7	369	41359	6.3	Isoform F of Membrane cofactor protein
3429	P15529-5	4	6	12.2	385	42683	6.4	Isoform H of Membrane cofactor protein
3429	P15529-3	4	6	12.2	384	42738	6.5	Isoform D of Membrane cofactor protein
3429	P15529-2	4	6	11.8	399	44238	6.9	Isoform B of Membrane cofactor protein
3430	A4PIW0	4	8	12.5	457	50106	6.2	Fusion protein SYT-SSX2
3430	A4PIV9	4	8	11.7	488	53819	5.8	Fusion protein SYT-SSX2
3431	Q9NVH1-3	4	5	12	507	57197	7.5	Isoform 3 of DnaJ log subfamily C member 11
3431	B4DPK2	4	5	11.7	521	58842	9	cDNA FLJ59729, highly similar to DnaJ log subfamily C member 11
3431	Q9NVH1	4	5	10.9	559	63278	8.4	DnaJ log subfamily C member 11
3432	B4DDD1	4	7	11.6	484	54097	6.7	cDNA FLJ57803, highly similar to sapiens RNA pseudouridylate synthase domain containing 2 (RPUSD2), mRNA
3432	Q8IZ73	4	7	10.3	545	61312	7.2	RNA pseudouridylate synthase domain-containing protein 2
3433	B3KMB3	4	42	11.4	484	52950	7.5	cDNA FLJ10629 fis, clone NT2RP2005620, highly similar to Epsin-2
3433	E9PBC1	4	42	11.4	484	53008	7.5	Uncharacterized protein
3433	E7EMC3	4	42	11	499	54211	7.1	Uncharacterized protein
3433	B7ZKM5	4	42	9.5	577	61524	7.5	Putative uncharacterized protein
3433	Q52LD0	4	42	9.4	584	62272	7.5	Epsin 2
3433	O95208-2	4	42	9.4	583	62213	7.5	Isoform 2 of Epsin-2
3433	E9PBC2	4	42	9.4	584	62300	7.5	Uncharacterized protein
3433	O95208	4	42	8.6	641	68482	7.5	Epsin-2
3434	Q9BXS6-4	4	10	11.8	425	47570	10	Isoform 4 of Nucleolar and spindle-associated protein 1
3434	Q9BXS6-5	4	10	11.4	440	49324	9.9	Isoform 5 of Nucleolar and spindle-associated protein 1
3434	Q9BXS6-3	4	10	11.4	439	49196	9.9	Isoform 3 of Nucleolar and spindle-associated protein 1
3434	Q9BXS6-2	4	10	11.4	440	49324	9.9	Isoform 2 of Nucleolar and spindle-associated protein 1
3434	A8K4B4	4	10	11.3	441	49439	9.9	cDNA FLJ78441, highly similar to sapiens nucleolar and spindle associated protein 1 (NUSAP1),mRNA
3434	Q9BXS6	4	10	11.3	441	49452	9.9	Nucleolar and spindle-associated protein 1
3435	Q5TEJ8	4	6	10.9	643	72049	6.1	Protein THEMIS2
3436	B4DW34	4	9	10.8	455	50786	5.6	cDNA FLJ56798, highly similar to Acid sphingomyelinase-like phosphodiesterase 3b (EC 3.1.4.-)
3436	Q92485	4	9	10.8	455	50814	5.6	Acid sphingomyelinase-like phosphodiesterase 3b
3437	Q53GF0	4	4	10.8	434	48329	7.9	Cytidine 5'-monophosphate N-acetylneuraminic acid synthetase variant (Fragment)
3437	Q8NFW8	4	4	10.8	434	48379	7.9	N-acylneuraminate cytidylyltransferase
3438	Q15650	4	7	10.7	581	66146	7.9	Activating signal cointegrator 1
3439	Q15018	4	7	10.6	415	46901	6.2	BRISC complex subunit Abro1
3440	Q9NUQ6-2	4	24	12.7	489	54178	9.6	Isoform 2 of SPATS2-like protein
3440	Q9NUQ6	4	24	11.1	558	61729	9.6	SPATS2-like protein
3440	B4DT67	4	24	10.5	588	65152	9.7	cDNA FLJ59751, weakly similar to Mus musculus spermatogenesis associated, serine-rich 2 (Spats2), mRNA
3441	Q59G73	4	8	12.8	431	49222	6.2	Exosome component 10 isoform 2 variant (Fragment)
3441	B4DFE4	4	8	10.4	529	61316	6.5	cDNA FLJ59618, highly similar to Exosome component 10
3441	B4DKG8	4	8	8.1	679	77770	6.7	cDNA FLJ60634, highly similar to Exosome component 10
3441	Q01780-2	4	8	6.4	860	98089	8.4	Isoform 2 of Exosome component 10
3441	Q01780	4	8	6.2	885	100831	8.5	Exosome component 10
3442	E9PNM1	4	4	12	410	47285	6.5	Uncharacterized protein
3442	P37268	4	4	11.8	417	48115	6.5	Squalene synthase
3442	B4DND3	4	4	10.3	474	53405	7.3	cDNA FLJ55760, highly similar to Squalene synthetase (EC 2.5.1.21)
3443	C9JL12	4	6	10.3	516	58958	6	Uncharacterized protein
3443	Q5VSQ6	4	6	9.9	534	60967	6	Procollagen-proline, 2-oxoglutarate 4-dioxygenase (Proline 4-hydroxylase), alpha polypeptide I
3443	P13674	4	6	9.9	534	61049	6	Prolyl 4-hydroxylase subunit alpha-1
3444	Q9H089	4	7	10	658	75225	6.4	Large subunit GTPase 1 log
3445	A8K7A0	4	4	9.8	509	58834	7.9	cDNA FLJ75753, highly similar to sapiens primase, polypeptide 2A, 58kDa (PRIM2A), mRNA
3445	P49643	4	4	9.8	509	58806	7.9	DNA primase large subunit
3446	Q9H9A6	4	4	9.8	602	68250	6.4	Leucine-rich repeat-containing protein 40
3447	Q8IY67	4	8	11.7	606	63877	8.5	Ribonucleoprotein PTB-binding 1
3447	Q8IY67-2	4	8	9.6	739	77860	8.8	Isoform 2 of Ribonucleoprotein PTB-binding 1
3447	E9PAU2	4	8	9.4	756	79579	8.9	Uncharacterized protein
3448	C9JT19	4	17	9.2	380	41494	5.2	Uncharacterized protein
3448	Q6ZPD6	4	17	9.2	379	41379	5.3	cDNA FLJ25992 fis, clone DMC03508
3448	E7ENV4	4	17	9.2	379	41365	5.3	Uncharacterized protein
3449	E1CEI4	4	8	9	599	68630	5.9	Glutamate-cysteine ligase delta4 alternative splicing variant
3449	P48506	4	8	8.5	637	72766	6.1	Glutamate--cysteine ligase catalytic subunit
3450	Q9H9T3	4	4	8.8	547	62259	8.9	Elongator complex protein 3
3451	E9PC74	4	7	8.7	705	78347	5.2	Uncharacterized protein
3451	Q13144	4	7	8.5	721	80380	5.1	Translation initiation factor eIF-2B subunit epsilon
3452	B7Z499	4	8	10.5	488	57205	8.3	cDNA FLJ50571, highly similar to Myotubularin (EC 3.1.3.48)
3452	B7Z491	4	8	9	566	66053	8.4	cDNA FLJ50569, highly similar to Myotubularin (EC 3.1.3.48)
3452	A8K6P5	4	8	8.5	603	69905	8.2	cDNA FLJ76887, highly similar to sapiens myotubularin 1 (MTM1), mRNA
3452	Q13496	4	8	8.5	603	69932	8.2	Myotubularin
3453	Q59GC1	4	5	10.2	527	59154	8.6	Fragile X mental retardation 1 variant (Fragment)
3453	Q06787-8	4	5	9.2	586	66246	8.5	Isoform 8 of Fragile X mental retardation 1 protein
3453	Q06787-5	4	5	8.9	607	68455	8.5	Isoform 4 of Fragile X mental retardation 1 protein
3453	UPI00004188C3	4	5	8.8	611	68966	7.4	fragile X mental retardation 1 protein isoform ISO7
3453	Q06787-3	4	5	8.7	620	70025	7.8	Isoform 2 of Fragile X mental retardation 1 protein
3453	Q06787	4	5	8.5	632	71175	7.4	Fragile X mental retardation 1 protein
3454	B2RDI0	4	4	8.4	608	68447	7.6	cDNA, FLJ96618, highly similar to sapiens tyrosyl-DNA phosphodiesterase 1 (TDP1), mRNA
3454	Q9NUW8	4	4	8.4	608	68420	7.6	Tyrosyl-DNA phosphodiesterase 1
3455	E9PHA7	4	9	8.5	698	79192	9.4	Uncharacterized protein
3455	E7ESY4	4	9	8.4	703	79373	9.1	Uncharacterized protein
3455	Q13330	4	9	8.3	715	80786	9.3	Metastasis-associated protein MTA1

Table S1. Proteins identified in the OVCAR-3 tumor supernatant by two or more unique peptides.

Protein or Protein Group	Accession Number	Unique Peptide Count	Spectrum Count	Sequence Coverage (%)	Length	MolWt	pI	Descriptive Name
3456	B4E367	4	6	8.3	515	58184	6.4	cDNA FLJ61564, highly similar to Plexin domain-containing protein 2
3456	Q6UX71	4	6	8.1	529	59583	6.4	Plexin domain-containing protein 2
3457	Q9UID3	4	11	8.3	782	86042	6.5	Protein fat-free log
3458	O75155-2	4	7	8.9	1119	122664	5.9	Isoform 2 of Cullin-associated NEDD8-dissociated protein 2
3458	O75155	4	7	8.1	1236	135256	5.7	Cullin-associated NEDD8-dissociated protein 2
3459	Q8TCG1	4	8	8.1	905	102185	6.2	Protein CIP2A
3460	A5PL29	4	8	10	601	66479	5.3	EPS15L1 protein
3460	A2RRF3	4	8	8	754	83248	5.1	EPS15L1 protein
3460	A8K5P4	4	8	7.9	756	83501	5.1	cDNA FLJ77869
3460	Q9UBC2	4	8	6.9	864	94255	5.1	Epidermal growth factor receptor substrate 15-like 1
3460	UPI0000D4C04A	4	8	6.6	910	99606	5.1	UPI0000D4C04A UniRef100 entry
3460	B4DKA3	4	8	6.6	910	99607	5	cDNA FLJ60624, highly similar to Epidermal growth factor receptor substrate 15-like 1
3461	D6W5A3	4	4	8	638	73352	5.4	Prolyl endopeptidase-like, isoform CRA_a
3461	Q4J6C6	4	4	7	727	83927	6.4	Prolyl endopeptidase-like
3462	UPI0001AE6648	4	7	9.4	534	60684	4.8	Rab11 family-interacting protein 4 (Rab11-FIP4).
3462	Q86YS3-2	4	7	9.3	535	60771	4.9	Isoform 2 of Rab11 family-interacting protein 4
3462	Q86YS3	4	7	7.8	637	71928	4.9	Rab11 family-interacting protein 4
3463	A8K3W4	4	11	7.7	756	84822	8.8	cDNA FLJ75163, highly similar to sapiens heterogeneous nuclear ribonucleoprotein U-like 1 (HNRPUL1), transcript variant 4, mRNA
3463	Q9BUJ2-4	4	11	7.7	756	84794	8.8	Isoform 4 of Heterogeneous nuclear ribonucleoprotein U-like protein 1
3463	Q9BUJ2-2	4	11	7.2	804	90292	6.9	Isoform 2 of Heterogeneous nuclear ribonucleoprotein U-like protein 1
3463	Q9BUJ2	4	11	6.8	856	95739	6.9	Heterogeneous nuclear ribonucleoprotein U-like protein 1
3463	A8K6U7	4	11	6.8	856	95679	6.9	cDNA FLJ78252, highly similar to sapiens heterogeneous nuclear ribonucleoprotein U-like 1 (HNRPUL1), transcript variant 1, mRNA
3463	A8K5K0	4	11	6.8	856	95649	7.6	cDNA FLJ78309, highly similar to sapiens heterogeneous nuclear ribonucleoprotein U-like 1 (HNRPUL1), transcript variant 1, mRNA
3464	O43290	4	5	7.6	800	90255	6.1	U4/U6.U5 tri-snRNP-associated protein 1
3465	P52815	4	5	16.2	198	21348	8.9	39S ribosomal protein L12, mitochondrial
3465	B4DLN1	4	5	7.2	442	48099	9.5	cDNA FLJ60124, highly similar to Mitochondrial dicarboxylate carrier
3466	Q6P275	4	10	7.2	1221	140090	5.5	STAG1 protein
3466	Q8WVM7	4	10	7	1258	144427	5.6	Cohesin subunit SA-1
3467	Q86Y56	4	6	7.1	855	93521	6.4	HEAT repeat-containing protein 2
3468	Q9UKW4-2	4	5	8	753	86696	6.8	Isoform Beta of Guanine nucleotide exchange factor VAV3
3468	Q9UKW4	4	5	7.1	847	97776	7.1	Guanine nucleotide exchange factor VAV3
3468	B7ZLR1	4	5	6.9	875	100814	7	VAV3 protein
3468	E9PQ97	4	5	6.9	875	100828	7	Uncharacterized protein
3469	Q6V1X1-4	4	5	7.8	782	89908	5.4	Isoform 4 of Dipeptidyl peptidase 8
3469	Q6V1X1-5	4	5	7.2	849	97739	5.4	Isoform 5 of Dipeptidyl peptidase 8
3469	Q6V1X1-2	4	5	7.2	847	97464	5.9	Isoform 2 of Dipeptidyl peptidase 8
3469	Q6V1X1-3	4	5	6.9	882	101422	5.6	Isoform 3 of Dipeptidyl peptidase 8
3469	B4DLA8	4	5	6.9	882	101416	5.6	cDNA FLJ56045, highly similar to Dipeptidyl peptidase 8 (EC 3.4.14.5)
3469	A8K4U2	4	5	6.8	898	103214	5.9	cDNA FLJ77467, highly similar to sapiens dipeptidylpeptidase 8 (DPP8), transcript variant 3, mRNA
3469	Q6V1X1	4	5	6.8	898	103359	5.8	Dipeptidyl peptidase 8
3470	B4DTP5	4	4	6.8	968	110345	6.2	cDNA FLJ61394, highly similar to Serine/threonine-protein kinase N2 (EC 2.7.11.13)
3470	Q16513	4	4	6.7	984	112035	6.3	Serine/threonine-protein kinase N2
3471	B4DGN8	4	4	7.1	690	79400	7	cDNA FLJ53377, highly similar to Procollagen-lysine, 2-oxoglutarate 5-dioxygenase 1 (EC 1.14.11.4)
3471	B2R5M9	4	4	6.7	727	83504	7	cDNA, FLJ92537, highly similar to sapiens procollagen-lysine, 2-oxoglutarate 5-dioxygenase (lysine hydroxylase, Ehlers-Danlos syndrome type VI) (PLOD), mRNA
3471	Q02809	4	4	6.7	727	83550	6.9	Procollagen-lysine,2-oxoglutarate 5-dioxygenase 1
3471	B4DR87	4	4	6.3	774	88273	7.1	cDNA FLJ59393, highly similar to Procollagen-lysine,2-oxoglutarate5-dioxygenase 1 (EC 1.14.11.4)
3472	Q5QP37	4	4	6.7	862	98676	6.3	Itchy E3 ubiquitin protein ligase log (Mouse)
3472	Q96J02	4	4	6.4	903	102803	6.3	E3 ubiquitin-protein ligase Itchy log
3473	A6NJZ9	4	5	6.6	800	92624	9.2	Uncharacterized protein
3473	Q8WTT2	4	5	6.6	800	92548	9.2	Nucleolar complex protein 3 log
3473	A8K905	4	5	6.6	800	92549	9.1	cDNA FLJ77615, highly similar to sapiens nucleolar complex associated 3 log (S. cerevisiae) (NOC3L), mRNA
3474	P51784	4	4	6.6	963	109817	5.5	Ubiquitin carboxyl-terminal hydrolase 11
3475	B4DLC7	4	8	6.5	847	97487	5.5	cDNA FLJ60293, highly similar to Signal transducer and activator of transcription 2
3475	UPI0001CB7F8B	4	8	6.5	847	97457	5.5	signal transducer and activator of transcription 2 isoform 2
3475	P52630	4	8	6.5	851	97916	5.5	Signal transducer and activator of transcription 2
3476	Q14432	4	5	6.5	1141	124979	6	cGMP-inhibited 3',5'-cyclic phosphodiesterase A
3477	P49746	4	7	6.4	956	104201	4.7	Thrombospondin-3
3477	Q53FK6	4	7	6.4	956	104299	4.7	Thrombospondin 3 variant (Fragment)
3477	Q2HIZ0	4	7	6.4	956	104173	4.7	Thrombospondin 3,
3478	B4DH53	4	7	6.5	1033	109788	7.6	cDNA FLJ55328, highly similar to sapiens BPY2 interacting protein 1 (BPY2IP1), mRNA
3478	A8K940	4	7	6.3	1059	112227	7.3	cDNA FLJ77630, highly similar to sapiens BPY2 interacting protein 1, mRNA
3478	Q66K74	4	7	6.3	1059	112211	7.3	Microtubule-associated protein 1S
3479	P23497	4	7	6.1	879	100417	8.2	Nuclear autoantigen Sp-100
3480	Q9P253	4	11	6.1	973	110186	6.1	Vacuolar protein sorting-associated protein 18 log
3481	Q9BZJ0-2	4	4	7.3	687	83148	6.8	Isoform 2 of Crooked neck-like protein 1
3481	Q5JY65	4	4	6	836	99173	8	Crooked neck pre-mRNA splicing factor-like 1 (Drosophila)
3481	Q9BZJ0-3	4	4	6	836	99074	7.9	Isoform 3 of Crooked neck-like protein 1
3481	Q9BZJ0	4	4	5.9	848	100452	8	Crooked neck-like protein 1
3482	Q9Y6X8	4	4	5.9	837	92307	6.9	Zinc fingers and homeoboxes protein 2
3483	A8K5Y5	4	6	5.8	969	105369	5.4	cDNA FLJ78654, highly similar to sapiens nuclear factor of kappa light polypeptide gene enhancer in B-cells 1 (p105) (NFKB1), mRNA
3483	P19838-2	4	6	5.8	969	105427	5.4	Isoform 2 of Nuclear factor NF-kappa-B p105 subunit
3483	P19838	4	6	5.8	968	105356	5.4	Nuclear factor NF-kappa-B p105 subunit
3484	Q2NKK4	4	5	5.3	838	90522	5	Transforming, acidic coiled-coil containing protein 3
3484	Q9Y6A5	4	5	5.3	838	90360	5	Transforming acidic coiled-coil-containing protein 3
3485	Q96JH7	4	4	5.2	1222	134320	7.2	Deubiquitinating protein VCIP135
3486	A6NMQ1	4	5	5.1	1468	166457	5.8	DNA polymerase
3486	P09884	4	5	5.1	1462	165912	5.8	DNA polymerase alpha catalytic subunit
3487	A8K2F9	4	4	5.1	1195	130402	6.3	cDNA FLJ77037, highly similar to sapiens RNA polymerase II associated protein 1, mRNA (Fragment)

Table S1. Proteins identified in the OVCAR-3 tumor supernatant by two or more unique peptides.

Protein or Protein Group	Accession Number	Unique Peptide Count	Spectrum Count	Sequence Coverage (%)	Length	MolWt	pI	Descriptive Name
3487	Q9BWH6-2	4	4	4.6	1315	143582	6.5	Isoform 2 of RNA polymerase II-associated protein 1
3487	Q9BWH6	4	4	4.4	1393	152754	6.4	RNA polymerase II-associated protein 1
3488	B1B0M1	4	4	5.4	796	90841	5.1	GRIP1 associated protein 1
3488	Q4V328-4	4	4	5.3	810	92738	5.2	Isoform 4 of GRIP1-associated protein 1
3488	A8K7Z3	4	4	5.1	841	95990	5.1	cDNA FLJ77229, highly similar to sapiens GRIP1 associated protein 1 (GRIPAP1), transcript variant 1, mRNA
3488	Q4V328	4	4	5.1	841	95989	5.1	GRIP1-associated protein 1
3489	Q86UK7	4	9	5.1	904	98637	8.4	Zinc finger protein 598
3489	Q86UK7-3	4	9	5.1	898	98124	8.4	Isoform 3 of Zinc finger protein 598
3489	Q86UK7-2	4	9	5.1	896	97806	8.3	Isoform 2 of Zinc finger protein 598
3490	Q9NQ66	4	4	4.9	1216	138567	6.2	1-phosphatidylinositol-4,5-bisphosphate phosphodiesterase beta-1
3491	B7Z758	4	6	5	1078	120156	5.8	cDNA FLJ61717, highly similar to Cytospin-A
3491	B2RMV2	4	6	4.8	1117	124576	5.8	CYTSA protein
3491	Q69YQ0	4	6	4.8	1117	124602	5.7	Cytospin-A
3492	E7EP14	4	6	5.1	792	88984	8.6	Uncharacterized protein
3492	B4DMI9	4	6	5.1	792	88929	8.5	cDNA FLJ55894, highly similar to Discs large log 7
3492	B4DRM8	4	6	4.8	842	94148	9.1	cDNA FLJ60069, highly similar to Discs large log 7
3492	A8K732	4	6	4.7	846	95131	9	cDNA FLJ78771, highly similar to sapiens discs, large log 7 (Drosophila), mRNA
3492	Q15398	4	6	4.7	846	95115	9	Disks large-associated protein 5
3492	Q86T11	4	6	4.6	869	97450	9.1	Full-length cDNA clone CS0DH002YN05 of T cells (Jurkat cell line) of sapiens (human) (Fragment)
3493	B3KMC2	4	11	7.9	647	72716	7.4	cDNA FLJ10676 fis, clone NT2RP2006464, highly similar to WD repeat and HMG-box DNA-binding protein 1
3493	B3KMD2	4	11	6.4	798	89594	5.8	cDNA FLJ10723 fis, clone NT2RP3001155, highly similar to WD repeat and HMG-box DNA-binding protein 1
3493	A8MRA5	4	11	5.1	1006	112704	5.7	Uncharacterized protein
3493	A8KAE0	4	11	4.5	1129	126015	5.6	cDNA FLJ78476, highly similar to sapiens WD repeat and HMG-box DNA binding protein 1 (WDHD1), transcript variant 1, mRNA
3493	O75717	4	11	4.5	1129	125967	5.6	WD repeat and HMG-box DNA-binding protein 1
3494	UPI0001BFBBED	4	5	4.7	1245	136540	4.7	WASH complex subunit FAM21C isoform 3
3494	B4DZQ6	4	5	4.7	1245	136464	4.7	cDNA FLJ61504, highly similar to Protein FAM21C
3494	UPI0001AE6CD7	4	5	4.6	1253	136705	4.8	UPI0001AE6CD7 UniRef100 entry
3494	UPI0000D60F51	4	5	4.6	1264	138257	4.8	Protein FAM21C.
3494	Q9Y4E1-3	4	5	4.6	1263	138145	4.8	Isoform 3 of WASH complex subunit FAM21C
3494	Q5SNT6	4	5	4.6	1253	136969	4.8	WASH complex subunit FAM21B
3494	E9PE42	4	5	4.6	1253	136862	4.8	Uncharacterized protein
3494	UPI0001BFBBEC	4	5	4.5	1279	140143	4.7	WASH complex subunit FAM21C isoform 2
3494	B9EK53	4	5	4.5	1279	140098	4.7	FAM21C protein
3494	A8K5W5	4	5	4.4	1320	144805	4.8	cDNA FLJ77522
3494	Q9Y4E1-4	4	5	4.4	1320	144911	4.8	Isoform 4 of WASH complex subunit FAM21C
3494	Q9Y4E1-2	4	5	4.4	1310	143648	4.8	Isoform 2 of WASH complex subunit FAM21C
3494	Q9Y4E1	4	5	4.4	1318	144668	4.8	WASH complex subunit FAM21C
3494	E7ER73	4	5	4.4	1319	144810	4.8	Uncharacterized protein
3494	B7ZME8	4	5	4.4	1320	145075	4.8	FAM21A protein
3494	UPI0001AE6CD6	4	5	4.3	1341	147054	4.8	Protein FAM21C.
3494	Q641Q2	4	5	4.3	1341	147183	4.8	WASH complex subunit FAM21A
3495	Q53F89	4	24	7.1	764	85563	7	Complement factor B (Fragment)
3495	P00751	4	24	7.1	764	85533	7.1	Complement factor B
3495	E7ETN3	4	24	4.8	1115	124016	6.8	Uncharacterized protein
3495	B4E1Z4	4	24	4.3	1266	140942	7.2	cDNA FLJ55673, highly similar to Complement factor B (EC 3.4.21.47)
3495	E7EVA3	4	24	4.3	1266	140943	7.1	Uncharacterized protein
3496	Q8N122	4	5	4.1	1335	149037	6.9	Regulatory-associated protein of mTOR
3497	Q96RT1-7	4	6	4	1302	146086	5.3	Isoform 7 of Protein LAP2
3497	A0AVR1	4	6	3.8	1371	153952	5.5	Erbb2 interacting protein
3497	Q96RT1-5	4	6	3.8	1356	152290	5.4	Isoform 5 of Protein LAP2
3497	Q96RT1-3	4	6	3.8	1360	152652	5.4	Isoform 3 of Protein LAP2
3497	Q96RT1-2	4	6	3.8	1371	153926	5.5	Isoform 2 of Protein LAP2
3497	Q1RMD0	4	6	3.8	1371	153788	5.5	Erbb2 interacting protein
3497	E7EQW9	4	6	3.8	1367	153542	5.4	Uncharacterized protein
3497	Q96RT1	4	6	3.7	1412	158297	5.5	Protein LAP2
3497	E9PCR8	4	6	3.7	1419	159023	5.6	Uncharacterized protein
3497	B7ZLV9	4	6	3.7	1419	159051	5.6	ERBB2IP protein
3498	Q2TAZ0	4	4	3.7	1938	212858	5.9	Autophagy-related protein 2 log A
3498	Q2TAZ0-3	4	4	3.7	1940	213085	5.9	Isoform 2 of Autophagy-related protein 2 log A
3499	UPI0001AE75B5	4	4	3.3	1383	154771	6.2	Receptor-type tyrosine-protein phosphatase gamma precursor (EC 3.1.3.48) (Protein-tyrosine phosphatase gamma) (R-PTP-gamma).
3499	UPI0001AE75B4	4	4	3.3	1354	151539	6	Receptor-type tyrosine-protein phosphatase gamma precursor (EC 3.1.3.48) (Protein-tyrosine phosphatase gamma) (R-PTP-gamma).
3499	B7ZLX5	4	4	3.2	1416	158744	6.2	PTPRG protein
3499	P23470-2	4	4	3.2	1416	158770	6.2	Isoform 2 of Receptor-type tyrosine-protein phosphatase gamma
3499	P23470	4	4	3.1	1445	162003	6.4	Receptor-type tyrosine-protein phosphatase gamma
3500	Q8IWQ7	4	10	3.2	1638	186037	6.4	CDC42-binding protein kinase beta
3500	Q5VT25-3	4	10	3.2	1638	186112	6.3	Isoform 3 of Serine/threonine-protein kinase MRCK alpha
3500	B9EGA7	4	10	3.1	1699	193662	6.7	CDC42BPA protein
3500	Q5VT25-5	4	10	3.1	1719	195921	6.5	Isoform 5 of Serine/threonine-protein kinase MRCK alpha
3500	Q5VT25-4	4	10	3.1	1691	193030	6.3	Isoform 4 of Serine/threonine-protein kinase MRCK alpha
3500	Q5VT25	4	10	3.1	1732	197306	6.6	Serine/threonine-protein kinase MRCK alpha
3500	Q5VT25-6	4	10	3	1781	202811	6.5	Isoform 6 of Serine/threonine-protein kinase MRCK alpha
3500	Q5VT25-2	4	10	3	1754	199809	6.4	Isoform 2 of Serine/threonine-protein kinase MRCK alpha
3501	E7EMX7	4	9	3	973	109164	6.1	Uncharacterized protein
3501	Q9P0K7-3	4	9	3	972	109080	6	Isoform 3 of Ankycorbin
3501	Q9P0K7-2	4	9	3	983	110423	6.1	Isoform 2 of Ankycorbin
3501	Q9P0K7	4	9	3	980	110041	6.2	Ankycorbin
3501	E9PED3	4	9	3	951	106905	6	Uncharacterized protein
3502	Q86XA9-2	4	8	3.2	1965	214081	6.5	Isoform 2 of HEAT repeat-containing protein 5A
3502	Q86XA9	4	8	3	2040	222002	6.6	HEAT repeat-containing protein 5A
3503	UPI0001881A85	4	7	3	1941	211695	5.6	WD repeat-containing protein 81 isoform 1
3504	Q96N67-6	4	8	3	2131	241670	6.8	Isoform 6 of Dedicator of cytokinesis protein 7
3504	Q96N67-5	4	8	3	2109	239420	6.8	Isoform 5 of Dedicator of cytokinesis protein 7
3504	Q96N67-4	4	8	3	2098	238272	6.8	Isoform 4 of Dedicator of cytokinesis protein 7
3504	Q96N67-3	4	8	3	2100	238532	6.8	Isoform 3 of Dedicator of cytokinesis protein 7
3504	Q96N67-2	4	8	3	2129	241410	6.8	Isoform 2 of Dedicator of cytokinesis protein 7
3504	Q96N67	4	8	2.9	2140	242558	6.8	Dedicator of cytokinesis protein 7
3505	P35658	4	26	2.6	2090	213618	7.5	Nuclear pore complex protein Nup214
3505	P35658-5	4	26	2.6	2093	215399	7.7	Isoform 5 of Nuclear pore complex protein Nup214
3505	P35658-4	4	26	2.6	2080	212641	7.4	Isoform 4 of Nuclear pore complex protein Nup214
3505	P35658-3	4	26	2.6	2091	213689	7.5	Isoform 3 of Nuclear pore complex protein Nup214

Table S1. Proteins identified in the OVCAR-3 tumor supernatant by two or more unique peptides.

Protein or Protein Group	Accession Number	Unique Peptide Count	Spectrum Count	Sequence Coverage (%)	Length	MolWt	pI	Descriptive Name
3505	P35658-2	4	26	2.6	2079	212569	7.4	Isoform 2 of Nuclear pore complex protein Nup214
3506	A8KA50	4	6	3.3	1267	137433	4.4	cDNA FLJ78617 (Fragment)
3506	B7Z3E7	4	6	2.4	1760	189918	4.7	cDNA FLJ56510, highly similar to Tumor suppressor p53-binding protein 1
3506	A6NNK5	4	6	2.2	1927	209018	4.7	Uncharacterized protein
3506	UPI000153D540	4	6	2.1	1975	213987	4.7	tumor suppressor p53-binding protein 1 isoform 2
3506	Q12888	4	6	2.1	1972	213573	4.7	Tumor suppressor p53-binding protein 1
3507	Q9HCU4	4	4	2.2	2923	317453	5.3	Cadherin EGF LAG seven-pass G-type receptor 2
3508	A4LAA3	4	6	1.7	2492	282585	6.6	Alpha thalassemia/mental retardation syndrome X-linked
3508	P46100	4	6	1.7	2492	282584	6.6	Transcriptional regulator ATRX
3509	Q96T58	4	7	1.5	3664	402251	7.6	Msx2-interacting protein
3510	B0I1S0	4	4	1.1	4307	492655	6.5	DYNC2H1 variant protein
3510	Q8NCM8-2	4	4	1.1	4314	493425	6.6	Isoform 2 of Cytoplasmic dynein 2 heavy chain 1
3510	Q8NCM8	4	4	1.1	4307	492627	6.5	Cytoplasmic dynein 2 heavy chain 1
3511	P62875	3	11	65.7	67	7645	7.8	DNA-directed RNA polymerases I, II, and III subunit RPABC5 DNA-directed RNA polymerases I, II, and III subunit RPABC5
3512	P61960	3	38	60	85	9118	9.3	Ubiquitin-fold modifier 1 Ubiquitin-fold modifier 1
3512	UPI0000EB10F0	3	38	60	85	9143	9.6	Ubiquitin-fold modifier 1 precursor.
3513	Q49AN9	3	31	78.1	64	7101	7.2	SNRPG protein SNRPG protein
3513	P62308	3	31	65.8	76	8496	8.9	Small nuclear ribonucleoprotein G Small nuclear ribonucleoprotein G
3513	C9J303	3	31	58.1	86	9711	7.2	Uncharacterized protein
3514	Q9NPE3	3	8	57.8	64	7706	10	H/ACA ribonucleoprotein complex subunit 3 H/ACA ribonucleoprotein complex subunit 3
3515	P61204	3	144	54.7	181	20601	7.4	ADP-ribosylation factor 3 ADP-ribosylation factor 3
3515	P84077	3	144	54.7	181	20697	6.8	ADP-ribosylation factor 1 ADP-ribosylation factor 1
3516	Q13885	3	9	52.6	445	49907	4.9	Tubulin beta-2A chain Tubulin beta-2A chain
3517	Q8BUR9	3	3	55.1	78	8132	4.8	Mitotic-spindle organizing protein 1
3517	Q08AG7	3	3	52.4	82	8479	4.8	Mitotic-spindle organizing protein 1
3518	P01116-2	3	6	52.1	188	21425	8.1	Isoform 2B of GTPase KRas
3518	Q5J7N1	3	6	52.1	188	21483	8.1	Kras protein
3519	P62857	3	26	46.4	69	7841	10.7	40S ribosomal protein S28 40S ribosomal protein S28
3520	Q99LT0	3	23	45.5	99	11213	4.9	Protein dpy-30 log
3520	Q9C005	3	23	45.5	99	11250	4.9	Protein dpy-30 log
3521	D6RAH7	3	18	44.9	118	13637	8.3	Ubiquitin carrier protein
3521	Q96RP6	3	18	44.9	118	13635	8.3	Ubiquitin carrier protein
3521	Q9D1S1	3	18	36.1	147	16629	8.2	Ubiquitin carrier protein
3521	P62837	3	18	36.1	147	16735	7.8	Ubiquitin-conjugating enzyme E2 D2 Ubiquitin-conjugating enzyme E2 D2
3521	P61077	3	18	36.1	147	16687	7.8	Ubiquitin-conjugating enzyme E2 D3 Ubiquitin-conjugating enzyme E2 D3
3521	P61077-2	3	18	35.8	148	16785	7.3	Isoform 2 of Ubiquitin-conjugating enzyme E2 D3
3521	P61077-3	3	18	35.6	149	16893	7.8	Isoform 3 of Ubiquitin-conjugating enzyme E2 D3
3522	Q13503	3	5	43.8	144	15564	4.4	Mediator of RNA polymerase II transcription subunit 21
3522	Q9CQ39	3	5	43.8	144	15588	4.5	Mediator of RNA polymerase II transcription subunit 21
3523	B5BU78	3	11	43.4	173	19787	9	Protein tyrosine phosphatase type IVA protein 1
3523	Q93096	3	11	43.4	173	19815	9	Protein tyrosine phosphatase type IVA 1 Protein tyrosine phosphatase type IVA 1
3524	P20340-2	3	12	42.8	208	23549	5.5	Isoform 2 of Ras-related protein Rab-6A
3524	Q6FGX3	3	12	42.8	208	23550	5.4	RAB6A protein
3524	Q3U4W5	3	12	42.8	208	23546	5.5	RAB6, member RAS oncogene family, isoform CRA_c
3525	B4DJP7	3	53	42.5	120	13292	8.9	cDNA FLJ51872, highly similar to Small nuclear ribonucleoprotein Sm D3
3525	Q91VM2	3	53	40.5	126	13985	10.5	Small nuclear ribonucleoprotein D3
3525	P62318	3	53	40.5	126	13916	10.3	Small nuclear ribonucleoprotein Sm D3 Small nuclear ribonucleoprotein Sm D3
3525	UPI0001610F70	3	53	29.1	175	18830	8.3	UPI0001610F70 UniRef100 entry
3526	P56377	3	10	40.8	157	18615	5.5	AP-1 complex subunit sigma-2 AP-1 complex subunit sigma-2
3527	P04271	3	5	40.2	92	10713	4.6	Protein S100-B
3527	P50114	3	5	40.2	92	10728	4.5	Protein S100-B
3528	Q3U281	3	5	36.9	198	22310	6.4	Putative uncharacterized protein
3528	Q9Y6B6	3	5	36.9	198	22410	6.1	GTP-binding protein SAR1b
3528	Q9CQC9	3	5	36.9	198	22382	6.1	GTP-binding protein SAR1b
3528	Q53F37	3	5	36.9	198	22410	6.1	SAR1a gene log 2 variant (Fragment)
3529	O35127	3	13	36.5	126	13194	5.1	Protein C10
3529	Q99622	3	13	36.5	126	13178	5.1	Protein C10
3530	P62745	3	12	35.7	196	22123	5.2	Rho-related GTP-binding protein RhoB Rho-related GTP-binding protein RhoB
3531	Q01658	3	15	34.7	176	19444	4.7	Protein Dr1
3531	Q91WV0	3	15	34.7	176	19431	4.7	Protein Dr1
3531	Q53F47	3	15	34.7	176	19472	4.7	Down-regulator of transcription 1 variant (Fragment)
3531	Q3UMQ2	3	15	34.7	176	19465	4.7	Putative uncharacterized protein
3532	Q5T179	3	30	38.8	67	8027	6.4	CDC28 protein kinase regulatory subunit 1B
3532	P61024	3	30	32.9	79	9660	8.9	Cyclin-dependent kinases regulatory subunit 1 Cyclin-dependent kinases regulatory subunit 1
3532	Q3UNC9	3	30	27.1	96	11553	8	MCG132387
3533	C9K025	3	15	28.7	94	10645	10.7	Uncharacterized protein
3533	UPI0000024BEC	3	15	24.5	110	12478	10.4	PREDICTED: 60S ribosomal protein L35a-like
3533	Q9DC85	3	15	24.5	110	12573	11.1	Putative uncharacterized protein
3533	Q8BQV7	3	15	24.5	110	12563	10.9	Putative uncharacterized protein
3533	P18077	3	15	24.5	110	12538	11.1	60S ribosomal protein L35a
3533	O55142	3	15	24.5	110	12554	10.9	60S ribosomal protein L35a
3533	E9PXU5	3	15	24.5	110	12483	10.7	Uncharacterized protein
3533	D3Z2T7	3	15	24.5	110	12550	10.9	MCG123716
3534	Q9D056	3	11	34.3	169	18796	4.9	Putative uncharacterized protein
3534	P25208	3	11	28	207	22831	4.6	Nuclear transcription factor Y subunit beta
3534	P63139	3	11	28	207	22787	4.6	Nuclear transcription factor Y subunit beta
3534	Q8C590	3	11	25.9	224	24587	4.6	Putative uncharacterized protein
3535	Q8TAX8	3	7	29.1	151	17248	6.5	MYC associated factor X
3535	Q8C4Y1	3	7	29.1	151	17172	6.5	Max protein
3535	E2QZ08	3	7	29.1	151	17202	6.5	Uncharacterized protein
3535	B2RS19	3	7	27.5	160	18245	6.3	Max protein
3535	P61244	3	7	27.5	160	18275	6.3	Protein max
3536	C9JDE5	3	5	26.5	132	15244	7.4	Uncharacterized protein
3536	Q9UL33-2	3	5	25.2	139	16059	6.8	Isoform 2 of Trafficking protein particle complex subunit 2-like protein
3536	Q9JME7	3	5	25.2	139	16018	6.8	Trafficking protein particle complex subunit 2-like protein
3536	Q9UL33	3	5	25	140	16146	6.8	Trafficking protein particle complex subunit 2-like protein
3537	E9PM92	3	21	25.6	156	17506	9.5	Uncharacterized protein
3537	Q9R0P4	3	21	22.1	181	20046	4.8	Small acidic protein
3537	O00193	3	21	21.9	183	20333	4.7	Small acidic protein
3538	O60613	3	6	25.3	162	17640	5	15 kDa selenoprotein
3538	Q9ERR7	3	6	25.3	162	17656	5.4	15 kDa selenoprotein

Table S1. Proteins identified in the OVCAR-3 tumor supernatant by two or more unique peptides.

Protein or Protein Group	Accession Number	Unique Peptide Count	Spectrum Count	Sequence Coverage (%)	Length	MolWt	pI	Descriptive Name
3539	Q9DCL2	3	10	23.8	160	18418	4.8	MIP18 family protein FAM96A
3539	Q9H5X1	3	10	23.8	160	18355	4.9	MIP18 family protein FAM96A
3540	Q8R2U6	3	5	21.8	179	20156	6.4	Diphosphoinositol polyphosphate phosphohydrolase 2
3540	Q9NZJ9	3	5	21.7	180	20306	6.4	Diphosphoinositol polyphosphate phosphohydrolase 2
3541	Q9WV95	3	5	21.6	125	13719	9.7	Pleckstrin logy-like domain family A member 3
3541	Q9Y5J5	3	5	21.3	127	13891	9.7	Pleckstrin logy-like domain family A member 3
3542	C9J212	3	5	21.3	164	18619	8.1	Ubiquitin carrier protein
3542	Q9CY34	3	5	18.9	185	21110	6.5	NEDD8-conjugating enzyme UBE2F
3542	Q969M7	3	5	18.9	185	21077	6.8	NEDD8-conjugating enzyme UBE2F
3542	Q969M7-2	3	5	16.6	211	23760	8	Isoform 2 of NEDD8-conjugating enzyme UBE2F
3543	O15525	3	3	19.8	162	17850	10	Transcription factor MafG
3543	O54790	3	3	19.8	162	17877	10	Transcription factor MafG
3544	Q9BQ15	3	5	21.3	211	22338	9.1	SOSS complex subunit B1
3544	Q8R2Y9	3	5	21.2	212	22628	9.1	SOSS complex subunit B1
3544	E9Q199	3	5	19.7	228	24284	8.7	Uncharacterized protein
3545	B7ZNK7	3	5	18.9	196	21872	6.4	Aes protein
3545	UPI0000231C8F	3	5	18.9	196	21842	6.4	amino-terminal enhancer of split isoform c
3545	Q08117	3	5	18.8	197	21970	6.4	Amino-terminal enhancer of split
3545	P63002	3	5	18.8	197	22000	6.4	Amino-terminal enhancer of split
3546	B4DRD6	3	19	18.1	177	19167	10.8	cDNA FLJ54804, highly similar to Histone H1.0
3546	Q8C1Y3	3	19	17.9	179	19253	10.2	Putative uncharacterized protein
3546	Q3U4Y0	3	19	16.5	194	20847	10.9	Putative uncharacterized protein
3546	P10922	3	19	16.5	194	20861	10.9	Histone H1.0
3546	P07305	3	19	16.5	194	20863	10.8	Histone H1.0
3547	Q9R1Q8	3	23	18.1	199	22471	7.3	Transgelin-3
3547	Q9UI15	3	23	18.1	199	22473	7.3	Transgelin-3
3548	B4DJG8	3	3	17.4	172	19100	6.2	cDNA FLJ57117, highly similar to Charged multivesicular body protein 2b
3548	Q9UQN3	3	3	14.1	213	23907	8.8	Charged multivesicular body protein 2b
3548	Q8BJF9	3	3	14.1	213	23935	8.8	Charged multivesicular body protein 2b
3549	C9J3J3	3	4	16.9	284	31973	4.9	Uncharacterized protein
3549	Q9NXR7-4	3	4	12.8	376	42697	5.4	Isoform 4 of BRCA1-A complex subunit BRE
3549	Q9NXR7-3	3	4	12.7	379	43224	6.2	Isoform 3 of BRCA1-A complex subunit BRE
3549	Q9NXR7	3	4	12.5	383	43552	5.8	BRCA1-A complex subunit BRE
3549	Q8K3W0	3	4	12.5	383	43545	5.9	BRCA1-A complex subunit BRE
3549	D3Z7P0	3	4	12.5	385	43750	6.2	Uncharacterized protein
3549	Q9NXR7-1	3	4	11.6	415	46974	6.1	Isoform 1 of BRCA1-A complex subunit BRE
3550	D6RHI7	3	5	21.3	249	28869	5.9	Uncharacterized protein
3550	E9PDB6	3	5	19.2	276	32101	8.4	Uncharacterized protein
3550	A8K6Q5	3	5	16.4	323	37633	7.2	cDNA FLJ77327, highly similar to sapiens cyclin H (CCNH), mRNA
3550	Q61458	3	5	16.4	323	37506	7.1	Cyclin-H
3550	P51946	3	5	16.4	323	37643	7.2	Cyclin-H
3550	Q3UUW5	3	5	16.2	327	38048	8.7	Putative uncharacterized protein
3551	C9JJQ5	3	3	23.5	149	17041	7.1	Uncharacterized protein
3551	A4D1S5	3	3	16.1	217	24400	6.5	Ras-related protein Rab-19
3551	P35294	3	3	16.1	217	24398	6.4	Ras-related protein Rab-19
3552	P35283	3	4	15.2	243	27329	8.4	Ras-related protein Rab-12
3552	Q6IQ22	3	4	15.2	244	27248	8.4	Ras-related protein Rab-12
3553	Q6UW46	3	28	14.3	315	35963	5.3	VGPW2523
3553	Q80WW9	3	28	14.3	315	35977	5.3	DDRGK domain-containing protein 1
3554	Q8NG11-2	3	4	15	253	28877	6.8	Isoform 2 of Tetraspanin-14
3554	Q8NG11	3	4	14.1	270	30691	6.8	Tetraspanin-14
3554	Q8QZY6	3	4	14.1	270	30674	6.5	Tetraspanin-14
3555	Q8BK26-2	3	6	16.6	211	24794	5.1	Isoform 2 of F-box only protein 44
3555	E9PUJ0	3	6	16.1	218	25446	5.2	Uncharacterized protein
3555	A2A7H5	3	6	13.7	255	29792	5.7	F-box protein 44
3555	Q9H4M3	3	6	13.7	255	29747	5.8	F-box only protein 44
3555	Q8BK26	3	6	13.7	255	29723	5.5	F-box only protein 44
3555	B7ZAN2	3	6	13.7	255	29689	6	cDNA, FLJ79246, highly similar to F-box only protein 44
3556	UPI0000456571	3	5	13.2	228	26510	5.7	Protein CWC15 log.
3556	Q9JHS9	3	5	13.1	229	26624	5.7	Protein CWC15 log
3556	Q9P013	3	5	13.1	229	26624	5.7	Protein CWC15 log
3557	Q5JQF8	3	18	13	200	22799	9.1	Polyadenylate-binding protein 1-like 2
3557	UPI00001E40D1	3	18	11.4	229	25630	5.6	PREDICTED: polyadenylate-binding protein 1-like 2-like
3558	Q7L523	3	4	12.5	313	36566	7.7	Ras-related GTP-binding protein A Ras-related GTP-binding protein A
3559	B1AL69	3	6	11.7	308	35580	5.7	Cell division cycle 37 log (S. cerevisiae)-like 1
3559	Q9CZP7-2	3	6	11.7	307	35407	5.9	Isoform 2 of Hsp90 co-chaperone Cdc37-like 1
3559	Q9CZP7-3	3	6	11.6	310	35812	6.1	Isoform 3 of Hsp90 co-chaperone Cdc37-like 1
3559	Q9CZP7-4	3	6	11.5	312	36037	5.9	Isoform 4 of Hsp90 co-chaperone Cdc37-like 1
3559	Q9CZP7	3	6	10.7	335	38439	5.4	Hsp90 co-chaperone Cdc37-like 1
3559	Q7L3B6	3	6	10.7	337	38835	5.3	Hsp90 co-chaperone Cdc37-like 1
3560	E9Q7N7	3	8	11.7	239	28128	9.6	Uncharacterized protein
3560	Q8BVJ2	3	8	11.7	239	28163	9.6	Putative uncharacterized protein
3560	Q9CSF1	3	8	11.4	245	28332	9.1	Putative uncharacterized protein (Fragment)
3560	Q8R5B7	3	8	5.5	508	57241	7	General transcription factor IIF, polypeptide 1
3560	Q6IBK5	3	8	5.4	517	58226	7.5	GTF2F1 protein
3560	P35269	3	8	5.4	517	58241	7.5	General transcription factor IIF subunit 1
3561	D3YWT0	3	3	16.5	170	19569	9.5	Uncharacterized protein
3561	Q9R0P6	3	3	15.6	179	20626	9.3	Signal peptidase complex catalytic subunit SEC11A
3561	Q6IAM7	3	3	15.6	179	20611	9.5	SPC18 protein
3561	P67812	3	3	15.6	179	20625	9.5	Signal peptidase complex catalytic subunit SEC11A
3561	D3Z569	3	3	14.7	190	21868	9.2	Sec11-like 1 (S. cerevisiae), isoform CRA_e
3561	D3YTS1	3	3	11.3	247	27931	7.7	Sec11-like 1 (S. cerevisiae), isoform CRA_d
3562	Q80UY2	3	20	11	381	41791	5.8	E3 ubiquitin-protein ligase KCMF1
3562	Q9P0J7	3	20	11	381	41945	5.7	E3 ubiquitin-protein ligase KCMF1
3563	Q8CIP2	3	9	17.1	251	27450	8.5	Transcription factor Pax8 (Fragment)
3563	Q06710-5	3	9	15	287	31044	8.6	Isoform 5 of Paired box protein Pax-8
3563	Q06710-4	3	9	13.4	321	34909	8.5	Isoform 4 of Paired box protein Pax-8
3563	Q06710-2	3	9	11.1	387	41741	8.4	Isoform 2 of Paired box protein Pax-8
3563	Q06710-3	3	9	10.8	398	43028	8.8	Isoform 3 of Paired box protein Pax-8
3563	A2AIU6	3	9	10.5	411	44760	8.7	Paired box gene 8 (Fragment)
3563	UPI00015E0A1A	3	9	9.6	450	48075	7.6	Paired box protein Pax-8.
3563	Q06710	3	9	9.6	450	48218	7.8	Paired box protein Pax-8
3563	Q6GU20	3	9	9.4	457	48793	7.8	Paired box gene 8
3563	Q00288	3	9	9.4	457	48851	7.6	Paired box protein Pax-8
3564	D3YUS1	3	6	18.9	244	28166	7.2	Uncharacterized protein
3564	Q8BM63	3	6	17.2	267	30466	6.5	Putative uncharacterized protein (Fragment)
3564	UPI0001C0B3B3	3	6	14.1	327	37383	6.5	5''-AMP-activated protein kinase subunit gamma-2 isoform 2
3564	Q91WG5-2	3	6	14.1	326	37360	6.5	Isoform B of 5'-AMP-activated protein kinase subunit gamma-2
3564	Q3TMN8	3	6	14.1	326	37312	6.5	Putative uncharacterized protein
3564	Q9UGJ0-2	3	6	14	328	37509	6.3	Isoform B of 5'-AMP-activated protein kinase subunit gamma-2

Table S1. Proteins identified in the OVCAR-3 tumor supernatant by two or more unique peptides.

Protein or Protein Group	Accession Number	Unique Peptide Count	Spectrum Count	Sequence Coverage (%)	Length	MolWt	pI	Descriptive Name
3564	B7Z6X8	3	6	10.4	444	50122	8.2	cDNA FLJ54740, highly similar to 5'-AMP-activated protein kinase subunit gamma-2
3564	Q8BIQ9	3	6	10.4	443	49981	8.4	Putative uncharacterized protein
3564	E9PGP6	3	6	10.3	445	50251	7.9	Uncharacterized protein
3564	Q9UGJ0	3	6	8.1	569	63066	9.3	5'-AMP-activated protein kinase subunit gamma-2
3564	Q91WG5	3	6	8.1	566	62997	9.4	5'-AMP-activated protein kinase subunit gamma-2
3564	Q3U4D5	3	6	8.1	566	62921	9.4	Protein kinase, AMP-activated, gamma 2 non-catalytic subunit, isoform CRA_c
3564	E9QK80	3	6	8.1	566	62949	9.4	Uncharacterized protein
3565	P36959	3	7	9.9	345	37419	7.1	GMP reductase 1
3565	Q9DCZ1	3	7	9.9	345	37482	7.1	GMP reductase 1
3566	E7EVU3	3	4	9.6	407	47337	5.5	Uncharacterized protein
3566	B3KMG5	3	4	9.3	418	48552	5.1	cDNA FLJ10938 fis, clone OVARC1001038, highly similar to Protein ariadne-2 log
3566	Q9Z1K6	3	4	7.9	492	57697	5.7	E3 ubiquitin-protein ligase ARIH2
3566	Q53ET9	3	4	7.9	493	57801	5.6	Ariadne log 2 variant (Fragment)
3566	O95376	3	4	7.9	493	57819	5.6	E3 ubiquitin-protein ligase ARIH2
3567	Q9QXV1	3	3	9.9	362	39860	9.9	Chromobox protein log 8
3567	Q9HC52	3	3	9.3	389	43396	9.9	Chromobox protein log 8
3568	A8MTG3	3	11	9.2	447	47749	9.1	Uncharacterized protein
3568	Q9Z326	3	11	8.9	460	49409	9.1	49 kDa zinc finger protein
3568	O43670-2	3	11	8.9	463	49692	9.1	Isoform 2 of Zinc finger protein 207
3568	Q99LA2	3	11	8.8	464	49792	9.1	Putative uncharacterized protein
3568	O43670	3	11	8.6	478	50751	9.1	Zinc finger protein 207
3568	E9PW12	3	11	8.6	479	50850	9.1	Uncharacterized protein
3568	Q9JMD0	3	11	8.3	495	52793	9.1	Putative uncharacterized protein
3568	Q59G94	3	11	8.3	496	52856	9	Zinc finger protein 207 variant (Fragment)
3568	E1P660	3	11	8.3	494	52694	9.1	Zinc finger protein 207, isoform CRA_a
3569	Q9NVG8-2	3	3	13.1	275	32171	5.6	Isoform 2 of TBC1 domain family member 13
3569	A7E2E7	3	3	9	400	46598	5.3	TBC1 domain family, member 13
3569	Q9NVG8	3	3	9	400	46554	5.2	TBC1 domain family member 13
3569	Q8R3D1	3	3	9	400	46453	5.4	TBC1 domain family member 13
3569	B9EGJ8	3	3	9	400	46526	5.2	TBC1 domain family, member 13
3570	O60535	3	3	18.5	200	22270	8.1	Antigen NY-CO-41 (Fragment)
3570	Q9UBB5-2	3	3	14.1	262	29168	9.1	Isoform 2 of Methyl-CpG-binding domain protein 2
3570	Q9UBB5	3	3	9	411	43255	10	Methyl-CpG-binding domain protein 2
3570	E9QMV9	3	3	8.9	414	43501	10	Uncharacterized protein
3570	Q9Z2E1	3	3	8.9	414	43543	10	Methyl-CpG-binding domain protein 2
3571	Q13573	3	6	8.8	536	61495	9.5	SNW domain-containing protein 1
3571	UPI00001B2443	3	6	8.8	536	61462	9.5	SNW domain-containing protein 1
3571	Q9CSN1	3	6	8.8	536	61476	9.5	SNW domain-containing protein 1
3571	Q6I9S2	3	6	8.8	536	61449	9.5	SNW1 protein
3572	Q8CDZ9	3	8	15.6	224	25538	8.6	Putative uncharacterized protein (Fragment)
3572	UPI0001F78E95	3	8	13.7	255	29201	8.8	UPI0001F78E95 UniRef100 entry
3572	Q921T1	3	8	13.7	255	29087	8.5	Tgfb2 protein
3572	Q3TWH5	3	8	10.3	341	39315	8.8	Putative uncharacterized protein (Fragment)
3572	B2R7T2	3	8	8.5	414	47778	8.5	cDNA, FLJ93591, highly similar to sapiens transforming growth factor, beta 2 (TGFB2), mRNA
3572	Q91VP5	3	8	8.5	414	47589	8.4	Transforming growth factor, beta 2
3572	P61812	3	8	8.5	414	47748	8.5	Transforming growth factor beta-2
3572	P27090	3	8	8.5	414	47602	8.5	Transforming growth factor beta-2
3572	P61812-2	3	8	7.9	442	50573	8.4	Isoform B of Transforming growth factor beta-2
3573	Q9H368	3	5	16.1	223	25546	4.5	PRO1670
3573	O88895-2	3	5	15.5	233	26906	5.1	Isoform Short of Histone deacetylase 3
3573	O88895	3	5	8.5	424	48364	5.3	Histone deacetylase 3
3573	O15379	3	5	8.4	428	48848	5.2	Histone deacetylase 3
3573	Q9JM08	3	5	8.4	428	48891	5.2	Histone deacetylase
3573	O15379-2	3	5	8.4	429	49111	5.3	Isoform 2 of Histone deacetylase 3
3574	P27601	3	5	7.7	377	44055	8.2	Guanine nucleotide-binding protein subunit alpha-13
3574	Q3UE40	3	5	7.7	377	44039	8.2	Putative uncharacterized protein
3574	Q14344	3	5	7.7	377	44050	8	Guanine nucleotide-binding protein subunit alpha-13
3575	B7Z1X2	3	8	7.4	566	65005	6.8	cDNA FLJ53876, highly similar to sapiens ADP-ribosylation factor-like 6 interacting protein 2 (ARL6IP2), mRNA
3575	B7Z7X8	3	8	7.4	565	64522	5.7	cDNA FLJ51152, highly similar to sapiens ADP-ribosylation factor-like 6 interacting protein 2 (ARL6IP2), mRNA
3575	Q8NHH9-2	3	8	7.3	579	66087	6.1	Isoform 2 of Atlastin-2
3575	Q8NHH9	3	8	7.2	583	66229	5.5	Atlastin-2
3575	Q6PA06	3	8	7.2	583	66224	5.4	Atlastin-2
3576	B4DM78	3	13	7.3	565	63033	6.8	cDNA FLJ58199, highly similar to Fragile X mental retardation syndrome-related protein 1
3576	E7ERF5	3	13	7.3	565	62991	6.8	Uncharacterized protein
3576	UPI0001F79065	3	13	6.3	650	72849	6.3	fragile X mental retardation gene 1, autosomal log
3576	Q61584-7	3	13	6.3	651	73358	8	Isoform G of Fragile X mental retardation syndrome-related protein 1
3576	Q61584-4	3	13	6.3	650	72814	6.3	Isoform C of Fragile X mental retardation syndrome-related protein 1
3576	UPI0001F79064	3	13	6.1	676	76110	7.3	UPI0001F79064 UniRef100 entry
3576	UPI0001F79063	3	13	6.1	677	76185	7.1	UPI0001F79063 UniRef100 entry
3576	Q61584-3	3	13	6.1	677	76207	7.5	Isoform B of Fragile X mental retardation syndrome-related protein 1
3576	Q61584	3	13	6.1	677	76222	7	Fragile X mental retardation syndrome-related protein 1
3577	A9UF08	3	6	7.6	406	44785	7.2	BCR/ABL fusion protein isoform Y6
3577	A9UEZ4	3	6	7.2	429	47149	6.7	BCR/ABL fusion protein isoform X1
3577	A9UEZ9	3	6	7	441	48603	7	BCR/ABL fusion protein isoform X6
3577	A9UF04	3	6	6.8	454	49792	6.9	BCR/ABL fusion protein isoform Y2
3577	A9UF03	3	6	6.8	458	50148	7.2	BCR/ABL fusion protein isoform Y1
3577	A9UF01	3	6	6.7	465	51155	6.2	BCR/ABL fusion protein isoform X8
3577	A9UF05	3	6	6.6	467	51447	7	BCR/ABL fusion protein isoform Y3
3577	A9UF06	3	6	6	513	56232	6.5	BCR/ABL fusion protein isoform Y4
3577	A9UEZ8	3	6	6	514	56291	6.3	BCR/ABL fusion protein isoform X5
3577	A9UF00	3	6	5.9	524	57619	6.5	BCR/ABL fusion protein isoform X7
3577	A9UEZ7	3	6	5.6	554	60714	6.4	BCR/ABL fusion protein isoform X4
3577	A9UEZ5	3	6	5.6	557	60877	6.5	BCR/ABL fusion protein isoform X2
3577	P11274-2	3	6	2.5	1227	137728	7.2	Isoform 2 of Breakpoint cluster region protein
3577	Q6PAJ1	3	6	2.4	1270	143061	6.9	Breakpoint cluster region protein
3577	P11274	3	6	2.4	1271	142819	7	Breakpoint cluster region protein
3577	E9PZL3	3	6	2.4	1270	143071	6.9	Uncharacterized protein
3577	A9UF02	3	6	1.9	1644	179322	8	BCR/ABL fusion protein isoform X9
3577	A9UEZ6	3	6	1.9	1633	178569	8.3	BCR/ABL fusion protein isoform X3
3577	A9UF07	3	6	1.7	1790	196571	8.4	BCR/ABL fusion protein isoform Y5
3578	Q8WYA6-2	3	12	10.6	376	43392	6.2	Isoform 2 of Beta-catenin-like protein 1
3578	Q0VAM0	3	12	7.1	563	65145	5	Catenin, beta like 1
3578	Q9CWL8	3	12	7.1	563	64980	5.1	Beta-catenin-like protein 1
3578	Q8WYA6	3	12	7.1	563	65173	5	Beta-catenin-like protein 1

Table S1. Proteins identified in the OVCAR-3 tumor supernatant by two or more unique peptides.

Protein or Protein Group	Accession Number	Unique Peptide Count	Spectrum Count	Sequence Coverage (%)	Length	MolWt	pI	Descriptive Name
3578	Q3UKS4	3	12	7.1	563	65008	5.1	Putative uncharacterized protein
3578	Q3UJX2	3	12	7.1	563	65039	5	Putative uncharacterized protein
3579	Q8BWG8-2	3	4	7.1	410	46216	6.4	Isoform 1B of Beta-arrestin-1
3579	P49407-2	3	4	7.1	410	46309	6.3	Isoform 1B of Beta-arrestin-1
3579	A8K103	3	4	6.9	418	47065	6.5	cDNA FLJ75454, highly similar to sapiens arrestin, beta 1 (ARRB1), transcript variant 1, mRNA
3579	Q8BWG8	3	4	6.9	418	46973	6.3	Beta-arrestin-1
3579	P49407	3	4	6.9	418	47066	6.2	Beta-arrestin-1
3580	Q8CA33	3	6	9.5	558	61661	6.6	Putative uncharacterized protein
3580	Q9ESN6	3	6	7.1	744	81445	7	Tripartite motif-containing protein 2
3580	Q9C040	3	6	7.1	744	81530	7	Tripartite motif-containing protein 2
3580	Q3TPI3	3	6	7.1	744	81427	7	Putative uncharacterized protein (Fragment)
3580	E9QKC6	3	6	6.9	770	84319	7.5	Uncharacterized protein
3580	Q3URK9	3	6	6.9	770	84337	7.5	Putative uncharacterized protein
3580	Q5DU27	3	6	6.7	787	86182	7.9	MKIAA0517 protein (Fragment)
3581	Q91Y86	3	6	7.6	384	44229	7.7	Mitogen-activated protein kinase 8 Mitogen-activated protein kinase 8
3581	B5BUB8	3	6	7.6	384	44285	8.1	Mitogen-activated protein kinase 8 isoform JNK1 alpha1 (Fragment)
3581	A6P3E4	3	6	7.6	384	44022	7.5	JNK1 beta1 protein kinase JNK1 beta1 protein kinase
3581	A1L4K2	3	6	6.8	427	48088	6.7	Mitogen-activated protein kinase 8
3581	UPI0000DBECFE	3	6	6.8	427	48178	6.7	mitogen activated protein kinase 8
3581	Q7TSJ7	3	6	6.8	427	48386	6.9	Mapk8 protein
3581	P45983	3	6	6.8	427	48296	6.9	Mitogen-activated protein kinase 8
3582	Q8C9R8	3	4	25.1	187	21440	6.4	Putative uncharacterized protein
3582	UPI0001B791F0	3	4	23	204	23636	7.1	UPI0001B791F0 UniRef100 entry
3582	Q8CBI1	3	4	21.3	221	24966	6.1	Putative uncharacterized protein (Fragment)
3582	UPI0001B791EF	3	4	15.9	295	33343	4.8	UPI0001B791EF UniRef100 entry
3582	O54781	3	4	6.9	681	76757	4.9	Serine/threonine-protein kinase SRPK2
3582	P78362	3	4	6.8	688	77527	5	Serine/threonine-protein kinase SRPK2
3582	C9JQJ0	3	4	6.7	699	79087	5.1	Uncharacterized protein
3582	P78362-2	3	4	6.7	699	79029	5.2	Isoform 2 of Serine/threonine-protein kinase SRPK2
3583	Q15428	3	9	6.9	464	49256	9.6	Splicing factor 3A subunit 2
3583	Q05DF2	3	9	6.7	481	51476	10.1	SF3A2 protein (Fragment)
3583	UPI000002186E	3	9	6.6	485	51279	9.6	splicing factor 3A subunit 2
3583	Q7TN25	3	9	6.6	485	51307	9.6	Sf3a2 protein
3584	B3KRJ9	3	26	6.6	514	58203	9.9	cDNA FLJ34439 fis, clone HLUNG2001146, highly similar to Splicing factor, arginine/serine-rich 12
3584	Q8BZX4-2	3	26	5.6	610	69121	10.3	Isoform 2 of Splicing regulatory glutamine/lysine-rich protein 1
3585	Q5HZI0	3	9	9.1	386	43651	9.6	Rab11fip2 protein (Fragment)
3585	UPI0000194734	3	9	6.8	512	58207	9.5	UPI0000194734 UniRef100 entry
3585	Q7L804	3	9	6.8	512	58279	9.3	Rab11 family-interacting protein 2
3585	B9EID4	3	9	6.6	529	59989	9.3	RAB11 family interacting protein 2 (Class I)
3585	Q3U366	3	9	6.6	529	60027	9.3	Putative uncharacterized protein
3585	Q3I768	3	9	6.6	532	60614	9.4	RAB11-FIP2 long isoform
3586	B7Z592	3	5	6.4	392	44891	7.9	cDNA FLJ61635, highly similar to sapiens likely ortholog of immediate early response, erythropoietin 4 (LEREPO4), mRNA
3586	Q8WU90	3	5	5.9	426	48603	5.3	Zinc finger CCCH domain-containing protein 15
3586	Q3TIV5	3	5	5.9	426	48327	5.3	Zinc finger CCCH domain-containing protein 15
3587	B4DVK6	3	4	6.1	727	81698	6.5	cDNA FLJ55564, highly similar to Probable E3 ubiquitin-protein ligase HECTD3
3587	E7EWG7	3	4	6.1	727	81702	6.5	Uncharacterized protein
3587	Q5T447	3	4	5.1	861	97113	5.6	E3 ubiquitin-protein ligase HECTD3
3587	Q3U487	3	4	5.1	861	97347	5.5	E3 ubiquitin-protein ligase HECTD3
3588	Q59GI5	3	4	7.3	600	68863	7.7	Dynamin 1 isoform 2 variant (Fragment)
3588	B4DHH5	3	4	5.6	790	89577	6.8	cDNA FLJ53472, highly similar to Dynamin-1 (EC 3.6.5.5)
3588	Q05193-3	3	4	5.2	851	96041	7	Isoform 3 of Dynamin-1
3588	P39053-5	3	4	5.2	851	96057	7	Isoform 5 of Dynamin-1
3588	Q05193-4	3	4	5.1	856	96397	7.2	Isoform 4 of Dynamin-1
3588	Q05193	3	4	5.1	864	97408	7.2	Dynamin-1
3588	P39053-6	3	4	5.1	864	97284	7.7	Isoform 6 of Dynamin-1
3588	P39053-4	3	4	5.1	864	97424	7.2	Isoform 4 of Dynamin-1
3588	P39053-2	3	4	5.1	861	97281	7.9	Isoform 2 of Dynamin-1
3588	P39053	3	4	5.1	867	97803	7.7	Dynamin-1
3588	E9QMG2	3	4	5.1	861	97279	7.9	Uncharacterized protein
3589	B4DNM0	3	3	5.6	644	72783	4.5	cDNA FLJ60738, highly similar to RNA polymerase-associated protein LEO1
3589	Q8WVC0	3	3	5.4	666	75404	4.5	RNA polymerase-associated protein LEO1
3589	Q5XJE5	3	3	5.4	667	75641	4.5	RNA polymerase-associated protein LEO1
3589	E9QPK8	3	3	5.4	667	75597	4.5	Uncharacterized protein
3590	B4DZ56	3	6	5.6	549	59246	6.4	cDNA FLJ56810, highly similar to Large proline-rich protein BAT2
3590	E7EMA1	3	6	5.6	549	59272	6.3	Uncharacterized protein
3590	C9JXK4	3	6	5.6	549	59288	6.3	Uncharacterized protein
3590	P48634-4	3	6	2	1533	163046	7	Isoform 4 of Protein PRRC2A
3590	C9JAP8	3	6	2	1533	163090	7	Uncharacterized protein
3590	Q5DTJ4	3	6	1.7	1796	191116	9.4	MKIAA4178 protein (Fragment)
3590	Q7TSC1	3	6	1.4	2158	229200	9.4	Protein PRRC2A
3590	P48634-3	3	6	1.4	2156	229062	9.5	Isoform 3 of Protein PRRC2A
3590	P48634-2	3	6	1.4	2144	227837	9.5	Isoform 2 of Protein PRRC2A
3590	P48634	3	6	1.4	2157	228861	9.5	Protein PRRC2A
3590	C9JXZ3	3	6	1.4	2149	228278	9.5	Uncharacterized protein
3590	C9JTM3	3	6	1.4	2138	227151	9.5	Uncharacterized protein
3590	C9JTL3	3	6	1.4	2149	228291	9.5	Uncharacterized protein
3590	C9JI93	3	6	1.4	2149	228264	9.5	Uncharacterized protein
3590	C9JI26	3	6	1.4	2149	228232	9.5	Uncharacterized protein
3590	C9JD87	3	6	1.4	2149	228275	9.5	Uncharacterized protein
3590	C9JB33	3	6	1.4	2138	227104	9.5	Uncharacterized protein
3590	C9J9U2	3	6	1.4	2138	227136	9.5	Uncharacterized protein
3590	C9J6L0	3	6	1.4	2138	227164	9.5	Uncharacterized protein
3590	C9J1F6	3	6	1.4	2138	227148	9.5	Uncharacterized protein
3591	Q14194	3	8	6.3	572	62184	7	Dihydropyrimidinase-related protein 1
3591	P97427	3	8	6.3	572	62168	7.1	Dihydropyrimidinase-related protein 1
3591	E9PD68	3	8	6.3	570	62030	6.7	Uncharacterized protein
3591	B3KXQ5	3	8	6.3	572	62214	7	cDNA FLJ45854 fis, clone OCBBF2024589, highly similar to Dihydropyrimidinase-related protein 1
3591	Q3TXY0	3	8	5.5	651	70245	6.6	Putative uncharacterized protein (Fragment)
3591	A0EJG6	3	8	5.2	686	74262	6.9	Long form collapsin response mediator protein 1
3591	Q6P1J1	3	8	5.2	686	74221	6.8	Crmp1 protein
3592	A8K897	3	7	5	819	93382	5.8	cDNA FLJ78686, highly similar to sapiens nucleoporin 93kDa (NUP93), mRNA
3592	Q8N1F7	3	7	5	819	93488	5.7	Nuclear pore complex protein Nup93
3592	Q8BJ71	3	7	5	819	93281	5.7	Nuclear pore complex protein Nup93
3593	B4E2Q6	3	6	5	934	100272	6.5	cDNA FLJ54323

Table S1. Proteins identified in the OVCAR-3 tumor supernatant by two or more unique peptides.

Protein or Protein Group	Accession Number	Unique Peptide Count	Spectrum Count	Sequence Coverage (%)	Length	MolWt	pI	Descriptive Name
3593	Q6NXI6-2	3	6	3.3	1425	151403	6.9	Isoform 2 of Regulation of nuclear pre-mRNA domain-containing protein 2
3593	Q5VT52-3	3	6	3.3	1435	152875	7	Isoform 3 of Regulation of nuclear pre-mRNA domain-containing protein 2
3593	Q6NXI6	3	6	3.2	1469	156585	7.6	Regulation of nuclear pre-mRNA domain-containing protein 2
3593	Q5VT52	3	6	3.2	1461	156019	7.4	Regulation of nuclear pre-mRNA domain-containing protein 2
3594	B4DUD8	3	8	4.8	789	89015	6.7	cDNA FLJ57597
3594	E7ER68	3	8	4.8	789	88987	6.8	Uncharacterized protein
3594	Q658Y4	3	8	4.5	838	93909	6.4	Protein FAM91A1
3594	Q3UVG3	3	8	4.5	837	93464	6.4	Protein FAM91A1
3594	B6YY23	3	8	4.5	838	93969	6.4	Skeletal muscle cells re-entry induced
3595	A2AUK6	3	15	6.5	631	70645	7.2	Erythrocyte protein band 4.1-like 1
3595	Q9H4G0-4	3	15	5.8	701	78831	7.1	Isoform 4 of Band 4.1-like protein 1
3595	E9PBB6	3	15	5.6	732	82004	6.6	Uncharacterized protein
3595	A2AUK7	3	15	5.6	730	81891	6.7	Erythrocyte protein band 4.1-like 1
3595	Q9H4G0-3	3	15	5.3	772	86506	5.8	Isoform 3 of Band 4.1-like protein 1
3595	Q9H4G0-2	3	15	5.3	779	87645	5.9	Isoform 2 of Band 4.1-like protein 1
3595	E7EN13	3	15	5.3	772	86492	5.8	Uncharacterized protein
3595	B3KUB6	3	15	5.3	772	86474	5.8	cDNA FLJ39529 fis, clone PUAEN2004067, highly similar to Band 4.1-like protein 1
3595	B7Z653	3	15	5.2	794	89604	6.3	cDNA FLJ61657, highly similar to Band 4.1-like protein 1
3595	Q8C8P2	3	15	4.9	834	93532	5.9	Putative uncharacterized protein
3595	A2AUK4	3	15	4.7	880	98495	5.5	Erythrocyte protein band 4.1-like 1 (Fragment)
3595	UPI00004542F9	3	15	4.7	866	96782	5.7	band 4.1-like protein 1 isoform b
3595	Q9H4G0	3	15	4.7	881	98503	5.6	Band 4.1-like protein 1
3595	B6ZHC9	3	15	4.7	879	98285	5.7	Erythrocyte protein band 4.1-like 1
3595	A2AUK8	3	15	4.7	867	96853	5.7	Erythrocyte protein band 4.1-like 1
3595	A2AUK5	3	15	4.7	879	98315	5.7	Erythrocyte protein band 4.1-like 1
3595	E9PV14	3	15	3.9	1064	117171	5.9	Uncharacterized protein
3596	Q9JLM8-2	3	13	9.4	363	40409	9.7	Isoform 2 of Serine/threonine-protein kinase DCLK1
3596	Q8VDT3	3	13	9.4	363	40356	9.6	Dclk1 protein
3596	Q80VB6	3	13	9.4	363	40381	9.7	Dclk1 protein
3596	Q5VZY9	3	13	9.4	363	40457	9.7	Doublecortin and CaM kinase-like 1
3596	Q3UGP0	3	13	9.4	363	40351	9.7	Putative uncharacterized protein
3596	Q8BRN4	3	13	9	379	42173	9.8	Putative uncharacterized protein
3596	Q9JLM7	3	13	6.5	527	58642	9.3	Double cortin and calcium/calmodulin-dependent protein kinase-like 1, isoform CRA_c
3596	B7Z2T7	3	13	5.5	619	68722	8.7	cDNA FLJ60535, highly similar to Serine/threonine-protein kinase DCAMKL1 (EC 2.7.11.1)
3596	O15075-2	3	13	4.7	729	81100	8.7	Isoform 1 of Serine/threonine-protein kinase DCLK1
3596	A3KN70	3	13	4.6	740	82192	8.7	Dclk1 protein
3596	O15075	3	13	4.6	740	82224	8.7	Serine/threonine-protein kinase DCLK1
3596	Q9JLM8	3	13	4.5	756	84153	8.9	Serine/threonine-protein kinase DCLK1
3596	Q8CHG1	3	13	4.3	791	87403	8.8	MKIAA0369 protein (Fragment)
3597	UPI00004563E0	3	3	7.9	404	46527	5.6	UPI00004563E0 UniRef100 entry
3597	Q9NUN4	3	3	7.9	404	46540	5.6	cDNA FLJ11251 fis, clone PLACE1008813
3597	Q9NTA6	3	3	5.4	596	69295	6.2	Putative uncharacterized protein DKFZp761I2124 (Fragment)
3597	A5YP36	3	3	4.6	701	81880	6.4	SEC15-like protein 3
3597	E7EW84	3	3	4.6	701	81852	6.3	Uncharacterized protein
3597	B2RDH5	3	3	4.2	757	88527	6.5	cDNA, FLJ96612, highly similar to sapiens SEC15 (S. cerevisiae)-like (SEC15L), mRNA
3597	Q8TAG9	3	3	4	804	93722	6.2	Exocyst complex component 6
3597	Q8R313	3	3	4	802	93077	6.2	Exocyst complex component 6
3597	Q3U9D6	3	3	4	804	93287	6.2	Putative uncharacterized protein
3597	E9PHI3	3	3	4	799	93407	6	Uncharacterized protein
3597	B4DEZ1	3	3	4	796	92990	6.3	cDNA FLJ55550, highly similar to Exocyst complex component 6
3597	B3KXY5	3	3	4	799	93433	6	cDNA FLJ46316 fis, clone TESTI4041482, highly similar to Exocyst complex component 6
3597	B7Z6G5	3	3	3.9	820	95348	6.1	cDNA FLJ56022, highly similar to Exocyst complex component 6
3598	UPI0001889E45	3	4	13.4	246	28784	5.7	UPI0001889E45 UniRef100 entry
3598	Q3UUZ6	3	4	9	366	42710	6.8	Putative uncharacterized protein (Fragment)
3598	E9Q272	3	4	4.6	720	83402	5.2	Uncharacterized protein
3598	Q8BUK6	3	4	4.6	718	83218	5.2	Protein Hook log 3
3598	Q86VS8	3	4	4.6	718	83126	5.2	Protein Hook log 3
3598	Q5BKS5	3	4	4.6	720	83460	5.2	Hook log 3 (Drosophila)
3599	Q9NZQ3-5	3	5	5.4	596	64196	6.1	Isoform 5 of NCK-interacting protein with SH3 domain
3599	Q9NZQ3-4	3	5	4.9	651	70540	6.3	Isoform 4 of NCK-interacting protein with SH3 domain
3599	Q9ESJ4	3	5	4.5	714	78572	6.1	NCK-interacting protein with SH3 domain
3599	Q9NZQ3-3	3	5	4.5	715	78237	6.4	Isoform 3 of NCK-interacting protein with SH3 domain
3599	Q9NZQ3-2	3	5	4.4	722	77931	6.5	Isoform 2 of NCK-interacting protein with SH3 domain
3599	Q9NZQ3	3	5	4.4	722	78960	6.4	NCK-interacting protein with SH3 domain
3600	B7Z1Q9	3	3	4.3	912	100720	5.5	cDNA FLJ59485, highly similar to Sodium/potassium-transporting ATPase alpha-3 chain (EC 3.6.3.9)
3600	D3YYN7	3	3	4.1	947	103586	5.4	Uncharacterized protein
3600	B7Z3F8	3	3	4	983	108297	5.3	cDNA FLJ59543, highly similar to Sodium/potassium-transporting ATPase alpha-3 chain (EC 3.6.3.9)
3600	B7Z3U6	3	3	3.9	992	109550	5.3	ATPase, Na+/K+ transporting, alpha 1 polypeptide, isoform CRA_a
3600	UPI0001A471A4	3	3	3.8	1023	113000	5.5	sodium/potassium-transporting ATPase subunit alpha-1 isoform c
3600	Q6ZQ49	3	3	3.8	1022	112291	5.6	MCG142115, isoform CRA_b (Fragment)
3600	Q6PIE5	3	3	3.8	1020	112217	5.5	Sodium/potassium-transporting ATPase subunit alpha-2
3600	Q6PIC6	3	3	3.8	1013	111691	5.4	Sodium/potassium-transporting ATPase subunit alpha-3
3600	Q53ES0	3	3	3.8	1013	111778	5.4	Na+/K+-ATPase alpha 3 subunit variant (Fragment)
3600	P13637	3	3	3.8	1013	111748	5.4	Sodium/potassium-transporting ATPase subunit alpha-3
3600	P05023	3	3	3.8	1023	112896	5.5	Sodium/potassium-transporting ATPase subunit alpha-1
3600	B7Z9V4	3	3	3.8	1026	113078	5.4	cDNA, FLJ78968, highly similar to Sodium/potassium-transporting ATPase alpha-3 chain (EC 3.6.3.9)
3600	B7Z401	3	3	3.8	1024	113203	5.4	cDNA FLJ54717, highly similar to Sodium/potassium-transporting ATPase alpha-3 chain (EC 3.6.3.9)
3600	B7Z3V1	3	3	3.8	1020	112438	5.4	cDNA FLJ60077, highly similar to Sodium/potassium-transporting ATPase alpha-1 chain (EC 3.6.3.9) (Fragment)
3600	B7Z2T5	3	3	3.8	1023	112908	5.5	cDNA FLJ55427, highly similar to Sodium/potassium-transporting ATPase alpha-1 chain (EC 3.6.3.9)
3600	B7Z2T0	3	3	3.8	1026	113059	5.4	cDNA FLJ59513, highly similar to Sodium/potassium-transporting ATPase alpha-3 chain (EC 3.6.3.9)
3600	Q8VCE0	3	3	3.7	1053	115969	5.6	ATPase, Na+/K+ transporting, alpha 3 polypeptide
3600	E9PC51	3	3	3.2	1226	133317	6.1	Uncharacterized protein
3601	E9QM30	3	5	4.2	829	91392	6.3	Uncharacterized protein
3601	Q9UP83	3	5	4.2	839	92743	6.6	Conserved oligomeric Golgi complex subunit 5
3601	Q8C0L8	3	5	4.2	829	91601	6.3	Conserved oligomeric Golgi complex subunit 5
3601	Q9UP83-2	3	5	4.1	860	94901	6.6	Isoform 2 of Conserved oligomeric Golgi complex subunit 5
3602	Q86YB2	3	4	3.9	1220	139314	8.4	DEAH (Asp-Glu-Ala-His) box polypeptide 8

Table S1. Proteins identified in the OVCAR-3 tumor supernatant by two or more unique peptides.

Protein or Protein Group	Accession Number	Unique Peptide Count	Spectrum Count	Sequence Coverage (%)	Length	MolWt	pI	Descriptive Name
3602	Q86X36	3	4	3.9	1220	139287	8.1	DEAH (Asp-Glu-Ala-His) box polypeptide 8
3602	Q14562	3	4	3.9	1220	139314	8.3	ATP-dependent RNA helicase DHX8
3602	A8K6J9	3	4	3.9	1220	139384	8.4	cDNA FLJ76879, highly similar to sapiens DEAH (Asp-Glu-Ala-His) box polypeptide 8 (DHX8), mRNA
3602	A2A4P0	3	4	3.8	1244	142571	8.1	ATP-dependent RNA helicase DHX8
3602	Q3UQQ4	3	4	3.8	1244	142555	8.1	Putative uncharacterized protein
3602	Q5DTN5	3	4	3.7	1264	144923	8.1	MKIAA4096 protein (Fragment)
3603	A8MYY5	3	4	3.4	893	100680	6.5	Uncharacterized protein
3603	Q05397-2	3	4	3.4	879	99358	6.4	Isoform 2 of Focal adhesion kinase 1
3603	C9JYH6	3	4	3.4	877	99107	6.4	Uncharacterized protein
3603	Q59GN8	3	4	3.1	975	110234	6.2	PTK2 protein tyrosine kinase 2 isoform b variant (Fragment)
3603	Q8IYN9	3	4	3	1006	114253	6.8	PTK2 protein
3603	Q59GM6	3	4	3	1007	114157	6.6	PTK2 protein tyrosine kinase 2 isoform b variant (Fragment)
3603	UPI0001E8F580	3	4	2.9	1017	115563	6.8	UPI0001E8F580 UniRef100 entry
3603	Q05397	3	4	2.9	1052	119233	6.6	Focal adhesion kinase 1
3603	P34152-3	3	4	2.9	1052	119243	6.6	Isoform 3 of Focal adhesion kinase 1
3603	UPI0001E8F57E	3	4	2.8	1065	120900	6.8	focal adhesion kinase 1 isoform c
3603	Q658W2	3	4	2.8	1074	121721	6.6	Putative uncharacterized protein DKFZp666O0110
3603	P34152-4	3	4	2.8	1055	119682	6.7	Isoform 4 of Focal adhesion kinase 1
3603	P34152-2	3	4	2.8	1083	122774	6.7	Isoform 2 of Focal adhesion kinase 1
3603	P34152	3	4	2.8	1090	123537	6.7	Focal adhesion kinase 1
3603	E9QP72	3	4	2.8	1085	122960	6.7	Uncharacterized protein
3603	B4E2N6	3	4	2.8	1065	120857	6.7	cDNA FLJ61553, highly similar to Focal adhesion kinase 1 (EC 2.7.10.2)
3603	E9QMQ8	3	4	2.7	1092	123723	6.7	Uncharacterized protein
3604	B3KV73	3	3	3.3	1075	121132	8.6	DNA-directed RNA polymerase
3604	Q9NW08	3	3	3.2	1133	127785	8.5	DNA-directed RNA polymerase III subunit RPC2
3604	Q7Z3R8	3	3	3.2	1133	127819	8.5	DNA-directed RNA polymerase
3604	P59470	3	3	3.2	1133	127715	8.5	DNA-directed RNA polymerase III subunit RPC2
3605	B7Z5S1	3	4	2.8	1302	148739	8.1	cDNA FLJ61699, highly similar to sapiens androgen-induced proliferation inhibitor (APRIN), transcript variant 1, mRNA
3605	Q9NTI5	3	4	2.6	1447	164666	8.5	Sister chromatid cohesion protein PDS5 log B
3605	Q4VA53-3	3	4	2.6	1448	164673	8.5	Isoform 3 of Sister chromatid cohesion protein PDS5 log B
3605	Q4VA53	3	4	2.6	1446	164418	8.5	Sister chromatid cohesion protein PDS5 log B
3605	E9QNG8	3	4	2.6	1449	164761	8.5	Uncharacterized protein
3605	E7ENA8	3	4	2.6	1448	164753	8.5	Uncharacterized protein
3606	A8K0J3	3	3	2.6	898	105331	7.4	cDNA FLJ76732, highly similar to sapiens TAO kinase 3 (TAOK3), mRNA
3606	Q9H2K8	3	3	2.6	898	105406	7.3	Serine/threonine-protein kinase TAO3
3606	Q8BYC6	3	3	2.6	898	105336	7.3	Serine/threonine-protein kinase TAO3
3606	B2RY27	3	3	2.6	898	105364	7.3	MCG125789, isoform CRA_a
3607	Q3US09	3	4	4.6	921	105537	6.5	Putative uncharacterized protein (Fragment)
3607	B1AZI6	3	4	2.6	1594	182772	8.4	THO complex subunit 2
3607	Q8NI27	3	4	2.6	1593	182774	8.4	THO complex subunit 2
3608	E9QMB0	3	3	2.7	1163	132351	6.9	Uncharacterized protein
3608	B7Z8Y4	3	3	2.6	1192	135995	7	cDNA FLJ61698, highly similar to Golgi apparatus protein 1
3608	Q92896	3	3	2.6	1179	134552	6.9	Golgi apparatus protein 1
3608	Q61543	3	3	2.6	1175	133734	6.8	Golgi apparatus protein 1
3609	B4E0F0	3	3	2.5	1110	119516	8.2	cDNA FLJ53428, highly similar to Disco-interacting protein 2 log A
3609	UPI0001989312	3	3	2.5	1110	119467	8.2	disco-interacting protein 2 log A isoform e
3609	Q8BWT5	3	3	1.8	1523	165273	7.7	Disco-interacting protein 2 log A
3609	Q14689	3	3	1.8	1571	170368	8	Disco-interacting protein 2 log A
3609	E9QP22	3	3	1.8	1562	169519	7.8	Uncharacterized protein
3609	E9PER1	3	3	1.8	1572	170439	8	Uncharacterized protein
3609	E0CXM7	3	3	1.8	1523	165254	7.6	Uncharacterized protein
3610	C9JRP8	3	25	2.2	1309	146120	5.8	Uncharacterized protein
3610	Q9H254-2	3	25	2.2	1309	146168	5.8	Isoform 2 of Spectrin beta chain, brain 3
3610	Q71S06	3	25	1.4	2002	225564	5.7	Beta spectrin IV
3610	Q9H254-4	3	25	1.3	2154	243422	5.7	Isoform 4 of Spectrin beta chain, brain 3
3610	Q9H254	3	25	1.1	2564	288984	6	Spectrin beta chain, brain 3
3610	Q91ZE6	3	25	1.1	2555	288133	6	Beta-spectrin 4
3610	Q8VIE5	3	25	1.1	2561	288807	6	BetaIV-spectrin sigma1
3610	Q71S07	3	25	1.1	2564	289060	6.1	Non-erythrocytic beta-spectrin 4
3610	E9PX29	3	25	1.1	2561	288822	6	Uncharacterized protein
3611	O14646	3	4	2	1710	196687	7.1	Chromodomain-helicase-DNA-binding protein 1
3611	Q14BJ0	3	4	2	1711	196384	7.4	Chromodomain helicase DNA binding protein 1
3611	P40201	3	4	2	1711	196410	7.4	Chromodomain-helicase-DNA-binding protein 1
3611	O14646-2	3	4	2	1709	196589	7.1	Isoform 2 of Chromodomain-helicase-DNA-binding protein 1
3612	Q9H6Y6	3	5	2.9	963	111759	6.3	cDNA: FLJ21683 fis, clone COL09335
3612	P21271	3	5	1.5	1818	210569	7.1	Myosin-Vb
3612	UPI000154773E	3	5	1.5	1844	213412	6.9	UPI000154773E UniRef100 entry
3612	UPI00002160ED	3	5	1.5	1818	210545	7.1	myosin-Vb
3612	Q9ULV0	3	5	1.5	1848	213671	7.2	Myosin-Vb
3612	P21271-3	3	5	1.5	1844	213436	7	Isoform 3 of Myosin-Vb
3613	UPI00001946D0	3	46	85	140	14932	8.8	hemoglobin alpha, adult chain 1
3613	Q91VB8	3	46	83.8	142	15112	8.2	Alpha globin 1
3613	Q9CY10	3	46	83.8	142	15202	8.8	Putative uncharacterized protein
3614	P57780	3	72	81.6	912	104977	5.4	Alpha-actinin-4
3615	P52480-2	3	9	75.9	531	57985	7.1	Isoform M1 of Pyruvate kinase isozymes M1/M2
3616	P15532	3	17	75.7	152	17208	7.4	Nucleoside diphosphate kinase A
3617	P27005	3	23	73	89	10295	5.7	Protein S100-A8
3618	P34884	3	21	58.3	115	12504	7.3	Macrophage migration inhibitory factor
3619	Q9DAS9	3	5	56.9	72	7997	9	Guanine nucleotide-binding protein G(I)/G(S)/G(O) subunit gamma-12
3620	D3YXL3	3	5	51.4	105	11697	6.1	Uncharacterized protein
3621	O54962	3	29	48.3	89	10103	6.1	Barrier-to-autointegration factor
3622	O08997	3	6	47.1	68	7338	6.5	Copper transport protein ATOX1
3623	Q9ERL7	3	8	45.8	142	16748	5.7	Glia maturation factor gamma
3623	UPI0001E6B834	3	8	39.4	165	19320	6.8	PREDICTED: glia maturation factor gamma-like
3624	Q3V4B5	3	8	41.4	87	9795	5.4	COMM domain-containing protein 6
3625	Q9Z121	3	10	41.2	97	11017	8.4	C-C motif chemokine 8
3626	D3YU37	3	33	40.9	115	12822	9.2	Uncharacterized protein
3626	UPI0000604660	3	33	40.9	115	12827	9.2	PREDICTED: 60S ribosomal protein L30-like
3627	Q8BFZ3	3	19	40.2	376	42004	5.5	Beta-actin-like protein 2
3628	Q9D7S7-2	3	6	39.7	121	14339	9.4	Isoform 2 of 60S ribosomal protein L22-like 1
3628	Q9D7S7	3	6	39.3	122	14467	9.4	60S ribosomal protein L22-like 1
3629	P56375	3	5	39.2	97	10895	8.7	Acylphosphatase-2
3630	Q9DAS3	3	6	41.5	106	11625	5.4	Putative uncharacterized protein
3630	A1E5T5	3	6	38.9	113	12482	5.8	Tctex-1
3630	P51807	3	6	38.9	113	12483	5.1	Dynein light chain Tctex-type 1
3631	P61028	3	8	37.7	207	23603	9.1	Ras-related protein Rab-8B
3632	A7YL62	3	12	37.3	102	11291	7.2	Apolipoprotein A-II
3632	Q3UKX6	3	12	37.3	102	11309	7.2	Apolipoprotein A-II, isoform CRA_a

Table S1. Proteins identified in the OVCAR-3 tumor supernatant by two or more unique peptides.

Protein or Protein Group	Accession Number	Unique Peptide Count	Spectrum Count	Sequence Coverage (%)	Length	MolWt	pI	Descriptive Name
3633	Q8VC41	3	4	37	413	45995	5.4	Serpina1d protein
3634	Q3U8P1	3	4	36.6	131	15296	7.3	Putative uncharacterized protein
3634	Q9ER81	3	4	36.6	131	15278	7.3	15kD interferon alpha responsive protein
3634	Q9D178	3	4	36.6	131	15306	7.3	Putative uncharacterized protein
3634	Q3UAA2	3	4	36.6	131	15250	7.3	Putative uncharacterized protein
3635	Q9D7F5	3	12	36.1	147	16619	7.3	Ubiquitin carrier protein
3636	Q8BHA3-2	3	6	28.3	166	18051	8.3	Isoform 2 of Probable D-tyrosyl-tRNA(Tyr) deacylase 2
3636	Q8BHA3	3	6	28	168	18236	7.9	Probable D-tyrosyl-tRNA(Tyr) deacylase 2
3637	P09041	3	8	27.6	417	44883	6.8	Phosphoglycerate kinase 2
3638	Q08879-2	3	13	27.6	685	75284	5.3	Isoform C of Fibulin-1
3639	O88593	3	5	26.4	182	20489	7.5	Peptidoglycan recognition protein 1
3640	O88851	3	6	25.8	186	20912	6	Putative hydrolase RBBP9
3640	Q80YU9	3	6	25.8	186	20913	6	Retinoblastoma binding protein 9
3641	UPI0000195B3B	3	7	25.3	83	9512	4.5	PREDICTED: small nuclear ribonucleoprotein F
3642	O35658	3	5	25.2	278	31013	4.9	Complement component 1 Q subcomponent-binding protein, mitochondrial
3642	Q8R5L1	3	5	25.1	279	31025	4.9	Complement component 1, q subcomponent binding protein
3643	P01831	3	12	24.1	162	18080	9	Thy-1 membrane glycoprotein
3643	Q53YX2	3	12	24.1	162	18108	9.2	CD90.1
3644	P11404	3	4	23.3	133	14819	6.6	Fatty acid-binding protein, heart
3645	Q9D0J8	3	18	22.8	101	11430	4.2	Parathymosin
3645	P04550	3	18	22.5	102	11559	4.2	Parathymosin
3645	UPI00015DEFD8	3	18	16.5	139	15347	4.6	parathymosin
3646	Q9DCT5	3	4	22.3	211	23159	7.3	Stromal cell-derived factor 2
3647	Q99JT9	3	16	21.8	179	21524	5.5	1,2-dihydroxy-3-keto-5-methylthiopentene dioxygenase
3648	D3Z5G7	3	6	21.7	567	62197	5.2	Uncharacterized protein
3649	Q91XV3	3	3	21.7	226	22087	4.5	Brain acid soluble protein 1
3650	Q61425	3	3	21.3	314	34464	8.6	Hydroxyacyl-coenzyme A dehydrogenase, mitochondrial
3651	P20801	3	3	21.2	160	18110	4.2	Troponin C, skeletal muscle
3651	Q3UZY7	3	3	21.2	160	18134	4.2	Putative uncharacterized protein
3652	P56376	3	8	32.3	99	11241	9.1	Acylphosphatase-1
3652	E9QJT5	3	8	20.4	157	17344	9.1	Uncharacterized protein
3653	P09671	3	7	19.4	222	24603	8.6	Superoxide dismutase [Mn], mitochondrial
3654	Q9Z0Y1	3	3	19.4	186	20978	6	Dynactin subunit 3
3655	Q6P3D0	3	3	19	195	21825	7.1	U8 snoRNA-decapping enzyme
3656	Q505B7-2	3	5	21.2	146	17207	4.7	Isoform 2 of Protein archease
3656	B2KGA7	3	5	18.6	167	19580	4.6	Zinc finger and BTB domain containing 8 opposite strand
3656	Q505B7	3	5	18.5	168	19727	4.6	Protein archease
3657	A2RTV1	3	3	18.1	409	45738	6.6	Serpina3h protein
3657	Q3U1B7	3	3	18.1	409	45738	6.7	Putative uncharacterized protein
3658	B1ARU4	3	5	17.8	7353	831666	5.4	Microtubule-actin crosslinking factor 1
3658	UPI0000609071	3	5	17.8	7355	831893	5.4	microtubule-actin cross-linking factor 1 isoform 1
3659	E9Q5X5	3	8	17.6	204	21940	4.6	Uncharacterized protein
3659	Q07797	3	8	6.2	577	64491	5.1	Galectin-3-binding protein
3660	O08692	3	5	17.4	167	19332	5.3	Myeloid bactenecin (F1)
3660	Q61903	3	5	17.4	167	19331	5.5	Myeloid secondary granule protein
3661	Q3TJB4	3	8	16.6	368	41549	5.7	Putative uncharacterized protein
3661	Q62419	3	8	16.6	368	41518	5.7	Endophilin-A2
3661	Q3U3C4	3	8	16.6	368	41532	5.7	Putative uncharacterized protein
3662	P26645	3	5	16.5	309	29661	4.3	Myristoylated alanine-rich C-kinase substrate
3663	Q9QZZ6	3	5	16.4	201	23995	4.8	Dermatopontin
3664	P13412	3	4	17	182	21357	8.5	Troponin I, fast skeletal muscle
3664	A2A6J8	3	4	16.3	190	22201	8.9	Troponin I, skeletal, fast 2 (Fragment)
3665	P04939	3	4	16.3	184	21465	4.8	Major urinary protein 3
3665	Q3KQQ2	3	4	16.3	184	21417	4.8	MCG15829, isoform CRA_a
3666	Q9CY62	3	3	15.8	165	19101	6	E3 ubiquitin-protein ligase RNF181
3667	D3Z380	3	3	15.6	205	23456	7	Uncharacterized protein
3667	O55060	3	3	13.3	240	27586	6.4	Thiopurine S-methyltransferase
3668	Q9CQG4	3	18	15.7	185	21087	8.1	Putative uncharacterized protein
3668	Q5QGU0	3	18	15.3	189	21490	8.4	Receptor expression enhancing protein 5
3669	Q05CZ9	3	5	15.3	248	26951	8.1	Esam1 protein
3669	D3Z5Y0	3	5	15.1	252	27217	8.5	Uncharacterized protein
3669	Q925F2	3	5	9.6	394	41810	9.3	Endothelial cell-selective adhesion molecule
3670	Q3V3M3	3	4	17	229	25801	6.8	Putative uncharacterized protein (Fragment)
3670	P97376	3	4	15.1	258	29127	8.9	Protein FRG1
3671	P49935	3	9	15	333	37184	8.4	Cathepsin H
3671	Q922Q7	3	9	15	333	37155	8.3	Cathepsin H
3671	Q3UCD6	3	9	15	333	37170	8.4	Cathepsin H, isoform CRA_b
3672	Q543M9	3	4	14.8	244	27392	9.2	MCG14605, isoform CRA_c
3672	Q99PW4	3	4	14.8	244	27394	9.2	TP53-regulating kinase
3672	Q5U452	3	4	14.8	244	27436	9.1	MCG14616
3673	B1AQD1	3	3	14.6	198	22128	7.2	RAB34, member of RAS oncogene family (Fragment)
3673	B1AQD3	3	3	11.6	251	28314	8.1	RAB34, member of RAS oncogene family
3673	Q64008	3	3	11.2	259	29101	8.3	Ras-related protein Rab-34
3673	Q0PD20	3	3	11.2	259	29129	8.3	RAB34, member of RAS oncogene family
3673	B1AQD4	3	3	10.9	265	29989	8.7	RAB34, member of RAS oncogene family (Fragment)
3673	UPI0001F788F3	3	3	6.5	444	48108	9.6	UPI0001F788F3 UniRef100 entry
3673	Q3TV85	3	3	6.5	445	48196	9.5	Putative uncharacterized protein (Fragment)
3674	Q9D0A3	3	3	14.6	226	25193	5.2	UPF0552 protein C15orf38 homolog
3675	E9QLT0	3	3	13.9	267	29395	5.6	Uncharacterized protein
3675	UPI00015D7B58	3	3	13.4	277	30450	6	putative hydroxypyruvate isomerase
3675	Q8R1F5	3	3	13.4	277	30449	6.4	Putative hydroxypyruvate isomerase
3676	UPI0001E6B56D	3	14	17.6	125	13562	9.9	PREDICTED: 60S ribosomal protein L29-like
3676	UPI00015DE8C3	3	14	13.9	158	17446	11.4	UPI00015DE8C3 UniRef100 entry
3676	D3YTZ1	3	14	13.8	160	17557	11.5	MCG114459
3676	UPI0001553466	3	14	13.8	160	17573	11.8	PREDICTED: 60S ribosomal protein L29-like
3676	UPI0000511B8C	3	14	13.8	160	17617	11.8	PREDICTED: 60S ribosomal protein L29-like
3676	P47915	3	14	13.8	160	17587	11.8	60S ribosomal protein L29
3676	D3Z1N9	3	14	13.8	160	17647	11.8	MCG9889
3676	D3YU34	3	14	13.8	160	17617	11.8	MCG142394
3676	UPI0001E6B983	3	14	13.1	168	18263	11.5	PREDICTED: 60S ribosomal protein L29-like
3676	UPI0001E6B809	3	14	9.8	224	24305	10.9	PREDICTED: 60S ribosomal protein L29-like
3676	UPI0001E6B927	3	14	8.4	263	28520	10.8	PREDICTED: hypothetical protein LOC670211
3677	P14106	3	5	13.8	253	26816	8.4	Complement C1q subcomponent subunit B
3677	Q3US82	3	5	13.8	253	26707	8.1	Putative uncharacterized protein
3677	Q3U793	3	5	13.8	253	26717	8.1	Complement component 1, q subcomponent, beta polypeptide
3677	Q3U6I5	3	5	13.8	253	26751	8.1	Putative uncharacterized protein
3678	UPI0001E6B85A	3	47	13.7	730	81772	9	PREDICTED: splicing factor U2AF 65 kDa subunit-like
3679	E9Q6X0	3	4	13	284	32265	5.3	Uncharacterized protein
3679	Q8R001-2	3	4	11.7	317	36070	6.3	Isoform 2 of Microtubule-associated protein RP/EB family member 2
3679	Q8R001	3	4	11.3	326	36946	5.4	Microtubule-associated protein RP/EB family member 2
3680	P39039	3	3	13	239	25396	7.6	Mannose-binding protein A
3681	E9PUZ8	3	5	12.9	256	27845	8	Uncharacterized protein

Table S1. Proteins identified in the OVCAR-3 tumor supernatant by two or more unique peptides.

Protein or Protein Group	Accession Number	Unique Peptide Count	Spectrum Count	Sequence Coverage (%)	Length	MolWt	pI	Descriptive Name
3681	Q80SX2	3	5	7.7	426	46647	6.4	C4b-binding protein alpha-chain
3681	Q91X48	3	5	7	469	51524	7.1	Complement component 4 binding protein
3681	Q3UW06	3	5	7	469	51623	7.3	Putative uncharacterized protein
3681	P08607	3	5	7	469	51552	7.1	C4b-binding protein
3682	Q641P0	3	15	12.7	418	47580	6	Actin-related protein 3B
3683	Q8BN05	3	3	12.5	408	43893	8.4	Putative uncharacterized protein (Fragment)
3683	Q9R118	3	3	10.6	480	51247	7.3	Serine protease HTRA1
3683	Q9QZK6	3	3	10.6	480	51214	7.7	HtrA serine peptidase 1, isoform CRA_b
3683	Q91WS3	3	3	10.6	480	51213	7.8	HtrA serine peptidase 1
3684	P70274	3	9	12.1	380	41205	7.1	Selenoprotein P
3685	A2A4I0	3	5	11.7	581	64698	5.6	FK506 binding protein 10
3685	Q61576	3	5	11.7	581	64669	5.6	Peptidyl-prolyl cis-trans isomerase FKBP10
3685	Q3UNJ3	3	5	11.7	580	64640	5.6	Putative uncharacterized protein
3686	P70460	3	3	11.5	375	39667	8.5	Vasodilator-stimulated phosphoprotein
3687	Q6ZPV5	3	8	11.5	426	47169	5.5	MKIAA1235 protein (Fragment)
3687	UPI0001F78AEB	3	8	11.3	433	48026	5.8	AT rich interactive domain 1B (Swi1 like)
3688	Q921D0	3	3	12.3	301	33758	5.6	Anxa8 protein
3688	O35640	3	3	11.3	327	36843	5.8	Annexin A8
3688	Q8K2N9	3	3	11.3	327	36724	5.9	Annexin A8
3689	A7LNR1	3	5	12.4	307	33049	7.4	CD93 antigen (Fragment)
3689	A7LNQ7	3	5	11	346	37378	6.6	CD93 antigen (Fragment)
3689	A7LNQ8	3	5	11	346	37355	6.5	CD93 antigen (Fragment)
3689	O89103	3	5	5.9	644	69355	5.1	Complement component C1q receptor
3690	P28654	3	4	11	354	39809	8.7	Decorin
3690	Q3TSV1	3	4	11	354	39775	8.7	Putative uncharacterized protein
3691	B8JJN2	3	6	10.9	623	69685	6.9	Complement component 2 (Within H-2S)
3691	P21180-2	3	6	9	753	83998	7.4	Isoform Short of Complement C2
3691	P21180	3	6	8.9	760	84726	7.5	Complement C2
3691	O70350	3	6	8.9	760	84741	7.6	C2
3692	P47199	3	4	10	331	35269	8.1	Quinone oxidoreductase
3692	Q80XR3	3	4	10	331	35368	8.3	Crystallin, zeta
3693	Q9Z247	3	3	9.6	570	62996	5.2	Peptidyl-prolyl cis-trans isomerase FKBP9
3694	O70172	3	3	8.9	405	46152	7	Phosphatidylinositol-5-phosphate 4-kinase type-2 alpha
3694	Q9CZS7	3	3	8.9	405	46091	7.1	Putative uncharacterized protein
3695	E9Q7B0	3	5	8.6	454	51754	6.7	Uncharacterized protein
3695	Q3TTT2	3	5	8.6	454	51755	6.7	Putative uncharacterized protein
3695	Q60715-2	3	5	7.3	534	60886	5.8	Isoform 2 of Prolyl 4-hydroxylase subunit alpha-1
3695	Q60715	3	5	7.3	534	60910	5.9	Prolyl 4-hydroxylase subunit alpha-1
3695	Q3TN84	3	5	7.3	534	60924	5.9	Putative uncharacterized protein
3695	Q3UF16	3	5	7	561	63809	5.9	Putative uncharacterized protein
3696	O88947	3	3	8.5	481	54018	5.7	Coagulation factor X
3696	Q4FJS7	3	3	8.5	481	54004	5.7	F10 protein
3697	Q3TW96	3	4	8.5	507	56614	5.4	UDP-N-acetylhexosamine pyrophosphorylase-like protein 1
3697	Q8BVK3	3	4	8.4	510	56873	5.4	Putative uncharacterized protein (Fragment)
3698	Q9D281	3	6	8.4	569	61013	4.6	Protein Noxp20
3699	Q9JJN5	3	4	8.1	457	51845	8.3	Carboxypeptidase N catalytic chain
3700	E9QPH2	3	7	8	650	72287	4.6	Uncharacterized protein
3700	Q3UYK7	3	7	8	650	72300	4.6	Putative uncharacterized protein
3700	Q3TYD5	3	7	8	650	72342	4.6	Putative uncharacterized protein
3700	P70663	3	7	8	650	72314	4.6	SPARC-like protein 1
3701	O55137	3	3	7.2	419	46136	6.6	Acyl-coenzyme A thioesterase 1
3701	Q99L15	3	3	7.1	420	46248	6.6	Acot1 protein (Fragment)
3701	Q9QYR9	3	3	6.6	453	49652	7.4	Acyl-coenzyme A thioesterase 2, mitochondrial
3701	Q6P2K2	3	3	6.6	453	49641	7.6	Acyl-CoA thioesterase 2
3701	Q3T9C9	3	3	6.6	453	49657	7.4	MCG4931
3702	Q9D2L5	3	6	7.1	764	86963	6.7	Inactive carboxypeptidase-like protein X2
3703	A3KMP2-2	3	4	11.5	279	31589	6.3	Isoform 2 of Tetratricopeptide repeat protein 38
3703	A3KMP2	3	4	6.9	465	52224	6.3	Tetratricopeptide repeat protein 38
3704	P00687	3	3	6.7	511	57624	7	Alpha-amylase 1
3704	Q921Y7	3	3	6.7	511	57644	7	Amy1 protein
3705	Q8R480	3	9	5.8	656	74776	5.6	Nuclear pore complex protein Nup85
3706	P08071	3	3	5.7	707	77866	8.6	Lactotransferrin
3706	Q8CBA0	3	3	5.7	707	77838	8.5	Putative uncharacterized protein
3706	Q3UBV2	3	3	5.7	707	77886	8.6	Putative uncharacterized protein
3706	Q3TP24	3	3	5.7	707	77872	8.5	Putative uncharacterized protein
3707	Q9ET02	3	4	6.1	770	86065	6.5	Ube1l (Fragment)
3707	E9Q2Z8	3	4	5.3	879	97331	6.1	Uncharacterized protein
3707	Q8BTN4	3	4	5.2	905	100434	6.1	Putative uncharacterized protein
3707	Q9DBK7	3	4	4.8	977	108679	6	MCG18845, isoform CRA_d
3708	Q3V1T4-3	3	3	7	560	64102	4.9	Isoform 3 of Prolyl 3-hydroxylase 1
3708	Q3V1T4	3	3	5.3	739	83651	5.1	Prolyl 3-hydroxylase 1
3708	A2A7Q5	3	3	5.2	748	84479	5.2	Leprecan 1
3708	A6PW84	3	3	5.2	746	84473	5.2	Leprecan 1
3709	Q3UW53	3	3	5.2	926	102649	4.8	Protein Niban
3710	E9PWS1	3	4	4.3	818	92908	8.5	Uncharacterized protein
3710	Q8BUT2	3	4	4.3	819	92863	8.2	Putative uncharacterized protein
3710	Q8BZ98-2	3	4	4.1	859	96804	8.2	Isoform 2 of Dynamin-3
3710	Q8BZ98	3	4	4.1	863	97190	8.4	Dynamin-3
3710	E9QLL2	3	4	4.1	863	97274	8.6	Uncharacterized protein
3711	Q3TAZ7	3	4	6.9	551	61648	6.4	Putative uncharacterized protein
3711	Q3U4N8	3	4	4.1	922	103724	6.7	Tyrosine-protein kinase receptor (Fragment)
3711	Q3U1X4	3	4	4	945	105535	6.6	Tyrosine-protein kinase receptor (Fragment)
3711	P09581	3	4	3.9	977	109179	6.2	Macrophage colony-stimulating factor 1 receptor
3711	Q6NXV8	3	4	3.9	977	109252	6.2	Tyrosine-protein kinase receptor
3711	Q3UKC6	3	4	3.9	977	109192	6.2	Tyrosine-protein kinase receptor
3711	Q3U3W0	3	4	3.9	977	109140	6.1	Tyrosine-protein kinase receptor
3711	Q3U210	3	4	3.9	977	109061	6.3	Tyrosine-protein kinase receptor
3711	Q3U1Y3	3	4	3.9	977	109110	6.1	Tyrosine-protein kinase receptor
3712	D3YXK2	3	11	3.8	937	105104	5.4	MCG127409
3713	Q5JC28	3	3	4.3	793	86678	4.7	Epidermal growth factor receptor pathway substrate 15 isoform B
3713	P42567	3	3	3.8	897	98471	4.6	Epidermal growth factor receptor substrate 15
3714	E9QMR4	3	3	3	1578	173945	7.2	Uncharacterized protein
3714	Q6A065	3	3	3	1588	175049	7.2	Centrosomal protein of 170 kDa
3715	Q62470-2	3	3	2.6	1057	117357	6.8	Isoform Alpha-3B of Integrin alpha-3
3715	Q62470	3	3	2.6	1053	116745	6.6	Integrin alpha-3
3715	Q5SWA8	3	3	2.5	1068	118883	6.8	Integrin alpha 3
3716	Q8CIH5	3	3	2.5	1265	147592	6.8	1-phosphatidylinositol-4,5-bisphosphate phosphodiesterase gamma-2
3717	B9EKJ7	3	5	2.2	1720	181825	4.9	Tankyrase 1 binding protein 1
3717	P58871	3	5	2.2	1720	181824	4.9	182 kDa tankyrase-1-binding protein
3718	E9QPI0	3	3	2.1	2038	219891	6.8	Uncharacterized protein
3718	Q5PRF0	3	3	2.1	2038	219819	6.8	HEAT repeat-containing protein 5A
3719	Q8C3Y4	3	3	1.9	2207	250356	6	Kinetochore-associated protein 1

Table S1. Proteins identified in the OVCAR-3 tumor supernatant by two or more unique peptides.

Protein or Protein Group	Accession Number	Unique Peptide Count	Spectrum Count	Sequence Coverage (%)	Length	MolWt	pI	Descriptive Name
3720	D3YXG0	3	3	0.6	5634	611568	6.6	Uncharacterized protein
3720	D3Z2Q7	3	3	0.6	5517	598265	6.6	Uncharacterized protein
3721	E9PUM2	3	5	0.5	8799	1010026	5.6	Uncharacterized protein
3721	Q6ZWR6-4	3	5	0.5	8797	1009691	5.6	Isoform 4 of Nesprin-1
3721	Q6ZWR6	3	5	0.5	8799	1009946	5.6	Nesprin-1
3722	P24666-2	3	21	69	158	17977	6.9	Isoform 2 of Low molecular weight phosphotyrosine protein phosphatase
3723	P30047	3	8	69	84	9698	6.5	GTP cyclohydrolase 1 feedback regulatory protein
3723	Q53F59	3	8	69	84	9679	6.3	GTP cyclohydrolase I feedback regulatory protein variant (Fragment)
3724	E5RI56	3	92	63.7	91	10271	4.4	Uncharacterized protein
3725	Q13542	3	5	61.7	120	12939	6.7	Eukaryotic translation initiation factor 4E-binding protein 2
3725	Q6FG68	3	5	61.7	120	12969	6.7	EIF4EBP2 protein (Fragment)
3726	O75506	3	9	59.2	76	8544	4.4	Heat shock factor-binding protein 1
3727	Q13541	3	5	58.5	118	12580	5.5	Eukaryotic translation initiation factor 4E-binding protein 1
3728	Q9UBI6	3	7	56.9	72	8006	9	Guanine nucleotide-binding protein G(I)/G(S)/G(O) subunit gamma-12
3729	Q9H3K6	3	55	51.2	86	10117	6.5	BolA-like protein 2
3730	Q9P086	3	7	49.6	117	13129	5.9	Mediator of RNA polymerase II transcription subunit 11
3731	Q9P1F3	3	10	46.9	81	9056	6.3	Costars family protein C6orf115
3732	A8MUW3	3	6	45.3	117	12225	4.9	Uncharacterized protein
3732	Q9NR33	3	6	45.3	117	12209	4.9	DNA polymerase epsilon subunit 4
3733	Q15004	3	6	45	111	11986	9.8	PCNA-associated factor
3734	P01040	3	4	42.9	98	11006	5.5	Cystatin-A
3735	Q9NS69	3	7	42.3	142	15522	4.3	Mitochondrial import receptor subunit TOM22 log
3736	Q5HYD9	3	4	51.4	107	11873	4.8	Putative uncharacterized protein DKFZp686M0619 (Fragment)
3736	D6RFH4	3	4	42	131	14846	6.2	Uncharacterized protein
3736	O43169	3	4	37.7	146	16332	5	Cytochrome b5 type B
3737	C9JHF5	3	3	40.6	138	15627	5.2	Uncharacterized protein
3737	Q9GZY8-4	3	3	25.7	218	25067	5.6	Isoform 4 of Mitochondrial fission factor
3737	Q9GZY8-5	3	3	23.5	238	27105	6.6	Isoform 5 of Mitochondrial fission factor
3737	Q9GZY8-3	3	3	23	243	27964	7.3	Isoform 3 of Mitochondrial fission factor
3737	Q9GZY8-2	3	3	19.2	291	32973	7.5	Isoform 2 of Mitochondrial fission factor
3737	Q9GZY8	3	3	16.4	342	38465	9	Mitochondrial fission factor
3738	O43678	3	7	40.4	99	10922	9.6	NADH dehydrogenase [ubiquinone] 1 alpha subcomplex subunit 2
3739	P51808	3	7	52.6	116	13062	5.7	Dynein light chain Tctex-type 3
3739	A6NGJ0	3	7	40.1	152	16979	5.9	Uncharacterized protein
3740	C9JYQ9	3	4	39.7	121	14478	9.4	Uncharacterized protein
3740	Q6P5R6	3	4	39.3	122	14606	9.4	60S ribosomal protein L22-like 1
3740	UPI0000EE2292	3	4	33.8	142	16900	9.2	UPI0000EE2292 UniRef100 entry
3741	Q6TXQ4	3	5	39.3	107	12104	10.6	Histone H3 (Fragment)
3742	Q14657	3	30	37.8	143	14804	8.6	L antigen family member 3
3743	Q92930	3	9	37.7	207	23584	9.1	Ras-related protein Rab-8B
3744	Q9H496	3	5	36.6	131	15347	7.3	IFRG15 protein
3745	B3KQA0	3	14	36.2	127	14550	4.6	cDNA FLJ90015 fis, clone HEMBA1000634, highly similar to sapiens Mof4 family associated protein 1 (MRFAP1), mRNA
3745	Q9Y605	3	14	36.2	127	14650	4.7	MORF4 family-associated protein 1
3746	Q8HZE3	3	10	43.8	96	11265	6.5	Beta-2 microglobulin (Fragment)
3746	P61769	3	10	35.3	119	13715	6.5	Beta-2-microglobulin
3746	B4E0X1	3	10	34.4	122	13946	7.5	cDNA FLJ57067, highly similar to Beta-2-microglobulin
3747	Q96E39	3	8	33.6	390	42142	9.9	RNA-binding motif protein X-linked-like 1 (Kynurenine aminotransferase III) (Protein RBM1)
3748	Q8WW01	3	9	33.3	171	18641	4.6	tRNA-splicing endonuclease subunit Sen15
3749	Q15828	3	6	32.9	149	16511	8.1	Cystatin-M
3749	Q6IBD2	3	6	32.9	149	16493	8.1	CST6 protein
3750	Q53GA4	3	3	32.9	152	17092	9.2	Pleckstrin logy-like domain family A member 2
3751	C9JLW8	3	4	32	97	10920	9.4	Protein FAM195B
3752	Q9Y6H1	3	8	31.1	151	15513	9.2	Coiled-coil-helix-coiled-coil-helix domain-containing protein 2, mitochondrial
3753	Q4TZM4	3	79	31.7	101	11022	6.5	Hemoglobin beta chain (Fragment)
3753	B3VL05	3	79	30.5	105	11488	6.7	Beta globin (Fragment)
3753	Q8IZI0	3	79	30.5	105	11505	5.7	Hemoglobin beta chain variant Hb-I_Toulouse (Fragment)
3753	Q8IUL9	3	79	30.5	105	11548	6.1	Hemoglobin beta chain variant Hb.Sinai-Bel Air (Fragment)
3753	Q6V0K9	3	79	30.5	105	11474	6.7	Mutant hemoglobin beta chain (Fragment)
3753	Q6J1Z7	3	79	30.5	105	11446	6.7	Hemoglobin beta (Fragment)
3753	Q670S4	3	79	30.5	105	11459	6.7	Hemoglobin Lepore-Baltimore (Fragment)
3753	Q52MT0	3	79	30.5	105	11476	6.4	Beta globin (Fragment)
3753	Q3LR79	3	79	30.5	105	11482	5.8	Hemoglobin beta (Fragment)
3753	E9NGZ5	3	79	30.5	105	11494	6.4	Hemoglobin beta globin chain (Fragment)
3753	Q9BWV6	3	79	28.8	111	12234	6.1	Mutant beta globin
3753	Q9UK54	3	79	25	128	13964	7	Hemoglobin beta subunit variant (Fragment)
3753	B3VL86	3	79	23	139	14956	6.8	Mutant beta-globin
3753	P68871	3	79	21.8	147	15998	7.3	Hemoglobin subunit beta
3753	C8C504	3	79	21.8	147	15997	8.1	Beta-globin
3753	Q14473	3	79	18.3	175	18931	6.8	Beta-globin gene from a thalassemia patient,
3754	Q9BTL3	3	7	30.5	118	14381	8.9	Protein FAM103A1
3755	C0LAA5	3	3	29.8	181	21253	5.7	MHC class I antigen (Fragment)
3756	P52657	3	6	29.4	109	12457	6.8	Transcription initiation factor IIA subunit 2
3757	Q92934	3	5	29.2	168	18392	7.1	Bcl2 antagonist of cell death
3758	B4DEY0	3	8	28.6	192	21809	5.1	cDNA FLJ60450, highly similar to OTU domain-containing protein 6B
3758	Q8N6M0	3	8	18.8	293	33813	6	OTU domain-containing protein 6B
3758	UPI0000E5AF0F	3	8	17	323	37326	6.7	OTU domain-containing protein 6B
3759	UPI000020D624	3	4	34.2	117	12366	8.5	acyl-coenzyme A thioesterase 13 isoform 2
3759	Q9NPJ3	3	4	28.6	140	14960	9.1	Acyl-coenzyme A thioesterase 13
3760	UPI00006C1B87	3	5	28.3	127	14961	10.3	PREDICTED: protein LLP log
3760	UPI00006C1B33	3	5	28.3	127	14986	10.4	PREDICTED: protein LLP log
3760	Q9BRT6	3	5	27.9	129	15225	10.4	Protein LLP log
3761	A6ZKI3	3	4	27.4	113	13171	5.1	Protein FAM127A
3761	Q9BWD3	3	4	27.4	113	13188	5.1	Protein FAM127B
3762	Q13158	3	12	27.4	208	23279	5.7	Protein FADD
3763	B4E2R3	3	3	27.3	154	16760	5.4	cDNA FLJ58875, highly similar to sapiens G protein beta subunit-like (GBL), mRNA
3763	Q9BVC4-4	3	3	19.4	217	23850	6.2	Isoform 3 of Target of rapamycin complex subunit LST8
3763	Q9BVC4	3	3	12.9	326	35876	5.9	Target of rapamycin complex subunit LST8
3764	Q8N1A6	3	3	26.6	199	23468	5.4	UPF0462 protein C4orf33
3765	B4E3K9	3	7	26.5	162	18262	8.4	Superoxide dismutase
3765	Q8HXP7	3	7	21.7	198	22204	7.3	Superoxide dismutase [Mn], mitochondrial
3765	Q7Z7M4	3	7	20.2	213	23673	7.3	Superoxide dismutase (Fragment)
3765	P04179	3	7	19.4	222	24722	8.3	Superoxide dismutase [Mn], mitochondrial
3766	P69905	3	136	25.4	142	15258	8.7	Hemoglobin subunit alpha
3767	B4DQE7	3	4	24.2	182	20141	6.9	cDNA FLJ58211, highly similar to Histone deacetylase 8
3767	Q9BY41	3	4	11.7	377	41758	5.6	Histone deacetylase 8

Table S1. Proteins identified in the OVCAR-3 tumor supernatant by two or more unique peptides.

Protein or Protein Group	Accession Number	Unique Peptide Count	Spectrum Count	Sequence Coverage (%)	Length	MolWt	pI	Descriptive Name
3768	Q14353	3	6	24.2	236	26318	6.1	Guanidinoacetate N-methyltransferase
3769	Q969H6	3	6	23.9	163	18820	7.2	Ribonuclease P/MRP protein subunit POP5
3770	Q6ZSJ8	3	11	23.6	110	11471	6.7	Uncharacterized protein C1orf122
3771	Q96EY4	3	5	23.2	203	23864	9.3	UPF0534 protein C4orf43
3771	UPI0001D3B969	3	5	19.4	242	27685	9.6	UPI0001D3B969 UniRef100 entry
3772	Q96E14	3	5	23.1	147	15865	7.9	RecQ-mediated genome instability protein 2
3773	Q9NWQ9	3	3	22.9	140	16009	4.9	Uncharacterized protein C14orf119
3774	Q15041	3	10	22.7	203	23363	9.3	ADP-ribosylation factor-like protein 6-interacting protein 1
3775	Q96CP2	3	3	22.1	140	14564	8.5	FLYWCH family member 2
3776	A6NNI4	3	9	22	159	17764	6.3	Uncharacterized protein
3776	P21926	3	9	15.4	228	25416	7.1	CD9 antigen
3777	Q9H0F7	3	3	22	186	21097	8.5	ADP-ribosylation factor-like protein 6
3778	Q9HD47	3	3	22	186	20448	4.9	Ran guanine nucleotide release factor
3779	B3KTR4	3	3	21.9	201	22584	7.6	cDNA FLJ38643 fis, clone HHDPC2005513, highly similar to ARF-related protein 1
3779	Q13795	3	3	21.9	201	22614	7.6	ADP-ribosylation factor-related protein 1
3780	B1AHF3	3	4	21.8	147	16696	9.7	Cytochrome b5 reductase 3 (Fragment)
3780	P00387	3	4	10.6	301	34235	7.6	NADH-cytochrome b5 reductase 3
3780	B7Z7L3	3	4	9.6	334	38226	6.9	cDNA FLJ56301, highly similar to NADH-cytochrome b5 reductase (EC 1.6.2.2)
3781	O00161-2	3	4	29.1	158	17789	4.7	Isoform SNAP-23b of Synaptosomal-associated protein 23
3781	O00161	3	4	21.8	211	23354	5	Synaptosomal-associated protein 23
3782	Q5TAL3	3	13	21.7	180	19687	9.6	U1 small nuclear ribonucleoprotein C
3783	Q8TBF2-4	3	4	26.5	162	17621	6.2	Isoform 4 of Prostamide/prostaglandin F synthase
3783	Q8TBF2-2	3	4	23.5	183	19465	8.5	Isoform 2 of Prostamide/prostaglandin F synthase
3783	Q8TBF2-5	3	4	22.5	191	20548	5.9	Isoform 5 of Prostamide/prostaglandin F synthase
3783	Q8TBF2	3	4	21.7	198	21223	6.7	Prostamide/prostaglandin F synthase
3783	Q8TBF2-7	3	4	21.4	201	21597	8.6	Isoform 7 of Prostamide/prostaglandin F synthase
3783	Q8TBF2-3	3	4	19.9	216	23355	7	Isoform 3 of Prostamide/prostaglandin F synthase
3783	Q8TBF2-6	3	4	19.2	224	24122	7.2	Isoform 6 of Prostamide/prostaglandin F synthase
3783	UPI0001E306A4	3	4	18.9	228	24278	6.7	hypothetical protein LOC127281 isoform b
3784	Q9NRW3	3	5	21.6	190	22826	7.6	Probable DNA dC- dU-editing enzyme APOBEC-3C
3785	Q53HV7	3	4	21.5	270	29862	6.6	Single-strand selective monofunctional uracil DNA glycosylase
3786	C6GKU9	3	3	21	143	16279	9.2	Putative mediator of RNA polymerase II transcription subunit 30 short
3786	Q96HR3	3	3	16.9	178	20277	8.3	Mediator of RNA polymerase II transcription subunit 30
3787	Q5T0D9	3	5	20.6	272	30212	7.3	Tumor protein p63-regulated gene 1-like protein
3788	P51948	3	5	20.4	309	35823	6.1	CDK-activating kinase assembly factor MAT1
3789	Q9BW30	3	4	19.9	176	18985	9.1	Tubulin polymerization-promoting protein family member 3
3790	O60927	3	5	19.8	126	13952	7	Protein phosphatase 1 regulatory subunit 11
3791	Q6LBL5	3	3	20.1	189	20363	5.3	GM2 activator protein
3791	B2R699	3	3	19.7	193	20889	5.6	cDNA, FLJ92855, highly similar to sapiens GM2 ganglioside activator protein (GM2A), mRNA
3791	P17900	3	3	19.7	193	20838	5.3	Ganglioside GM2 activator
3792	B2RB06	3	5	19.7	314	34261	8.9	cDNA, FLJ95242, highly similar to sapiens L-3-hydroxyacyl-Coenzyme A dehydrogenase, short chain (HADHSC), mRNA
3792	Q16836	3	5	19.7	314	34294	8.9	Hydroxyacyl-coenzyme A dehydrogenase, mitochondrial
3792	E9PF18	3	5	19.5	318	35237	7.2	Uncharacterized protein
3792	B3KTT6	3	5	19.5	318	35221	7.2	cDNA FLJ38699 fis, clone KIDNE2002168, highly similar to Short chain 3-hydroxyacyl-CoA dehydrogenase, mitochondrial (EC 1.1.1.35)
3792	UPI0001D34148	3	5	18.7	331	36035	8.5	hydroxyacyl-coenzyme A dehydrogenase, mitochondrial isoform 1 precursor
3792	UPI000013D5C7	3	5	15.9	390	42123	9.3	Hydroxyacyl-coenzyme A dehydrogenase, mitochondrial precursor (EC 1.1.1.35) (Short chain 3-hydroxyacyl-CoA dehydrogenase) (HCDH) (Medium and short chain L-3-hydroxyacyl-coenzyme A dehydrogenase).
3792	Q16836-2	3	5	15.9	390	42140	9.3	Isoform 2 of Hydroxyacyl-coenzyme A dehydrogenase, mitochondrial
3793	P16035	3	9	19.5	220	24399	7.5	Metalloproteinase inhibitor 2
3794	O00505	3	5	19.4	521	57811	4.9	Importin subunit alpha-3
3794	Q8IYQ9	3	5	19.4	521	57901	4.9	Karyopherin alpha 3 (Importin alpha 4)
3795	Q2TAM9	3	3	19.3	212	23390	11.2	Tumor suppressor candidate gene 1 protein
3796	O15551	3	14	19.1	220	23319	8.1	Claudin-3
3797	UPI0001AE691F	3	7	18.9	355	39542	7.4	UPI0001AE691F UniRef100 entry
3797	Q8TDR6	3	7	18.8	356	39531	7	Cytoplasmic protein Ndr1
3797	Q9UN36-2	3	7	18.8	357	39289	5.4	Isoform 2 of Protein NDRG2
3797	Q9UN36-3	3	7	18.6	360	39679	5.4	Isoform 3 of Protein NDRG2
3797	Q9UN36	3	7	18.1	371	40798	5.2	Protein NDRG2
3798	A6NG64	3	3	18.6	226	25689	6.5	Uncharacterized protein
3799	Q8IWT0	3	5	18.6	167	19491	4.5	Protein archease
3800	Q96HR9	3	6	18.5	184	20733	8.6	Receptor expression-enhancing protein 6
3801	P17028-2	3	3	34.7	193	22382	4.6	Isoform 2 of Zinc finger protein 24
3801	P17028	3	3	18.2	368	42155	6.2	Zinc finger protein 24
3802	Q96HA8	3	5	18	205	23680	5.8	Protein N-terminal glutamine amidohydrolase
3803	B3KUU1	3	8	22.3	265	29467	5	Target of myb1 (Chicken), isoform CRA_a
3803	B0QY01	3	8	17.6	335	37023	4.8	Target of myb1 (Chicken) (Fragment)
3803	E7EPD0	3	8	13	454	49597	5	Uncharacterized protein
3803	B4DJ44	3	8	13	454	49578	5.1	cDNA FLJ54716, highly similar to Target of Myb protein 1
3803	O60784-2	3	8	12	493	53875	4.7	Isoform 2 of Target of Myb protein 1
3803	O60784	3	8	12	492	53818	4.7	Target of Myb protein 1
3803	E7EPZ3	3	8	11.8	501	54852	4.8	Uncharacterized protein
3803	B4DKQ5	3	8	11.8	501	54832	4.8	cDNA FLJ54710, highly similar to Target of Myb protein 1
3804	Q92871	3	3	17.6	262	29747	5.7	Phosphomannomutase 1
3805	Q5TIH2	3	5	25.9	108	11756	8.1	SFT2 domain containing 2
3805	O95562	3	5	17.5	160	17779	9.1	Vesicle transport protein SFT2B
3806	P50225	3	4	17.3	295	34165	6.6	Sulfotransferase 1A1
3807	UPI000002B290	3	3	33.3	123	13604	9.5	RNA-binding protein 38 (RNA-binding motif protein 38) (RNA-binding region-containing protein 1) (HSRNASEB) (ssDNA-binding protein SEB4) (CLL-associated antigen KW-5).
3807	Q9H0Z9	3	3	17.2	239	25498	8.6	RNA-binding protein 38
3808	A1X4Q1	3	3	17	230	26056	5.3	PMCA4b (Fragment)
3808	P23634-7	3	3	3.3	1193	132594	6.2	Isoform ZB of Plasma membrane calcium-transporting ATPase 4
3808	Q68DH9	3	3	3.2	1205	133874	6.5	Putative uncharacterized protein DKFZp686M088
3808	P23634-8	3	3	3.2	1229	136583	6.4	Isoform ZD of Plasma membrane calcium-transporting ATPase 4
3808	P23634-6	3	3	3.2	1205	133930	6.4	Isoform XB of Plasma membrane calcium-transporting ATPase 4
3808	P23634	3	3	3.1	1241	137920	6.6	Plasma membrane calcium-transporting ATPase 4
3809	Q6N063	3	4	16.9	350	38996	5.7	2-oxoglutarate and iron-dependent oxygenase domain-containing protein 2

Table S1. Proteins identified in the OVCAR-3 tumor supernatant by two or more unique peptides.

Protein or Protein Group	Accession Number	Unique Peptide Count	Spectrum Count	Sequence Coverage (%)	Length	MolWt	pI	Descriptive Name
3810	UPI00018814B8	3	4	33.9	121	14085	5.9	Thiamin pyrophosphokinase 1 (EC 2.7.6.2) (Thiamine pyrophosphokinase 1) (hTPK1) (Placental protein 20) (PP20).
3810	UPI00001AEE82	3	4	21.1	194	21886	5.4	thiamin pyrophosphokinase 1 isoform b
3810	Q9H3S4	3	4	16.9	243	27265	5.2	Thiamin pyrophosphokinase 1
3811	Q9P2W1-2	3	3	17.6	205	23636	8.2	Isoform 2 of logous-pairing protein 2 log
3811	Q9P2W1	3	3	16.6	217	24906	7.8	logous-pairing protein 2 log
3812	O95707	3	6	16.4	220	25425	10.1	Ribonuclease P protein subunit p29
3813	Q92520	3	8	16.3	227	24680	8.3	Protein FAM3C
3814	O95197-3	3	10	16.1	236	25609	8.5	Isoform 3 of Reticulon-3
3815	Q96R05	3	4	21.6	134	15536	7.8	Retinoid-binding protein 7
3815	B1AQ60	3	4	16	181	20874	8.2	Retinol binding protein 7, cellular
3815	Q8N4L3	3	4	12.9	225	25846	9.7	RBP7 protein (Fragment)
3816	Q9NRG1	3	4	16	225	25673	6.1	Phosphoribosyltransferase domain-containing protein 1
3817	Q3SY19	3	5	15.8	247	26556	4.8	PRSS1 protein
3818	Q9UHW5	3	3	15.8	284	32761	4.5	GPN-loop GTPase 3
3818	Q9UHW5-2	3	3	15.3	294	33947	4.6	Isoform 2 of GPN-loop GTPase 3
3818	UPI0001B25F61	3	3	13.9	323	37438	4.7	GPN-loop GTPase 3 isoform 2
3819	A9UFC0	3	3	15.7	242	27666	5.3	Caspase 14
3819	P31944	3	3	15.7	242	27680	5.6	Caspase-14
3820	Q9BYG3	3	5	15.7	293	34222	9.9	MKI67 FHA domain-interacting nucleolar phosphoprotein
3821	A8K670	3	4	15.6	301	33184	8.8	cDNA FLJ75703, highly similar to sapiens nitric oxide synthase interacting protein (NOSIP), mRNA
3821	Q9Y314	3	4	15.6	301	33172	8.8	Nitric oxide synthase-interacting protein
3822	B3KW72	3	3	15.5	181	20955	9.7	cDNA FLJ42396 fis, clone ASTRO2001107
3822	C9J384	3	3	12.4	225	26228	9.9	Uncharacterized protein
3822	E9PHS3	3	3	10.7	261	29964	9.5	Uncharacterized protein
3822	Q9BQ75	3	3	10	279	31884	9.2	Uncharacterized protein C3orf26
3823	Q9BQ24	3	3	15.4	234	26506	8.4	Zinc finger FYVE domain-containing protein 21
3823	Q9BQ24-2	3	3	14.3	252	28477	8.3	Isoform 2 of Zinc finger FYVE domain-containing protein 21
3824	UPI00003B0EC0	3	6	17.6	306	32800	5.4	NF-kappa-B inhibitor beta isoform b
3824	Q96LS1	3	6	17.6	306	32803	5.4	cDNA FLJ25133 fis, clone CBR06869, highly similar to THYROID RECEPTOR INTERACTING PROTEIN 9
3824	Q15653	3	6	15.2	356	37771	4.8	NF-kappa-B inhibitor beta
3824	Q59EM7	3	6	15	361	37778	4.9	NF-kappaB inhibitor beta variant (Fragment)
3825	B4DUB7	3	3	15.1	813	93008	7.2	Phosphorylase
3825	UPI0001B1A4B7	3	3	15.1	813	93134	7.3	glycogen phosphorylase, liver form isoform 2
3825	E9PK47	3	3	15	819	94070	7.3	Uncharacterized protein
3825	P06737	3	3	14.5	847	97149	7.2	Glycogen phosphorylase, liver form
3826	Q53GL1	3	5	15.1	279	31920	8.9	Nicotinamide nucleotide adenylyltransferase 1 variant (Fragment)
3826	Q9HAN9	3	5	15.1	279	31932	8.9	Nicotinamide mononucleotide adenylyltransferase 1
3827	E9PDW1	3	3	14.9	248	28046	5.3	Uncharacterized protein
3827	Q9NPJ6	3	3	13.7	270	29745	5.1	Mediator of RNA polymerase II transcription subunit 4
3828	E9PMJ2	3	5	14.7	347	39137	6	Uncharacterized protein
3828	Q9BPY3	3	5	14.5	351	39499	6	Protein FAM118B
3829	Q96DX7	3	5	14.5	344	38472	4.2	Tripartite motif-containing protein 44
3830	A8MX32	3	4	14.3	300	34305	8.5	Uncharacterized protein
3830	C9JU95	3	4	14.1	305	34756	8.4	Uncharacterized protein
3830	B4DKE6	3	4	14.1	305	34833	8.6	cDNA FLJ60629, highly similar to Replication factor C subunit 3
3830	Q5U0C2	3	4	12.1	356	40570	8.3	Replication factor C (Activator 1) 3, 38kDa
3830	P40938	3	4	12.1	356	40556	8.3	Replication factor C subunit 3
3831	Q5R372-8	3	3	14.1	370	42689	7.3	Isoform 8 of Rab GTPase-activating protein 1-like
3832	A8K644	3	22	14	494	56708	11.5	Splicing factor, arginine/serine-rich 4, isoform CRA_b
3832	Q08170	3	22	14	494	56678	11.5	Serine/arginine-rich splicing factor 4
3833	Q96CS2-2	3	3	19.3	202	22744	5.8	Isoform 2 of HAUS augmin-like complex subunit 1
3833	Q96CS2	3	3	14	278	31863	5.5	HAUS augmin-like complex subunit 1
3834	Q99836-2	3	4	14.7	251	28280	8.3	Isoform 2 of Myeloid differentiation primary response protein MyD88
3834	B4DKU4	3	4	13.5	275	31496	6.1	cDNA FLJ54834, highly similar to Myeloid differentiation primary response protein MyD88
3834	Q99836	3	4	12.5	296	33233	6.1	Myeloid differentiation primary response protein MyD88
3834	UPI0000E5A580	3	4	11.7	317	35444	7.5	myeloid differentiation primary response protein MyD88 isoform 1
3835	B2R7D2	3	6	13.1	487	55120	8	cDNA, FLJ93389, highly similar to sapiens multiple inositol polyphosphate histidine phosphatase, 1 (MINPP1), mRNA
3835	Q9UNW1	3	6	13.1	487	55051	7.8	Multiple inositol polyphosphate phosphatase 1
3836	B4DZ90	3	4	13.1	251	27658	6.8	cDNA FLJ56312, highly similar to sapiens TruB pseudouridine (psi) synthase log 1 (TRUB1), mRNA
3836	Q8WWH5	3	4	9.5	349	37253	8.3	Probable tRNA pseudouridine synthase 1
3837	Q8NEZ2-2	3	5	14	372	41456	5.8	Isoform 2 of Vacuolar protein sorting-associated protein 37A
3837	Q8NEZ2	3	5	13.1	397	44314	5.6	Vacuolar protein sorting-associated protein 37A
3838	Q9Y3V2	3	3	13.1	267	30543	6.2	RWD domain-containing protein 3
3838	UPI0001596894	3	3	13.1	267	30515	6.2	RWD domain-containing protein 3 isoform a
3839	UPI0001AE6707	3	4	14.4	299	33806	8.9	tRNA-dihydrouridine synthase 1-like (EC 1.-.-.-).
3839	Q9BTJ3	3	4	14.4	299	33796	8.9	DUS1L protein (Fragment)
3839	B4DPG7	3	4	12.8	336	37379	8.8	cDNA FLJ51922, highly similar to tRNA-dihydrouridine synthase 1-like (EC 1.-.-.-)
3839	Q6P1R4	3	4	9.1	473	53230	8.3	tRNA-dihydrouridine synthase 1-like
3840	B5ME06	3	7	12.8	266	30406	11.7	Uncharacterized protein
3841	D3DU85	3	5	12.6	326	36153	8.2	Solute carrier family 9 (Sodium/hydrogen exchanger), member 3 regulator 2, isoform CRA_b
3841	Q15599-2	3	5	12.6	326	36152	8.5	Isoform 2 of Na(+)/H(+) exchange regulatory cofactor NHE-RF2
3841	Q15599	3	5	12.2	337	37414	7.9	Na(+)/H(+) exchange regulatory cofactor NHE-RF2
3842	Q7LBR1	3	11	12.6	199	22109	8.1	Charged multivesicular body protein 1b
3843	B2RBI2	3	7	12.5	352	39905	4.5	cDNA, FLJ95525, highly similar to sapiens synapse associated protein 1, SAP47 log (Drosophila) (SYAP1), mRNA
3843	Q96A49	3	7	12.5	352	39933	4.5	Synapse-associated protein 1
3843	Q53GS5	3	7	12.5	352	39934	4.5	SYAP1 protein variant (Fragment)
3844	Q96LA8-2	3	4	16.1	292	32509	5	Isoform 2 of Protein arginine N-methyltransferase 6
3844	Q96LA8	3	4	12.5	375	41938	5.4	Protein arginine N-methyltransferase 6
3845	Q8N584	3	3	12.2	583	65870	7	Tetratricopeptide repeat protein 39C
3846	B7Z385	3	3	13.7	365	39911	5.6	Adenylyl cyclase-associated protein
3846	Q5JPJ8	3	3	12.7	394	43539	5.9	Adenylyl cyclase-associated protein
3846	E9PDI0	3	3	12.7	395	43594	5.7	Uncharacterized protein
3846	B7Z1C4	3	3	12.1	413	45245	5.7	Adenylyl cyclase-associated protein
3846	P40123	3	3	10.5	477	52824	6.4	Adenylyl cyclase-associated protein 2
3847	O75191	3	3	11.8	536	58382	5.9	Xylulose kinase
3848	P0CB43	3	5	11.8	390	42128	4.8	Protein FAM203B
3848	Q9BTY7	3	5	11.8	390	42129	4.8	Protein FAM203A
3849	B3KN49	3	4	11.7	359	40607	4.9	cDNA FLJ13562 fis, clone PLACE1008080, highly similar to sapiens hexamethylene bis-acetamide inducible 1 (HEXIM1), mRNA
3849	O94992	3	4	11.7	359	40623	4.9	Protein HEXIM1

Table S1. Proteins identified in the OVCAR-3 tumor supernatant by two or more unique peptides.

Protein or Protein Group	Accession Number	Unique Peptide Count	Spectrum Count	Sequence Coverage (%)	Length	MolWt	pI	Descriptive Name
3850	P57081-3	3	3	18	266	29946	7.4	Isoform 3 of tRNA (guanine-N(7)-)-methyltransferase subunit WDR4
3850	P57081	3	3	11.7	412	45490	7.1	tRNA (guanine-N(7)-)-methyltransferase subunit WDR4
3850	P57081-2	3	3	11.7	411	45362	6.9	Isoform 2 of tRNA (guanine-N(7)-)-methyltransferase subunit WDR4
3851	Q9H6Y2-2	3	3	27.3	165	18180	5.4	Isoform 2 of WD repeat-containing protein 55
3851	Q9H6Y2	3	3	11.7	383	42070	4.9	WD repeat-containing protein 55
3852	Q9Y3B9	3	5	11.7	282	31484	5.5	RRP15-like protein
3853	Q9H1Y0-2	3	3	16.2	197	22931	6.2	Isoform Short of Autophagy protein 5
3853	B7Z5Y6	3	3	16.2	197	22989	6	cDNA FLJ50845, highly similar to Autophagy protein 5
3853	B3KMH8	3	3	11.6	275	32375	5.9	cDNA FLJ11037 fis, clone PLACE1004316, highly similar to Autophagy protein 5
3853	Q9H1Y0	3	3	11.6	275	32447	5.8	Autophagy protein 5
3854	Q03426	3	4	11.6	396	42451	6.5	Mevalonate kinase
3854	Q59ET9	3	4	10.9	421	44771	6.6	Mevalonate kinase variant (Fragment)
3855	Q9H467	3	3	11.5	287	32009	4.8	CUE domain-containing protein 2
3856	P57060	3	4	11.3	319	36332	5.9	RWD domain-containing protein 2B
3856	Q53FD2	3	4	11.3	319	36360	5.9	Chromosome 21 open reading frame 6 variant (Fragment)
3857	Q9BRP1	3	6	11.2	358	39417	4.9	Programmed cell death protein 2-like
3858	B4DTK9	3	3	13.3	443	48358	7.2	cDNA FLJ58282, highly similar to Probable transcription factor PML
3858	A4VCI9	3	3	10.9	540	59571	6.1	PML protein (Fragment)
3858	P29590-6	3	3	7.2	819	90986	6.6	Isoform PML-9 of Protein PML
3858	P29590-11	3	3	7.1	834	92564	6.3	Isoform PML-11 of Protein PML
3858	P29590-7	3	3	6.8	865	95677	6.2	Isoform PML-10 of Protein PML
3858	P29590	3	3	6.7	882	97551	6.2	Protein PML
3859	Q6P6C2-2	3	7	12.7	394	44256	9.1	Isoform 2 of Probable alpha-ketoglutarate-dependent dioxygenase ABH5
3859	Q6P6C2	3	7	10.9	458	51409	8.4	Probable alpha-ketoglutarate-dependent dioxygenase ABH5
3860	A8K7R5	3	4	10.6	416	46367	6	cDNA FLJ78720, highly similar to sapiens caspase 9, apoptosis-related cysteine peptidase (CASP9), transcript variant alpha, mRNA
3860	A8K7U6	3	4	10.6	416	46337	6.2	cDNA FLJ75893, highly similar to sapiens caspase 9, apoptosis-related cysteine peptidase (CASP9), transcript variant alpha, mRNA
3861	B2RAA8	3	3	10.6	282	31137	5.1	cDNA, FLJ94802
3861	Q9BR61	3	3	10.6	282	31151	5.1	Acyl-CoA-binding domain-containing protein 6
3862	Q9BQC3	3	4	10.6	489	52083	5.5	Diphthamide biosynthesis protein 2
3863	Q9NRF8	3	5	10.6	586	65678	6.9	CTP synthase 2
3864	B4DKL4	3	4	12.4	493	54498	8.9	cDNA FLJ55699, highly similar to sapiens liver-specific bHLH-Zip transcription factor (LISCH7), transcript variant 3, mRNA
3864	A6NDW3	3	4	10.5	581	64053	8.8	Uncharacterized protein
3864	Q9BWS2	3	4	10.5	582	64347	7.3	LSR protein (Fragment)
3864	Q9BT33	3	4	10.4	588	64894	7.3	LSR protein (Fragment)
3864	Q86X29-2	3	4	10.3	591	65587	8	Isoform 2 of Lipolysis-stimulated lipoprotein receptor
3864	Q86X29-3	3	4	9.7	629	69429	8.1	Isoform 3 of Lipolysis-stimulated lipoprotein receptor
3864	E9PHD4	3	4	9.7	630	69558	8.1	Uncharacterized protein
3864	Q86X29	3	4	9.4	649	71439	8	Lipolysis-stimulated lipoprotein receptor
3865	Q86YA9	3	3	12.3	552	61379	6	Golgi associated, gamma adaptin ear containing, ARF binding protein 1
3865	Q9UJY5-2	3	3	11.2	606	66563	5.1	Isoform 2 of ADP-ribosylation factor-binding protein GGA1
3865	UPI0001C53D2C	3	3	10.7	635	69815	5.2	ADP-ribosylation factor-binding protein GGA1 isoform 4
3865	Q9UJY5	3	3	10.6	639	70384	5.3	ADP-ribosylation factor-binding protein GGA1
3865	Q6IC75	3	3	10.4	656	72277	5.4	GGA1 protein
3866	UPI000022ACCC	3	6	12.4	362	40867	7	tubulointerstitial nephritis antigen-like isoform 3
3866	B4DPK6	3	6	10.3	436	48808	7.3	cDNA FLJ55020, highly similar to Tubulointerstitial nephritis antigen-like
3866	Q9GZM7	3	6	9.6	467	52387	7	Tubulointerstitial nephritis antigen-like
3867	O95400	3	4	10.3	341	37646	4.6	CD2 antigen cytoplasmic tail-binding protein 2
3868	O96013	3	87	10.2	591	64072	9.7	Serine/threonine-protein kinase PAK 4
3869	B2R8K8	3	3	9.9	302	34537	8.3	cDNA, FLJ93949, highly similar to sapiens NIMA (never in mitosis gene a)-related kinase 7 (NEK7), mRNA
3869	Q8TDX7	3	3	9.9	302	34551	8.3	Serine/threonine-protein kinase Nek7
3870	UPI00001404C1	3	3	12.6	341	38073	7.7	UPI00001404C1 UniRef100 entry
3870	B3KM88	3	3	9.8	441	49341	6.3	cDNA FLJ10527 fis, clone NT2RP2000932, highly similar to Ankyrin repeat and MYND domain-containing protein 2
3870	Q8IV38	3	3	9.8	441	49299	6.3	Ankyrin repeat and MYND domain-containing protein 2
3870	B3KN56	3	3	9.8	441	49352	6.4	cDNA FLJ13642 fis, clone PLACE1011263, highly similar to Ankyrin repeat and MYND domain-containing protein 2
3871	Q86Y37-2	3	3	11.9	303	33584	5.1	Isoform 2 of Uncharacterized protein C10orf46
3871	Q86Y37	3	3	9.8	369	41064	5.2	Uncharacterized protein C10orf46
3872	B4DPW1	3	11	9.7	462	52599	6.5	cDNA FLJ59373, highly similar to sapiens pericentrin 1 (PCNT1), mRNA
3872	Q9BW27	3	11	6.9	656	75019	5.6	Nuclear pore complex protein Nup85
3873	A8K3W7	3	3	9.6	429	49960	6.6	cDNA FLJ76271, highly similar to sapiens protein O-fucosyltransferase 2 (POFUT2), transcript variant 3, mRNA
3873	Q9Y2G5	3	3	9.6	429	49976	6.6	GDP-fucose protein O-fucosyltransferase 2
3874	Q9Y6W5	3	6	9.6	498	54284	5.5	Wiskott-Aldrich syndrome protein family member 2
3875	B7Z7E2	3	3	11.3	592	62921	5.9	cDNA FLJ58548, highly similar to ADP-ribosylation factor-binding protein GGA3
3875	B7Z456	3	3	11.1	601	64208	5.5	cDNA FLJ59569, highly similar to ADP-ribosylation factor-binding protein GGA3
3875	B7Z7M9	3	3	10.3	651	70088	6.2	cDNA FLJ58553, highly similar to ADP-ribosylation factor-binding protein GGA3
3875	Q9NZ52-2	3	3	9.7	690	74470	5.3	Isoform Short of ADP-ribosylation factor-binding protein GGA3
3875	A8K6M0	3	3	9.7	690	74469	5.3	cDNA FLJ75879, highly similar to sapiens GGA3 short isoform
3875	A8K6I6	3	3	9.3	723	78256	5.7	cDNA FLJ75092, highly similar to sapiens golgi associated, gamma adaptin ear containing, ARF binding protein 3 (GGA3), transcript variant long, mRNA
3875	Q9NZ52	3	3	9.3	723	78315	5.6	ADP-ribosylation factor-binding protein GGA3
3876	B7Z4K6	3	3	10.8	305	33615	8.3	cDNA FLJ51996, highly similar to Deoxyribonuclease-2-alpha (EC 3.1.22.1)
3876	C0LQF2	3	3	9.6	344	38181	8.3	Deoxyribonuclease II (Fragment)
3876	B3KQS9	3	3	9.2	360	39566	8	cDNA PSEC0141 fis, clone PLACE1005913, highly similar to Deoxyribonuclease-2-alpha (EC 3.1.22.1)
3876	O00115	3	3	9.2	360	39581	8	Deoxyribonuclease-2-alpha
3877	O00425	3	7	9.2	579	63705	8.9	Insulin-like growth factor 2 mRNA-binding protein 3
3878	B4DP28	3	3	9	435	47172	5.2	cDNA FLJ51260, highly similar to DNA polymerase subunit delta 2 (EC 2.7.7.7)
3878	C9JEQ6	3	3	8.6	455	49613	5.6	Uncharacterized protein
3878	P49005	3	3	8.3	469	51289	5.6	DNA polymerase delta subunit 2
3879	Q9HAT2-2	3	3	9.6	488	54572	7.3	Isoform 2 of Sialate O-acetylesterase
3879	Q9HAT2	3	3	9	523	58315	7.3	Sialate O-acetylesterase

Table S1. Proteins identified in the OVCAR-3 tumor supernatant by two or more unique peptides.

Protein or Protein Group	Accession Number	Unique Peptide Count	Spectrum Count	Sequence Coverage (%)	Length	MolWt	pI	Descriptive Name
3880	B3KS50	3	6	8.9	447	51759	8.5	cDNA FLJ35494 fis, clone SMINT2008720, highly similar to Interferon-induced protein with tetratricopeptide repeats 1
3880	P09914	3	6	8.4	478	55360	7.2	Interferon-induced protein with tetratricopeptide repeats 1
3881	Q13613-2	3	7	10.2	568	63330	7.5	Isoform 1A of Myotubularin-related protein 1
3881	Q13613	3	7	8.7	665	74678	7.1	Myotubularin-related protein 1
3881	UPI000004FD4F	3	7	8.6	673	75718	7.4	UPI000004FD4F UniRef100 entry
3882	Q8IWE2	3	3	8.7	563	60742	4.7	Protein NOXP20
3883	Q96AB6	3	3	8.7	310	34677	6.3	Protein N-terminal asparagine amidohydrolase
3884	Q96L92-3	3	3	8.9	528	59811	6.3	Isoform 2 of Sorting nexin-27
3884	Q96L92	3	3	8.7	541	61265	6.5	Sorting nexin-27
3885	B4DYC0	3	6	8.6	757	84392	5.2	cDNA FLJ57012, highly similar to AT-rich interactive domain-containing protein 1B
3885	E9PD33	3	6	8.6	757	84408	5.2	Uncharacterized protein
3885	UPI0001D3BCFD	3	6	3.7	1758	190696	6.3	UPI0001D3BCFD UniRef100 entry
3885	Q8NFD5-2	3	6	2.9	2249	237659	6.7	Isoform 2 of AT-rich interactive domain-containing protein 1B
3885	Q8NFD5	3	6	2.9	2236	236121	6.7	AT-rich interactive domain-containing protein 1B
3885	Q8NFD5-3	3	6	2.8	2289	241461	6.7	Isoform 3 of AT-rich interactive domain-containing protein 1B
3886	B4E0X9	3	3	11.3	390	43422	5.7	cDNA FLJ54223, highly similar to Transmembrane protein 1
3886	B4DI17	3	3	8.5	518	57526	5.9	cDNA FLJ55683, highly similar to Transmembrane protein 1
3886	P48553	3	3	3.5	1259	142189	6	Trafficking protein particle complex subunit 10
3887	Q7Z434-2	3	4	33.3	138	15996	7	Isoform 2 of Mitochondrial antiviral-signaling protein
3887	Q7Z434	3	4	8.5	540	56528	5.5	Mitochondrial antiviral-signaling protein
3888	Q8N543	3	9	8.5	542	63246	5.1	2-oxoglutarate and iron-dependent oxygenase domain-containing protein 1
3889	A8MWB4	3	3	8.3	398	46427	5.5	Uncharacterized protein
3889	Q15438-2	3	3	8.3	397	46356	5.5	Isoform 2 of Cytohesin-1
3889	Q15438	3	3	8.3	398	46413	5.5	Cytohesin-1
3890	B4DE20	3	3	8.3	568	65076	7.4	cDNA FLJ57256, highly similar to Plexin-A1
3890	Q9UIW2	3	3	2.5	1896	211066	6.9	Plexin-A1
3891	B4E1J2	3	8	8.2	461	50840	4.8	cDNA FLJ54229
3891	E7ENZ8	3	8	8.2	461	50824	4.8	Uncharacterized protein
3891	Q9NRY5	3	8	7.5	505	55468	4.9	Protein FAM114A2
3891	UPI0001AE74C1	3	8	7.4	512	56242	4.9	UPI0001AE74C1 UniRef100 entry
3892	Q969S3	3	3	8.2	477	54272	6.2	Zinc finger protein 622
3893	Q96I24	3	11	8.2	572	61640	8.4	Far upstream element-binding protein 3
3894	A6NG79	3	5	8.1	459	48960	5.5	Uncharacterized protein
3894	E9PGC6	3	5	7.9	467	50376	7.5	Uncharacterized protein
3894	Q96FT4	3	5	7.5	491	52422	5.1	Papillary renal cell carcinoma (Translocation-associated)
3894	Q92733	3	5	7.5	491	52418	5.1	Proline-rich protein PRCC
3895	Q59H78	3	4	8.1	583	61473	7.4	PREDICTED: filaggrin variant (Fragment)
3896	Q14738-3	3	9	9.7	496	58453	6.8	Isoform Delta-3 of Serine/threonine-protein phosphatase 2A 56 kDa regulatory subunit delta isoform
3896	Q59EF0	3	9	9.5	503	59336	7.4	Delta isoform of regulatory subunit B56, protein phosphatase 2A isoform 1 variant (Fragment)
3896	B4DSD7	3	9	8.6	559	65404	7.8	cDNA FLJ55863, highly similar to Serine/threonine-protein phosphatase 2A 56 kDa regulatory subunit delta isoform
3896	Q14738-2	3	9	8.4	570	66181	7.6	Isoform Delta-2 of Serine/threonine-protein phosphatase 2A 56 kDa regulatory subunit delta isoform
3896	E9PFR3	3	9	8.1	594	69117	8.1	Uncharacterized protein
3896	A8K6Y2	3	9	8	602	69979	7.8	cDNA FLJ76895, highly similar to sapiens protein phosphatase 2, regulatory subunit B (B56), delta isoform (PPP2R5D), transcript variant 1, mRNA
3896	Q14738	3	9	8	602	69992	8.1	Serine/threonine-protein phosphatase 2A 56 kDa regulatory subunit delta isoform
3897	B4E2S7	3	4	8	363	39812	5.7	cDNA FLJ58780, highly similar to sapiens lysosomal-associated membrane protein 2 (LAMP2), transcript variant LAMP2B, mRNA
3897	Q6Q3G8	3	4	7.1	411	45170	5.9	Lysosomal-associated membrane protein 2, isoform CRA_b
3897	P13473-2	3	4	7.1	410	44956	5.7	Isoform LAMP-2B of Lysosome-associated membrane glycoprotein 2
3897	P13473	3	4	7.1	410	44961	5.6	Lysosome-associated membrane glycoprotein 2
3898	Q6RW13	3	10	30.2	159	17419	6.1	Type-1 angiotensin II receptor-associated protein
3898	D7RF68	3	10	8	597	66206	8.9	AGTRAP-BRAF fusion protein
3899	B1AP16	3	3	11.9	293	32190	5.5	CD55 molecule, decay accelerating factor for complement (Cromer blood group) (Fragment)
3899	UPI0001F782BB	3	3	10.7	326	35809	5.5	Complement decay-accelerating factor precursor (CD55 antigen).
3899	E7ER69	3	3	9.8	358	38964	7.4	Uncharacterized protein
3899	Q14UF5	3	3	9.6	364	39759	7.6	Decay-accelerating factor splicing variant 2
3899	E9PSH2	3	3	9.3	377	41228	8	Uncharacterized protein
3899	P08174	3	3	9.2	381	41400	7.6	Complement decay-accelerating factor
3899	Q14UF4	3	3	9.1	384	41900	7.9	Decay-accelerating factor splicing variant 3
3899	Q14UF6	3	3	8	439	48513	7.9	Decay-accelerating factor splicing variant 1
3899	P08174-2	3	3	8	440	48717	8.4	Isoform 1 of Complement decay-accelerating factor
3899	B1AP13	3	3	7.9	444	49338	8.8	CD55 molecule, decay accelerating factor for complement (Cromer blood group)
3899	Q14UF3	3	3	6.7	525	56218	8.5	Decay-accelerating factor splicing variant 4
3899	Q14UF2	3	3	6.4	551	59038	8.8	Decay-accelerating factor splicing variant 5
3900	Q8N9N2-2	3	4	8.1	357	41228	5.5	Isoform 2 of Activating signal cointegrator 1 complex subunit 1
3900	B7Z518	3	4	7.7	379	43582	5.6	cDNA FLJ55237, highly similar to sapiens activating signal cointegrator 1 complex subunit 1 (ASCC1), mRNA
3901	Q9NXW2	3	5	7.7	375	41819	8.5	DnaJ log subfamily B member 12
3901	Q9NXW2-2	3	5	7.7	377	42263	8.5	Isoform 2 of DnaJ log subfamily B member 12
3901	UPI0000E592BF	3	5	7.1	409	45490	9.4	dnaJ log subfamily B member 12
3902	Q5JR89	3	8	19.2	287	32448	9.2	Kinesin family member 2C (Fragment)
3902	Q5JR91	3	8	16.4	336	37922	9.1	Kinesin family member 2C (Fragment)
3902	B7Z6Q6	3	8	8	684	76983	8.1	cDNA FLJ54088, highly similar to Kinesin-like protein KIF2C
3902	A8K3S3	3	8	7.6	725	81341	7.8	cDNA FLJ75664, highly similar to sapiens kinesin family member 2C (KIF2C), mRNA
3902	Q99661	3	8	7.6	725	81313	7.8	Kinesin-like protein KIF2C
3903	UPI000198CADE	3	3	11.4	621	69561	5.9	UPI000198CADE UniRef100 entry
3903	Q53H56	3	3	7.8	916	103975	5.4	Ubiquitin carboxyl-terminal hydrolase (Fragment)
3903	Q13107-2	3	3	7.8	916	103958	5.5	Isoform 2 of Ubiquitin carboxyl-terminal hydrolase 4
3903	Q13107	3	3	7.4	963	108565	5.7	Ubiquitin carboxyl-terminal hydrolase 4
3903	Q53FJ3	3	3	7.4	963	108591	5.7	Ubiquitin carboxyl-terminal hydrolase (Fragment)
3904	Q59EA5	3	4	11.4	298	34219	6.8	Tripartite motif-containing 38 variant (Fragment)
3904	B2R862	3	4	7.3	465	53400	7	cDNA, FLJ93754, highly similar to sapiens tripartite motif-containing 38 (TRIM38), mRNA
3904	O00635	3	4	7.3	465	53416	7	Tripartite motif-containing protein 38
3905	Q86TV4	3	9	7.2	545	60023	5.1	Full-length cDNA clone CS0DI085YI08 of Placenta of sapiens (human) (Fragment)
3906	Q86V21	3	4	7.1	672	75144	6.2	Acetoacetyl-CoA synthetase

Table S1. Proteins identified in the OVCAR-3 tumor supernatant by two or more unique peptides.

Protein or Protein Group	Accession Number	Unique Peptide Count	Spectrum Count	Sequence Coverage (%)	Length	MolWt	pI	Descriptive Name
3907	A8K045	3	3	7	489	55238	5.6	cDNA FLJ76988, highly similar to sapiens HSPB (heat shock 27kDa) associated protein 1 (HSPBAP1), mRNA
3907	Q96EW2	3	3	7	488	55167	5.6	HSPB1-associated protein 1
3908	A8K3R2	3	6	7	746	83570	6.1	cDNA FLJ78061, highly similar to sapiens block of proliferation 1, mRNA
3908	Q14137	3	6	7	746	83630	6.2	Ribosome biogenesis protein BOP1
3908	B2RDN4	3	6	7	746	83586	6.2	cDNA, FLJ96693, highly similar to sapiens block of proliferation 1 (BOP1), mRNA
3909	Q9NXH9-2	3	3	7.3	630	69306	7.2	Isoform 2 of N(2),N(2)-dimethylguanosine tRNA methyltransferase
3909	A8K8L2	3	3	7	659	72148	7.8	cDNA FLJ77676, highly similar to sapiens TRM1 tRNA methyltransferase 1 log (S. cerevisiae) (TRMT1), mRNA
3909	Q9NXH9	3	3	7	659	72234	7.6	N(2),N(2)-dimethylguanosine tRNA methyltransferase
3910	Q9UI10-3	3	3	7.3	522	57458	9.4	Isoform 3 of Translation initiation factor eIF-2B subunit delta
3910	Q9UI10	3	3	7.3	523	57557	9.4	Translation initiation factor eIF-2B subunit delta
3910	E7ERK9	3	3	7	544	59715	9.4	Uncharacterized protein
3910	Q9UI10-2	3	3	7	543	59616	9.4	Isoform 2 of Translation initiation factor eIF-2B subunit delta
3911	B2RAH1	3	5	6.9	726	80788	5.6	cDNA, FLJ94913, highly similar to sapiens holocarboxylase synthetase(biotin-[proprionyl-Coenzyme A-carboxylase (ATP-hydrolysing)]ligase) (HLCS), mRNA
3911	P50747	3	5	6.9	726	80760	5.6	Biotin--protein ligase
3912	B4E212	3	5	7.6	700	77235	7.7	Oxysterol-binding protein
3912	B3KUL5	3	5	6.9	764	83943	8.3	Oxysterol-binding protein
3912	Q9BXB5	3	5	6.9	764	83970	8.3	Oxysterol-binding protein-related protein 10
3913	O94804	3	3	6.9	968	112135	6.9	Serine/threonine-protein kinase 10
3913	Q6NSK0	3	3	6.9	968	112121	6.9	Serine/threonine kinase 10
3914	Q13188	3	4	7.1	491	56301	5.2	Serine/threonine-protein kinase 3
3914	B3KYA7	3	4	6.7	519	59462	5.3	cDNA FLJ16404 fis, clone UTERU2008019, highly similar to Serine/threonine-protein kinase 3 (EC 2.7.11.1)
3915	Q16222-2	3	4	6.9	505	57028	6.4	Isoform AGX1 of UDP-N-acetylhexosamine pyrophosphorylase
3915	Q16222	3	4	6.7	522	58769	6.3	UDP-N-acetylhexosamine pyrophosphorylase
3915	Q16222-3	3	4	6.7	521	58682	6.3	Isoform 3 of UDP-N-acetylhexosamine pyrophosphorylase
3916	B3KX22	3	3	6.5	934	107932	6.1	cDNA FLJ44514 fis, clone UTERU3002218
3916	Q96JG6	3	3	6.3	964	111174	6.2	Coiled-coil domain-containing protein 132
3917	Q4VBY2	3	5	10.4	521	57583	7.7	SEMA4B protein (Fragment)
3917	Q7Z3N1	3	5	7.9	681	75402	6.8	Putative uncharacterized protein DKFZp686A04130
3917	Q9NPR2-2	3	5	7.3	737	81403	6.2	Isoform 2 of Semaphorin-4B
3917	Q2NL81	3	5	6.5	837	92750	7	Sema domain, immunoglobulin domain (Ig), transmembrane domain (TM) and short cytoplasmic domain, (Semaphorin) 4B
3917	Q9NPR2	3	5	6.5	832	92193	6.9	Semaphorin-4B
3918	Q9H1I8	3	4	6.5	757	86360	5.2	Activating signal cointegrator 1 complex subunit 2
3919	P57740	3	4	6.4	925	106374	5.4	Nuclear pore complex protein Nup107
3920	B2RCD8	3	4	6.3	584	61714	8.2	cDNA, FLJ96012, highly similar to sapiens interferon regulatory factor 2 binding protein 1 (IRF2BP1), mRNA
3920	Q8IU81	3	4	6.3	584	61688	8.2	Interferon regulatory factor 2-binding protein 1
3921	Q96M96	3	8	7.2	766	86626	6.1	FYVE, RhoGEF and PH domain-containing protein 4
3921	B7Z8F9	3	8	6.3	878	98679	6.7	cDNA FLJ56188, highly similar to FYVE, RhoGEF and PH domain-containing protein4
3921	E9PJX4	3	8	6.3	878	98695	6.7	Uncharacterized protein
3922	E9PQ61	3	4	7.2	652	72532	8.6	Uncharacterized protein
3922	B4DLG2	3	4	6.2	756	82772	8.6	cDNA FLJ58196, highly similar to Zinc finger CCCH domain-containing protein 11A
3922	O75152	3	4	5.8	810	89131	8.4	Zinc finger CCCH domain-containing protein 11A
3923	P42898	3	3	6.2	656	74597	5.3	Methylenetetrahydrofolate reductase
3923	Q59GJ6	3	3	6.1	672	75049	6.3	Methylenetetrahydrofolate reductase (Fragment)
3923	Q5SNW6	3	3	5.9	697	78965	5.7	Methylenetetrahydrofolate reductase
3924	Q6PJT7-4	3	4	7.7	571	64502	8.2	Isoform 4 of Zinc finger CCCH domain-containing protein 14
3924	Q6PJT7-3	3	4	7.6	579	65425	7.9	Isoform 3 of Zinc finger CCCH domain-containing protein 14
3924	Q6PJT7-7	3	4	6.5	673	75763	7.2	Isoform 7 of Zinc finger CCCH domain-containing protein 14
3924	B5MD57	3	4	6.5	673	75777	7.2	Uncharacterized protein
3924	UPI0001AE69BB	3	4	6.2	711	80035	7.3	nuclear protein UKp68 isoform 3
3924	Q6PJT7-5	3	4	6.2	711	80021	7.3	Isoform 5 of Zinc finger CCCH domain-containing protein 14
3924	B4E2H4	3	4	6	730	82276	7.3	cDNA FLJ56206, highly similar to sapiens nuclear protein UKp68 , transcript variant 1, mRNA
3924	UPI0001A4717A	3	4	6	730	82312	7.3	zinc finger CCCH domain-containing protein 14 isoform 6
3924	Q6PJT7-2	3	4	6	735	82805	7.3	Isoform 2 of Zinc finger CCCH domain-containing protein 14
3924	Q6PJT7	3	4	6	736	82876	7.3	Zinc finger CCCH domain-containing protein 14
3924	B5MDV7	3	4	6	735	82819	7.3	Uncharacterized protein
3924	B5MBX9	3	4	6	736	82890	7.3	Uncharacterized protein
3925	B3KTC7	3	4	5.9	711	81070	6.2	cDNA FLJ38069 fis, clone CTONG2015434, highly similar to DOUBLE-STRAND BREAK REPAIR PROTEIN MRE11A
3925	P49959	3	4	5.9	708	80593	5.9	Double-strand break repair protein MRE11A
3926	O60518	3	21	5.9	1105	124713	5	Ran-binding protein 6
3927	Q9UPN7	3	3	5.8	881	96724	4.6	Serine/threonine-protein phosphatase 6 regulatory subunit 1
3928	Q9H6T3-2	3	3	6	631	71866	7.6	Isoform 2 of RNA polymerase II-associated protein 3
3928	Q9H6T3	3	3	5.7	665	75719	6.8	RNA polymerase II-associated protein 3
3929	Q9H6E5	3	4	5.6	874	93847	6.2	Speckle targeted PIP5K1A-regulated poly(A) polymerase
3930	Q12873	3	4	5.7	2000	226590	7.3	Chromodomain-helicase-DNA-binding protein 3
3930	E9PG89	3	4	5.5	2059	233035	6.2	Uncharacterized protein
3931	Q15003	3	5	5.5	741	82563	5.1	Condensin complex subunit 2
3932	Q9UBB6	3	4	5.5	729	78864	5.5	Neurochondrin
3932	Q9UBB6-3	3	4	5.5	731	79158	5.6	Isoform 3 of Neurochondrin
3933	Q9UNN5-2	3	8	7	498	56934	5.1	Isoform Short of FAS-associated factor 1
3933	A8K9X5	3	8	5.4	650	73920	4.9	cDNA FLJ76472, highly similar to sapiens Fas (TNFRSF6) associated factor 1 (FAF1), transcript variant 1, mRNA
3933	Q9UNN5	3	8	5.4	650	73954	4.9	FAS-associated factor 1
3934	E5RGH0	3	5	50.8	61	7252	4.6	Uncharacterized protein
3934	E9PN20	3	5	36.5	85	9937	4.9	Uncharacterized protein
3934	B4E159	3	5	23.5	132	15651	7.4	cDNA FLJ57767, moderately similar to Zinc finger protein 141
3934	C9JRN4	3	5	6.4	481	55703	9.2	Uncharacterized protein
3934	Q8TB69	3	5	5.7	540	63020	9.4	Zinc finger protein 519
3934	B4DXR9	3	5	5.3	585	67840	9	Zinc finger protein 732
3934	Q7Z3I0	3	5	4.8	648	74302	9.1	Putative uncharacterized protein DKFZp313E1411
3935	Q92888-2	3	4	5.7	879	98768	5.6	Isoform 2 of Rho guanine nucleotide exchange factor 1
3935	Q92888	3	4	5.5	912	102435	5.7	Rho guanine nucleotide exchange factor 1
3935	Q6NX52	3	4	5.3	948	105854	6.5	ARHGEF1 protein
3936	Q8N1G2	3	3	5.3	835	95321	7	Cap-specific mRNA (nucleoside-2'-O-)-methyltransferase 1
3937	Q9NV70	3	5	5.3	894	101982	6.6	Exocyst complex component 1
3937	Q9NV70-2	3	5	5.3	879	100283	6.6	Isoform 2 of Exocyst complex component 1
3938	Q96RU3-4	3	6	5.8	551	64056	5.2	Isoform 4 of Formin-binding protein 1
3938	B7ZL14	3	6	5.8	551	64071	5.2	FNBP1 protein
3938	Q5QP69	3	6	5.6	572	65815	6.1	Formin binding protein 1 (Fragment)
3938	Q96RU3-3	3	6	5.4	592	68883	5.8	Isoform 3 of Formin-binding protein 1

Table S1. Proteins identified in the OVCAR-3 tumor supernatant by two or more unique peptides.

Protein or Protein Group	Accession Number	Unique Peptide Count	Spectrum Count	Sequence Coverage (%)	Length	MolWt	pI	Descriptive Name
3938	E9PGQ4	3	6	5.3	608	70324	5.7	Uncharacterized protein
3938	B7ZL13	3	6	5.3	607	70199	5.7	FNBP1 protein
3938	B7ZL12	3	6	5.2	612	70752	5.7	FNBP1 protein
3938	UPI0001AE6E3F	3	6	5.2	615	71089	5.8	Formin-binding protein 1 (Formin-binding protein 17) (hFBP17).
3938	UPI0001AE6E3D	3	6	5.2	612	70861	5.7	Formin-binding protein 1 (Formin-binding protein 17) (hFBP17).
3938	Q96RU3-2	3	6	5.2	616	71184	5.8	Isoform 2 of Formin-binding protein 1
3938	Q96RU3	3	6	5.2	617	71307	5.7	Formin-binding protein 1
3939	Q6UWE0	3	7	5.1	723	83594	5.9	E3 ubiquitin-protein ligase LRSAM1
3940	UPI000173A297	3	10	14	328	35880	9.7	UPI000173A297 UniRef100 entry
3940	A0FGR8-5	3	10	14	328	35846	9.7	Isoform 5 of Extended synaptotagmin-2
3940	UPI000173A296	3	10	5.2	884	98010	8.6	F-box/LRR-repeat protein 18 (F-box and leucine-rich repeat protein 18).
3940	A0FGR8-2	3	10	5.2	893	98902	8.7	Isoform 2 of Extended synaptotagmin-2
3940	A0FGR8	3	10	5	921	102357	9.3	Extended synaptotagmin-2
3940	UPI0001AE715A	3	10	5	921	102372	9.3	UPI0001AE715A UniRef100 entry
3940	A0FGR8-6	3	10	4.9	942	104708	9.3	Isoform 6 of Extended synaptotagmin-2
3941	UPI0001B796D1	3	3	11.5	425	48034	8.6	UPI0001B796D1 UniRef100 entry
3941	A8K5B6	3	3	4.9	1009	113937	8.6	cDNA FLJ76830, highly similar to sapiens SWI/SNF related, matrix associated, actin dependent regulator of chromatin, subfamily a, member 3 (SMARCA3), transcript variant 1, mRNA
3941	Q59GQ7	3	3	4.9	992	111966	8.7	SWI/SNF-related matrix-associated actin-dependent regulator of chromatin a3 variant (Fragment)
3941	Q14527	3	3	4.9	1009	113928	8.6	Helicase-like transcription factor
3942	E7EUZ3	3	9	4.9	918	101679	4.7	Uncharacterized protein
3942	Q14966	3	9	2.3	1978	220623	6.4	Zinc finger protein 638
3943	C9JHZ3	3	3	4.5	740	82176	9.8	Uncharacterized protein
3943	Q14684-2	3	3	4.5	740	82176	9.8	Isoform 2 of Ribosomal RNA processing protein 1 log B
3943	Q14684	3	3	4.4	758	84428	9.8	Ribosomal RNA processing protein 1 log B
3944	B7Z6Y7	3	3	4.8	924	104545	7.1	cDNA FLJ53412, highly similar to DNA-directed RNA polymerase I 135 kDa polypeptide (EC 2.7.7.6)
3944	Q2TAM4	3	3	4.6	952	107812	7.7	DNA-directed RNA polymerase
3944	E9PB89	3	3	4.6	952	107858	7.6	Uncharacterized protein
3944	B7Z1W6	3	3	4.4	996	112852	7.5	DNA-directed RNA polymerase
3944	Q9H9Y6-2	3	3	4.1	1079	121988	7.6	Isoform 2 of DNA-directed RNA polymerase I subunit RPA2
3944	Q9H9Y6	3	3	3.9	1135	128229	7.8	DNA-directed RNA polymerase I subunit RPA2
3944	B7Z823	3	3	3.8	1173	132168	8	DNA-directed RNA polymerase
3945	Q13751	3	5	4.4	1172	129572	7.2	Laminin subunit beta-3
3946	A2RU29	3	4	4.3	938	106998	5.6	MORC3 protein
3946	Q4VBZ9	3	4	4.3	939	107041	5.6	MORC family CW-type zinc finger 3
3946	Q14149	3	4	4.3	939	107113	5.6	MORC family CW-type zinc finger protein 3
3946	E9PGS1	3	4	4.3	940	107241	5.6	Uncharacterized protein
3947	A8KAQ3	3	3	4.3	831	92037	5.8	cDNA FLJ76489, highly similar to sapiens sortilin 1 (SORT1), mRNA
3947	Q99523	3	3	4.3	831	92068	5.8	Sortilin
3948	Q53TB6	3	3	9.9	435	48454	4.7	Putative uncharacterized protein MDA5 (Fragment)
3948	A8KA76	3	3	4.2	1025	116630	5.5	cDNA FLJ78660, highly similar to sapiens interferon induced with helicase C domain 1 (IFIH1), mRNA
3948	Q9BYX4	3	3	4.2	1025	116689	5.5	Interferon-induced helicase C domain-containing protein 1
3949	B4DKV5	3	3	5.3	718	81460	5.4	cDNA FLJ55614
3949	UPI000013D26F	3	3	4.3	891	101065	5.4	UPI000013D26F UniRef100 entry
3949	Q96EK7	3	3	4.2	910	103783	5.7	Constitutive coactivator of peroxisome proliferator-activated receptor gamma
3949	B4DG54	3	3	4.1	922	105248	5.8	cDNA FLJ56635
3949	B4DSS4	3	3	4.1	933	106116	5.9	cDNA FLJ56631
3950	O75170-6	3	6	4.3	905	98126	4.9	Isoform 6 of Serine/threonine-protein phosphatase 6 regulatory subunit 2
3950	O75170-4	3	6	4.2	932	101246	4.9	Isoform 4 of Serine/threonine-protein phosphatase 6 regulatory subunit 2
3950	O75170-3	3	6	4.2	933	101402	4.9	Isoform 3 of Serine/threonine-protein phosphatase 6 regulatory subunit 2
3950	O75170-2	3	6	4.2	927	101007	4.9	Isoform 2 of Serine/threonine-protein phosphatase 6 regulatory subunit 2
3950	O75170-5	3	6	4.1	959	104235	4.9	Isoform 5 of Serine/threonine-protein phosphatase 6 regulatory subunit 2
3950	O75170	3	6	4	966	104942	4.9	Serine/threonine-protein phosphatase 6 regulatory subunit 2
3951	Q6P2E9	3	9	3.9	1401	151661	5.9	Enhancer of mRNA-decapping protein 4
3952	B4DDL3	3	3	3.8	999	113963	6	cDNA FLJ55430, highly similar to Serologically defined colon cancer antigen 1 (Fragment)
3952	B4E3F3	3	3	3.6	1055	120776	6.5	cDNA FLJ61574, highly similar to Serologically defined colon cancer antigen 1
3952	UPI00001AA2F0	3	3	3.5	1076	122970	6.3	serologically defined colon cancer antigen 1
3952	O60524	3	3	3.5	1076	122954	6.3	Serologically defined colon cancer antigen 1
3953	E9PBL8	3	3	3.7	940	104787	7.2	Uncharacterized protein
3953	Q4G0L8	3	3	3.7	940	104774	7.2	COG1 protein
3953	Q8WTW3	3	3	3.6	980	108978	7.3	Conserved oligomeric Golgi complex subunit 1
3954	Q9P0U0	3	3	6.2	533	58974	4.8	PC326 protein
3954	Q58WW2-2	3	3	3.8	880	98423	5.2	Isoform 2 of DDB1- and CUL4-associated factor 6
3954	Q58WW2	3	3	3.8	860	96292	5.3	DDB1- and CUL4-associated factor 6
3954	B4DNB8	3	3	3.6	920	103070	5.4	cDNA FLJ58595, highly similar to sapiens IQ motif and WD repeats 1 (IQWD1), transcript variant 1, mRNA
3954	Q58WW2-3	3	3	3.5	951	106542	5.4	Isoform 3 of DDB1- and CUL4-associated factor 6
3955	Q9UIQ6-2	3	4	3.6	1011	115636	5.7	Isoform 2 of Leucyl-cystinyl aminopeptidase
3955	B2RAK1	3	4	3.5	1025	117345	5.7	cDNA, FLJ94965, highly similar to sapiens leucyl/cystinyl aminopeptidase (LNPEP), mRNA
3955	Q9UIQ6	3	4	3.5	1025	117349	5.7	Leucyl-cystinyl aminopeptidase
3956	E7ESU4	3	7	3.5	953	107297	7	Uncharacterized protein
3956	UPI0001929506	3	7	3.5	953	107271	7	N-acetyltransferase 10 isoform b
3956	Q9H0A0	3	7	3.2	1025	115730	8.3	N-acetyltransferase 10
3957	Q01804	3	3	3.2	1113	123916	6.7	OTU domain-containing protein 4
3957	UPI0000E825C1	3	3	3.2	1114	124045	6.7	UPI0000E825C1 UniRef100 entry
3958	A8K6X9	3	3	3.1	1173	133530	6.8	cDNA FLJ76427, highly similar to sapiens SH2 domain binding protein 1 (tetratricopeptide repeat containing) (SH2BP1), mRNA
3958	Q6PD62	3	3	3.1	1173	133502	6.8	RNA polymerase-associated protein CTR9 log
3959	Q9ULK4-5	3	4	3.2	1364	156082	7.4	Isoform 5 of Mediator of RNA polymerase II transcription subunit 23
3959	Q9ULK4-3	3	4	3.2	1365	156193	7.4	Isoform 3 of Mediator of RNA polymerase II transcription subunit 23
3959	E7EX31	3	4	3.1	1374	157114	7.4	Uncharacterized protein
3959	Q9ULK4	3	4	3.1	1368	156473	7.4	Mediator of RNA polymerase II transcription subunit 23
3959	Q5JWT2	3	4	3.1	1374	157113	7.5	Cofactor required for Sp1 transcriptional activation, subunit 3, 130kDa
3960	Q24JQ7	3	3	3	1006	109037	8.7	ATXN2 protein
3960	Q99700-2	3	3	3	995	106048	9.7	Isoform 2 of Ataxin-2

Table S1. Proteins identified in the OVCAR-3 tumor supernatant by two or more unique peptides.

Protein or Protein Group	Accession Number	Unique Peptide Count	Spectrum Count	Sequence Coverage (%)	Length	MolWt	pI	Descriptive Name
3960	UPI0000E5996A	3	3	2.9	1050	113882	8.8	Ataxin-2 (Spinocerebellar ataxia type 2 protein) (Trinucleotide repeat-containing gene 13 protein).
3960	Q2M2R5	3	3	2.7	1127	122181	8.7	ATXN2 protein (Fragment)
3960	Q99700	3	3	2.3	1313	140283	9.6	Ataxin-2
3961	P23468	3	3	2.9	1912	214758	6.6	Receptor-type tyrosine-protein phosphatase delta
3961	UPI0000EE416B	3	3	2.9	1890	212158	6.7	Receptor-type tyrosine-protein phosphatase delta precursor (EC 3.1.3.48) (Protein-tyrosine phosphatase delta) (R-PTP-delta).
3961	Q2HXI4	3	3	2.9	1912	214784	6.6	Protein tyrosine phosphatase receptor type D
3961	P23468-2	3	3	2.9	1890	212131	6.7	Isoform 2 of Receptor-type tyrosine-protein phosphatase delta
3962	B4E2S2	3	3	2.8	1005	112792	5.6	cDNA FLJ61556, highly similar to Poly (ADP-ribose) polymerase 4 (EC 2.4.2.30)
3962	Q9UKK3	3	3	1.6	1724	192593	5.7	Poly [ADP-ribose] polymerase 4
3963	Q5IJ48-3	3	3	3.8	953	99830	6.2	Isoform 3 of Crumbs log 2
3963	Q5IJ48-2	3	3	3.1	1176	123213	5.6	Isoform 2 of Crumbs log 2
3963	Q5IJ48	3	3	2.8	1285	134265	5.6	Crumbs log 2
3964	Q9UPT8	3	6	2.8	1303	140256	6.3	Zinc finger CCCH domain-containing protein 4
3965	Q9HAU0-5	3	6	3.3	1060	121272	8.1	Isoform 5 of Pleckstrin logy domain-containing family A member 5
3965	Q9HAU0	3	6	3.1	1116	127464	7.5	Pleckstrin logy domain-containing family A member 5
3965	Q9HAU0-4	3	6	3	1179	134593	7.3	Isoform 4 of Pleckstrin logy domain-containing family A member 5
3965	Q9HAU0-2	3	6	3	1174	134323	7.6	Isoform 2 of Pleckstrin logy domain-containing family A member 5
3965	E9PHQ3	3	6	2.7	1277	146394	7.7	Uncharacterized protein
3966	P16885	3	3	2.7	1265	147870	6.6	1-phosphatidylinositol-4,5-bisphosphate phosphodiesterase gamma-2
3967	O95104-2	3	11	2.7	1125	123643	9.6	Isoform 2 of Splicing factor, arginine/serine-rich 15
3967	C9JD39	3	11	2.7	1125	123645	9.6	Uncharacterized protein
3967	C9J1W7	3	11	2.6	1147	125871	9.6	Uncharacterized protein
3967	Q0P607	3	11	2.6	1146	125740	9.6	Splicing factor, arginine/serine-rich 15
3967	O95104	3	11	2.6	1147	125869	9.6	Splicing factor, arginine/serine-rich 15
3968	A6H8X9	3	5	2.5	1460	161437	7	Centrosomal protein 170kDa
3968	Q5SW79-3	3	5	2.5	1486	164538	7.2	Isoform 3 of Centrosomal protein of 170 kDa
3968	Q5SW79-2	3	5	2.5	1460	161407	7	Isoform 2 of Centrosomal protein of 170 kDa
3968	B2RTS4	3	5	2.5	1486	164568	7.2	Centrosomal protein 170kDa
3968	Q5SW79	3	5	2.3	1584	175292	7.1	Centrosomal protein of 170 kDa
3968	E9PEY0	3	5	2.3	1624	179628	7.2	Uncharacterized protein
3969	Q59EE6	3	7	2.4	1700	181811	5	Latent transforming growth factor beta binding protein 2 variant (Fragment)
3969	Q14767	3	7	2.3	1821	195051	5.2	Latent-transforming growth factor beta-binding protein 2
3969	Q6AZ94	3	7	2.3	1821	195037	5.2	Latent transforming growth factor beta binding protein 2
3970	Q59H44	3	5	2.6	1340	154596	7	Lymphocyte antigen 75 variant (Fragment)
3970	O60449	3	5	2	1722	198310	6.7	Lymphocyte antigen 75
3970	O60449-3	3	5	1.9	1817	209437	6.2	Isoform 3 of Lymphocyte antigen 75
3970	O60449-2	3	5	1.9	1873	215779	6.2	Isoform 2 of Lymphocyte antigen 75
3971	UPI0001662CC8	3	3	1.7	2934	336125	6.5	PREDICTED: serine-protein kinase ATM-like, partial
3971	Q13315	3	3	1.6	3056	350714	6.8	Serine-protein kinase ATM
3972	P98164	3	4	1.1	4655	521961	5.1	Low-density lipoprotein receptor-related protein 2
3972	Q7Z5C1	3	4	1.1	4655	521992	5.1	Glycoprotein receptor gp330/megalin
3972	Q7Z5C0	3	4	1.1	4655	521935	5.1	Glycoprotein receptor gp330/megalin
3973	Q12830-4	3	5	1.2	2903	322217	6.4	Isoform 4 of Nucleosome-remodeling factor subunit BPTF
3973	Q12830-2	3	5	1.2	2920	325123	7.1	Isoform 2 of Nucleosome-remodeling factor subunit BPTF
3973	Q12830	3	5	1.1	3046	338262	6.5	Nucleosome-remodeling factor subunit BPTF
3974	Q7RTM4	3	5	0.6	8407	966361	5.6	Spectrin-like protein of the nuclear envelope and Golgi
3974	E7ENN3	3	5	0.6	8409	966670	5.6	Uncharacterized protein
3974	B7ZBC3	3	5	0.5	8797	1011054	5.5	Spectrin repeat containing, nuclear envelope 1
3974	Q8NF91-4	3	5	0.5	8749	1005204	5.5	Isoform 4 of Nesprin-1
3974	Q8NF91	3	5	0.5	8797	1011050	5.5	Nesprin-1
3974	Q5JV23	3	5	0.5	8749	1005208	5.5	Spectrin repeat containing, nuclear envelope 1
3974	E9PEL9	3	5	0.5	8749	1005226	5.5	Uncharacterized protein
3974	E7EQI5	3	5	0.5	8797	1011072	5.5	Uncharacterized protein
3975	E9PAM3	2	3	85.5	62	6850	4.3	Uncharacterized protein
3975	Q9Y4Y9	2	3	58.2	91	9937	4.5	U6 snRNA-associated Sm-like protein LSm5 U6 snRNA-associated Sm-like protein LSm5
3976	Q71U36	2	46	77.2	451	50136	5.1	Tubulin alpha-1A chain Tubulin alpha-1A chain
3977	P24844	2	3	55.8	172	19827	4.9	Myosin regulatory light polypeptide 9
3977	Q9CQ19	2	3	55.8	172	19854	4.9	Myosin regulatory light polypeptide 9
3977	Q6IBG1	2	3	55.8	172	19849	5.1	MYL9 protein
3978	UPI000198C9D7	2	7	56.3	151	16928	7.7	UPI000198C9D7 UniRef100 entry
3978	C9J8K2	2	7	53.1	160	18000	8.8	Ubiquitin carrier protein
3978	P52482	2	7	44	193	21333	8.5	Ubiquitin-conjugating enzyme E2 E1
3978	P51965	2	7	44	193	21404	8.5	Ubiquitin-conjugating enzyme E2 E1
3979	D3YU44	2	4	52.9	68	7295	9.5	Uncharacterized protein
3979	UPI000012B8BE	2	4	52.9	68	7251	9.1	Guanine nucleotide-binding protein G(I)/G(S)/G(O) gamma-5-like subunit.
3979	P63218	2	4	52.9	68	7318	9.9	Guanine nucleotide-binding protein G(I)/G(S)/G(O) subunit gamma-5 Guanine nucleotide-binding protein G(I)/G(S)/G(O) subunit gamma-5
3980	Q16777	2	98	49.6	129	13988	10.9	Histone H2A type 2-C Histone H2A type 2-C
3980	Q6FI13	2	98	49.2	130	14095	10.9	Histone H2A type 2-A Histone H2A type 2-A
3980	UPI0001E6B57B	2	98	22	291	32213	6.8	PREDICTED: src substrate cortactin
3981	P16104	2	11	47.6	143	15145	10.7	Histone H2A.x
3981	P27661	2	11	47.6	143	15143	10.7	Histone H2A.x
3982	P59768	2	4	42.3	71	7850	8	Guanine nucleotide-binding protein G(I)/G(S)/G(O) subunit gamma-2 Guanine nucleotide-binding protein G(I)/G(S)/G(O) subunit gamma-2
3983	P08134	2	21	49.7	193	22006	6.6	Rho-related GTP-binding protein RhoC Rho-related GTP-binding protein RhoC
3983	E9PN11	2	21	41.9	229	25485	5.6	Uncharacterized protein
3984	P60763	2	4	40.1	192	21379	8.2	Ras-related C3 botulinum toxin substrate 3 Ras-related C3 botulinum toxin substrate 3
3985	Q13509	2	2	39.6	450	50433	4.9	Tubulin beta-3 chain
3985	Q9ERD7	2	2	39.6	450	50419	4.9	Tubulin beta-3 chain
3986	P31786	2	10	39.1	87	10000	8.8	Acyl-CoA-binding protein Acyl-CoA-binding protein
3986	Q3ULV8	2	10	39.1	87	9995	8.3	Putative uncharacterized protein
3986	UPI0000F220C0	2	10	33.7	101	11548	8.2	UPI0000F220C0 UniRef100 entry
3986	Q4VWZ5	2	10	25.2	135	15229	9.1	Diazepam binding inhibitor, splice form 1b
3987	Q13951	2	3	39	182	21508	6.6	Core-binding factor subunit beta
3987	Q66HA7	2	3	39	182	21517	6.8	Core-binding factor, beta subunit
3988	P11908	2	3	38.4	318	34769	6.6	Ribose-phosphate pyrophosphokinase 2
3988	Q9CS42	2	3	38.4	318	34786	6.6	Ribose-phosphate pyrophosphokinase 2
3988	P11908-2	2	3	38	321	35054	6.4	Isoform 2 of Ribose-phosphate pyrophosphokinase 2
3989	UPI00004DC55A	2	8	51.4	109	12421	7.9	ubiquitin-conjugating enzyme E2 D1 isoform 2

Table S1. Proteins identified in the OVCAR-3 tumor supernatant by two or more unique peptides.

Protein or Protein Group	Accession Number	Unique Peptide Count	Spectrum Count	Sequence Coverage (%)	Length	MolWt	pI	Descriptive Name
3989	P51668	2	8	38.1	147	16602	7.4	Ubiquitin-conjugating enzyme E2 D1 Ubiquitin-conjugating enzyme E2 D1
3990	Q3TJ84	2	11	35.5	124	14776	4.1	Putative uncharacterized protein
3990	Q9D6V8	2	11	35.5	124	14700	4.1	Polyadenylate-binding protein-interacting protein 2
3990	Q3U438	2	11	35.5	124	14749	4.1	Putative uncharacterized protein
3990	Q9BPZ3	2	11	34.6	127	14984	4.1	Polyadenylate-binding protein-interacting protein 2
3991	Q78YZ6-2	2	10	53.7	82	9355	4.7	Isoform 2 of Short coiled-coil protein
3991	Q9UIL1-3	2	10	36.4	121	13857	4.8	Isoform 3 of Short coiled-coil protein Isoform 3 of Short coiled-coil protein
3991	Q9UIL1-2	2	10	36.1	122	13929	4.8	Isoform 2 of Short coiled-coil protein
3991	Q78YZ6	2	10	35.2	125	14155	4.7	Short coiled-coil protein
3991	UPI000199A558	2	10	27.8	158	17974	8.8	short coiled-coil protein isoform 2
3991	Q9UIL1	2	10	27.7	159	18045	8.8	Short coiled-coil protein
3992	Q9CX60	2	2	34.3	105	12138	4.3	Protein LBH
3992	Q5RD13	2	2	34.3	105	12218	4.3	Protein LBH
3992	Q53QV2	2	2	34.3	105	12217	4.4	Protein LBH
3992	B5MCP4	2	2	32.4	111	12585	4.4	Uncharacterized protein
3993	P63146	2	6	32.2	152	17312	5	Ubiquitin-conjugating enzyme E2 B Ubiquitin-conjugating enzyme E2 B
3994	Q9BRV4	2	16	33	100	11310	6.8	Vesicle-associated membrane protein 3 (Cellubrevin)
3994	Q15836	2	16	33	100	11309	8.8	Vesicle-associated membrane protein 3
3994	P63024	2	16	32	103	11480	8.5	Vesicle-associated membrane protein 3
3995	C9J7M8	2	2	31.7	123	13172	8.8	Uncharacterized protein
3995	Q9EQH4-5	2	2	17.6	221	24361	8.6	Isoform 5 of Transcription initiation factor TFIID subunit 8
3995	E9Q8H2	2	2	17.6	222	24467	8.2	Uncharacterized protein
3995	Q9EQH4-3	2	2	14.7	265	29196	5.8	Isoform 3 of Transcription initiation factor TFIID subunit 8
3995	E9Q367	2	2	14.7	265	29243	5.5	Uncharacterized protein
3995	Q9EQH4	2	2	12.7	308	33988	6.4	Transcription initiation factor TFIID subunit 8
3995	Q7Z7C8	2	2	12.6	310	34262	6.4	Transcription initiation factor TFIID subunit 8
3995	Q7Z7C8-2	2	2	12.5	311	34436	6.2	Isoform 2 of Transcription initiation factor TFIID subunit 8
3995	Q9EQH4-4	2	2	12.1	322	35440	6.3	Isoform 4 of Transcription initiation factor TFIID subunit 8
3995	Q7Z7C8-4	2	2	11.5	338	37411	8.4	Isoform 3 of Transcription initiation factor TFIID subunit 8
3996	Q9Y3L5	2	9	31.1	183	20745	4.9	Ras-related protein Rap-2c Ras-related protein Rap-2c
3997	A1L0U3	2	70	30.8	133	15030	11.1	Histone H3 (Fragment)
3997	Q71DI3	2	70	30.1	136	15388	11.3	Histone H3.2 Histone H3.2
3998	D2HCJ0	2	5	30.6	62	7763	7.2	Putative uncharacterized protein (Fragment)
3998	UPI00006075BD	2	5	24.1	79	9846	7.7	PREDICTED: cyclin-dependent kinases regulatory subunit 2-like
3998	UPI0000604B9A	2	5	24.1	79	9917	9	PREDICTED: cyclin-dependent kinases regulatory subunit 2-like
3998	P56390	2	5	24.1	79	9874	8.5	Cyclin-dependent kinases regulatory subunit 2
3998	P33552	2	5	24.1	79	9860	8.5	Cyclin-dependent kinases regulatory subunit 2
3999	Q9HB72	2	4	30.3	76	8468	10.2	PNAS-106
3999	Q9Y5V0	2	4	30.3	76	8498	10	Zinc finger protein 706 Zinc finger protein 706
4000	C9JTN7	2	4	29.5	285	31625	8.3	Uncharacterized protein
4000	Q8CII5	2	4	29.5	285	31581	8.3	Tia1 protein
4000	P31483-2	2	4	22.4	375	41801	7.8	Isoform Short of Nucleolysin TIA-1 isoform p40
4000	Q80ZW7	2	4	22.3	377	41893	8	Tia1 protein
4001	Q9WV98	2	3	29.2	89	10344	7.2	Mitochondrial import inner membrane translocase subunit Tim9
4001	Q9Y5J7	2	3	29.2	89	10378	7.2	Mitochondrial import inner membrane translocase subunit Tim9
4002	O14907	2	7	28.2	124	13735	8.5	Tax1-binding protein 3
4002	UPI0000604485	2	7	28.2	124	13783	7.4	PREDICTED: tax1-binding protein 3-like isoform 2
4002	Q9DBG9	2	7	28.2	124	13723	8.5	Tax1-binding protein 3
4003	Q9NPA8	2	6	25.7	101	11529	9.3	Enhancer of yellow 2 transcription factor log Enhancer of yellow 2 transcription factor log
4004	Q9QZV9	2	5	25	140	15847	5	NTF2-related export protein 1
4004	Q9UKK6	2	5	25	140	15847	5	NTF2-related export protein 1
4005	Q96DE5	2	2	24.5	110	11667	5	Anaphase-promoting complex subunit 16 Anaphase-promoting complex subunit 16
4006	D3Z4V3	2	3	24.4	90	10443	8.4	Uncharacterized protein
4006	Q9Y6I3-3	2	3	4	550	57504	4.9	Isoform 3 of Epsin-1
4006	Q9Y6I3	2	3	4	551	57575	4.9	Epsin-1
4006	Q9Y6I3-2	2	3	3.8	576	60293	4.8	Isoform 2 of Epsin-1
4006	Q80VP1-2	2	3	3.8	576	60283	4.8	Isoform 2 of Epsin-1
4006	Q80VP1	2	3	3.8	575	60212	4.8	Epsin-1
4007	B2R5J1	2	4	23.9	117	13918	8.8	cDNA, FLJ92494, highly similar to sapiens GABA(A) receptor-associated protein (GABARAP), mRNA
4007	O95166	2	4	23.9	117	13918	8.8	Gamma-aminobutyric acid receptor-associated protein Gamma-aminobutyric acid receptor-associated protein
4008	D6RCG4	2	7	56.6	76	8727	6.8	Uncharacterized protein
4008	Q96B02	2	7	28.5	151	17331	7.8	Probable ubiquitin-conjugating enzyme E2 W
4008	Q8VDW4	2	7	28.5	151	17345	7.8	Probable ubiquitin-conjugating enzyme E2 W
4008	Q3V321	2	7	27.9	154	17411	9	Putative uncharacterized protein
4008	UPI0001E8F39A	2	7	27.7	155	17779	8.2	UPI0001E8F39A UniRef100 entry
4008	Q1XBE0	2	7	26.5	162	18660	8.1	Ubiquitin-conjugating enzyme 16
4008	C9JS56	2	7	26.5	162	18637	7.8	Uncharacterized protein
4008	B4DIV1	2	7	23.9	180	20689	9	Ubiquitin-conjugating enzyme E2W (Putative), isoform CRA_a
4009	Q7Z422-2	2	3	25	132	14605	11.3	Isoform 2 of UPF0485 protein C1orf144
4009	A2AA86	2	3	25	132	14634	11.5	Novel protein (D4Ertd22e)
4009	Q7Z422-4	2	3	24.8	133	14761	11.4	Isoform 4 of UPF0485 protein C1orf144
4009	Q6NXN1-2	2	3	24.8	133	14790	11.5	Isoform 2 of UPF0485 protein C1orf144 log
4009	Q7Z422-3	2	3	24.6	134	14744	5.7	Isoform 3 of UPF0485 protein C1orf144
4009	A2AA85	2	3	22.6	146	16277	9.8	Novel protein (D4Ertd22e) (Fragment)
4009	C9K0U0	2	3	21.9	151	16910	9	Uncharacterized protein
4009	Q7Z422	2	3	21.7	152	16997	9	UPF0485 protein C1orf144
4009	Q6NXN1	2	3	21.7	152	16998	9	UPF0485 protein C1orf144 log
4010	P62072	2	6	22.2	90	10333	6.3	Mitochondrial import inner membrane translocase subunit Tim10 Mitochondrial import inner membrane translocase subunit Tim10
4011	Q9BV40	2	2	22	100	11438	7.4	Vesicle-associated membrane protein 8
4011	O70404	2	2	21.8	101	11451	8.2	Vesicle-associated membrane protein 8
4012	Q80Y14	2	3	21.7	152	16292	6.5	Glutaredoxin-related protein 5, mitochondrial
4012	Q86SX6	2	3	21	157	16628	6.8	Glutaredoxin-related protein 5, mitochondrial
4013	Q9DBW3	2	3	21.8	110	12731	8.5	Protein GTLF3B
4013	Q8N6N6	2	3	21.2	113	13032	8.6	Protein GTLF3B
4014	O35207	2	8	20.2	114	12354	9.4	Cyclin-dependent kinase 2-associated protein 1
4014	O14519	2	8	20	115	12365	9.4	Cyclin-dependent kinase 2-associated protein 1
4015	A0PJI3	2	7	19.7	203	23112	9.8	Ppp1r10 protein (Fragment)
4015	Q58F28	2	7	12.8	312	34734	9.9	PPP1R10 protein (Fragment)
4015	Q80W00-2	2	7	4.6	874	93052	9.2	Isoform 2 of Serine/threonine-protein phosphatase 1 regulatory subunit 10
4015	E9QPZ8	2	7	4.6	874	93025	9.2	Uncharacterized protein
4015	Q80W00	2	7	4.5	888	94372	9.2	Serine/threonine-protein phosphatase 1 regulatory subunit 10
4015	Q96QC0	2	7	4.3	940	99058	9.2	Serine/threonine-protein phosphatase 1 regulatory subunit 10
4016	B5BU61	2	2	19.7	482	55076	5.5	Histone deacetylase
4016	Q13547	2	2	19.7	482	55103	5.5	Histone deacetylase 1
4016	O09106	2	2	19.7	482	55075	5.5	Histone deacetylase 1

Table S1. Proteins identified in the OVCAR-3 tumor supernatant by two or more unique peptides.

Protein or Protein Group	Accession Number	Unique Peptide Count	Spectrum Count	Sequence Coverage (%)	Length	MolWt	pI	Descriptive Name
4016	D3YYI8	2	2	19.7	482	55048	5.4	Histone deacetylase
4017	B2R5H5	2	4	19.6	102	11829	4.7	cDNA, FLJ92476, highly similar to sapiens LSM3 log, U6 small nuclear RNA associated (S. cerevisiae) (LSM3), mRNA
4017	P62310	2	4	19.6	102	11845	4.7	U6 snRNA-associated Sm-like protein LSm3 U6 snRNA-associated Sm-like protein LSm3
4018	A2A9C8	2	3	19.4	129	14256	9.1	Mediator of RNA polymerase II transcription, subunit 8 log (Yeast)
4018	Q9CY05	2	3	14	179	18976	9.1	Mediator of RNA polymerase II transcription, subunit 8 log (Yeast)
4018	Q96G25-3	2	3	14	179	19002	9.1	Isoform 3 of Mediator of RNA polymerase II transcription subunit 8
4018	Q9DAY7	2	3	10.7	234	25952	6.9	Putative uncharacterized protein
4018	Q9D7W5	2	3	9.3	268	29199	7	Mediator of RNA polymerase II transcription subunit 8
4018	Q96G25	2	3	9.3	268	29080	7.5	Mediator of RNA polymerase II transcription subunit 8
4019	D6R9Q5	2	2	19.3	109	12346	8.4	Uncharacterized protein
4019	D6RD74	2	2	16.7	126	14218	7.5	Uncharacterized protein
4019	Q9UM13	2	2	11.4	185	21252	9.1	Anaphase-promoting complex subunit 10
4019	Q8K2H6	2	2	11.4	185	21266	9.1	Anaphase-promoting complex subunit 10 Anaphase-promoting complex subunit 10
4020	D3YZ20	2	3	18.9	111	12585	6.8	Uncharacterized protein
4020	Q9GZM3	2	3	18.3	115	13074	6.3	DNA-directed RNA polymerase II subunit RPB11-b1
4020	P52435	2	3	17.9	117	13293	5.9	DNA-directed RNA polymerase II subunit RPB11-a DNA-directed RNA polymerase II subunit RPB11-a
4020	O08740	2	3	17.9	117	13251	5.9	DNA-directed RNA polymerase II subunit RPB11
4020	Q9H1A6	2	3	16.5	127	14131	5.9	RPB11a protein
4020	E2QRJ6	2	3	13.3	158	17190	5.5	Uncharacterized protein
4020	Q59FR2	2	3	11.7	180	19038	6.7	MGC13098 protein variant (Fragment)
4021	P24390	2	3	18.9	212	24542	8.6	ER lumen protein retaining receptor 1
4021	Q99JH8	2	3	18.9	212	24560	8.6	ER lumen protein retaining receptor 1
4022	D3Z497	2	7	18.8	112	12652	6.7	Uncharacterized protein
4022	B2R4X4	2	7	18.6	113	12741	5.2	cDNA, FLJ92255, highly similar to sapiens ring finger protein 7 (RNF7), mRNA
4022	UPI0001552CE3	2	7	18.6	113	12706	5.8	PREDICTED: RING-box protein 2-like
4022	UPI00006069DC	2	7	18.6	113	12734	5.5	PREDICTED: RING-box protein 2-like
4022	Q9WTZ1	2	7	18.6	113	12707	5.5	RING-box protein 2
4022	Q9UBF6	2	7	18.6	113	12683	5.5	RING-box protein 2
4023	Q9Y5U9	2	3	34.1	82	8969	8.3	Immediate early response 3-interacting protein 1
4023	Q9CR20	2	3	34.1	82	9017	8.3	Immediate early response 3-interacting protein 1
4023	D6RI20	2	3	18.4	152	16254	8.7	Uncharacterized protein
4024	O96015	2	3	18.1	105	12009	5.5	Dynein light chain 4, axonemal
4024	Q9DCM4	2	3	18.1	105	12023	5.5	Dynein light chain 4, axonemal
4024	Q6FGB2	2	3	18.1	105	11995	5.2	DNAL4 protein (Fragment)
4024	Q53FE0	2	3	18.1	105	11937	5.8	Dynein light chain 4, axonemal variant (Fragment)
4024	Q8CDT8	2	3	13.7	139	15876	6.5	Dynein, axonemal, light chain 4, isoform CRA_c
4025	Q13595-2	2	7	28.3	113	12952	10	Isoform Short of Transformer-2 protein log alpha Isoform Short of Transformer-2 protein log alpha
4025	B4DQI6	2	7	17.8	180	20590	10.2	cDNA FLJ50614, highly similar to Transformer-2 protein log
4025	B4DUA9	2	7	17.7	181	20746	10.3	cDNA FLJ50682, highly similar to Transformer-2 protein log
4025	Q6PFR5	2	7	11.4	281	32316	11.3	Transformer-2 protein log alpha
4025	Q13595	2	7	11.3	282	32689	11.3	Transformer-2 protein log alpha
4025	E9QP00	2	7	11.3	282	32579	11.3	Uncharacterized protein
4026	B3KN29	2	3	16.3	178	19290	9.2	cDNA FLJ13371 fis, clone PLACE1000656, highly similar to PRA1 family protein 2
4026	Q9JIG8	2	3	16.3	178	19478	9.6	PRA1 family protein 2
4026	O60831	2	3	16.3	178	19258	9.2	PRA1 family protein 2
4027	UPI000021097A	2	3	17.5	126	14548	7	Golgin subfamily A member 7 (Golgi complex-associated protein of 16 kDa).
4027	UPI00001B2F27	2	3	16.4	134	15578	8.4	Golgin subfamily A member 7 isoform b
4027	Q7Z5G4	2	3	16.1	137	15824	7	Golgin subfamily A member 7
4027	Q91W53	2	3	16.1	137	15792	7	Golgin subfamily A member 7
4027	Q91W53-2	2	3	14.4	153	17549	7.3	Isoform 2 of Golgin subfamily A member 7
4028	Q5T092	2	4	18.4	158	18389	7.4	RER1 retention in endoplasmic reticulum 1 log (S. cerevisiae) (Fragment)
4028	Q5T091	2	4	15.5	187	21024	8.7	RER1 retention in endoplasmic reticulum 1 log (S. cerevisiae)
4028	O15258	2	4	14.8	196	22958	9.5	Protein RER1
4028	Q9CQU3	2	4	14.8	196	22988	9.5	Protein RER1
4028	Q9P0H9	2	4	13.6	214	24817	9.6	RER1 protein
4029	P55854	2	29	20.4	103	11637	5.5	Small ubiquitin-related modifier 3
4029	Q9Z172-2	2	29	19.1	110	12345	5.9	Isoform 2 of Small ubiquitin-related modifier 3
4029	Q9Z172	2	29	19.1	110	12430	5.9	Small ubiquitin-related modifier 3
4029	A8MUA9	2	29	15.6	135	15318	10.4	SMT3 suppressor of mif two 3 log 3 (Yeast), isoform CRA_d
4029	B4DUW4	2	29	14.9	141	15768	6.9	cDNA FLJ57440, moderately similar to Small ubiquitin-related modifier 3
4029	A8MU27	2	29	14.3	147	16956	9.7	Uncharacterized protein
4030	E9PL82	2	3	14	129	14584	7.5	Uncharacterized protein
4030	E9PMR4	2	3	7.2	251	28067	7.7	Uncharacterized protein
4030	Q921J7	2	3	7.1	253	28241	7.3	Cd151 protein
4030	P48509	2	3	7.1	253	28295	7.5	CD151 antigen
4030	O35566	2	3	7.1	253	28246	7.5	CD151 antigen
4031	P49006	2	14	14.4	195	19529	4.7	MARCKS-related protein
4031	P28667	2	14	14	200	20165	4.6	MARCKS-related protein
4032	E3UN45	2	3	15.2	165	18219	6.5	Insulin-like growth factor II transcript variant 3 isoform 2 (Fragment)
4032	A8K6C9	2	3	13.9	180	20172	9.3	cDNA FLJ78037, highly similar to sapiens insulin-like growth factor 2 (somatomedin A), mRNA
4032	P09535	2	3	13.9	180	20030	9.1	Insulin-like growth factor II
4032	P01344	2	3	13.9	180	20140	9.3	Insulin-like growth factor II
4032	P01344-2	2	3	13.7	183	20477	9.5	Isoform 2 of Insulin-like growth factor II
4032	C9JAF2	2	3	10.6	236	26331	9.1	Uncharacterized protein
4033	O94817	2	3	13.6	140	15113	5.1	Ubiquitin-like protein ATG12
4033	Q9CQY1	2	3	13.5	141	15207	5	Ubiquitin-like protein ATG12
4034	Q3TDG7	2	2	21	138	15576	5.6	Putative uncharacterized protein
4034	E9PXG6	2	2	21	138	15635	5.4	Uncharacterized protein
4034	B7ZBD5	2	2	17.6	165	18477	4.6	RAB, member of RAS oncogene family-like 2A
4034	A8MXF6	2	2	17.6	165	18477	4.6	Uncharacterized protein
4034	Q8K2P9	2	2	13	223	25553	5.6	Rabl2a protein
4034	E9Q9D5	2	2	13	223	25610	5.6	Uncharacterized protein
4034	A0AUY0	2	2	12.9	224	25737	4.8	RABL2A protein
4034	Q9UNT1-2	2	2	12.7	229	26172	4.9	Isoform 2 of Rab-like protein 2B
4034	Q9UNT1	2	2	12.7	228	26101	4.9	Rab-like protein 2B
4034	Q9UBK7	2	2	12.7	228	26115	4.9	Rab-like protein 2A
4034	Q6IC14	2	2	12.7	229	26186	4.9	RABL2A protein
4034	E9PAN8	2	2	12.2	238	27186	4.9	Uncharacterized protein

Table S1. Proteins identified in the OVCAR-3 tumor supernatant by two or more unique peptides.

Protein or Protein Group	Accession Number	Unique Peptide Count	Spectrum Count	Sequence Coverage (%)	Length	MolWt	pI	Descriptive Name
4034	B7ZBD6	2	2	12.2	238	27201	4.9	RAB, member of RAS oncogene family-like 2A
4034	Q9D133	2	2	10.2	285	31677	6.3	Putative uncharacterized protein
4034	E9Q9T7	2	2	10.2	285	31676	6.6	Uncharacterized protein
4035	UPI0000D61354	2	2	12.8	187	20711	5.6	Ornithine decarboxylase antizyme 2 (ODC-Az 2) (AZ2).
4035	O08608	2	2	12.7	189	21025	5.6	Ornithine decarboxylase antizyme 2
4035	Q9HD73	2	2	12.7	189	20999	5.6	Ornithine decarboxylase antizyme
4035	O95190	2	2	12.7	189	21011	5.4	Ornithine decarboxylase antizyme 2
4036	B2R8A2	2	3	12.6	214	25078	7.2	cDNA, FLJ93804, highly similar to sapiens gp25L2 protein (HSGP25L2G), mRNA
4036	Q9BVK6	2	3	11.5	235	27277	8	Transmembrane emp24 domain-containing protein 9
4036	Q99KF1	2	3	11.5	235	27127	8.4	Transmembrane emp24 domain-containing protein 9
4036	Q3U7N8	2	3	11.5	235	27142	8	Putative uncharacterized protein
4036	Q9CZL0	2	3	10	269	30880	9.4	Putative uncharacterized protein
4036	Q3TFU8	2	3	10	269	31259	9.6	Putative uncharacterized protein
4036	E9PWI2	2	3	10	269	30742	9.1	Uncharacterized protein
4037	P62273	2	2	14.3	56	6677	10.1	40S ribosomal protein S29 40S ribosomal protein S29
4037	A8MZ73	2	2	11.9	67	8087	10	Ribosomal protein S29
4038	B7Z4T5	2	3	20	220	24073	5.9	cDNA FLJ52704, highly similar to Homeobox protein Meis1
4038	Q53RD5	2	3	17.8	247	26996	5.9	Putative uncharacterized protein MEIS1 (Fragment)
4038	Q60954-3	2	3	11.9	370	40622	7	Isoform 3 of Homeobox protein Meis1
4038	B2RC57	2	3	11.3	390	42958	6.4	cDNA, FLJ95861, highly similar to sapiens Meis1, myeloid ecotropic viral integration site 1log () (MEIS1), mRNA
4038	Q60954	2	3	11.3	390	43002	6.3	Homeobox protein Meis1
4038	O00470	2	3	11.3	390	43016	6.3	Homeobox protein Meis1
4038	Q60954-2	2	3	9.5	465	50764	6.6	Isoform 2 of Homeobox protein Meis1
4038	O00470-2	2	3	9.5	463	50576	6.5	Isoform 2 of Homeobox protein Meis1
4038	E9PGN2	2	3	9.5	465	50803	6.6	Uncharacterized protein
4039	P62861	2	16	18.6	59	6648	12.1	40S ribosomal protein S30 40S ribosomal protein S30
4039	E9PR30	2	16	11.2	98	10905	11.6	Uncharacterized protein
4039	Q3THB2	2	16	8.3	133	14420	10.1	Putative uncharacterized protein
4039	Q920W8	2	16	8	137	14845	9.9	Fau protein (Fragment)
4039	Q91V99	2	16	8	137	14787	10.1	Fau protein (Fragment)
4040	Q5PR73	2	3	11.1	199	22498	8.8	GTP-binding protein Di-Ras2
4040	Q96HU8	2	3	11.1	199	22485	8.8	GTP-binding protein Di-Ras2
4041	Q15773	2	2	10.9	248	28147	6.9	Myeloid leukemia factor 2
4041	Q99KX1	2	2	10.9	247	28055	7	Myeloid leukemia factor 2
4041	Q3URS8	2	2	10.9	247	28154	7.3	Putative uncharacterized protein
4042	B1AWT7	2	5	17.8	157	16784	6	Ubiquitin-conjugating enzyme E2 J1 (Fragment)
4042	A3KG52	2	5	10.7	261	28809	7.3	Ubiquitin-conjugating enzyme E2 J1 (Fragment)
4042	B4DUF8	2	5	9.2	303	33311	6.2	cDNA FLJ52165, highly similar to Ubiquitin-conjugating enzyme E2 J1 (EC 6.3.2.19)
4042	Q9Y385	2	5	8.8	318	35199	6.8	Ubiquitin-conjugating enzyme E2 J1
4042	Q9JJZ4	2	5	8.8	318	34990	7	Ubiquitin-conjugating enzyme E2 J1
4042	Q53F25	2	5	8.8	318	35185	6.8	Ubiquitin-conjugating enzyme E2, J1 (UBC6 log, yeast), isoform CRA_a (Fragment)
4042	Q3UDP8	2	5	8.8	318	35018	7.2	Putative uncharacterized protein
4042	Q3TV38	2	5	8.8	318	34976	7	Putative uncharacterized protein
4043	E9QPC5	2	2	10.6	350	40709	5.6	Uncharacterized protein
4043	Q3UIK4-2	2	2	10.6	350	40721	5.6	Isoform 2 of Methyltransferase-like protein 14
4043	Q9HCE5	2	2	8.1	456	52150	6.2	Methyltransferase-like protein 14
4043	Q3UIK4	2	2	8.1	456	52122	6.2	Methyltransferase-like protein 14
4044	B7Z9Q2	2	2	12.5	320	35895	5.4	cDNA, FLJ78916, highly similar to Mothers against decapentaplegic log 3 (SMAD3) cDNA, FLJ78916, highly similar to Mothers against decapentaplegic log 3 (SMAD3)
4044	B7Z4Z5	2	2	10.5	381	43237	7	cDNA FLJ54532, highly similar to Mothers against decapentaplegic log 3 (SMAD3)
4044	Q59F45	2	2	10.4	386	43659	6.8	MAD, mothers against decapentaplegic log 3 variant (Fragment)
4044	P84022	2	2	9.4	425	48081	7.2	Mothers against decapentaplegic log 3 Mothers against decapentaplegic log 3
4044	B7Z5N5	2	2	9.3	431	48669	7.1	cDNA FLJ50177, highly similar to Mothers against decapentaplegic log 2(SMAD 2)
4044	Q15796-2	2	2	9.2	437	48956	7.1	Isoform Short of Mothers against decapentaplegic log 2 Isoform Short of Mothers against decapentaplegic log 2
4044	E9Q3M0	2	2	9.2	434	48683	6.8	Uncharacterized protein
4044	Q62432	2	2	8.6	467	52266	6.6	Mothers against decapentaplegic log 2
4044	Q15796	2	2	8.6	467	52306	6.6	Mothers against decapentaplegic log 2
4045	B4DS19	2	2	10.3	368	40922	4.8	cDNA FLJ50634, highly similar to Peroxisomal targeting signal 1 receptor
4045	P50542-2	2	2	6.3	602	66830	4.5	Isoform 2 of Peroxisomal targeting signal 1 receptor
4045	O09012-2	2	2	6.3	602	66806	4.6	Isoform 2 of Peroxisomal targeting signal 1 receptor
4045	P50542-3	2	2	6	631	69872	4.6	Isoform 3 of Peroxisomal targeting signal 1 receptor
4045	D3Z600	2	2	6	632	69836	4.5	Uncharacterized protein
4045	P50542	2	2	5.9	639	70865	4.5	Peroxisomal targeting signal 1 receptor
4045	O09012	2	2	5.9	639	70756	4.5	Peroxisomal targeting signal 1 receptor
4045	B4DZ45	2	2	5.8	654	72291	4.5	cDNA FLJ52372, highly similar to Peroxisomal targeting signal 1 receptor
4046	C9J9G5	2	3	10.2	187	21114	5.2	RWD domain containing 4A
4046	Q9CPR1	2	3	10.1	188	21138	5.6	RWD domain-containing protein 4
4046	Q6NW29	2	3	10.1	188	21251	5.3	RWD domain-containing protein 4
4047	A6XGP7	2	3	10.1	199	22252	9.2	Nogo-C
4047	Q7L7Q5	2	3	10.1	199	22395	9.3	RTN4
4047	Q78NS1	2	3	10.1	199	22466	9.4	RTN4
4048	E7EWD7	2	4	10	230	25444	6	Uncharacterized protein
4048	Q6V963	2	4	10	230	25445	5.5	CREB
4048	UPI0000D62CF1	2	4	8.7	264	28713	5.5	cAMP responsive element binding protein 1
4048	Q8VDJ8	2	4	7	327	35096	5.4	cAMP responsive element binding protein 1
4048	Q543W0	2	4	7	327	35122	5.3	cAMP responsive element binding protein 1, isoform CRA_a
4048	Q53X93	2	4	7	327	35136	5.3	CAMP responsive element binding protein 1, isoform CRA_b (Fragment)
4048	Q01147	2	4	6.7	341	36674	5.6	Cyclic AMP-responsive element-binding protein 1
4048	P16220	2	4	6.7	341	36688	5.6	Cyclic AMP-responsive element-binding protein 1
4049	UPI0001889EB5	2	2	11.7	128	14423	4.8	NADH dehydrogenase (ubiquinone) 1, alpha/beta subcomplex, 1
4049	O14561	2	2	9.6	156	17417	4.9	Acyl carrier protein, mitochondrial
4049	UPI0000024168	2	2	9.6	156	17442	5.1	PREDICTED: acyl carrier protein, mitochondrial-like
4049	Q9CR21	2	2	9.6	156	17370	5.2	Acyl carrier protein, mitochondrial
4049	Q3TJG2	2	2	9.6	156	17386	5.2	Acyl carrier protein
4050	A2A9E9	2	2	9.4	310	34540	5.1	GPN-loop GTPase 2
4050	Q9H9Y4	2	2	9.4	310	34561	5.2	GPN-loop GTPase 2
4050	Q8VEJ1	2	2	9.4	310	34556	5.1	GPN-loop GTPase 2
4051	UPI0001D8D93F	2	3	10.5	612	66784	6.8	bifunctional UDP-N-acetylglucosamine 2-epimerase/N-acetylmannosamine kinase isoform 5
4051	B7Z372	2	3	10.5	612	66756	6.9	cDNA FLJ51479, highly similar to Bifunctional UDP-N-acetylglucosamine2-epimerase/N- acetylmannosamine kinase

Table S1. Proteins identified in the OVCAR-3 tumor supernatant by two or more unique peptides.

Protein or Protein Group	Accession Number	Unique Peptide Count	Spectrum Count	Sequence Coverage (%)	Length	MolWt	pI	Descriptive Name
4051	A7UNU7	2	3	9.9	648	71278	6.8	Mutant UDP-N-acetylglucosamine 2-epimerase/N-acetylmannosamine kinase
4051	A6PZH3	2	3	9.4	681	74645	6.9	UDP-N-acetylglucosamine 2-epimerase/N-acetylmannosamine kinase
4051	B7Z428	2	3	9.4	681	74702	6.9	cDNA FLJ52319, highly similar to Bifunctional UDP-N-acetylglucosamine 2-epimerase/N-acetylmannosamine kinase
4051	Q9Y223	2	3	8.9	722	79275	6.8	Bifunctional UDP-N-acetylglucosamine 2-epimerase/N-acetylmannosamine kinase
4051	Q91WG8	2	3	8.9	722	79199	6.8	Bifunctional UDP-N-acetylglucosamine 2-epimerase/N-acetylmannosamine kinase
4051	Q6QNY7	2	3	8.9	722	79305	6.7	UDP-N-acetylglucosamine-2-epimerase / N-acetylmannosamine kinase
4051	Q6QNY6	2	3	8.9	722	79232	6.7	UDP-N-acetylglucosamine-2-epimerase / N-acetylmannosamine kinase
4051	Q6QNY5	2	3	8.9	722	79374	6.9	UDP-N-acetylglucosamine-2-epimerase / N-acetylmannosamine kinase
4052	B3KUJ0	2	20	9.4	424	44484	8.2	cDNA FLJ39996 fis, clone STOMA2002166, highly similar to Splicing factor 3B subunit 4
4052	Q8QZY9	2	20	9.4	424	44356	8.6	Splicing factor 3B subunit 4
4052	Q53FG6	2	20	9.4	424	44387	8.2	Splicing factor 3b, subunit 4 variant (Fragment)
4052	Q15427	2	20	9.4	424	44386	8.6	Splicing factor 3B subunit 4
4053	Q8IV50	2	2	9.3	215	23463	5.4	LysM and putative peptidoglycan-binding domain-containing protein 2
4053	UPI00000239D2	2	2	9.3	215	23594	5.5	lysM and putative peptidoglycan-binding domain-containing protein 2
4053	Q9D7V2	2	2	9.3	215	23694	5.7	LysM and putative peptidoglycan-binding domain-containing protein 2
4054	Q13432-2	2	3	10.9	220	24112	7.3	Isoform B of Protein unc-119 log A
4054	Q9Z2R6	2	3	10	240	27010	6.2	Protein unc-119 log A
4054	Q13432	2	3	10	240	26962	6.4	Protein unc-119 log A
4054	B1AQD9	2	3	9.2	262	29217	6.2	Unc-119 log (C. elegans)
4055	B3KRS9	2	3	9.1	132	15257	6.7	cDNA FLJ34851 fis, clone NT2NE2011998, highly similar to BTB/POZ domain-containing protein KCTD10
4055	B3KVY5	2	3	9.1	132	15315	6.4	Potassium channel tetramerisation domain containing 10, isoform CRA_a
4055	Q9H3F6-2	2	3	4.1	290	32779	6.6	Isoform 2 of BTB/POZ domain-containing adapter for CUL3-mediated RhoA degradation protein 3
4055	Q9H3F6	2	3	3.8	313	35432	6.3	BTB/POZ domain-containing adapter for CUL3-mediated RhoA degradation protein 3
4055	Q922M3	2	3	3.8	315	35701	6.3	BTB/POZ domain-containing adapter for CUL3-mediated RhoA degradation protein 3
4055	E9PU91	2	3	3.8	316	35830	6.3	Uncharacterized protein
4056	B7ZA62	2	3	12.5	271	31202	6.1	cDNA, FLJ79076, highly similar to Dual specificity mitogen-activated protein kinase kinase 4 (EC 2.7.12.2)
4056	Q8K2U0	2	3	12.1	281	32249	6.6	Map2k4 protein
4056	B7Z9Y5	2	3	12.1	281	32161	6.9	cDNA, FLJ78999, highly similar to Dual specificity mitogen-activated protein kinase kinase 4 (EC 2.7.12.2)
4056	B4DH44	2	3	9	376	42545	8.1	cDNA FLJ52538, highly similar to Dual specificity mitogen-activated proteinkinase kinase 4 (EC 2.7.12.2)
4056	P47809	2	3	8.6	397	44114	8.1	Dual specificity mitogen-activated protein kinase kinase 4
4056	P45985	2	3	8.5	399	44288	8.1	Dual specificity mitogen-activated protein kinase kinase 4
4056	P45985-2	2	3	8.3	410	45584	8	Isoform 2 of Dual specificity mitogen-activated protein kinase kinase 4
4057	P12532	2	2	8.9	417	47037	8.3	Creatine kinase U-type, mitochondrial
4057	P30275	2	2	8.9	418	47004	8.2	Creatine kinase U-type, mitochondrial
4057	P12532-2	2	2	8.3	448	50421	8.6	Isoform 2 of Creatine kinase U-type, mitochondrial
4058	B2KFR4	2	2	8.6	464	53772	7	MCG15023
4058	Q9Y2H1	2	2	8.6	464	54003	6.8	Serine/threonine-protein kinase 38-like
4058	Q7TSE6	2	2	8.6	464	53786	7	Serine/threonine-protein kinase 38-like
4058	Q7TSE6-2	2	2	8.5	471	54624	7	Isoform 2 of Serine/threonine-protein kinase 38-like
4058	B2KFR5	2	2	8.5	471	54610	7	Serine/threonine kinase 38 like
4059	Q00534	2	2	8.6	326	36938	6.5	Cyclin-dependent kinase 6
4059	Q64261	2	2	8.6	326	37028	6.6	Cyclin-dependent kinase 6
4059	Q3U4J9	2	2	8.6	326	36959	6.5	Putative uncharacterized protein
4060	A6QKW0	2	2	8.5	247	27951	9.7	SHINC3
4060	Q9CR67	2	2	8.5	247	28031	9.7	Transmembrane protein 33
4060	P57088	2	2	8.5	247	27978	9.7	Transmembrane protein 33
4061	B2KG22	2	2	8.3	229	26202	7.1	Likely orthologue of H. sapiens chromosome 12 open reading frame 11 (C12orf11) (Fragment)
4061	UPI00004565E4	2	2	6.5	292	33629	6.4	Uncharacterized protein C12orf11 (Sarcoma antigen NY-SAR-95).
4061	Q9NW22	2	2	6.5	294	33873	6.4	cDNA FLJ10360 fis, clone NT2RM2001247
4061	B4DNK1	2	2	3.1	605	68495	6.8	cDNA FLJ58152
4061	Q9NVM9	2	2	2.7	706	80225	6.7	Cell cycle regulator Mat89Bb log
4061	Q8QZV7	2	2	2.6	732	82750	6.9	Cell cycle regulator Mat89Bb log
4061	E9QLV7	2	2	2.6	732	82780	6.9	Uncharacterized protein
4062	UPI0001F78EF4	2	3	22.1	204	23242	9.4	UPI0001F78EF4 UniRef100 entry
4062	E9Q648	2	3	13.5	334	38775	6	Uncharacterized protein
4062	B1AA15	2	3	8.2	547	62210	5.8	Gastric cancer hepatocellular carcinoma suppressor 1 variant
4062	E9PYD3	2	3	8.2	547	62232	5.6	Uncharacterized protein
4062	E7EVT8	2	3	8.2	547	62167	5.7	Uncharacterized protein
4062	Q8CCB4-2	2	3	6.9	655	73703	6.6	Isoform 2 of Vacuolar protein sorting-associated protein 53 log
4062	C9JS52	2	3	6.8	666	75809	5.9	Uncharacterized protein
4062	Q5VIR6-3	2	3	6.7	673	76569	5.8	Isoform 3 of Vacuolar protein sorting-associated protein 53 log
4062	B1AA17	2	3	6.7	670	76395	6.1	Gastric cancer hepatocellular carcinoma suppressor 1 variant
4062	Q5VIR6	2	3	6.4	699	79653	6	Vacuolar protein sorting-associated protein 53 log
4062	E9PXG9	2	3	5.6	803	91122	6.6	Uncharacterized protein
4062	UPI0000DBEF01	2	3	5.4	832	94405	6.7	vacuolar protein sorting-associated protein 53 log isoform 1
4062	Q8CCB4	2	3	5.4	832	94423	6.6	Vacuolar protein sorting-associated protein 53 log
4062	Q3UDN2	2	3	5.4	832	94351	6.7	Putative uncharacterized protein
4062	Q3TX19	2	3	5.4	832	94481	6.5	Putative uncharacterized protein
4062	Q3TLZ3	2	3	5.4	832	94391	6.6	Putative uncharacterized protein
4062	Q3TJT1	2	3	5.4	832	94427	6.6	Putative uncharacterized protein
4062	B3FH42	2	3	5.4	832	94377	6.6	Vps53 long isoform
4063	Q9UGV2-2	2	4	8.8	363	39982	5.4	Isoform 2 of Protein NDRG3
4063	Q9UGV2	2	4	8.5	375	41409	5.3	Protein NDRG3
4063	Q9QYF9	2	4	8.5	375	41555	5.2	Protein NDRG3
4063	Q5TH30	2	4	8.2	388	42850	5.3	NDRG family member 3
4063	Q8VCV2	2	4	8.2	388	42983	5.3	N-myc downstream regulated gene 3
4064	Q8K479	2	2	8.2	243	25437	6.7	Complement C1q tumor necrosis factor-related protein 5
4064	Q9BXJ0	2	2	8.2	243	25298	6.5	Complement C1q tumor necrosis factor-related protein 5
4064	Q8R002	2	2	8.2	243	25421	6.7	C1q and tumor necrosis factor related protein 5
4065	Q9NQL2-2	2	5	12.9	249	28611	7.2	Isoform 2 of Ras-related GTP-binding protein D

Protein or Protein Group	Accession Number	Unique Peptide Count	Spectrum Count	Sequence Coverage (%)	Length	MolWt	pI	Descriptive Name
4065	B4DQ03	2	5	11.5	279	31695	5.3	cDNA FLJ57859, highly similar to Ras-related GTP-binding protein C
4065	B1AWT4	2	5	11.5	279	32023	7.5	Ras-related GTP binding D
4065	D3DPT8	2	5	9.6	333	37903	5.6	Ras-related GTP binding C, isoform CRA_a
4065	E7ENI3	2	5	8.8	365	40262	5.1	Uncharacterized protein
4065	B4DQG4	2	5	8.8	365	40230	5.1	cDNA FLJ55138, highly similar to Ras-related GTP-binding protein C
4065	B1AWT2	2	5	8	399	45145	4.9	Ras-related GTP binding D
4065	Q9NQL2	2	5	8	400	45588	4.9	Ras-related GTP-binding protein D
4065	Q9HB90	2	5	8	399	44224	5.1	Ras-related GTP-binding protein C
4065	Q99K70	2	5	8	398	44121	5.1	Ras-related GTP-binding protein C
4065	Q7TT45	2	5	7.1	449	51232	5.3	Ras-related GTP-binding protein D
4065	B1AWT3	2	5	7	454	51803	5.2	Ras-related GTP binding D
4066	A8K766	2	2	7.8	255	27922	8.4	cDNA FLJ77343, highly similar to sapiens electron-transfer-flavoprotein, beta polypeptide(ETFB), mRNA
4066	Q9DCW4	2	2	7.8	255	27623	8.1	Electron transfer flavoprotein subunit beta
4066	P38117	2	2	7.8	255	27844	8.1	Electron transfer flavoprotein subunit beta
4066	P38117-2	2	2	5.8	346	37434	7.2	Isoform 2 of Electron transfer flavoprotein subunit beta
4067	Q3V1W5	2	7	7.9	394	44685	6	Putative uncharacterized protein
4067	B4DP78	2	7	7.8	396	45053	6.3	cDNA FLJ52341, highly similar to sapiens sarcoglycan, epsilon (SGCE), mRNA
4067	E9PEH6	2	7	7.8	396	45069	6.1	Uncharacterized protein
4067	Q4H438	2	7	7.4	419	47282	6.5	Epsilon-Sarcoglycan splicing variant 3A
4067	Q6L8N9	2	7	7.3	427	48436	6.1	Brain epsilon-sarcoglycan
4067	D3Z572	2	7	7.2	428	48635	6.5	Uncharacterized protein
4067	C9JR67	2	7	7.2	428	48751	6.5	Uncharacterized protein
4067	O70258	2	7	7.1	437	49736	6.4	Epsilon-sarcoglycan
4067	O43556	2	7	7.1	437	49851	6.5	Epsilon-sarcoglycan
4067	E9PXU0	2	7	7.1	434	49139	6.3	Uncharacterized protein
4067	E9PX46	2	7	7	443	50241	6.1	Uncharacterized protein
4067	E9PF60	2	7	6.9	451	51414	6.2	Uncharacterized protein
4067	UPI0000D4E987	2	7	6.7	462	52525	6.5	epsilon-sarcoglycan isoform 1
4067	B5MDA7	2	7	6.7	462	52646	6.5	Uncharacterized protein
4067	O70258-2	2	7	6.6	473	53532	7	Isoform 2 of Epsilon-sarcoglycan
4067	B7Z2R4	2	7	6.6	471	53434	6.7	cDNA FLJ50763, highly similar to sapiens sarcoglycan, epsilon (SGCE), mRNA
4068	B4DSY4	2	2	7.6	331	35787	6.2	cDNA FLJ52894, highly similar to Isocitrate dehydrogenase
4068	E7EWD2	2	2	7.6	331	35786	6.4	Uncharacterized protein
4068	Q9D6R2	2	2	6.8	366	39639	6.7	Isocitrate dehydrogenase [NAD] subunit alpha, mitochondrial
4068	Q53GF8	2	2	6.8	366	39620	6.9	Isocitrate dehydrogenase 3 (NAD+) alpha variant (Fragment)
4068	P50213	2	2	6.8	366	39592	6.9	Isocitrate dehydrogenase [NAD] subunit alpha, mitochondrial
4069	B4E2M1	2	3	7.5	428	48557	6.8	cDNA FLJ58478, highly similar to Glypican-6
4069	Q9Y625	2	3	5.8	555	62736	5.4	Glypican-6
4069	Q9R087	2	3	5.8	555	63057	5.4	Glypican-6
4069	Q3TP69	2	3	5.8	555	62985	5.5	Putative uncharacterized protein
4069	Q8R3X6	2	3	5.7	565	64340	5.7	Glypican 6
4070	B7Z7L2	2	3	7.5	429	50244	5.8	cDNA FLJ51383, highly similar to Serine/threonine-protein phosphatase 2A 56 kDa regulatory subunit alpha isoform
4070	Q6PD03	2	3	6.6	486	56347	7	Serine/threonine-protein phosphatase 2A 56 kDa regulatory subunit alpha isoform
4070	Q15172	2	3	6.6	486	56194	6.7	Serine/threonine-protein phosphatase 2A 56 kDa regulatory subunit alpha isoform
4070	UPI00015DF6A4	2	3	6.5	490	56810	7.1	protein phosphatase 2, regulatory subunit B (B56), alpha isoform
4070	Q3ULN8	2	3	6.5	489	56770	6.9	Putative uncharacterized protein (Fragment)
4071	C9JM82	2	3	9	301	34673	6.2	Uncharacterized protein
4071	E7ENS5	2	3	7.4	366	42406	6.7	Uncharacterized protein
4071	Q9Z2Q6	2	3	7.3	369	42748	6.7	Septin-5
4071	Q99719	2	3	7.3	369	42777	6.7	Septin-5
4071	B7ZNM7	2	3	7.1	381	44081	6.8	Sept5 protein
4071	Q7KYV2	2	3	7	385	44158	6.5	H5 (Fragment)
4071	Q59GE1	2	3	6.9	394	45220	6.7	H5 variant (Fragment)
4071	Q99648	2	3	6.5	417	47622	7.4	Septin (Fragment)
4072	O95571	2	2	7.1	254	27873	6.8	Protein ETHE1, mitochondrial
4072	Q9DCM0	2	2	7.1	254	27739	7.2	Protein ETHE1, mitochondrial
4073	UPI0001CB7927	2	25	10.7	224	25726	8.9	nebulette isoform 3
4073	Q9DC07	2	25	8.9	270	31113	8.3	Nebulette
4073	Q70I54	2	25	8.9	270	31196	8.3	LIM-nebulette
4073	Q59FZ8	2	25	7	345	38697	9	Nebulette non-muscle isoform variant (Fragment)
4074	E5RG71	2	4	23.5	85	9212	9.9	Uncharacterized protein
4074	E9Q786	2	4	22.5	89	9653	10	Uncharacterized protein
4074	Q8BJ85	2	4	13.9	144	15859	9.7	Putative uncharacterized protein
4074	B3KT61	2	4	7.1	280	31074	7.9	cDNA FLJ37707 fis, clone BRHIP2017642, moderately similar to RNA-binding protein Raly
4074	B3KSX3	2	4	6.9	291	32347	7.9	cDNA FLJ37224 fis, clone BRAMY2000052, moderately similar to RNA-binding protein Raly
4074	Q86SE5	2	4	6.9	291	32331	7.9	RNA-binding Raly-like protein
4074	Q8BTF8	2	4	6.8	293	32452	7.9	RNA-binding Raly-like protein
4075	B7Z970	2	7	8	552	62001	6.7	cDNA FLJ52404, highly similar to sapiens related to CPSF subunits 74 kDa (RC74), mRNA
4075	B7Z6M5	2	7	6.9	637	71468	6.6	cDNA FLJ53924, highly similar to sapiens related to CPSF subunits 74 kDa (RC74), mRNA
4075	Q9NV88	2	7	6.7	658	73815	6.3	Integrator complex subunit 9
4075	Q8K114	2	7	6.7	658	74078	6.4	Integrator complex subunit 9
4076	Q8IUR7-6	2	3	11.9	385	42984	7.2	Isoform 6 of Armadillo repeat-containing protein 8
4076	UPI00006E0467	2	3	11.5	399	44499	7.2	UPI00006E0467 UniRef100 entry
4076	Q9DBR3-2	2	3	11.5	399	44456	7.2	Isoform 2 of Armadillo repeat-containing protein 8
4076	Q8IUR7-2	2	3	7	659	73994	6.7	Isoform 2 of Armadillo repeat-containing protein 8
4076	A8MTG8	2	3	6.8	672	75471	6.9	Uncharacterized protein
4076	UPI0000D63695	2	3	6.8	673	75402	6.7	armadillo repeat-containing protein 8 isoform 1
4076	Q9DBR3	2	3	6.8	673	75364	6.7	Armadillo repeat-containing protein 8
4076	Q8IUR7	2	3	6.8	673	75509	6.7	Armadillo repeat-containing protein 8
4077	B3KRK2	2	2	6.7	496	55410	8.2	DEAD (Asp-Glu-Ala-Asp) box polypeptide 41, isoform CRA_a
4077	Q9UJV9	2	2	5.3	622	69838	6.8	Probable ATP-dependent RNA helicase DDX41
4077	Q53HI2	2	2	5.3	622	69839	6.8	DEAD-box protein abstrakt variant (Fragment)
4077	Q3UAC4	2	2	5.3	622	69765	6.6	Putative uncharacterized protein
4077	Q3U0E0	2	2	5.3	622	69820	6.7	Putative uncharacterized protein
4077	UPI0001D3BB5A	2	2	5.2	640	71649	7.5	Probable ATP-dependent RNA helicase DDX41 (EC 3.6.1.-) (DEAD box protein 41) (DEAD box protein abstrakt log).
4078	B4E217	2	2	6.7	421	45410	5	cDNA FLJ50950, highly similar to BAG family molecular chaperone regulator 4
4078	Q8CI61	2	2	6.1	457	49095	5.3	BAG family molecular chaperone regulator 4
4078	O95429	2	2	6.1	457	49594	5.1	BAG family molecular chaperone regulator 4
4079	Q8BSR7	2	2	9.6	280	32522	6.9	Putative uncharacterized protein (Fragment)

Table S1. Proteins identified in the OVCAR-3 tumor supernatant by two or more unique peptides.

Protein or Protein Group	Accession Number	Unique Peptide Count	Spectrum Count	Sequence Coverage (%)	Length	MolWt	pI	Descriptive Name
4079	O08586	2	2	6.7	403	47152	6.4	Phosphatidylinositol-3,4,5-trisphosphate 3-phosphatase and dual-specificity protein phosphatase PTEN
4079	P60484	2	2	6.7	403	47166	6.4	Phosphatidylinositol-3,4,5-trisphosphate 3-phosphatase and dual-specificity protein phosphatase PTEN
4079	O43460	2	2	6.7	403	47163	6.4	Phosphatase and tensin log 2 (Fragment)
4079	Q3UUN5	2	2	6.6	407	46893	6.8	Putative uncharacterized protein
4080	B4DE48	2	2	6.6	440	49755	8	cDNA FLJ53824, highly similar to Sorting nexin-17
4080	B4DQ37	2	2	6.4	450	50836	8	cDNA FLJ56223, highly similar to Sorting nexin-17
4080	Q8BVL3	2	2	6.2	470	52797	7.5	Sorting nexin-17
4080	Q15036	2	2	6.2	470	52901	7.5	Sorting nexin-17
4081	Q9UID7	2	3	6.6	334	37247	8.1	CYR61 protein
4081	B4DI61	2	3	6.2	357	39680	8.3	cDNA FLJ58182, highly similar to Protein CYR61
4081	E9PCE2	2	3	6.1	358	39783	8.3	Uncharacterized protein
4081	Q53FA4	2	3	5.8	381	41995	8.3	Cysteine-rich, angiogenic inducer, 61 variant (Fragment)
4081	P18406	2	3	5.8	379	41709	8.1	Protein CYR61
4081	O00622	2	3	5.8	381	42027	8.2	Protein CYR61
4082	B7Z3D0	2	3	7.4	352	40870	6.5	cDNA FLJ60571, highly similar to Origin recognition complex subunit 4
4082	B7Z2M4	2	3	7.3	357	41005	7.6	cDNA FLJ60061, highly similar to Origin recognition complex subunit 4
4082	A8K7H4	2	3	6	436	50393	8	cDNA FLJ75764, highly similar to sapiens origin recognition complex, subunit 4-like (yeast) (ORC4L), transcript variant 2, mRNA
4082	O88708	2	3	6	433	49982	7.3	Origin recognition complex subunit 4
4082	O43929	2	3	6	436	50377	8	Origin recognition complex subunit 4
4083	E7EP30	2	6	6	695	78983	5.2	Uncharacterized protein
4083	Q8N8L9	2	6	6	695	78969	5.2	cDNA FLJ39238 fis, clone OCBBF2007946
4083	UPI000059D3B1	2	6	5.5	768	87184	5.2	conserved oligomeric Golgi complex subunit 4 isoform 2
4083	Q6PIW8	2	6	5.5	763	86647	5.2	COG4 protein (Fragment)
4083	Q9H9E3	2	6	5.4	785	89083	5.2	Conserved oligomeric Golgi complex subunit 4
4083	Q8R1U1	2	6	5.4	785	88661	5.1	Conserved oligomeric Golgi complex subunit 4
4084	Q9H999	2	2	7	370	41094	6.6	Pantothenate kinase 3
4084	Q8R2W9	2	2	7	370	41120	6.6	Pantothenate kinase 3
4084	Q7M752	2	2	7	370	41106	6.6	Pantothenate kinase 3
4084	Q3UWJ1	2	2	6	430	48239	7.4	Putative uncharacterized protein
4085	Q99LE7	2	2	9.3	378	41782	7.5	Pxn protein (Fragment)
4085	Q6ZQU7	2	2	8.7	403	44524	7.3	cDNA FLJ46879 fis, clone UTERU3015011, highly similar to Paxillin
4085	E7EMK8	2	2	8.7	403	44524	7.5	Uncharacterized protein
4085	P49023-4	2	2	8.3	424	46604	7.1	Isoform 4 of Paxillin
4085	Q8VI36-2	2	2	6.3	557	60812	6.3	Isoform Alpha of Paxillin
4085	Q3UG90	2	2	6.3	557	60906	6.3	Putative uncharacterized protein
4085	P49023-2	2	2	6.3	557	60967	6.4	Isoform Alpha of Paxillin
4085	B3KVL0	2	2	5.9	589	64159	6.4	cDNA FLJ16691 fis, clone TRACH3000692, highly similar to Paxillin
4085	UPI0000D77AD6	2	2	5.9	591	64506	6	paxillin isoform beta
4085	Q8VI36	2	2	5.9	591	64476	6	Paxillin
4085	P49023	2	2	5.9	591	64505	6.2	Paxillin
4085	B7ZMB4	2	2	5.9	591	64475	6.2	Paxillin
4085	P49023-3	2	2	5.8	605	66213	6.4	Isoform Gamma of Paxillin
4086	E9PF31	2	3	6.9	422	46137	6.3	Uncharacterized protein
4086	O94788-2	2	3	6	480	53060	6.1	Isoform 2 of Retinal dehydrogenase 2
4086	B4DH89	2	3	5.9	489	53791	6.1	cDNA FLJ55560, highly similar to Retinal dehydrogenase 2 (EC 1.2.1.36)
4086	B4DZR2	2	3	5.8	497	54639	6.6	cDNA FLJ59801, highly similar to Retinal dehydrogenase 2 (EC 1.2.1.36)
4086	Q62148	2	3	5.6	518	56626	5.7	Retinal dehydrogenase 2
4086	O94788	2	3	5.6	518	56724	6	Retinal dehydrogenase 2
4087	C9JJA4	2	3	5.9	407	46043	5.8	Uncharacterized protein
4087	Q8TAF3-4	2	3	4	595	66707	6.5	Isoform 4 of WD repeat-containing protein 48
4087	Q8TAF3-3	2	3	3.6	668	75349	7.1	Isoform 3 of WD repeat-containing protein 48
4087	Q8BH57-2	2	3	3.6	662	74401	7.2	Isoform 2 of WD repeat-containing protein 48
4087	Q8BH57	2	3	3.6	676	76007	7.2	WD repeat-containing protein 48
4087	Q8TAF3	2	3	3.5	677	76211	7	WD repeat-containing protein 48
4088	P55081	2	2	5.9	439	51958	5	Microfibrillar-associated protein 1
4088	Q9CQU1	2	2	5.9	439	51954	5	Microfibrillar-associated protein 1
4089	D3YU58	2	4	6.6	503	56168	8.5	Uncharacterized protein
4089	B3KMP2	2	4	6.6	503	56314	8.5	cDNA FLJ11747 fis, clone HEMBA1005530, highly similar to Anaphase-promoting complex subunit 7
4089	UPI0001B790E7	2	4	6.2	530	59345	8.6	UPI0001B790E7 UniRef100 entry
4089	Q9UJX3-2	2	4	6.1	537	60002	8.4	Isoform 2 of Anaphase-promoting complex subunit 7
4089	A5D8X2	2	4	5.8	565	63115	5.7	Anaphase promoting complex subunit 7
4089	Q9WVM3	2	4	5.8	565	63021	5.7	Anaphase-promoting complex subunit 7
4089	Q9UJX3	2	4	5.5	599	66856	5.6	Anaphase-promoting complex subunit 7
4090	B4DTT5	2	4	6.6	273	31228	5.6	cDNA FLJ50214, highly similar to Follistatin-related protein 1
4090	Q6GTX2	2	4	5.9	306	34554	5.8	Follistatin-like 1
4090	Q62356	2	4	5.9	306	34538	6	Follistatin-related protein 1
4090	A8K523	2	4	5.8	308	34909	5.5	cDNA FLJ78447, highly similar to sapiens follistatin-like 1 (FSTL1), mRNA
4090	Q12841	2	4	5.8	308	34985	5.5	Follistatin-related protein 1
4091	Q96B13	2	7	6.8	384	44878	6.5	Protein phosphatase 2, regulatory subunit B', gamma isoform
4091	B4DYJ8	2	7	5.8	449	52650	6.7	cDNA FLJ57234, highly similar to Serine/threonine-protein phosphatase 2A 56 kDa regulatory subunit gamma isoform
4091	Q60996-2	2	7	5.8	452	52818	7	Isoform 1 of Serine/threonine-protein phosphatase 2A 56 kDa regulatory subunit gamma isoform
4091	Q13362-2	2	7	5.8	449	52625	7.2	Isoform Gamma-1 of Serine/threonine-protein phosphatase 2A 56 kDa regulatory subunit gamma isoform
4091	Q6ZQK4	2	7	5.7	458	53017	9	MKIAA0044 protein (Fragment)
4091	Q96L52	2	7	5.5	475	55556	6.8	PP2A B56 gamma 2
4091	Q60996-3	2	7	5.4	485	56552	7	Isoform 3 of Serine/threonine-protein phosphatase 2A 56 kDa regulatory subunit gamma isoform
4091	Q13362-3	2	7	5.4	485	56661	7.2	Isoform Gamma-2 of Serine/threonine-protein phosphatase 2A 56 kDa regulatory subunit gamma isoform
4091	Q60996-4	2	7	5.2	496	57517	6.8	Isoform 4 of Serine/threonine-protein phosphatase 2A 56 kDa regulatory subunit gamma isoform
4091	UPI00001E77E5	2	7	5.1	509	59365	8.7	serine/threonine-protein phosphatase 2A 56 kDa regulatory subunit gamma isoform isoform d
4091	Q7L7W2	2	7	5.1	514	59956	6.6	PP2A B56 gamma 3
4091	Q60996	2	7	5	524	60825	6.6	Serine/threonine-protein phosphatase 2A 56 kDa regulatory subunit gamma isoform
4091	Q13362	2	7	5	524	61061	6.9	Serine/threonine-protein phosphatase 2A 56 kDa regulatory subunit gamma isoform

Table S1. Proteins identified in the OVCAR-3 tumor supernatant by two or more unique peptides.

Protein or Protein Group	Accession Number	Unique Peptide Count	Spectrum Count	Sequence Coverage (%)	Length	MolWt	pI	Descriptive Name
4091	Q6ZN33	2	7	4.8	540	62755	8.7	cDNA FLJ16473 fis, clone BRHIP3026052, highly similar to Serine/threonine protein phosphatase 2A, 56 kDa regulatory subunit, gamma isoform
4091	UPI0001A7AE62	2	7	4.7	555	64292	6.7	serine/threonine-protein phosphatase 2A 56 kDa regulatory subunit gamma isoform isoform e
4092	E7EV65	2	4	6.5	307	34407	9.1	Uncharacterized protein
4092	A4FUP2	2	4	5.6	357	40000	8.3	TEAD1 protein
4092	E9QAK6	2	4	5.6	357	40002	8.3	Uncharacterized protein
4092	UPI0001F784CD	2	4	5.4	368	41140	7.7	UPI0001F784CD UniRef100 entry
4092	Q59EF3	2	4	5.4	371	41580	7.8	TEA domain family member 1 variant (Fragment)
4092	E9Q387	2	4	5.1	390	43896	7.8	Uncharacterized protein
4092	E9PVS2	2	4	5.1	394	44315	7.8	Uncharacterized protein
4092	Q3UFP5	2	4	4.8	415	46789	8.6	Putative uncharacterized protein
4092	E9Q681	2	4	4.8	415	46791	8.5	Uncharacterized protein
4092	E9PZ73	2	4	4.8	415	46671	8.4	Uncharacterized protein
4092	UPI000013D412	2	4	4.7	426	47928	8.1	UPI000013D412 UniRef100 entry
4092	P30051	2	4	4.7	426	47948	8.1	Transcriptional enhancer factor TEF-1
4092	P28347	2	4	4.7	426	47946	8.1	Transcriptional enhancer factor TEF-1
4092	Q3USK5	2	4	4.6	436	49146	9	Putative uncharacterized protein
4092	E9Q0H2	2	4	4.6	436	49148	8.9	Uncharacterized protein
4093	Q9UBB9-2	2	4	14.8	196	22431	6.5	Isoform 2 of Tuftelin-interacting protein 11
4093	E9PHU6	2	4	6.4	455	52765	5.3	Uncharacterized protein
4093	B4DSX8	2	4	6.4	454	52631	5.3	cDNA FLJ57193, highly similar to Tuftelin-interacting protein 11
4093	B4DPE5	2	4	5.6	522	61273	5.2	cDNA FLJ58206, highly similar to Tuftelin-interacting protein 11
4093	E7EMD1	2	4	5.6	522	61287	5.2	Uncharacterized protein
4093	Q9UBB9	2	4	3.5	837	96820	5.7	Tuftelin-interacting protein 11
4093	Q9ERA6	2	4	3.5	838	96305	5.9	Tuftelin-interacting protein 11
4093	Q8N523	2	4	3.5	837	96794	5.7	Tuftelin interacting protein 11
4093	Q3UGE9	2	4	3.5	838	96335	6	Putative uncharacterized protein
4094	Q9D1P2-2	2	5	12.9	194	21398	7.6	Isoform 2 of Probable histone acetyltransferase MYST1
4094	Q3V268	2	5	5.5	458	52604	8.4	Putative uncharacterized protein
4094	Q9H7Z6	2	5	5.5	458	52403	8.3	Probable histone acetyltransferase MYST1
4094	Q9D1P2	2	5	5.5	458	52574	8.4	Probable histone acetyltransferase MYST1
4094	UPI00001B02B6	2	5	5.4	467	53085	8	probable histone acetyltransferase MYST1 isoform 2
4094	Q9H7Z6-2	2	5	5.4	467	53103	8	Isoform 2 of Probable histone acetyltransferase MYST1
4095	Q6ZMK1-2	2	2	8	274	31760	7.7	Isoform 2 of Cysteine and histidine-rich protein 1
4095	Q9QXA1	2	2	7.1	311	35757	7.7	Cysteine and histidine-rich protein 1
4095	Q6ZMK1	2	2	6.1	362	40703	7.2	Cysteine and histidine-rich protein 1
4095	E9PJD7	2	2	5.4	404	43813	6.8	Uncharacterized protein
4096	D3Z1E6	2	2	18.6	145	16405	9	Uncharacterized protein
4096	E9Q162	2	2	7.1	381	43434	8.5	Uncharacterized protein
4096	A0AV88	2	2	5.3	512	57897	7.2	ADAM10 protein
4096	E9PYF2	2	2	5.3	513	57792	7.4	Uncharacterized protein
4096	A8MY20	2	2	4.8	567	63152	7.9	ADAM metallopeptidase domain 10, isoform CRA_a
4096	UPI00015DFCDA	2	2	3.6	747	84075	7.8	ADAM 10 precursor (EC 3.4.24.81) (A disintegrin and metalloproteinase domain 10) (Mammalian disintegrin-metalloprotease) (Kuzbanian protein log) (CD156c antigen) (CDw156).
4096	O35598	2	2	3.6	749	83967	8	Disintegrin and metalloproteinase domain-containing protein 10
4096	O14672	2	2	3.6	748	84142	7.8	Disintegrin and metalloproteinase domain-containing protein 10
4096	B8JJJ0	2	2	3.6	749	83968	7.9	A disintegrin and metallopeptidase domain 10
4097	B4DPV9	2	5	5.7	824	94399	6.7	cDNA FLJ55742, highly similar to Phosphatidylinositol 3-kinase catalyticsubunit type 3 (EC 2.7.1.137)
4097	A8MYT4	2	5	5.3	888	101713	6.8	Uncharacterized protein
4097	Q8NEB9	2	5	5.3	887	101549	6.8	Phosphatidylinositol 3-kinase catalytic subunit type 3
4097	Q6PF93	2	5	5.3	887	101487	6.7	Phosphatidylinositol 3-kinase catalytic subunit type 3
4098	Q96HS5	2	3	11.7	179	21021	7.9	CYTH3 protein
4098	B7Z2V9	2	3	6.7	314	36793	8.6	cDNA FLJ56345, highly similar to Cytohesin-3
4098	B2R9C3	2	3	5.3	399	46288	5.5	cDNA, FLJ94328, highly similar to sapiens pleckstrin logy, Sec7 and coiled-coil domains 3 (PSCD3), mRNA
4098	Q3TGW1	2	3	5.3	399	46181	5.5	Putative uncharacterized protein
4098	O43739-2	2	3	5.3	399	46292	5.5	Isoform 2 of Cytohesin-3
4098	O08967	2	3	5.3	399	46280	5.5	Cytohesin-3
4098	P97696	2	3	5.2	400	46337	5.5	Cytohesin-3
4098	O43739	2	3	5.2	400	46349	5.5	Cytohesin-3
4098	UPI0001F78FB4	2	3	4.6	453	51949	7.7	pleckstrin logy, Sec7 and coiled-coil domains 3
4099	P52655	2	5	5.3	376	41514	4.5	Transcription initiation factor IIA subunit 1
4099	Q99PM3	2	5	5.3	378	41614	4.5	Transcription initiation factor IIA subunit 1
4099	Q0VGZ0	2	5	5.3	378	41598	4.6	Gtf2a1 protein
4100	UPI00000372D7	2	2	6.4	393	43056	7.6	WD repeat-containing protein 13 isoform 2
4100	Q06DX0	2	2	6.4	393	43056	7.6	WDR13
4100	Q06DW8	2	2	6.4	393	43056	7.6	WDR13
4100	B2RBN3	2	2	5.2	485	53636	9.1	cDNA, FLJ95601, highly similar to sapiens WD repeat domain 13 (WDR13), mRNA
4100	Q91V09	2	2	5.2	485	53664	9.1	WD repeat-containing protein 13
4100	Q8K412	2	2	5.2	485	53680	9.1	Memory-related protein
4100	Q6NXG4	2	2	4.7	535	58715	9.2	WDR13 protein (Fragment)
4101	B4DRU9	2	2	5.1	668	75574	7.2	cDNA FLJ57179, highly similar to sapiens ATP-binding cassette, sub-family F (GCN20), member 3 (ABCF3), mRNA
4101	Q9NUQ8-2	2	2	4.8	703	78984	6.2	Isoform 2 of ATP-binding cassette sub-family F member 3
4101	Q9NUQ8	2	2	4.8	709	79745	6.3	ATP-binding cassette sub-family F member 3
4101	Q8K268	2	2	4.8	709	79865	6.2	ATP-binding cassette sub-family F member 3
4102	UPI000198CA16	2	5	13.8	240	26339	8.2	Peptide-N(4)-(N-acetyl-beta-glucosaminyl)asparagine amidase (EC 3.5.1.52) (PNGase) (hPNGase) (Peptide:N-glycanase) (N-glycanase 1).
4102	Q96IV0-3	2	5	5.9	558	63828	8.1	Isoform 3 of Peptide-N(4)-(N-acetyl-beta-glucosaminyl)asparagine amidase
4102	Q96IV0-2	2	5	5.2	636	72285	8.1	Isoform 2 of Peptide-N(4)-(N-acetyl-beta-glucosaminyl)asparagine amidase
4102	Q9JI78	2	5	5.1	651	74275	6.9	Peptide-N(4)-(N-acetyl-beta-glucosaminyl)asparagine amidase
4102	Q96IV0	2	5	5	654	74390	6.9	Peptide-N(4)-(N-acetyl-beta-glucosaminyl)asparagine amidase
4103	B1ARR0	2	4	4.6	604	67810	8.8	Netrin 1
4103	O95631	2	4	4.6	604	67748	8.8	Netrin-1
4103	O09118	2	4	4.6	604	67768	8.8	Netrin-1
4104	B4E192	2	164	4.7	407	47336	5.3	cDNA FLJ54224, highly similar to Glial fibrillary acidic protein, astrocyte
4104	B4DJW0	2	164	4.6	415	48200	5.5	cDNA FLJ55696, highly similar to Glial fibrillary acidic protein, astrocyte
4104	P14136-3	2	164	4.4	431	49506	6.1	Isoform 3 of Glial fibrillary acidic protein
4104	P14136	2	164	4.4	432	49880	5.5	Glial fibrillary acidic protein
4104	P03995-2	2	164	4.4	428	49365	5.5	Isoform 2 of Glial fibrillary acidic protein
4104	P03995	2	164	4.4	430	49900	5.3	Glial fibrillary acidic protein
4104	E9PAX3	2	164	4.4	431	49508	6.1	Uncharacterized protein

Table S1. Proteins identified in the OVCAR-3 tumor supernatant by two or more unique peptides.

Protein or Protein Group	Accession Number	Unique Peptide Count	Spectrum Count	Sequence Coverage (%)	Length	MolWt	pI	Descriptive Name
4104	P14136-2	2	164	4.3	438	50289	5.7	Isoform 2 of Glial fibrillary acidic protein
4105	Q5SRX1-5	2	8	7.7	286	31789	5.2	Isoform 5 of TOM1-like protein 2
4105	Q5SRX1-3	2	8	5	440	48408	5.1	Isoform 3 of TOM1-like protein 2
4105	Q5SRX1-4	2	8	4.9	450	49439	5.1	Isoform 4 of TOM1-like protein 2
4105	B7Z2L7	2	8	4.6	483	53368	4.8	cDNA FLJ60511, highly similar to Mus musculus target of myb1-like 2 (chicken) (Tom1l2), transcript variant 1, mRNA
4105	Q5SRX1-2	2	8	4.5	487	53383	4.9	Isoform 2 of TOM1-like protein 2
4105	Q6ZVM7	2	8	4.3	507	55556	4.8	TOM1-like protein 2
4105	Q5SRX1	2	8	4.3	507	55663	4.8	TOM1-like protein 2
4106	Q9NXS9	2	5	7.1	424	47994	6.9	CDNA FLJ20071 fis, clone COL01887
4106	Q7RTS9-2	2	5	6.3	479	54425	5.8	Isoform 2 of Dymeclin
4106	Q7RTS9	2	5	4.5	669	75936	5.9	Dymeclin
4106	Q8CHY3	2	5	4.5	669	75848	5.8	Dymeclin
4107	B4DF77	2	3	4.4	499	54461	9.1	cDNA FLJ58767, highly similar to Phosphofurin acidic cluster sorting protein 1
4107	Q8K212	2	3	2.3	961	104829	7.7	Phosphofurin acidic cluster sorting protein 1
4107	Q6VY07	2	3	2.3	963	104898	7.7	Phosphofurin acidic cluster sorting protein 1
4107	Q6VY07-2	2	3	2.2	1008	109342	7.4	Isoform 2 of Phosphofurin acidic cluster sorting protein 1
4108	Q9JK48	2	2	4.4	365	40855	6	Endophilin-B1
4108	Q9Y371	2	2	4.4	365	40796	6	Endophilin-B1
4108	Q9Y371-2	2	2	4.1	386	43196	5.7	Isoform 2 of Endophilin-B1
4108	Q9JK48-2	2	2	4.1	386	43239	5.7	Isoform 2 of Endophilin-B1
4109	B3KXT2	2	3	4.3	650	72236	5.7	cDNA FLJ45998 fis, clone SMINT2009292, highly similar to sapiens NMDA receptor regulated 2 (NARG2), transcript variant 1, mRNA
4109	Q659A1	2	3	2.9	982	110011	7.1	NMDA receptor-regulated protein 2
4109	Q3UZ18	2	3	2.8	988	109879	6.8	NMDA receptor-regulated protein 2
4110	B3KY61	2	5	4.3	793	88802	9.4	cDNA FLJ16787 fis, clone PLACE6013222, weakly similar to Mus musculus RNA binding motif protein 26 (Rbm26), mRNA (Fragment)
4110	Q5SFM8-2	2	5	3.4	1005	112715	9.1	Isoform 2 of RNA-binding protein 27
4110	E9QM05	2	5	3.4	1005	112705	9.1	Uncharacterized protein
4110	Q9P2N5	2	5	3.2	1060	118718	9.2	RNA-binding protein 27
4110	Q5SFM8	2	5	3.2	1060	118561	9.2	RNA-binding protein 27
4110	E9QM85	2	5	3.2	1060	118551	9.2	Uncharacterized protein
4111	UPI00000EA81A	2	4	5	579	64194	4.7	UPI00000EA81A UniRef100 entry
4111	E9PB39	2	4	4.8	610	68223	4.7	Uncharacterized protein
4111	C9JE94	2	4	4.3	679	77512	6.3	Uncharacterized protein
4111	UPI0001DD33FA	2	4	2.9	988	109407	8	UPI0001DD33FA UniRef100 entry
4111	UPI00001AE820	2	4	2.9	988	109653	8	UPI00001AE820 UniRef100 entry
4111	Q8C033-2	2	4	2.2	1306	143451	5.6	Isoform 2 of Rho guanine nucleotide exchange factor 10
4111	Q8C033	2	4	2.2	1345	147945	5.7	Rho guanine nucleotide exchange factor 10
4111	Q3UGZ6	2	4	2.2	1345	147944	5.8	Putative uncharacterized protein
4111	O15013-7	2	4	2.2	1306	144269	5.4	Isoform 3 of Rho guanine nucleotide exchange factor 10
4111	O15013-5	2	4	2.2	1344	148869	5.6	Isoform 5 of Rho guanine nucleotide exchange factor 10
4111	O15013-4	2	4	2.2	1340	148328	5.8	Isoform 4 of Rho guanine nucleotide exchange factor 10
4111	O15013-6	2	4	2.1	1368	151483	5.7	Isoform 2 of Rho guanine nucleotide exchange factor 10
4111	O15013	2	4	2.1	1369	151611	5.7	Rho guanine nucleotide exchange factor 10
4112	Q86SW5	2	2	8.7	242	27093	8.6	Full-length cDNA 5-PRIME end of clone CS0DL002YI06 of B cells (Ramos cell line) of sapiens (human) (Fragment)
4112	Q05BE7	2	2	6.5	322	36186	9.5	Numb protein
4112	B1P2N8	2	2	4.7	445	48825	8.4	Numb isoform 8
4112	B1P2N7	2	2	4.6	456	50198	8.7	Numb isoform 7
4112	B1P2N6	2	2	4.3	494	53947	7.6	Numb isoform 6
4112	B1P2N5	2	2	4.2	505	55319	8.1	Numb isoform 5
4112	Q9QZS3-4	2	2	3.5	593	64394	8.5	Isoform 4 of Protein numb log
4112	Q9QZS3-2	2	2	3.5	604	65766	8.7	Isoform 2 of Protein numb log
4112	P49757-4	2	2	3.5	592	64527	8.4	Isoform 4 of Protein numb log
4112	P49757-2	2	2	3.5	603	65899	8.7	Isoform 2 of Protein numb log
4112	Q9QZS3-3	2	2	3.3	642	69440	8.4	Isoform 3 of Protein numb log
4112	P49757-3	2	2	3.3	640	69431	8.2	Isoform 3 of Protein numb log
4112	B2RCI6	2	2	3.3	640	69359	8.3	cDNA, FLJ96094, highly similar to sapiens numb log (Drosophila) (NUMB), mRNA
4112	Q9QZS3	2	2	3.2	653	70813	8.6	Protein numb log
4112	P49757	2	2	3.2	651	70804	8.5	Protein numb log
4113	B2RC26	2	2	4.2	766	88170	5.5	cDNA, FLJ95817, highly similar to sapiens ZYG log (ZYG), mRNA
4113	Q80ZJ6-2	2	2	4.2	766	87738	5.8	Isoform 2 of Protein zer-1 log
4113	Q7Z7L7	2	2	4.2	766	88170	5.6	Protein zer-1 log
4113	Q80ZJ6	2	2	4.1	779	89076	5.9	Protein zer-1 log
4114	Q6ZMI0-4	2	2	9.1	363	42133	6.9	Isoform 4 of KLRAQ motif-containing protein 1
4114	UPI000173A4AB	2	2	5.9	556	63251	7.9	UPI000173A4AB UniRef100 entry
4114	Q6ZMI0-3	2	2	5.7	574	65352	6.8	Isoform 3 of KLRAQ motif-containing protein 1
4114	B7ZKY7	2	2	4.5	738	83700	7.2	KLRAQ1 protein
4114	Q6ZMI0-2	2	2	4.3	769	87153	6.9	Isoform 2 of KLRAQ motif-containing protein 1
4114	B2RPZ6	2	2	4.2	780	88337	6.9	KLRAQ motif containing 1
4114	Q6ZMI0	2	2	4.2	780	88314	6.8	KLRAQ motif-containing protein 1
4114	Q3TDD9	2	2	4.2	780	88363	7	KLRAQ motif-containing protein 1
4115	B7ZLV6	2	2	4.2	853	97526	6.9	TAOK1 protein
4115	Q7L7X3	2	2	3.6	1001	116070	7.7	Serine/threonine-protein kinase TAO1
4115	Q5F2E8	2	2	3.6	1001	116050	7.6	Serine/threonine-protein kinase TAO1
4116	UPI000024DC9E	2	2	4.5	556	61453	6.6	menin isoform c
4116	Q8CI72	2	2	4.5	556	61479	6.6	Men1 protein
4116	E7EN32	2	2	4.5	555	61419	6.9	Uncharacterized protein
4116	O00255-3	2	2	4.3	575	63748	6.6	Isoform 3 of Menin
4116	D3YZC2	2	2	4.3	576	63752	6.4	Uncharacterized protein
4116	A5HBC6	2	2	4.1	610	67384	6.6	Menin W471C variant
4116	Q91UZ7	2	2	4.1	611	67501	6.4	MEN1 tumor suppressor
4116	O88559	2	2	4.1	611	67473	6.5	Menin
4116	O00255-2	2	2	4.1	610	67497	6.6	Isoform 2 of Menin
4116	O00255	2	2	4.1	615	68023	6.6	Menin
4116	E9Q4P8	2	2	4.1	616	68060	6.4	Uncharacterized protein
4116	A5HBD1	2	2	4.1	603	66644	6.4	Menin 375del21 variant
4116	A5HBD0	2	2	4.1	610	67501	6.6	Menin I147F variant
4116	A5HBC8	2	2	4.1	610	67510	6.7	Menin L413R variant
4116	A5HBC7	2	2	4.1	610	67451	6.6	Menin L414P variant
4117	B4DNM1	2	3	4.2	683	75982	8.6	YME1-like 1 (S. cerevisiae), isoform CRA_c
4117	A8K5H7	2	3	4.1	716	79845	8.6	cDNA FLJ77542, highly similar to sapiens YME1-like 1 (S. cerevisiae) (YME1L1), transcript variant 3, mRNA
4117	Q96TA2-2	2	3	4.1	716	79832	8.6	Isoform 2 of ATP-dependent zinc metalloprotease YME1L1
4117	O88967	2	3	4.1	715	80028	9	ATP-dependent zinc metalloprotease YME1L1
4117	Q96TA2	2	3	3.8	773	86455	8.8	ATP-dependent zinc metalloprotease YME1L1
4118	Q92783-2	2	2	5.5	403	44972	4.9	Isoform 2 of Signal transducing adapter molecule 1

Table S1. Proteins identified in the OVCAR-3 tumor supernatant by two or more unique peptides.

Protein or Protein Group	Accession Number	Unique Peptide Count	Spectrum Count	Sequence Coverage (%)	Length	MolWt	pI	Descriptive Name
4118	Q3UGN9	2	2	4.8	462	51145	4.9	Signal transducing adaptor molecule (SH3 domain and ITAM motif) 1
4118	B2RAY1	2	2	4.1	540	59150	4.8	cDNA, FLJ95184, highly similar to sapiens signal transducing adaptor molecule (SH3 domain and ITAM motif) 1 (STAM), mRNA
4118	Q92783	2	2	4.1	540	59180	4.8	Signal transducing adapter molecule 1
4118	Q3UMC8	2	2	4	553	60243	4.8	Putative uncharacterized protein
4118	P70297	2	2	4	548	59771	4.8	Signal transducing adapter molecule 1
4119	Q7Z721	2	3	4.7	451	51016	6.5	RPS6KB1 protein
4119	B4DDM0	2	3	4.4	481	54122	8.4	cDNA FLJ54786, highly similar to Ribosomal protein S6 kinase beta-1 (EC 2.7.11.1)
4119	Q8BSK8-2	2	3	4.2	502	56229	6.3	Isoform Alpha II of Ribosomal protein S6 kinase beta-1
4119	P23443-2	2	3	4.2	502	56189	6.4	Isoform Alpha II of Ribosomal protein S6 kinase beta-1
4119	B2R779	2	3	4	525	59106	6.7	cDNA, FLJ93319, highly similar to sapiens ribosomal protein S6 kinase, 70kDa, polypeptide 1(RPS6KB1), mRNA
4119	Q8BSK8	2	3	4	525	59218	6.7	Ribosomal protein S6 kinase beta-1
4119	Q5SWG1	2	3	4	525	59146	6.8	Ribosomal protein S6 kinase, polypeptide 1
4119	Q3UXD8	2	3	4	525	59145	7.1	Putative uncharacterized protein
4119	P23443	2	3	4	525	59140	6.7	Ribosomal protein S6 kinase beta-1
4120	B4E0Q1	2	2	4.1	758	86412	8.1	cDNA FLJ50335, highly similar to Trpc4-associated protein
4120	B4DQU2	2	2	4	782	89139	7.1	cDNA FLJ51744, highly similar to Trpc4-associated protein
4120	A2AQP2	2	2	3.9	789	89914	7.6	Transient receptor potential cation channel subfamily C member 4 associated protein
4120	Q9JLV2	2	2	3.9	797	90727	7.6	Short transient receptor potential channel 4-associated protein
4120	Q8TEL6	2	2	3.9	797	90852	7.6	Short transient receptor potential channel 4-associated protein
4120	E1P5Q0	2	2	3.9	789	90043	7.6	Transient receptor potential cation channel, subfamily C, member 4 associated protein, isoform CRA_e
4120	B2RDK8	2	2	3.9	797	90765	7.7	cDNA, FLJ96658, highly similar to sapiens transient receptor potential cation channel, subfamilyC, member 4 associated protein (TRPC4AP), mRNA
4121	A3KGB4	2	2	3.9	1114	127893	5.5	TBC1 domain family member 8B
4121	UPI0001D3B452	2	2	3.9	1114	128108	6	TBC1 domain family, member 8B (with GRAM domain) isoform a
4121	B9EKQ1	2	2	3.9	1110	127476	5.5	Tbc1d8b protein
4121	Q0IIM8	2	2	3.8	1120	128709	5.9	TBC1 domain family member 8B
4122	Q8WUA7-3	2	2	5.6	324	36514	6.1	Isoform 3 of TBC1 domain family member 22A
4122	B4DWA9	2	2	4.5	403	47100	6.6	cDNA FLJ57214, highly similar to TBC1 domain family member 22A
4122	Q8WUA7-2	2	2	4.1	439	50931	5.9	Isoform 2 of TBC1 domain family member 22A
4122	E9QMS2	2	2	4	448	51324	6.6	Uncharacterized protein
4122	B0QYI1	2	2	3.9	458	53040	6.1	TBC1 domain family, member 22A
4122	B0QYI3	2	2	3.8	470	53908	5.4	TBC1 domain family, member 22A
4122	Q8WUA7	2	2	3.5	517	59121	6	TBC1 domain family member 22A
4122	Q8R5A6-2	2	2	3.5	521	59938	6.4	Isoform 2 of TBC1 domain family member 22A
4122	Q8R5A6	2	2	3.5	516	59362	6.5	TBC1 domain family member 22A
4122	B3KP29	2	2	3.5	517	59160	6.1	cDNA FLJ31029 fis, clone HSYRA1000119, highly similar to TBC1 domain family member 22A
4122	B2RE30	2	2	3.5	517	59102	6.1	cDNA, FLJ96895, highly similar to sapiens TBC1 domain family, member 22A (TBC1D22A), mRNA
4123	Q6ZMM9	2	7	6.3	317	35359	6.7	cDNA FLJ16815 fis, clone THYMU3044175, highly similar to Adenosylcysteinase (EC 3.3.1.1)
4123	Q80TQ9	2	7	4.2	478	53495	6.6	Adenosylcysteinase (Fragment)
4123	B4E168	2	7	4.1	483	53754	7.5	Adenosylcysteinase
4123	Q2NKW8	2	7	4	505	56469	7.8	Adenosylcysteinase (Fragment)
4123	B4DIZ5	2	7	3.9	508	56697	8.1	Adenosylcysteinase
4123	UPI0001B7934E	2	7	3.9	518	58118	8	UPI0001B7934E UniRef100 entry
4123	Q68FL4-2	2	7	3.9	508	56757	8.1	Isoform 2 of Putative adenosylcysteinase 3
4123	D9N155	2	7	3.9	508	56944	7.1	Adenosylcysteinase Adenosylcysteinase
4123	D7UEQ7	2	7	3.9	509	57072	7.1	Adenosylcysteinase
4123	Q6ZNS6	2	7	3.8	530	59596	8	Adenosylcysteinase
4123	O43865	2	7	3.8	530	58951	6.9	Putative adenosylcysteinase 2 Putative adenosylcysteinase 2
4123	Q96HN2-2	2	7	3.3	610	66593	7.4	Isoform 2 of Putative adenosylcysteinase 3
4123	Q96HN2	2	7	3.3	611	66721	7.4	Putative adenosylcysteinase 3
4123	Q68FL4	2	7	3.3	613	66899	7.4	Putative adenosylcysteinase 3
4123	E9PZ90	2	7	3.3	612	66771	7.4	Uncharacterized protein
4124	UPI0000D77ABF	2	3	4.3	603	67324	6.7	UPI0000D77ABF UniRef100 entry
4124	Q14161-11	2	3	4.1	631	70183	6.6	Isoform 11 of ARF GTPase-activating protein GIT2
4124	Q14161-7	2	3	4	646	71967	6.7	Isoform 7 of ARF GTPase-activating protein GIT2
4124	Q14161-6	2	3	3.9	661	73403	6.7	Isoform 6 of ARF GTPase-activating protein GIT2
4124	C9JXL4	2	3	3.8	681	76046	7.5	Uncharacterized protein
4124	UPI0000167B56	2	3	3.8	679	75734	7.2	ARF GTPase-activating protein GIT2 isoform 3
4124	Q80XR8	2	3	3.8	679	75632	7.5	G protein-coupled receptor kinase-interactor 2
4124	Q14161-10	2	3	3.8	681	76072	7.5	Isoform 10 of ARF GTPase-activating protein GIT2
4124	D3Z1I2	2	3	3.8	681	75971	7.7	Uncharacterized protein
4124	Q9JLQ2	2	3	3.7	708	78796	7.6	ARF GTPase-activating protein GIT2
4124	Q14161-8	2	3	3.7	694	77518	7.3	Isoform 8 of ARF GTPase-activating protein GIT2
4124	Q14161-4	2	3	3.7	709	78954	7.5	Isoform 4 of ARF GTPase-activating protein GIT2
4124	E9QPU7	2	3	3.7	708	78766	7.7	Uncharacterized protein
4124	Q5DU47	2	3	3.6	722	79774	8.1	MKIAA0148 protein (Fragment)
4124	Q14161-5	2	3	3.6	729	81322	7	Isoform 5 of ARF GTPase-activating protein GIT2
4124	Q14161-3	2	3	3.5	744	82759	7.1	Isoform 3 of ARF GTPase-activating protein GIT2
4124	Q14161	2	3	3.4	759	84543	7.2	ARF GTPase-activating protein GIT2
4124	E9PVA6	2	3	3.4	759	84457	7.6	Uncharacterized protein
4125	Q15291-2	2	3	4.2	500	55072	5	Isoform 2 of Retinoblastoma-binding protein 5
4125	Q8BX09	2	3	3.9	538	59098	5.1	Retinoblastoma-binding protein 5
4125	Q15291	2	3	3.9	538	59153	5.1	Retinoblastoma-binding protein 5
4125	B4DMM7	2	3	3.7	573	63304	5.2	cDNA FLJ59722, highly similar to Retinoblastoma-binding protein 5
4126	B4DSE0	2	2	3.8	928	103510	5.6	Ephrin receptor
4126	A6NJM0	2	2	3.6	981	109460	5.6	Ephrin receptor
4126	A3KG00	2	2	3.5	987	110055	5.8	Ephrin receptor
4126	Q4LE53	2	2	3.5	1004	111512	5.7	Ephrin receptor (Fragment)
4126	P54763-2	2	2	3.5	995	110916	5.6	Isoform 2 of Ephrin type-B receptor 2
4126	P54763	2	2	3.5	994	110760	5.6	Ephrin type-B receptor 2
4126	P29323-3	2	2	3.5	987	110030	5.7	Isoform 3 of Ephrin type-B receptor 2
4126	A3KG02	2	2	3.5	987	110027	5.7	Ephrin receptor
4126	A3KG01	2	2	3.5	986	109899	5.7	Ephrin receptor
4126	Q6P5F1	2	2	3.4	1029	114283	6.3	Ephrin receptor (Fragment)
4126	P29323	2	2	3.3	1055	117493	6.5	Ephrin type-B receptor 2
4127	Q6PIN6	2	2	12.1	149	16694	5.9	GIT1 protein (Fragment)
4127	Q3V322	2	2	7.1	253	27752	5.7	Putative uncharacterized protein
4127	B4DS81	2	2	5.1	351	38692	6.1	cDNA FLJ57186, highly similar to ARF GTPase-activating protein GIT1

Table S1. Proteins identified in the OVCAR-3 tumor supernatant by two or more unique peptides.

Protein or Protein Group	Accession Number	Unique Peptide Count	Spectrum Count	Sequence Coverage (%)	Length	MolWt	pI	Descriptive Name
4127	B4DMF7	2	2	3.5	520	57821	6	cDNA FLJ58515, highly similar to ARF GTPase-activating protein GIT1
4127	Q9Y2X7	2	2	2.4	761	84341	6.8	ARF GTPase-activating protein GIT1
4127	Q5F258	2	2	2.4	761	84195	6.7	G protein-coupled receptor kinase-interactor 1
4127	B4DSV3	2	2	2.4	747	83132	6.9	cDNA FLJ60869, highly similar to ARF GTPase-activating protein GIT1
4127	Q68FF6	2	2	2.3	770	85300	6.9	ARF GTPase-activating protein GIT1
4127	Q59FC3	2	2	2.3	774	85900	7.1	G protein-coupled receptor kinase interactor 1 variant (Fragment)
4128	Q921C5-3	2	2	6.8	338	39315	5	Isoform 3 of Protein bicaudal D log 2
4128	Q3TBD4	2	2	3.1	746	84716	5.7	Putative uncharacterized protein
4128	Q921C5	2	2	2.8	820	93391	5.4	Protein bicaudal D log 2
4128	Q8TD16	2	2	2.8	824	93533	5.4	Protein bicaudal D log 2
4128	Q921C5-2	2	2	2.7	851	96647	5.4	Isoform 2 of Protein bicaudal D log 2
4128	Q8TD16-2	2	2	2.7	855	96806	5.4	Isoform 2 of Protein bicaudal D log 2
4129	Q8N6Z3	2	3	4.1	463	51805	7.2	AVL9 protein
4129	UPI0001AE70A4	2	3	3.4	559	61894	5.5	K0241_HUMAN Isoform 2 of Q8NBF6 - sapiens (Human)
4129	UPI0000375385	2	3	3.3	568	63293	6.2	UPI0000375385 UniRef100 entry
4129	Q8NBF6-2	2	3	3.2	591	65313	6	Isoform 2 of Late secretory pathway protein AVL9 log
4129	B8ZZW5	2	3	3	630	69801	6	Uncharacterized protein
4129	Q8NBF6	2	3	2.9	648	71947	6.2	Late secretory pathway protein AVL9 log
4129	Q80U56	2	3	2.9	649	72186	6.1	Late secretory pathway protein AVL9 log
4130	Q8CBI2	2	6	3.7	512	56877	5	Putative uncharacterized protein
4130	A2AD03	2	6	2.9	662	73664	4.7	Choroidermia
4130	Q9QXG2	2	6	2.9	665	73977	4.7	Rab proteins geranylgeranyltransferase component A 1
4130	P24386	2	6	2.9	653	73476	4.7	Rab proteins geranylgeranyltransferase component A 1
4130	A8K545	2	6	2.9	653	73492	4.7	cDNA FLJ78016, highly similar to sapiens choroideremia (Rab escort protein 1) (CHM), transcript variant, mRNA
4131	UPI0001889C7E	2	6	9.7	299	32989	7.5	UPI0001889C7E UniRef100 entry
4131	B2RNR6	2	6	2.7	1074	117014	9	Zinc finger RNA binding protein
4131	Q96KR1	2	6	2.7	1074	117012	9	Zinc finger RNA-binding protein
4131	O88532	2	6	2.7	1074	116859	9	Zinc finger RNA-binding protein
4131	B5MEH6	2	6	2.7	1056	115154	9.3	Uncharacterized protein
4132	Q8BXH1	2	2	6	368	41932	9.4	Putative uncharacterized protein (Fragment)
4132	A2AT91	2	2	2.5	883	100651	5.4	Phospholipase C, beta 4 (Fragment)
4132	Q5FWA6	2	2	2.5	892	101775	5.8	Plcb4 protein (Fragment)
4132	Q15147-2	2	2	2.2	1022	117162	7.1	Isoform 1 of 1-phosphatidylinositol-4,5-bisphosphate phosphodiesterase beta-4
4132	E2QRH8	2	2	2.1	1031	118024	7.1	Uncharacterized protein
4132	Q91UZ1	2	2	1.9	1175	134527	6.9	Phospholipase C beta 4
4132	Q15147	2	2	1.9	1175	134463	6.9	1-phosphatidylinositol-4,5-bisphosphate phosphodiesterase beta-4
4132	Q15147-4	2	2	1.8	1194	136106	6.8	Isoform 3 of 1-phosphatidylinositol-4,5-bisphosphate phosphodiesterase beta-4
4133	Q52KI8-2	2	17	2.5	897	101163	11.9	Isoform 2 of Serine/arginine repetitive matrix protein 1
4133	E9QKA4	2	17	2.5	897	101162	11.9	Uncharacterized protein
4133	A2A8V8	2	17	2.4	909	102660	11.8	Serine/arginine repetitive matrix 1
4133	Q8IYB3-2	2	17	2.4	902	102126	11.8	Isoform 2 of Serine/arginine repetitive matrix protein 1
4133	Q8IYB3	2	17	2.4	904	102335	11.8	Serine/arginine repetitive matrix protein 1
4133	E9PUK6	2	17	2.4	923	104306	11.9	Uncharacterized protein
4133	B7Z7U0	2	17	2.4	916	103797	11.8	cDNA FLJ61739, highly similar to Serine/arginine repetitive matrix protein 1
4133	A9Z1X7	2	17	2.4	913	103390	11.8	Serine/arginine repetitive matrix 1
4133	A2A8V9	2	17	2.4	918	103715	11.9	Serine/arginine repetitive matrix 1
4133	Q52KI8	2	17	2.3	946	106892	11.9	Serine/arginine repetitive matrix protein 1
4133	E9QNW8	2	17	2.3	946	106862	11.9	Uncharacterized protein
4134	Q8BM64	2	3	4.1	682	73972	5.3	Putative uncharacterized protein
4134	Q6PIJ6-2	2	3	2.5	1113	125957	6.2	Isoform 2 of F-box only protein 38
4134	Q6PIJ6	2	3	2.4	1188	133944	6.3	F-box only protein 38
4134	Q8BMI0	2	3	2.3	1194	133928	6.1	F-box only protein 38
4135	Q59GY7	2	2	2.4	765	87535	6.5	Signal transducer and activator of transcription 5A variant (Fragment)
4135	A8K6I5	2	2	2.3	794	90646	6.3	cDNA FLJ75126, highly similar to sapiens signal transducer and activator of transcription 5A (STAT5A), mRNA
4135	Q8WWS9	2	2	2.3	791	90350	6.4	Signal transducer and activator of transcription 5A
4135	Q59H39	2	2	2.3	788	89966	6.2	Signal transducer and activator of transcription 5B variant (Fragment)
4135	P51692	2	2	2.3	787	89866	6.1	Signal transducer and activator of transcription 5B
4135	P42232	2	2	2.3	786	90002	6.1	Signal transducer and activator of transcription 5B
4135	P42230	2	2	2.3	793	90831	6.4	Signal transducer and activator of transcription 5A
4135	P42229	2	2	2.3	794	90647	6.4	Signal transducer and activator of transcription 5A
4135	B2C3G8	2	2	2.3	797	91623	7.7	STAT5a variant delta E18
4136	Q59FL3	2	3	2.1	1289	134543	5.3	Notch log 3 variant (Fragment)
4136	Q9UM47	2	3	1.2	2321	243629	5.4	Neurogenic locus notch log protein 3
4136	Q61982	2	3	1.2	2318	244246	5.3	Neurogenic locus notch log protein 3
4137	B7Z3G4	2	5	2.7	789	90990	6.2	cDNA FLJ60271, highly similar to SLIT-ROBO Rho GTPase-activating protein 2
4137	UPI00003E59F4	2	5	2.5	836	95843	6	SLIT-ROBO Rho GTPase-activating protein 2 isoform b
4137	A2RUF3	2	5	2	1071	120870	6.7	SLIT-ROBO Rho GTPase activating protein 2
4137	Q91Z67	2	5	2	1071	120798	6.6	SLIT-ROBO Rho GTPase-activating protein 2
4137	O75044	2	5	2	1071	120880	6.7	SLIT-ROBO Rho GTPase-activating protein 2
4137	B7ZM87	2	5	2	1070	120742	6.7	SRGAP2 protein
4138	C4IXU1	2	2	2	1118	126652	5.4	Phosphodiesterase 4D interacting protein (Myomegalin)
4138	Q80YT7-2	2	2	2	1118	126615	5.3	Isoform 2 of Myomegalin
4138	E9PL24	2	2	2	1116	126897	5.3	Uncharacterized protein
4138	C9JZD2	2	2	1.9	1131	128351	5.1	Uncharacterized protein
4139	B2RTQ5	2	5	1.8	1365	150914	7.8	LRRC16A protein
4139	UPI0001CB792A	2	5	1.8	1365	150884	7.8	leucine-rich repeat-containing protein 16A isoform 2
4139	Q5VZK9-2	2	5	1.8	1326	147087	7.8	Isoform 2 of Leucine-rich repeat-containing protein 16A
4139	Q5VZK9	2	5	1.8	1371	151556	7.9	Leucine-rich repeat-containing protein 16A
4139	E9PU95	2	5	1.8	1330	146814	8	Uncharacterized protein
4139	D3Z030	2	5	1.8	1332	147155	7.9	Uncharacterized protein
4139	B8X1J0	2	5	1.8	1371	151586	7.9	CARMIL1a
4139	Q6EDY6	2	5	1.7	1374	151860	7.8	Leucine-rich repeat-containing protein 16A
4139	D3Z024	2	5	1.7	1374	151916	7.8	Uncharacterized protein
4140	E7EMW7	2	6	1.8	2792	308574	5.8	Uncharacterized protein
4140	UPI0001E6FA93	2	6	1.8	2806	310363	6	PREDICTED: e3 ubiquitin-protein ligase UBR5-like isoform 1, partial
4140	UPI000153C072	2	6	1.8	2798	309223	5.8	E3 ubiquitin-protein ligase EDD1 (EC 6.3.2.-) (Hyperplastic discs protein log) (hHYD) (Progestin-induced protein).
4140	UPI0000607490	2	6	1.8	2792	308318	5.8	E3 ubiquitin-protein ligase UBR5 isoform 2
4140	Q80TP3	2	6	1.8	2792	308351	5.9	E3 ubiquitin-protein ligase UBR5
4140	O95071	2	6	1.8	2799	309352	5.8	E3 ubiquitin-protein ligase UBR5

Table S1. Proteins identified in the OVCAR-3 tumor supernatant by two or more unique peptides.

Protein or Protein Group	Accession Number	Unique Peptide Count	Spectrum Count	Sequence Coverage (%)	Length	MolWt	pI	Descriptive Name
4140	E9Q2H1	2	6	1.8	2798	308967	5.8	Uncharacterized protein
4141	P12036-2	2	15	1.9	963	105639	5.9	Isoform 2 of Neurofilament heavy polypeptide
4141	P12036	2	15	1.8	1026	112479	6.2	Neurofilament heavy polypeptide
4141	UPI00001AEF71	2	15	1.8	1020	111838	6.1	neurofilament heavy polypeptide
4141	UPI000024DAAE	2	15	1.7	1046	112570	5.7	neurofilament, heavy polypeptide
4141	Q80TQ3	2	15	1.7	1046	112544	5.7	MKIAA0845 protein (Fragment)
4141	P19246	2	15	1.7	1090	116994	5.8	Neurofilament heavy polypeptide
4142	Q53QS8	2	4	4	552	61036	6.9	Putative uncharacterized protein ERBB4 (Fragment)
4142	UPI000198C696	2	4	3	731	81013	6.9	Receptor tyrosine-protein kinase erbB-4 precursor (EC 2.7.10.1) (p180erbB4) (Tyrosine kinase-type cell surface receptor HER4).
4142	B2KGF7	2	4	3	732	81204	6.9	V-erb-a erythroblastic leukemia viral oncogene log 4 (Avian) (Fragment)
4142	B2KGF5	2	4	1.7	1292	145245	6.4	V-erb-a erythroblastic leukemia viral oncogene log 4 (Avian)
4142	Q61527-3	2	4	1.7	1308	146922	6.4	Isoform JM-A CYT-1 of Receptor tyrosine-protein kinase erbB-4
4142	Q61527-2	2	4	1.7	1282	143953	6.2	Isoform JM-B CYT-2 of Receptor tyrosine-protein kinase erbB-4
4142	Q61527	2	4	1.7	1292	145213	6.4	Receptor tyrosine-protein kinase erbB-4
4142	Q15303-4	2	4	1.7	1282	143968	6.2	Isoform JM-B CYT-2 of Receptor tyrosine-protein kinase erbB-4
4142	Q15303-3	2	4	1.7	1292	145198	6.4	Isoform JM-A CYT-2 of Receptor tyrosine-protein kinase erbB-4
4142	Q15303-2	2	4	1.7	1298	145578	6.2	Isoform JM-B CYT-1 of Receptor tyrosine-protein kinase erbB-4
4142	Q15303	2	4	1.7	1308	146808	6.4	Receptor tyrosine-protein kinase erbB-4
4142	E9QL41	2	4	1.7	1282	143985	6.2	Uncharacterized protein
4142	B2KGF6	2	4	1.7	1308	146855	6.4	V-erb-a erythroblastic leukemia viral oncogene log 4 (Avian)
4143	B2RX07	2	2	1.6	1553	172760	8.4	Cleavage and polyadenylation factor subunit log (S. cerevisiae)
4143	O94913	2	2	1.6	1555	173050	8.5	Pre-mRNA cleavage complex 2 protein Pcf11
4143	Q69ZY3	2	2	1.5	1641	182204	8.8	MKIAA0824 protein (Fragment)
4144	B4E0T8	2	3	2.5	1181	133547	7.1	cDNA FLJ59649, highly similar to Myosin-18A
4144	Q5QD01	2	3	1.8	1581	180550	5.9	SP-A receptor subunit SP-R210 alphaS
4144	E9QA74	2	3	1.8	1642	186535	5.9	Uncharacterized protein
4144	B4DYM1	2	3	1.8	1642	186534	5.9	cDNA FLJ60372, highly similar to Myosin-18A
4144	Q9JMH9-7	2	3	1.7	1716	195276	5.7	Isoform 7 of Myosin-XVIIIa
4144	Q9JMH9-5	2	3	1.7	1704	194046	5.7	Isoform 5 of Myosin-XVIIIa
4144	Q9JMH9-2	2	3	1.7	1719	195893	5.8	Isoform 2 of Myosin-XVIIIa
4144	Q92614-2	2	3	1.7	1723	196436	5.9	Isoform 2 of Myosin-XVIIIa
4144	E9Q405	2	3	1.7	1700	193645	5.8	Uncharacterized protein
4144	E9Q0J6	2	3	1.7	1722	195988	5.6	Uncharacterized protein
4144	Q9JMH9-4	2	3	1.5	1998	226356	6.2	Isoform 4 of Myosin-XVIIIa
4144	B2RRE2	2	3	1.4	2047	232136	6.1	Myo18a protein
4144	Q9JMH9-6	2	3	1.4	2062	233983	6.2	Isoform 6 of Myosin-XVIIIa
4144	Q9JMH9-1	2	3	1.4	2035	230906	6.2	Isoform 1 of Myosin-XVIIIa
4144	Q9JMH9	2	3	1.4	2050	232753	6.3	Myosin-XVIIIa
4144	Q92614-4	2	3	1.4	2039	231238	6.2	Isoform 4 of Myosin-XVIIIa
4144	Q92614-3	2	3	1.4	2002	226688	6.2	Isoform 3 of Myosin-XVIIIa
4144	Q92614	2	3	1.4	2054	233113	6.3	Myosin-XVIIIa
4144	E9QAX2	2	3	1.4	2083	235815	6.1	Uncharacterized protein
4145	Q3UG84	2	2	1.6	1359	150848	6.6	Putative uncharacterized protein (Fragment)
4145	Q3TBX9	2	2	1.6	1416	157258	6.7	Putative uncharacterized protein (Fragment)
4145	E9PGN1	2	2	1.3	1741	193334	7.1	Uncharacterized protein
4145	Q7TT21	2	2	1.3	1742	194097	6.9	Tuberous sclerosis 2
4145	Q61037-7	2	2	1.3	1704	189854	6.9	Isoform F of Tuberin
4145	Q3UGI8	2	2	1.3	1678	186575	6.8	Putative uncharacterized protein (Fragment)
4145	Q3TCQ7	2	2	1.3	1742	194047	7	Putative uncharacterized protein
4145	P49815-6	2	2	1.3	1704	189297	7	Isoform 6 of Tuberin
4145	P49815-5	2	2	1.3	1740	193247	7.1	Isoform 5 of Tuberin
4145	UPI0000434D93	2	2	1.2	1785	198849	7	tuberin isoform 1
4145	Q61037-6	2	2	1.2	1786	198913	7	Isoform E of Tuberin
4145	Q61037-5	2	2	1.2	1791	199549	6.9	Isoform D of Tuberin
4145	Q61037-4	2	2	1.2	1770	197162	6.8	Isoform C of Tuberin
4145	Q61037-3	2	2	1.2	1775	197890	7.1	Isoform B of Tuberin
4145	Q61037-2	2	2	1.2	1777	197981	7	Isoform A of Tuberin
4145	Q61037	2	2	1.2	1814	202070	7	Tuberin
4145	Q3UHB2	2	2	1.2	1808	201369	7.1	Putative uncharacterized protein
4145	P49815-4	2	2	1.2	1784	198096	7.3	Isoform 4 of Tuberin
4145	P49815-3	2	2	1.2	1763	195758	7.1	Isoform 3 of Tuberin
4145	P49815-2	2	2	1.2	1764	195845	7.1	Isoform 2 of Tuberin
4145	P49815	2	2	1.2	1807	200607	7.3	Tuberin
4145	E9QN11	2	2	1.2	1768	197107	6.9	Uncharacterized protein
4145	E9QN07	2	2	1.2	1773	197733	7.1	Uncharacterized protein
4145	E9QMZ9	2	2	1.2	1775	197878	7	Uncharacterized protein
4145	E9QMZ6	2	2	1.2	1789	199425	7	Uncharacterized protein
4145	E9QMW6	2	2	1.2	1812	201945	7	Uncharacterized protein
4145	E9QLU3	2	2	1.2	1786	198977	7	Uncharacterized protein
4146	Q9UKE5-8	2	2	1.3	1268	144251	7.1	Isoform 8 of TRAF2 and NCK-interacting protein kinase
4146	Q9UKE5-7	2	2	1.3	1297	147894	7	Isoform 7 of TRAF2 and NCK-interacting protein kinase
4146	Q9UKE5-6	2	2	1.3	1323	150365	7.4	Isoform 6 of TRAF2 and NCK-interacting protein kinase
4146	Q9UKE5-5	2	2	1.3	1276	145186	7.1	Isoform 5 of TRAF2 and NCK-interacting protein kinase
4146	Q9UKE5-4	2	2	1.3	1352	154008	7.2	Isoform 4 of TRAF2 and NCK-interacting protein kinase
4146	Q9UKE5-3	2	2	1.3	1305	148829	7	Isoform 3 of TRAF2 and NCK-interacting protein kinase
4146	Q9UKE5-2	2	2	1.3	1331	151300	7.4	Isoform 2 of TRAF2 and NCK-interacting protein kinase
4146	P83510-2	2	2	1.3	1289	146713	8	Isoform 2 of Traf2 and NCK-interacting protein kinase
4146	P83510	2	2	1.3	1323	150366	7.3	Traf2 and NCK-interacting protein kinase
4146	E9Q617	2	2	1.3	1289	146759	7.9	Uncharacterized protein
4146	E9PUL9	2	2	1.3	1331	151301	7.3	Uncharacterized protein
4146	E0CZF8	2	2	1.3	1268	144253	7.1	Uncharacterized protein
4146	E0CZD7	2	2	1.3	1276	145188	7.1	Uncharacterized protein
4146	E0CY98	2	2	1.3	1305	148831	6.9	Uncharacterized protein
4146	E0CXD6	2	2	1.3	1297	147896	6.9	Uncharacterized protein
4146	B9EKN8	2	2	1.3	1352	154010	7.1	TRAF2 and NCK interacting kinase
4146	B2RXX2	2	2	1.3	1323	150427	7.3	Tnik protein
4146	B2RQ80	2	2	1.2	1360	154945	7.1	Tnik protein
4146	UPI0001AADE61	2	2	1.2	1363	155263	7	traf2 and NCK-interacting protein kinase isoform 4
4146	Q9UKE5	2	2	1.2	1360	154943	7.2	TRAF2 and NCK-interacting protein kinase
4146	B2RY17	2	2	1.2	1363	155297	7	Tnik protein
4147	Q6GYP7-4	2	3	2.2	1037	117170	5.9	Isoform 5 of Ral GTPase-activating protein subunit alpha-1
4147	Q6GYQ0-3	2	3	2.1	1104	124733	6.2	Isoform 3 of Ral GTPase-activating protein subunit alpha-1
4147	Q6GYQ0-4	2	3	1.2	1961	221097	6.1	Isoform 4 of Ral GTPase-activating protein subunit alpha-1
4147	B7ZNA3	2	3	1.1	2082	234379	6.1	Garnl1 protein
4147	Q6GYQ0-6	2	3	1.1	2083	234851	6.2	Isoform 6 of Ral GTPase-activating protein subunit alpha-1
4147	Q6GYQ0-5	2	3	1.1	2073	234124	6.3	Isoform 5 of Ral GTPase-activating protein subunit alpha-1
4147	Q6GYQ0-2	2	3	1.1	2083	234729	6.3	Isoform 2 of Ral GTPase-activating protein subunit alpha-1
4147	Q6GYQ0	2	3	1.1	2036	229830	6.2	Ral GTPase-activating protein subunit alpha-1
4147	Q6GYP7-2	2	3	1.1	2068	232644	6.1	Isoform 2 of Ral GTPase-activating protein subunit alpha-1
4147	Q6GYP7	2	3	1.1	2035	229387	6.1	Ral GTPase-activating protein subunit alpha-1
4147	E9QMM1	2	3	1.1	2083	234238	6.2	Uncharacterized protein
4147	E9Q680	2	3	1.1	2082	234409	6.1	Uncharacterized protein

Table S1. Proteins identified in the OVCAR-3 tumor supernatant by two or more unique peptides.

Protein or Protein Group	Accession Number	Unique Peptide Count	Spectrum Count	Sequence Coverage (%)	Length	MolWt	pI	Descriptive Name
4147	E7EQJ8	2	3	1.1	2073	234154	6.3	Uncharacterized protein
4147	B9EK38	2	3	1.1	2049	231136	6.1	GARNL1 protein
4148	A2AQP0	2	4	1	1941	221495	5.9	Myosin-7B
4148	A7E2Y1	2	4	1	1941	221386	6	Myosin-7B
4149	E9PX61	2	4	0.7	4126	452569	6.2	Uncharacterized protein
4149	Q9Y4D8-4	2	4	0.7	3984	438033	6.2	Isoform 4 of Probable E3 ubiquitin-protein ligase C12orf51
4149	Q9Y4D8	2	4	0.7	3996	439348	6.2	Probable E3 ubiquitin-protein ligase C12orf51
4149	UPI0001E6B9D9	2	4	0.6	4392	481720	6.1	PREDICTED: LOW QUALITY PROTEIN: probable E3 ubiquitin-protein ligase KIAA0614
4150	Q5VST9-5	2	3	0.5	3911	427048	6.6	Isoform 4 of Obscurin
4150	UPI00004578F0	2	3	0.3	6620	721580	5.8	obscurin isoform a
4150	Q5VST9-3	2	3	0.3	6620	721557	5.7	Isoform 3 of Obscurin
4150	A2AAJ9-2	2	3	0.3	7167	779871	5.9	Isoform 2 of Obscurin
4150	A2AAJ9	2	3	0.2	8891	966386	5.9	Obscurin
4150	UPI0001C2BFA8	2	3	0.2	8025	873829	5.9	obscurin isoform 1
4150	UPI0001C2BFA7	2	3	0.2	7489	814925	5.8	obscurin isoform 2
4150	UPI0001545E46	2	3	0.2	7968	868519	6	obscurin isoform b
4150	Q5VST9	2	3	0.2	7968	868496	6	Obscurin
4150	E9QQ96	2	3	0.2	7490	815053	5.8	Uncharacterized protein
4150	E9QNK1	2	3	0.2	8026	873957	5.9	Uncharacterized protein
4151	Q7TPR4	2	20	78.7	892	103068	5.4	Alpha-actinin-1
4152	B0LAA9	2	12	71.1	76	8484	10	Phosphoglycerate kinase (Fragment)
4153	P62983	2	11	68.6	156	17951	9.6	Ubiquitin-40S ribosomal protein S27a
4154	P47955	2	7	51.8	114	11475	4.3	60S acidic ribosomal protein P1
4155	P57776	2	5	49.5	281	31293	5	Elongation factor 1-delta
4155	P57776-3	2	5	21.1	660	72931	6.4	Isoform 3 of Elongation factor 1-delta
4156	E9QAV5	2	2	46.9	326	37413	4.7	Uncharacterized protein
4157	D4A9P7	2	5	41.9	86	10187	5.8	RCG40058
4157	Q8BGS2	2	5	41.9	86	10215	6.2	BolA-like protein 2
4158	P99025	2	3	40.5	84	9584	6.3	GTP cyclohydrolase 1 feedback regulatory protein
4159	E9Q456	2	4	39.9	248	28668	4.8	Uncharacterized protein
4160	P08009	2	6	38.5	218	25681	7.3	Glutathione S-transferase Yb-3
4161	UPI0000512260	2	10	37.1	89	10273	7.9	hypothetical protein LOC627788
4162	Q62426	2	12	32.7	98	11046	7.4	Cystatin-B
4163	Q61820	2	3	31.9	216	24452	6.5	GTP-binding nuclear protein Ran, testis-specific isoform
4164	Q8K5B2	2	3	28.3	145	16168	4.7	Multiple coagulation factor deficiency protein 2 homolog
4165	P33622	2	22	38.4	99	10982	4.7	Apolipoprotein C-III
4165	E9QP56	2	22	27.7	137	15163	5.6	Uncharacterized protein
4166	Q6P7T7	2	14	27.6	76	8801	6.5	Rab2b protein
4167	UPI00006037ED	2	4	27.7	119	13294	6.5	PREDICTED: replication protein A 14 kDa subunit-like
4167	UPI000002273D	2	4	27.7	119	13256	5.5	PREDICTED: replication protein A 14 kDa subunit-like
4167	Q9CQ71	2	4	27.3	121	13584	4.8	Replication protein A 14 kDa subunit
4168	E9QMV2	2	8	27.2	81	9058	5.8	Uncharacterized protein
4168	Q4KML4	2	8	27.2	81	9030	5.8	Costars family protein C6orf115 homolog
4169	B2CSK2	2	15	24.2	641	70607	6.2	Heat shock protein 1-like protein
4169	P16627	2	15	24.2	641	70637	6.2	Heat shock 70 kDa protein 1-like
4170	P34928	2	2	22.7	88	9696	9.1	Apolipoprotein C-I
4171	Q5M8N0	2	3	23.8	164	18612	8	CB1 cannabinoid receptor-interacting protein 1
4171	Q5F272	2	3	21.2	184	20726	9.4	Cannabinoid receptor interacting protein 1 (Fragment)
4172	Q9D8S9	2	2	21.2	137	14379	8.8	BolA-like protein 1
4173	E9Q138	2	6	20.4	137	15197	9.4	Uncharacterized protein
4173	E9PU89	2	6	19.6	143	15992	9.7	Uncharacterized protein
4173	P05017	2	6	18.3	153	17093	9.4	Insulin-like growth factor I
4173	Q4VJB9	2	6	17.6	159	17888	9.6	Insulin-like growth factor 1 isoform Eb
4173	Q8CAR0	2	6	17	165	18473	9.8	Putative uncharacterized protein
4174	Q3TVE3	2	7	19.4	124	14296	5.5	Putative uncharacterized protein
4174	Q9D708	2	7	19.4	124	14324	5.9	S100 calcium binding protein A16
4175	P21460	2	11	19.3	140	15531	9	Cystatin-C
4175	Q9EPX9	2	11	19.3	140	15517	8.8	Cystatin C
4176	Q4FJY2	2	4	19.1	152	17676	8.1	Ubiquitin carrier protein
4176	Q9QZU9	2	4	19	153	17841	8.1	Ubiquitin/ISG15-conjugating enzyme E2 L6
4177	Q8BH80	2	11	18.9	243	26918	7.8	Vesicle-associated membrane protein, associated protein B and C
4177	Q9QY76	2	11	18.9	243	26946	7.8	Vesicle-associated membrane protein-associated protein B
4178	Q920X5	2	3	32.4	105	11923	5	Cathelicidin (Fragment)
4178	P51437	2	3	19.7	173	19582	8.7	Cathelin-related antimicrobial peptide
4178	E9Q9D3	2	3	18.5	184	20663	8.8	Uncharacterized protein
4178	Q3TZ97	2	3	18.4	185	20750	8.8	Putative uncharacterized protein
4179	Q9CQ86	2	3	18.3	115	12295	4.5	Protein C17orf37 homolog
4180	Q9D937	2	2	17.9	123	14099	11.5	MCG127334
4181	Q60648	2	2	16.6	193	20824	5.9	Ganglioside GM2 activator
4182	Q9WV35	2	2	16.5	224	25660	4.8	Probable C- U-editing enzyme APOBEC-2
4183	P24472	2	2	16.2	222	25564	7.4	Glutathione S-transferase A4
4184	D3YU00	2	2	16	206	23166	5.5	StAR-related lipid transfer (START) domain containing 5, isoform CRA_a
4184	Q9EPQ7	2	2	15.5	213	23922	6.4	StAR-related lipid transfer protein 5
4185	Q8R5J9	2	4	16	188	21558	9.6	PRA1 family protein 3
4186	Q8BFQ8	2	5	15.5	220	23277	7	Parkinson disease 7 domain-containing protein 1
4187	UPI0000025ADB	2	5	14.7	251	27965	5.7	apolipoprotein N
4188	Q9JHH9	2	3	14.6	205	22934	5.2	Coatomer subunit zeta-2
4189	Q14AS7	2	32	13.4	417	46677	7.2	Serine (Or cysteine) peptidase inhibitor, clade A, member 3C
4190	Q6S9I0	2	9	13.4	433	47887	6.2	Kng2 protein
4191	P63300	2	2	12.5	88	9537	8.7	Selenoprotein W
4192	Q60739-2	2	4	19.6	219	24875	5	Isoform 2 of BAG family molecular chaperone regulator 1
4192	Q60739	2	4	12.1	355	39740	8.5	BAG family molecular chaperone regulator 1
4192	UPI0000022EA1	2	4	12.1	355	39696	8.5	Bcl2-associated athanogene 1
4193	Q8K1I3	2	6	11.8	203	23136	8.4	Secreted phosphoprotein 24
4194	Q8QZV3	2	2	11.8	289	32250	9	Dci protein
4194	Q9DBN7	2	2	11.8	289	32223	9	Putative uncharacterized protein
4195	UPI0001E6B772	2	12	10.8	232	25487	10.1	PREDICTED: 40S ribosomal protein S2-like
4196	Q8BZS4	2	2	10.7	382	43148	9.5	Putative uncharacterized protein
4196	Q9D6X6	2	2	10.7	382	43072	9.5	Serine protease 23
4197	Q9JI75	2	3	11.7	231	26248	7	Ribosyldihydronicotinamide dehydrogenase [quinone]
4197	Q9CVI1	2	3	10.2	266	30226	8.9	Putative uncharacterized protein (Fragment)
4197	Q9CVF5	2	3	10.1	267	30503	9	Putative uncharacterized protein (Fragment)
4198	A2A5K2	2	6	9.8	441	49023	6.5	Phospholipid transfer protein
4198	Q4FJT5	2	6	8.7	493	54467	6.6	Pltp protein
4198	P55065	2	6	8.7	493	54453	6.6	Phospholipid transfer protein
4199	D3YTN4	2	2	9.8	328	38044	6.2	Uncharacterized protein
4200	Q3TEB0	2	2	9.8	379	42998	6.2	Putative uncharacterized protein
4201	Q9CQ37	2	3	9.8	204	22975	8	Ubiquitin-conjugating enzyme E2 T
4202	Q9CYT3	2	2	10	261	27274	9	Putative uncharacterized protein
4202	Q99N15	2	2	10	261	27274	8.8	17beta-hydroxysteroid dehydrogenase type 10/short chain L-3-hydroxyacyl-CoA dehydrogenase

Table S1. Proteins identified in the OVCAR-3 tumor supernatant by two or more unique peptides.

Protein or Protein Group	Accession Number	Unique Peptide Count	Spectrum Count	Sequence Coverage (%)	Length	MolWt	pI	Descriptive Name
4202	O08756	2	2	10	261	27419	8.4	3-hydroxyacyl-CoA dehydrogenase type-2
4202	A2AFQ2	2	2	9.6	271	28374	8.8	Hydroxysteroid (17-beta) dehydrogenase 10
4203	P03958	2	2	9.4	352	39992	5.7	Adenosine deaminase
4203	Q4FJZ7	2	2	9.4	352	39962	5.8	Ada protein
4204	UPI0001889CA2	2	5	21.2	151	16719	8.6	UPI0001889CA2 UniRef100 entry
4204	Q8R1G6	2	5	9.2	349	37703	8.7	PDZ and LIM domain protein 2
4205	B1AWZ5	2	2	9.1	197	22887	9.5	Nipsnap homolog 3A (C. elegans)
4205	Q9CQE1	2	2	7.3	247	28308	9.5	Protein NipSnap homolog 3A
4206	P43276	2	2	9	223	22576	10.9	Histone H1.5
4207	Q9ESP1	2	2	9	221	23648	7.4	Stromal cell-derived factor 2-like protein 1
4208	P70271	2	2	8.8	330	35572	8	PDZ and LIM domain protein 4
4208	Q5SWV2	2	2	8.8	330	35556	8	PDZ and LIM domain 4
4209	O08797	2	2	8.3	374	42259	5.4	SPI6
4210	P21300	2	3	8.2	316	35988	7.3	Aldose reductase-related protein 1
4210	Q3UGS8	2	3	8.2	316	35988	7.6	Putative uncharacterized protein
4211	Q91X52	2	3	7.8	244	25746	7.3	L-xylulose reductase
4212	Q9CX34	2	3	7.7	336	38159	5.4	Suppressor of G2 allele of SKP1 homolog
4213	Q9WVJ3-2	2	2	7.9	458	50491	6.4	Isoform 2 of Plasma glutamate carboxypeptidase
4213	Q9WVJ3	2	2	7.7	470	51813	6.2	Plasma glutamate carboxypeptidase
4214	Q3U6T2	2	3	7.5	415	47251	6.4	Putative uncharacterized protein
4214	Q9QWR8	2	3	7.5	415	47235	6.4	Alpha-N-acetylgalactosaminidase
4215	Q8R082	2	2	7.3	506	56254	5.4	Dipeptidylpeptidase 7
4215	Q9ET22	2	2	7.3	506	56270	5.4	Dipeptidyl peptidase 2
4216	E9Q0X7	2	2	7.2	472	52385	7.1	Uncharacterized protein
4216	Q3UM16	2	2	7.2	472	52351	7	Putative uncharacterized protein
4216	Q9Z175	2	2	4.5	754	83681	6.9	Lysyl oxidase homolog 3
4216	Q91VN8	2	2	4.5	754	83740	7	Lysyl oxidase-like 3
4217	Q62219-7	2	3	8.6	350	38289	7.5	Isoform 7 of Transforming growth factor beta-1-induced transcript 1 protein
4217	Q62219-5	2	3	7.5	402	44059	7	Isoform 5 of Transforming growth factor beta-1-induced transcript 1 protein
4217	Q62219-4	2	3	7.2	415	45266	6.6	Isoform 4 of Transforming growth factor beta-1-induced transcript 1 protein
4217	E9Q1D5	2	3	7.1	422	46006	6.9	Uncharacterized protein
4217	Q62219	2	3	6.5	461	50101	6.7	Transforming growth factor beta-1-induced transcript 1 protein
4217	Q62219-3	2	3	6.2	483	51988	7.6	Isoform 3 of Transforming growth factor beta-1-induced transcript 1 protein
4218	Q3UE99	2	5	12.8	243	27229	5.1	Putative uncharacterized protein (Fragment)
4218	O89017	2	5	7.1	435	49373	6.4	Legumain
4219	Q9CYG7	2	3	7.1	309	34278	9.1	Mitochondrial import receptor subunit TOM34
4219	Q9CYG7-2	2	3	7.1	309	34280	9.1	Isoform 2 of Mitochondrial import receptor subunit TOM34
4220	A8Y5C5	2	3	9.2	240	27264	4.8	Lymphocyte specific 1 (Fragment)
4220	A2A6J4	2	3	6.8	322	35763	5.1	Lymphocyte specific 1
4220	A2A6J7	2	3	6.8	324	35929	4.7	Lymphocyte specific 1
4220	P19973-2	2	3	6.7	328	36548	5.3	Isoform 2 of Lymphocyte-specific protein 1
4220	P19973	2	3	6.7	330	36714	4.8	Lymphocyte-specific protein 1
4221	Q71S36	2	2	9.5	379	40566	6	TAGL-M splice variant
4221	Q8VCS0-3	2	2	8	450	49063	6.8	Isoform 3 of N-acetylmuramoyl-L-alanine amidase
4221	Q8VCS0-2	2	2	7.2	501	54551	7.1	Isoform 2 of N-acetylmuramoyl-L-alanine amidase
4221	Q76L85	2	2	7.2	500	54448	7.1	TagL-beta
4221	A2TJ61	2	2	6.8	530	57587	7	Peptidoglycan recognition protein 2
4221	Q8VCS0	2	2	6.8	530	57707	7	N-acetylmuramoyl-L-alanine amidase
4221	Q54A77	2	2	6.8	529	57604	7	TagL-alpha
4222	Q64442	2	2	6.7	357	38249	7	Sorbitol dehydrogenase
4223	B1ATU5	2	3	6.6	482	54165	8.5	Dihydrouridine synthase 1-like (S. cerevisiae)
4224	Q91YJ2	2	3	6.4	450	51778	5.8	Sorting nexin-4
4225	P29758	2	2	6.2	439	48355	6.6	Ornithine aminotransferase, mitochondrial
4225	Q3UKT3	2	2	6.2	439	48354	6.8	Putative uncharacterized protein
4225	Q3UJK5	2	2	6.2	439	48418	6.6	Putative uncharacterized protein
4226	Q8R1M0	2	2	5.9	353	40169	8.6	UPF0361 protein C3orf37 homolog
4227	Q9D964	2	2	5.9	423	48297	7.9	Glycine amidinotransferase, mitochondrial
4228	Q921X9	2	2	5.8	517	59267	7.5	Protein disulfide-isomerase A5
4229	Q9QZ29	2	7	5.8	343	39246	5	Immunoglobulin-binding protein 1b
4229	UPI000002342D	2	7	5.8	343	39246	5	immunoglobulin-binding protein 1b
4230	P33587	2	2	5.2	460	51818	6.4	Vitamin K-dependent protein C
4231	Q8BH43	2	2	5.2	497	54074	5.5	Wiskott-Aldrich syndrome protein family member 2
4232	Q3TCN2-2	2	4	6.7	451	49936	5.9	Isoform 2 of Putative phospholipase B-like 2
4232	Q3TCN2	2	4	5.1	594	66290	6.1	Putative phospholipase B-like 2
4233	D3YUK7	2	4	5	682	76157	5.6	Uncharacterized protein
4233	Q8CG14	2	4	4.9	688	76858	5.1	Complement C1s-A subcomponent
4233	Q3T9K7	2	4	4.9	688	76485	5.1	Putative uncharacterized protein
4233	E9Q6C2	2	4	4.9	694	77500	5.1	Uncharacterized protein
4233	E9Q493	2	4	4.9	687	76513	5.1	Uncharacterized protein
4234	D3YWH0	2	5	19.4	124	13460	7.3	Uncharacterized protein
4234	D3YZE3	2	5	11.2	214	23248	5.9	Uncharacterized protein
4234	Q61398	2	5	5.1	468	50168	8.4	Procollagen C-endopeptidase enhancer 1
4234	Q3UN82	2	5	5.1	468	50236	8.4	Putative uncharacterized protein
4234	Q3TX39	2	5	5.1	468	50169	8.2	Putative uncharacterized protein
4234	D3YUE2	2	5	4.9	493	53134	8.5	Uncharacterized protein
4235	P28798	2	2	4.8	589	63458	6.8	Granulins
4235	Q3UC19	2	2	4.8	589	63504	6.8	Putative uncharacterized protein
4235	Q3TX66	2	2	4.8	589	63405	6.9	Putative uncharacterized protein
4235	Q3TWT4	2	2	4.8	589	63401	6.7	Putative uncharacterized protein
4235	Q3TVQ3	2	2	4.8	589	63382	6.9	Putative uncharacterized protein
4235	Q9D2V3	2	2	4.7	602	64965	6.9	Putative uncharacterized protein
4235	Q3UD85	2	2	4.7	602	65071	7.1	Putative uncharacterized protein
4235	Q3UAJ3	2	2	4.7	602	65002	7	Putative uncharacterized protein
4235	Q3U8W3	2	2	4.7	602	65019	6.9	Putative uncharacterized protein
4235	Q3U5Q6	2	2	4.7	602	64965	6.9	Putative uncharacterized protein
4235	Q3TW77	2	2	4.7	602	65004	7	Putative uncharacterized protein
4236	P81117	2	2	4.8	420	50305	5.1	Nucleobindin-2
4236	Q3UKN6	2	2	4.8	420	50354	5.1	Putative uncharacterized protein
4237	Q3ULX3	2	6	4.5	422	48013	6.2	Putative uncharacterized protein
4237	Q8CB30	2	6	4.5	422	48055	6.2	Putative uncharacterized protein
4238	D3YVU1	2	2	4.1	754	85382	6.4	Uncharacterized protein
4238	UPI0000022690	2	2	3.8	825	93434	6.2	ubiquitin carboxyl-terminal hydrolase 16
4238	Q99LG0	2	2	3.8	825	93406	6.2	Ubiquitin carboxyl-terminal hydrolase 16
4239	Q3USK2	2	2	4	428	49121	9.9	Casein kinase 1, delta, isoform CRA_b
4240	Q61210-3	2	3	4.7	791	89294	6.1	Isoform 3 of Rho guanine nucleotide exchange factor 1
4240	Q61210-4	2	3	4.3	856	95823	6	Isoform 4 of Rho guanine nucleotide exchange factor 1
4240	Q61210-2	2	3	4	919	102733	5.6	Isoform 2 of Rho guanine nucleotide exchange factor 1
4240	Q61210	2	3	4	920	102804	5.6	Rho guanine nucleotide exchange factor 1
4240	Q6KAM6	2	3	3.9	939	104635	5.6	MFLJ00369 protein (Fragment)
4240	E9PUF7	2	3	3.8	976	108693	6.1	Uncharacterized protein

Table S1. Proteins identified in the OVCAR-3 tumor supernatant by two or more unique peptides.

Protein or Protein Group	Accession Number	Unique Peptide Count	Spectrum Count	Sequence Coverage (%)	Length	MolWt	pI	Descriptive Name
4240	Q61210-5	2	3	3.8	979	109249	6.3	Isoform 5 of Rho guanine nucleotide exchange factor 1
4241	Q91X51	2	2	3.8	446	46882	4.7	Golgi reassembly-stacking protein 1
4242	Q8K2H0	2	27	3.6	753	84060	6.6	LIM domain and actin binding 1
4242	Q9ERG0	2	27	3.6	753	84090	6.6	LIM domain and actin-binding protein 1
4243	Q922F6	2	6	3.5	491	56940	5.1	Putative uncharacterized protein (Fragment)
4244	E9Q9W5	2	2	3	667	75289	5.6	Uncharacterized protein
4244	Q99JX6	2	2	3	667	75261	5.5	Anxa6 protein
4244	Q8BSS4	2	2	3	673	75885	5.5	Putative uncharacterized protein
4244	Q3UI56	2	2	3	673	75912	5.5	Putative uncharacterized protein
4244	Q3UDK4	2	2	3	673	75983	5.6	Putative uncharacterized protein
4244	Q3TUI1	2	2	3	667	75273	5.6	Putative uncharacterized protein
4244	P14824	2	2	3	673	75886	5.5	Annexin A6
4245	Q8JZT6	2	2	3	706	78777	5.9	Adducin 3
4245	Q9QYB5	2	2	3	706	78763	5.9	Gamma-adducin
4246	O54782	2	2	2.4	1018	115609	7.4	Epididymis-specific alpha-mannosidase
4246	Q3TND6	2	2	2.4	1018	115549	7.4	Putative uncharacterized protein
4247	Q9D620	2	7	3.1	645	70698	9.5	Rab11 family-interacting protein 1
4247	Q05A58	2	7	3.1	645	70684	9.5	MCG2247
4247	E9Q8L9	2	7	1.7	1166	125025	5.8	Uncharacterized protein
4248	P97789	2	2	1.6	1719	194306	7.5	5'-3' exoribonuclease 1
4248	UPI000058C305	2	2	1.6	1723	194756	7.6	5''-3'' exoribonuclease 1
4248	P97789-3	2	2	1.6	1687	191414	7.4	Isoform 3 of 5'-3' exoribonuclease 1
4248	P97789-2	2	2	1.6	1706	192976	7.5	Isoform 2 of 5'-3' exoribonuclease 1
4249	Q6P5H2	2	3	1.5	1864	207122	4.3	Nestin
4249	Q6P5H2-2	2	3	1.5	1820	201768	4.3	Isoform 2 of Nestin
4250	Q3U962	2	2	1.4	1497	145018	6.7	Collagen alpha-2(V) chain
4251	A2AVA0	2	4	1.3	3567	387458	5.6	Sushi, von Willebrand factor type A, EGF and pentraxin domain-containing protein 1
4252	E9PZM4	2	2	1.3	1827	210802	8.1	Uncharacterized protein
4253	A2AA80	2	15	1.2	1845	208267	5.5	Ciliary rootlet coiled-coil, rootletin
4253	Q8CJ40-2	2	15	1.2	1845	208237	5.5	Isoform 2 of Rootletin
4253	Q8CJ40-3	2	15	1.1	1988	224425	5.6	Isoform 3 of Rootletin
4253	Q8CJ40	2	15	1.1	2009	226912	5.6	Rootletin
4253	A2AA81	2	15	1.1	2009	226942	5.6	Ciliary rootlet coiled-coil, rootletin
4254	P35969	2	4	1.2	1333	149876	8.3	Vascular endothelial growth factor receptor 1
4255	Q3UH53	2	4	1.2	2193	240303	6.8	Protein sidekick-1
4256	B2RQQ5	2	2	0.9	2464	270304	4.8	Microtubule-associated protein 1B
4256	P14873	2	2	0.9	2464	270409	4.8	Microtubule-associated protein 1B
4256	E9QM11	2	2	0.9	2464	270252	4.8	Uncharacterized protein
4257	E9PV60	2	2	0.8	3024	337363	6.4	Uncharacterized protein
4257	E9Q2M9	2	2	0.8	3149	350926	6.3	Uncharacterized protein
4258	E9QPU1	2	2	0.6	2816	309471	5.5	Uncharacterized protein
4258	Q8CIZ8	2	2	0.6	2813	309266	5.5	von Willebrand factor
4258	Q2I0J8	2	2	0.6	2813	309141	5.5	VWF
4258	Q2I0J7	2	2	0.6	2813	309100	5.5	VWF
4259	P15531	2	40	89.5	152	17149	6.2	Nucleoside diphosphate kinase A
4260	Q0VGL1	2	18	61.6	99	10741	6.5	UPF0539 protein C7orf59
4261	Q8IUE6	2	12	52.3	130	13995	10.9	Histone H2A type 2-B
4262	Q15786	2	8	50.2	430	47085	4.8	Testis calpastatin
4263	O95989	2	7	45.3	172	19471	6.4	Diphosphoinositol polyphosphate phosphohydrolase 1
4264	Q8N3F0	2	4	41.2	131	14925	4.2	UPF0452 protein C7orf41
4265	Q8IYN2	2	5	41	117	13616	5.4	Transcription elongation factor A protein-like 8
4266	Q6PIK3	2	4	38	121	13536	9.2	HCG1995540, isoform CRA_b (Hcg2044074, isoform cra_d)
4267	Q8N6N7	2	5	36.4	88	9790	6.7	Acyl-CoA-binding domain-containing protein 7
4267	UPI0000D60F0F	2	5	36.4	88	9758	6.7	Acyl-CoA-binding domain-containing protein 7.
4268	Q9UHA2	2	2	36.4	77	8835	5.9	SS18-like protein 2
4269	P15259	2	2	33.2	253	28766	8.9	Phosphoglycerate mutase 2
4270	P34931	2	5	33.1	641	70375	6	Heat shock 70 kDa protein 1-like
4271	Q86WV5	2	2	32.8	122	13725	7.9	CST complex subunit TEN1
4272	Q96FH0	2	2	32.8	119	13403	5.8	UPF0402 protein
4273	D6RC73	2	16	32.5	80	9258	10.5	Chemokine (C-C motif) ligand 28, isoform CRA_b
4273	Q9NRJ3	2	16	20.5	127	14280	10.2	C-C motif chemokine 28
4274	Q68D61	2	3	43.6	94	10718	4.4	MCFD2 protein
4274	E9PD95	2	3	38	108	12337	4.5	Uncharacterized protein
4274	B4DF17	2	3	32.3	127	14404	4.9	cDNA FLJ59187, highly similar to Multiple coagulation factor deficiency protein 2 log
4274	Q8NI22	2	3	28.1	146	16390	4.6	Multiple coagulation factor deficiency protein 2
4275	B3KNS4	2	5	32.1	109	11601	7.8	HCG2043597, isoform CRA_a
4275	Q6ZRD3	2	5	22.3	157	17332	5.2	cDNA FLJ44718 fis, clone BRACE3022051
4276	Q9NSU9	2	3	31.2	375	41075	5.1	Putative uncharacterized protein DKFZp434G0719 (Fragment)
4277	C9J2T4	2	3	31.1	148	16903	5.4	Uncharacterized protein
4277	UPI00015E049A	2	3	31.1	148	16881	6.1	UPI00015E049A UniRef100 entry
4277	O75575	2	3	31.1	148	16871	5.4	DNA-directed RNA polymerase III subunit RPC9
4278	Q96GX2	2	5	30.9	97	10771	4.4	Putative ataxin-7-like protein 3B
4279	UPI0001B79342	2	2	31.2	112	13101	5.1	NADH dehydrogenase [ubiquinone] 1 alpha subcomplex subunit 5 (EC 1.6.5.3) (EC 1.6.99.3) (NADH-ubiquinone oxidoreductase 13 kDa-B subunit) (Complex I-13kD-B) (CI-13kD-B) (Complex I subunit B13).
4279	Q16718	2	2	30.2	116	13459	6	NADH dehydrogenase [ubiquinone] 1 alpha subcomplex subunit 5
4279	Q5H9R2	2	2	30.2	116	13563	5.6	Putative uncharacterized protein DKFZp781K1356
4280	B3KY81	2	34	29.9	1647	182990	8	cDNA FLJ46662 fis, clone TRACH3006800, highly similar to sapiens mucin 16, cell surface associated (MUC16), mRNA
4281	P33764	2	5	29.7	101	11713	4.8	Protein S100-A3
4282	Q8WY91-2	2	10	33.9	165	18627	7	Isoform 2 of THAP domain-containing protein 4
4282	B5MCC0	2	10	29.2	192	21490	7.2	Uncharacterized protein
4282	Q8WY91	2	10	9.7	577	62890	9.3	THAP domain-containing protein 4
4283	Q9BRT3	2	4	28.7	115	12403	4.4	Protein C17orf37
4284	A8MZ71	2	2	27.7	83	9388	9.4	Uncharacterized protein
4284	Q496I0	2	2	27.7	83	9426	9.6	COX7A2 protein
4284	P14406	2	2	27.7	83	9396	9.7	Cytochrome c oxidase subunit 7A2, mitochondrial
4284	D6RIE3	2	2	25.3	91	10264	9.7	Uncharacterized protein
4284	D6RGV5	2	2	22.3	103	11511	9.9	Uncharacterized protein
4285	Q9P021	2	3	27.7	101	11216	9.5	Cysteine-rich PDZ-binding protein
4286	A6NDJ8	2	3	27.6	181	20210	6.5	Putative Rab-43-like protein ENSP00000330714
4286	Q86YS6	2	3	23.6	212	23339	5.7	Ras-related protein Rab-43
4287	Q53XZ0	2	3	27.2	125	13938	7.9	Interferon induced transmembrane protein 1 (9-27)
4288	P09341	2	3	27.1	107	11301	10.4	Growth-regulated alpha protein
4289	Q53S33	2	3	26.2	107	12114	9.6	BolA-like protein 3
4290	Q9NPJ8	2	2	26.1	142	16228	5.5	NTF2-related export protein 2
4290	Q9NPJ8-2	2	2	19.7	188	21050	7.3	Isoform 2 of NTF2-related export protein 2
4291	Q9NZ45	2	3	25.9	108	12199	9.1	CDGSH iron-sulfur domain-containing protein 1
4292	Q14508-2	2	17	43.8	73	8120	7.5	Isoform 2 of WAP four-disulfide core domain protein 2
4292	Q14508-3	2	17	42.1	76	8108	7.8	Isoform 3 of WAP four-disulfide core domain protein 2

Table S1. Proteins identified in the OVCAR-3 tumor supernatant by two or more unique peptides.

Protein or Protein Group	Accession Number	Unique Peptide Count	Spectrum Count	Sequence Coverage (%)	Length	MolWt	pI	Descriptive Name
4292	Q14508	2	17	25.8	124	12993	4.8	WAP four-disulfide core domain protein 2
4293	Q9BTM9	2	5	25.7	101	11380	4.7	Ubiquitin-related modifier 1 log
4293	Q9BTM9-2	2	5	17.8	146	15815	5.3	Isoform 2 of Ubiquitin-related modifier 1 log
4294	O00488	2	5	25.4	134	15199	9.8	Zinc finger protein 593
4295	A8K0V8	2	2	25.2	218	25705	7.4	cDNA FLJ78039, highly similar to sapiens glutathione S-transferase M5, mRNA (cDNA clone MGC:64821 IMAGE:6193461)
4296	P37840-2	2	2	25.9	112	11372	8.3	Isoform 2-4 of Alpha-synuclein
4296	E7EPV7	2	2	25.2	115	11777	9.2	Uncharacterized protein
4296	P37840	2	2	20.7	140	14460	4.7	Alpha-synuclein
4297	Q9UL45	2	4	24.4	172	19744	6.4	Pallidin
4298	P40305	2	3	24.4	119	11268	11.1	Interferon alpha-inducible protein 27, mitochondrial
4298	A8K0H0	2	3	23.8	122	11542	11.1	Interferon, alpha-inducible protein 27, isoform CRA_a
4298	E7EQA7	2	3	23.8	122	11540	11.1	Uncharacterized protein
4298	C9JX49	2	3	23.8	122	11542	10.4	Uncharacterized protein
4299	Q1M185	2	2	40.7	86	9273	12	HMGA2d'
4299	Q1M188	2	2	38.9	90	9742	11.6	HMGA2c
4299	Q1M186	2	2	38	92	10063	11.9	HMGA2d
4299	Q1M187	2	2	34	103	10845	11.8	HMGA2c'
4299	Q1M182	2	2	33	106	11456	12	HMGA2f
4299	P52926	2	2	32.1	109	11832	10.6	High mobility group protein HMGI-C
4299	E7EWA2	2	2	23.8	147	16284	11.6	Uncharacterized protein
4299	Q1M183	2	2	23.8	147	16324	11.5	HMGA2e
4300	Q8WUR7	2	2	23.8	126	13322	9.4	UPF0235 protein C15orf40
4301	UPI000059D7E8	2	8	24.4	127	14362	6.7	Uncharacterized protein C20orf27.
4301	E9PAL2	2	8	23.3	133	14893	6.8	Uncharacterized protein
4301	Q9GZN8	2	8	17.8	174	19291	6.8	UPF0687 protein C20orf27
4301	Q9GZN8-2	2	8	15.6	199	21645	7.4	Isoform 2 of UPF0687 protein C20orf27
4302	Q15388	2	9	22.8	145	16298	8.6	Mitochondrial import receptor subunit TOM20 log
4303	Q9H4X1-2	2	2	26.5	117	12924	4.8	Isoform 2 of Response gene to complement 32 protein
4303	Q9H4X1	2	2	22.6	137	14559	4.8	Response gene to complement 32 protein
4304	Q9H204	2	2	22.5	178	19520	5.6	Mediator of RNA polymerase II transcription subunit 28
4305	Q96EL3	2	3	22.3	112	12107	8.8	39S ribosomal protein L53, mitochondrial
4306	P29992	2	7	22	359	42123	5.7	Guanine nucleotide-binding protein subunit alpha-11
4307	E9PIQ7	2	2	21.2	151	17167	5.4	Uncharacterized protein
4307	O00165-5	2	2	13.9	231	26100	5.2	Isoform 5 of HCLS1-associated protein X-1
4307	O00165	2	2	11.5	279	31621	4.9	HCLS1-associated protein X-1
4307	O00165-2	2	2	11.1	287	32418	5	Isoform 2 of HCLS1-associated protein X-1
4308	P51970	2	3	20.9	172	20105	7.6	NADH dehydrogenase [ubiquinone] 1 alpha subcomplex subunit 8
4309	Q12962	2	3	20.6	218	21711	6.5	Transcription initiation factor TFIID subunit 10
4310	Q6F3H0	2	2	21.6	273	31684	6.7	MHC class I antigen (Fragment)
4310	Q4VYE6	2	2	21.6	273	31650	6.6	MHC class I antigen (Fragment)
4310	D5H3W2	2	2	21.6	273	31574	5.5	MHC class I antigen (Fragment)
4310	D2KZ35	2	2	21.6	273	31629	6.5	MHC class I antigen (Fragment)
4310	A7Y1U3	2	2	20.3	291	33437	5.8	MHC class I antigen (Fragment)
4310	UPI00006C0B15	2	2	19.7	299	34130	6.2	PREDICTED: HLA class I histocompatibility antigen, A-30 alpha chain-like isoform 11
4310	Q52YL7	2	2	16.2	365	40950	7	MHC class I antigen
4310	D5H3T1	2	2	16.2	365	40964	7	MHC class I antigen
4310	UPI00006C0B09	2	2	15.9	371	41348	6.7	PREDICTED: HLA class I histocompatibility antigen, A-30 alpha chain-like isoform 3
4311	Q86WB4	2	2	29.9	117	13773	9.7	Neuromedin U (Fragment)
4311	P48645	2	2	20.1	174	19741	8.9	Neuromedin-U
4312	Q9H867	2	3	20.1	229	25807	4.9	Uncharacterized protein C14orf138
4313	A8K2P7	2	2	20	205	23545	5.3	cDNA FLJ77652, highly similar to sapiens START domain containing 4, sterol regulated (STARD4), mRNA
4313	Q96DR4	2	2	20	205	23517	5.3	StAR-related lipid transfer protein 4
4314	B4DKK1	2	2	19.8	162	18224	5.4	cDNA FLJ57120, highly similar to UBX domain-containing protein 6
4314	O00124-3	2	2	13.7	233	26457	6.8	Isoform 3 of UBX domain-containing protein 8
4314	O00124	2	2	11.9	270	30541	7.8	UBX domain-containing protein 8
4315	Q9BQ49	2	2	26.7	75	8631	8.1	UPF0608 protein C19orf42
4315	B7WNH4	2	2	19.4	103	11800	8.8	Uncharacterized protein
4316	Q8IVT1	2	5	18.9	132	14639	6	PGPEP1 protein
4316	Q9NXJ5	2	5	12	209	23138	5.9	Pyroglutamyl-peptidase 1
4317	B4DV65	2	2	18.6	156	17656	8.7	cDNA FLJ60902, highly similar to Ubiquitin carboxyl-terminal hydrolase 3 (EC 3.1.2.15)
4317	Q498Y2	2	2	7.4	393	44351	8.2	USP3 protein (Fragment)
4317	Q6JHV3	2	2	5.8	498	56649	8.3	Ubiquitin carboxyl-terminal hydrolase
4317	Q9Y6I4	2	2	5.6	520	58897	8.2	Ubiquitin carboxyl-terminal hydrolase 3
4318	O14582	2	4	18.6	140	16445	6.5	Trafficking protein particle complex subunit 2
4318	O14582-2	2	4	17.7	147	17260	6.7	Isoform 2 of Trafficking protein particle complex subunit 2
4319	P10606	2	2	18.6	129	13696	8.8	Cytochrome c oxidase subunit 5B, mitochondrial
4319	Q6FHM4	2	2	18.6	129	13705	8.8	COX5B protein
4320	Q9H633-3	2	2	20.8	144	16544	10.1	Isoform 3 of Ribonuclease P protein subunit p21
4320	Q5JPM0	2	2	20.8	144	16553	10.1	Ribonuclease P 21kDa subunit
4320	Q9H633	2	2	19.5	154	17570	9.5	Ribonuclease P protein subunit p21
4320	B0S836	2	2	18.5	162	18366	9.8	Ribonuclease P 21kDa subunit
4320	Q5SU41	2	2	18.5	162	18366	9.6	Ribonuclease P 21kDa subunit
4320	Q5JPM3	2	2	18.5	162	18375	9.6	Ribonuclease P 21kDa subunit
4321	P01034	2	14	18.5	146	15799	8.8	Cystatin-C
4322	D5KL87	2	7	33.3	45	5187	9.7	p53 (Fragment)
4322	B4XAL0	2	7	33.3	45	5171	9.7	p53 (Fragment)
4322	B5AKF7	2	7	32.6	46	5162	8.4	Mutant p53 tumor suppressor (Fragment)
4322	A4GWB4	2	7	18.3	82	9250	8.1	Cellular tumor antigen p53 (Fragment)
4322	Q53GA5	2	7	9.5	158	17656	9.3	Cellular tumor antigen p53 (Fragment)
4322	E7ESS1	2	7	7.5	199	22545	8.1	Cellular tumor antigen p53
4322	P04637-9	2	7	7	214	24401	8	Isoform 9 of Cellular tumor antigen p53
4322	P04637-7	2	7	5.7	261	29553	8.7	Isoform 7 of Cellular tumor antigen p53
4322	P04637-5	2	7	5	302	33493	6.8	Isoform 5 of Cellular tumor antigen p53
4322	P04637-6	2	7	4.9	307	34168	7	Isoform 6 of Cellular tumor antigen p53
4322	Q1MSW8	2	7	4.4	343	37993	6.6	Cellular tumor antigen p53 (Fragment)
4322	P04637-2	2	7	4.4	341	37826	5.8	Isoform 2 of Cellular tumor antigen p53
4322	P04637-3	2	7	4.3	346	38501	6	Isoform 3 of Cellular tumor antigen p53
4322	E7ERG1	2	7	4.1	368	41293	7.1	Cellular tumor antigen p53
4322	B4DMH2	2	7	4.1	368	41236	7.1	Cellular tumor antigen p53
4322	Q1MSX0	2	7	3.9	382	42627	6.7	Cellular tumor antigen p53 (Fragment)
4322	B4DNI2	2	7	3.9	383	42808	6.8	Cellular tumor antigen p53
4322	Q5U0E4	2	7	3.8	393	43730	6.9	Cellular tumor antigen p53
4322	Q2XSC7	2	7	3.8	393	43622	6.6	Cellular tumor antigen p53
4322	P04637	2	7	3.8	393	43653	6.8	Cellular tumor antigen p53
4322	E5RMA8	2	7	3.8	393	43669	6.8	Cellular tumor antigen p53
4322	B6E4X6	2	7	3.8	393	43754	6.9	Cellular tumor antigen p53

Table S1. Proteins identified in the OVCAR-3 tumor supernatant by two or more unique peptides.

Protein or Protein Group	Accession Number	Unique Peptide Count	Spectrum Count	Sequence Coverage (%)	Length	MolWt	pI	Descriptive Name
4323	P81605	2	3	20	110	11284	6.5	Dermcidin
4323	A5JHP3	2	3	18.2	121	12414	8	Dermcidin isoform 2
4324	Q8WVK7	2	2	18.2	121	14188	7.3	Spindle and kinetochore-associated protein 2
4325	Q14620	2	6	20.4	137	15177	9.3	Insulin-like growth factor 1 (Somatomedin C), isoform CRA_c
4325	E9PD02	2	6	17.7	158	17762	9.3	Uncharacterized protein
4325	Q59GC5	2	6	15.2	184	20618	8.9	Insulin-like growth factor 1 (Somatomedin C); insulin-like growth factor 1 (Somatomedia C) variant (Fragment)
4325	P05019	2	6	14.4	195	21841	9.7	Insulin-like growth factor I
4326	B2R6X5	2	49	17.4	643	70958	6.2	cDNA, FLJ93166, highly similar to sapiens heat shock 70kDa protein 6 (HSP70B') (HSPA6), mRNA
4326	Q53FC7	2	49	17.4	643	71004	6.1	Heat shock 70kDa protein 6 (HSP70B') variant (Fragment)
4326	P17066	2	49	17.4	643	71028	6.1	Heat shock 70 kDa protein 6
4326	B3KSM6	2	49	17.4	643	70972	6.1	cDNA FLJ36606 fis, clone TRACH2015654, highly similar to HEAT SHOCK 70 kDa PROTEIN 6
4327	Q6QNY0	2	3	17.3	202	21256	5.1	Biogenesis of lysosome-related organelles complex 1 subunit 3
4328	UPI0000D4D860	2	4	22.8	145	15156	8.6	claudin-7 isoform 2
4328	C9JC38	2	4	17	194	20729	8.4	Uncharacterized protein
4328	O95471	2	4	15.6	211	22418	8.6	Claudin-7
4329	Q8TAC2	2	2	17	188	20756	7.4	Josephin-2
4330	Q53HB4	2	3	16.2	160	18995	5.3	M-phase phosphoprotein 6 variant (Fragment)
4330	Q99547	2	3	16.2	160	19024	5.2	M-phase phosphoprotein 6
4331	Q5T6T5	2	3	18.4	125	14802	5.3	Progestagen-associated endometrial protein (Fragment)
4331	UPI000059DA92	2	3	18.3	126	14915	5.3	UPI000059DA92 UniRef100 entry
4331	Q5T6T0	2	3	17.7	130	14952	7.9	Progestagen-associated endometrial protein (Fragment)
4331	A6XNE0	2	3	16.1	143	16361	7.4	Glycodelin
4331	E9PH67	2	3	15.9	145	16453	7.8	Uncharacterized protein
4331	B4E3C0	2	3	14.6	158	18279	5.4	cDNA FLJ52183, highly similar to Glycodelin
4331	B2R4F9	2	3	14.2	162	18884	5.9	cDNA, FLJ92074, highly similar to sapiens progestagen-associated endometrial protein (placentalprotein 14, pregnancy-associated endometrial alpha-2-globulin,alpha uterine protein) (PAEP), mRNA
4331	P09466	2	3	12.8	180	20624	5.6	Glycodelin
4332	B4DZZ0	2	5	18.2	165	19206	9.8	cDNA FLJ52128, highly similar to PRA1 family protein 3
4332	E9PER8	2	5	18.1	166	19263	9.8	Uncharacterized protein
4332	B3KQB4	2	5	16	188	21555	9.8	cDNA FLJ90131 fis, clone HEMBB1000447, highly similar to PRA1 family protein 3
4332	O75915	2	5	16	188	21615	9.8	PRA1 family protein 3
4333	A4UCU0	2	3	15.9	182	19802	4.8	DEAD box polypeptide 47 isoform 1 variant (Fragment)
4333	UPI00002371B1	2	3	7.1	406	45169	9.2	probable ATP-dependent RNA helicase DDX47 isoform 2
4333	Q53GJ1	2	3	6.5	448	49882	9.2	DEAD (Asp-Glu-Ala-Asp) box polypeptide 47 isoform 1 variant (Fragment)
4333	Q9H4E3	2	3	6.4	455	50635	8.9	DEAD box protein
4333	Q9H0S4	2	3	6.4	455	50647	9.1	Probable ATP-dependent RNA helicase DDX47
4334	B4DUV1	2	6	17.6	641	70155	5.3	cDNA FLJ53207, highly similar to sapiens fibulin 1 (FBLN1), transcript variant C, mRNA
4334	UPI000019A000	2	6	16.5	683	74462	5.3	fibulin-1 isoform C precursor
4334	P23142-4	2	6	16.5	683	74434	5.2	Isoform C of Fibulin-1
4334	B1AHL2	2	6	15.7	721	78329	5.4	Fibulin 1
4335	B4DS97	2	4	15.7	134	14946	9	cDNA FLJ57579, highly similar to Dynactin subunit 5
4335	E7EWW8	2	4	15.7	134	14916	9	Uncharacterized protein
4335	UPI00001FF0B1	2	4	13.8	152	16633	8.4	dynactin subunit 5 isoform 3
4335	Q9BTE1	2	4	11.5	182	20127	8	Dynactin subunit 5
4336	Q5EG05-2	2	2	32	97	10737	5.2	Isoform 2 of Caspase recruitment domain-containing protein 16
4336	Q5EG05	2	2	15.7	197	22625	8.4	Caspase recruitment domain-containing protein 16
4337	Q9UI14	2	2	15.7	185	20648	7.3	Prenylated Rab acceptor protein 1
4338	Q9UKR5	2	2	15.7	140	15864	9.8	Probable ergosterol biosynthetic protein 28
4339	Q3KRA9	2	2	15.5	238	26483	8.8	Probable alpha-ketoglutarate-dependent dioxygenase ABH6
4340	Q9NSY2	2	2	15.5	213	23794	6.7	StAR-related lipid transfer protein 5
4341	Q9NUS5	2	2	15.5	200	22522	6.9	Protein C20orf29
4342	B3KVZ3	2	4	15.4	228	26239	4.9	Centromere protein H, isoform CRA_b
4342	Q9H3R5	2	4	14.2	247	28481	5.3	Centromere protein H
4343	C9J4K0	2	3	18.4	190	21356	9.7	Uncharacterized protein
4343	B4E2G9	2	3	15.4	228	25675	10.1	cDNA FLJ55159
4343	Q9BVC5	2	3	15.1	232	25858	9.7	Ashwin
4344	O75380	2	3	21	124	13712	8.3	NADH dehydrogenase [ubiquinone] iron-sulfur protein 6, mitochondrial
4344	D6RBT3	2	3	15.1	172	18752	8.7	Uncharacterized protein
4345	Q9NYG6	2	2	15.7	198	22099	7	Cyclin-dependent kinase inhibitor p27kip1
4345	P46527	2	2	15.7	198	22073	7	Cyclin-dependent kinase inhibitor 1B
4345	E7ES52	2	2	15.1	205	23183	6.6	Uncharacterized protein
4346	Q9H7X7	2	2	15.1	185	20835	5.1	Rab-like protein 5
4347	Q96B54	2	4	14.9	188	20481	4.2	Zinc finger protein 428
4348	C9JNG8	2	8	14.8	183	20699	7.9	Uncharacterized protein
4348	Q8NI32	2	8	14.8	183	20656	7.8	Ly6/PLAUR domain-containing protein 6B
4348	Q8NI32-2	2	8	13	207	23340	7.9	Isoform 2 of Ly6/PLAUR domain-containing protein 6B
4349	Q96G53	2	2	14.8	237	26001	5.6	SLC27A4 protein
4349	A6NIF3	2	2	14.7	238	26169	5.9	Uncharacterized protein
4349	Q9BTA1	2	2	13.6	258	28826	5.6	Putative uncharacterized protein (Fragment)
4349	B3KNH1	2	2	6.8	511	57195	7	cDNA FLJ14598 fis, clone NT2RM4002558, highly similar to Long-chain fatty acid transport protein 4 (EC 6.2.1.-)
4349	Q6P1M0	2	2	5.4	643	72064	8.5	Long-chain fatty acid transport protein 4
4350	O95832	2	6	14.7	211	22744	8.1	Claudin-1
4350	Q7Z4X9	2	6	14.7	211	22721	8.1	Claudin-2
4351	Q86YL5	2	2	15.7	185	20403	6	Uncharacterized protein C8orf42
4351	B6VF03	2	2	14.6	198	22285	8.2	TDRP2
4352	B7Z2P6	2	2	14.6	751	84779	5.1	cDNA FLJ55296, highly similar to sapiens WD repeat domain 42A (WDR42A), mRNA
4353	A8K0H3	2	26	14.5	159	17780	11.7	cDNA FLJ78093, highly similar to sapiens ribosomal protein L29 (RPL29), mRNA
4353	P47914	2	26	14.5	159	17752	11.7	60S ribosomal protein L29
4353	Q6IPI1	2	26	14.3	161	17951	11.7	Ribosomal protein L29
4354	A6NGX6	2	2	14.4	195	22638	4.6	Uncharacterized protein
4354	Q9BT43	2	2	12.8	218	25334	4.5	DNA-directed RNA polymerase III subunit RPC7-like
4355	Q5T6J7	2	2	14.4	187	20578	6.2	Probable gluconokinase
4355	Q5T6J7-2	2	2	11.7	230	25297	8.5	Isoform 2 of Probable gluconokinase
4356	B4DJG2	2	3	14.2	162	18489	9.6	cDNA FLJ55079, highly similar to sapiens lectin, galactoside-binding, soluble, 8, transcript variant 4, mRNA
4356	E9PN19	2	3	7.9	290	32632	7.2	Uncharacterized protein
4356	Q96QS7	2	3	7.7	300	33739	8.8	Lymphocyte/NHL galectin-8 short isoform
4356	E7EQ99	2	3	7.7	300	33985	9.2	Uncharacterized protein
4356	O00214	2	3	7.3	317	35808	8.3	Galectin-8
4356	Q59E97	2	3	6.8	338	38200	8.9	Galectin-8 variant (Fragment)
4356	O00214-2	2	3	6.4	359	40397	9	Isoform 2 of Galectin-8

Table S1. Proteins identified in the OVCAR-3 tumor supernatant by two or more unique peptides.

Protein or Protein Group	Accession Number	Unique Peptide Count	Spectrum Count	Sequence Coverage (%)	Length	MolWt	pI	Descriptive Name
4356	C9JNF5	2	3	6.4	357	40355	8.4	Uncharacterized protein
4357	Q6ISL4	2	2	14.3	133	15473	5.1	RBP2 protein (Fragment)
4357	P50120	2	2	14.2	134	15707	5.4	Retinol-binding protein 2
4358	Q99714	2	2	14.2	261	26923	7.8	3-hydroxyacyl-CoA dehydrogenase type-2
4359	Q05D20	2	27	13.9	373	41613	11.6	THRAP3 protein (Fragment)
4359	Q7Z5U1	2	27	13.9	373	41600	11.5	THRAP3 protein (Fragment)
4359	Q6PJV4	2	27	13.9	375	41858	11.5	THRAP3 protein (Fragment)
4360	O94811	2	10	13.7	219	23694	9.4	Tubulin polymerization-promoting protein
4361	Q9NV56	2	2	13.7	204	22417	5.8	MRG-binding protein
4362	B4DSX4	2	2	13.6	242	27442	6.8	cDNA FLJ60870, highly similar to Methyltransferase-like protein 2 (EC 2.1.1.-)
4362	Q6P1Q9-2	2	2	10.5	313	36057	5.9	Isoform 2 of Methyltransferase-like protein 2B
4362	Q6P1Q9	2	2	8.7	378	43426	5.9	Methyltransferase-like protein 2B
4363	UPI0001BEF277	2	2	22.6	133	14773	6.7	UDP-N-acetylglucosamine transferase subunit ALG13 log isoform 4
4363	Q9NP73-2	2	2	18.2	165	18225	6.5	Isoform 2 of UDP-N-acetylglucosamine transferase subunit ALG13 log
4363	D6RE84	2	2	13.5	222	24293	7.8	Uncharacterized protein
4363	Q9NP73	2	2	2.6	1137	126056	6.7	UDP-N-acetylglucosamine transferase subunit ALG13 log
4364	Q9NSK7-3	2	6	17.8	107	11113	8.5	Isoform 3 of Uncharacterized protein C19orf12
4364	Q9NSK7	2	6	13.5	141	15007	5.9	Uncharacterized protein C19orf12
4365	Q8N4B8	2	8	15.9	195	22725	6.9	Golgi SNAP receptor complex member 2
4365	O14653-2	2	8	14.6	213	24584	8.5	Isoform B of Golgi SNAP receptor complex member 2
4365	O14653	2	8	14.6	212	24775	8.1	Golgi SNAP receptor complex member 2
4365	E7EQ34	2	8	13.4	232	26624	7.7	Uncharacterized protein
4366	Q9BQD3	2	4	13.6	176	19668	4.9	UPF0459 protein C19orf50
4366	Q7Z4E2	2	4	13.4	179	20149	5.8	MSTP096
4367	Q9H9U4	2	4	13.1	442	49368	5.7	cDNA FLJ12546 fis, clone NT2RM4000616, moderately similar to ACETYL-COENZYME A SYNTHETASE (EC 6.2.1.1)
4368	P32929-2	2	2	13.3	361	39505	6.9	Isoform 2 of Cystathionine gamma-lyase
4368	B4E1R2	2	2	12.9	373	41286	6.9	cDNA FLJ50357, highly similar to Cystathionine gamma-lyase (EC 4.4.1.1)
4368	E9PDV0	2	2	12.9	373	41260	6.9	Uncharacterized protein
4368	P32929	2	2	11.9	405	44508	6.7	Cystathionine gamma-lyase
4369	Q58FG1	2	3	12.9	418	47712	5.2	Putative heat shock protein HSP 90-alpha A4
4370	P50453	2	2	12.8	376	42404	5.9	Serpin B9
4371	Q9BVC3	2	2	12.7	393	44825	5.2	Sister chromatid cohesion protein DCC1
4372	Q9NXR1-2	2	5	13.1	335	37721	5.2	Isoform 2 of Nuclear distribution protein nudE log 1
4372	Q9NXR1	2	5	12.7	346	38808	5.3	Nuclear distribution protein nudE log 1
4373	UPI0001E8F293	2	3	18.8	186	20753	5.4	UPI0001E8F293 UniRef100 entry
4373	Q5T013-4	2	3	17.2	204	22616	5.8	Isoform 4 of Putative hydroxypyruvate isomerase
4373	Q5T015	2	3	15.3	229	25229	6.5	Hydroxypyruvate isomerase log (E. coli)
4373	Q5T013	2	3	12.6	277	30406	5.5	Putative hydroxypyruvate isomerase
4374	B4DXN3	2	2	12.4	137	15301	9.5	cDNA FLJ57657, highly similar to Charged multivesicular body protein 1a
4374	Q9HD42	2	2	8.7	196	21703	8.1	Charged multivesicular body protein 1a
4375	O00560-3	2	3	12.7	292	31762	7.5	Isoform 3 of Syntenin-1
4375	O00560	2	3	12.4	298	32444	7.5	Syntenin-1
4376	P51151	2	3	12.4	201	22838	5.5	Ras-related protein Rab-9A
4377	B1AHE1	2	2	12.5	256	28957	5.8	TRIO and F-actin binding protein (Fragment)
4377	B1AHD5	2	2	12.2	262	29540	6.9	TRIO and F-actin binding protein (Fragment)
4377	B1AHD8	2	2	12.2	262	29615	8.1	TRIO and F-actin binding protein (Fragment)
4377	B1AHD9	2	2	12.1	265	29582	8	TRIO and F-actin binding protein (Fragment)
4377	B1AHD6	2	2	8.6	372	41618	7.1	TRIO and F-actin binding protein
4377	UPI000045707F	2	2	7.4	431	47624	9.1	TRIO and F-actin-binding protein isoform 2
4377	E9PC58	2	2	4.9	652	74048	6.9	Uncharacterized protein
4377	Q9H2D6	2	2	1.4	2365	261373	8.5	TRIO and F-actin-binding protein
4378	Q5QPB1	2	2	12.3	211	22540	9.3	Zinc finger, AN1-type domain 5 (Fragment)
4378	O76080	2	2	12.2	213	23132	8.5	AN1-type zinc finger protein 5
4379	Q53S08	2	3	12.2	254	28242	6.7	Putative uncharacterized protein RAB6C
4379	Q9H0N0	2	3	12.2	254	28355	7.7	Ras-related protein Rab-6C
4380	Q6P1K2	2	2	12.2	205	23339	5.5	Polyamine-modulated factor 1
4380	Q6P1K2-2	2	2	12.1	207	22946	6.1	Isoform 2 of Polyamine-modulated factor 1
4380	UPI000046FD09	2	2	11.8	211	23769	7.3	PMF1-BGLAP protein isoform 2
4381	Q8IY26	2	4	12.2	295	32194	10	Presqualene diphosphate phosphatase
4382	Q92623	2	2	12.2	222	24379	9	Tetratricopeptide repeat protein 9A
4383	Q8NAV1	2	4	11.9	312	37477	10	Pre-mRNA-splicing factor 38A
4384	Q9H488	2	2	11.9	388	43956	8.5	GDP-fucose protein O-fucosyltransferase 1
4385	Q5VXM9	2	2	17.3	208	24158	9.5	Brix domain containing 1 (Fragment)
4385	Q5VXN0	2	2	16.8	214	24605	9.6	Brix domain containing 1 (Fragment)
4385	B4DWS5	2	2	14.3	251	28956	9.8	cDNA FLJ57639, highly similar to Brix domain-containing protein 1
4385	A8K800	2	2	11.8	306	35573	10	cDNA FLJ76924, highly similar to sapiens brix domain containing 1 (BXDC1), mRNA
4385	Q9H7B2	2	2	11.8	306	35583	10	Ribosome production factor 2 log
4386	Q8WV07	2	4	19	137	15354	5.8	Oral cancer-overexpressed protein 1
4386	B4DFA5	2	4	11.8	221	24768	7.2	cDNA FLJ58145, highly similar to Oral cancer overexpressed protein 1
4387	B4DL37	2	3	11.8	204	23364	8	Aldo-keto reductase family 1, member C3 (3-alpha hydroxysteroid dehydrogenase, type II), isoform CRA_c
4387	Q2XPP3	2	3	7.4	323	36768	8.1	Type II 3a-hydroxysteroid dehydrogenase variant
4387	P42330	2	3	7.4	323	36853	7.9	Aldo-keto reductase family 1 member C3
4388	Q96EY5-3	2	3	12.5	257	27050	9.4	Isoform 3 of Multivesicular body subunit 12A
4388	A8K0C2	2	3	11.7	273	28809	8.9	cDNA FLJ75950
4388	Q96EY5	2	3	11.7	273	28783	8.9	Multivesicular body subunit 12A
4388	E9PQA6	2	3	11.7	273	28703	9.6	Uncharacterized protein
4389	Q96IY1	2	3	11.7	281	32162	6.8	Kinetochore-associated protein NSL1 log
4390	Q8TBF4	2	2	11.5	217	24592	8.5	Zinc finger CCHC-type and RNA-binding motif-containing protein 1
4391	Q96CG3	2	2	11.4	184	21445	5.1	TRAF-interacting protein with FHA domain-containing protein A
4392	Q9BQ69	2	2	11.4	325	35505	9.5	MACRO domain-containing protein 1
4393	UPI0001E8F63E	2	3	13.3	225	25941	5.5	UPI0001E8F63E UniRef100 entry
4393	E5RH53	2	3	12.1	248	28634	8	Uncharacterized protein
4393	B4DNF5	2	3	11.1	271	30833	8.8	cDNA FLJ59260, highly similar to Protein FAM82B
4393	E7EVI2	2	3	11.1	271	30847	8.9	Uncharacterized protein
4393	B4DZW6	2	3	10.6	284	32365	8.9	Uncharacterized protein
4393	Q96DB5	2	3	9.6	314	35808	8.5	Regulator of microtubule dynamics protein 1
4394	E9PC70	2	3	10.7	242	26818	5.1	Uncharacterized protein
4394	Q7Z5N2	2	3	10.7	242	26788	5.1	Kangai 1
4394	Q6LET0	2	3	9.7	267	29644	5.2	KAI1 protein (Fragment)
4394	P27701	2	3	9.7	267	29626	5.2	CD82 antigen
4395	C9JU10	2	2	10.5	258	28659	7	Uncharacterized protein
4395	Q86YJ6-3	2	2	7	384	42718	6.8	Isoform 3 of Threonine synthase-like 2
4395	Q86YJ6-2	2	2	6.7	405	45037	6.5	Isoform 2 of Threonine synthase-like 2

Table S1. Proteins identified in the OVCAR-3 tumor supernatant by two or more unique peptides.

Protein or Protein Group	Accession Number	Unique Peptide Count	Spectrum Count	Sequence Coverage (%)	Length	MolWt	pI	Descriptive Name
4395	Q86YJ6-4	2	2	6.4	423	47428	6.2	Isoform 4 of Threonine synthase-like 2
4395	Q86YJ6	2	2	5.6	484	54116	6.5	Threonine synthase-like 2
4396	Q6IQ49-3	2	2	12.6	356	39367	5	Isoform 3 of UPF0667 protein C1orf55
4396	C9JIQ2	2	2	10.3	439	48528	6.2	Uncharacterized protein
4396	Q6IQ49-2	2	2	10.3	439	48505	6.1	Isoform 2 of UPF0667 protein C1orf55
4396	Q6IQ49	2	2	10	451	49742	6	UPF0667 protein C1orf55
4397	Q9UII2	2	6	10.4	106	12249	9.4	ATPase inhibitor, mitochondrial
4397	Q5JXL8	2	6	10.3	107	12405	9.6	Putative uncharacterized protein DKFZp564G0422
4398	B2RC46	2	2	10.2	226	25329	5.6	cDNA, FLJ95848
4398	Q9NRH1	2	2	10.2	226	25299	5.6	Uncharacterized protein C7orf36
4399	B3KM50	2	3	10.2	353	39145	6	cDNA FLJ10310 fis, clone NT2RM2000322, highly similar to sapiens KIAA0859 protein
4399	Q8N6R0-2	2	3	9.1	396	44563	5.6	Isoform 5 of Methyltransferase-like protein 13
4399	Q8N6R0-1	2	3	6.6	543	61128	6.1	Isoform 4 of Methyltransferase-like protein 13
4399	Q8N6R0-3	2	3	5.9	613	68689	6.7	Isoform 3 of Methyltransferase-like protein 13
4399	B3KNM8	2	3	5.9	613	68657	6.7	cDNA FLJ14980 fis, clone Y79AA1000181, highly similar to sapiens CGI-01 protein mRNA
4399	Q8N6R0-4	2	3	5.6	642	72261	7	Isoform 2 of Methyltransferase-like protein 13
4399	Q8N6R0	2	3	5.2	699	78768	6.7	Methyltransferase-like protein 13
4399	B4E2X3	2	3	5.2	698	78688	6.7	cDNA FLJ56024
4399	B3KNI0	2	3	5.2	699	78690	6.5	cDNA FLJ14646 fis, clone NT2RP2001883, highly similar to sapiens CGI-01 protein mRNA
4400	Q9P016-2	2	2	13.9	166	18812	9.3	Isoform 2 of Thymocyte nuclear protein 1
4400	Q9P016	2	2	10.2	225	25697	9.2	Thymocyte nuclear protein 1
4401	A8K883	2	5	10.1	524	59228	5	cDNA FLJ77590, highly similar to sapiens leucine rich repeat containing 1, mRNA
4401	Q9BTT6	2	5	10.1	524	59242	5	Leucine-rich repeat-containing protein 1
4402	Q96Q83	2	2	10.1	286	33375	8.4	Alpha-ketoglutarate-dependent dioxygenase alkB log 3
4403	E7ESK6	2	5	11.5	165	18388	6	Syndecan
4403	B7Z5H2	2	5	11	172	18914	4.8	Syndecan
4403	B4DQ56	2	5	9.9	192	21179	4.9	Syndecan
4403	P34741	2	5	9.5	201	22160	4.9	Syndecan-2
4404	Q5BJH7-2	2	2	10.2	283	31175	7.4	Isoform 2 of Protein YIF1B
4404	E9PB34	2	2	9.7	299	32825	8.6	Uncharacterized protein
4404	Q5BJH7-3	2	2	9.3	311	33965	8.8	Isoform 3 of Protein YIF1B
4404	Q5BJH7	2	2	9.2	314	34435	9.2	Protein YIF1B
4405	Q5T8V1	2	2	9.7	238	27809	7.8	Chromosome 9 open reading frame 41 (Fragment)
4405	Q8N4J0	2	2	5.6	409	47186	6.3	UPF0586 protein C9orf41
4406	Q86U90	2	2	9.7	279	29328	8.6	YrdC domain-containing protein, mitochondrial
4407	Q9H469	2	5	9.7	300	32998	7.4	F-box only protein 37
4408	A8MZA9	2	5	9.6	270	30387	4.4	Uncharacterized protein
4408	Q96EV8	2	5	7.4	351	39493	4.7	Dysbindin
4409	B4E2D2	2	4	13.7	263	30000	5.7	cDNA FLJ58874, highly similar to Caspase-4 (EC 3.4.22.-)
4409	B3KPZ9	2	4	9.5	377	43209	5.9	cDNA FLJ32532 fis, clone SMINT2000229, highly similar to CASPASE-4 (EC 3.4.22.-)
4409	P49662	2	4	9.5	377	43262	6	Caspase-4
4410	B7Z860	2	2	9.2	261	28348	5	cDNA FLJ54991
4410	Q8IWD4	2	2	8.6	279	30541	5.1	Coiled-coil domain-containing protein 117
4411	UPI0001881777	2	3	16.4	122	12508	10.5	UPI0001881777 UniRef100 entry
4411	UPI000198C5FF	2	3	14.3	140	15540	10.3	UPI000198C5FF UniRef100 entry
4411	Q6P582	2	3	12.7	158	16221	9.6	Mitotic-spindle organizing protein 2A
4411	Q6NZ67	2	3	12.7	158	16226	10.2	Mitotic-spindle organizing protein 2B
4411	B8ZZ87	2	3	9.2	218	22281	10.2	Uncharacterized protein
4412	Q6PID6	2	2	9.2	262	29411	5.4	Tetratricopeptide repeat protein 33
4413	B7Z5I6	2	2	10.2	557	61460	5.1	cDNA FLJ56516, highly similar to Nuclear pore complex protein Nup88
4413	B4DP20	2	2	9.1	626	71285	5.9	cDNA FLJ58372, highly similar to Nuclear pore complex protein Nup88
4413	Q99567	2	2	7.7	741	83542	5.7	Nuclear pore complex protein Nup88
4414	Q8TBZ6	2	2	9.1	339	39719	7.6	RNA (guanine-9-)-methyltransferase domain-containing protein 2
4415	O75792	2	3	9	299	33395	5.3	Ribonuclease H2 subunit A
4415	Q6UQL6	2	3	9	299	33425	5.2	Ribonuclease
4415	Q6IBG3	2	3	9	299	33353	5.3	Ribonuclease
4416	P10620	2	2	9	155	17599	9.4	Microsomal glutathione S-transferase 1
4416	Q6LET6	2	2	9	155	17571	9.4	MGST1 protein (Fragment)
4417	Q06265-3	2	4	10.7	355	39235	5.1	Isoform 3 of Exosome complex exonuclease RRP45
4417	Q06265-4	2	4	10.2	372	41089	5.1	Isoform 4 of Exosome complex exonuclease RRP45
4417	B4DXG8	2	4	9.7	392	43562	4.9	cDNA FLJ55260, highly similar to Exosome complex exonuclease RRP45 (EC 3.1.13.-)
4417	D6RIY6	2	4	9	423	46978	5.2	Exosome component 9, isoform CRA_f
4417	A5PLM5	2	4	8.7	439	48919	5.3	Exosome component 9
4417	Q06265	2	4	8.7	439	48949	5.3	Exosome complex exonuclease RRP45
4417	Q06265-2	2	4	8.3	456	50803	5.3	Isoform 2 of Exosome complex exonuclease RRP45
4418	D3YRV2	2	3	29.3	75	8493	6.3	p53-induced protein with RING-H2 variant D
4418	Q9Y3X7	2	3	21.4	103	12252	6.5	Putative uncharacterized protein DKFZp586C1620 (Fragment)
4418	UPI0001D3B499	2	3	19.8	111	12953	6.3	UPI0001D3B499 UniRef100 entry
4418	C7E543	2	3	12.3	179	20735	6.6	p53-induced protein with RING-H2 variant C
4418	Q2KN33	2	3	11.7	188	21697	6.5	Ring finger and CHY zinc finger domain containing 1 variant 2
4418	UPI000048C392	2	3	11	200	23155	6.6	RING finger and CHY zinc finger domain-containing protein 1 isoform 2
4418	E7ETW5	2	3	9.2	239	27676	6.7	Uncharacterized protein
4418	C7E542	2	3	8.7	252	28998	6.8	p53-induced protein with RING-H2 variant B
4418	Q96PM5-2	2	3	8.7	252	28983	6.9	Isoform 2 of RING finger and CHY zinc finger domain-containing protein 1
4418	Q96PM5	2	3	8.4	261	30110	6.7	RING finger and CHY zinc finger domain-containing protein 1
4419	D6RBR7	2	4	8.7	229	25833	8.3	Uncharacterized protein
4419	Q9Y3S2	2	4	6.2	320	36201	6.2	Zinc finger protein 330
4419	Q53EN0	2	4	6.2	320	36227	6.1	Zinc finger protein 330 variant (Fragment)
4420	Q7Z528	2	7	8.6	266	30425	5.2	E3-16
4420	Q9Y287	2	7	8.6	266	30338	5.1	Integral membrane protein 2B
4420	Q9NX12	2	7	8.6	266	30312	5.2	cDNA FLJ20496 fis, clone KAT08729
4420	Q96B24	2	7	8.6	266	30368	5.1	Integral membrane protein 2B
4421	Q9BYH9	2	3	60	55	6246	9.6	Squamous cell carcinoma antigen 1 (Fragment)
4421	O95740	2	3	32.4	102	11386	6.9	Squamous cell carcinoma antigen 2 (Fragment)
4421	C9JZ65	2	3	15.6	211	24374	6.6	Uncharacterized protein
4421	UPI0000D60EED	2	3	9.8	338	38837	6.2	Serpin B3 (Squamous cell carcinoma antigen 1) (SCCA-1) (Protein T4-A).
4421	P29508-2	2	3	9.8	338	38550	6.7	Isoform 2 of Serpin B3
4421	Q9BYF7	2	3	8.9	369	42287	6	SCCA2b
4421	A8K847	2	3	8.5	390	44853	6.6	cDNA FLJ75229, highly similar to sapiens serpin peptidase inhibitor, clade B (ovalbumin), member 4 (SERPINB4), mRNA
4421	Q86W05	2	3	8.5	390	44830	6.2	Squamous cell carcinoma antigen 2
4421	Q5K684	2	3	8.5	390	44770	6.6	SCCA1/SCCA2 fusion protein

Table S1. Proteins identified in the OVCAR-3 tumor supernatant by two or more unique peptides.

Protein or Protein Group	Accession Number	Unique Peptide Count	Spectrum Count	Sequence Coverage (%)	Length	MolWt	pI	Descriptive Name
4421	Q5K634	2	3	8.5	390	44649	6.4	SCCA2/SCCA1 fusion protein isoform 1
4421	Q5K633	2	3	8.5	390	44810	6.5	SCCA2/SCCA1 fusion protein isoform 2
4421	P48594	2	3	8.5	390	44854	6.2	Serpin B4
4421	P29508	2	3	8.5	390	44565	6.8	Serpin B3
4421	B3W5Y6	2	3	8.5	390	44579	6.8	Squamous cell carcinoma antigen-1 isoform SCCA-PD
4422	Q86TP1-3	2	6	12.2	271	30127	6.2	Isoform 3 of Protein prune log
4422	E7EQ15	2	6	8.5	386	42472	5.5	Uncharacterized protein
4422	Q86TP1-2	2	6	8.5	386	42456	5.5	Isoform 2 of Protein prune log
4422	Q86TP1	2	6	7.3	453	50200	5.5	Protein prune log
4423	Q9HBQ7	2	2	18.5	151	16839	4.6	Cathepsin L, isoform CRA_b
4423	A5PLM9	2	2	8.4	333	37533	5.4	Cathepsin L1
4423	P07711	2	2	8.4	333	37564	5.4	Cathepsin L1
4423	B3KQK4	2	2	8.4	333	37530	5.4	cDNA FLJ90619 fis, clone PLACE1002374, highly similar to Cathepsin L (EC 3.4.22.15)
4424	B3KMF7	2	4	8.4	534	61358	8.6	cDNA FLJ10863 fis, clone NT2RP4001575, highly similar to Vacuolar protein sorting protein 52
4424	B4DNI9	2	4	7.5	598	68496	8.4	cDNA FLJ54452, highly similar to Vacuolar protein sorting protein 52
4424	Q8N1B4	2	4	6.2	723	82221	6	Vacuolar protein sorting-associated protein 52 log
4425	O14828-2	2	6	9	321	35202	7.9	Isoform 2 of Secretory carrier-associated membrane protein 3
4425	O14828	2	6	8.4	347	38287	7.6	Secretory carrier-associated membrane protein 3
4425	Q6FHJ5	2	6	8.4	347	38243	7.6	SCAMP3 protein (Fragment)
4426	Q0VDF9	2	3	8.4	509	54795	5.6	Heat shock 70 kDa protein 14
4427	Q5JTV1	2	13	8.4	479	51234	6.3	Glucocorticoid modulatory element binding protein 2
4427	Q9UKD1	2	13	7.5	530	56421	5.6	Glucocorticoid modulatory element-binding protein 2
4428	Q5T750	2	2	8.4	250	26238	8	Skin-specific protein 32
4429	B1APR7	2	3	8.2	416	45549	5.8	Eyes absent log 3 (Drosophila)
4429	Q99504-2	2	3	7.6	447	48652	5.8	Isoform 2 of Eyes absent log 3
4429	B4DIR7	2	3	6.5	527	57827	5.1	cDNA FLJ50734, highly similar to Eyes absent log 3 (EC 3.1.3.48)
4429	Q8IVX7	2	3	6.3	536	58698	5	EYA3 protein
4429	Q99504	2	3	5.9	573	62663	5.2	Eyes absent log 3
4430	E9PC09	2	2	12.1	305	33564	5.9	Uncharacterized protein
4430	B4DJN0	2	2	8.2	451	50289	5.6	cDNA FLJ51641, highly similar to Nicastrin
4430	Q92542-2	2	2	5.4	689	76744	5.7	Isoform 2 of Nicastrin
4430	Q92542	2	2	5.2	709	78411	6	Nicastrin
4431	P02647	2	2	8.2	267	30778	5.8	Apolipoprotein A-I
4432	Q96TC7-2	2	17	9.7	341	38247	4.7	Isoform 2 of Regulator of microtubule dynamics protein 3
4432	B3KUI4	2	17	8.1	407	45338	4.8	cDNA FLJ39967 fis, clone SPLEN2027488, highly similar to sapiens family with sequence similarity 82, member C (FAM82C), mRNA
4432	Q96TC7	2	17	7	470	52118	5.1	Regulator of microtubule dynamics protein 3
4433	Q99557	2	4	32.6	86	9780	10.1	NF-IL6 (Fragment)
4433	Q9BSC0	2	4	20.4	137	14860	9.8	CEBPB protein (Fragment)
4433	P17676	2	4	8.1	345	36106	8.3	CCAAT/enhancer-binding protein beta
4434	Q5HYI8	2	3	8.1	236	26423	7.1	Rab-like protein 3
4435	Q6DD87	2	7	8.1	382	40428	8	Zinc finger protein 787
4436	B2RDT9	2	4	8	348	39384	5.1	cDNA, FLJ96765
4437	B3KSE0	2	2	7.9	316	35961	5.5	cDNA FLJ36069 fis, clone TESTI2019406, highly similar to HEME OXYGENASE 2 (EC 1.14.99.3)
4437	Q6IBP2	2	2	7.9	316	36015	5.4	HMOX2 protein
4437	Q6FHB5	2	2	7.9	316	36049	5.4	HMOX2 protein (Fragment)
4437	Q53HF1	2	2	7.9	316	35934	5.4	Heme oxygenase (Decyclizing) 2 variant (Fragment)
4437	P30519	2	2	7.9	316	36033	5.4	Heme oxygenase 2
4438	Q8NBN7	2	3	7.9	331	35932	8.1	Retinol dehydrogenase 13
4439	O94768	2	2	7.8	372	42344	5.3	Serine/threonine-protein kinase 17B
4439	Q6FG98	2	2	7.8	372	42245	5.2	STK17B protein
4439	Q6FG83	2	2	7.8	372	42291	5.2	STK17B protein (Fragment)
4440	Q9NUJ1	2	3	7.8	306	33933	8.6	Abhydrolase domain-containing protein 10, mitochondrial
4441	B4DMK2	2	2	7.7	312	35541	7.3	cDNA FLJ57398
4441	E7EMP6	2	2	7.7	312	35571	7.3	Uncharacterized protein
4441	Q96FZ2	2	2	6.8	354	40575	8.1	UPF0361 protein C3orf37
4442	E7EW99	2	3	18.9	201	23119	8.8	Uncharacterized protein
4442	B7Z7K8	2	3	7.7	493	55931	6.6	cDNA FLJ51143, highly similar to Melanoma antigen preferentially expressed in tumors
4442	B7ZAT0	2	3	7.7	493	55883	6.6	cDNA, FLJ79294, highly similar to Melanoma antigen preferentially expressed in tumors
4442	P78395	2	3	7.5	509	57890	6.9	Melanoma antigen preferentially expressed in tumors
4443	C9JFK9	2	3	7.7	325	34760	9.4	Uncharacterized protein
4443	Q53GY1	2	3	4.3	575	61604	7.4	BCL2-associated athanogene 3 variant (Fragment)
4443	O95817	2	3	4.3	575	61595	7	BAG family molecular chaperone regulator 3
4444	D3VVR8	2	2	10.1	218	25349	4.8	Ataxin 3 variant r (Fragment)
4444	D3VVN3	2	2	9.2	238	27335	4.8	Ataxin 3 variant at (Fragment)
4444	D3VVD9	2	2	8.3	265	30726	5.2	Ataxin 3 variant y (Fragment)
4444	D3VVB2	2	2	7.7	287	33279	5	Ataxin 3 variant h (Fragment)
4444	D3VVR4	2	2	7.6	289	33245	4.7	Ataxin 3 variant e (Fragment)
4444	D3VVE3	2	2	7.6	290	33693	5	Ataxin 3 variant h (Fragment)
4444	D3VVJ1	2	2	6.7	328	37943	4.9	Ataxin 3 variant ref (Fragment)
4444	D3VVJ2	2	2	6.6	335	38512	4.7	Ataxin 3 variant ref (Fragment)
4444	D3VVI8	2	2	6.6	334	38488	4.8	Ataxin 3 variant ref (Fragment)
4444	D3VVG7	2	2	6.6	335	38656	4.9	Ataxin 3 variant ref (Fragment)
4444	D3VVT6	2	2	6.5	336	38785	4.9	Ataxin 3 variant ref (Fragment)
4444	D3VVJ3	2	2	6.5	341	39497	4.8	Ataxin 3 variant ref (Fragment)
4444	D3VVD7	2	2	6.5	336	38812	4.9	Ataxin 3 variant ref (Fragment)
4444	D3VVB3	2	2	6.5	341	39443	4.9	Ataxin 3 variant ref (Fragment)
4444	D3VVN1	2	2	6.4	343	39681	4.9	Ataxin 3 variant ref (Fragment)
4444	D3VVN0	2	2	6.4	344	39795	4.9	Ataxin 3 variant ref (Fragment)
4444	D3VVS1	2	2	5.7	387	44960	4.7	Ataxin 3 variant ref (Fragment)
4444	D3VVR6	2	2	5.7	383	44649	4.9	Ataxin 3 variant ref (Fragment)
4444	D3VVK7	2	2	5.6	395	46347	4.9	Ataxin 3 variant ref (Fragment)
4445	B7Z4S8	2	4	8	410	47072	6.7	cDNA FLJ53066, highly similar to Legumain (EC 3.4.22.34)
4445	A8K669	2	4	7.6	433	49401	6.6	cDNA FLJ78452, highly similar to sapiens legumain (LGMN), transcript variant 2, mRNA
4445	Q99538	2	4	7.6	433	49411	6.6	Legumain
4445	Q6I9U9	2	4	7.6	433	49353	6.6	LGMN protein
4446	B4DLP1	2	3	7.6	355	38798	5.3	cDNA FLJ52560
4447	Q96IZ0	2	2	7.6	340	36568	5.4	PRKC apoptosis WT1 regulator protein
4448	B2RAZ3	2	2	7.3	427	47253	5.3	cDNA, FLJ95207, highly similar to sapiens interferon regulatory factor 3 (IRF3), mRNA
4448	Q14653	2	2	7.3	427	47219	5.3	Interferon regulatory factor 3
4448	Q96GL3	2	2	6.9	452	49120	6.5	IRF3 protein
4448	Q7Z5G6	2	2	6.9	452	49135	6.5	IRF3 protein
4449	Q6IPN0	2	3	7.3	343	36918	4.8	RTN4 protein
4449	Q7L7Q6	2	3	6.7	373	40318	4.8	RTN4

Table S1. Proteins identified in the OVCAR-3 tumor supernatant by two or more unique peptides.

Protein or Protein Group	Accession Number	Unique Peptide Count	Spectrum Count	Sequence Coverage (%)	Length	MolWt	pI	Descriptive Name
4449	Q9NQC3-5	2	3	6.4	392	42274	4.7	Isoform 5 of Reticulon-4
4449	Q9NQC3	2	3	2.1	1192	129931	4.5	Reticulon-4
4450	Q6PD74	2	4	7.3	315	34594	4.6	Alpha- and gamma-adaptin-binding protein p34
4451	E7EX40	2	8	7.2	501	54833	9	Uncharacterized protein
4451	Q4JIW2	2	8	7.2	501	54803	9	Rab11-family interacting protein 1D
4451	Q6WKZ4-1	2	8	5.9	612	65217	4.8	Isoform 3 of Rab11 family-interacting protein 1
4451	Q6WKZ4-3	2	8	5.5	649	70983	9.3	Isoform 2 of Rab11 family-interacting protein 1
4451	Q307T1	2	8	5.5	649	70953	9.3	RAB11-family interacting protein 1C
4451	UPI0000D624B1	2	8	2.8	1283	137167	5.4	rab11 family-interacting protein 1 isoform 3
4451	Q6WKZ4	2	8	2.8	1283	137195	5.5	Rab11 family-interacting protein 1
4452	Q9H8M7	2	2	7.2	445	49725	4.8	Protein FAM188A
4453	Q86WB0-3	2	7	7.9	431	47771	5.2	Isoform 3 of Nuclear-interacting partner of ALK
4453	C9J0I9	2	7	7.4	459	50562	5.8	Uncharacterized protein
4453	A4D1L4	2	7	7.1	481	53603	6.2	Nuclear interacting partner of anaplastic lymphoma kinase (ALK)
4453	Q86WB0-2	2	7	7.1	481	53559	6.4	Isoform 2 of Nuclear-interacting partner of ALK
4453	Q86WB0	2	7	6.8	502	55262	5.6	Nuclear-interacting partner of ALK
4454	Q7L8W6	2	3	7.1	267	30307	5.4	ATP-binding domain-containing protein 4
4455	Q68DQ7	2	3	7.3	385	42838	5.2	Branched-chain-amino-acid aminotransferase
4455	P54687	2	3	7.3	386	42966	5.3	Branched-chain-amino-acid aminotransferase, cytosolic
4455	B7Z2M5	2	3	7	398	44100	5.4	Branched-chain-amino-acid aminotransferase
4455	UPI0001D27F89	2	3	7	398	44132	5.4	branched-chain-amino-acid aminotransferase, cytosolic isoform 4
4456	P51531-2	2	4	7.1	1572	179280	7.3	Isoform Short of Probable global transcription activator SNF2L2
4456	P51531	2	4	7	1590	181278	7.2	Probable global transcription activator SNF2L2
4457	Q13371	2	4	7	301	34282	4.7	Phosducin-like protein
4458	Q86T04	2	2	7	383	42555	6.1	Full-length cDNA clone CS0DJ014YO21 of T cells (Jurkat cell line) of sapiens (human) (Fragment)
4458	Q8N0Z6	2	2	6.1	440	48928	6.5	Tetratricopeptide repeat protein 5
4459	Q9BRX9	2	2	7	315	34343	5.6	WD repeat domain-containing protein 83
4460	B7Z1Q8	2	2	8.7	527	59344	5.6	cDNA FLJ59617, highly similar to DCC-interacting protein 13 beta
4460	B7Z411	2	2	7.4	621	69730	5.1	cDNA FLJ54016, highly similar to DCC-interacting protein 13 beta
4460	A8K3E7	2	2	6.9	664	74466	4.9	cDNA FLJ76257, highly similar to sapiens DIP13 beta (DIP13B), mRNA
4460	Q8NEU8	2	2	6.9	664	74493	4.9	DCC-interacting protein 13-beta
4461	Q659C5	2	2	7.1	423	47494	6.1	Putative uncharacterized protein DKFZp434E1212 (Fragment)
4461	E9PD44	2	2	6.9	434	48673	6.3	Uncharacterized protein
4461	Q9HBU6	2	2	6.6	452	50968	6.5	Ethanolamine kinase 1
4462	O95551	2	2	6.9	362	40930	5.1	Tyrosyl-DNA phosphodiesterase 2
4463	Q9H6D7	2	2	6.9	363	42400	5.7	HAUS augmin-like complex subunit 4
4464	Q16831	2	2	6.8	310	33934	7.9	Uridine phosphorylase 1
4465	P29466-2	2	2	7	383	42888	6.2	Isoform Beta of Caspase-1
4465	A8K257	2	2	7	383	42860	6.2	cDNA FLJ75298, highly similar to sapiens caspase 1, apoptosis-related cysteine protease(interleukin 1, beta, convertase) (CASP1), transcript variant beta,mRNA
4465	A8K249	2	2	6.7	404	45099	5.9	cDNA FLJ77899, highly similar to sapiens caspase 1, apoptosis-related cysteine protease(interleukin 1, beta, convertase) (CASP1), transcript variantalpha, mRNA
4465	P29466	2	2	6.7	404	45159	5.9	Caspase-1
4465	B2R8U9	2	2	6.7	404	45171	5.9	cDNA, FLJ94074, highly similar to sapiens caspase 1, apoptosis-related cysteine protease(interleukin 1, beta, convertase) (CASP1), transcript variantalpha, mRNA
4466	O75909	2	4	6.9	580	64240	8.4	Cyclin-K
4466	C9JI13	2	4	6.7	600	66110	8.6	Uncharacterized protein
4466	O75909-4	2	4	6.7	600	66137	8.6	Isoform 4 of Cyclin-K
4467	P20248	2	3	6.7	432	48551	6.5	Cyclin-A2
4468	Q08431-2	2	2	8	312	35189	8.9	Isoform 2 of Lactadherin
4468	UPI000013D7A2	2	2	7.5	335	37505	8.2	lactadherin isoform b
4468	Q08431-3	2	2	7.5	335	37523	8.2	Isoform 3 of Lactadherin
4468	B3KTQ2	2	2	6.6	379	42460	7.8	cDNA FLJ38589 fis, clone HCHON2010074, highly similar to LACTADHERIN
4468	Q08431	2	2	6.5	387	43123	8.1	Lactadherin
4469	B3KTS4	2	2	6.6	709	80184	7.5	cDNA FLJ38665 fis, clone HLUNG2003378, highly similar to Rho guanine nucleotide exchange factor 16
4469	Q5VV41	2	2	6.6	709	80105	7.4	Rho guanine nucleotide exchange factor 16
4470	B7Z8M9	2	3	7.4	363	41127	8	cDNA FLJ58824, highly similar to Periphilin-1
4470	B7Z695	2	3	6.6	409	46732	8.5	cDNA FLJ52742, highly similar to Periphilin-1
4470	Q8NEY8-3	2	3	6.2	434	49624	8.7	Isoform 3 of Periphilin-1
4470	E9PEG4	2	3	6.2	434	49655	8.7	Uncharacterized protein
4470	Q8NEY8	2	3	5.9	458	52737	9.1	Periphilin-1
4471	Q2TAM5	2	2	7.2	377	42868	8.4	RELA protein
4471	E9PKH5	2	2	6.5	418	46697	8.4	Uncharacterized protein
4471	Q04206-3	2	2	5	541	59068	6	Isoform 3 of Transcription factor p65
4471	Q04206-2	2	2	5	537	58807	5.9	Isoform 2 of Transcription factor p65
4471	Q04206-4	2	2	4.9	548	59910	5.7	Isoform 4 of Transcription factor p65
4471	Q04206	2	2	4.9	551	60219	5.7	Transcription factor p65
4472	A0PJ61	2	2	6.4	440	49490	10.1	RSL1D1 protein (Fragment)
4472	Q32Q62	2	2	6	464	52165	10.2	RSL1D1 protein (Fragment)
4472	O76021	2	2	5.7	490	54973	10.1	Ribosomal L1 domain-containing protein 1
4472	E9PE52	2	2	5.7	489	54844	10.1	Uncharacterized protein
4473	Q9NSC1	2	3	7.1	325	36023	5.5	Homer-3B protein
4473	E9PCW9	2	3	7.1	325	35954	5.4	Uncharacterized protein
4473	B2RA10	2	3	6.4	361	39905	5.6	Homer log 3 (Drosophila), isoform CRA_b
4473	Q9NSC5-2	2	3	6.4	358	39479	5.7	Isoform 2 of Homer protein log 3
4473	Q9NSC5	2	3	6.4	361	39836	5.4	Homer protein log 3
4473	Q5U0L9	2	3	6.4	361	39963	5.5	Homer log 3 (Drosophila)
4474	Q5TZV8	2	2	7.2	442	48075	7	Interferon-related developmental regulator 2
4474	Q12894	2	2	6.3	506	54814	7.9	Interferon-related developmental regulator 2
4474	Q9UJ88	2	2	5.3	608	65598	8.6	Interferon-related IFRD2 (PC4-B) protein
4475	Q9HB40-2	2	6	8.4	296	32957	5	Isoform 2 of Retinoid-inducible serine carboxypeptidase
4475	B4DP22	2	6	6.2	402	45132	6	cDNA FLJ55993, highly similar to Retinoid-inducible serine carboxypeptidase (EC 3.4.16.-)
4475	Q9HB40	2	6	5.5	452	50831	5.8	Retinoid-inducible serine carboxypeptidase
4476	E9PNJ4	2	2	6.2	512	57620	8.3	Uncharacterized protein
4476	Q13586	2	2	4.7	685	77423	6.7	Stromal interaction molecule 1
4477	E7EV76	2	2	7.4	461	51132	6.4	Uncharacterized protein
4477	B4DN79	2	2	7.4	461	51142	6.4	cDNA FLJ53367, highly similar to Transmembrane glycoprotein NMB
4477	B2R5R1	2	2	6.1	560	62613	6.6	cDNA, FLJ92578, highly similar to sapiens glycoprotein (transmembrane) nmb (GPNMB), mRNA
4477	Q14956-2	2	2	6.1	560	62643	6.6	Isoform 2 of Transmembrane glycoprotein NMB
4477	Q14956	2	2	5.9	572	63923	6.6	Transmembrane glycoprotein NMB

Table S1. Proteins identified in the OVCAR-3 tumor supernatant by two or more unique peptides.

Protein or Protein Group	Accession Number	Unique Peptide Count	Spectrum Count	Sequence Coverage (%)	Length	MolWt	pI	Descriptive Name
4477	B4E3D4	2	2	5.6	607	68073	6.2	cDNA FLJ56293, highly similar to Transmembrane glycoprotein NMB
4478	UPI0001F77FD0	2	4	11.6	198	22344	5.7	UPI0001F77FD0 UniRef100 entry
4478	C9J3B0	2	4	6.1	378	43060	7.6	Uncharacterized protein
4478	Q5JTV8-2	2	4	6.1	378	43019	7.2	Isoform 2 of Torsin-1A-interacting protein 1
4478	E9PD36	2	4	5.5	419	47578	6.6	Uncharacterized protein
4478	Q5JTV8	2	4	3.9	583	66248	8.2	Torsin-1A-interacting protein 1
4479	Q7L775	2	4	6.1	607	70370	6.1	EPM2A-interacting protein 1
4480	Q9C0C4	2	2	6.1	833	92623	7.3	Semaphorin-4C
4481	A0PJ71	2	2	5.9	478	53226	9.1	NBN protein (Fragment)
4481	O60934	2	2	3.7	754	84959	6.9	Nibrin
4481	A8K1D3	2	2	3.7	754	84928	7.1	cDNA FLJ78201, highly similar to sapiens cell cycle regulatory protein p95 (NBS1) mRNA
4481	A6H8Y5	2	2	3.7	754	84974	7.1	Nibrin
4482	P28288-3	2	2	16.9	236	27121	9.8	Isoform 3 of ATP-binding cassette sub-family D member 3
4482	P28288-2	2	2	7.3	549	62726	9.2	Isoform 2 of ATP-binding cassette sub-family D member 3
4482	P28288	2	2	6.1	659	75476	9.4	ATP-binding cassette sub-family D member 3
4482	B4DL07	2	2	5.9	683	78460	9.1	cDNA FLJ53353, highly similar to ATP-binding cassette sub-family D member 3
4482	E7EUE1	2	2	5.9	683	78402	9.2	Uncharacterized protein
4483	B3KS28	2	3	5.8	362	39243	5.5	cDNA FLJ35355 fis, clone PUAEN2000152, moderately similar to N-terminal acetyltransferase C complexcatalytic subunit MAK3 (EC 2.3.1.88)
4483	Q147X3	2	3	5.8	362	39320	5.5	N-alpha-acetyltransferase 30, NatC catalytic subunit
4484	Q16773	2	5	7.1	422	47875	6.5	Kynurenine--oxoglutarate transaminase 1
4484	B7Z4W5	2	5	5.8	516	57668	7.4	Cysteine conjugate-beta lyase; cytoplasmic (Glutamine transaminase K, kyneurenine aminotransferase), isoform CRA_b
4485	UPI0001B793AD	2	2	11	210	24098	8.2	UPI0001B793AD UniRef100 entry
4485	UPI0000378301	2	2	10.8	212	24315	8.3	UPI0000378301 UniRef100 entry
4485	O77932	2	2	5.8	396	44929	7.7	Protein Dom3Z
4486	P09529	2	2	5.7	407	45122	8	Inhibin beta B chain
4487	Q3KQV9	2	2	5.7	507	57030	6.3	UDP-N-acetylhexosamine pyrophosphorylase-like protein 1
4488	E9PAW8	2	3	6	601	67712	4.7	Uncharacterized protein
4488	B3KMT5	2	3	5.6	644	72818	4.9	cDNA FLJ12554 fis, clone NT2RM4000741, highly similar to SGT1 protein
4488	Q6I9U8	2	3	5.6	644	72710	4.9	HSGT1 protein
4488	O95905	2	3	5.6	644	72758	4.9	Protein SGT1
4488	C9JX46	2	3	5.3	677	76508	4.9	Uncharacterized protein
4489	Q13884-2	2	2	7.9	382	40805	9.1	Isoform 2 of Beta-1-syntrophin
4489	Q13884	2	2	5.6	538	58061	8.6	Beta-1-syntrophin
4490	Q9UJX5-2	2	2	6.9	551	62639	6.2	Isoform 2 of Anaphase-promoting complex subunit 4
4490	B3KN47	2	2	5.5	697	79600	5.3	cDNA FLJ13535 fis, clone PLACE1006506, highly similar to Anaphase-promoting complex subunit 4
4490	Q9UJX5	2	2	4.7	808	92116	5.5	Anaphase-promoting complex subunit 4
4490	E9PCR4	2	2	4.7	809	92216	5.5	Uncharacterized protein
4491	B4E1J7	2	3	5.8	616	70189	6	cDNA FLJ56935, highly similar to Centromere/kinetochore protein zw10 log
4491	A1A528	2	3	5.4	672	76807	6.3	ZW10 protein
4491	O43264	2	3	4.6	779	88830	6.3	Centromere/kinetochore protein zw10 log
4491	B2R7C2	2	3	4.6	779	88840	6.3	cDNA, FLJ93375, highly similar to sapiens ZW10, kinetochore associated, log (Drosophila) (ZW10), mRNA
4492	B2R6F5	2	3	5.4	350	39625	5.1	cDNA, FLJ92928, highly similar to sapiens retinitis pigmentosa 2 (X-linked recessive) (RP2), mRNA
4492	O75695	2	3	5.4	350	39641	5.1	Protein XRP2
4493	B2R9R2	2	8	5.4	497	56624	6.6	cDNA, FLJ94517, highly similar to sapiens baculoviral IAP repeat-containing 4 (BIRC4), mRNA
4493	P98170	2	8	5.4	497	56685	6.6	Baculoviral IAP repeat-containing protein 4
4494	B7Z8U2	2	2	6	319	34124	4.7	cDNA FLJ57282, highly similar to GA-binding protein beta chain
4494	B7Z726	2	2	5.4	354	37372	4.7	cDNA FLJ60177, highly similar to GA-binding protein beta chain
4494	Q1RMI3	2	2	5	383	41321	4.8	GA-binding protein subunit beta-1
4494	Q06547	2	2	4.8	395	42483	4.9	GA-binding protein subunit beta-1
4495	Q05D28	2	2	5.4	370	41874	5.4	CCDC91 protein (Fragment)
4495	Q7Z6B0-3	2	2	4.9	405	45920	5	Isoform 3 of Coiled-coil domain-containing protein 91
4495	Q7Z6B0-2	2	2	4.9	411	47067	5.4	Isoform 2 of Coiled-coil domain-containing protein 91
4495	Q7Z6B0	2	2	4.5	441	49971	5.1	Coiled-coil domain-containing protein 91
4496	Q6NVY1	2	2	5.4	386	43482	8.2	3-hydroxyisobutyryl-CoA hydrolase, mitochondrial
4497	Q92851-6	2	2	5.7	455	51785	5.7	Isoform 6 of Caspase-10
4497	Q92851-2	2	2	5.4	479	54566	6.4	Isoform B of Caspase-10
4497	Q92851-4	2	2	5	522	58994	6.6	Isoform D of Caspase-10
4498	A8K4T3	2	5	5.2	477	54178	6.8	cDNA FLJ77716, highly similar to sapiens Zwilch, kinetochore associated, log (Drosophila), mRNA
4498	Q9H900-2	2	5	5.2	477	54224	6.8	Isoform 2 of Protein zwilch log
4498	Q9H900	2	5	4.2	591	67214	6.3	Protein zwilch log
4499	Q68D63	2	4	5.2	463	53667	9	Putative uncharacterized protein DKFZp686L0787
4499	Q9NQX6	2	4	5.2	463	53739	8.9	Zinc finger protein 331
4500	P16435	2	3	5.2	677	76690	5.6	NADPH--cytochrome P450 reductase
4500	A8K3B4	2	3	5.1	680	77034	5.6	cDNA FLJ77653
4500	Q63HL4	2	3	5.1	680	77018	5.6	Putative uncharacterized protein DKFZp686G04235
4500	Q59ED7	2	3	5.1	686	77737	5.6	Putative uncharacterized protein (Fragment)
4500	B2R6E5	2	3	5.1	680	77021	5.6	cDNA, FLJ92914, highly similar to sapiens P450 (cytochrome) oxidoreductase (POR), mRNA
4501	E9PN23	2	2	24.8	141	15906	5.4	Uncharacterized protein
4501	UPI000020E295	2	2	23.8	147	16665	4.7	HBS1-like protein (ERFS).
4501	E9PMN1	2	2	20.8	168	19162	4.7	Uncharacterized protein
4501	Q5T7G1	2	2	16.2	216	24334	5.4	HBS1-like (S. cerevisiae) (Fragment)
4501	Q9P0A5	2	2	14.2	246	27190	5.7	HSPC276 (Fragment)
4501	UPI0000D614D8	2	2	7.7	453	50365	7.3	UPI0000D614D8 UniRef100 entry
4501	Q9Y450-2	2	2	5.5	632	70128	5.9	Isoform 2 of HBS1-like protein
4501	A8K9E1	2	2	5.1	684	75503	6.6	cDNA FLJ77957, highly similar to sapiens HBS1-like (S. cerevisiae) (HBS1L), mRNA
4501	Q9Y450	2	2	5.1	684	75473	6.6	HBS1-like protein
4501	B3KNB9	2	2	5.1	684	75443	6.6	cDNA FLJ14196 fis, clone NT2RP3002330, highly similar to HBS1-like protein
4502	Q9P054	2	3	14.5	172	18700	5.5	HSPC328 (Fragment)
4502	B4DGI6	2	3	5.1	487	55570	9.1	cDNA FLJ50498, highly similar to ATP-dependent RNA helicase DDX24 (EC 3.6.1.-)
4502	E7ETZ2	2	3	5.1	487	55542	9.1	Uncharacterized protein
4502	Q59FS7	2	3	3.7	676	75048	8.5	DEAD (Asp-Glu-Ala-Asp) box polypeptide 24 variant (Fragment)
4502	E7EMJ4	2	3	3.3	766	86234	9	Uncharacterized protein
4502	B4DKV2	2	3	3.3	766	86189	9	cDNA FLJ54363, highly similar to ATP-dependent RNA helicase DDX24 (EC 3.6.1.-)
4502	Q4V9L5	2	3	3.1	804	90298	8.9	DDX24 protein

Table S1. Proteins identified in the OVCAR-3 tumor supernatant by two or more unique peptides.

Protein or Protein Group	Accession Number	Unique Peptide Count	Spectrum Count	Sequence Coverage (%)	Length	MolWt	pI	Descriptive Name
4502	Q9GZR7	2	3	2.9	859	96332	9.1	ATP-dependent RNA helicase DDX24
4503	E9PGJ9	2	2	5.1	573	63843	9.9	Uncharacterized protein
4503	Q9NX28	2	2	5.1	572	63701	9.9	cDNA FLJ20475 fis, clone KAT07206
4503	Q6P1N0-2	2	2	3.1	950	103934	8.1	Isoform 2 of Coiled-coil and C2 domain-containing protein 1A
4503	Q6P1N0	2	2	3	951	104062	8.1	Coiled-coil and C2 domain-containing protein 1A
4504	P41214-2	2	2	6.5	460	51013	8.9	Isoform 2 of Eukaryotic translation initiation factor 2D
4504	P41214	2	2	5.1	584	64707	7.6	Eukaryotic translation initiation factor 2D
4505	UPI000056D41D	2	4	6.6	472	52175	7.8	UPI000056D41D UniRef100 entry
4505	Q6L8Q7-2	2	4	5.8	535	58890	6.9	Isoform 2 of 2',5'-phosphodiesterase 12
4505	Q6L8Q7	2	4	5.1	609	67352	6.6	2',5'-phosphodiesterase 12
4506	Q9BV38	2	2	5.1	432	47405	6.7	WD repeat-containing protein 18
4507	B3KSN1	2	3	5	614	68900	5.8	cDNA FLJ36670 fis, clone UTERU2004037, highly similar to Conserved oligomeric Golgi complex component 7
4507	P83436	2	3	4	770	86344	5.5	Conserved oligomeric Golgi complex subunit 7
4508	B4E074	2	2	5	461	50729	7	cDNA FLJ58655, highly similar to Notchless log 1
4508	E9PEC6	2	2	5	462	50772	7	Uncharacterized protein
4508	Q9NVX2	2	2	4.7	485	53320	7.3	Notchless protein log 1
4509	Q5T8J2	2	2	7.7	286	32908	8.9	Bystin-like
4509	Q13895	2	2	5	437	49601	8.1	Bystin
4510	Q96JB2	2	2	5	828	94096	5.6	Conserved oligomeric Golgi complex subunit 3
4511	Q9NVR5	2	3	5	837	91114	5.2	Protein kintoun
4512	E9PF19	2	5	5.4	411	45935	9.5	Uncharacterized protein
4512	B4DY50	2	5	5.4	411	45949	9.5	cDNA FLJ55528, highly similar to Transducin beta-like 2 protein
4512	B2RB52	2	5	4.9	447	49828	9.4	cDNA, FLJ95314, highly similar to sapiens transducin (beta)-like 2 (TBL2), transcript variant 1, mRNA
4512	Q9Y4P3	2	5	4.9	447	49798	9.4	Transducin beta-like protein 2
4512	Q96E41	2	5	4.9	446	49525	9.2	TBL2 protein
4512	Q8N2L6	2	5	4.9	447	49824	9.4	cDNA FLJ90138 fis, clone HEMBB1000905, weakly similar to TRANSCRIPTIONAL REPRESSOR RCO-1
4513	B4E014	2	4	4.9	649	75032	7.7	cDNA FLJ54334, highly similar to Origin recognition complex subunit 3
4513	E7ENV6	2	4	4.9	649	75036	7.6	Uncharacterized protein
4513	Q9UBD5-2	2	4	4.5	712	82325	7.6	Isoform 2 of Origin recognition complex subunit 3
4513	Q9UBD5	2	4	4.5	711	82254	7.6	Origin recognition complex subunit 3
4514	B4E2Z3	2	4	4.9	511	55940	5.2	cDNA FLJ54090, highly similar to 4F2 cell-surface antigen heavy chain
4514	P08195-3	2	4	4.4	568	61816	5.2	Isoform 3 of 4F2 cell-surface antigen heavy chain
4514	UPI00004EC298	2	4	4	631	68101	5	4F2 cell-surface antigen heavy chain isoform b
4514	P08195	2	4	4	630	67994	5	4F2 cell-surface antigen heavy chain
4514	P08195-4	2	4	3.8	661	71123	5	Isoform 4 of 4F2 cell-surface antigen heavy chain
4515	Q15050	2	2	4.9	365	41193	10.7	Ribosome biogenesis regulatory protein log
4516	Q68BL7	2	5	4.9	652	73054	7.8	Olfactomedin-like protein 2A
4517	UPI000198C8F9	2	2	9.3	269	30349	6.7	UPI000198C8F9 UniRef100 entry
4517	Q9BV44	2	2	4.9	507	57003	6.4	THUMP domain-containing protein 3
4518	A1BQX2	2	2	4.8	442	51121	7.6	Tricellulin isoform c
4518	Q8N4S9-3	2	2	3.8	546	62797	7.2	Isoform 3 of MARVEL domain-containing protein 2
4518	Q8N4S9	2	2	3.8	558	64168	7.5	MARVEL domain-containing protein 2
4519	Q9BTE6	2	3	6.8	412	45480	6.4	Alanyl-tRNA editing protein Aarsd1
4519	C9J5N1	2	3	5.7	495	55066	6.5	Uncharacterized protein
4519	B4DI73	2	3	4.8	586	65702	6.5	Alanyl-tRNA synthetase domain containing 1, isoform CRA_a
4520	E9PKG6	2	3	5.9	338	40342	5.2	Uncharacterized protein
4520	D3DQX5	2	3	4.8	420	50223	5.1	Nucleobindin 2, isoform CRA_a
4520	P80303-2	2	3	4.8	420	50201	5.1	Isoform 2 of Nucleobindin-2
4520	P80303	2	3	4.8	420	50196	5.1	Nucleobindin-2
4521	B7Z4B1	2	3	5.3	490	58839	9.2	cDNA FLJ50801, highly similar to Coiled-coil domain-containing protein 55
4521	B7ZL27	2	3	4.9	534	63685	8.9	CCDC55 protein (Fragment)
4521	B2R9Y2	2	3	4.7	558	66389	9	cDNA, FLJ94609
4521	Q9H0G5	2	3	4.7	558	66390	8.8	Coiled-coil domain-containing protein 55
4522	B8ZZG1	2	2	4.7	428	48758	8.8	Membrane protein, palmitoylated 6 (MAGUK p55 subfamily member 6), isoform CRA_a
4522	Q9NZW5	2	2	3.7	540	61117	6.2	MAGUK p55 subfamily member 6
4522	B9A001	2	2	3.4	582	65914	6.1	Uncharacterized protein
4523	C9JWC5	2	3	7.5	305	34866	7.5	Uncharacterized protein
4523	B4DVR0	2	3	5.5	420	47411	7.9	cDNA FLJ51115, moderately similar to Tripartite motif-containing protein 22
4523	Q8IYM9-2	2	3	4.7	494	56429	7.7	Isoform 2 of E3 ubiquitin-protein ligase TRIM22
4523	D3DQT1	2	3	4.7	494	56373	7.7	Tripartite motif-containing 22, isoform CRA_a
4523	A8K319	2	3	4.6	498	56892	7.6	cDNA FLJ76655, highly similar to sapiens tripartite motif-containing 22, mRNA
4523	Q8IYM9	2	3	4.6	498	56947	7.7	E3 ubiquitin-protein ligase TRIM22
4523	D3DQT0	2	3	4.6	498	56891	7.7	Tripartite motif-containing 22, isoform CRA_b
4524	Q3YEC7-5	2	2	9.3	258	29672	8.4	Isoform 5 of Putative GTP-binding protein Parf
4524	C6K8I5	2	2	9.3	257	29585	8.4	Rab-like GTP-binding protein 1D
4524	C6K8I4	2	2	7.6	314	35809	7.7	Rab-like GTP-binding protein 1C
4524	UPI000046FF02	2	2	5	484	53859	7.9	UPI000046FF02 UniRef100 entry
4524	A8QVZ8	2	2	4.6	520	57397	8.3	Chromosome 9 open reading frame 86
4524	C9JJY3	2	2	4.6	521	57484	8.3	Uncharacterized protein
4524	Q3YEC7-2	2	2	3.3	730	79636	5.2	Isoform 2 of Putative GTP-binding protein Parf
4524	Q3YEC7	2	2	3.3	729	79549	5.2	Putative GTP-binding protein Parf
4525	B4E1K0	2	2	4.5	673	77699	8	cDNA FLJ58416, highly similar to Kinesin-like protein KIF23
4525	Q02241-2	2	2	3.5	856	98105	8.5	Isoform 2 of Kinesin-like protein KIF23
4525	UPI00015DFCE3	2	2	3.1	960	110099	8.5	Kinesin-like protein KIF23 (Mitotic kinesin-like protein 1) (Kinesin-like protein 5).
4525	Q02241	2	2	3.1	960	110059	8.5	Kinesin-like protein KIF23
4526	Q9UBT7-3	2	2	4.9	650	72616	7.1	Isoform 3 of Alpha-catulin
4526	Q9UBT7-2	2	2	4.5	718	80152	6.7	Isoform 2 of Alpha-catulin
4526	B2RBI4	2	2	4.4	734	81859	6.6	cDNA, FLJ95528, highly similar to sapiens catenin (cadherin-associated protein), alpha-like 1 (CTNNAL1), mRNA
4526	Q9UBT7	2	2	4.4	734	81896	6.6	Alpha-catulin
4526	B3KMX6	2	2	4.4	734	81897	6.6	cDNA FLJ12864 fis, clone NT2RP2003604, highly similar to Alpha-catulin
4527	B3KUM5	2	2	4.4	612	71321	5.8	cDNA FLJ40245 fis, clone TESTI2023968, highly similar to GC-RICH SEQUENCE DNA-BINDING FACTOR
4527	P16383-2	2	2	3.6	743	84847	6.2	Isoform 2 of GC-rich sequence DNA-binding factor
4527	P16383	2	2	3.5	781	89385	6	GC-rich sequence DNA-binding factor
4528	B4E184	2	6	4.4	520	59613	5.3	cDNA FLJ53267, highly similar to Optineurin
4528	B7ZA56	2	6	4.4	520	59560	5.2	cDNA, FLJ79070, highly similar to Optineurin
4528	Q96CV9-2	2	6	4	571	65202	5.2	Isoform 2 of Optineurin
4528	Q96CV9	2	6	4	577	65921	5.2	Optineurin
4529	O00186	2	4	4.4	592	67764	7.8	Syntaxin-binding protein 3
4530	Q6P3X3	2	3	4.4	843	96632	5.6	Tetratricopeptide repeat protein 27

Table S1. Proteins identified in the OVCAR-3 tumor supernatant by two or more unique peptides.

Protein or Protein Group	Accession Number	Unique Peptide Count	Spectrum Count	Sequence Coverage (%)	Length	MolWt	pI	Descriptive Name
4531	B4DKY2	2	5	4.3	650	72763	5.2	cDNA FLJ52322, highly similar to Tyrosine-protein phosphatase non-receptortype 12 (EC 3.1.3.48)
4531	E9PBR5	2	5	4.2	661	74139	5.2	Uncharacterized protein
4531	Q05209	2	5	3.6	780	88106	5.6	Tyrosine-protein phosphatase non-receptor type 12
4532	Q9UBH6-2	2	2	4.8	631	73919	7.5	Isoform 2 of Xenotropic and polytropic retrovirus receptor 1
4532	Q9UBH6	2	2	4.3	696	81535	8.4	Xenotropic and polytropic retrovirus receptor 1
4533	B4DQ08	2	2	4.2	719	81947	8.1	cDNA FLJ53406, highly similar to U3 small nucleolar RNA-associated protein 14 log A
4533	E9PEL7	2	2	4.2	719	82005	7.9	Uncharacterized protein
4533	Q9BVJ6	2	2	3.9	771	87978	7.9	U3 small nucleolar RNA-associated protein 14 log A
4534	Q96P63	2	2	4.4	405	46276	5.5	Serpin B12
4534	Q3SYB4	2	2	4.2	425	48446	5.4	SERPINB12 protein
4535	Q9Y4R8	2	6	4.1	837	91747	5.8	Telomere length regulation protein TEL2 log
4536	E9PNS7	2	2	4.5	757	83489	6.1	Uncharacterized protein
4536	B2RAF9	2	2	4	855	94751	6.5	cDNA, FLJ94888, highly similar to sapiens suppression of tumorigenicity 14 (colon carcinoma, matriptase, epithin) (ST14), mRNA
4536	Q9Y5Y6	2	2	4	855	94770	6.6	Suppressor of tumorigenicity 14 protein
4537	B7WP27	2	2	4	908	105524	7	Uncharacterized protein
4537	Q9HCG8	2	2	4	908	105466	7	Pre-mRNA-splicing factor CWC22 log
4538	Q8IXB1-2	2	2	4.3	747	86136	7.4	Isoform 2 of DnaJ log subfamily C member 10
4538	Q8IXB1	2	2	4	793	91080	7.2	DnaJ log subfamily C member 10
4539	A2RRC9	2	3	3.9	1631	184517	7.5	IQ motif containing GTPase activating protein 3
4539	UPI0000231C93	2	3	3.9	1631	184598	7.6	ras GTPase-activating-like protein IQGAP3
4539	Q86VI3	2	3	3.9	1631	184698	7.6	Ras GTPase-activating-like protein IQGAP3
4540	Q8N954	2	2	3.9	259	30199	5.2	Coiled-coil domain-containing protein 75
4541	B2RDL5	2	2	3.8	990	107277	6.3	cDNA, FLJ96668, sapiens fucokinase (FUK), mRNA
4541	Q8N0W3-2	2	2	3.5	1090	118393	6.4	Isoform 2 of L-fucose kinase
4541	Q8N0W3	2	2	3.5	1084	117623	6.3	L-fucose kinase
4542	E9PCQ5	2	2	3.8	765	82479	7.7	Uncharacterized protein
4542	Q8N3F8	2	2	3.4	863	93441	7.2	MICAL-like protein 1
4543	E9PPE7	2	2	5	764	81488	4.6	Uncharacterized protein
4543	A2RUM0	2	2	3.7	1025	109940	5	Poly (ADP-ribose) polymerase family, member 10
4543	Q96K72	2	2	3.7	1025	109884	5	cDNA FLJ14464 fis, clone MAMMA1000309
4543	Q53GL7	2	2	3.7	1025	109998	5	Poly [ADP-ribose] polymerase 10
4543	E9PNI7	2	2	3.7	1037	111392	5	Uncharacterized protein
4543	E9PK67	2	2	3.7	1016	109097	5	Uncharacterized protein
4543	B4E0C4	2	2	3.7	1037	111406	5	cDNA FLJ57250, highly similar to sapiens poly (ADP-ribose) polymerase family, member 10 (PARP10), mRNA
4544	Q8WXA9	2	10	3.7	508	59380	10.4	Splicing regulatory glutamine/lysine-rich protein 1
4545	Q9BRZ2	2	4	3.7	755	81488	7.7	E3 ubiquitin-protein ligase TRIM56
4546	P14384	2	2	3.6	443	50514	7.4	Carboxypeptidase M
4547	P51511	2	3	3.6	669	75807	7.5	Matrix metalloproteinase-15
4548	C9JP68	2	2	3.5	780	85605	5.1	Uncharacterized protein
4548	UPI0000E0C769	2	2	2	1341	149425	5.9	immunoglobulin superfamily member 1 isoform 3
4548	Q8N6C5-2	2	2	2	1327	147972	5.8	Isoform 2 of Immunoglobulin superfamily member 1
4548	Q8N6C5	2	2	2	1336	148935	5.9	Immunoglobulin superfamily member 1
4549	Q53FX3	2	2	3.5	941	107768	6.9	Vacuolar protein sorting 11 (Yeast log) variant (Fragment)
4549	UPI0000161C99	2	2	3.5	941	107809	7.1	vacuolar protein sorting-associated protein 11 log
4549	Q9H270	2	2	3.5	941	107837	7.1	Vacuolar protein sorting-associated protein 11 log
4549	Q658K0	2	2	3.5	940	107641	7	Putative uncharacterized protein DKFZp564P2364
4550	Q8N4U5	2	2	3.5	519	58091	5	T-complex protein 11-like protein 2
4551	Q8ND04-3	2	2	6	588	65088	7.7	Isoform 3 of Protein SMG8
4551	Q8ND04	2	2	3.5	991	109684	7.7	Protein SMG8
4551	Q8ND04-2	2	2	3.4	1023	113334	7.7	Isoform 2 of Protein SMG8
4552	Q9H1H9-3	2	2	3.5	1749	196069	5.7	Isoform 3 of Kinesin-like protein KIF13A
4553	Q9Y5B0	2	2	3.5	961	104399	5.3	RNA polymerase II subunit A C-terminal domain phosphatase
4554	A8K6X5	2	4	3.4	1068	122248	7.2	cDNA FLJ78680, highly similar to sapiens dishevelled associated activator of morphogenesis 1, mRNA
4554	Q9Y4D1-2	2	4	3.4	1068	122306	7.2	Isoform 2 of Disheveled-associated activator of morphogenesis 1
4554	Q9Y4D1	2	4	3.3	1078	123473	7.2	Disheveled-associated activator of morphogenesis 1
4555	Q3KQU3-3	2	2	5	377	42105	9.2	Isoform 3 of MAP7 domain-containing protein 1
4555	B5MDZ6	2	2	3.4	552	60987	9.9	Uncharacterized protein
4555	Q6NX58	2	2	2.8	678	75668	10.3	MAP7D1 protein (Fragment)
4555	Q3KQU3-4	2	2	2.4	808	89467	10.1	Isoform 4 of MAP7 domain-containing protein 1
4555	Q3KQU3-2	2	2	2.4	803	88726	10.1	Isoform 2 of MAP7 domain-containing protein 1
4555	Q3KQU3	2	2	2.3	841	92820	10.1	MAP7 domain-containing protein 1
4555	D3DPS3	2	2	2.3	840	92692	10.1	Arginine/proline rich coiled-coil 1, isoform CRA_b
4556	Q92541	2	3	3.4	710	80314	8.2	RNA polymerase-associated protein RTF1 log
4557	B4E3T7	2	3	3.5	1090	122310	4.9	cDNA FLJ55448, highly similar to Nuclear pore complex protein Nup133
4557	B4E206	2	3	3.3	1140	127402	5	cDNA FLJ55441, highly similar to Nuclear pore complex protein Nup133
4557	Q8WUM0	2	3	3.3	1156	128979	5.1	Nuclear pore complex protein Nup133
4558	B4E0F9	2	5	6.1	408	45499	9.3	cDNA FLJ60383, highly similar to Ubiquitin-like PHD and RING fingerdomain-containing protein 1 (EC 6.3.2.-)
4558	A8K024	2	5	3.2	793	89842	7.3	cDNA FLJ78091, highly similar to sapiens ubiquitin-like, containing PHD and RING finger domains, 1 (UHRF1), mRNA
4558	UPI0000456BDE	2	5	3.2	792	89743	7.6	UPI0000456BDE UniRef100 entry
4558	Q96T88	2	5	3.2	793	89814	7.6	E3 ubiquitin-protein ligase UHRF1
4558	B2RBA9	2	5	3.2	793	89843	7.7	cDNA, FLJ95411, highly similar to sapiens ubiquitin-like, containing PHD and RING finger domains, 1 (UHRF1), mRNA
4558	UPI0001AE63C5	2	5	3.1	805	91013	7.4	E3 ubiquitin-protein ligase UHRF1 (EC 6.3.2.-) (Ubiquitin-like PHD and RING finger domain-containing protein 1) (Ubiquitin-like-containing PHD and RING finger domains protein 1) (Inverted CCAAT box-binding protein of 90 kDa) (Transcription factor ICBP90)
4558	Q2HIX7	2	5	3.1	806	91116	7.4	Ubiquitin-like with PHD and ring finger domains 1
4559	UPI0001C0B383	2	3	3.9	722	77731	5.4	breast cancer anti-estrogen resistance protein 1 isoform 8
4559	B7Z7X7	2	3	3.9	722	77759	5.5	cDNA FLJ55908, highly similar to Breast cancer anti-estrogen resistance protein 1
4559	B2RBL9	2	3	3.2	870	93405	5.6	cDNA, FLJ95582, highly similar to sapiens breast cancer anti-estrogen resistance 1 (BCAR1),mRNA
4559	UPI0001C0B382	2	3	3.2	888	95454	5.9	breast cancer anti-estrogen resistance protein 1 isoform 4
4559	UPI00001FFC19	2	3	3.2	870	93291	5.6	breast cancer anti-estrogen resistance protein 1 isoform 5
4559	Q6QEF7	2	3	3.2	888	95339	6	Breast cancer anti-estrogen resistance 1
4559	Q6P5Z4	2	3	3.2	870	93389	5.7	Breast cancer anti-estrogen resistance 1
4559	P56945	2	3	3.2	870	93372	5.7	Breast cancer anti-estrogen resistance protein 1
4559	E9PCV2	2	3	3.2	888	95216	5.7	Uncharacterized protein
4559	D3DUK1	2	3	3.2	888	95206	5.7	Breast cancer anti-estrogen resistance 1, isoform CRA_a
4559	B4DIW5	2	3	3.2	888	95469	5.7	cDNA FLJ55515, highly similar to Breast cancer anti-estrogen resistanceprotein 1

Table S1. Proteins identified in the OVCAR-3 tumor supernatant by two or more unique peptides.

Protein or Protein Group	Accession Number	Unique Peptide Count	Spectrum Count	Sequence Coverage (%)	Length	MolWt	pI	Descriptive Name
4559	B3KWD7	2	3	3.2	888	95246	5.7	cDNA FLJ42825 fis, clone BRCAN2016619, highly similar to Breast cancer anti-estrogen resistance protein 1
4559	E9PCL5	2	3	3.1	916	97877	5.6	Uncharacterized protein
4559	B4DEV4	2	3	3.1	916	97867	5.6	cDNA FLJ56057, highly similar to Breast cancer anti-estrogen resistance protein 1
4560	Q9UQ88-5	2	2	6.3	397	45229	9.2	Isoform SV7 of Cyclin-dependent kinase 11A
4560	Q96CA8	2	2	5.4	464	52406	5.1	CDC2L2 protein (Fragment)
4560	P21127-4	2	2	5.4	461	51925	5.2	Isoform 3 of Cyclin-dependent kinase 11B
4560	Q6P5Y5	2	2	5.3	475	53688	4.8	CDC2L2 protein (Fragment)
4560	Q4VBY6	2	2	5.3	475	53757	4.8	CDC2L2 protein (Fragment)
4560	P21127-10	2	2	4.4	565	63937	4.7	Isoform SV11 of Cyclin-dependent kinase 11B
4560	Q5QPR4	2	2	3.4	746	86824	5.3	Cell division cycle 2-like 2 (PITSLRE proteins)
4560	P21127-6	2	2	3.4	738	86063	5.3	Isoform SV5 of Cyclin-dependent kinase 11B
4560	E9PGA8	2	2	3.4	735	85596	5.3	Uncharacterized protein
4560	E7ERC1	2	2	3.4	746	86755	5.4	Uncharacterized protein
4560	P21127-9	2	2	3.3	748	87138	5.4	Isoform SV10 of Cyclin-dependent kinase 11B
4560	B4E3D9	2	2	3.2	772	90235	5.4	cDNA FLJ59152, highly similar to PITSLRE serine/threonine-protein kinaseCDC2L1 (EC 2.7.11.22)
4560	UPI0001AE7818	2	2	3.2	780	90949	5.4	PITSLRE serine/threonine-protein kinase CDC2L2 (EC 2.7.11.22) (Galactosyltransferase-associated protein kinase p58/GTA) (Cell division cycle 2-like protein kinase 2) (CDK11).
4560	UPI0000160F88	2	2	3.2	771	90047	5.4	cyclin-dependent kinase 11B isoform 9
4560	UPI0000160F86	2	2	3.2	780	91009	5.4	cyclin-dependent kinase 11B isoform 8
4560	Q9UQ88-4	2	2	3.2	770	90045	5.3	Isoform SV3 of Cyclin-dependent kinase 11A
4560	Q9UQ88-3	2	2	3.2	779	91055	5.3	Isoform SV2 of Cyclin-dependent kinase 11A
4560	Q9UQ88-2	2	2	3.2	780	91018	5.4	Isoform SV1 of Cyclin-dependent kinase 11A
4560	Q9UQ88	2	2	3.2	783	91362	5.4	Cyclin-dependent kinase 11A
4560	Q5QPR3	2	2	3.2	779	91007	5.3	Cell division cycle 2-like 2 (PITSLRE proteins)
4560	P21127-8	2	2	3.2	772	90256	5.4	Isoform 8 of Cyclin-dependent kinase 11B
4560	P21127-3	2	2	3.2	781	91219	5.4	Isoform 2 of Cyclin-dependent kinase 11B
4560	P21127-2	2	2	3.2	782	91332	5.5	Isoform SV1 of Cyclin-dependent kinase 11B
4560	E7EP51	2	2	3.2	778	90752	5.4	Uncharacterized protein
4560	B7ZVY7	2	2	3.2	782	91303	5.5	Cell division cycle 2-like 1 (PITSLRE proteins)
4560	P21127	2	2	3.1	795	92707	5.6	Cyclin-dependent kinase 11B
4560	E7EUE4	2	2	3.1	797	91867	5.4	Uncharacterized protein
4561	P42338	2	4	3.1	1070	122762	7.1	Phosphatidylinositol-4,5-bisphosphate 3-kinase catalytic subunit beta isoform
4562	B4DG99	2	3	3	1024	115310	7	cDNA FLJ60247, highly similar to Tudor domain-containing protein 7
4562	Q8NHU6	2	3	2.8	1098	123585	7.2	Tudor domain-containing protein 7
4563	Q6ZMP0-3	2	7	9.1	219	24246	9.4	Isoform 3 of Thrombospondin type-1 domain-containing protein 4
4563	B4E1J6	2	7	6.1	329	36378	7.8	cDNA FLJ54862, weakly similar to sapiens thrombospondin repeat containing 1 (TSRC1), transcript variant 2, mRNA
4563	Q6ZMP0-2	2	7	3.8	523	56628	8.9	Isoform 2 of Thrombospondin type-1 domain-containing protein 4
4563	B4DR13	2	7	3	658	73409	6	cDNA FLJ53165, weakly similar to sapiens thrombospondin repeat containing 1 (TSRC1), transcript variant 1, mRNA
4563	Q6ZMP0	2	7	2	1018	112450	7.6	Thrombospondin type-1 domain-containing protein 4
4564	B4DTQ6	2	2	3	944	106506	6.9	cDNA FLJ54883, highly similar to Unc-13 log D
4564	Q70J99	2	2	2.6	1090	123282	6.7	Protein unc-13 log D
4564	Q70J99-3	2	2	2.5	1142	128819	6.6	Isoform 3 of Protein unc-13 log D
4565	D6RD46	2	18	3	980	109935	6.5	Uncharacterized protein
4565	Q9UPQ0-4	2	18	2.7	1069	120496	6.6	Isoform 4 of LIM and calponin logy domains-containing protein 1
4565	Q9UPQ0-2	2	18	2.7	1057	119078	6.3	Isoform 2 of LIM and calponin logy domains-containing protein 1
4565	Q9UPQ0	2	18	2.7	1083	121867	6.5	LIM and calponin logy domains-containing protein 1
4565	E9PHM7	2	18	2.7	1056	118950	6.3	Uncharacterized protein
4566	C9JFV6	2	3	2.9	1162	124218	8.7	Uncharacterized protein
4566	Q92618	2	3	2.9	1163	124289	8.7	Zinc finger protein 516
4567	P32019-2	2	2	3.2	913	103987	5.4	Isoform 2 of Type II inositol-1,4,5-trisphosphate 5-phosphatase
4567	P32019	2	2	2.9	993	112852	5.6	Type II inositol-1,4,5-trisphosphate 5-phosphatase
4568	UPI000022C483	2	2	5.2	366	41735	8	guanylate cyclase soluble subunit alpha-3 isoform C
4568	D6RDW3	2	2	3	624	70184	7.4	Uncharacterized protein
4568	B3KU69	2	2	2.8	690	77353	7	cDNA FLJ39269 fis, clone OCBBF2010420, highly similar to Guanylate cyclase soluble subunit alpha-3 (EC 4.6.1.2)
4568	Q02108	2	2	2.8	690	77453	7.1	Guanylate cyclase soluble subunit alpha-3
4569	Q5T5P2-4	2	13	4.4	866	95123	8.2	Isoform 4 of Sickle tail protein log
4569	Q5T5P2-8	2	13	4.2	906	99494	8.5	Isoform 7 of Sickle tail protein log
4569	Q5T5P2-6	2	13	4.1	932	102692	7.8	Isoform 6 of Sickle tail protein log
4569	Q5T5P1	2	13	4.1	933	103666	9.1	KIAA1217 (Fragment)
4569	UPI0001533DB3	2	13	2.9	1309	143053	9.1	sickle tail protein log isoform 3
4569	Q5T5P2-7	2	13	2.8	1349	147424	9.1	Isoform 5 of Sickle tail protein log
4569	Q5T5P2-2	2	13	2.8	1344	146878	9.2	Isoform 2 of Sickle tail protein log
4569	B9EGK4	2	13	2.8	1374	150523	9	KIAA1217 protein
4569	B7ZM29	2	13	2.8	1349	147440	9.1	KIAA1217 protein
4569	A6NLF3	2	13	2.7	1410	154447	9.1	Uncharacterized protein
4569	C9JRK3	2	13	2.6	1461	161951	6.2	Uncharacterized protein
4569	E9PHU3	2	13	2.5	1534	168161	6.9	Uncharacterized protein
4569	B4DTY7	2	13	2.5	1532	167981	6.9	cDNA FLJ57813, weakly similar to p130Cas-associated protein
4569	Q5T5P2	2	13	2	1943	214114	7.1	Sickle tail protein log
4570	Q59HA5	2	11	2.8	1196	130790	5.8	Cyclin G-associated kinase variant (Fragment)
4570	B4DS37	2	11	2.7	1213	132145	5.7	cDNA FLJ58258, highly similar to Cyclin G-associated kinase (EC 2.7.11.1)
4570	Q5U4P5	2	11	2.7	1232	134410	5.8	GAK protein
4570	E9PGR2	2	11	2.7	1232	134464	5.8	Uncharacterized protein
4570	O14976	2	11	2.5	1311	143190	5.7	Cyclin-G-associated kinase
4571	Q86VM9-2	2	4	3.4	757	84100	5.6	Isoform 2 of Zinc finger CCCH domain-containing protein 18
4571	C9JA72	2	4	3.4	757	84086	5.6	Uncharacterized protein
4571	B4DTK7	2	4	2.7	977	108978	8.4	cDNA FLJ61387, highly similar to sapiens conserved nuclear protein NHN1 (NHN1), mRNA
4571	Q86VM9	2	4	2.7	953	106379	8.3	Zinc finger CCCH domain-containing protein 18
4571	E7ERS3	2	4	2.7	977	108940	8.4	Uncharacterized protein
4572	Q8WVK6	2	2	7.6	343	37501	5.9	IREB2 protein
4572	E1CJT9	2	2	7.6	343	37467	5.9	Iron-responsive element binding protein 2 variant
4572	D3DW85	2	2	2.7	963	105046	7.1	Iron-responsive element binding protein 2, isoform CRA_a
4572	P48200	2	2	2.7	963	105044	7.1	Iron-responsive element-binding protein 2
4573	A6NDZ9	2	2	2.6	1038	109883	4.3	Uncharacterized protein
4573	O15451	2	2	2.6	1021	109140	4.3	Proline and glutamic acid rich nuclear protein isoform (Fragment)
4573	Q8IZL8	2	2	2.4	1130	119700	4.3	Proline-, glutamic acid- and leucine-rich protein 1
4573	A8K548	2	2	2.4	1130	119642	4.3	cDNA FLJ75008, highly similar to sapiens proline-, glutamic acid-, leucine-rich protein 1 (PELP1), mRNA
4573	B4DR36	2	2	2.3	1180	124868	4.4	cDNA FLJ56414, highly similar to sapiens proline-, glutamic acid-, leucine-rich protein 1 (PELP1), mRNA
4573	Q8IZL8-2	2	2	2.1	1275	135495	4.4	Isoform 2 of Proline-, glutamic acid- and leucine-rich protein 1

Table S1. Proteins identified in the OVCAR-3 tumor supernatant by two or more unique peptides.

Protein or Protein Group	Accession Number	Unique Peptide Count	Spectrum Count	Sequence Coverage (%)	Length	MolWt	pI	Descriptive Name
4573	C9JFV4	2	2	2.1	1274	135512	4.4	Uncharacterized protein
4574	O60245	2	2	2.6	1069	116071	5.2	Protocadherin-7
4574	O60245-2	2	2	2.6	1072	116429	5.2	Isoform B of Protocadherin-7
4574	O60245-3	2	2	2.3	1200	130303	5.2	Isoform C of Protocadherin-7
4574	UPI0001CB27C3	2	2	2.2	1255	136570	5.2	protocadherin-7 isoform d precursor
4574	UPI0001CB27C2	2	2	2.2	1247	135672	5.2	protocadherin-7 isoform c precursor
4575	Q02413	2	2	2.6	1049	113748	5	Desmoglein-1
4576	Q9BXW9-3	2	3	3	1249	140737	6.1	Isoform 3 of Fanconi anemia group D2 protein
4576	Q9BXW9	2	3	2.6	1471	166461	6.2	Fanconi anemia group D2 protein
4576	Q9BXW9-2	2	3	2.6	1451	164128	5.9	Isoform 2 of Fanconi anemia group D2 protein
4577	B4DZ36	2	2	2.5	1156	129834	6.9	cDNA FLJ58441, highly similar to Attractin
4577	O75882-3	2	2	2.4	1198	133701	7	Isoform 3 of Attractin
4577	C9J7K1	2	2	2.3	1268	140942	7.1	Uncharacterized protein
4577	C9IZD4	2	2	2.3	1272	141413	7	Uncharacterized protein
4577	O75882	2	2	2	1429	158536	7.3	Attractin
4578	P11137	2	3	2.4	1827	199525	4.9	Microtubule-associated protein 2
4578	P11137-3	2	3	2.4	1823	199082	4.9	Isoform 3 of Microtubule-associated protein 2
4579	B7Z6Q8	2	2	2.5	935	104507	7	Ephrin receptor
4579	Q58F15	2	2	2.4	949	105744	6.2	Ephrin receptor
4579	A8K2P1	2	2	2.3	986	109831	6.6	Ephrin receptor
4579	P54764	2	2	2.3	986	109860	6.6	Ephrin type-A receptor 4
4579	B2R601	2	2	2.3	986	109846	6.6	Ephrin receptor
4580	O43314-2	2	4	2.4	1222	138106	8.1	Isoform 2 of Inositol hexakisphosphate and diphosphoinositol-pentakisphosphate kinase 2
4580	B4DGV1	2	4	2.3	1270	143477	7.5	cDNA FLJ61589 (Fragment)
4580	O43314	2	4	2.3	1243	140406	8.2	Inositol hexakisphosphate and diphosphoinositol-pentakisphosphate kinase 2
4580	E9PGM8	2	4	2.3	1278	144399	7.9	Uncharacterized protein
4581	Q96S69	2	2	8.5	260	29060	6.5	Pex1pQ261Ter
4581	Q96S70	2	2	3.5	632	71221	6.9	Pex1pR633Ter
4581	B4DIM7	2	2	2.3	961	105955	6.3	cDNA FLJ50168, highly similar to Peroxisome biogenesis factor 1
4581	E9PE75	2	2	2.3	961	105999	6.2	Uncharacterized protein
4581	O43933	2	2	1.7	1283	142867	6.3	Peroxisome biogenesis factor 1
4582	Q6NUQ2	2	9	2.3	1002	111640	4.9	Calmin (Calponin-like, transmembrane)
4582	Q96JQ2	2	9	2.3	1002	111651	4.9	Calmin
4583	Q7Z3T8	2	2	2.3	1539	168903	4.8	Zinc finger FYVE domain-containing protein 16
4583	UPI00015CFC08	2	2	2.3	1539	168860	4.8	zinc finger FYVE domain-containing protein 16
4584	Q8IVG5	2	2	2.3	1584	184531	8	Sterile alpha motif domain-containing protein 9-like
4585	O75665-3	2	3	2.3	972	111745	5.9	Isoform 3 of Oral-facial-digital syndrome 1 protein
4585	A8K2T9	2	3	2.2	1011	116543	6.1	cDNA FLJ78705, highly similar to sapiens oral-facial-digital syndrome 1 (OFD1), mRNA
4585	O75665	2	3	2.2	1012	116671	6.1	Oral-facial-digital syndrome 1 protein
4586	O60382	2	2	2.2	1791	191306	11.9	KIAA0324 (Fragment)
4586	Q9UQ35	2	2	1.4	2752	299616	12.1	Serine/arginine repetitive matrix protein 2
4587	Q14004-2	2	3	2.3	1452	158434	9.7	Isoform 2 of Cyclin-dependent kinase 13
4587	Q14004	2	3	2.2	1512	164922	9.7	Cyclin-dependent kinase 13
4588	Q5VU97	2	6	2.2	1274	142289	6.4	VWFA and cache domain-containing protein 1
4589	Q86U86-6	2	9	3.5	856	99142	8.2	Isoform 6 of Protein polybromo-1
4589	UPI00018814F4	2	9	2.8	1085	126292	6.9	UPI00018814F4 UniRef100 entry
4589	E7EVG2	2	9	2.1	1461	168505	6.6	Uncharacterized protein
4589	Q86U86-9	2	9	1.9	1609	184041	6.7	Isoform 9 of Protein polybromo-1
4589	Q86U86-7	2	9	1.9	1597	183884	6.9	Isoform 7 of Protein polybromo-1
4589	Q86U86-5	2	9	1.9	1582	182117	6.8	Isoform 5 of Protein polybromo-1
4589	Q86U86-4	2	9	1.9	1582	181084	6.9	Isoform 4 of Protein polybromo-1
4589	Q86U86-3	2	9	1.9	1602	183689	6.7	Isoform 3 of Protein polybromo-1
4589	Q86U86-8	2	9	1.8	1652	189643	7	Isoform 8 of Protein polybromo-1
4589	Q86U86-2	2	9	1.8	1634	187187	6.8	Isoform 2 of Protein polybromo-1
4589	Q86U86	2	9	1.8	1689	192946	6.9	Protein polybromo-1
4590	Q8NG08	2	2	2.1	1087	123252	5.9	DNA helicase B
4591	C9J840	2	2	2.5	987	113561	6.8	Uncharacterized protein
4591	UPI000198C5A4	2	2	2.4	1056	121375	7.8	UPI000198C5A4 UniRef100 entry
4591	Q5MD60	2	2	2.4	1042	119405	6.6	Mitogen-activated protein kinase kinase kinase kinase 4 isoform
4591	E7EX83	2	2	2.4	1038	119000	6.9	Uncharacterized protein
4591	E7ENQ1	2	2	2.2	1154	132166	7.5	Uncharacterized protein
4591	UPI000198C5A2	2	2	2.1	1170	134171	8	UPI000198C5A2 UniRef100 entry
4591	UPI000014131B	2	2	2.1	1166	133488	7.7	mitogen-activated protein kinase kinase kinase kinase 4 isoform 1
4591	UPI000013E7B3	2	2	2.1	1166	133571	7.9	Mitogen-activated protein kinase kinase kinase kinase 4 (EC 2.7.11.1) (MAPK/ERK kinase kinase kinase 4) (MEK kinase kinase 4) (MEKKK 4) (HPK/GCK-like kinase HGK) (Nck-interacting kinase).
4591	UPI000003E780	2	2	2.1	1212	138505	7.5	mitogen-activated protein kinase kinase kinase kinase 4 isoform 3
4591	O95819-4	2	2	2.1	1165	133401	7.7	Isoform 4 of Mitogen-activated protein kinase kinase kinase kinase 4
4591	O95819-2	2	2	2.1	1211	138417	7.5	Isoform 2 of Mitogen-activated protein kinase kinase kinase kinase 4
4591	E7ESS2	2	2	2.1	1187	136117	7.7	Uncharacterized protein
4591	B7Z388	2	2	2	1279	145900	7.9	cDNA FLJ56564, highly similar to Mitogen-activated protein kinase kinase kinase 4 (EC 2.7.11.1)
4591	O95819	2	2	2	1239	142101	7.5	Mitogen-activated protein kinase kinase kinase kinase 4
4591	E9PH72	2	2	2	1265	145188	7.4	Uncharacterized protein
4591	E7EN19	2	2	2	1272	144917	7.5	Uncharacterized protein
4591	B7Z3V5	2	2	2	1235	141097	7.5	cDNA FLJ60617, highly similar to Mitogen-activated protein kinase kinase kinase 4 (EC 2.7.11.1)
4591	UPI000014130C	2	2	1.9	1320	151083	7.6	Mitogen-activated protein kinase kinase kinase kinase 4 (EC 2.7.11.1) (MAPK/ERK kinase kinase kinase 4) (MEK kinase kinase 4) (MEKKK 4) (HPK/GCK-like kinase HGK) (Nck-interacting kinase).
4591	O95819-3	2	2	1.9	1319	150913	7.4	Isoform 3 of Mitogen-activated protein kinase kinase kinase kinase 4
4592	Q13017	2	2	2	1502	172459	6.6	Rho GTPase-activating protein 5
4592	Q13017-2	2	2	2	1501	172331	6.6	Isoform 2 of Rho GTPase-activating protein 5
4593	O75179-5	2	7	3.1	751	77825	4.8	Isoform 5 of Ankyrin repeat domain-containing protein 17
4593	B5MEE3	2	7	1.9	1181	124716	5	Uncharacterized protein
4593	O75179-3	2	7	1.9	1187	124637	4.9	Isoform 3 of Ankyrin repeat domain-containing protein 17
4593	C9JRC1	2	7	1.9	1186	124494	4.9	Uncharacterized protein
4593	O75179-4	2	7	1.1	2010	210591	6.5	Isoform 4 of Ankyrin repeat domain-containing protein 17
4593	E7EWI7	2	7	1.1	2010	210532	6.4	Uncharacterized protein
4593	UPI00002263B1	2	7	1	2352	246748	6.7	ankyrin repeat domain-containing protein 17 isoform b
4593	O75179-2	2	7	0.9	2602	274185	6.5	Isoform 2 of Ankyrin repeat domain-containing protein 17
4593	O75179	2	7	0.9	2603	274256	6.5	Ankyrin repeat domain-containing protein 17

Table S1. Proteins identified in the OVCAR-3 tumor supernatant by two or more unique peptides.

Protein or Protein Group	Accession Number	Unique Peptide Count	Spectrum Count	Sequence Coverage (%)	Length	MolWt	pI	Descriptive Name
4594	P42695	2	2	1.9	1498	168890	7.5	Condensin-2 complex subunit D3
4594	UPI000047C8C7	2	2	1.9	1494	168072	7.5	UPI000047C8C7 UniRef100 entry
4595	Q5SRE5-2	2	2	2	1638	182292	6.8	Isoform 2 of Nucleoporin NUP188 log
4595	Q5SRE5	2	2	1.9	1749	196041	6.7	Nucleoporin NUP188 log
4596	Q9HCE0	2	2	1.9	2579	292479	6.4	UPF0493 protein KIAA1632
4597	A6QRH2	2	2	1.8	1266	142698	6.2	Plexin B2
4597	O15031	2	2	1.3	1838	205126	6.2	Plexin-B2
4598	Q8N201	2	3	1.8	2190	244295	6.1	Integrator complex subunit 1
4598	A4D212	2	3	1.7	2394	265754	6.7	DKFZP586J0619 protein
4598	UPI0001AE702D	2	3	1.7	2404	266207	6.7	Integrator complex subunit 1 (Int1).
4598	A4D213	2	3	1.7	2408	266641	6.7	DKFZP586J0619 protein
4599	Q7RTP6-4	2	2	1.9	966	109075	8	Isoform 4 of Protein MICAL-3
4599	Q7RTP6-2	2	2	1.9	948	106837	8.2	Isoform 2 of Protein MICAL-3
4599	E9PEF0	2	2	1.9	966	109047	7.9	Uncharacterized protein
4599	Q7RTP6-3	2	2	1.8	976	109911	8.4	Isoform 3 of Protein MICAL-3
4599	B2RXJ5	2	2	1.7	1073	121173	8.6	MICAL3 protein
4599	Q7RTP6	2	2	0.9	2002	224293	5.6	Protein MICAL-3
4600	UPI0001881A7B	2	3	1.9	1254	134592	5.7	pleckstrin logy domain containing, family G (with RhoGef domain) member 2
4600	Q9H7P9-3	2	3	1.8	1357	144720	5.7	Isoform 3 of Pleckstrin logy domain-containing family G member 2
4600	Q9H7P9	2	3	1.7	1386	147969	5.9	Pleckstrin logy domain-containing family G member 2
4601	A8MXE8	2	2	3.4	942	101428	5.5	Uncharacterized protein
4601	E7ETW1	2	2	3.3	966	104303	5.5	Uncharacterized protein
4601	Q7Z5J4-3	2	2	2	1640	175342	9.1	Isoform 3 of Retinoic acid-induced protein 1
4601	E7EVZ3	2	2	2	1640	175376	9.1	Uncharacterized protein
4601	Q7Z5J4-2	2	2	1.7	1862	198390	8.9	Isoform 2 of Retinoic acid-induced protein 1
4601	Q7Z5J4	2	2	1.7	1906	203350	8.8	Retinoic acid-induced protein 1
4601	C9JWB0	2	2	1.7	1862	198465	8.9	Uncharacterized protein
4601	A8MTC3	2	2	1.6	1993	212244	8.9	Uncharacterized protein
4602	UPI0001DD21C3	2	6	1.6	1661	177912	6	absent in melanoma 1-like protein
4603	Q8IZH2	2	2	1.5	1706	194106	7.2	5'-3' exoribonuclease 1
4603	UPI0000480180	2	2	1.5	1693	192713	7.3	5""-3"" exoribonuclease 1 isoform b
4603	Q8IZH2-2	2	2	1.5	1694	192842	7.3	Isoform 2 of 5'-3' exoribonuclease 1
4604	Q9UG01-2	2	2	1.8	1525	172437	6.2	Isoform 2 of Intraflagellar transport protein 172 log
4604	Q9UG01	2	2	1.5	1749	197575	6.1	Intraflagellar transport protein 172 log
4605	Q9Y3S1-3	2	2	4.1	779	84868	5.4	Isoform 3 of Serine/threonine-protein kinase WNK2
4605	C9JGD6	2	2	4.1	779	84710	5.3	Uncharacterized protein
4605	A6PVV2	2	2	4.1	790	85882	5.3	WNK lysine deficient protein kinase 2 (Fragment)
4605	A6PVR3	2	2	1.4	2219	234470	5.9	WNK lysine deficient protein kinase 2 (Fragment)
4605	UPI00001B09BB	2	2	1.4	2217	234378	5.8	serine/threonine-protein kinase WNK2
4605	Q9Y3S1-4	2	2	1.4	2254	238276	5.9	Isoform 4 of Serine/threonine-protein kinase WNK2
4605	Q9Y3S1-2	2	2	1.4	2224	234946	5.9	Isoform 2 of Serine/threonine-protein kinase WNK2
4605	Q9Y3S1	2	2	1.4	2297	242673	6.1	Serine/threonine-protein kinase WNK2
4606	B4DG55	2	2	1.4	1306	148375	8.4	cDNA FLJ53905, highly similar to Phosphatidylinositol-4-phosphate 3-kinase C2domain-containing alpha polypeptide (EC 2.7.1.154)
4606	O00443	2	2	1.1	1686	190678	8	Phosphatidylinositol-4-phosphate 3-kinase C2 domain-containing subunit alpha
4607	Q13395	2	5	1.4	1621	181674	7.1	Probable methyltransferase TARBP1
4608	Q9BU62	2	2	9.1	339	36378	9.7	BAT2L protein
4608	Q5JSZ5-5	2	2	2	1535	165526	8.8	Isoform 1 of Protein PRRC2B
4608	Q5JSZ5	2	2	1.4	2229	242964	8.3	Protein PRRC2B
4609	Q96PE2	2	2	1.4	2063	221671	6.3	Rho guanine nucleotide exchange factor 17
4610	Q5DT20	2	4	1.3	2850	282360	10	Hornerin
4610	Q86YZ3	2	4	1.3	2850	282389	10	Hornerin
4611	O95425-2	2	2	1.5	1788	200846	6.9	Isoform 2 of Supervillin
4611	O95425	2	2	1.2	2214	247744	7	Supervillin
4612	B7ZM65	2	5	1.1	1645	178188	9	PHRF1 protein
4612	Q9P1Y6-3	2	5	1.1	1648	178578	8.9	Isoform 3 of PHD and RING finger domain-containing protein 1
4612	Q9P1Y6	2	5	1.1	1649	178665	8.9	PHD and RING finger domain-containing protein 1
4612	E9PJ24	2	5	1.1	1645	178216	9	Uncharacterized protein
4612	B7ZM66	2	5	1.1	1647	178477	9	PHRF1 protein
4613	Q59FF5	2	2	1.3	1409	164920	9	Myosin Va variant (Fragment)
4613	A8CDT9	2	2	1	1828	212257	8.6	MYO5A variant protein
4613	UPI000188136E	2	2	1	1855	215389	8.5	myosin-Va isoform 1
4613	Q9Y4I1-3	2	2	1	1880	218606	8.5	Isoform 3 of Myosin-Va
4613	Q9Y4I1-2	2	2	1	1828	212271	8.6	Isoform 2 of Myosin-Va
4613	Q9Y4I1	2	2	1	1855	215403	8.5	Myosin-Va
4613	E7ERV4	2	2	1	1852	215045	8.5	Uncharacterized protein
4614	P78559	2	4	0.9	2803	305484	4.9	Microtubule-associated protein 1A
4614	UPI00015DFCF5	2	4	0.9	2805	305671	4.9	Microtubule-associated protein 1A (MAP 1A) (Proliferation-related protein p80) [Contains: MAP1 light chain LC2].
4614	P78559-2	2	4	0.9	2805	305671	4.9	Isoform 2 of Microtubule-associated protein 1A
4615	Q04721	2	4	0.9	2471	265403	5.1	Neurogenic locus notch log protein 2
4616	Q461N2	2	2	1	1995	222935	7.1	Ciprofibrate bound protein p240 isoform PRIC320-2
4616	B7ZML1	2	2	0.7	2897	326006	7	CHD9 protein
4616	Q3L8U1-3	2	2	0.7	2882	324127	6.9	Isoform 3 of Chromodomain-helicase-DNA-binding protein 9
4616	Q3L8U1-2	2	2	0.7	2881	324056	6.9	Isoform 2 of Chromodomain-helicase-DNA-binding protein 9
4616	Q3L8U1	2	2	0.7	2897	326022	7	Chromodomain-helicase-DNA-binding protein 9
4617	Q9C0G6	2	2	0.7	4158	475986	6	Dynein heavy chain 6, axonemal
4618	UPI0001AE79C6	2	4	0.6	5706	590788	6.7	Mucin-5B precursor (Mucin-5 subtype B, tracheobronchial) (High molecular weight salivary mucin MG1) (Sublingual gland mucin).
4618	Q9HC84	2	4	0.6	5762	596344	6.6	Mucin-5B
4618	E9PBJ0	2	4	0.6	5765	596691	6.7	Uncharacterized protein
4618	A7Y9J9	2	4	0.5	6207	648809	6.8	Mucin 5AC, oligomeric mucus/gel-forming
4618	E7EVQ7	2	4	0.5	6205	648695	6.8	Uncharacterized protein
4619	E9PFR5	2	2	0.4	4588	506278	5	Uncharacterized protein
4619	UPI0000418CED	2	2	0.4	4591	506583	5	Cadherin-related tumor suppressor log precursor (Protein fat log).
4619	Q14517	2	2	0.4	4590	506283	5	Protocadherin Fat 1
