# Supplementary material for: Identification of Multiple Novel Protein Biomarkers Shed by Human Serous Ovarian Tumors into the Blood of Immunocompromised Mice and Verified in Patient Sera
Source: PLoS One. 2013 Mar 27;8(3):e60129. doi: 10.1371/journal.pone.0060129 (PMC3609810; doi:10.1371/journal.pone.0060129)
Supplement: Table S2 — Sample classification for ovarian cancer patient sera. (PDF) [file pone.0060129.s003.pdf]

Table S2. Sample classification for ovarian cancer patient sera.

Sample Code	Age	Sample Classification	Grade	Histotype[1]	4h LC-MS/MS Pools	MRM Pools
WCS-02	56	Normal				Pool N
WCS-04	50	Normal				Pool N
WCS-12	57	Normal				Pool N
WCS-13	73	Normal				Pool N
WCS-14	55	Normal				Pool N
WCS-15	54	Normal				Pool N
WCS-16	60	Normal				Pool N
WCS-18	53	Normal				Pool N
WCS-20	68	Normal				Pool N
B23-01		Benign			Pool B	Pool B
B25-01		Benign			Pool B	Pool B
B70-01		Benign			Pool B	Pool B
B77-01		Benign			Pool B	Pool B
B79-01		Benign			Pool B	Pool B
B80-01		Benign			Pool B	Pool B
B81-01		Benign			Pool B	Pool B
B82-01		Benign			Pool B	Pool B
B83-01		Benign			Pool B	Pool B
B78-01		Benign				Pool B
471	58	Stage 1	3	UN		Pool E
484	72	Stage 1		OT		Pool E
522	46	Stage 1	3	SP		Pool E
537	66	Stage 1	1	EN		Pool E
542	60	Stage 1	2	MC		Pool E
545	55	Stage 1	3	SP		Pool E
546	37	Stage 1	1	UN		Pool E
547	50	Stage 1	1	SP		Pool E
550	27	Stage 1	0	EN		Pool E
554		Stage 1				Pool E
608	69	Stage 1	2	EN		Pool E
624	54	Stage 1	1	MC		Pool E
626	55	Stage 1	3	SP		Pool E
454	61	Stage 2	1	SA		Pool E
504	54	Stage 2	3	SP		Pool E
555	59	Stage 2	3	SP		Pool E
580	64	Stage 2	2	UN		Pool E
627	81	Stage 2	3	SP		Pool E
475	50	Stage 3	3	EN	Pool C1	Pool L
476	28	Stage 3	1	MU	Pool C1	Pool L
478	80	Stage 3	3	SP	Pool C1	Pool L
536	28	Stage 3	0	SP	Pool C1	Pool L
539	60	Stage 3	3	SP	Pool C1	Pool L
541	69	Stage 3		SP	Pool C1	Pool L
543	51	Stage 3	2	SP	Pool C1	Pool L
553	54	Stage 3	3	SP	Pool C1	Pool L
577	41	Stage 3	3	UN	Pool C1	Pool L
487	62	Stage 3	3	UN	Pool C2	Pool L
497	66	Stage 3	3	SP	Pool C2	Pool L
500	49	Stage 3	3	SP	Pool C2	Pool L
502	64	Stage 3	3	SP	Pool C2	Pool L
503	63	Stage 3	3	SP	Pool C2	Pool L
505	67	Stage 3	3	UN	Pool C2	Pool L
508	68	Stage 3	3	SP	Pool C2	Pool L
511	70	Stage 3	3	SP	Pool C2	Pool L
514	53	Stage 3	3	SP	Pool C2	Pool L
509		Stage 3				Pool L
521	54	Stage 3	3	EN		Pool L
551		Stage 3	3			Pool L
558	61	Stage 3	3	SP		Pool L
579	74	Stage 3	3	SP		Pool L
599	73	Stage 3	3	SP		Pool L
600	46	Stage 3	3	AD		Pool L
602	56	Stage 3	3	UN		Pool L
604	38	Stage 3	0	SP		Pool L
605	52	Stage 3	3	CC		Pool L
607	57	Stage 3	3	SP		Pool L
474	66	Stage 4	3	SP	Pool C3	
482	68	Stage 4	2	SP	Pool C3	
510	70	Stage 4	3	SP	Pool C3	
517	57	Stage 4	3	SP	Pool C3	
557	67	Stage 4	3	UN	Pool C3	

[1] AD: Serous pappilary adenocarcinoma; CC: Clear cell; EN: Endometroid; MC: Mucinous; MU: Mullerian; OT: Other non-epithelial; UN: Undifferentiated; SA: Sarcoma; SP: Serous papillary
