# Supplementary material for: Identification of Multiple Novel Protein Biomarkers Shed by Human Serous Ovarian Tumors into the Blood of Immunocompromised Mice and Verified in Patient Sera
Source: PLoS One. 2013 Mar 27;8(3):e60129. doi: 10.1371/journal.pone.0060129 (PMC3609810; doi:10.1371/journal.pone.0060129)
Supplement: Table S3 — Peptide transitions monitored by MRM. (PDF) [file pone.0060129.s004.pdf]

Table S3. Peptide transitions monitored by MRM.

					Transition			Area				Normalized Amount			
Accession	Gene Name	Protein Description	Peptide Sequence	Precursor Charge	Q1	Q3	CE	Normal	Benign	Early	Late	Normal (N)	Benign B	Early (E)	Late (L)
P18065	IGFBP2	Insulin-like growth factor-binding protein 2	MPCAELVR	2	496.24	587.35	32.7	1066368	1592517	1984164	4200943	0.48	0.72	0.90	1.90
P18065	IGFBP2	Insulin-like growth factor-binding protein 2	MPCAELVR	2	488.24	587.35	32.4	207894	297010	392773	1308117	0.38	0.54	0.71	2.37
P18065	IGFBP2	Insulin-like growth factor-binding protein 2	LEGEACGVYTPR	2	676.32	692.37	35.2	1127067	1593738	2020452	4359782	0.50	0.70	0.89	1.92
P18065	IGFBP2	Insulin-like growth factor-binding protein 2	TPCQQELDQVLER	3	539.26	644.37	26.9	696899	832768	984816	1888070	0.63	0.76	0.89	1.72
P18065	IGFBP2	Insulin-like growth factor-binding protein 2	HGLYNLK	2	422.74	585.28	24.1	411038	625671	757242	1624984	0.48	0.73	0.89	1.90
Normalized Protein Amount												0.52	0.72	0.88	1.88
Q14508	WFDC2 (HE4)	WAP four-disulfide core domain protein 2	EGSCPQVNINFPQLGLCR	3	697.00	843.45	29.5	0	0	0	133199	0.00	0.00	0.00	4.00
Q14508	WFDC2 (HE4)	WAP four-disulfide core domain protein 2	DQCQVDSQCPGQMK	2	848.84	576.28	39.4	0	0	0	31256	0.00	0.00	0.00	4.00
Q14508	WFDC2 (HE4)	WAP four-disulfide core domain protein 2	VSCVTPNF	2	462.22	547.25	19.5	5555	0	46985	215360	0.08	0.00	0.70	3.22
Normalized Protein Amount												0.03	0.00	0.23	3.74
P02750	LRG1	Leucine-rich alpha-2-glycoprotein	ENQLEVLEVSWLHGLK	3	632.01	640.87	32	27779779	23079851	35225497	49782419	0.82	0.68	1.04	1.47
P02750	LRG1	Leucine-rich alpha-2-glycoprotein	ALGHLDLSGNR	2	576.81	661.33	29.6	64419206	50012202	80760604	109000000	0.85	0.66	1.06	1.43
P02750	LRG1	Leucine-rich alpha-2-glycoprotein	ALGHLDLSGNR	3	384.88	484.75	18.5	161000000	142000000	206000000	236000000	0.86	0.76	1.11	1.27
P02750	LRG1	Leucine-rich alpha-2-glycoprotein	DLLLPQPDLR	2	590.34	725.39	24.1	268000000	213000000	300000000	400000000	0.91	0.72	1.02	1.35
P02750	LRG1	Leucine-rich alpha-2-glycoprotein	VAAGAFQGLR	2	495.28	620.35	20.7	69795002	50142589	76442196	111000000	0.91	0.65	0.99	1.44
Normalized Protein Amount												0.87	0.69	1.04	1.39
O00468	AGRN	Agrin	SAGDVDTLAFDGR	2	662.31	678.36	34.7	21240	20463	23125	57167	0.70	0.67	0.76	1.87
O00468	AGRN	Agrin	ALQSNHFELSLR	3	472.25	615.81	19.3	81869	60144	76506	116087	0.98	0.72	0.91	1.39
O00468	AGRN	Agrin	AYGTGFVGCLR	2	600.80	604.32	30.5	37500	22925	56351	83947	0.75	0.46	1.12	1.67
Normalized Protein Amount												0.81	0.62	0.93	1.65
Q9UL46	PSME2	Proteasome activator complex subunit 2	QNLFQEAEEFLYR	2	843.91	927.46	39.2	25152	123990	58615	122028	0.31	1.50	0.71	1.48
Q9UL46	PSME2	Proteasome activator complex subunit 2	QNLFQEAEEFLYR	3	562.94	598.33	22.2	70762	461121	196726	408713	0.25	1.62	0.69	1.44
Q9UL46	PSME2	Proteasome activator complex subunit 2	CGFLPGNEK	2	511.24	544.27	27.3	200948	1403150	544058	800572	0.27	1.90	0.74	1.09
Q9UL46	PSME2	Proteasome activator complex subunit 2	VEAFQTTISK	2	562.30	895.49	27.1	14019	76818	46152	60069	0.28	1.56	0.94	1.22
Q9UL46	PSME2	Proteasome activator complex subunit 2	DEAAYGELR	2	512.24	637.33	25.3	130474	893079	331613	681241	0.26	1.75	0.65	1.34
Q9UL46	PSME2	Proteasome activator complex subunit 2	AFYAELYHIISSNLEK	3	633.33	840.44	26	181002	987970	403268	1169079	0.26	1.44	0.59	1.71
Normalized Protein Amount												0.27	1.63	0.72	1.38
P60174	TPI1	Triosephosphate isomerase	HVFGESDELIGQK	2	729.86	1222.60	35.1	16610	300536	150648	110683	0.11	2.08	1.04	0.77
P60174	TPI1	Triosephosphate isomerase	HVFGESDELIGQK	3	486.91	558.36	20.1	357306	4395308	2130746	1155653	0.18	2.19	1.06	0.58
P60174	TPI1	Triosephosphate isomerase	VVFEQTK	2	425.74	652.33	22.2	1344835	13244452	6559759	8390093	0.18	1.79	0.89	1.14
P60174	TPI1	Triosephosphate isomerase	VVLAYEPVWAIGTGK	2	801.95	928.53	37.7	444647	3170742	2063707	3075644	0.20	1.45	0.94	1.41
P60174	TPI1	Triosephosphate isomerase	SNVSDAVAQSTR	2	617.80	661.36	29.1	228593	2044587	1042127	1371255	0.20	1.75	0.89	1.17
Normalized Protein Amount												0.17	1.85	0.96	1.01
O95865	DDAH2	N(G),N(G)-dimethylarginine dimethylaminohydrolase 2	GAEIVADTFR	2	539.78	609.30	22.3	96778	277216	316482	320085	0.38	1.10	1.25	1.27
O95865	DDAH2	N(G),N(G)-dimethylarginine dimethylaminohydrolase 2	TVVAGSSDAAQK	2	567.29	763.36	25.3	17841	54920	62043	65848	0.36	1.09	1.24	1.31
O95865	DDAH2	N(G),N(G)-dimethylarginine dimethylaminohydrolase 2	LSDVTLVPVSCSELEK	3	592.64	629.35	23.8	8959	31544	43731	40851	0.29	1.01	1.40	1.31
Normalized Protein Amount												0.34	1.07	1.30	1.30
P17900	GM2A	Ganglioside GM2 activator	EVAGLWIK	2	458.27	616.38	21.4	181886	103882	140836	282443	1.03	0.59	0.79	1.59
P17900	GM2A	Ganglioside GM2 activator	TYGLPCHCPFK	3	460.55	558.26	16.6	1225603	594958	1463196	1802724	0.96	0.47	1.15	1.42
P17900	GM2A	Ganglioside GM2 activator	IESVLSSSGK	2	503.77	764.41	23	1288466	466123	1077162	1377983	1.22	0.44	1.02	1.31
Normalized Protein Amount												1.07	0.50	0.99	1.44
P31946	YWHAB	14-3-3 protein beta/alpha	AVTEQGHELSNEER	3	533.59	714.82	43.7	703517	5297758	2833637	3450420	0.23	1.72	0.92	1.12
P31946	YWHAB	14-3-3 protein beta/alpha	VISSIEQK	2	452.26	691.36	19.1	2834603	18189412	9621293	14512282	0.25	1.61	0.85	1.29
P31946	YWHAB	14-3-3 protein beta/alpha	YLSEVASGDNK	2	591.79	690.34	30.2	60829	517712	276247	395114	0.19	1.66	0.88	1.26
Normalized Protein Amount												0.22	1.66	0.89	1.22

Table S3. Peptide transitions monitored by MRM.

Accession	Gene Name	Protein Description	Peptide Sequence	Precursor Charge	Transition Q1	Transition Q3	CE	Area Normal	Area Benign	Area Early	Area Late	Normalized Amount Normal (N)	Normalized Amount Benign B	Normalized Amount Early (E)	Normalized Amount Late (L)
Q04917	YWHAH	14-3-3 protein eta	AVTELNEPLSNEDR	2	793.89	830.40	39.4	135890	774538	580709	918029	0.23	1.29	0.96	1.52
Q04917	YWHAH	14-3-3 protein eta	ELETVCNDVLSLLDK	2	874.44	1176.59	38.3	33971	109047	86337	169145	0.34	1.09	0.87	1.70
Q04917	YWHAH	14-3-3 protein eta	NCNDFQYESK	2	652.76	654.31	32.4	12340	28287	29926	39502	0.45	1.03	1.09	1.44
Q04917	YWHAH	14-3-3 protein eta	YLAEVASGEK	2	533.77	790.39	24.1	142278	1008920	792586	1246138	0.18	1.27	0.99	1.56
Q04917	YWHAH	14-3-3 protein eta	NSVVEASEAAYK	2	634.31	668.33	29.7	9050	40021	34819	46843	0.28	1.22	1.07	1.43
Normalized Protein Amount												0.29	1.18	1.00	1.53
P25786	PSMA1	Proteasome subunit alpha type-1	NQYDNDVTVWSPQGR	3	593.61	730.36	23.9	56917	238183	226500	319455	0.27	1.13	1.08	1.52
P25786	PSMA1	Proteasome subunit alpha type-1	IHQIEYAMEAVK	3	483.25	593.30	21.9	73522	192050	129185	228584	0.47	1.23	0.83	1.47
P25786	PSMA1	Proteasome subunit alpha type-1	IHQIEYAMEAVK	3	477.91	577.30	19.6	0	28381	46105	30402	0.00	1.08	1.76	1.16
P25786	PSMA1	Proteasome subunit alpha type-1	FVFDRPLPVSR	3	444.92	543.81	19.8	3544765	8988152	6862773	11598107	0.46	1.16	0.89	1.50
P25786	PSMA1	Proteasome subunit alpha type-1	ETLPAEQDLTTK	2	673.35	501.76	31.1	1208544	2950503	2238974	4122633	0.46	1.12	0.85	1.57
Normalized Protein Amount												0.40	1.16	0.94	1.50
P20618	PSMB1	Proteasome subunit beta type-1	LSEGFSIHTR	3	382.87	517.26	16.4	83046	59162	76524	151111	0.90	0.64	0.83	1.63
P20618	PSMB1	Proteasome subunit beta type-1	GAVYSFDPVGSYQR	2	773.37	806.42	42.7	285951	630046	617877	1477014	0.38	0.84	0.82	1.96
P20618	PSMB1	Proteasome subunit beta type-1	DVFISAAER	2	504.26	533.27	25	461828	1733014	2394713	4394982	0.21	0.77	1.07	1.96
P20618	PSMB1	Proteasome subunit beta type-1	DVYTGDALR	2	505.25	531.29	27	524283	512736	767559	1634706	0.61	0.60	0.89	1.90
Normalized Protein Amount												0.52	0.71	0.90	1.86
P49721	PSMB2	Proteasome subunit beta type-2	VAASNIVQMK	2	538.79	835.43	26.3	65829	47288	121125	213424	0.59	0.42	1.08	1.91
P49721	PSMB2	Proteasome subunit beta type-2	VAASNIVQMK	2	530.79	890.48	22	6634	12324	6732	10879	0.73	1.35	0.74	1.19
P49721	PSMB2	Proteasome subunit beta type-2	YYTPTISR	2	500.76	573.34	22.9	858517	797441	1375063	2646199	0.60	0.56	0.97	1.86
P49721	PSMB2	Proteasome subunit beta type-2	FILNLPTFSVR	2	653.88	706.39	28.4	823794	2317631	1345557	2866281	0.45	1.26	0.73	1.56
Normalized Protein Amount												0.55	0.77	0.92	1.76
P28070	PSMB4	Proteasome subunit beta type-4	FEGGVVIAADMLGSYGSLAR	3	671.68	810.41	45.1	0	0	15487	39081	0.00	0.00	1.14	2.86
P28070	PSMB4	Proteasome subunit beta type-4	AIHSWLTR	3	328.52	400.21	15.5	0	120293	224303	464891	0.00	0.59	1.11	2.30
P28070	PSMB4	Proteasome subunit beta type-4	QPVLSQTEAR	2	564.80	691.34	29.2	498522	540332	804003	1602987	0.58	0.63	0.93	1.86
P28070	PSMB4	Proteasome subunit beta type-4	FQIATVTEK	2	518.79	648.36	23.5	122040	372343	841132	1705739	0.16	0.49	1.11	2.24
Normalized Protein Amount												0.18	0.43	1.07	2.32
